# Unlocking Li‒S chemistry via acoustic-induced entropy-driven electrolyte

**DOI:** 10.1038/s41467-026-72486-6

**Published:** 2026-04-30

**Authors:** Kuiyou Wang, Guanwu Li, Yunfeng Zhang, Hechao Xu, Bo Zhao, Jianshuang Wei, Wenbin Kang, Dong Wang, Lixian Song, Weitao Zheng, Yingze Song, Jingyu Sun

**Affiliations:** 1 School of Materials and Chemistry, State Key Laboratory of Environment-Friendly Energy Materials, Southwest University of Science and Technology, Mianyang, China; 2 State Key Laboratory of High Pressure and Superhard Materials, School of Materials Science and Engineering, Jilin University, Changchun, China; 3 College of Energy, Soochow Institute for Energy and Materials Innovations, Jiangsu Key Laboratory of Advanced Negative Carbon Technologies, Soochow University, Suzhou, China

**Keywords:** Batteries, Batteries

## Abstract

Li‒S batteries offer a transformative alternative to present energy storage technologies. However, their practical viability is impeded by polysulfide shuttling and lithium dendrite formation. Electrolyte engineering seeks to solve these thorny issues, yet conventional approaches rely on internal, static or invasive modifications. Here we report an acoustic-induced entropy-driven electrolyte design to steer Li‒S chemistry, realizing enhanced sulfur conversion kinetics and sustained lithium working interface. With the aid of comprehensive instrumental and computational toolbox, it is shown that the entropy-driven state of the electrolyte propels the homogeneous nucleation of both sulfur and lithium species, along with dictating Li-ion desolvation process. The designed electrolyte at a lean dosage of 2.9 µL mg^–1^ readily enables a 1.1 Ah-level pouch cell with a delivered specific energy of 404.1 Wh kg^-1^ without packaging. Our electrolyte formulation concept using external field modulation offers an appealing solution to overcome key hurdles in Li–S technology toward high-performance devices.

## Abstract (web summary)

Li–S batteries typically rely on static internal modifications. Here, authors apply dynamic external acoustic modulation to create a non-equilibrium entropy-driven electrolyte, enhancing mass transfer and Li-ion desolvation to accelerate sulfur conversion and stabilize lithium negative electrode.

## Introduction

Driven by the demand for high-capacity power sources, lithium-sulfur (Li‒S) batteries are recognized as a leading technology for next-generation electronic devices, attributed to a practical energy density^1,2^. However, their real implementation faces several fundamental challenges arising from the inherent physicochemical features of Li‒S electrochemistry: (i) slow solid-solid conversion^3,4^; (ii) lithium polysulfide (LiPS) shuttle^4,5^; (iii) positive electrode volume variations^6,7^; (iv) uncontrollable Li dendrite formation^8,9^. These cumulative challenges significantly hinder the energy output and operational lifespan of Li‒S battery systems, requesting a dedicated focus on material innovation and interfacial design.

In a Li‒S battery, the electrolyte serves a critical role that extends beyond mere ion transport. It actively orchestrates sulfur conversion and mediates the transport, followed by the subsequent precipitation or decomposition of sulfur or Li species. These functions are essential to facilitate smooth electrochemical reactions^10–12^. Electrolyte engineering is a key strategy for advancing Li‒S conversion and enhancing overall battery performance. For instance, the incorporation of LiNO_3_ salt can modify solvation structures, thereby lowering the reactive activity of sulfur species. Similarly, the in situ introduction of organometallic-based molecules within the electrolyte has promoted homogeneous sulfur catalysis^13–15^. Furthermore, a recent study demonstrated the use of a homonuclear copper-based electrolyte. This approach provides active catalytic centers and optimizes interfacial contact, leading to more effective activation of the sulfur positive electrode and enhanced stabilization of the Li negative electrode as compared to congeners employing heterogeneous catalysts^16,17^.

The realm of electrolyte engineering has witnessed fruitful strategies to address the persistent issues in Li‒S battery technology. These advancements, however, have primarily relied upon internal routes, i.e., direct addition of chemical agents into the electrolyte. These additives might be irreversibly consumed during cycling, offering a static solution that cannot adapt to the dynamic operating conditions, accompanied by introducing unforeseen parasitic reactions to degrade performances over time^18–20^. In stark contrast, strategies that utilize external fields to dictate the Li‒S chemistry represent a flexible paradigm. The distinct merit of this approach lies in its ability to offer dynamic, on-demand control over the battery’s internal environment^21–23^. By being contactless and non-invasive, it preserves the pristine integrity of the electrolyte while enabling precise and targeted interventions. Nevertheless, despite its potential, the exploration is in its infancy^24–26^. The body of related research is still scarce, the fundamental mechanisms through which external electrolyte manipulation governs the complex electrochemical procedures in Li‒S systems remain elusive.

In this study, we propose an external strategy for leveraging an acoustic field to induce a state of high entropy within the electrolyte. This is obviously distinctive from conventional high-entropy systems, where entropy increases through static configurational mixing of multiple chemical constituents^27–29^. As for our electrolyte, the entropy-driven state is a dynamic, non-equilibrium phenomenon^30^. The acoustic field facilitates energy dispersal that disrupts the ordered Li-ion solvation shells, transitioning the electrolyte from a localized ordered state to a disordered, partially desolvated state. Such a modification constitutes an increase in structural entropy^31–34^, rendering the degree of disorder induced by the external field rather than compositional complexity. Our entropy-mediated regulation is expected to significantly impact the electrochemical dynamics. To elucidate the underlying mechanism as well as its impact on a Li‒S battery, a comprehensive suite of analytical techniques was employed, including synchrotron radiation X-ray three-dimensional nano-computed tomography (SR X-ray 3D nano-CT), small-angle neutron scattering (SANS), operando Raman spectroscopy, ab initio molecular dynamics (AIMD) simulations, and finite-element modeling (FEM). This work demonstrates the considerable potential of integrating external acoustic strategies into the design of advanced battery configurations.

## Results

Supplementary Fig. 1 shows a schematic of the research workflow for an ultrasonic cavitation-enhanced Li‒S battery. In this configuration, a sealed Li‒S battery is subjected to an external acoustic field generated by an ultrasonic transducer operating at a frequency exceeding 20 kHz. The transducer induces rapid bubble nucleation and collapse on a microsecond timescale. This transient cavitation process generates localized high-pressure regions and micro-jets^35^, which facilitate the formation of an entropic electrolyte. An integrated theoretical and experimental methodology is presented to elucidate two fundamental aspects. The initial focus is on the coupling mechanism between the ultrasonic field and the Li‒S system, achieved through external electrolyte modulation. The desolvation behaviors of Li species and the trajectory of Li-ion rotational motion within the ultrasonic cavitation field are systematically probed using AIMD and FEM simulations, and operando Raman spectroscopy. Moreover, the nucleation kinetics of Li_2_S and the deposition behaviors of Li in the entropy-driven electrolyte are qualitatively and quantitatively investigated using SR X-ray 3D nano-CT and SANS, respectively. SR X-ray 3D nano-CT offers advantages in terms of detection resolution, depth, and sensitivity, enabling detailed analysis of Li_2_S products post-nucleation through volume ratios and sizes. SANS, as a non-destructive and strong-penetration technique, provides insights into the accumulated Li deposition cavities. Through these integrated theoretical and experimental explorations, the mechanisms underlying the formation of entropy-driven electrolytes, localized active species nucleation, and related processes can be reasonably explained, which contributes to unveiling the fundamental working mechanisms of entropy-driven electrolytes induced by acoustic stimulation in practical Li–S battery systems.

**Fig. 1:**
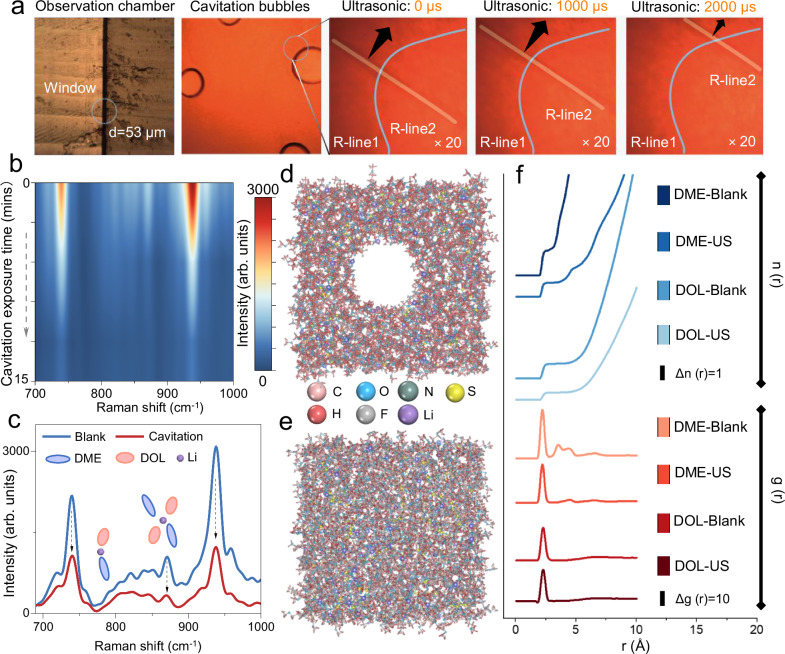
Effect of the ultrasonic field on the electrolyte. **a** Digital images from the observation window tracking cavitation bubble trajectories via the changing position of R-line2. **b** Operando Raman spectra analysis of the Li–S battery electrolyte under an ultrasonic field. **c** Comparison of Raman spectra between the blank electrolyte and the electrolyte after 60 s of ultrasonication; the inset schematically compares a solvent-rich Li⁺ complex in the blank electrolyte (right scheme) with its desolvated counterpart under ultrasonication (left scheme). Snapshots from AIMD simulations of the electrolyte (**d**) with and (**e**) without ultrasonication. **f** Corresponding radial distribution functions, *g*(*r*), and coordination numbers, *n*(*r*), derived from the simulations.

The application of an ultrasonic field promotes a dynamic, acoustic-induced entropy-driven state. This state is not merely a product of local chaotic disturbances, but is rooted in the fundamental modification of the electrolyte’s solvation structures. The primary mechanism responsible for this phenomenon is acoustic cavitation, which involves the formation, rapid growth, and violent collapse of microscopic bubbles^36^. The collapse of these cavitation bubbles generates high-energy micro-jets and shockwaves, inducing turbulent fluid motion that manifests as enhanced convective mass transfer. To visualize this process, real-time optical microscopy was employed to monitor bubble dynamics in a rhodamine B solution under an ultrasonic field (Fig. 1a and Supplementary Fig. 2). Bubbles generated by the acoustic field are clearly observed through the chamber’s viewing window (Supplementary Fig. 3 and 4). The trajectory of a representative bubble is tracked by monitoring its periphery, denoted as the R-line2. Time-resolved observations over 2000 µs reveal a steady migration of this line toward the upper right corner of the field of view. The calculated migration velocity of the bubble is 369.5 µm s^–1^. This value is substantially greater than the conventional electrolyte drift velocity of 0.1 µm s^–1^ ^37^, thereby substantiating the role of the ultrasonic field in creating an entropy-driven electrolyte characterized by cavitation-induced enhanced convective mass transfer. To ensure statistical validity, multiple independent measurements were conducted by tracking bubble trajectories within the liquid phase over a fixed time interval (Supplementary Fig. 5). The average migration speed is determined to be 398.5 μm s^–1^, which is consistent with our initial observations. The shockwaves and shear forces generated during cavitation are sufficient to overcome intermolecular forces, thereby disrupting the existing solvation shells around Li-ions. To investigate this phenomenon, operando Raman spectroscopy was utilized to monitor the dynamic Li-ion solvation environment in a representative electrolyte subjected to an ultrasonic field (Supplementary Fig. 6). The evolution of time-resolved Raman spectra, recorded over a 15-min period, reveals a progressive attenuation and shifting of peak intensities, signifying dynamic changes in the microenvironment of the resulting entropy-driven electrolyte (Fig. 1b)^38^. Notably, a prominent decrease in Raman spectral intensity was observed within the initial 60 s of sonication, which suggests a rapid and significant desolvation process induced by ultrasonic cavitation (Fig. 1c).

To further probe the dynamic behavior of the entropy-driven electrolyte, AIMD simulations were performed. The simulated electrolyte features a cavity corresponding to the low-pressure rarefaction phase of the acoustic wave (Fig. 1d, e). During this rarefaction phase, the local pressure drops below the saturated vapor pressure of the electrolyte, overcoming intermolecular forces and inducing rupture of the liquid structure. This leads to the formation of cavities with internal negative pressure^39^. In the AIMD model, a central ~20 Å void is generated to mimic this state, creating an instantaneous pressure gradient and interfacial tension. The resulting local shear forces at the cavity boundary drive the structural transition from an ordered solvated state to a disordered, partially desolvated state, providing a molecular link between macroscopic acoustic mediation and microscopic solvation modification. To elucidate the local structures of the ion-solvent complex, the radial distribution function of *g*(*r*), and coordination number of *n*(*r*), were analyzed for both entropy-driven and blank electrolyte, verifying the composition of the Li-ion solvation shell and the interactions within the Li-ion-DME/TFSI^–^/NO_3_^–^/DOL system (Fig. 1f). The analysis identifies distinct solvated structures for Li-ion, with the entropy-driven electrolyte exhibiting a less coordinated solvation structure compared to the blank electrolyte, as depicted in the insets of Fig. 1c. Furthermore, the simulations reveal that the average distance between Li-ions and the surrounding DME molecules increases under the influence of ultrasonic field. This reduction in coordination number, combined with the increased spacing within the solvation shell, substantiates the effective desolvation of Li-ion-solvent clusters. These findings validate that the intense, localized shear forces originating from cavitation profoundly alter the microscopic structure of the electrolyte. According to established theoretical frameworks, the liberation of coordinated solvent molecules into the bulk electrolyte increases the system’s disorder^40^. To quantitatively substantiate this, structural entropy (s_2_) was derived from radial distribution functions, g(r), using AIMD simulations and partitioned into specific Li^+^-DME and Li^+^-DOL correlations^41^. The data reveal that ultrasonication induces an increase in structural entropy for both interactions. The effect is more pronounced in the Li^+^-DME component (∆*s*_2_ = 6.23 *k*_B_) relative to Li^+^-DOL (2.42 *k*_B_). This heightened sensitivity in DME is consistent with its bidentate coordination, as the acoustic disruption of the chelated solvation shell is likely accompanied by enhanced configurational degrees of freedom than the monodentate DOL^42,43^. This quantified increase in structural entropy unequivocally validates that the external ultrasonic field disrupts liquid-structure correlations, effectively shifting the electrolyte from a structured solvated state toward a more randomized, “gas-like” configurational state. This field-induced entropy increase is functionally equivalent to the ‘weakened interactions’ often sought in static high-entropy electrolytes but is achieved here through external energy dispersal^44^.

**Fig. 2:**
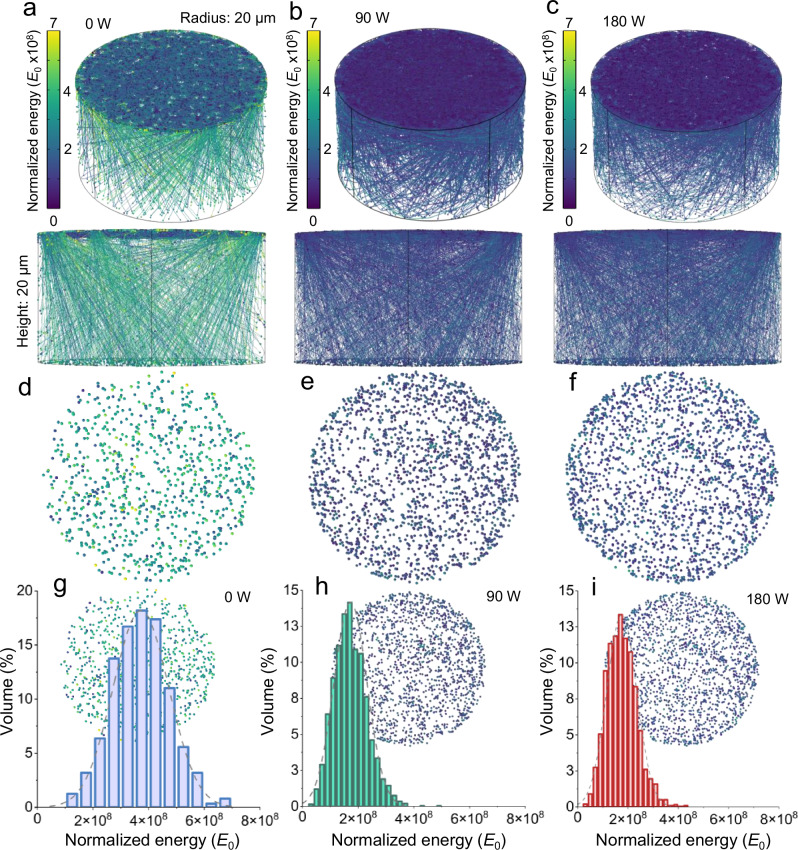
FEM simulations of Li-ion transport under various ultrasonic field strengths. **a**–**c** Simulated Li-ion movement trajectories at ultrasonic field intensities of 0, 90, and 180 W, respectively; the simulation box measures 20 µm in radius and 20 µm in height; the color scales representing the normalized energy are shared with the following plots. **d**–**f** Corresponding distribution maps of Li-ion reaching the bottom electrode surface for each condition. **g**–**i** Associated histograms showing the statistical distribution of the solvation energy for Li-ion reaching the bottom electrode surface.

To evaluate the influence of the entropy-driven electrolyte on electrochemical behavior under practical operational conditions, a 3D mass transfer model was developed using FEM. This multi-physics model was designed to simulate the transport trajectory of Li-ion within a Li‒S battery by coupling the ultrasonic, fluid, concentration, and electric fields with the electrochemical reaction. As illustrated in Supplementary Fig. 7, the model incorporates rotational interface mass transfer and electrochemical reaction kinetics. The 3D modeling domain was configured as a cylindrical structure representing the complete electrolyte region of cell. The simulated transport trajectories of Li-ion are presented for conditions without ultrasound, with low-power ultrasound, and with high-power ultrasound (Fig. 2a–c). The results demonstrate that the ultrasonic field significantly alters the transport pathways of Li-ions. Specifically, higher ultrasonic power induces a greater curvature in the rotational trajectory of the Li-ions (Supplementary Figs. 8–10). This observation suggests an enhanced motion of solvated Li-ions within the electrolyte, driven by the acoustic field.

The influence of the external acoustic field power on the normalized solvation energy and subsequent distribution of Li-ions was quantified, as illustrated in Fig. 2d–f, which maps the distribution of Li-ions arriving at the bottom electrode. An increase in ultrasonic power is found to significantly increase the flux of Li-ions reaching the reaction interface, a finding that is consistent with the enhanced convective mass transfer described previously. Furthermore, the simulations reveal a more uniform spatial distribution of Li-ions, with a notable reduction in localized ion clustering. Histograms of the Li-ion solvation energies, for those ions reaching the electrode surface, are presented in Fig. 2g–i. A distinct shift towards lower normalized solvation energies is observed in systems subjected to an ultrasonic field. Lower solvation energy implies a weaker interaction between a Li-ion and its surrounding solvation shell^45^. This is attributed to the disruption of the solvation structure by the significant shear forces generated during cavitation, resulting in a less bulky solvated complex, a conclusion consistent with the preceding spectroscopic and theoretical results. The less bulky, partially desolvated Li-ion is expected to exhibit higher mobility and undergo less hydrodynamic drag^46^. This, in turn, facilitates the transport of more ions to the electrode surface, as observed in the flux simulations (Fig. 2d–f). To experimentally validate the Li-ion mobility enhancement suggested by the computational results, the electrolyte ionic conductivity (σ) was quantified under varying ultrasonic power levels using electrochemical impedance spectroscopy. As shown in Supplementary Fig. 11, a clear positive correlation between acoustic power and ion transport was observed, with ionic conductivity increasing progressively as the ultrasonic power is raised. Additionally, concentration field simulations (Supplementary Figs. 12–14) demonstrate that the ultrasonic environment mitigates concentration polarization. Cross-sectional analysis of the Li-ion concentration shows that without ultrasound, the concentration varies from approximately 0.7 to 1.3 M. In contrast, under ultrasonic influence, this range narrows substantially to 0.96–1.04 M, reflecting a more uniform ion distribution throughout the electrolyte.

**Fig. 3:**
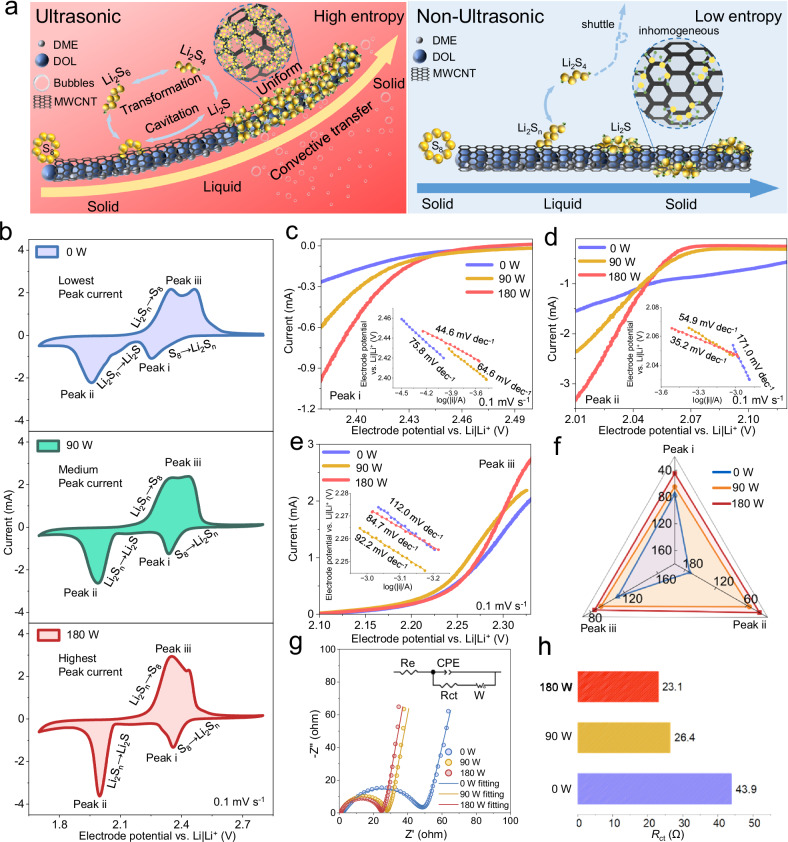
Kinetic analysis of Li‒S conversion in the entropy-driven electrolyte. **a** Schematic comparing sulfur conversion kinetics in the entropy-driven versus a conventional electrolyte. The ascending yellow arrow signifies the acoustic-induced enhancement in structural entropy achieved under ultrasonication, whereas the flat blue arrow indicates no entropy gain under conventional electrode conditions. **b** CV curves of Li‒S cells at a scan rate of 0.1 mV s⁻^1^. **c**–**e** LSV curves and the corresponding inset Tafel plots for Peaks i, ii, and iii. **f** Ternary plot comparing the calculated Tafel slopes for all three peaks. **g** EIS profiles for cells subjected to different ultrasonic field strengths; the inset shows the equivalent circuit adopted to fit the plots. **h** Comparison of the corresponding *R*_ct_ values derived from the EIS data.

The combined results from the operando Raman spectroscopy, AIMD calculations, and FEM simulations elucidate the beneficial role of an externally applied ultrasonic field. This field induces an entropy-driven state characterized by enhanced convective mass transfer, an optimized Li-ion solvation structure, and a uniform ion flux. The relationship between ultrasound-induced desolvation and enhanced mass transfer was characterized by a parallel mechanism, both originating simultaneously from the physical phenomenon of acoustic cavitation. On the macroscopic scale, the collapse of cavitation bubbles generates turbulent fluid motion, which drives enhanced convective mass transfer and ensures a high flux of active species to the electrode interface. Concurrently, on the microscopic scale, the localized shear forces produced during bubble collapse overcome the intermolecular forces within the solvation shell, resulting in a partially desolvated Li-ion structure. These two effects function in parallel to resolve different kinetic bottlenecks. By simultaneously augmenting transport and lowering the desolvation barrier, ultrasonic irradiation ensures a high flux of kinetically active species at the electrode surface. These modifications are anticipated to accelerate the reaction kinetics of the rate-limiting steps in Li‒S batteries. Specifically, the enhanced transport and modified solvation environment are expected to promote the conversion of polysulfide species into dense and uniform solid-phase products, alleviating the accumulation of soluble sulfur species at the positive electrode, thereby suppressing the shuttle effect (Fig. 3a).

Prior to electrochemical testing, the potential for cavitation-induced damage to the electrodes was evaluated. An H-type electrolytic cell was used to validate the non-destructive nature of the process on both the positive and negative electrodes. Observations confirm that even after prolonged exposure to high-power ultrasound, no stripping or delamination of active materials occurs on the electrode surfaces (Supplementary Figs. 15 and 16). The long-term mechanical stability of the cell components under sustained ultrasonic exposure was further verified across different sulfur loadings (2 and 6 mg cm^–2^). Digital photographs of the electrodes and separators harvested after 100 cycles (0.5 C) at 180 W confirm that all components maintain their structural integrity without delamination or physical damage, even at high sulfur loadings (Supplementary Fig. 17). Subsequently, the electrochemical properties of the Li‒S batteries under the influence of an ultrasonic field were investigated (Supplementary Figs. 18 and 19). The external acoustic field is applied in situ throughout the duration of the electrochemical measurements to maintain the transient cavitation state, rather than serving as a pre-treatment for the electrolyte. The enhancement provided by the acoustic field is highly dynamic and reversible, as demonstrated in Supplementary Figs. 20 and 21. Figure 3b presents the slow-scan cyclic voltammetry (CV) curves recorded at 0.1 mV s⁻^1^ under various ultrasonic power levels. The voltammograms exhibit two characteristic reduction peaks (Peak i at 2.3–2.4 V and Peak ii at 1.9–2.1 V) and one oxidation peak (Peak iii at 2.4–2.6 V). These peaks correspond respectively to the reduction of S_8_ to soluble long-chain LiPSs, the subsequent transformation to short-chain species and insoluble Li_2_S, and the reverse oxidation of discharge products back to S_8_ during charging. The CV results demonstrate that as ultrasonic power increases, the currents for all redox peaks rise significantly, reflecting a substantial enhancement in electrochemical reactivity. Furthermore, with increasing ultrasonic power, the reduction peaks shift to higher potentials while the oxidation peak shifts to lower potentials, substantiating the ultrasonic power-triggered electrochemical activity elevation.

To quantify the influence of the ultrasonic field strength on charge transfer kinetics during the various stages of LiPS conversion, a Tafel analysis was conducted (Fig. 3c–f). The results indicate a significant acceleration of kinetics for all redox processes under ultrasonic irradiation. Specifically, for the initial reduction of long-chain LiPSs, the Tafel slope decreases from a baseline of 75.8 to 64.6 mV dec⁻^1^ and 44.6 mV dec⁻^1^ under low- and high-power ultrasound, respectively. The kinetics for the critical nucleation step, involving the conversion of soluble short-chain LiPSs to Li_2_S, are similarly enhanced, with the Tafel slope decreasing from 171.0 to 54.9 mV dec⁻^1^ and 35.2 mV dec⁻^1^. During the charging cycle, the oxidation process also exhibits faster kinetics, as the Tafel slope decreases from 112.0 to 92.2 mV dec⁻^1^ and 84.7 mV dec⁻^1^. These consistent and substantial reductions in the Tafel slopes confirm that the ultrasonic field effectively realizes the kinetically boosted Li‒S redox reactions.

**Fig. 4:**
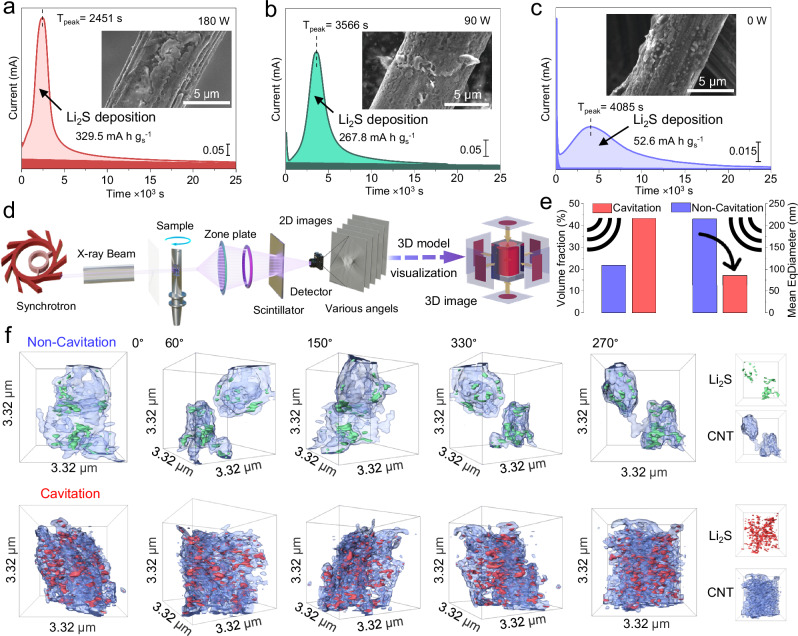
Effect of the entropy-driven electrolyte on Li_2_S nucleation. **a**–**c** Potentiostatic deposition curves for Li_2_S and corresponding SEM images of the deposits. The shaded areas represent the integrated charge capacities corresponding to the precipitation of Li_2_S. The electrolytes were subjected to an ultrasonic power of **a** 180 W, **b** 90 W, and **c** 0 W (no sonication). **d** Schematic of the SR X-ray 3D-nano-CT facility and the illustration of the 3D reconstruction process. **e** Statistical analysis of Li_2_S mean equivalent diameter (EqDiameter) and volume fraction. **f** Reconstructed 3D-nano-CT images of Li_2_S precipitates (green, red) on a carbon substrate (blue) in conventional and entropy-driven electrolytes.

Electrochemical impedance spectroscopy (EIS) tests were performed on Li–S batteries to elucidate the influence of the ultrasonic field on their charge-transport properties, with measurements conducted on the same cell under varying ultrasonic power levels (Fig. 3g). The impedance spectra were fitted using a standard Randles–type equivalent circuit (Fig. 3g inset). The diameter of the semicircle in the high-to-medium frequency region, which represents the interfacial charge transfer resistance (*R*_ct_), decreases substantially with the application of ultrasonic power. Concurrently, the slope of the low-frequency tail becomes steeper, indicating enhanced ionic transport within the battery. A quantitative analysis, shown in Fig. 4h, confirms this trend. The calculated *R*_ct_ value progressively decreases from 43.9 Ω in the absence of ultrasound to 26.4 Ω at a power of 90 W, and further to 23.1 Ω at 180 W. The detailed fitting results for each circuit element, along with the corresponding fitting errors, are provided in Supplementary Table 1. These findings corroborate that the ultrasonic field strengthens the charge transfer within the battery system.

During the sulfur species evolution process, the insulating nature of the discharge end-product Li_2_S renders the nucleation transformation of Li_2_S_4_ into Li_2_S a critical step that determines the reaction rate. Figure 4a–c shows the discharge curves of Li–S batteries under a constant cell voltage of 2.05 V, subjected to different ultrasonic field strengths. By integrating these curves, it is evident that the Li_2_S deposition capacity under a 180 W ultrasonic field reaches 329.5 mA h g_S_⁻^1^, which is significantly higher than those under low-power ultrasonic (90 W) and no ultrasonic (0 W) conditions, with capacities of 267.8 and 56.2 mA h g_S_⁻^1^, respectively. Furthermore, it is observed that as the ultrasonic power increases, the peak time is markedly advanced. In conjunction with SEM image analysis, these results indicate that the ultrasonic field accelerates the nucleation process of Li_2_S on the substrate in a manner of full coverage. To further investigate the temporal deposition behavior of Li_2_S as mediated by the acoustic field, the deposits were characterized post-mortem. The disassembled battery components were analyzed using SR X-ray 3D nano-CT, a technique with high penetration depth, as shown in Fig. 4d. By scanning at ±60° and subsequently reconstructing the data, the 3D morphology of the deposits on the substrate was obtained, enabling the evaluation of critical parameters such as the total volume proportion and mean size of Li_2_S deposits (Supplementary Fig. 22). As illustrated in Fig. 4f, the 3D reconstructions reveal the morphology of Li_2_S deposits formed without the influence of an ultrasonic field. In this environment, the Li_2_S deposits are characterized by large, block-shaped particles that display a high degree of agglomeration^47^. In contrast, in the entropy-driven electrolyte environment induced by the ultrasonic field, the Li_2_S deposits display not only a more uniform distribution but also a significantly reduced size (Supplementary Figs. 22 and 23). Based on the afore-mentioned SR X-ray 3D-nano CT results, the volume proportion and average particle size of Li_2_S deposits can be further statistically analyzed and quantitatively derived, with the detailed results presented in Fig. 4e. Under the entropy-driven electrolyte environment induced by the ultrasonic field, the volume proportion of Li_2_S significantly increases from 21.8% to 43.3%. This finding is consistent with the nucleation data obtained from the fitting of the constant voltage discharge curves described earlier, indicating that under ultrasonic conditions, the deposition capacity of the discharge final product Li_2_S is markedly higher than that of the blank group. Further analysis reveals that under the influence of the external field, the average particle size of Li_2_S deposits is 85.2 nm, which is substantially smaller than the Li_2_S deposit size in the electrolyte without the ultrasonic field (214.9 nm).

To further examine the influence of the entropy-driven electrolyte under the field effect on the Li_2_S decomposition reaction during the charging process, the decomposition curves at a constant cell voltage of 2.35 V were collected. As depicted in Supplementary Fig. 24, based on the time-dependent current variation curve during the charging process, the decomposition capacities of Li_2_S are calculated. Under the influence of 180 W high-power ultrasound, the decomposition capacity of Li_2_S reaches 413.3 mA h g_S_^–1^, which is significantly higher than that under 90 W low-power ultrasound (346.4 mA h g_S_^–1^) and in the absence of an ultrasonic field (166.5 mA h g_S_^–1^). Further disassembly of the batteries after the completion of the decomposition experiment and analysis of the electrode morphology via SEM characterization reveals that the electrode surface corresponding to the 180 W high-power ultrasound condition is smoother and cleaner, indicating more thorough Li_2_S decomposition under this condition (Supplementary Fig. 25). This result demonstrates that the ultrasonic field exhibits catalytic capability for the decomposition reaction of Li_2_S. Based on these results, it is concluded that the high-power ultrasonic field significantly enhances both the nucleation and decomposition kinetics of Li_2_S. This enhancement is attributed to the formation of an entropy-driven electrolyte with a partially desolvated Li-ion structure, marked by enhanced mass transfer, and high ionic flux. This exerts a critical, bidirectional impact on the sulfur conversion reactions during both the charge and discharge procedures. While enhanced mass transport typically aids ion supply, it theoretically poses a risk of accelerating the migration of soluble LiPSs to the negative electrode. To rigorously address this, operando Raman spectroscopy was performed at the negative electrode-separator interface during discharge. The results indicate that the ultrasonic field effectively suppresses the shuttle effect as shown in Supplementary Fig. 26.

**Fig. 5:**
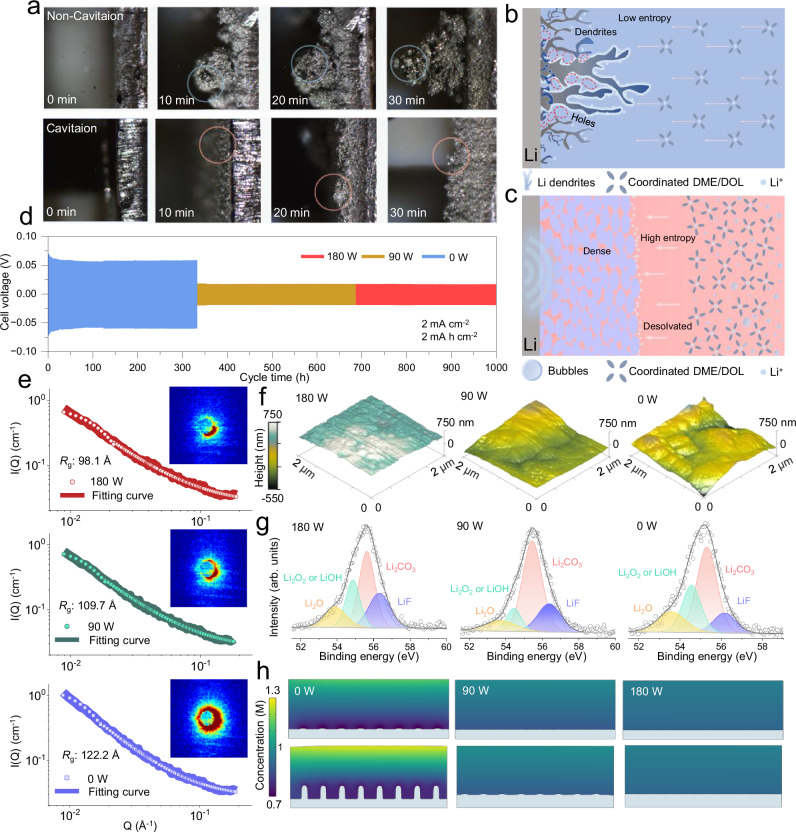
Impact of entropy-driven electrolytes on the Li electrodes. **a** In situ optical microscopy images of lithium plating in different electrolytes at 2 mA cm⁻² with time-resolved observations shown from 0 to 30 min. **b**, **c** Schematic illustrating the Li-ion desolvation and deposition process at the Li electrode interface; the blue and pink colors denote electrolytes in low- and high-entropy states, respectively. **d** Plating/stripping cycling performance of Li | |Li symmetric cells at 2.0 mA cm⁻^2^ and 2.0 mAh cm⁻^2^ within 1000 h. **e** SANS I(Q) vs. Q curves for Li electrodes after 100 plating/stripping cycles, with the inset showing the SANS scattering pattern; each individual pixel in the arrayed detector has dimensions of 8 mm. **f** AFM topography images of different Li electrodes from symmetric cells after 100 plating/stripping cycles. **g** High-resolution XPS Li *1s* spectra for different Li electrodes after 100 plating/stripping cycles. **h** FEM simulations showing Li⁺ concentration profiles and the state of Li deposition at the initial and final stage of the plating process in different electrolytes.

An in situ observation cell, integrating an ultrasonic module, was utilized to visualize the influence of the acoustic field on Li plating and stripping behavior (Supplementary Figs. 27 and 28). The results, presented in Fig. 5a, show that without ultrasonic assistance, significant Li dendrite growth occurs. In stark contrast, under the influence of the ultrasonic field, no sharp dendrites can be observed, and a flat Li working surface is maintained even after 30 min. These findings indicate that the application of ultrasound promotes uniform nucleation and growth of Li at the electrode-electrolyte interface. Based on the preceding experimental and theoretical results, a mechanism can be proposed to explain the influence of the ultrasonic cavitation field on Li-ion migration in the entropy-driven electrolyte. As illustrated in Fig. 5b and c, the process in a conventional electrolyte is contrasted with the entropy-driven environment. In the absence of ultrasound, the non-uniform distribution of Li-ion flux and strong solvation effects govern the deposition process. During plating, Li-ions preferentially deposit at regions with higher electric-field intensity, such as surface defects or weak points in the solid electrolyte interphase (SEI). This localization creates high current densities, causing the Li-ion reduction rate to exceed the local diffusion rate, which in turn leads to the formation of microscopic protrusions on the Li surface that evolves into dendrites. In the acoustic-induced entropy-driven environment, the ultrasonic cavitation modifies the Li-ion solvation structure. This effect disassembles large ion clusters and partially strips coordinated solvent molecules from the Li-ions. The resulting reduction in solvation shell significantly enhances Li-ion evolution kinetics, lowers the charge transfer resistance at the interface, and mitigates the formation of steep concentration gradients. Consequently, Li deposition proceeds more uniformly across the electrode, promoting the smooth growth of Li metal and forming a more stable working interface. This is expected to extend the cycle life of the battery and minimize the safety risks associated with internal short circuits.

To verify the afore-mentioned hypothesis, Li | |Li symmetric cell tests were conducted. As illustrated in Fig. 5d and Supplementary Fig. 29, the galvanostatic cycling performance is evaluated at a current density of 2 mA cm⁻^2^ with a capacity of 2 mA h cm⁻^2^. The overpotential during the Li plating and stripping processes decreases after the introduction of an ultrasonic field. Specifically, Li | |Li symmetric cells operated under 180 and 90 W ultrasonic field conditions exhibit stable cycling performance for over 1000 and 650 h, respectively, without significant fluctuations in overpotential, indicating highly stable Li deposition and dissolution behaviors as the ultrasonic power increases. In contrast, the cell under the 0 W condition suffers from current fluctuations and rapid short-circuit failure within 350 cycles. To systematically evaluate the influence of the ultrasonic field on plating efficiency, Li | |Cu half-cells were assembled and tested. The Coulombic efficiency (CE) of Li deposition on copper foil was assessed at a current density of 1 mA cm⁻^2^ and a capacity of 1 mA h cm⁻^2^ under three conditions: no ultrasound, a 90 W ultrasonic field, and a 180 W ultrasonic field. As presented in Supplementary Fig. 30, the results demonstrate a significant improvement in cycling stability with the application of ultrasound. Under 180 W ultrasonic conditions, the cell maintains a stable CE above 94% for over 90 cycles. In contrast, the cell without ultrasonic assistance exhibits a marked decrease in CE after approximately 50 cycles, indicating unstable Li plating and stripping behavior.

To elucidate the structure-activity relationship between the ultrasonic field and Li deposition behavior, the chemical compositions of the SEI were analyzed. Cells were disassembled after 100 cycles at 2.0 mA cm^–2^ and 2.0 mA h cm^–2^, with disassembly carried out at the symmetric cell’s near-zero cell voltage state for post-mortem analysis. The SEI layers were then characterized by X-ray photoelectron spectroscopy (XPS) combined with argon ion etching. As shown in Fig. 5g, the SEI formed under all conditions primarily comprises lithium oxides, hydroxides, carbonate, and lithium fluoride (LiF). It is established that LiF is a critical component for a stable SEI^48^. A comparison of the relative atomic concentrations (Supplementary Fig. 31) reveals a significant enrichment of LiF in the SEI formed under ultrasonic irradiation. The LiF proportion increases from 12.3% without ultrasound to 21.3% under the 90 W field, and further to 23.8% under the 180 W high-power condition. This trend is further corroborated by analysis of the high-resolution F *1 s* core-level spectra of Li electrodes cycled under ultrasonication at different power levels (Supplementary Fig. 32). This increased LiF content is likely a result of the ultrasonic field’s effect on the Li-ion solvation shell. By promoting the stripping of organic solvent molecules, the acoustic field facilitates decomposition pathways that favor the formation of LiF. To decouple the respective contributions of LiNO_3_ depletion and LiTFSI decomposition to SEI formation, the SEI compositions of Li electrodes cycled with different LiNO_3_ concentrations (0.5 and 2.0 wt%) were analyzed. As shown in Supplementary Fig. 33, depth-profiling XPS analysis after ~10 nm of etching reveals that the LiF atomic content in the dense inner SEI remains essentially unchanged (22.3 at% vs. 23.5 at%) despite the substantial difference in LiNO_3_ concentration. These results indicate that the formation of the LiF-rich interphase is predominantly governed by ultrasound-mediated desolvation and subsequent decomposition of the bulk LiTFSI salt, thereby effectively decoupling this process from the LiNO_3_ additive. The resulting LiF-rich layer constitutes a robust and stable SEI to the battery system, which in turn promotes the flatter and denser Li deposition morphology observed.

To quantitatively evaluate surface flatness after Li deposition, the morphologies of Li electrodes cycled in symmetric cells for 100 cycles at 2.0 mA cm^–2^ and 2.0 mA h cm^–2^ were characterized by atomic force microscopy (AFM), with electrodes harvested at the symmetric cell’s near-zero cell voltage state for post-mortem analysis. As illustrated in Fig. 5f, in the absence of an ultrasonic field, the surface of Li electrodes after cycling exhibits pronounced local sharp protrusions. In contrast, under low-power ultrasonic conditions, the surface morphology of Li electrodes following Li plating and stripping cycling shows noticeable improvement compared to the non-ultrasonic sample, with a tendency toward greater overall uniformity. Under high-power ultrasonic conditions, facilitated by enhanced convective mass transfer and optimized solvation structure, the surface of Li electrodes displays a highly uniform state. These findings further confirm that the ultrasonic field exerts a significantly regulated influence on Li-ion deposition and dissolution behaviors. A quantitative statistical analysis was performed on the AFM data (Supplementary Fig. 34). As depicted in Supplementary Fig. 35, the introduction of an ultrasonic field can improve the Li surface states. Notably, the surface of the high-power ultrasonic sample exhibits remarkable flatness. This finding is further corroborated by the roughness statistics presented in Supplementary Fig. 36.

The surface morphologies of the Li electrodes were characterized post-cycling using SEM, with the results presented in Supplementary Fig. 37. The electrode subjected to long-term ultrasound (180 W) is free of fracture and damage. In the absence of an ultrasonic field, the electrode surface exhibits a sharp and disordered morphology characteristic of typical Li dendrite formation. In contrast, under low-power ultrasonic conditions, the formation of sharp dendrites is mitigated, and the deposition morphology begins to change toward spherical structures; however, prominent pore structures remain observable. When high-power ultrasound is applied, the Li electrode produces a dense and smooth surface morphology, indicating the effective suppression of dendrite growth.

SANS was employed to characterize the nanostructure of the deposited Li layer after 100 plating/stripping cycles at 2.0 mA cm^–2^ and 2.0 mA h cm^–2^, with disassembly carried out at the symmetric cell’s near-zero cell voltage state. This technique offers significant advantages in non-destruction and detection depth over traditional imaging methods. The radius of gyration (*R*_g_), calculated from the SANS data, provides a measure of the average size of inhomogeneities, such as spatial voids, within the Li deposition layers. As shown in Fig. 5e, the *R*_g_ value for the Li layer formed with the 180 W entropic electrolyte is 98.1 Å. This is notably smaller than the values obtained for the 90 W (109.7 Å) and 0 W (122.2 Å) conditions. This decrease in *R*_g_ suggests that the application of an ultrasonic field to the electrolyte promotes the formation of more compact Li structures. Such a morphology is indicative of more uniform and smooth Li plating and stripping behavior.

Subsequently, the finite-element method was utilized to simulate the Li-ion deposition process at the working interface between the electrolyte and the Li electrode under varied ultrasonic field strengths (Supplementary Figs. 38–40). The FEM results are presented in Fig. 5h, where the color scale represents the Li-ion concentration distribution within the electrolyte during the deposition process. As the deposition proceeds, in the electrolyte without ultrasonic treatment, the concentration polarization becomes more pronounced, leading to the gradual formation of dendritic structures on the electrode surface. In contrast, under entropy-driven electrolyte conditions, the degree of Li-ion concentration polarization at the interface is significantly mitigated. Notably, under the 180 W high-power ultrasonic condition, the working surface of the Li electrode remains relatively flat throughout the deposition process. While ultrasonication does not fundamentally alter the static electric-field distribution dictated by electrode geometry, it profoundly reshapes the dynamic deposition landscape. By sustaining a high Li-ion flux to the interface, modifying the Li-ion solvation structure, and promoting the formation of a robust LiF-rich SEI, the acoustic field homogenizes Li deposition. These combined effects lower the nucleation barrier and diminish sensitivity to local electric-field heterogeneity, thereby enabling uniform, dendrite-free Li growth, consistent with the preceding findings.

**Fig. 6:**
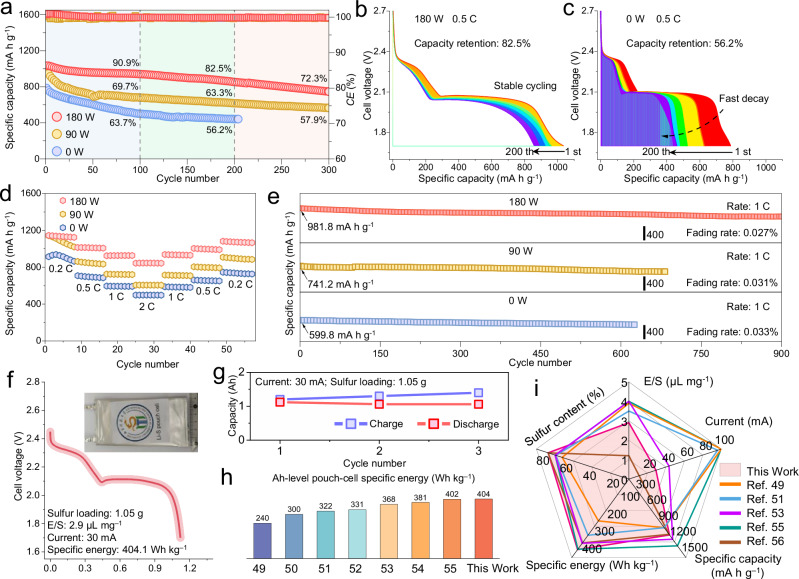
Electrochemical performance of Li‒S batteries with entropy-driven electrolytes. **a** Cycling stability at 0.5 C; 1 C = 1672 mA g⁻^1^. Galvanostatic discharge curves for the first 200 cycles at 0.5 C for cells with electrolytes prepared using **b** 180 W and **c** 0 W of ultrasonic power. **d** Rate capabilities and **e** long-term cycling performance for cells using electrolytes prepared with 180, 90, and 0 W of sonication. **f** Digital photographs and discharge curve for a high-loading (1.05 g sulfur) multilayer stacked pouch cell, achieving a specific energy of 404.1 Wh kg⁻^1^. **g** Cycling performance of the high-loading stacked pouch cell. Comparison of **h** specific energy and **i** other critical performance metrics of this work and prominent literature reports; the source of the literature data shown in these figures can be found in Supplementary Information, Tables 3 and 4.

To assess the performance impact of the entropy-driven electrolyte state induced by ultrasound, coin-type Li‒S batteries with sulfur loadings of approximately 2.0 mg cm⁻^2^ were tested under various ultrasonic power levels. First, the cell polarization, defined as the cell voltage difference between the charging and second discharge plateaus (Supplementary Fig. 41), was analyzed. This polarization is primarily constrained by the conversion of Li_2_S (Supplementary Fig. 42). A statistical analysis of the overpotential at a 0.5 C rate (Supplementary Fig. 43) reveals a clear trend: the cell under the 180 W high-power condition exhibits the overpotential of 23.1 mV, the value of which is lower than the 24.2 mV observed at 90 W and the 29.3 mV without ultrasound. This result confirms that the acoustically stimulated, entropy-driven electrolyte enhances the electrochemical kinetics of the Li‒S system. Subsequently, the cycling performance at a 0.5 C rate was evaluated (Fig. 6a–c), with a zoomed-in view of the CE variation provided in Supplementary Fig. 44. The cell operating under the 180 W condition delivers an initial discharge capacity of 1034.7 mA h g⁻^1^ and maintains 853.6 mA h g⁻^1^ after 200 cycles, corresponding to a capacity retention of 82.5%. In contrast, under the 90 W condition, the initial capacity is 976.3 mA h g⁻^1^, with the retention dropping to 63.3%. Without ultrasonic assistance, the performance is further diminished, with an initial capacity of 785.8 mA h g⁻^1^ and a final retention of only 56.2% after 200 cycles.

The rate performance of the battery was evaluated at various rates (0.2, 0.5, 1.0, and 2.0 C) to assess the performance of the entropy-driven electrolyte under different operating conditions. As shown in Fig. 6d, under the 180 W environment, the discharge specific capacities of the battery at each rate are 1144.9, 1013.9, 924.8, and 843.4 mA h g⁻^1^, respectively. In contrast, under the 90 W condition, the corresponding discharge specific capacities are slightly lower, measuring 1142.0, 862.1, 725.2, and 606.1 mA h g⁻^1^, respectively. As a control, in the absence of an ultrasonic field, the rate performance of the battery is significantly reduced, with discharge specific capacities of only 914.2, 707.8, 595.5, and 497.7 mA h g^–1^, respectively. These results indicate that as ultrasonic power increases, the discharge specific capacity of the battery exhibits a gradually increasing trend. Following cycling, the working interface of the electrode was further examined using SEM. As observed from the microscopic morphologies of the front and cross-section in Supplementary Figs. 45 and 46, the electrode structure under the field influence is well-preserved, with the surface developing a noticeably denser and more uniform morphology. Conversely, the electrode without ultrasonic treatment exhibits noticeable cracks and collapse, resulting from inhomogeneous reaction processes and uncontrolled volume expansion, which severely compromises the operational stability of practical Li–S batteries. To validate the long-term and high-rate cycling performance, Li‒S batteries were tested at a 1.0 C rate under various ultrasonic field strengths (Fig. 6e and Supplementary Fig. 47). The battery operating under the 180 W ultrasonic field demonstrates enhanced stability, maintaining capacity for over 900 cycles. The capacity retention at the 200th, 400th, 600th, and 800th cycles is 89.0%, 85.9%, 83.1%, and 76.5%, respectively. This corresponds to a low average capacity decay rate of only 0.027% per cycle over 900 cycles, a performance that significantly surpasses the comparison samples. These results further confirm that the entropy-driven electrolyte, which promotes the efficient conversion of sulfur and Li species, has a beneficial regulatory effect on the charge and discharge processes. This mechanism is key to ensuring the long-term cycling stability of Li‒S batteries, particularly at high rates. To determine the upper limit of the beneficial acoustic field, the ultrasonic power was increased to 210 W. Experimental results indicate that 180 W represents the optimal threshold for the current cell configuration (Supplementary Figs. 48 and 49). To assess the mechanical robustness of the battery architecture under the 180 W ultrasonic field, devices across a range of positive electrode areal loadings and E/S ratios were systematically evaluated as shown in Supplementary Fig. 50.

To evaluate the performance of the entropy-driven electrolyte concept in a practical application, a pouch cell was assembled (Supplementary Fig. 51). Digital photographs and performance data for the assembled Li‒S pouch cell are presented in Supplementary Fig. 52 and 53. Operating under the influence of the ultrasonic field, the high-loading single-layer pouch cell (5.2 mg cm^–2^) delivers a high initial specific capacity of 1129.5 mA h g⁻^1^ at 0.5 C. It demonstrates stable performance for 150 cycles, exhibiting a low average capacity decay rate of only 0.15% per cycle. The charge/discharge curves were also extracted for the pouch cell at the 1st (initial), 75th (mid-cycle), and 150th (end-of-life) cycles (Supplementary Fig. 54). The comparison reveals that the voltage plateaus remain stable within 150 cycles, confirming the reversibility even at a high loading density, enabled by the ultrasonic field. The optimized ultrasonic power of 180 W also does not compromise the pouch-cell positive electrode interface even at this high loading density (Supplementary Fig. 55). These results indicate that the ultrasonic-induced, entropy-driven approach is robust, meeting the demanding requirements of high sulfur loading and low electrolyte-to-sulfur ratios. This demonstrates a strong potential for practical, industrial-scale applications of the technology. To demonstrate scalability, an Ah-level Li‒S stacked pouch cell with a total sulfur loading of 1.05 g was assembled. This stacked pouch cell was designed to maximize specific energy under extreme conditions, featuring a high positive electrode loading (9.4 mg cm^–2^), a thin Li reservoir (50 μm), and a lean electrolyte condition (E/S = 2.9 μL mg^–1^). Aluminum and nickel tabs are ultrasonically welded to the positive electrode and negative electrode, respectively, and the electrodes are sealed in an aluminum-laminated film (Supplementary Fig. 56). The pouch cell exhibits favorable electrochemical performance under the influence of the ultrasonic field, as shown by its charge-discharge voltage profiles (Fig. 6f, g). At a current of 30 mA, the cell delivers a total cell capacity of 1.12 Ah, marking a significant advancement from the milliampere-hour level typical of lab-scale cells to the Ah-level required for practical applications. The total energy of the battery reached 2.4 Wh, translating to a specific energy of 404.1 Wh kg^–1^ based on the stack mass (excluding the Al-plastic film housing, Supplementary Fig. 57). To provide a strictly practical evaluation, the energy density was also calculated using the total cell weight, including the Al-plastic film. (Detailed weight breakdown in Supplementary Fig. 58) The efficacy of the acoustically induced, entropy-driven strategy is further substantiated through comparison with a stacked pouch-cell control device prepared under identical parameters except employing a conventional electrolyte without acoustic treatment, as shown in Supplementary Fig. 59. Such a pouch cell exhibits a markedly reduced discharge capacity, an unstable discharge plateau, and consequently a lower specific energy compared to the cell utilizing the entropy-driven electrolyte. These results clearly demonstrate the potential of the acoustically induced approach as a critical enabler for the operation of Li–S batteries under demanding conditions. As in Fig. 6h, i and Supplementary Table 2‒4, this high-specific-energy performance is highly competitive with previously reported state-of-the-art Li‒S cells^49–56^. This performance is attributed to the ultrasonically stimulated, entropy-driven state of the electrolyte, which enables a more thorough and reversible conversion of the active species at both the sulfur positive electrode and the Li negative electrode. The performance of the scaled-up stacked pouch cell confirms the significant enhancement afforded by the ultrasonic external field on Li‒S cell operation. To provide a proof-of-concept for its practical application, the assembled pouch cell was used to power a toy boat. As depicted in Supplementary Figs. 60 and 61, the cell successfully powers the electronic gadget in an ultrasonic environment for over 10 min, demonstrating that the Li‒S system, when assisted by ultrasonic cavitation, holds distinct practical value. To further enhance practical relevance, potential engineering routes for integrating ultrasonic enhancement into commercial battery systems are outlined. Rather than the bulky probe-type sonicators commonly used in laboratory studies, compact thin-film piezoelectric transducers could be laminated onto pouch-cell surfaces or embedded between cells, enabling efficient acoustic coupling with minimal impact on volumetric energy density. In addition, for electric-vehicle battery packs employing liquid cooling, the coolant itself could serve as an acoustic transmission medium, allowing ultrasonication to be delivered through cooling plates for concurrent electrochemical regulation and thermal management without significant architectural modification.

## Discussion

An approach is developed that couples an external acoustic field with the complex chemistry of Li‒S batteries to resolve the persistent issues of sluggish sulfur conversion and Li dendrite growth. Through a combined investigation using AIMD and FEM simulations with operando Raman spectroscopy, it is established that an ultrasonic field enhances convective mass transfer with facilitated ion flux. Furthermore, the Li-ion solvation structure is found to be disrupted by the partial stripping away of solvent molecules. Advanced characterizations, including SR X-ray 3D nano-CT and SANS, unequivocally verify that this acoustically stimulated entropy-driven electrolyte steers the reversible conversion of sulfur species and facilitates dendrite-free Li metal growth. The practical viability of this mechanism is demonstrated in a 1.1 Ah-level pouch cell, which achieves a specific energy of 404.1 Wh kg⁻^1^ without packaging under a lean electrolyte condition of 2.9 μL mg⁻^1^. This study examines the multiscale effects of applying an external acoustic field and presents a broadly applicable strategy for mitigating major bottlenecks in Li‒S batteries.

## Methods

### Ultrasonic field generation

An ultrasonic cavitation field was generated using an LC-JY92-IIDN cell disruptor (Shanghai Lichen Technology) operating at a frequency greater than 20 kHz. The system was equipped with power gradient probes to accommodate different sample types. To eliminate potential thermal runaway risks, a closed-loop control system was implemented to maintain the cell temperature at 28 °C (Supplementary Fig. 62).

### Material characterizations

The morphologies of the materials were characterized using a Zeiss Sigma 500 SEM. The surface chemical compositions were determined by XPS using a Thermo Scientific K-Alpha spectrometer. Furthermore, AFM images were obtained with a Cypher ES Atomic Force Microscope to investigate the surface topography. In particular, for the ex situ XPS and AFM measurements, the samples were encapsulated in aluminum-plastic laminated films within an Ar-filled glovebox. The hermetically sealed samples were then transferred to Ar-filled acrylic jars to maintain an inert atmosphere prior to XPS and AFM analysis.

Operando Raman measurements were conducted using a confocal Raman spectroscopic system (Horiba LabRAM HR Evolution). The system was equipped with a 532 nm excitation laser (100 mW) and a back-illuminated deep-depletion CCD detector. The operando experiments were performed using a specialized in situ electrochemical cell featuring a quartz window (ALSBs-Raman cell, Beijing Scistar Tech. Co., Ltd.). To capture the evolution of the electrolyte structure, testing was conducted by directly adding the electrolyte into the operando cell during assembly, with an external ultrasonic cavitation device applied. To track the real-time evolution of polysulfides, the assembly process of the Raman cell was conducted in alignment with standard coin-cell protocols.

To investigate the dynamic evolution of lithium deposition and dendrite growth, in situ optical microscopy was conducted using a YUESCOPE YM710TR microscope integrated with a LIB-MS-I in situ imaging system. A Li | |Li symmetric cell was constructed by encapsulating two Li foil electrodes with dimensions of 1*1 cm^2^ within a transparent electrochemical cell featuring a high-purity quartz window. After the injection of the electrolyte, the cell was connected to an electrochemical workstation for galvanostatic deposition at a constant current density of 2 mA cm⁻^2^. The morphological evolution of the lithium deposition interface was captured in transmission optical mode at a high-speed frame rate of 500 fps.

### Li_2_S nucleation/decomposition

The catholyte (0.2 M Li_2_S_8_ solution) was prepared in a high-purity Ar-filled glovebox (water <0.01 ppm, O_2_ < 0.01 ppm). Analytical-grade sulfur (Beijing Deke Daojin) and Li_2_S (Shanghai Macklin) were added to anhydrous 1,2-dimethoxyethane (DME, Shanghai Aladdin) at a 7:1 stoichiometric molar ratio. The mixture was rapidly stirred in a flask until a complete color change was observed. The resulting electrolyte was used immediately after preparation. The anolyte, purchased from Canrd Technology Co. (MA-EN-ET-043602), consists of a 1.0 M LiTFSI solution in a 1:1 (v/v) mixture of 1,2-dimethoxyethane (DME) and 1,3-dioxolane (DOL), with an addition of 2.0 wt.% lithium nitrate (LiNO_3_). The anolyte was used as received. Carbon paper (CP, FM-EM-GD-0009, Canrd Technology Co.; thickness: 0.19 mm; porosity: 78%) was used as the current collector. It was cut into disks with a diameter of 13 mm. Subsequently, 20 mg of multi-walled carbon nanotubes (MWCNTs, XFNANO, purity 95%, diameter 5–15 nm, length 10–30 μm) were dispersed in ethanol and deposited onto the CP disks to form the CP-MWCNT positive electrodes. Electrochemical cells were assembled using the prepared CP-MWCNT as the positive electrode and Li foil (MA-EN-CC-001303, Canrd Technology Co.; purity >99.9%) as the negative electrode. The electrolytes were transferred to the cell using disposable polypropylene pipette tips in the Ar-filled glovebox.

For the Li_2_S nucleation tests, the assembled cells were first discharged galvanostatically at a current of 0.112 mA to a voltage of 2.06 V. This was followed by a potentiostatic discharge at 2.05 V until the current decreased below 10⁻^5^ A. These tests were conducted under various ultrasonic environments. The nucleation capacity of Li_2_S was calculated by integrating the area under the current-time curve, in accordance with Faraday’s Law. For the Li_2_S decomposition tests, cells were fabricated following the same assembly procedure. To ensure the complete conversion of LiPSs to Li_2_S, the cells were initially discharged galvanostatically at 0.112 mA until the voltage dropped below 1.7 V. Subsequently, the cells were charged potentiostatically at 2.35 V until the current fell below 10⁻^5^ A.

### Lithium plating/stripping

Cu (MA-EN-CU-000201, purity >99.8%) was procured from Canrd Technology Co., and used as received for electrochemical cell fabrication, without additional rolling or compression. The metal electrodes were prepared immediately prior to cell assembly. Li | |Cu asymmetrical cells were assembled utilizing Cu and Li foils (MA-EN-CC-001303). 20 μL of the 1.0 M LiTFSI electrolyte (MA-EN-ET-043602) was applied and transferred to the cell using disposable polypropylene pipette tips in the Ar-filled glovebox. The electrochemical performance of these cells was evaluated using a Neware Battery Measurement System. This evaluation involved a galvanostatic plating process where Li was deposited onto the Cu foil at a current density of 1.0 mA cm⁻^2^ for a total capacity of 1.0 mA h cm⁻^2^ (geometric area: 1.91 cm^2^). Subsequently, a stripping process was conducted at the same current density of 1.0 mA cm⁻^2^. Li | |Li symmetrical cells were also assembled. These cells were constructed with two Li foil electrodes, serving as both the working and counter electrodes. The cycling protocol for the symmetrical cells involved galvanostatic plating and stripping at specified current densities (geometric area: 1.91 cm^2^). The coulombic efficiency is calculated as the ratio of discharge capacity divided by the charge capacity in the preceding charge cycle.

### Battery performance tests

MWCNTs and sulfur powder were mixed at a mass ratio of 1:3 using a dual asymmetric centrifugal mixer (Thinky, ARE-310) for 30 min. The obtained mixture was subsequently collected and further ground for 30 min to ensure homogeneous dispersion. The ground composite was then transferred into a weighing bottle, sealed, and heated at 155 °C for 6 h in a DHG-9053A drying oven to obtain the composite electrode material (S@MWCNT). Subsequently, the as-prepared composite (80 wt.%), Super P (10 wt.%), and PVDF binder (10 wt.%) were dispersed in analytical-grade NMP solvent (Chengdu Kelong Chemical) and homogenized using the mixer to form a uniform slurry (with a sulfur content of 60 wt.%). The slurry was then uniformly coated onto an aluminum current collector (MA-EN-CU-000101, purity >99.6%, Canrd Technology Co.) using an AFA-HC100 doctor blade coater at 50 °C. The sulfur loading on the electrode could be adjusted by controlling the blade thickness and coating cycles. After coating, the electrodes were maintained at 50 °C on the hot plate for approximately 20 min, followed by drying at 60 °C for 12 h in a vacuum oven (DZF-6030) to ensure complete solvent removal. The dried electrodes were then punched into circular disks with a diameter of 13 mm using an MSK-T10 manual cutter for CR2032 coin-cell assembly. Prior to assembly, the prepared electrodes were stored in the Ar-filled glovebox (water <0.01 ppm, O_2_ < 0.01 ppm). The standard CR2032 coin cell measures 20 mm in diameter and 3.2 mm thick, with both its outer casing and internal spring made of stainless steel. The coin-type cells with an areal mass loading of around 2.0 mg cm^–2^ were fabricated using the prepared composite electrode as the positive electrode, Li metal foil as the negative electrode (N/P ratio of 90.9), and Celgard 2500 polypropylene (PP) film with a diameter of 19 mm as the separator (25 µm in thickness, porosity of 55%, average pore size of 0.064 µm). The separator wetting procedure involved applying 1.0 M LiTFSI electrolyte (MA-EN-ET-043602) sequentially to the positive electrode and the PP separator (transferred via polypropylene pipette tips) before Li anode placement, maintaining an E/S ratio of 15.0 μL mg⁻¹.

Single-layer pouch cells were similarly prepared, using single-sided electrodes (3 cm * 3 cm) with a loading density of 5.2 mg cm⁻^2^ and a separator measuring 5.5 cm * 6.5 cm. The N/P ratio was set to 2.63, and the E/S ratio was maintained at 7.8 μL mg⁻^1^. The pouch cell was pre-sealed on three sides, leaving a single port for electrolyte injection. A precise volume of electrolyte was introduced, followed by vacuum-assisted infiltration to ensure thorough wetting of the porous sulfur cathode. Finally, the filling port was vacuum-sealed to hermetically isolate the cell. The stacked pouch cells featured double-sided electrodes with a loading density of 9.4 mg cm⁻^2^ per side. Specific configuration parameters for both the single-layer and stacked pouch cells are summarized in Supplementary Table 5.

All electrochemical measurements were conducted at a constant temperature of 28 °C within a thermostatic test chamber. Galvanostatic charge/discharge cycling, as well as rate and cycling performance evaluations, were performed on a Neware Battery Measurement System over a cell voltage range of 1.7–2.8 V. Further electrochemical characterization, including CV and EIS, was carried out using a Metrohm Autolab G204 Electrochemical Workstation. CVs were performed within a potential range of 1.7 V–2.8 V vs. Li|Li^+^ at a constant scan rate of 0.1 mV s⁻^1^. To investigate the reaction kinetics, Tafel plots were derived from the LSV data, specifically focusing on the linear regions adjacent to the prominent redox peaks identified in the CV profiles. Potentiostatic EIS measurements were conducted at 28 °C under open-circuit conditions, with the frequency range set from 100 kHz to 0.01 Hz at a voltage amplitude of 5 mV and 10 points per decade. The EIS spectra were analyzed by fitting the experimental data using ZView software. The refined parameters for each circuit element, including the associated fitting errors, are summarized in Supplementary Table 1. The ionic conductivity of the electrolyte was also characterized using EIS in a symmetric blocking electrode configuration. The 1 M LiTFSI electrolyte (MA-EN-ET-043602) was applied. To perform the measurement, CR2032-type coin cells were assembled by sandwiching a Celgard 2500 separator between two stainless steel electrodes. A total of 50 µL electrolyte was employed, carefully dispensed onto each side of the separator to ensure uniform wetting. Potentiostatic EIS measurements were conducted under open-circuit conditions, with the frequency range set from 100 kHz to 1 Hz at a voltage amplitude of 5 mV and 10 points per decade. The bulk resistance was determined from the high-frequency intercept on the Nyquist plot.

The C-rate is defined as 1 C = 1672 mA g⁻^1^. For calculations of specific current and specific capacity, the mass basis refers specifically to the sulfur active material, which constitutes 60 wt% of the composite positive electrode. The full composition and weight breakdown of the electrode are detailed above. Specific energy is defined as the ratio of total cell energy to the mass of the cell components excluding packaging. This calculation accounts for the cumulative mass of the current collectors, positive and negative electrodes, electrolyte, and separator. Additionally, the specific energy inclusive of packaging is also reported. A comprehensive weight breakdown for all components is provided in Supplementary Figs. 57 and 58.

### SR X-ray 3D-nano-CT tests

SR X-ray 3D-nano-CT tests were performed at the BL07W beamline of the National Synchrotron Radiation Laboratory (NSRL) in Hefei, China. The beamline provided a photon flux of 2 × 10^10^ Phs s⁻^1^ and a spatial resolution of 30 nm. For the analysis, cells previously subjected to Li_2_S nucleation tests were disassembled in a pure argon atmosphere. Samples were encapsulated in aluminum-plastic laminated films within the Ar-filled glovebox. The hermetically sealed samples were then transferred using Ar-filled acrylic jars to maintain an inert atmosphere before the SR X-ray 3D-nano-CT test. The powder sample was then carefully deposited onto a carbon-free formvar film (100 mesh) and dried at room temperature. The film loaded with the sample was then transferred into the instrument’s vacuum chamber for imaging. Two-dimensional (2D) tomographic projections were acquired over a tilt-angle range of −60° to +60°, with an angular step of 1° and an exposure time of 2 s per projection. The collected 2D tomograms were subsequently reconstructed into 3D volumes using the Xradia XRM Reconstructor software. Further 3D visualization and quantitative segmentation of the reconstructed volumes were conducted using Avizo Fire VSG software.

### SANS tests

For post-mortem analysis, Li metal electrodes were harvested from cycled Li | |Li symmetric cells. The harvested electrodes were first washed with DME and subsequently dried under the ambient conditions of the Ar-filled glovebox. To prevent atmospheric contamination during analysis, the prepared samples were hermetically sealed within an aluminum-plastic laminated film. The sealed samples were then transferred using Ar-filled acrylic jars to maintain an inert atmosphere. SANS measurements were then conducted using the SANS-Suanni spectrometer at the China Mianyang Research Reactor (CMRR). The analysis utilized a neutron wavelength (λ) of 0.53 nm. The raw two-dimensional scattering data were subsequently processed and converted into one-dimensional intensity profiles of scattering intensity *I*(Q) versus *q* using the BerSANS software package.

### FEM simulations

Finite element analysis was employed to simulate the distribution of Li-ion concentration within the electrolyte during battery operation. The computational domain was modeled as a cylinder, geometrically representing the electrolyte confined between the negative electrode at the upper boundary and the positive electrode at the lower boundary. To account for the scale disparity between the microscopic cavitation bubbles and the overall domain, a dispersed two-phase flow model was employed (Supplementary Fig. 63). Boundary conditions for current density were defined in accordance with the actual galvanostatic discharge tests. The simulation was executed to calculate the temporal and spatial evolution of the Li-ion concentration profile throughout the electrolyte domain. This analysis was performed for two distinct scenarios: one representing standard discharge and another incorporating the effects of applied ultrasonic waves, thereby enabling a comparative assessment of its impact on ion transport.

The propagation equation of ultrasonic waves in the electrolyte is described by Eq. (1).

1 $${\nabla }^{2}p=\frac{1}{{v}^{2}}\frac{{\partial }^{2}p}{\partial {t}^{2}}$$

Here, v represents the speed of sound in the electrolyte. At this point, the pressure change caused by the electrolyte can be regarded as a time-varying function, which can be expressed by Eq. (2).

2 $$p\left(t\right)={p}_{0}+\Delta p\sin \left(\omega t\right)$$

Substituting the pressure into the Navier–Stokes equation yields Eq. (3)

3 $$\frac{\partial {{\rm{u}}}}{\partial t}+{{\rm{u}}}\cdot \nabla {{\rm{u}}}=-\frac{1}{\rho }\nabla \left({p}_{0}+\Delta p\sin \left(\omega t\right)\right)+\frac{\mu}{\rho} {\nabla }^{2}{{\rm{u}}}$$

The convective flow field induced by ultrasound was first determined. This calculation solves for the flow velocity (u) as a function of time (*t*), based on the physical properties of the liquid electrolyte, including its density (ρ), pressure (*p*), and dynamic viscosity (μ). It is noted that, while individual ultrasonic cavitation events, including bubble formation/collapse, are inherently random at the microscale, their collective behavior in an ensemble leads to well-defined macroscopic phenomena. Such microscale randomness is reconciled in this model by coupling the bubble-generated impacts as an averaged term into the Navier–Stokes equations to solve for the velocity distribution, as demonstrated in Supplementary Fig. 64. With the convection field established, the total mass transfer flux for Li-ions is described by Eq. (4).

4 $${J}_{i}=-{D}_{i}\nabla {c}_{i}-\frac{{z}_{i}F}{{RT}}{D}_{i}{c}_{i}\nabla \varphi+{c}_{i}{{\rm{u}}}$$

The total flux of reactive ions, denoted as *J*, is determined by considering contributions from both diffusion and migration. This relationship is described by the Nernst-Planck equation. In this context, *D* represents the diffusion coefficient of the reactive ions, *C* is their concentration, and $\varphi$ is the local electric potential. Subsequently, the rate of the Faradaic reaction at the electrode-electrolyte interface is calculated using the Butler–Volmer equation, which is presented as Eq. 5.

5 $${i}_{l}={i}_{0}\left\{\exp \left(\frac{\alpha F\eta }{{RT}}\right)-{c}_{{\mathrm{i}}}\exp \left(\frac{-\beta F\eta}{{RT}}\right)\right\}$$

In the equation, ${i}_{l}$ is the local current density of the electrode, ${i}_{0}$ is the exchange current density, $\alpha$ and $\beta$ is the reaction exchange coefficient, $\eta$ is the overpotential of the reaction. Then, the coupling of the Faraday reaction and concentration is realized based on Eq. (6).

6 $${i}_{l}=F{J}_{i}$$

The ${i}_{l}$ and ${J}_{i}$ in the equation obtained from the above two equations are applied to derive the ion concentration near the electrode.

### DFT-based molecular dynamics simulations

First-principles calculations were performed using the Vienna Ab initio Simulation Package (VASP). The exchange-correlation interactions were described by the Perdew–Burke–Ernzerhof (PBE) functional within the generalized gradient approximation (GGA). The projector augmented-wave (PAW) method was employed to represent the core-valence electron interactions, with a kinetic energy cutoff of 450 eV applied to the plane-wave basis set. For structural optimizations, the Brillouin zone was sampled using a Γ-centered 1 × 1 × 1 Monkhorst–Pack k-point mesh. All atomic positions were fully relaxed until the energy and interatomic forces converged to within 1 × 10^–6^ eV per atom and 0.01 eV Å⁻^1^, respectively. An on-the-fly machine learning force field was utilized, trained via ab initio DFT calculations. These calculations employed the PBE functional with DFT-D3 dispersion corrections to accurately capture the van der Waals interactions critical for solvation dynamics. The system was equilibrated in the canonical ensemble (NVT) at a constant temperature. The structural models for the simulation were constructed using Materials Studio software. The electrolyte model comprised a mixed solvent system of DOL, DME, LiNO_3_, and LiTFSI salt at a concentration of 1 M. The simulation box was constructed with a Li-ion: solvent molecular ratio of approximately 1:7.6, containing around 100 Li-ions, 75 TFSI^−^, 25 NO_3_^−^, 454 DOL, and 306 DME molecules, with the atomic coordinates reported in Supplementary Data files.

### Structural entropy analysis

The two-body correlation contribution to the excess entropy (*s*_2_), also referred to as “structural entropy,” is employed as a predictor of dynamic behavior in non-equilibrium systems^57–60^. In this work, *s*_2_ is quantified from the radial distribution functions g(r) obtained via AIMD simulations, according to:

7 $${s}_{2}=-2\pi \rho {k}_{B}{\int }_{0}^{\infty }\left[g\left(r\right)\log \left(g\left(r\right)\right)-g\left(r\right)+1\right]{r}^{2}{dr}$$

where $\rho$ denotes the number density, *k*_B_ is the Boltzmann constant, and r represents the separation distance.

## Supplementary information

Supplementary Information

Description of Additional Supplementary Files

Supplementary Data

Transparent Peer Review file

## Source data

Source Data

## Data Availability

The data supporting the findings of this work are available within the article and its Supplementary Information. Source data are provided with this paper.
