# Supplementary material for: Unlocking Li‒S chemistry via acoustic-induced entropy-driven electrolyte
Source: Nat Commun. 2026 Apr 30;17:5915. doi: 10.1038/s41467-026-72486-6 (PMC13338023; doi:10.1038/s41467-026-72486-6)
Supplement: Supplementary file 1 — Supplementary Information [file 41467_2026_72486_MOESM1_ESM.pdf]

Supplementary Information

Unlocking Li–S chemistry via acoustic-induced entropy-driven electrolyte

Kuiyou Wang[1†], Guanwu Li[2†], Yunfeng Zhang[1], Hechao Xu[1], Bo Zhao[1], Jianshuang Wei[3], Wenbin Kang[1]*, Dong Wang[2]*, Lixian Song[1], Weitao Zheng[2], Yingze Song[1]*, and Jingyu Sun[3]*

[1]School of Materials and Chemistry, State Key Laboratory of Environment-Friendly Energy Materials, Southwest University of Science and Technology, Mianyang 621010, China.

[2]State Key Laboratory of High Pressure and Superhard Materials, School of Materials Science and Engineering, Jilin University, Changchun 130013, China.

[3]College of Energy, Soochow Institute for Energy and Materials Innovations, Jiangsu Key Laboratory of Advanced Negative Carbon Technologies, Soochow University, Suzhou 215006, China.

*Corresponding author. E-mail: yzsong@swust.edu.cn (Y.S.); sunjy86@suda.edu.cn (J.S.); wenbin.kang@swust.edu.cn (W. K.); wangdong2023@jlu.edu.cn (D.W.).

†These authors contributed equally to this work.

Supporting Figures

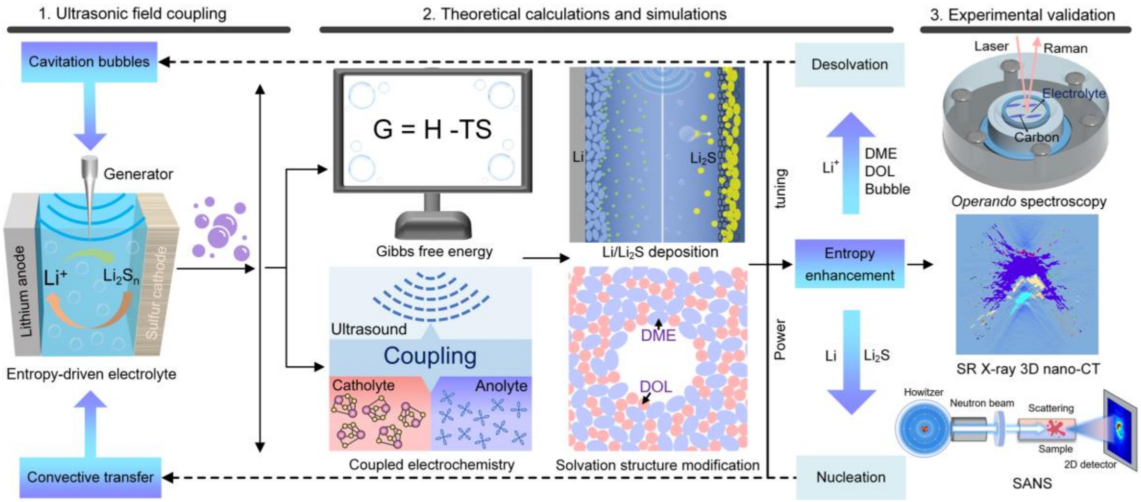

Supplementary Fig. 1|A workflow diagram illustrating the research methodology, which combines ultrasonic field application, theoretical modeling, and experimental validation.

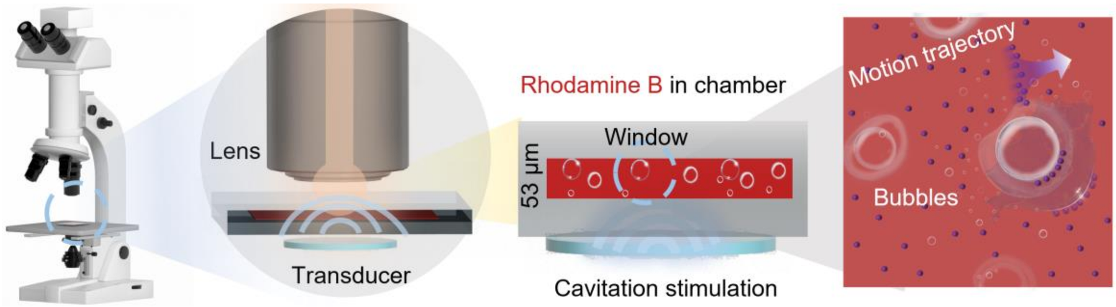

Supplementary Fig. 2|Schematic of the custom-built setup for operando observation of cavitation bubble movement.

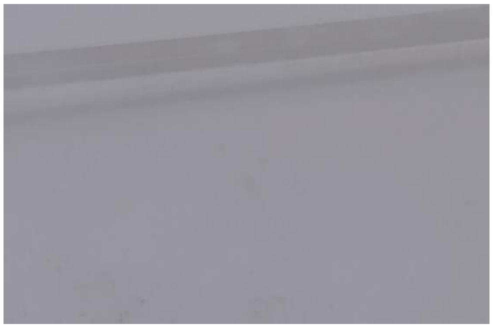

Supplementary Fig. 3|A digital photograph of the custom-built acrylic chamber for operando observation of ultrasonic cavitation.

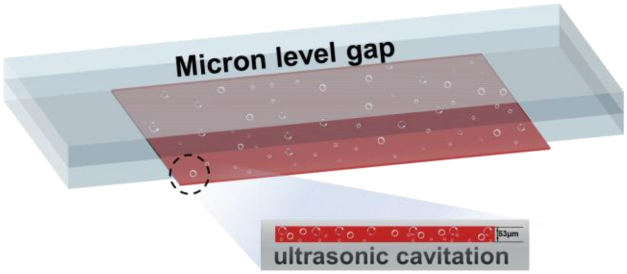

Supplementary Fig. 4|Schematic diagram of the experimental setup for operando observation of ultrasonic cavitation within a miniature acrylic chamber.

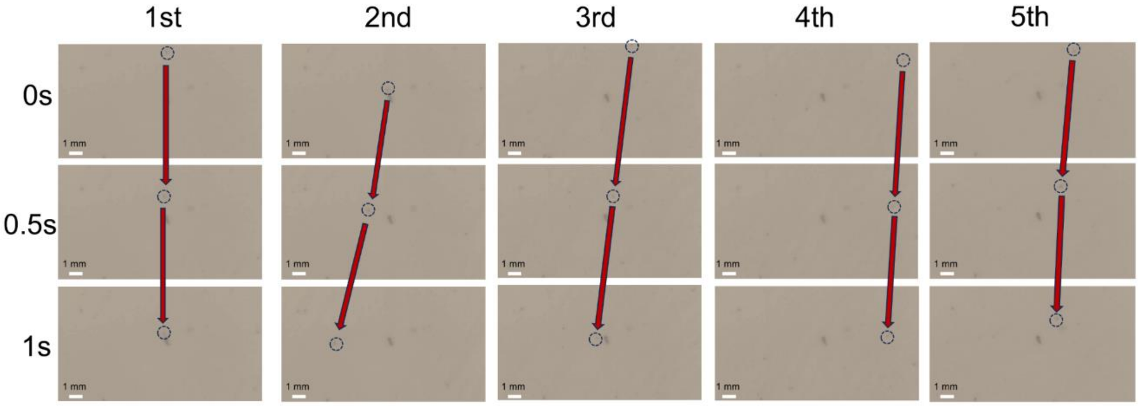

Supplementary Fig. 5|Representative time-resolved microscopy images showing the displacement of multiple bubbles in the ultrasonic field.

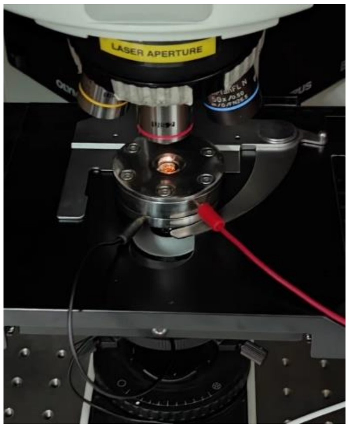

Supplementary Fig. 6|The digital photograph of the operando Raman spectrometer.

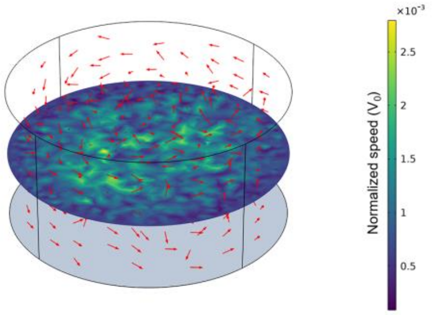

Supplementary Fig. 7|The rotational mass transfer model for electrolyte in Li-S battery in the FEM analysis.

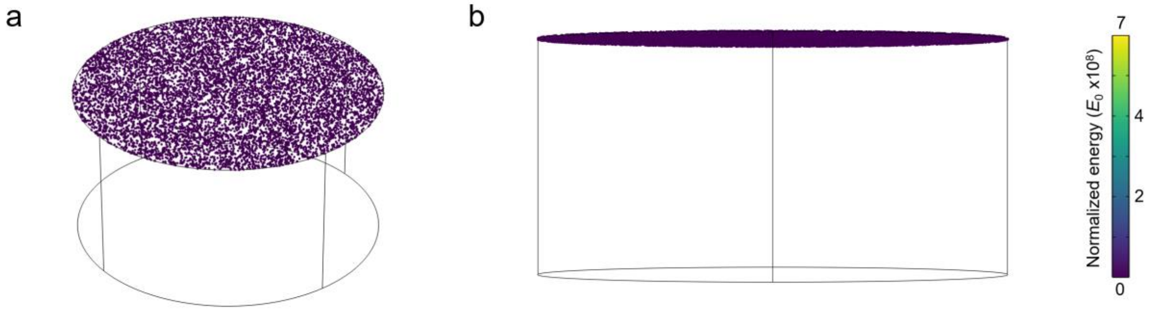

Supplementary Fig. 8|The initial state of the ion rotation trajectory for the electrolyte in the finite element analysis: (a) tilted view and (b) front view.

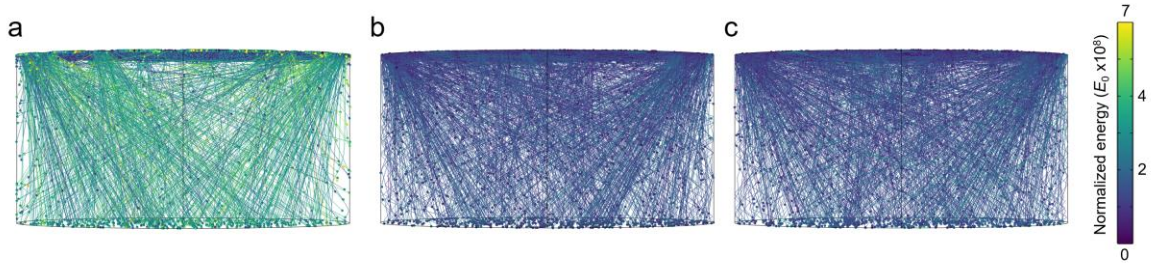

Supplementary Fig. 9|The mid-state front view of the ion rotation trajectory for the electrolyte in the finite element analysis under ultrasonic environments of (a) 0 W, (b) 90 W and (c) 180 W.

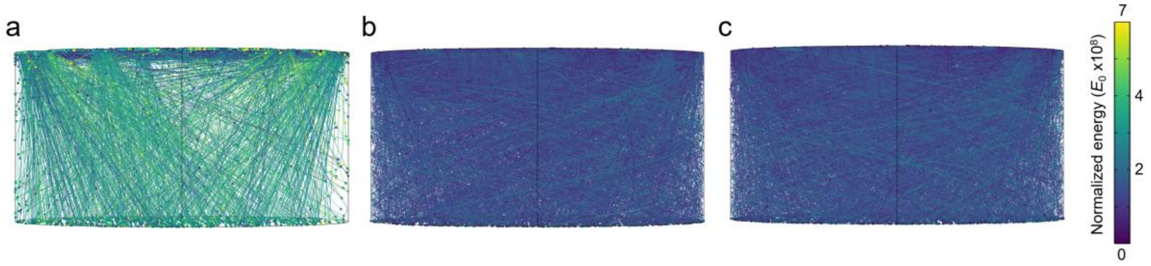

Supplementary Fig. 10|The final-state state front view of the ion rotation trajectory for the electrolyte in the finite element analysis under ultrasonic environments of (a) 0 W, (b) 90 W and (c) 180 W.

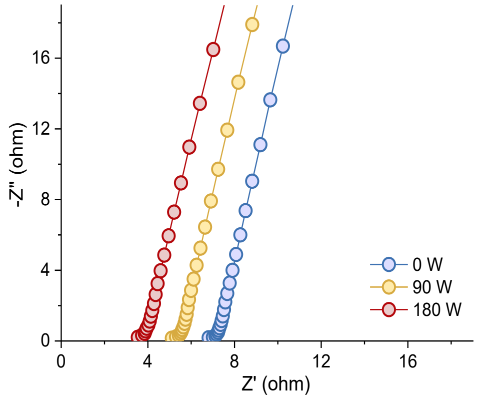

Supplementary Fig. 11|Electrolyte ionic conductivity under ultrasonication at different power levels evaluated by EIS in symmetric cells.

The ionic conductivity σ was calculated using the equation $\sigma=L/R_bA$, where L is the distance between electrodes, A is the area, and R_b is the bulk resistance derived from the high-frequency intercept of the Nyquist plot. The conductivity increased from 0.187 mS cm^{-1} in the pristine state (0 W) to 0.250 mS cm^{-1} at 90 W, and further to 0.361 mS cm^{-1} under 180 W excitation.

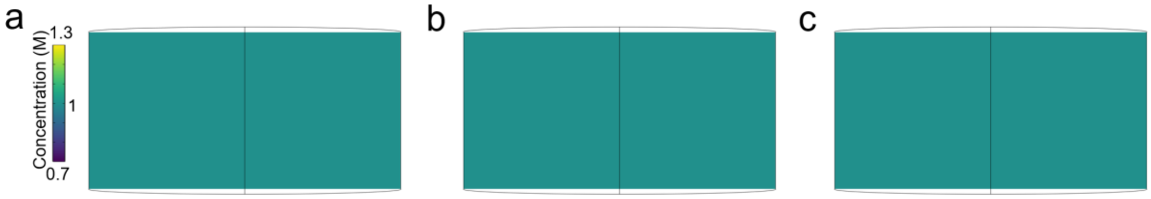

Supplementary Fig. 12|The initial state front view of the concentration field for the electrolyte in the finite element analysis at (a) 0 W, (b) 90 W and (c) 180 W.

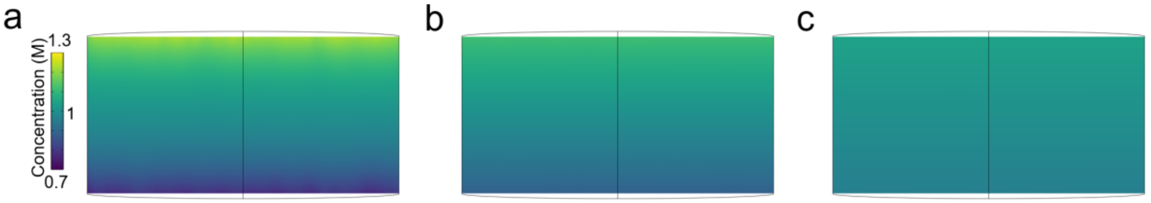

Supplementary Fig. 13|The mid-state front view of the concentration field for the electrolyte in the finite element analysis at (a) 0 W, (b) 90 W and (c) 180 W.

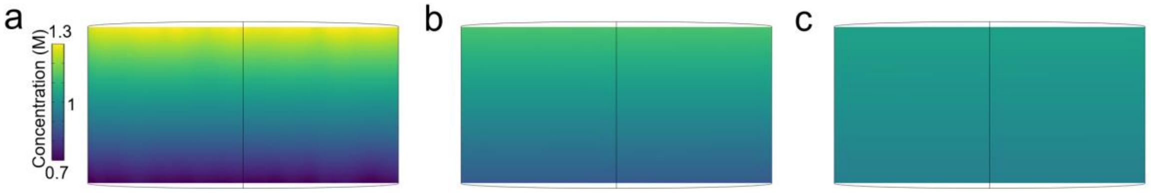

Supplementary Fig. 14|The final-state front view of the concentration field for the electrolyte in the finite element analysis at (a) 0 W, (b) 90 W and (c) 180 W.

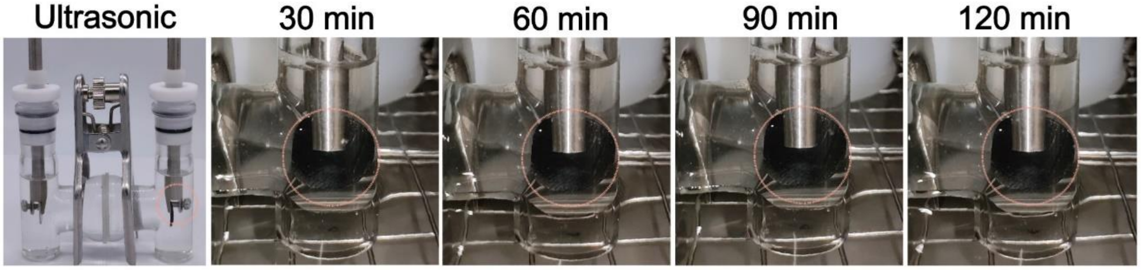

Supplementary Fig. 15|Digital photographs showing the application of an ultrasonic field to the positive electrode of Li–S battery.

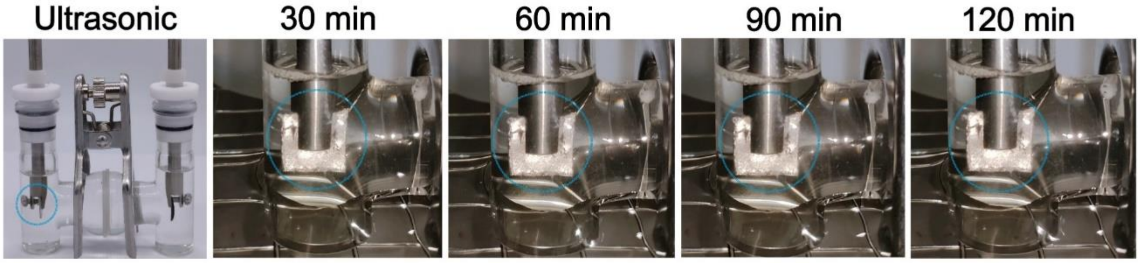

Supplementary Fig. 16|Digital photographs showing the application of an ultrasonic field to the negative electrode of Li–S battery.

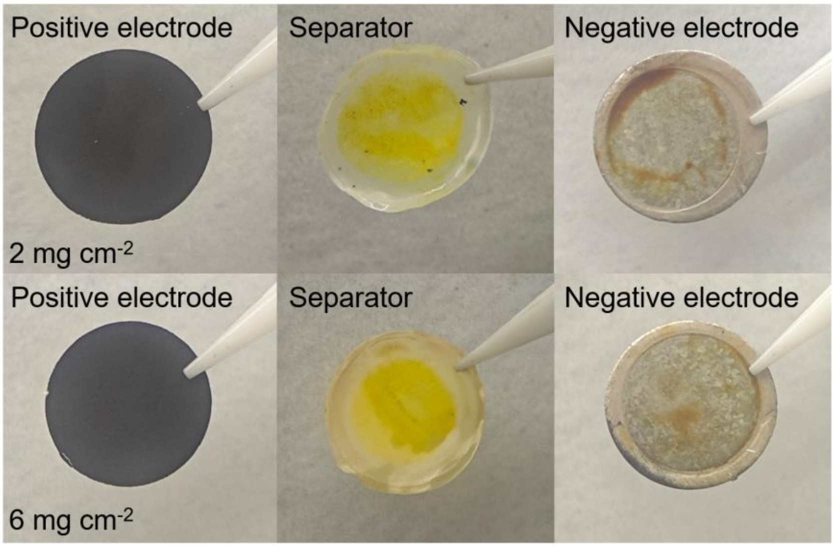

Supplementary Fig. 17|Photographs of cell components after 100 cycles (0.5 C, 180 W ultrasonication) at different sulfur loadings (2 vs. 6 mg cm^{-2}).

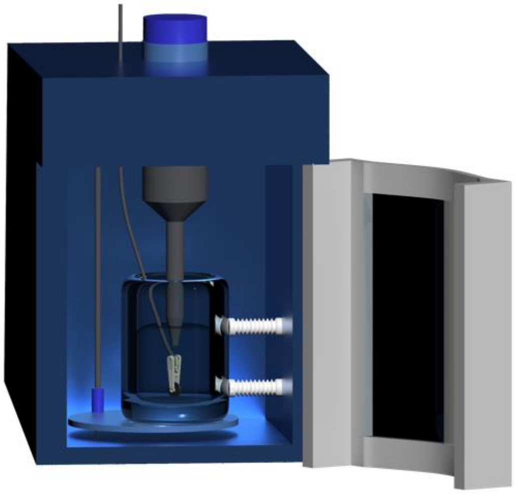

Supplementary Fig. 18|A schematic diagram illustrating the application of an ultrasonic field to a Li-S battery using a transducer.

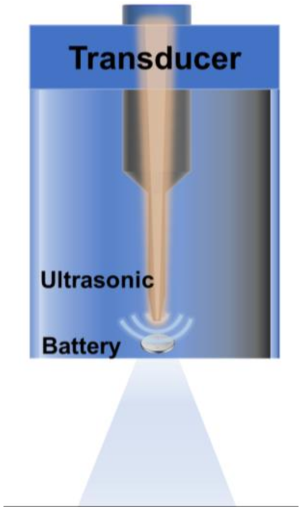

Supplementary Fig. 19|Schematic illustrating the effects of a transducer-generated ultrasonic field on a Li–S battery.

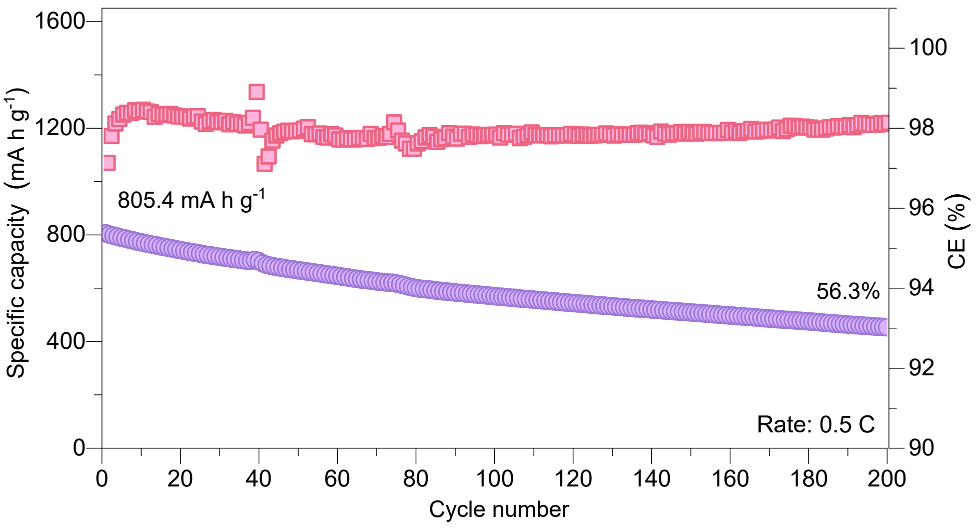

Supplementary Fig. 20|Cycling performance over 200 cycles at 0.5 C of a cell pre-sonicated at 180 W for 1 h and subsequently tested without ultrasonication.

As shown in Supplementary Fig. 20, the performance of the pre-sonicated cell is very similar to that obtained at 0 W (i.e., without sonication), confirming that the impact of the acoustic field is dynamic rather than permanently encoded in the material.

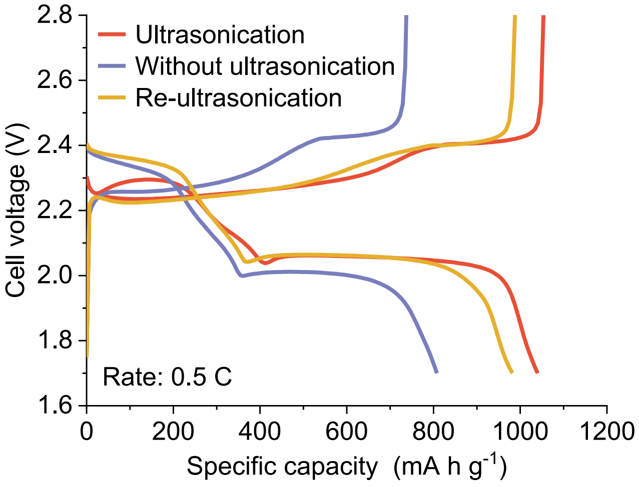

Supplementary Fig. 21|Charge-discharge profiles at 0.5 C of a cell subjected sequentially to ultrasonication, without ultrasonication, and re-ultrasonication.

The beneficial effects on capacity and polarization are only sustained during active sonication; removal of the field leads to an immediate performance decline, which can be promptly restored upon re-application (Supplementary Fig. 21). This 'on/off' dependence

confirms that the enhancement is a transient reversible phenomenon governed by active cavitation.

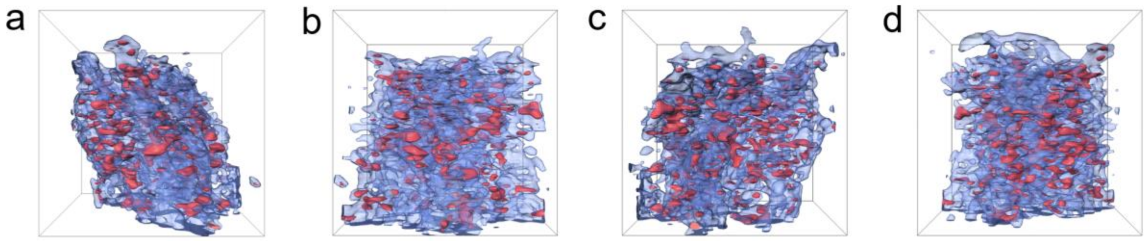

Supplementary Fig. 22|SR 3D nano-CT images of Li_2S precipitates (red) within a MWCNT matrix (blue), shown at various rotation angles in the entropy-driven electrolyte. The rotation angles are (a) 0°, (b) 90°, (c) 180°, and (d) 270°, respectively.

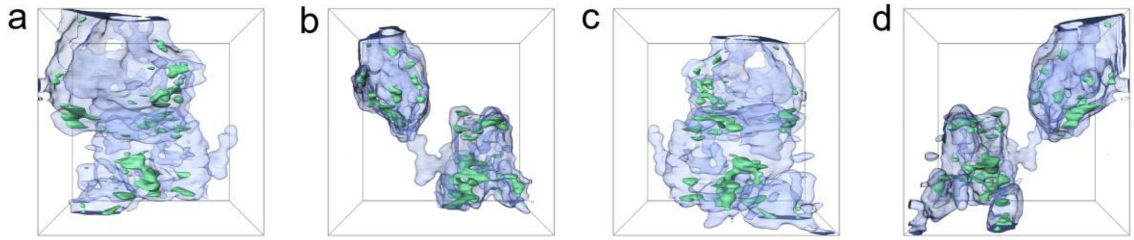

Supplementary Fig. 23|SR 3D nano-CT images of Li_2S precipitates (green) within a MWCNT matrix (blue), shown at various rotation angles in the regular electrolyte. The rotation angles are (a) 0°, (b) 90°, (c) 180°, and (d) 270°, respectively.

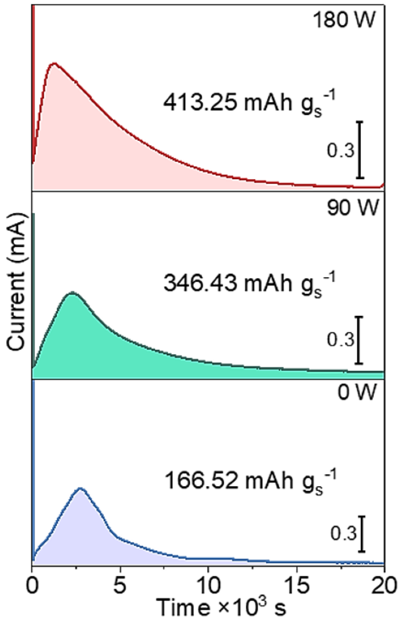

Supplementary Fig. 24|Potentiostatic decomposition curves of Li_2S using electrolytes influenced by ultrasonic field of different strengths. The shaded areas represent the integrated discharge capacities.

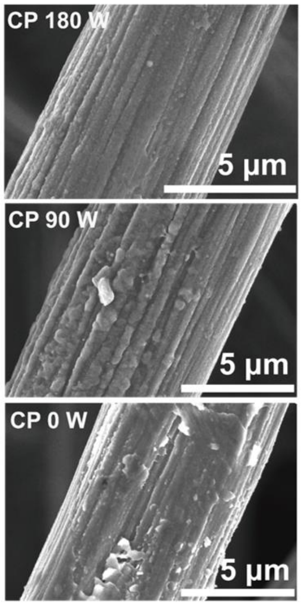

Supplementary Fig. 25|SEM images of carbon paper after potentiostatic charging in electrolytes under ultrasonic fields of varying strengths.

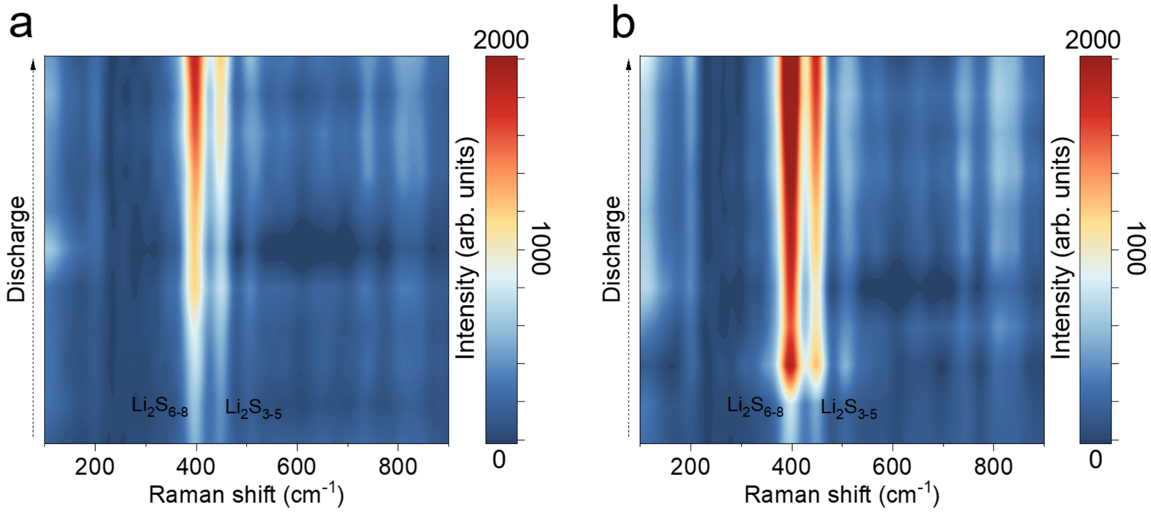

Supplementary Fig. 26|Time-resolved Raman spectra recorded at the negative electrode/separator interface during discharge (a) with and (b) without 180 W ultrasonication.

In the absence of ultrasound, Raman mapping reveals a rapid increase in signal intensity for sulfur species at the negative electrode side, confirming significant shuttling. In stark contrast, under 180 W ultrasonication, the appearance of lithium polysulfide signals is markedly delayed and the overall intensity remains substantially lower throughout the process (Supplementary Fig. 26). This suppression of the shuttle effect, despite enhanced convection, is attributed to the accelerated sulfur conversion kinetics at the positive electrode. By facilitating the rapid transformation of soluble LiPSs into solid Li_2S, the source of shuttle species is depleted, thereby reducing the total amount of lithium polysulfide available for migration.

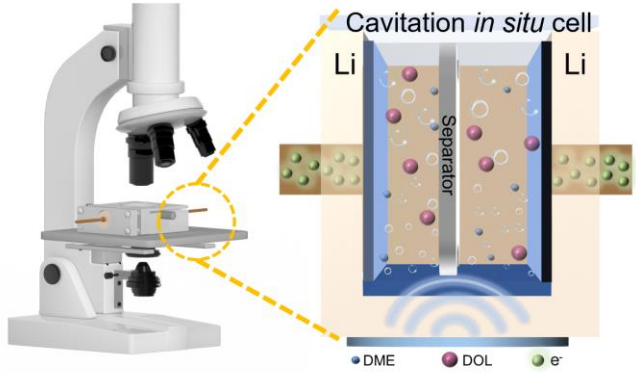

Supplementary Fig. 27|In situ optical microscopy to monitor lithium surface. The left panel presents the setup for the time-resolved in situ optical microscopy observations. The right panel provides a detailed schematic illustration of the in situ observation cell under the influence of ultrasonication.

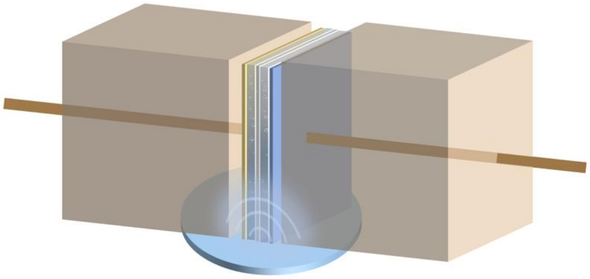

Supplementary Fig. 28|Schematic oblique view of the in situ observation setup. The electrode modules for in situ optical microscopy are shown on the left and right sides of the schematic. The central region illustrates the observation cell, consisting of a symmetric Li||Li configuration. The bottom part indicates the source of the applied ultrasonic field.

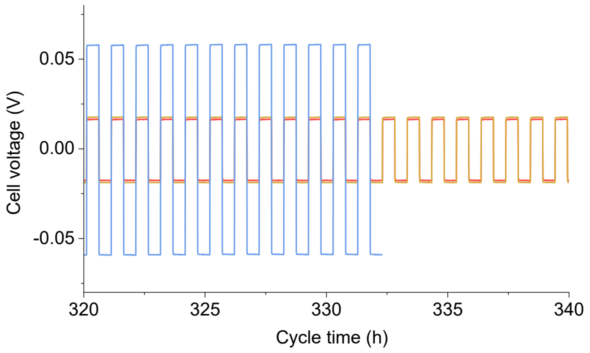

Supplementary Fig. 29|Voltage-time curves of Li||Li symmetric cells using electrolytes under different ultrasonic fields. The batteries were cycled at a current density of 2.0 mA cm^{-2} with a plating/stripping capacity of 2.0 mA h cm^{-2}.

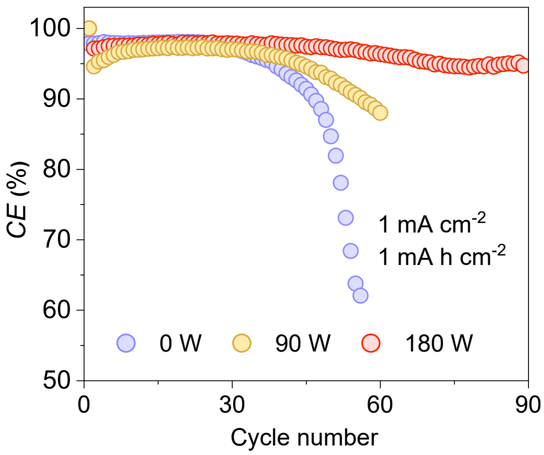

Supplementary Fig. 30|Coulombic efficiency (CE) of Li||Cu cells at 1 mA cm^{-2}.

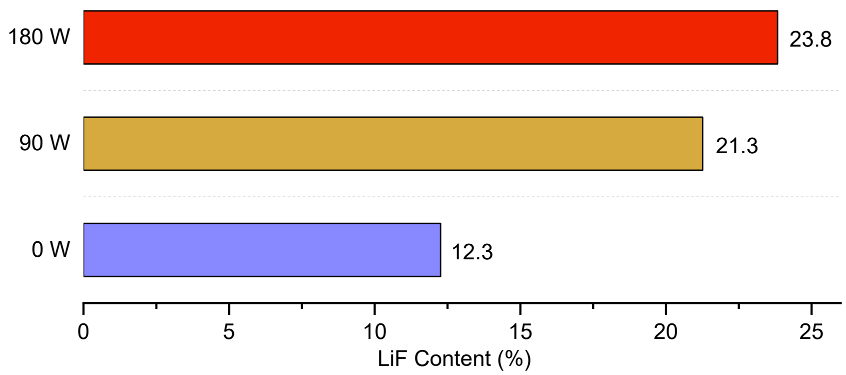

Supplementary Fig. 31|Comparison of the LiF content in the SEI regarding cycled lithium metal electrodes.

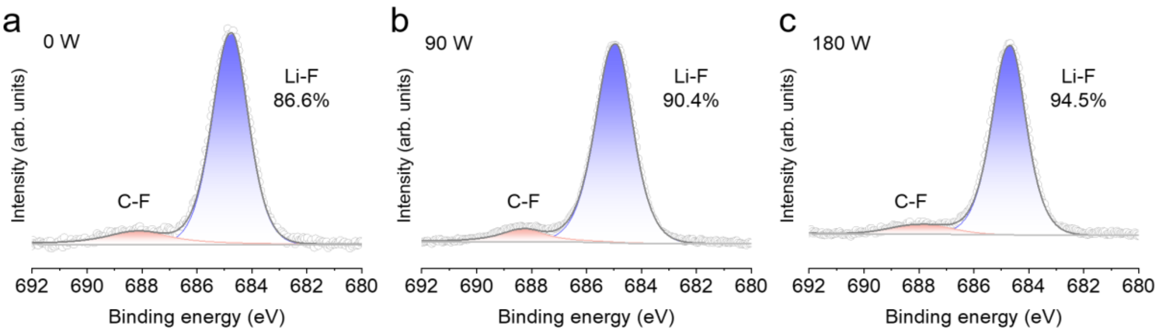

Supplementary Fig. 32|Depth-profiling X-ray photoelectron spectroscopy analysis of F *1s* spectra for SEI formed on Li-metal electrodes under varying ultrasonication power of (a) 0 W, (b) 90 W, and (c) 180 W. Samples were harvested from Li||Li symmetric cells after 100 cycles at a current density of 2 mA cm^{-2} and an areal capacity of 2 mA h cm^{-2}.

To further corroborate the chemical signature of LiF, fine F *1s* core-level spectra were analyzed (Supplementary Fig. 32). A dominant peak at approximately 684.8 eV, characteristic of the Li-F bond, is observed across all samples. Quantitative analysis reveals that the relative atomic proportion of LiF increases from 86.6% in the control group (0 W) to 90.4% at 90 W, and reaches a maximum of 94.5% under the 180 W ultrasonic condition. This trend directly supports the Li *1s* data, confirming that the ultrasonic field facilitates a LiF-rich SEI layer.

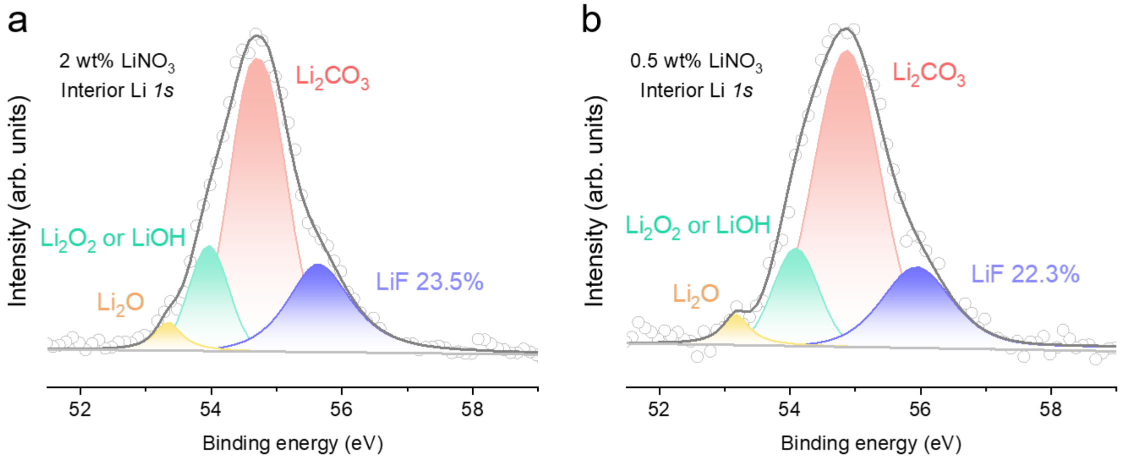

Supplementary Fig. 33|Depth-profiling X-ray photoelectron spectroscopy analysis of Li *1s* spectra was used to evaluate the effect of (a) 2 wt% and (b) 0.5 wt% $LiNO_3$ concentration on LiF content within the inner Solid Electrolyte Interphase (SEI). The samples were obtained from ultrasonication mediated Li||Li symmetric cells cycled 100 times at 2 mA cm^{-2} and 2 mA h cm^{-2}.

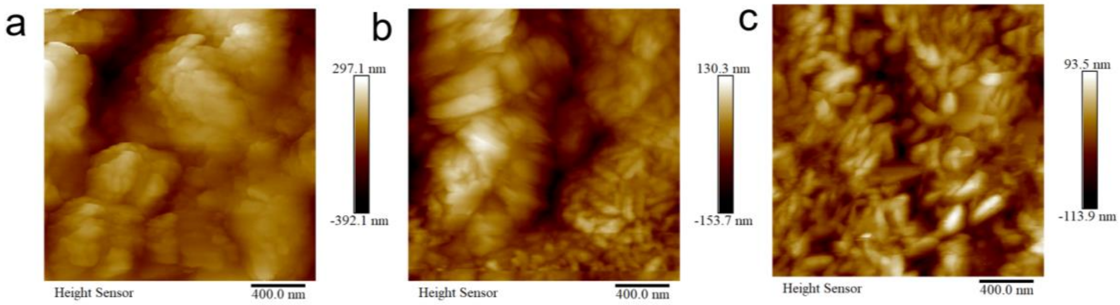

Supplementary Fig. 34|AFM images of lithium foils after cycling in electrolytes mediated by ultrasonication of (a) 0 W, (b) 90 W, and (c) 180 W. Samples were harvested from Li||Li symmetric cells after 100 cycles at a current density of 2 mA cm^{-2} and an areal capacity of 2 mA h cm^{-2}.

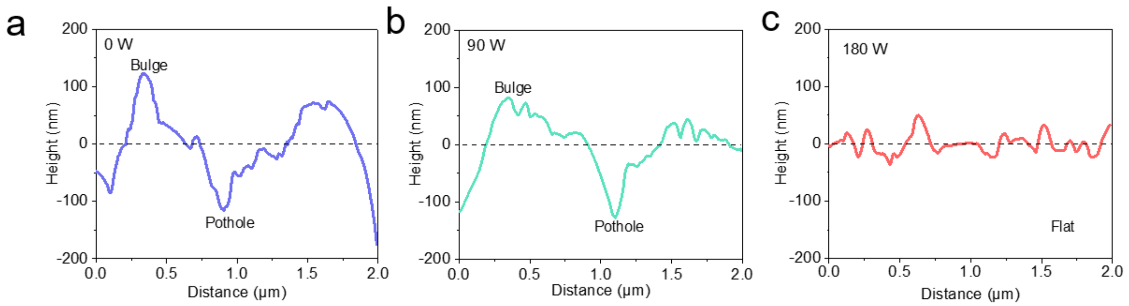

Supplementary Fig. 35|AFM surface line profiles of cycled Li electrodes by ultrasonication of (a) 0 W, (b) 90 W, and (c) 180 W. Samples were harvested from Li||Li symmetric cells after 100 plating/stripping cycles at a current density of 2 mA cm^{-2} and an areal capacity of 2 mA h cm^{-2}.

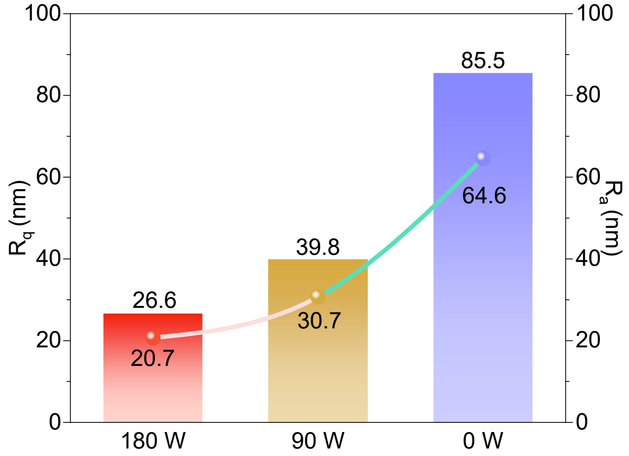

Supplementary Fig. 36|Comparison of surface roughness values for cycled lithium electrodes, derived from AFM data. The values shown are the arithmetic average roughness (R_a) and the root-mean-square roughness (R_q).

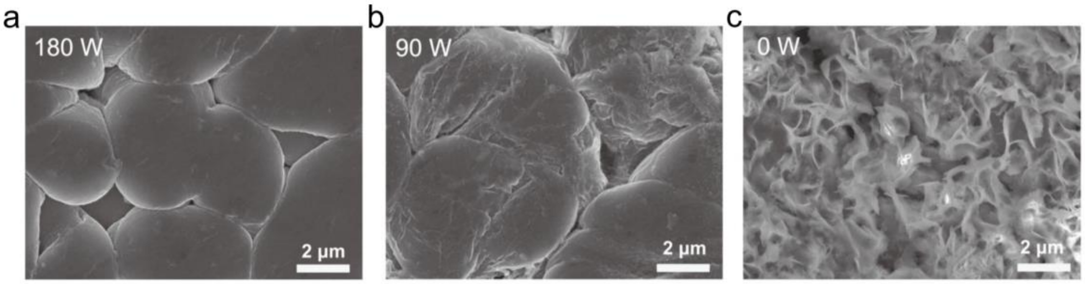

Supplementary Fig. 37|SEM images of lithium electrodes after 100 cycles at 2.0 mA cm^{-2} and 2.0 mAh cm^{-2} in electrolytes subjected to ultrasonic fields of (a) 180 W, (b) 90 W, and (c) 0 W. Cells were disassembled at the symmetric cell's near-zero potential state for post-mortem analysis.

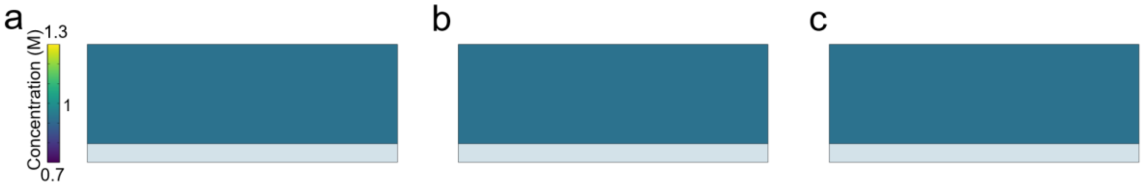

Supplementary Fig. 38|The initial state front view of the Li-ion concentration profile and the state of lithium deposition in electrolytes subjected to ultrasonication at (a) 0 W, (b) 90 W and (c) 180 W.

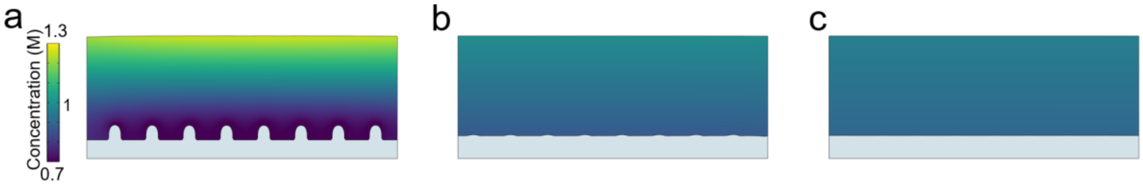

Supplementary Fig. 39|The mid-state front view of the Li-ion concentration profile and the state of lithium deposition in electrolytes subjected to ultrasonication at (a) 0 W, (b) 90 W and (c) 180 W.

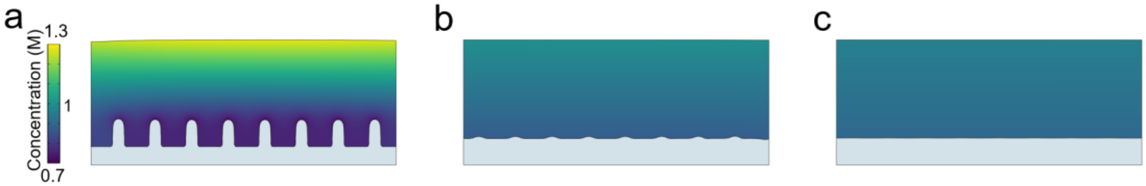

Supplementary Fig. 40|The final-state front view of the Li-ion concentration profile and the state of lithium deposition in electrolytes subjected to ultrasonication at (a) 0 W, (b) 90 W and (c) 180 W.

By supplying Li-ions to the electrode interface continuously and at high flux, the acoustic field mitigates localized ion depletion.[1] In parallel, ultrasound induces modifications to the Li-ion solvation structure, facilitating desolvation and lowering the energy barrier for lithium metal deposition. As a result, the system becomes markedly less sensitive to localized electric-field "hot spots".[2] Moreover, ultrasonication promotes the formation of a chemically stable and mechanically robust, LiF-rich SEI. This interphase helps homogenize Li-ion entry points across the electrode surface, effectively shielding the lithium metal from electric-field non-uniformities and enabling smoother deposition. From a theoretical perspective based on electrochemical nucleation-growth models,[3] ultrasound-enhanced Li-ion transport increases the local chemical potential near the electrode, thereby reducing the critical nucleation barrier. This effect favors dense, multi-site nucleation even under identical electric field distributions, ultimately driving lithium deposition toward a dendrite-free morphology (Supplementary Fig. 40).

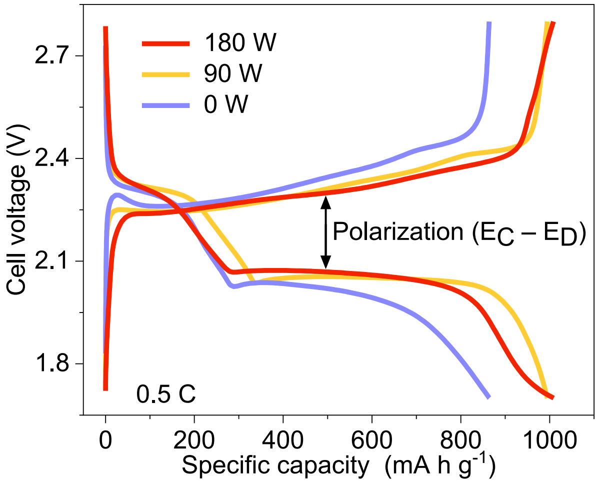

Supplementary Fig. 41|Comparison of GCD curves at the rate of 0.5 C for batteries with different electrolytes.

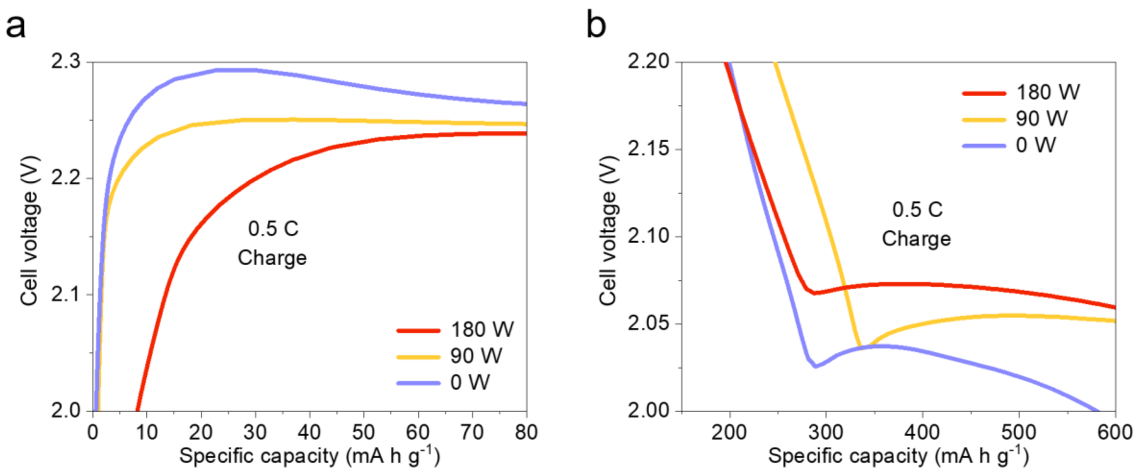

Supplementary Fig. 42|Comparison of (a) charge and (b) discharge curves of cells using electrolytes under different ultrasonic field strengths at 0.5 C.

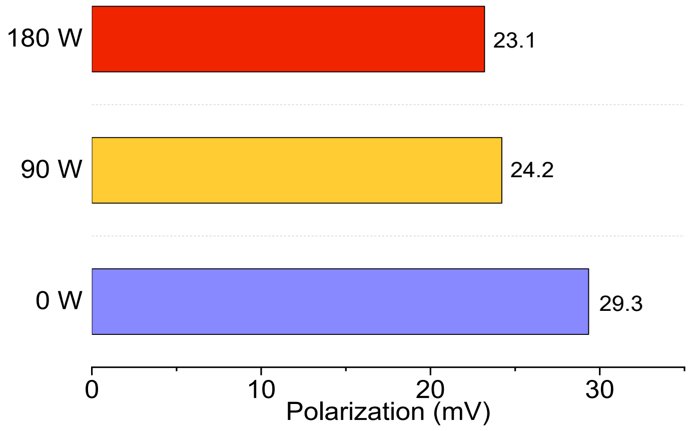

Supplementary Fig. 43|Comparison in cell polarization of batteries using electrolytes under different ultrasonic field strengths at 0.5 C.

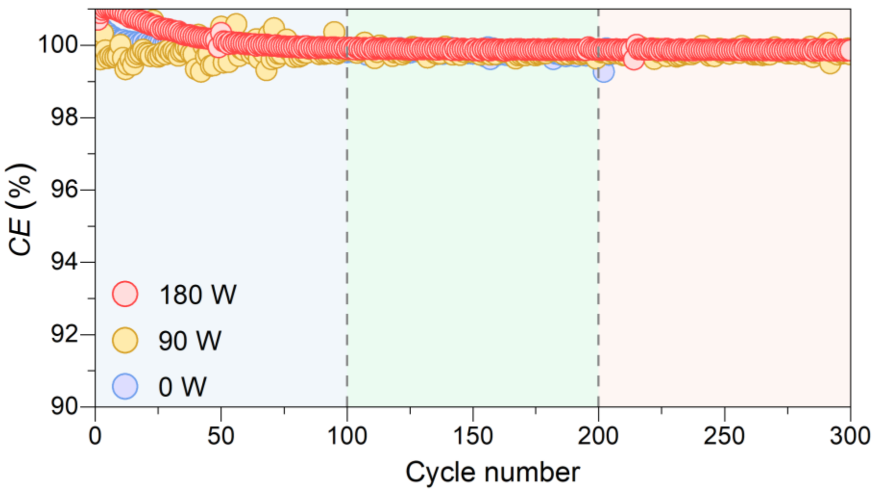

Supplementary Fig. 44|A zoomed-in view of the CE variation for cells subjected to different sonication conditions at 0.5 C over 300 cycles.

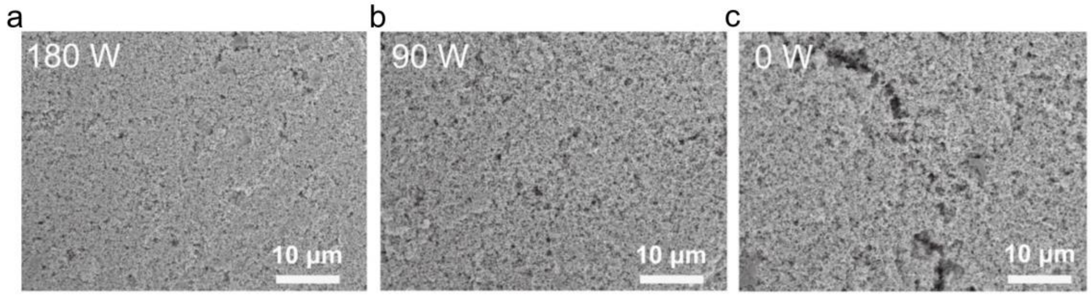

Supplementary Fig. 45|Front-view SEM images of sulfur positive electrodes cycled for 100 times at 0.5 C within a cell voltage window of 1.7–2.8 V, using ultrasonic-field-enabled electrolytes. Ultrasonic power at (a) 180 W, (b) 90 W, and (c) 0 W. Cells were disassembled in the fully charged state (2.8 V) for post-mortem analysis.

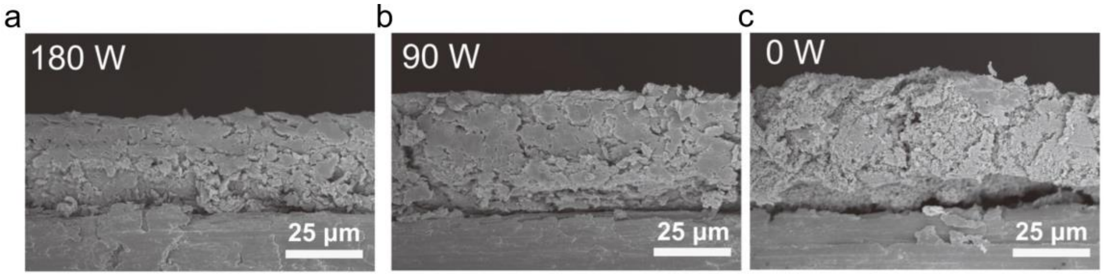

Supplementary Fig. 46|Cross-sectional SEM images of sulfur positive electrodes cycled for 100 times at 0.5 C within a cell voltage window of 1.7–2.8 V using ultrasonic-field-enabled electrolytes. Ultrasonic power at (a) 180 W, (b) 90 W, and (c) 0 W. Cells were disassembled in the fully charged state (2.8 V) for post-mortem analysis.

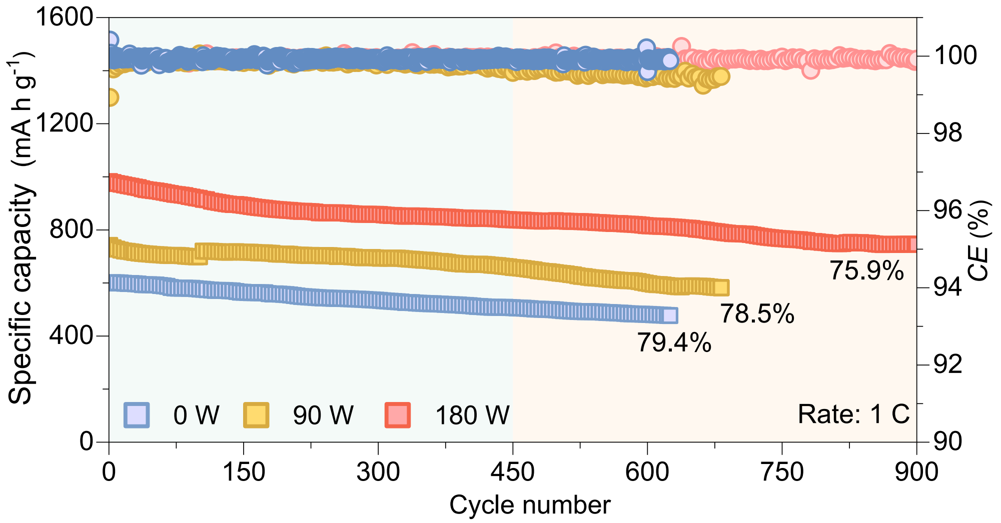

Supplementary Fig. 47|The long-term cycling stability of Li–S cells at 1 C in electrolytes mediated by ultrasonic fields of different strengths.

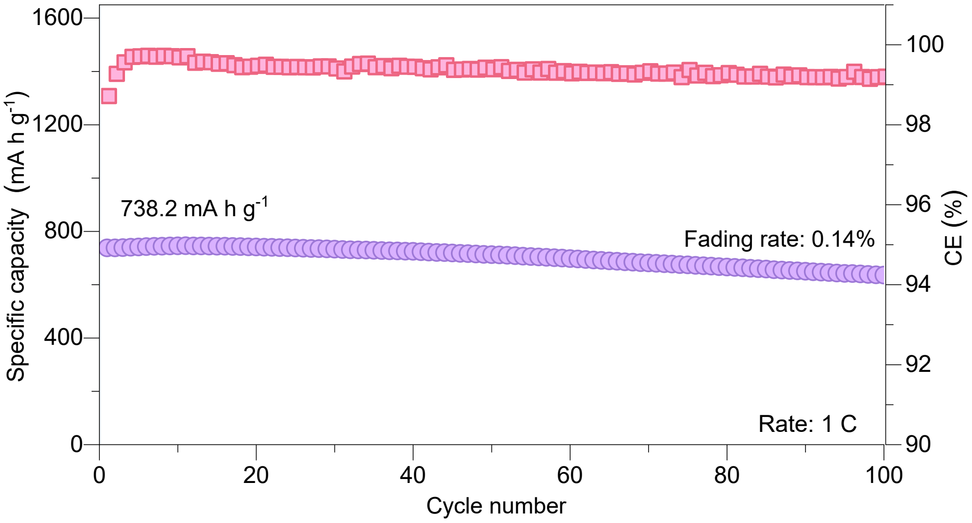

Supplementary Fig. 48|The cycling performance at 1 C under 210 W.

At 210 W, the electrochemical performance noticeably degraded, with the discharge specific capacity at 1.0 C dropping to 738.2 mA h g^{-1} and the capacity decay rate increasing significantly to 0.14% per cycle over 100 cycles (Supplementary Fig. 48). This contrast is stark compared to the 180 W condition, which maintained superior capacity and a low fading rate of 0.027% per cycle over 900 cycles.

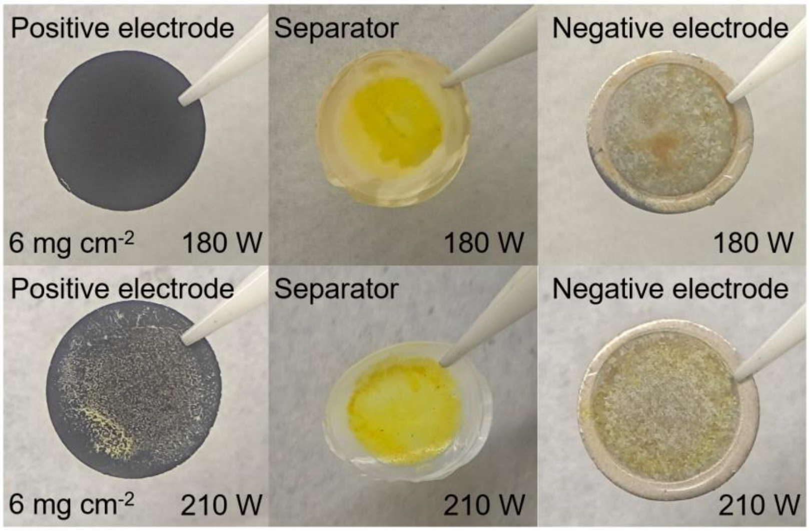

Supplementary Fig. 49|Digital photographs of disassembled cells (6 mg cm^{-2}) after 100 cycles at 0.5 C under ultrasonication at 180 W (top) and 210 W (bottom).

This performance degradation at 210 W is likely attributed to mechanical stress exceeding the binding strength of the electrode components. This is evidenced in the comparison of digital photographs of electrode components dissembled from cycled cells (Supplementary Fig. 49). Specifically, the electrode cycled at 210 W exhibits pronounced surface delamination and pulverization, accompanied by the widespread appearance of yellow patches on the black electrode surface. While at 180 W, the mechanical stress remains within the tolerance of the electrode structure.

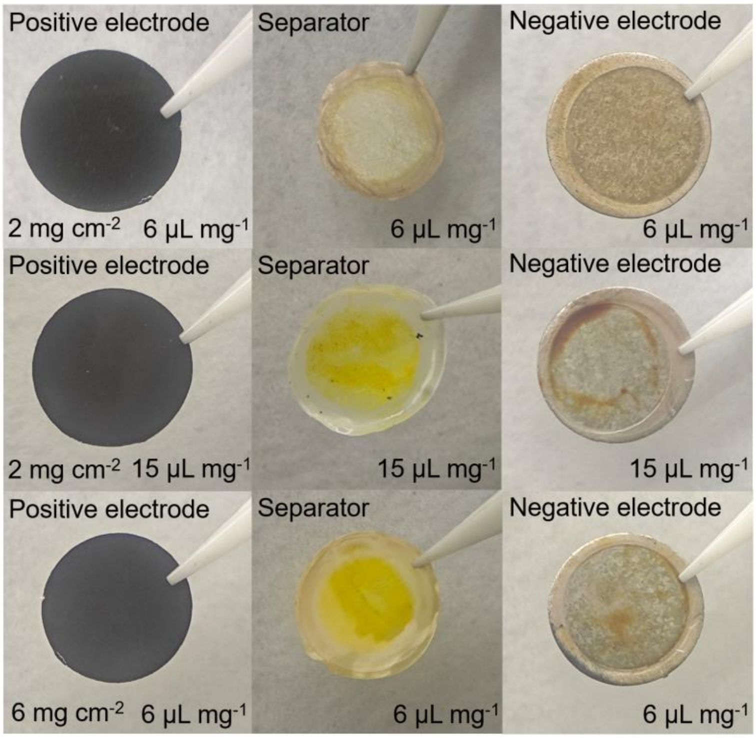

Supplementary Fig. 50| Examination of cell components harvested from disassembled cells after 100 cycles at 0.5 C, 180 W ultrasonication under different conditions: 2 mg cm^{-2}, E/S = 6 μL mg^{-1}); 2 mg cm^{-2}, E/S = 15 μL mg^{-1}; and 6 mg cm^{-2}, E/S = 6 μL mg^{-1}.

The post-mortem analysis confirms that the devices maintain excellent structural integrity across all tested parameter combinations. Even under the most rigorous condition, high mass loading (6 mg cm^{-2}) combined with a lean electrolyte (E/S = 6 μL mg^{-1}), no evidence of active-material delamination or detachment from the current collector was observed. Furthermore, the separators exhibited no mechanical damage, and the lithium negative electrodes showed no signs of pulverization, demonstrating that the 180 W ultrasonic field is mechanically compatible with a wide range of cell configurations (Supplementary Fig. 50).

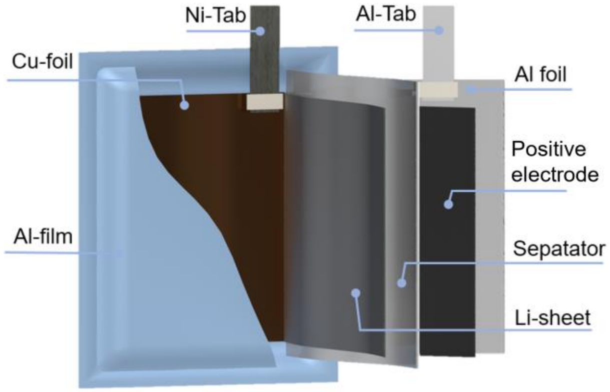

Supplementary Fig. 51|Schematic of a soft-packaged pouch cell.

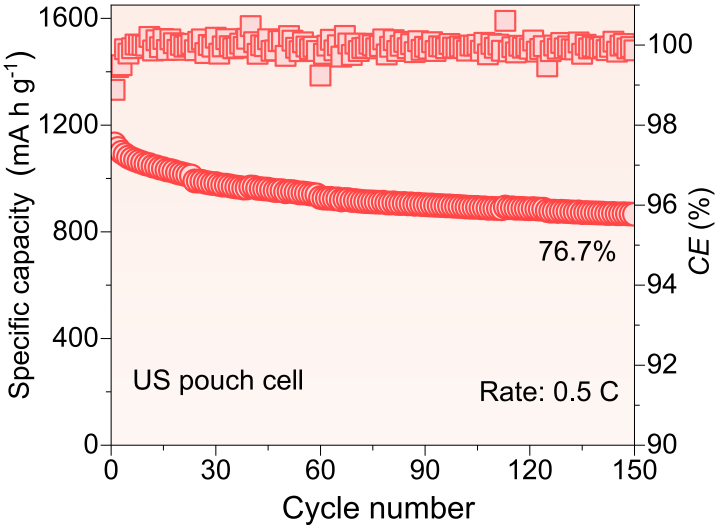

Supplementary Fig. 52|Cycling performance of the high-loading single-layer pouch cell (5.2 mg cm^{-2}) optimized by the entropy-driven electrolyte at 0.5 C.

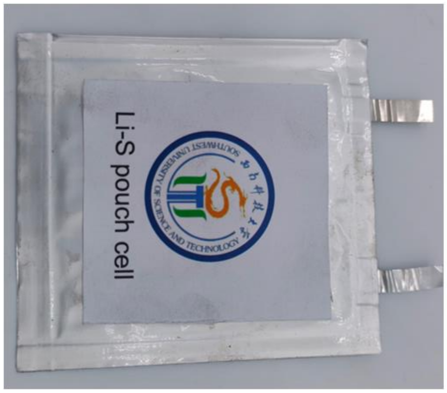

Supplementary Fig. 53|A photograph of the assembled single-layer soft-package pouch cell.

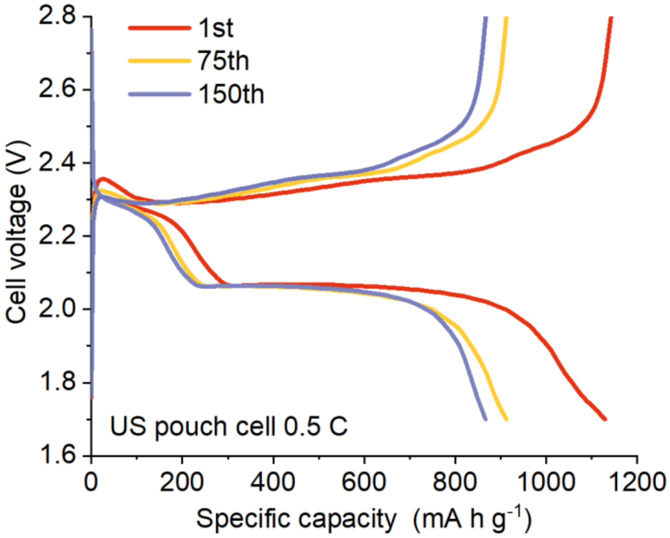

Supplementary Fig. 54|Charge-discharge profiles of the high-loading single-layer pouch cell (5.2 mg cm^{-2}) at the initial, mid-cycle, and end-of-life stages.

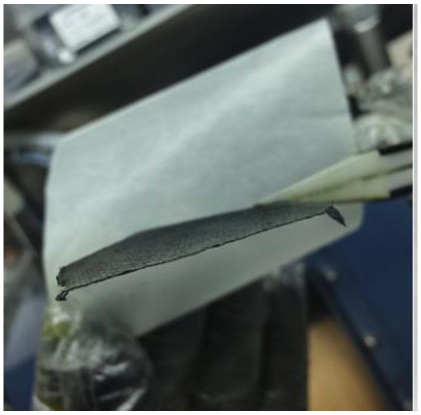

Supplementary Fig. 55|Tilted-angle digital photographs examining the surface condition and adhesion to the current collector of the positive electrode harvested from a high-loading pouch cell (5.2 mg cm^{-2}) after cycling under ultrasonication at 180 W.

The robust electrochemical performance of the high-loading pouch cell provides strong evidence that electrode integrity was maintained. Visual confirmation of the structural integrity of the high-loading pouch cell positive electrode after 150 cycles under ultrasonication at 180 W was also conducted (Supplementary Fig. 55). The digital photograph of the harvested positive electrode reveals a uniform surface with no signs of detachment, confirming that the optimized ultrasonic power (180 W) delivers sufficient enhancement without exceeding the mechanical yield strength of the thick, porous positive electrode structure.

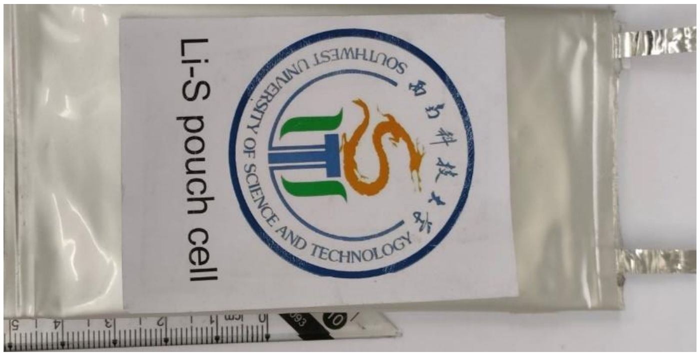

Supplementary Fig. 56|A photograph of the assembled multi-layer stacked pouch cell.

To demonstrate the scalability and practical potential of this strategy, an Ah-level stacked pouch cell was assembled using stringent parameters:[4] a high positive electrode loading (9.4 mg cm^{-2}), a thin lithium reservoir (50 μm), and a lean electrolyte condition (E/S = 2.9 μL mg^{-1}). While the single-layer pouch cell demonstrates the long-term chemical stability of the system over 150 cycles, The primary objective of this Ah-level prototype was to validate the effectiveness of acoustic-driven mediation under extreme constraints required for state-of-the-art specific energy, in comparison with related prominent works (Supplementary Table 2). Achieving 1.12 Ah and 404.1 Wh kg^{-1} under these conditions confirms that the strategy remains robust even as the E/S ratio and lithium excess are minimized to their practical limits.

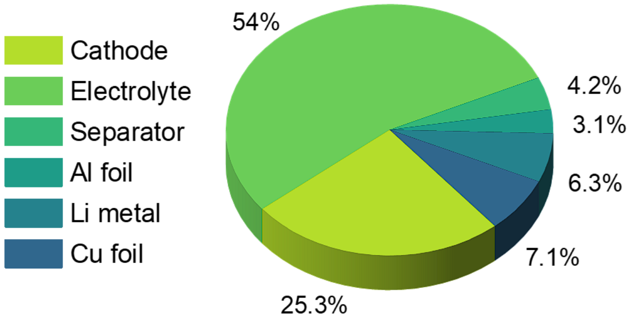

Component	Weight (g)
Positive electrode	1.5
Electrolyte	3.21
Separator	0.25
Al foil	0.18
Li foil	0.37
Cu foil	0.42
Total weight	5.94

Supplementary Fig. 57|Pie chart weight analysis of the Ah-level stacked pouch cell. Note: Specific energy calculations exclude the weight of Al-plastic film.

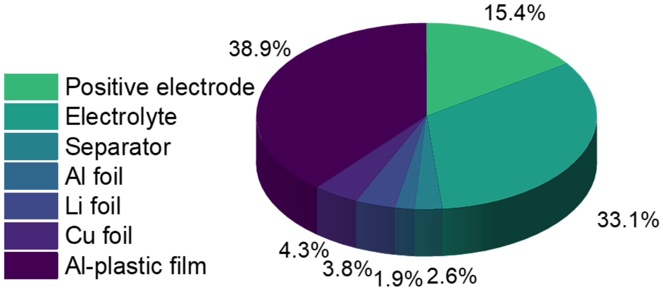

Component	Weight (g)
Positive electrode	1.5
Electrolyte	3.21
Separator	0.25
Al foil	0.18
Li foil	0.37
Cu foil	0.42
Al-plastic film	3.78
Total weight	9.72

Supplementary Fig. 58|Weight breakdown of the Ah-level stacked pouch cell. The pie chart and accompanying table specify the gravimetric contribution of each constituent, used for calculating the specific energy (with the weight of the Al-plastic film considered).

A comprehensive mass breakdown of all components in the stacked cell, including the Al–plastic laminate film, is provided in Supplementary Fig. 58. When the full mass of the cell housing is considered, the resulting specific energy is 247.0 Wh kg^{-1}.

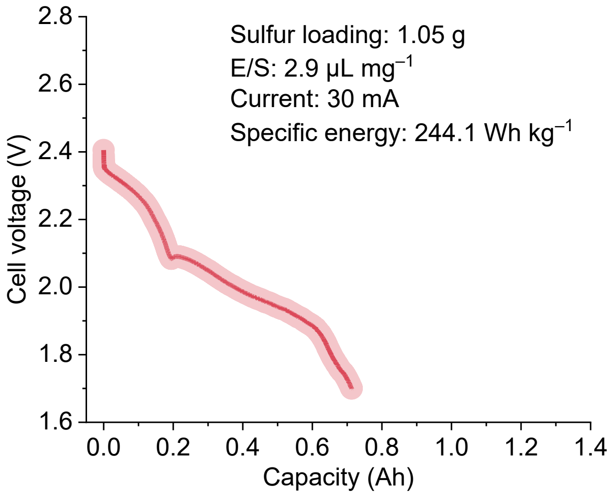

Supplementary Fig. 59|Discharge curve of a high-loading (1.05 g sulfur) multilayer Li–S stacked pouch cell operating without ultrasonic mediation, delivering a specific energy of 244.1 Wh kg^{-1} without packaging.

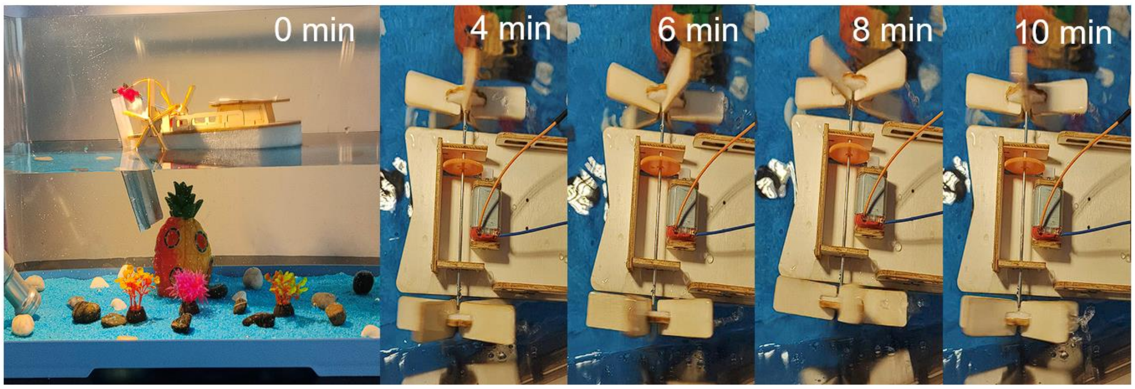

Supplementary Fig. 60|An electronic device powered by a multi-layer pouch cell utilizing the entropy-driven electrolyte

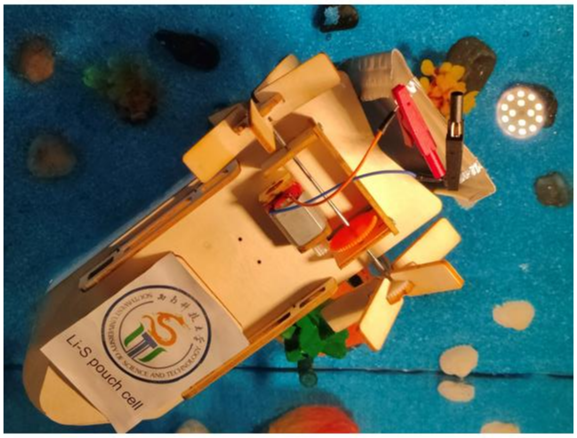

Supplementary Fig. 61|Demonstration of a toy boat powered by a multilayer pouch cell employing an entropy-driven electrolyte under ultrasonic operation.

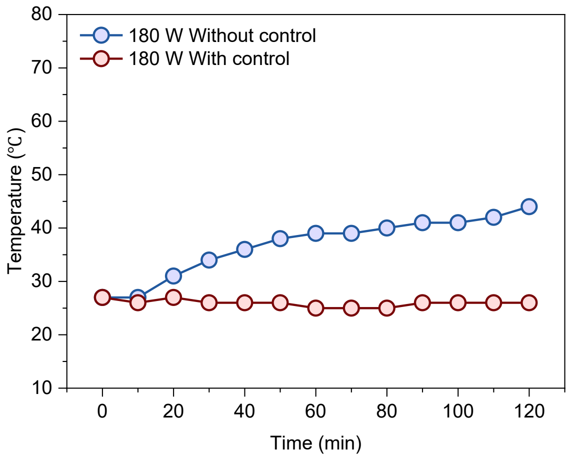

Supplementary Fig. 62|Temperature profiles of cells subjected to ultrasonication under conditions with and without temperature control.

To validate the safety protocols, control experiments were conducted comparing the thermal behavior of a cell operating with active closed-loop temperature control against an unregulated system (Supplementary Fig. 62). In the absence of active regulation, the cell temperature climbed steadily over time. In contrast, with the closed-loop system engaged, the cell temperature is clamped at 28 °C throughout operation. Furthermore, thermal stability is chemically complemented by the formation of a robust interface. Ultrasonic treatment promotes the formation of a LiF-rich passivation layer, which chemically stabilizes the electrode surface and suppresses continuous parasitic electrolyte decomposition.

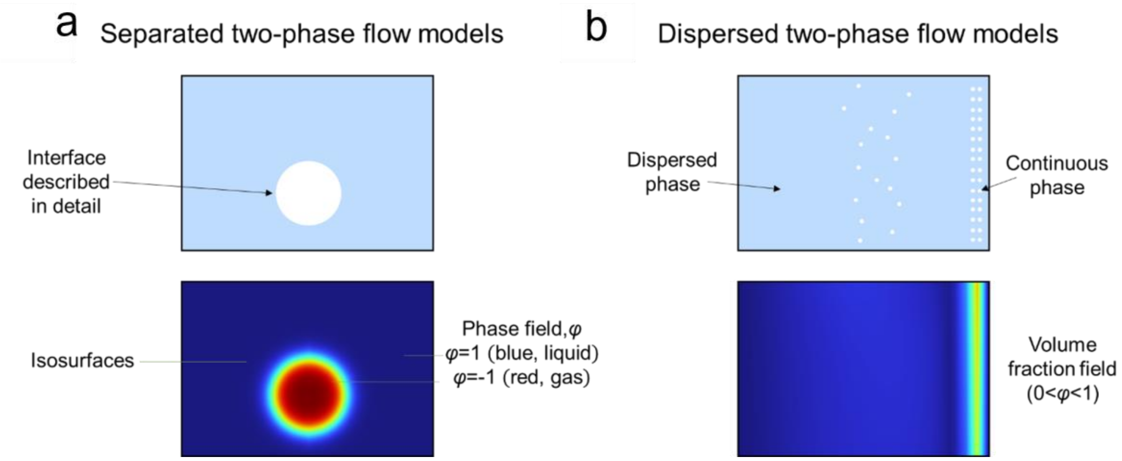

Supplementary Fig. 63|Schematic diagram for (a) separated two-phase flow models and (b) dispersed two-phase flow models.

The choice between separated and dispersed two-phase flow models is governed by the relative length scales of the bubbles and the computational domain. A separated two-phase flow model is employed when the bubble size is comparable to the model scale, enabling explicit tracking of individual bubble interfaces (Supplementary Fig. 63a). In contrast, a dispersed two-phase flow model is adopted here due to the extreme scale disparity between the cavitation bubbles and the simulation domain, wherein the gas and liquid phases are represented by field variables ($0 < \varphi < 1$) (Supplementary Fig. 63b). This framework accounts for interfacial effects, such as surface tension, buoyancy, and mass transfer. These source terms mathematically describe the energy dispersal from the cavitation field, which drives the formation of toroidal vortices and governs the macroscopic rotational trajectories of reactive species.

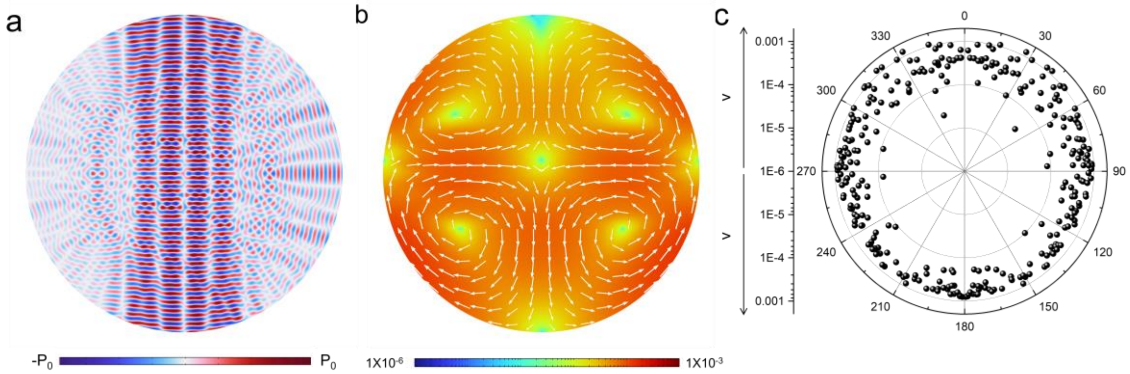

Supplementary Fig. 64|(a) The 2D in-plane distribution of acoustic pressure in the calculated domain. (b) The corresponding spatial distribution of the normalized fluid velocity, as represented by white arrows. (c) Polar coordinate statistics of the normalized velocity, in which the radial direction corresponds to the velocity magnitude and the angular coordinate represents the deflection angle, θ.

Even though individual bubble structures are rapidly morphing, time-averaged result reveals macroscopic features that converge toward a steady-state velocity distribution, providing a stable basis for fluid dynamic modeling.[5–7] Furthermore, the bounded geometry of the battery cell plays a role in regularizing these motions. In such a confined environment, incident ultrasonic waves reflect off the boundaries, interfering with incoming waves to form acoustic standing waves. This is confirmed from a steady 2D in-plane acoustic pressure distribution within the cell domain from the simulation (Supplementary Fig. 64a). These standing waves further dictate the behavior of the cavitation bubbles through Bjerknes radiation forces, which would effectively guide bubbles trajectories, preventing the chaotic "wandering".[8] This phenomenon further promotes the formation of steady-state velocity distribution characterized by macroscopic regularity. As a result, the microscale randomness is reconciled in this work by coupling the bubble-generated impacts as an averaged term into the Navier-Stokes equations to solve for the velocity distribution. The results indicate that the electrolyte domain reaches a converged steady state dominated by a uniform vortex flow, which aligns with the circulating vortex patterns characteristic of acoustic streaming.[9] This is evidenced in the uniform velocity distribution observed in the simulation results (Supplementary Fig. 64b and 64c).

Supplementary Table 1|Information related to the fitting of the EIS curve. The values in parentheses represent the fitting errors.

Sample	Re (Ω)	Rct (Ω)	CPE-P	CPE-T (Ω^{-1} s^n)	W (Ω $s^{-1/2}$)
0 W	2.91(0.10)	43.9(0.38)	0.75(0.006)	$3.73*10^{-5}(2.13*10^{-6})$	12.7(1.7)
90 W	0.95(0.08)	26.4(0.26)	0.82(0.008)	$2.54*10^{-5}$ $(1.85*10^{-6})$	4.9(1.0)
180 W	0.97(0.08)	23.1(0.21)	0.82(0.008)	$2.56*10^{-5}(1.81*10^{-6})$	5.5(0.8)

Supplementary Table 2|Specific Energy comparison of multi-layer soft-packaged Li–S pouch cells in this work and literatures.

Ref.	E/S (μL mg^{-1})	Specific energy (Wh kg^{-1})	Sulfur content (%)	Cycle number
This work	2.9	404	70	3
10	3.5	353	63	1
11	-	436	63	1
12	-	307	60	5
13	1.2	415	70	1
14	4.5	410	63	1
15	-	470	65	1

Comparisons with the literature is conducted using studies that involve similar cell types and experimental conditions.

Supplementary Table 3|Comparison of pouch cells' specific energy of this work and prominent literature reports.

Ref.	16	17	18	19	20	21	22	This work
Specific energy (Wh kg^{-1})	240	300	322	331	368	381	402	404

Comparisons with the literature is conducted using studies that involve similar cell types and experimental conditions.

Supplementary Table 4|Comparison of pouch cells' critical performance metrics of this work and prominent literature reports.

Ref.	E/S (μL mg^{-1})	Current (mA)	Specific capacity (mA h g^{-1})	Specific energy (Wh kg^{-1})	Sulfur content (%)
This work	2.9	30	1067	404	70
22	4	100	1275	402	64
16	3.9	100	930	240	58
23	1.2	12	1055	366	70
18	3.5	96	929	322	64
20	4	44	1149	368	64

Comparisons with the literature is conducted using studies that involve similar cell types and experimental conditions.

Supplementary Table 5|Specifications of assembled pouch cells

Metrics	Single-layer	Stacked
Number of positive electrodes	1 (5.2 mg cm^{-2}, single-sided)	2 (9.4 mg cm^{-2} per side, double-sided)
Number of negative electrodes	1 (single-sided)	3 (1*double-sided and 2*single-sided)
Number of separators (Celgard 2500 PP film, 25 μm in thickness, porosity of 55%, average pore size of 0.064 μm)	1	4
Size of positive electrodes (cm*cm)	3*3	4*7
Size of negative electrodes (cm*cm)	4.5*5	4.6*7.6
Size of separators (cm*cm)	5.5*6.5	6.5*8
N/P ratio	2.63	0.82
E/S ratio (μL mg^{-1})	7.8 (366 μL injected)	2.9 (3050 μL injected)

Supplementary References

1. Duan, H. *et al.* Lithium-ion charged polymer channels flattening lithium metal anode. *Nano-Micro Lett.* **16**, 78 (2024).
2. Sheng, L. *et al.* Suppressing electrolyte-lithium metal reactivity via Li^+-desolvation in uniform nano-porous separator. *Nat. Commun.* **13**, 172 (2022).
3. Ely, D.R. *et al*. Heterogeneous nucleation and growth of lithium electrodeposits on negative electrodes. *J. Electrochem. Soc.* **160**, A662 (2013).
4. Dörfler, S. *et al.* Challenges and key parameters of lithium-sulfur batteries on pouch cell level. *Joule* **4**, 539–554 (2020).
5. Nyborg, Wesley L. Acoustic Streaming near a Boundary. *J. Acoust. Soc. Am.* **30**, 4, 329 (1958).
6. Rozenberg, L. D. High-intensity ultrasonic fields. *Springer Science & Business Media* (2013).
7. Bishtawi, B. A. *et al.* On the early stagnation point during transient acoustic cavitation. *Phys. Fluids* **37**, 023312 (2025).
8. Bjerknes, V. Fields of force; supplementary lectures, applications to meteorology; a course of lectures in mathematical physics delivered December 1 to 23, 1905. *Columbia University Press* (1906).
9. Ashokkumar, M. *et al.* Handbook of Ultrasonics and Sonochemistry. *Singapore:*

Springer (2016).

10. Song, Y. W. *et al.* Cationic lithium polysulfides in lithium–sulfur batteries. *Chem* **8**, 3031–3050 (2022).
11. Song, Y. W. *et al.* Reducing the cathode Thiele modulus to promote the discharge capacity of lithium–sulfur batteries. *J. Energy Chem.* **106**, 993–1001 (2025).
12. Xiao, Y. *et al.* Triple-site integrated redox-active metal–organic cages enable complementary acceleration mechanisms for serially enhancing sulfur redox kinetics. *ACS Nano* **19**, 34858–34868 (2025).
13. Xie, Y. *et al*. Semi-flooded sulfur cathode with ultralean absorbed electrolyte in Li–S battery. *Adv. Sci.* **7**, 1903168 (2020).
14. Lin, Y. *et al*. Catalytic disproportionation for suppressing polysulfide shuttle in Li–S pouch cells: beyond adsorption interactions. *Adv. Energy Mater.* **12**, 2201912 (2022).
15. Chen, J. *et al.* Improving lithium–sulfur battery performance under lean electrolyte through nanoscale confinement in soft swellable gels. *Nano Lett.* **17**, 3061–3067 (2017).
16. Luo, Z.-H. *et al.* 2D nanochannel interlayer realizing high-performance lithium-sulfur batteries. *Adv. Mater.* **37**, 2417321 (2025).
17. Zhao, M. *et al.* Redox comediation with organopolysulfides in working lithium-sulfur batteries. *Chem* **6**, 3297-3311 (2020).
18. Li, S. *et al.* Engineering of lignocellulose pulp binder for Ah-scale lithium-sulfur batteries. *Adv. Energy Mater.* **15**, 2405461 (2025).
19. Liu, Y. et al. Surface-localized phase mediation accelerates quasi-solid-state reaction kinetics in sulfur batteries. *Nat. Chem.* **17**, 614–623 (2025).
20. Yang, Z. *et al.* Oxygen Bridges of $CoTe_2$/Co–O–NC Enhancing adsorption-catalysis of polysulfide for stable lithium-sulfur batteries. *Adv. Mater.* **37**, 2417321 (2025).
21. Sander, J. *et al.* High-performance battery electrodes via magnetic templating. *Nat. Energy* **1**, 16099 (2016).
22. Han, Z. *et al.* Machine-learning-assisted design of a binary descriptor to decipher electronic and structural effects on sulfur reduction kinetics. *Nat. Catal.* **6**, 1073–1086 (2023).
23. Xue, W. *et al.* Intercalation-conversion hybrid cathodes enabling Li–S full-cell architectures with jointly superior gravimetric and volumetric energy densities. *Nat. Energy* **4**, 374–382 (2019)
