# Supplementary material for: Unlocking Li‒S chemistry via acoustic-induced entropy-driven electrolyte
Source: Nat Commun. 2026 Apr 30;17:5915. doi: 10.1038/s41467-026-72486-6 (PMC13338023; doi:10.1038/s41467-026-72486-6)
Supplement: Supplementary file 2 — Description of Additional Supplementary Files [file 41467_2026_72486_MOESM2_ESM.pdf]

Description of Additional Supplementary Files

File Name: Supplementary Data 1

Description:

The atomic coordinates for the blank control electrolyte system without ultrasonication, corresponding to the simulation snapshot depicted in Figure 1e.

File Name: Supplementary Data 2

Description:

The atomic coordinates for the optimized cavity-mediated entropy-driven electrolyte system, corresponding to the simulation snapshot depicted in Figure 1d.
