# Supplementary material for: Unlocking Li‒S chemistry via acoustic-induced entropy-driven electrolyte
Source: Nat Commun. 2026 Apr 30;17:5915. doi: 10.1038/s41467-026-72486-6 (PMC13338023; doi:10.1038/s41467-026-72486-6)
Supplement: Supplementary file 3 — Supplementary Data [file 41467_2026_72486_MOESM3_ESM.zip › Supplementary Data files/Supplementary Data 1.docx]

model-blank

O 17.147682 32.846184 32.747910

C 18.360714 33.572731 32.811958

C 18.138741 34.917770 33.439739

C 17.328096 31.611151 32.085411

O 19.378660 35.598358 33.370312

C 19.306789 36.914555 33.870487

H 18.775812 33.751190 31.802811

H 19.128328 33.030510 33.395248

H 17.790134 34.805317 34.483604

H 17.346676 35.452656 32.882854

H 16.345840 31.117823 32.058029

H 18.036554 30.953054 32.618343

H 19.019789 36.936554 34.935371

H 18.599798 37.536469 33.296974

H 20.311537 37.348721 33.769279

H 17.680614 31.743162 31.047907

O 28.329954 1.385622 20.157522

C 28.127050 2.467131 19.254473

C 27.960327 3.776968 19.971729

C 28.901022 0.309002 19.430975

O 27.815113 4.784161 18.985075

C 27.537889 6.049506 19.542452

H 27.246000 2.284760 18.609577

H 28.995386 2.591807 18.584642

H 28.853106 3.972051 20.588268

H 27.095413 3.732965 20.654764

H 29.060413 48.365738 20.124765

H 29.877901 0.581824 18.994364

H 28.288883 6.356064 20.287579

H 26.549768 6.063196 20.024046

H 27.543760 6.774097 18.715675

H 28.247816 48.855152 18.610556

O 48.698208 0.541732 39.839809

C 0.423901 1.770408 39.524937

C 1.199829 1.554791 38.259594

C 47.802002 0.608715 40.927185

O 1.865260 2.759021 37.931522

C 2.513579 2.666613 36.681824

H 1.102044 2.108258 40.329227

H 48.576462 2.572739 39.355770

H 0.493328 1.242855 37.466064

H 1.910731 0.720680 38.404804

H 47.294495 48.526592 40.971188

H 47.036339 1.390511 40.796642

H 1.805611 2.420194 35.870689

H 3.322755 1.917087 36.680847

H 2.951169 3.653759 36.475494

H 48.321243 0.779351 41.884018

O 27.800446 30.347761 3.561351

C 27.928057 28.945515 3.415651

C 26.754141 28.409649 2.648523

C 28.863863 30.837667 4.354882

O 26.959980 27.022074 2.445129

C 25.872114 26.405535 1.788988

H 27.974014 28.441429 4.398396

H 28.855551 28.680517 2.877830

H 26.669556 28.957249 1.695113

H 25.824688 28.612066 3.210790

H 28.713276 31.915752 4.493248

H 29.846121 30.683167 3.874754

H 25.721525 26.801077 0.771039

H 24.928970 26.517500 2.350277

H 26.108753 25.333803 1.715159

H 28.883909 30.367317 5.352295

O 5.360118 47.950146 35.228241

C 4.331902 47.334095 34.471378

C 4.684419 47.366856 33.008995

C 5.004667 48.026909 36.593815

O 3.477745 47.139503 32.304939

C 3.528593 47.271027 30.898293

H 3.378492 47.881695 34.582367

H 4.146598 46.294147 34.801407

H 5.458881 46.617817 32.772842

H 5.112231 48.356934 32.773819

H 5.802598 48.587708 37.099857

H 4.924483 47.028027 37.056831

H 4.091349 46.456959 30.418165

H 3.957872 48.232258 30.569735

H 2.486688 47.219200 30.547243

H 4.051746 48.562775 36.747341

O 24.849766 39.604145 22.757153

C 25.967455 38.791058 22.429573

C 26.526789 38.203854 23.684650

C 24.139353 39.980621 21.594482

O 25.554312 37.320362 24.220026

C 26.031992 36.582573 25.325005

H 26.728228 39.377281 21.880995

H 25.668232 37.954014 21.770496

H 26.760984 39.014011 24.398485

H 27.471395 37.670921 23.473433

H 23.778036 39.104950 21.030260

H 24.749537 40.603516 20.919762

H 26.333174 37.233334 26.164492

H 26.881260 35.931316 25.052670

H 25.203259 35.941586 25.657475

H 23.267105 40.563911 21.920597

O 46.656933 45.747036 46.543503

C 47.190350 46.820232 45.788597

C 46.143066 47.272491 44.812695

C 47.532112 45.328026 47.570248

O 46.570393 48.480145 44.206425

C 45.573467 0.082141 43.338085

H 48.108070 46.525410 45.246864

H 47.448017 47.679768 46.433983

H 45.200901 47.417217 45.373497

H 45.959232 46.480427 44.061703

H 47.040741 44.491959 48.088512

H 47.724751 46.127911 48.306084

H 44.616634 0.249354 43.861240

H 45.395008 48.294842 42.485889

H 45.930382 1.046794 42.951836

H 48.497746 44.972576 47.173237

O 26.866102 14.681041 38.228790

C 26.734583 14.097750 39.523472

C 27.987705 14.335857 40.329227

C 26.127335 13.966717 37.249470

O 27.616119 14.390128 41.693336

C 28.735765 14.545119 42.542114

H 26.519455 13.016729 39.475071

H 25.883848 14.563209 40.049068

H 28.437519 15.291712 40.011425

H 28.740166 13.546727 40.140011

H 26.243698 14.504048 36.299969

H 25.053158 13.899245 37.494423

H 29.318079 15.454524 42.313293

H 29.412931 13.675805 42.496643

H 28.350977 14.632636 43.568863

H 26.510164 12.941436 37.102299

O 33.966805 5.974700 12.566427

C 33.260796 6.577059 13.631313

C 34.223003 6.644043 14.795450

C 33.205055 5.836822 11.384199

O 33.621620 6.020170 15.920962

C 34.491425 6.050484 17.039141

H 32.393925 5.958076 13.918313

H 32.862804 7.564693 13.335022

H 34.538361 7.676659 15.031114

H 35.141701 6.101821 14.499160

H 33.901779 5.515597 10.597025

H 32.742039 6.785343 11.060529

H 34.706066 7.081633 17.370144

H 35.452656 5.543954 16.838192

H 33.989296 5.525375 17.864941

H 32.418861 5.069205 11.486385

O 17.491888 0.120765 45.948963

C 17.885963 0.839000 44.773094

C 18.320621 2.255914 45.078186

C 16.087198 0.082629 46.143066

O 18.405695 3.091003 43.928223

C 19.623613 2.890054 43.231503

H 17.081678 0.869803 44.020145

H 18.713718 0.271844 44.320347

H 19.255451 2.253469 45.666851

H 17.556915 2.709150 45.723080

H 15.651073 1.091287 46.242321

H 15.904828 48.420006 47.074474

H 19.777138 1.840325 42.931789

H 20.497818 3.218613 43.813816

H 19.589878 3.504147 42.326496

H 15.572845 48.443474 45.324116

O 37.645008 30.172726 45.804729

C 38.346622 29.295099 46.682354

C 39.388527 29.985954 47.531136

C 36.625599 30.887049 46.482384

O 40.150276 29.028145 48.253773

C 41.348152 29.631971 48.703094

H 38.860977 28.567085 46.034039

H 37.653324 28.714741 47.314537

H 38.921112 30.708590 48.225903

H 40.030003 30.574623 46.853481

H 36.124935 31.526567 45.740681

H 37.027004 31.525099 47.285202

H 41.160404 30.542843 0.395544

H 42.007713 29.903324 47.859692

H 41.868370 28.904446 0.447370

H 35.872646 30.220150 46.932198

O 44.710018 0.734859 39.695087

C 43.857819 1.697069 40.295002

C 42.873608 0.967100 41.175560

C 45.339760 1.194451 38.511879

O 42.623276 1.717604 42.357788

C 41.573059 1.117200 43.096069

H 43.339558 2.313118 39.538628

H 44.458710 2.376190 40.919361

H 43.308754 48.876175 41.426868

H 41.941708 0.765661 40.617203

H 46.024750 0.398476 38.186253

H 45.926472 2.114614 38.672737

H 41.825832 0.093874 43.426094

H 40.630894 1.073686 42.521088

H 41.390690 1.736672 43.984940

H 44.613213 1.378777 37.700260

O 10.797486 14.407241 2.340498

C 12.089723 14.248828 2.897876

C 13.061712 13.940315 1.795833

C 9.869989 14.741669 3.354046

O 14.328524 13.642070 2.359566

C 15.234996 13.241637 1.346997

H 12.106346 13.413251 3.621979

H 12.409970 15.160191 3.434230

H 13.125272 14.805717 1.109378

H 12.665191 13.085669 1.216453

H 8.871598 14.743624 2.901299

H 10.068494 15.738592 3.786258

H 15.322025 13.991652 0.541243

H 14.948484 12.274049 0.897672

H 16.225565 13.126249 1.810012

H 9.861678 13.999964 4.167622

O 41.290459 30.365362 24.175533

C 42.056610 29.214914 24.485514

C 43.043755 29.623171 25.543554

C 40.082317 30.088629 23.495924

O 43.678871 28.476633 26.077953

C 44.587299 28.828661 27.101278

H 42.560692 28.823772 23.586376

H 41.439091 28.393026 24.885946

H 42.483932 30.173212 26.324373

H 43.775681 30.325758 25.107920

H 39.522984 31.033239 23.459743

H 39.458935 29.345459 24.020544

H 44.102283 29.395819 27.916321

H 45.434612 29.415863 26.710136

H 44.983822 27.893833 27.520290

H 40.245129 29.732201 22.467220

O 20.081249 30.281267 44.553562

C 19.636326 29.132284 45.254684

C 19.634859 27.959837 44.313988

C 20.383896 31.349085 45.430698

O 19.168423 26.823566 45.018047

C 19.241272 25.631073 44.268028

H 18.617401 29.280920 45.655609

H 20.293446 28.893200 46.110798

H 20.664053 27.812668 43.937515

H 18.991920 28.191099 43.444675

H 20.640585 32.218399 44.807316

H 21.248322 31.119778 46.078529

H 20.279755 25.374872 43.996185

H 18.632069 25.679964 43.349823

H 18.838884 24.833143 44.908035

H 19.531694 31.615061 46.078041

O 16.040749 30.317936 5.471104

C 15.972300 29.543964 4.283009

C 17.301205 29.513651 3.584820

C 14.809628 30.271976 6.165871

O 18.126516 28.499123 4.150021

C 18.992407 27.929033 3.180476

H 15.195882 29.948305 3.608289

H 15.693122 28.497656 4.504004

H 17.803335 30.490040 3.672338

H 17.145239 29.335680 2.509179

H 14.901058 30.930075 7.041541

H 14.576410 29.252562 6.519855

H 19.606012 27.175106 3.691406

H 19.657839 28.679050 2.719906

H 18.430630 27.420059 2.377657

H 13.967207 30.633295 5.550311

O 27.090033 16.985359 32.475574

C 27.798491 17.414148 31.310947

C 27.642033 18.896090 31.020037

C 27.868406 17.144260 33.648026

O 28.150518 19.334169 29.758604

C 29.528807 19.666639 29.829496

H 27.358456 16.842102 30.483194

H 28.863863 17.129591 31.351040

H 28.064957 19.492092 31.845346

H 26.564926 19.113663 30.999500

H 28.123627 18.201811 33.832352

H 28.802748 16.557058 33.607933

H 29.742468 20.432791 30.594669

H 29.822653 20.072939 28.851641

H 30.152189 18.786079 30.048048

H 27.264091 16.774143 34.486534

O 47.866051 5.137655 37.646969

C 47.948681 6.408868 37.024563

C 47.536514 7.454196 38.005840

C 48.115402 4.124107 36.693069

O 48.498234 7.550515 39.042854

C 48.050865 8.413473 40.065205

H 47.284714 6.450427 36.140579

H 0.084583 6.610796 36.674000

H 46.556213 7.152039 38.410671

H 47.399124 8.430097 37.507133

H 47.958946 3.156519 37.187374

H 0.253754 4.169578 36.306324

H 48.799904 8.387560 40.868027

H 47.086212 8.085891 40.488617

H 47.956989 9.450978 39.706333

H 47.426994 4.178867 35.833046

O 13.107671 26.164982 11.350952

C 12.971260 26.451494 12.728751

C 13.210345 25.155346 13.457254

C 12.937036 27.284626 10.506574

O 13.382936 25.311314 14.867811

C 12.283338 24.882034 15.654496

H 11.969935 26.849970 12.964415

H 13.717363 27.194662 13.066112

H 14.146642 24.729000 13.068068

H 12.408993 24.442490 13.211323

H 13.107671 26.930155 9.479337

H 13.666026 28.085979 10.717301

H 12.220756 23.782436 15.698990

H 11.322595 25.281488 15.291224

H 12.459841 25.264378 16.667555

H 11.918109 27.704126 10.566712

O 0.323673 45.927452 37.954502

C 48.049400 46.387043 38.631668

C 46.711205 46.200760 37.960857

C 0.465462 44.519337 38.010731

O 45.757305 46.612926 38.925514

C 44.421066 46.461849 38.491833

H 48.195587 47.466599 38.768078

H 47.971657 45.950432 39.642773

H 46.559143 45.144680 37.678257

H 46.641285 46.805077 37.037762

H 1.467272 44.277321 37.630341

H 48.618999 43.994232 37.382458

H 44.179047 45.419456 38.222923

H 44.186378 47.108212 37.629856

H 43.780079 46.760586 39.333279

H 0.378431 44.134552 39.038456

O 45.249798 9.094550 32.049721

C 45.650719 7.905965 31.386244

C 46.835392 7.360322 32.129414

C 43.944359 9.488136 31.678623

O 47.041718 6.005991 31.778852

C 47.864094 5.349361 32.723461

H 45.873180 8.088336 30.319403

H 44.862076 7.132970 31.426334

H 46.582127 7.448818 33.200169

H 47.741863 7.962682 31.947044

H 43.665180 10.333982 32.320095

H 43.208035 8.685806 31.850237

H 47.810802 4.276653 32.495621

H 47.522335 5.487728 33.762924

H 0.020044 5.681343 32.665279

H 43.886665 9.808873 30.624983

O 38.231236 17.722174 6.896818

C 37.843517 17.556425 5.542488

C 37.758930 18.869198 4.818385

C 38.189678 16.448515 7.516290

O 37.571182 18.548950 3.451343

C 37.245560 19.660772 2.645100

H 36.857838 17.056253 5.461326

H 38.572018 16.924730 5.001244

H 38.688381 19.446133 4.967508

H 36.922863 19.470091 5.221262

H 38.560284 16.556080 8.541573

H 38.829681 15.708767 7.001937

H 38.075268 20.384386 2.574695

H 36.343483 20.185881 3.002507

H 37.031895 19.269630 1.639864

H 37.161461 16.045151 7.562738

O 8.768924 20.988213 37.063675

C 8.296130 19.894480 36.306324

C 8.355780 20.261665 34.855675

C 8.560151 20.806332 38.449783

O 7.781778 19.222692 34.080723

C 7.921611 19.511650 32.704395

H 7.246402 19.665663 36.543945

H 8.898978 18.990940 36.500919

H 9.404041 20.440125 34.579922

H 7.817958 21.217518 34.713886

H 8.846663 21.745562 38.939201

H 9.178156 19.993242 38.864395

H 7.462997 20.478260 32.434994

H 7.400903 18.717630 32.152393

H 8.980629 19.528273 32.390011

H 7.503578 20.609781 38.696209

O 10.934875 19.075525 12.512156

C 10.465992 17.822403 12.048163

C 9.325324 17.365744 12.911610

C 12.033985 19.505783 11.734760

O 8.938581 16.083286 12.453974

C 7.948013 15.472616 13.252394

H 11.260500 17.054296 12.087277

H 10.121299 17.879608 10.997947

H 8.493657 18.091803 12.848050

H 9.664150 17.342766 13.963294

H 12.311696 20.509064 12.087767

H 11.783653 19.569832 10.661564

H 7.011227 16.055416 13.271461

H 8.292218 15.311269 14.288431

H 7.740708 14.490849 12.801601

H 12.904766 18.838396 11.848192

O 27.054342 11.417935 33.041264

C 28.245371 11.508387 32.284405

C 29.260872 12.344944 33.004105

C 26.077953 10.690411 32.324009

O 30.437723 12.322452 32.217422

C 31.540255 12.901833 32.883831

H 28.065933 11.953800 31.287479

H 28.686872 10.507551 32.128437

H 29.430531 11.918109 34.009342

H 28.885866 13.376581 33.139538

H 25.196413 10.600937 32.974770

H 26.427536 9.677841 32.062431

H 31.817968 12.351299 33.798126

H 31.350554 13.945694 33.169365

H 32.390991 12.875919 32.188084

H 25.773838 11.205741 31.395535

O 12.840227 5.926296 3.754478

C 13.718830 4.824741 3.573086

C 14.449288 4.540674 4.861411

C 12.544426 6.573148 2.530691

O 15.385097 3.499747 4.628681

C 16.353174 3.374581 5.649563

H 14.481558 5.037426 2.803024

H 13.163409 3.932937 3.234259

H 13.748167 4.270786 5.667653

H 14.941151 5.475994 5.178236

H 11.916641 7.441973 2.770266

H 11.995359 5.917984 1.832502

H 15.900428 3.119849 6.619596

H 16.957491 4.290832 5.766417

H 17.024961 2.557093 5.351806

H 13.454809 6.943266 2.026118

O 27.488508 29.454489 46.704357

C 26.574213 29.100994 45.671253

C 25.561644 30.183969 45.381317

C 27.009848 29.158688 48.001484

O 24.388706 29.568899 44.876255

C 23.559484 30.508129 44.221581

H 26.036882 28.168608 45.903984

H 27.185375 28.889776 44.779938

H 25.983101 30.924696 44.678242

H 25.326471 30.728634 46.312241

H 27.708038 29.632458 48.702606

H 26.993225 28.074734 48.193142

H 24.037657 30.904161 43.310219

H 23.289108 31.356909 44.872345

H 22.644699 29.978130 43.926758

H 26.002169 29.565964 48.188251

O 12.919923 48.417072 36.497986

C 13.804393 48.257191 37.595627

C 13.035799 48.645889 38.827240

C 13.539393 48.086067 35.271751

O 13.824440 48.559349 40.009468

C 12.969304 48.511436 41.137913

H 14.703531 48.889866 37.489044

H 14.150064 47.211376 37.682171

H 12.150839 47.985840 38.876133

H 12.659325 0.780329 38.686916

H 12.809425 48.285061 34.475292

H 13.822483 47.021183 35.224815

H 12.334675 47.606430 41.131557

H 12.318541 0.508485 41.201473

H 13.597088 48.478188 42.039005

H 14.438532 48.695763 35.077160

O 9.242207 6.724226 40.190369

C 8.246748 5.722902 40.327271

C 7.621899 5.477460 38.973915

C 10.097342 6.756496 41.319305

O 7.988594 4.174468 38.548550

C 7.685459 3.950049 37.185421

H 7.522157 6.022615 41.105156

H 8.696074 4.768515 40.651917

H 8.025264 6.241166 38.288441

H 6.521810 5.584536 38.966095

H 10.878648 7.502600 41.116890

H 10.597025 5.786952 41.496296

H 8.231103 4.642860 36.520966

H 6.604439 4.030233 36.979580

H 8.007663 2.927212 36.942421

H 9.560987 7.046919 42.238491

O 39.199314 32.443794 7.118792

C 39.252605 31.054262 6.865527

C 37.892899 30.516439 7.187730

C 40.429455 33.110203 6.951089

O 37.922722 29.101484 7.239556

C 36.643684 28.643847 7.631188

H 39.989910 30.551153 7.516779

H 39.517605 30.835222 5.816776

H 37.163906 30.881180 6.438204

H 37.603451 30.946209 8.164608

H 40.276909 34.141354 7.303607

H 40.745792 33.143940 5.895983

H 35.851624 28.994896 6.946689

H 36.379177 28.974852 8.650114

H 36.661289 27.546202 7.612609

H 41.229343 32.645233 7.546604

O 23.144384 24.957819 8.561130

C 22.081944 24.020054 8.419340

C 22.146971 23.283730 7.107057

C 22.822182 25.820288 9.638726

O 20.829800 22.826582 6.840591

C 20.696812 22.287783 5.540043

H 21.104088 24.537342 8.418851

H 22.059942 23.308664 9.264697

H 22.869118 22.445217 7.125637

H 22.485798 23.992674 6.331129

H 23.610332 26.578613 9.718422

H 22.763510 25.279043 10.598492

H 21.348551 21.411135 5.382119

H 20.911451 23.034376 4.755803

H 19.650015 21.970469 5.427590

H 21.864861 26.348818 9.478358

O 39.455021 6.221120 37.505180

C 40.539951 6.784854 36.783520

C 41.847347 6.541368 37.476330

C 38.304573 6.255834 36.678402

O 42.796844 7.389169 36.850502

C 44.141396 7.110480 37.165371

H 40.409897 7.876141 36.701378

H 40.604492 6.387355 35.752857

H 42.121147 5.474527 37.402500

H 41.739780 6.780453 38.546108

H 37.430370 6.048039 37.306183

H 38.357376 5.495061 35.879490

H 44.400040 6.047061 37.022118

H 44.382927 7.400903 38.197010

H 44.750599 7.711861 36.475494

H 38.158875 7.242002 36.206097

O 43.037399 14.209715 42.066387

C 42.591496 13.105226 41.292904

C 41.561321 13.530594 40.295490

C 44.115974 13.826395 42.897564

O 42.239956 14.153487 39.219360

C 41.366241 14.440977 38.146164

H 42.207195 12.308762 41.952957

H 43.425117 12.674970 40.714989

H 40.838200 14.219004 40.769749

H 40.990257 12.654923 39.936127

H 44.338432 14.673219 43.561039

H 45.020977 13.591710 42.313293

H 40.512085 15.065338 38.457611

H 40.972164 13.524237 37.674347

H 41.944153 14.999822 37.396633

H 43.870045 12.954636 43.522903

O 48.697227 28.362223 33.639225

C 0.614582 27.487532 34.401951

C 2.020251 27.553047 33.859730

C 47.388859 28.428228 34.170689

O 2.438773 26.236855 33.537037

C 3.826839 26.182093 33.279861

H 0.259621 26.449049 34.312477

H 0.560800 27.732973 35.476616

H 2.712573 28.029263 34.573078

H 2.021717 28.188164 32.957169

H 46.826099 29.149397 33.562462

H 47.377121 28.781725 35.216507

H 4.421865 26.394777 34.182911

H 4.137797 26.882729 32.487309

H 4.048324 25.157789 32.957661

H 46.871082 27.455263 34.130596

O 4.287899 24.240072 9.466135

C 5.575735 23.810793 9.899815

C 6.164404 24.890835 10.766684

C 3.734921 23.303776 8.559663

O 7.574961 24.778870 10.793574

C 8.151896 25.856958 11.508876

H 6.256811 23.661182 9.042723

H 5.525864 22.845650 10.440569

H 5.746371 24.840963 11.786588

H 5.863225 25.855490 10.322248

H 2.770266 23.705185 8.216924

H 3.553529 22.323475 9.036367

H 7.859029 25.857935 12.574250

H 7.889831 26.836769 11.071774

H 9.244162 25.743526 11.450694

H 4.374928 23.150742 7.671280

O 12.997662 28.780746 15.842245

C 13.187366 29.820698 16.787832

C 14.631659 30.239706 16.716448

C 11.677556 28.283506 15.875004

O 14.955330 31.077730 17.814091

C 16.288635 31.534878 17.706038

H 12.955125 29.476490 17.810669

H 12.522424 30.682186 16.594215

H 14.830653 30.734015 15.751305

H 15.250154 29.324434 16.722803

H 11.579282 27.574074 15.044803

H 10.924118 29.076548 15.733703

H 16.420647 32.196884 16.832813

H 17.013227 30.706635 17.628298

H 16.517942 32.105946 18.616911

H 11.462916 27.753510 16.818146

O 33.652424 24.882524 33.007042

C 33.025131 23.680250 32.605141

C 33.287193 23.459743 31.143736

C 33.511616 25.093252 34.395107

O 32.684345 22.241335 30.738415

C 33.128292 21.858503 29.453512

H 31.937754 23.723763 32.784576

H 33.413338 22.812403 33.166428

H 34.378971 23.456322 30.986300

H 32.884319 24.305099 30.562401

H 33.972672 26.064753 34.620502

H 34.027920 24.310966 34.977909

H 34.211269 21.643864 29.440800

H 32.914143 22.626120 28.689808

H 32.588520 20.938829 29.190956

H 32.454060 25.129919 34.706066

O 44.482178 1.395890 30.493950

C 45.480572 0.390653 30.505196

C 46.798233 1.051195 30.790731

C 43.175274 0.857091 30.504707

O 47.778530 0.056716 31.035194

C 0.058669 0.630717 31.570080

H 45.514797 48.734386 29.547386

H 45.300156 48.540771 31.303127

H 46.649109 1.700492 31.673733

H 47.090122 1.706359 29.948305

H 42.483444 1.698047 30.648451

H 43.022728 0.147167 31.336372

H 48.780346 1.054129 32.574825

H 0.468394 1.415447 30.919319

H 0.811131 48.728519 31.644398

H 42.925434 0.353984 29.556187

O 1.854015 26.865128 8.461388

C 0.552978 27.411747 8.665760

C 48.521214 26.367399 9.232428

C 2.583984 27.747643 7.621899

O 47.284714 26.990294 9.522850

C 46.331795 26.125866 10.104675

H 0.120765 27.798491 7.724573

H 0.594047 28.274216 9.358571

H 0.084585 25.942520 10.138900

H 48.398983 25.538664 8.510281

H 3.631757 27.416147 7.603808

H 2.555627 28.790037 7.986150

H 46.669643 25.713213 11.070308

H 46.060440 25.291756 9.435332

H 45.432167 26.731159 10.287045

H 2.197731 27.746664 6.585860

O 27.550602 47.790268 23.477345

C 27.526157 46.789433 22.460375

C 26.305794 46.869617 21.594482

C 26.998604 47.254398 24.673262

O 26.378645 48.050865 20.796553

C 25.812464 47.815693 19.514093

H 27.662077 45.775883 22.877430

H 28.403294 46.973267 21.822323

H 25.385630 46.875973 22.204176

H 26.247122 45.970478 20.965231

H 26.992249 48.044022 25.434525

H 27.602919 46.414913 25.060984

H 24.748068 47.528202 19.571299

H 26.349796 47.023136 18.962093

H 25.901939 48.744656 18.935204

H 25.961098 46.902863 24.532942

O 22.754709 1.123066 45.452213

C 22.719505 0.795488 44.076370

C 23.342888 48.326130 43.938004

C 22.463797 2.473489 45.722103

O 22.707283 47.631855 42.879963

C 23.207945 46.311749 42.816891

H 23.244614 1.545012 43.457386

H 21.678089 0.743661 43.717983

H 23.204521 47.796623 44.898746

H 24.434175 48.398983 43.774212

H 22.689680 2.648524 46.779163

H 21.401846 2.705730 45.566624

H 22.928766 45.718678 43.706253

H 24.306566 46.289745 42.716171

H 22.774265 45.837490 41.928509

H 23.083267 3.166296 45.130989

O 27.027939 41.999893 25.139700

C 25.665297 42.299603 24.885946

C 25.543554 42.902451 23.512547

C 27.183907 41.293392 26.356152

O 24.233715 43.426094 23.355602

C 23.950626 43.754654 22.009583

H 25.271223 43.017841 25.627161

H 25.030180 41.394600 24.939728

H 25.771397 42.126034 22.759598

H 26.301880 43.697449 23.397648

H 28.253683 41.065063 26.463717

H 26.627508 40.339005 26.360064

H 23.991697 42.874096 21.345619

H 24.637081 44.516891 21.608171

H 22.927301 44.155087 21.985136

H 26.862192 41.885483 27.229868

O 15.124009 22.085365 37.805866

C 14.514317 22.724884 36.697956

C 14.336836 24.177979 37.016739

C 15.302958 20.708055 37.534512

O 13.861597 24.829231 35.857491

C 13.714430 26.218273 36.068707

H 13.528149 22.282404 36.460339

H 15.139655 22.644211 35.788551

H 15.308336 24.588188 37.343830

H 13.635224 24.288475 37.858673

H 15.828555 20.267532 38.392582

H 15.914607 20.535954 36.631462

H 14.659528 26.688133 36.383575

H 12.943879 26.446604 36.825081

H 13.408851 26.664179 35.110409

H 14.340746 20.180014 37.411793

O 14.996399 11.816412 45.406742

C 15.548887 10.638584 44.830296

C 15.230107 10.604360 43.355202

C 15.011556 11.711781 46.819256

O 15.998214 9.706688 42.563137

C 15.774773 8.352845 42.908810

H 15.129387 9.735535 45.305046

H 16.642132 10.608271 44.982353

H 15.426656 11.603239 42.939121

H 14.153487 10.406343 43.234436

H 14.639972 12.658834 47.233868

H 16.026571 11.534789 47.219200

H 16.342419 7.741686 42.192528

H 14.708909 8.071712 42.847202

H 16.141958 8.120605 43.920403

H 14.347591 10.903584 47.172752

O 31.964647 46.312725 30.511063

C 32.365078 46.800186 31.785208

C 32.980637 45.684452 32.589985

C 31.388199 47.372723 29.769846

O 32.744484 45.938698 33.964848

C 33.109226 44.816120 34.744690

H 31.487452 47.166397 32.348454

H 33.066200 47.651409 31.689869

H 34.062637 45.579823 32.388058

H 32.500023 44.739845 32.282448

H 31.154491 46.996250 28.766079

H 32.080032 48.226391 29.654461

H 34.179489 44.564808 34.643970

H 32.512733 43.924313 34.484089

H 32.911209 45.060093 35.797840

H 30.451900 47.735020 30.228951

O 43.326843 20.937853 6.872861

C 42.106476 21.572969 7.230268

C 40.941853 20.842512 6.611773

C 44.400528 21.703512 7.391613

O 39.658905 21.324594 7.015139

C 39.257496 22.460863 6.263657

H 42.095234 22.622698 6.890951

H 41.975933 21.580793 8.327910

H 40.981457 19.799139 6.947667

H 41.051861 20.827354 5.512174

H 45.338291 21.213608 7.095323

H 44.369728 21.765120 8.495124

H 39.266785 22.266270 5.177258

H 39.891148 23.341423 6.463628

H 38.225368 22.704838 6.555546

H 44.409328 22.733196 6.987759

O 35.437012 28.239502 23.124338

C 36.738049 27.843962 23.520370

C 36.952198 26.422646 23.087667

C 35.148056 29.547386 23.583441

O 38.316311 26.075998 23.261238

C 38.552464 24.711887 22.979126

H 36.874462 27.921211 24.614103

H 37.503712 28.482010 23.045132

H 36.650532 26.340019 22.029629

H 36.281391 25.765039 23.672426

H 34.160912 29.824120 23.186432

H 35.884380 30.289577 23.228970

H 38.404804 24.479158 21.912287

H 37.914410 24.041567 23.582464

H 39.600231 24.510448 23.240213

H 35.104053 29.598234 24.685974

O 42.264893 29.313189 10.847357

C 43.187500 28.746031 11.763119

C 44.024544 29.879856 12.313163

C 41.169693 28.483965 10.518308

O 45.387672 29.641258 12.007093

C 46.169960 30.766283 12.364500

H 43.846577 28.036108 11.238009

H 42.670700 28.174477 12.552248

H 43.898888 30.033869 13.400049

H 43.671535 30.807354 11.827168

H 40.544353 29.059923 9.819140

H 40.554623 28.233147 11.397889

H 46.254543 30.876780 13.459209

H 45.771484 31.704536 11.943533

H 47.174217 30.622049 11.943044

H 41.489941 27.550116 10.028891

O 30.867491 21.549011 25.227707

C 30.127741 21.540213 24.020054

C 28.695673 21.190628 24.324656

C 32.182709 22.033541 25.028713

O 28.102114 20.653296 23.153675

C 26.786411 20.198593 23.393250

H 30.529152 20.783840 23.321865

H 30.180058 22.516602 23.512058

H 28.143673 22.076077 24.686462

H 28.697140 20.457724 25.149967

H 32.676037 22.046253 26.011459

H 32.195419 23.062244 24.626814

H 26.108753 21.017059 23.687096

H 26.753651 19.415819 24.170645

H 26.418245 19.765892 22.452551

H 32.766487 21.385712 24.353014

O 21.870237 26.943354 32.141636

C 20.593159 27.539846 31.997402

C 19.766869 26.619684 31.144714

C 22.845650 27.824404 32.662346

O 18.534283 27.237688 30.823000

C 17.813602 26.463226 29.884747

H 20.652319 28.517704 31.486963

H 20.111565 27.709503 32.977703

H 19.625570 25.651606 31.657110

H 20.350649 26.411890 30.233351

H 23.772167 27.243559 32.766487

H 22.571850 28.227770 33.651447

H 17.538826 25.476082 30.291044

H 18.371469 26.314592 28.942583

H 16.890507 27.010338 29.653971

H 23.053932 28.668295 31.982735

O 7.276715 15.711212 42.344585

C 7.544648 16.393755 41.131557

C 9.014854 16.550701 40.886604

C 5.880337 15.724413 42.565582

O 9.168378 17.265026 39.671127

C 10.526131 17.566204 39.412975

H 7.110480 17.409748 41.142315

H 7.113414 15.845668 40.274956

H 9.490092 15.557200 40.819618

H 9.465158 17.092922 41.738316

H 5.678899 15.165569 43.489166

H 5.331271 15.239397 41.742226

H 11.140224 16.657288 39.290745

H 10.974478 18.191545 40.206017

H 10.558888 18.134829 38.472279

H 5.491150 16.750185 42.687817

O 12.611898 18.467789 1.104488

C 13.767724 19.287720 0.946076

C 13.921247 19.756603 48.408272

C 12.260358 18.432096 2.482776

O 15.194415 19.352259 47.924721

C 15.688233 20.189304 46.891617

H 14.678596 18.731810 1.231610

H 13.705629 20.148722 1.627641

H 13.794126 20.850822 48.328575

H 13.139451 19.297010 47.789780

H 11.503498 17.650301 2.627499

H 11.849659 19.395773 2.832849

H 15.741036 21.249788 47.199154

H 15.081472 20.120853 45.972923

H 16.710581 19.850965 46.669155

H 13.119893 18.213057 3.129139

O 15.189526 8.763057 32.562115

C 15.772327 9.124374 33.802036

C 17.212221 9.492537 33.563927

C 13.860130 8.309331 32.699993

O 17.894764 9.426532 34.801895

C 19.290165 9.582989 34.644947

H 15.762061 8.278529 34.513428

H 15.226685 9.957019 34.282166

H 17.302183 10.492884 33.102379

H 17.627810 8.765012 32.845203

H 13.507124 8.038465 31.694756

H 13.196656 9.094061 33.100426

H 19.553207 10.567201 34.220558

H 19.728245 8.793859 34.008366

H 19.736067 9.507694 35.647251

H 13.790703 7.416059 33.343910

O 23.319420 4.771448 16.959934

C 23.882666 5.693566 16.043682

C 24.513384 6.808811 16.824501

C 22.859829 3.611222 16.295969

O 24.986664 7.796445 15.925851

C 25.645741 8.841285 16.612307

H 23.109180 6.115511 15.375808

H 24.648817 5.217350 15.403677

H 25.329405 6.394200 17.444462

H 23.753590 7.226356 17.509979

H 22.400726 2.961926 17.054296

H 23.686117 3.058733 15.814376

H 26.602081 8.507835 17.051853

H 25.021868 9.273986 17.412193

H 25.857445 9.629926 15.875981

H 22.095634 3.838574 15.534710

O 21.203341 18.667271 38.593044

C 20.983812 19.490137 37.450417

C 21.859972 20.721746 37.402992

C 20.532042 19.149355 39.743492

O 21.785654 21.372997 36.128845

C 21.644842 22.785023 36.226631

H 19.922348 19.782026 37.357521

H 21.225342 18.858442 36.582573

H 22.904810 20.423500 37.581451

H 21.569059 21.394510 38.221458

H 20.794107 18.483433 40.576622

H 19.436354 19.135664 39.614414

H 22.496555 23.259771 36.741959

H 20.714901 23.068600 36.748318

H 21.594482 23.181543 35.201839

H 20.842512 20.171701 40.017288

O 16.846992 28.311377 21.338774

C 16.375175 29.088772 20.250908

C 15.073649 28.528950 19.807940

C 18.033621 28.914713 21.818901

O 15.234507 27.161907 19.419731

C 14.384262 26.809389 18.337732

H 17.089010 29.069214 19.406530

H 16.250988 30.149744 20.538399

H 14.661973 29.129353 18.993385

H 14.352482 28.606201 20.637651

H 18.418406 28.312841 22.653990

H 17.857117 29.940485 22.185596

H 13.335510 27.104700 18.512281

H 14.430221 25.719080 18.224302

H 14.713310 27.268982 17.391169

H 18.808083 28.960184 21.036615

O 32.194439 46.277523 21.012657

C 33.129761 47.220177 20.524708

C 32.386589 48.483566 20.198593

C 32.809513 45.026356 21.235121

O 33.320442 0.649298 20.095428

C 32.761597 1.831033 19.561520

H 33.663181 46.843212 19.632416

H 33.897869 47.451439 21.284990

H 31.652710 48.673271 21.000435

H 31.809166 48.342266 19.270119

H 32.061943 44.379993 21.710848

H 33.677849 45.100674 21.912775

H 33.565395 2.580074 19.540985

H 31.940199 2.226577 20.179035

H 32.390503 1.683380 18.532814

H 33.126827 44.556984 20.288555

O 28.077179 14.704998 48.196079

C 28.329464 15.695077 0.290423

C 29.264297 16.732094 48.613132

C 26.996649 13.882133 48.593575

O 29.993288 17.480154 0.687922

C 29.166510 18.352402 1.440382

H 28.798347 15.242330 1.180761

H 27.396101 16.175205 0.629739

H 28.719631 17.392635 47.913475

H 30.023111 16.201118 48.020550

H 26.880774 13.093492 47.838669

H 26.048616 14.446845 48.648827

H 29.833410 19.018322 2.004605

H 28.526991 17.806269 2.155195

H 28.518194 18.977741 0.805753

H 27.173639 13.404940 0.680099

O 30.101831 6.585860 8.198344

C 30.619118 7.895209 8.345512

C 30.834732 8.439875 6.953045

C 29.985954 5.859313 9.401596

O 30.272955 9.739935 6.873838

C 30.365852 10.222507 5.547866

H 31.538790 7.904988 8.956183

H 29.887678 8.540105 8.859864

H 30.327713 7.754887 6.249967

H 31.901085 8.452587 6.659199

H 29.486269 4.913726 9.147842

H 29.380173 6.387355 10.154546

H 29.790384 9.600591 4.838921

H 31.411179 10.280200 5.195838

H 29.944885 11.235565 5.538087

H 30.972122 5.620716 9.829897

O 32.571407 18.278572 5.108809

C 32.770889 16.931087 4.722555

C 32.259960 16.792721 3.312976

C 33.020729 18.529394 6.420114

O 33.385468 16.580036 2.478376

C 33.051533 16.723293 1.114756

H 32.285873 16.242678 5.434924

H 33.845551 16.676357 4.724511

H 31.738272 17.729019 3.049444

H 31.514832 15.986479 3.180965

H 32.691193 19.545385 6.682178

H 34.120331 18.478054 6.492964

H 32.782619 17.763243 0.859047

H 32.218399 16.065195 0.817488

H 33.942360 16.442648 0.538310

H 32.579716 17.821915 7.140304

O 40.125832 30.408388 15.079028

C 40.891495 30.640141 13.913424

C 40.167881 31.649286 13.062690

C 40.674408 29.385550 15.880382

O 40.298424 31.241520 11.712759

C 39.401241 31.967089 10.900649

H 40.973633 29.715578 13.317910

H 41.918240 30.960386 14.165711

H 40.541912 32.679947 13.195189

H 39.108376 31.652710 13.377070

H 40.114098 29.399731 16.825480

H 41.740273 29.557163 16.104311

H 39.571388 33.057888 10.947098

H 38.358845 31.762230 11.193028

H 39.542542 31.636087 9.863144

H 40.566357 28.392048 15.415411

O 34.018631 13.401517 23.687584

C 34.660595 12.168440 23.411339

C 35.906384 12.102434 24.220026

C 32.825161 13.593665 22.956148

O 35.513775 12.223200 25.578756

C 36.597725 11.984115 26.453938

H 34.872787 12.050609 22.337652

H 34.015697 11.325039 23.705185

H 36.599194 12.916988 23.939383

H 36.450069 11.158804 24.039124

H 32.488777 14.618459 23.165409

H 32.030651 12.892542 23.258305

H 36.367443 12.484776 27.399036

H 37.546246 12.391391 26.074530

H 36.726803 10.907984 26.644621

H 32.967438 13.505169 21.865837

O 16.679289 46.939041 36.846104

C 17.340809 46.171425 37.846451

C 16.481762 45.115341 38.487434

C 16.559992 46.216896 35.633560

O 17.055275 44.757446 39.737621

C 16.630886 43.469608 40.142456

H 17.622433 46.880371 38.636555

H 18.283463 45.728458 37.475353

H 16.382999 44.246517 37.817604

H 15.471637 45.526531 38.637047

H 16.205517 46.917042 34.865944

H 15.827578 45.400387 35.724991

H 16.909575 42.698082 39.402706

H 15.541553 43.421696 40.301357

H 17.137415 43.237858 41.089508

H 17.520245 45.792995 35.292290

O 41.478207 8.339156 40.628937

C 41.108574 9.697398 40.474438

C 39.604145 9.785893 40.521862

C 42.877029 8.174876 40.554134

O 39.257004 10.887448 41.344727

C 37.852806 11.026304 41.429802

H 41.524654 10.123743 39.542049

H 41.509010 10.298779 41.308060

H 39.223759 8.843241 40.953098

H 39.151886 9.876346 39.515652

H 43.073090 7.096301 40.526752

H 43.388447 8.600732 41.431271

H 37.376591 10.150145 41.900639

H 37.387348 11.184717 40.440212

H 37.652836 11.909308 42.052696

H 43.294575 8.623222 39.637882

O 16.115067 23.863110 16.612307

C 15.585557 22.648613 17.119814

C 15.589470 22.757643 18.604198

C 16.095510 23.900267 15.200282

O 16.941355 22.790400 19.032501

C 17.048918 22.925344 20.437679

H 16.201118 21.779785 16.821079

H 14.563699 22.475531 16.735518

H 15.036981 21.925976 19.074549

H 15.066316 23.687096 18.883865

H 16.280813 24.939728 14.893724

H 15.119610 23.589798 14.795450

H 16.441181 22.190975 20.995058

H 16.760450 23.935959 20.775040

H 18.102558 22.746397 20.688499

H 16.874861 23.259771 14.760736

O 5.451058 21.295748 14.860967

C 5.262821 19.931149 14.543652

C 6.023593 19.095572 15.529331

C 4.703487 22.125948 13.998009

O 5.898916 17.737329 15.148457

C 6.456783 16.886106 16.125824

H 5.628539 19.699886 13.526682

H 4.194513 19.654417 14.583255

H 5.619739 19.277943 16.542879

H 7.083100 19.415331 15.533731

H 4.950396 23.164431 14.257140

H 3.617578 21.978291 14.129042

H 5.906250 16.941843 17.081678

H 7.523135 17.105146 16.304281

H 6.379533 15.858869 15.746414

H 4.961641 21.967535 12.937036

O 17.115902 46.812901 43.561039

C 17.961748 45.690319 43.772747

C 19.319012 46.137691 44.212292

C 15.994791 46.422245 42.791466

O 19.275497 46.681866 45.525063

C 20.580446 47.085232 45.898117

H 18.064423 45.096272 42.845249

H 17.520245 45.022934 44.534496

H 19.698910 46.876949 43.480366

H 20.017200 45.283043 44.181488

H 15.367007 47.309650 42.650658

H 15.391943 45.639961 43.284309

H 20.941763 47.925701 45.282066

H 21.317261 46.263832 45.833576

H 20.539865 47.414772 46.943932

H 16.282768 46.052620 41.792099

O 0.331005 7.432194 24.966131

C 48.651272 6.177605 25.289801

C 47.451927 6.367798 26.174273

C 1.413003 7.271337 24.072859

O 46.942955 5.079473 26.468607

C 45.862911 5.107831 27.377033

H 0.480127 5.528798 25.817842

H 48.331997 5.631961 24.383816

H 46.698490 6.993137 25.663342

H 47.755062 6.909531 27.088568

H 1.824680 8.273150 23.884623

H 1.095689 6.834236 23.110159

H 44.982841 5.619739 26.954111

H 46.131824 5.586492 28.335333

H 45.591068 4.062503 27.579941

H 2.208485 6.635731 24.497736

O 24.120773 13.371202 16.831347

C 24.389194 12.084833 17.358410

C 24.013210 12.117103 18.810528

C 24.401907 13.451386 15.449636

O 24.121752 10.832688 19.403597

C 23.546284 10.858113 20.695345

H 25.451637 11.815434 17.241556

H 23.799059 11.306949 16.845036

H 22.976192 12.495532 18.863819

H 24.659573 12.850494 19.326834

H 24.223450 14.492804 15.149922

H 23.744787 12.796712 14.850698

H 22.472597 11.098665 20.652807

H 24.045479 11.581238 21.363708

H 23.651403 9.857766 21.134401

H 25.453102 13.204477 15.220329

O 39.421772 4.190602 23.673405

C 39.027210 2.932101 24.191179

C 38.045933 2.306273 23.240704

C 40.332649 4.846743 24.530985

O 37.799023 0.968566 23.638201

C 36.903305 0.313892 22.761553

H 38.575439 3.033309 25.193972

H 39.888702 2.249069 24.288475

H 38.481079 2.352721 22.227644

H 37.115013 2.898854 23.220169

H 40.546307 5.826066 24.082148

H 41.282146 4.295232 24.631704

H 37.327694 0.193127 21.749474

H 35.936207 0.839978 22.675991

H 36.713600 48.207809 23.180565

H 39.917061 5.008090 25.539644

O 37.248978 14.208735 2.277915

C 37.998997 14.499160 3.442542

C 38.711849 13.248971 3.872310

C 36.488697 15.328871 1.870639

O 39.756691 13.600021 4.764114

C 40.464172 12.444195 5.169436

H 37.355080 14.868789 4.261985

H 38.756344 15.278022 3.238659

H 39.107395 12.751730 2.969260

H 37.996063 12.544914 4.339725

H 35.952831 15.042848 0.954876

H 37.120392 16.205517 1.645732

H 41.079731 12.037407 4.350970

H 39.792873 11.645776 5.531732

H 41.124222 12.734618 5.997680

H 35.744549 15.622717 2.631899

O 10.494350 25.435991 27.164349

C 9.733579 26.503319 27.701193

C 10.215661 27.798000 27.113012

C 10.296824 24.255228 27.912901

O 9.504760 28.860931 27.724174

C 10.159435 30.106718 27.593628

H 9.850432 26.578613 28.798838

H 8.658426 26.362997 27.491932

H 10.065560 27.807291 26.021727

H 11.301571 27.864985 27.298807

H 10.963233 23.494457 27.485577

H 9.255407 23.894890 27.855694

H 10.265532 30.418655 26.540478

H 11.154892 30.105253 28.067888

H 9.542896 30.843044 28.126070

H 10.564267 24.391150 28.975342

O 20.383408 29.634903 40.250507

C 21.423357 29.048191 39.490711

C 20.847891 28.695183 38.148609

C 20.820021 29.993288 41.544209

O 21.770008 27.887962 37.438683

C 21.221432 27.397570 36.233963

H 21.801298 28.125584 39.968887

H 22.285337 29.731222 39.379238

H 20.600492 29.618771 37.594162

H 19.893991 28.165676 38.325596

H 19.946306 30.399588 42.074207

H 21.605728 30.768238 41.523678

H 20.926607 28.206745 35.545067

H 20.341848 26.761473 36.415844

H 21.999804 26.790321 35.750416

H 21.202852 29.127398 42.110390

O 16.710581 26.181604 6.100355

C 16.001637 25.568489 7.156929

C 16.460249 26.223164 8.424229

C 16.424070 25.591957 4.850655

O 15.810953 25.625692 9.531162

C 16.267612 26.235386 10.720724

H 16.208452 24.484045 7.211199

H 14.909859 25.697077 7.042030

H 16.244633 27.307117 8.359692

H 17.559849 26.114134 8.489745

H 17.047453 26.101421 4.103573

H 15.366517 25.720545 4.565121

H 16.026083 27.311518 10.747126

H 17.356455 26.110710 10.855668

H 15.756193 25.750858 11.562658

H 16.671955 24.516315 4.827675

O 39.116196 21.728939 47.762398

C 39.704376 21.321661 46.539101

C 38.794971 21.691778 45.402344

C 40.029026 21.654621 48.835106

O 39.243317 21.022436 44.237225

C 38.453697 21.383755 43.121490

H 39.857410 20.227930 46.526390

H 40.690544 21.793964 46.374821

H 38.793503 22.788935 45.265442

H 37.763332 21.396467 45.660007

H 39.490227 21.975357 0.844377

H 40.884647 22.329830 48.677181

H 38.505035 22.466730 42.910767

H 37.391258 21.109467 43.249592

H 38.849728 20.839579 42.251690

H 40.409409 20.631783 0.100232

O 28.970451 19.561031 45.761703

C 29.070190 20.352606 46.931709

C 27.693861 20.518841 47.509624

C 30.204506 19.472536 45.081116

O 27.775999 21.392067 48.622421

C 26.548302 21.473719 0.427321

H 29.483826 21.355885 46.715603

H 29.729755 19.883234 47.685146

H 27.294405 19.527784 47.794666

H 27.030384 20.929052 46.725872

H 30.019690 18.904890 44.157532

H 30.970165 18.944494 45.675163

H 26.307261 20.533508 0.953900

H 25.701477 21.739204 48.664471

H 26.654888 22.268715 1.177830

H 30.599560 20.465548 44.806339

O 26.455894 1.120134 14.885901

C 27.171684 1.268768 13.670916

C 26.557100 2.432906 12.938013

C 26.937977 0.048893 15.669653

O 27.563316 3.125716 12.223200

C 27.039675 4.266386 11.575859

H 27.148216 0.345183 13.065133

H 28.235102 1.492697 13.860620

H 26.097998 3.090514 13.697807

H 25.748415 2.095546 12.262803

H 26.288679 48.871288 16.552658

H 27.971083 0.212195 16.012392

H 26.656843 5.008578 12.296050

H 26.236366 4.010676 10.863980

H 27.865475 4.720600 11.012614

H 26.899841 47.978504 15.135745

O 21.897129 0.655161 10.394608

C 21.160803 48.536369 11.062485

C 22.114214 47.599586 11.751384

C 21.132935 1.308860 9.401107

O 21.376909 46.712181 12.575716

C 22.223734 45.713303 13.112559

H 20.546711 47.946236 10.359406

H 20.477772 0.087031 11.808589

H 22.846138 48.186787 12.336143

H 22.683815 47.046608 10.984745

H 21.814011 1.991404 8.872577

H 20.305180 1.894109 9.835276

H 23.029488 46.137199 13.736432

H 22.687237 45.094318 12.324409

H 21.605728 45.059605 13.743766

H 20.718813 0.600893 8.661359

O 12.022739 8.275595 36.642220

C 13.431341 8.428630 36.621197

C 13.940804 8.098114 37.999481

C 11.450205 8.436942 35.363186

O 14.957773 9.010943 38.383289

C 15.589470 8.498057 39.540096

H 13.709541 9.469558 36.378689

H 13.893377 7.783733 35.852112

H 14.294787 7.051319 38.047890

H 13.093980 8.175854 38.703049

H 10.359895 8.395383 35.495682

H 11.748451 7.632166 34.668419

H 16.087687 7.529491 39.351368

H 14.870256 8.363602 40.365406

H 16.346329 9.225093 39.857410

H 11.713248 9.408931 34.912884

O 27.545715 48.139362 31.086531

C 27.304672 47.859692 29.715088

C 27.383389 46.378242 29.482357

C 26.931131 0.454703 31.493809

O 27.113991 46.125954 28.113848

C 26.974648 44.740822 27.870852

H 28.040998 48.381870 29.080462

H 26.301880 48.194122 29.397285

H 26.642666 45.888336 30.143389

H 28.379826 46.000790 29.774736

H 27.166307 0.592092 32.559181

H 25.833490 0.417546 31.384289

H 26.172804 44.289051 28.477610

H 27.905075 44.184914 28.070333

H 26.714046 44.620056 26.810366

H 27.305162 1.326952 30.932030

O 18.286886 28.480055 11.664845

C 17.590162 29.668638 11.334329

C 17.989616 30.071028 9.942840

C 18.004284 28.026331 12.971748

O 17.217600 31.192627 9.552675

C 17.571583 31.639997 8.259949

H 17.821424 30.490040 12.036429

H 16.494474 29.520983 11.359263

H 17.823381 29.211981 9.266164

H 19.073082 30.294958 9.927683

H 18.551884 27.082699 13.103271

H 16.928644 27.833694 13.126249

H 17.433706 30.855757 7.495755

H 18.616911 31.993002 8.213990

H 16.907619 32.481930 8.017442

H 18.347511 28.735275 13.740833

O 23.033398 7.032740 21.280592

C 21.799833 7.111946 20.588268

C 20.742281 6.493453 21.456604

C 24.150599 7.173552 20.432791

O 19.523384 6.415224 20.739838

C 18.570951 5.681343 21.480562

H 21.830145 6.551635 19.638771

H 21.550480 8.159230 20.341848

H 20.624449 7.078699 22.385078

H 21.100666 5.491150 21.753384

H 25.031645 7.087989 21.081108

H 24.176512 8.146029 19.914526

H 18.333332 6.160982 22.446684

H 18.910269 4.650194 21.674667

H 17.655190 5.630006 20.877224

H 24.196068 6.376110 19.673485

O 20.218151 11.386643 43.027130

C 20.631783 10.043559 43.217323

C 21.991493 9.897369 42.582207

C 18.916624 11.620352 43.521435

O 22.118612 8.597310 42.032162

C 23.363424 8.462854 41.376511

H 19.943861 9.340969 42.718616

H 20.632273 9.766826 44.287586

H 22.807512 10.108586 43.297504

H 22.060432 10.662053 41.787209

H 18.709806 12.692081 43.388939

H 18.826662 11.377844 44.594143

H 24.212692 8.570419 42.073723

H 23.485655 9.194780 40.559025

H 23.392271 7.454196 40.943806

H 18.156830 11.043905 42.968460

O 7.082122 41.738804 33.951160

C 5.732192 41.921177 33.539482

C 5.210017 43.176743 34.199535

C 7.777866 40.858246 33.089180

O 3.803371 43.209988 34.379951

C 3.124250 43.487701 33.169853

H 5.115165 41.061153 33.856308

H 5.639296 41.986691 32.440861

H 5.580135 44.079792 33.678829

H 5.628050 43.212925 35.214550

H 8.799726 40.750679 33.477390

H 7.845338 41.266991 32.066341

H 3.285107 42.709816 32.403702

H 3.411251 44.465557 32.746441

H 2.053987 43.515572 33.406494

H 7.315341 39.856434 33.038330

O 47.113590 33.355160 48.422939

C 46.295128 34.424442 47.964325

C 46.980114 35.429680 47.076431

C 47.253914 32.324497 47.464153

O 46.209076 36.615818 47.109188

C 46.571861 37.520821 46.087330

H 45.970478 34.961773 48.867374

H 45.381317 34.054810 47.474911

H 47.099899 35.052711 46.049194

H 47.993172 35.620850 47.459263

H 47.815201 32.654522 46.573326

H 46.281437 31.913307 47.141949

H 46.301968 37.140926 45.086987

H 47.648968 37.748665 46.098087

H 46.008125 38.444897 46.273613

H 47.819111 31.516787 47.948681

O 2.292583 46.506832 25.988968

C 2.451485 47.906631 26.111198

C 1.892640 48.571087 24.883501

C 2.772222 45.817444 27.124258

O 1.686802 1.041415 25.203259

C 1.142136 1.762094 24.118330

H 3.511970 48.188744 26.251032

H 1.895574 48.295330 26.984425

H 0.941186 48.076778 24.615568

H 2.570294 48.446899 24.018099

H 2.562961 44.749626 26.961935

H 2.262759 46.132801 28.050776

H 0.166724 1.359218 23.795637

H 1.828102 1.777743 23.254881

H 0.992035 2.797156 24.455690

H 3.860087 45.946518 27.254801

O 16.829880 23.071045 27.540335

C 17.271872 22.013493 28.387648

C 17.687458 22.610476 29.703352

C 15.564534 22.907743 26.918909

O 17.751020 21.594482 30.690008

C 17.715328 22.183641 31.975889

H 18.094248 21.473719 27.892853

H 16.469051 21.290369 28.591530

H 16.917887 23.359512 29.954662

H 18.642824 23.161009 29.614368

H 15.270688 23.909069 26.577637

H 14.788605 22.546913 27.612700

H 16.786854 22.756176 32.147995

H 18.576330 22.849560 32.160217

H 17.746132 21.367132 32.702927

H 15.618804 22.229601 26.053017

O 39.925373 10.004445 24.131041

C 39.479469 10.526619 22.892586

C 40.105297 11.886329 22.701416

C 39.490711 8.674561 24.330034

O 40.387897 12.048653 21.320194

C 41.230320 13.159986 21.089909

H 38.376450 10.551555 22.843204

H 39.822208 9.890524 22.056519

H 41.032795 11.906375 23.300842

H 39.473602 12.711149 23.080824

H 39.934174 8.326933 25.274643

H 39.826607 7.999840 23.523792

H 42.199863 13.056822 21.607685

H 40.762905 14.110462 21.401356

H 41.423447 13.202034 20.009378

H 38.392582 8.603177 24.414619

O 8.588998 48.293373 31.202894

C 8.679450 0.630717 31.897173

C 9.701798 0.467415 32.983574

C 7.626299 48.362801 30.171747

O 9.719889 1.629106 33.794704

C 10.680632 1.513719 34.824387

H 7.712350 0.909405 32.354813

H 8.973784 1.457492 31.223431

H 10.687966 0.271846 32.522514

H 9.431910 48.462540 33.571262

H 7.625810 47.387878 29.669617

H 7.869296 0.243485 29.422709

H 11.708848 1.468251 34.425419

H 10.512441 0.625827 35.457546

H 10.591158 2.410413 35.452168

H 6.611285 48.557392 30.560934

O 37.186886 2.951169 31.899130

C 37.327209 2.246624 30.674366

C 36.112221 1.369976 30.491016

C 38.425831 3.432275 32.383167

O 36.134712 0.534398 29.335680

C 36.969311 48.297771 29.525383

H 38.229279 1.611996 30.680231

H 37.427929 2.946280 29.829496

H 35.222370 2.011450 30.393721

H 35.977764 0.775440 31.410690

H 38.235146 3.892845 33.362000

H 38.866352 4.193536 31.719204

H 36.627552 47.667057 30.364874

H 38.022953 48.573528 29.702864

H 36.928242 47.700790 28.604733

H 39.159222 2.618698 32.522999

O 36.961979 23.426985 20.047026

C 36.659821 24.620459 19.347858

C 37.792179 24.975908 18.431608

C 35.996838 23.185453 21.051773

O 37.494423 26.247122 17.887430

C 38.566151 26.816721 17.171152

H 36.533676 25.474615 20.037737

H 35.721569 24.529028 18.767990

H 37.911964 24.212202 17.642477

H 38.723587 24.993509 19.027122

H 36.308281 22.276537 21.582258

H 34.988663 23.029488 20.631294

H 38.832127 26.224144 16.280813

H 39.463333 26.941399 17.799423

H 38.230747 27.812668 16.848457

H 35.941586 24.004896 21.789564

O 10.313936 15.860825 22.843204

C 8.983563 16.292547 23.070557

C 9.014365 17.729507 23.501303

C 10.339360 14.613080 22.185596

O 7.683504 18.205723 23.601044

C 7.671769 19.606012 23.791725

H 8.481923 15.671121 23.834263

H 8.372403 16.232899 22.152349

H 9.588856 18.294218 22.745909

H 9.557565 17.829737 24.458136

H 11.390555 14.327057 22.074612

H 9.893458 14.668328 21.178406

H 8.173899 20.136499 22.963482

H 8.151407 19.899370 24.741224

H 6.621552 19.926260 23.816660

H 9.828430 13.824927 22.762043

O 43.810883 1.675067 25.638895

C 44.545250 1.231121 26.764408

C 45.963142 1.026749 26.318993

C 42.442863 1.848148 25.937630

O 46.634441 0.206817 27.256269

C 47.958946 48.880577 26.824547

H 44.161934 0.264999 27.137949

H 44.488049 1.947400 27.605364

H 46.473583 2.000693 26.196760

H 45.930382 0.547110 25.322559

H 41.946110 2.099457 24.992044

H 42.281025 2.673458 26.649998

H 48.514370 0.932875 26.704760

H 47.989262 48.330532 25.868690

H 48.466454 48.280170 27.591187

H 41.977890 0.931897 26.342461

O 33.721363 26.876373 18.972851

C 32.956192 28.064466 18.849152

C 32.946903 28.583220 17.442017

C 33.876842 26.573727 20.346739

O 32.050209 29.675974 17.457663

C 31.943621 30.389807 16.244633

H 33.340000 28.865822 19.504316

H 31.905485 27.893343 19.141531

H 32.613453 27.792133 16.747740

H 33.963871 28.888309 17.142303

H 34.311501 25.569956 20.423012

H 32.915123 26.563457 20.882603

H 31.620930 29.753714 15.403677

H 32.887253 30.891449 15.969855

H 31.176495 31.161337 16.408422

H 34.546188 27.292940 20.850334

O 36.920418 24.429775 2.800091

C 38.324623 24.497248 2.937479

C 38.703541 25.943010 3.088069

C 36.401176 23.121405 2.711106

O 40.118008 26.043240 3.088558

C 40.531155 27.394146 3.025975

H 38.839462 24.090948 2.047631

H 38.681049 23.932047 3.814616

H 38.267906 26.349796 4.020455

H 38.250793 26.504297 2.248580

H 35.319180 23.231413 2.554649

H 36.562523 22.545448 3.638113

H 40.238285 27.958368 3.928536

H 40.125343 27.913387 2.139060

H 41.627815 27.396591 2.954103

H 36.817745 22.557671 1.859393

O 35.659473 20.754015 46.660355

C 34.617569 21.710360 46.722935

C 34.393150 22.316139 45.370075

C 35.994877 20.299313 47.954544

O 33.535084 23.427963 45.545597

C 33.062778 23.915913 44.308121

H 34.866920 22.537624 47.411346

H 33.673447 21.259079 47.081810

H 33.956539 21.564659 44.692417

H 35.362206 22.627588 44.939327

H 36.870548 19.645126 47.850407

H 35.173481 19.721399 48.413162

H 32.420326 23.180077 43.796215

H 33.880756 24.217093 43.631447

H 32.451618 24.798918 44.521782

H 36.268188 21.130001 48.629269

O 29.375282 22.374813 38.885910

C 28.744566 23.508636 38.312397

C 27.258226 23.270039 38.302128

C 30.782415 22.504868 38.856087

O 26.683733 24.012232 37.239201

C 25.276110 24.048412 37.328674

H 29.067745 23.655804 37.269028

H 29.000275 24.440533 38.852665

H 26.807434 23.534550 39.273632

H 27.088568 22.189020 38.150562

H 31.198984 21.604261 39.327900

H 31.128578 23.389338 39.418354

H 24.938261 24.671307 38.175007

H 24.847321 23.040243 37.427437

H 24.899147 24.491381 36.398243

H 31.176004 22.572340 37.826893

O 25.838867 10.198549 36.985939

C 25.859890 8.945427 37.650391

C 27.246490 8.714653 38.174030

C 24.709442 10.352073 36.148403

O 27.365789 7.389658 38.657581

C 28.705450 7.107546 39.011566

H 25.621782 8.113760 36.964424

H 25.126499 8.920979 38.474724

H 27.469440 9.452934 38.964138

H 27.943701 8.892622 37.334053

H 24.825808 11.314282 35.628181

H 23.766788 10.368696 36.720936

H 29.043301 7.702083 39.876480

H 29.405107 7.287472 38.177452

H 28.754833 6.043150 39.277054

H 24.645393 9.557076 35.385185

O 42.512287 45.576889 29.028145

C 41.318329 46.251610 29.387505

C 40.192814 45.254684 29.411953

C 43.685715 46.242809 29.443243

O 39.054100 45.859493 30.000622

C 38.021976 44.910480 30.179569

H 41.103687 47.079853 28.689318

H 41.390198 46.688221 30.396164

H 40.536530 44.390263 30.009911

H 39.979153 44.879189 28.393517

H 44.524719 45.607204 29.130819

H 43.729721 46.367977 30.539907

H 38.354446 44.032856 30.762373

H 37.615673 44.555031 29.216381

H 37.220619 45.410164 30.740860

H 43.794746 47.229954 28.967028

O 29.094639 1.905841 23.164919

C 28.820839 2.676881 24.334925

C 28.207233 1.793877 25.386608

C 30.226995 2.420194 22.485798

O 27.443527 2.592785 26.269125

C 26.849480 1.819301 27.290495

H 28.107492 3.480190 24.085083

H 29.726334 3.165320 24.733889

H 28.979738 1.214986 25.923939

H 27.567228 1.068797 24.863457

H 30.388830 1.789477 21.603283

H 31.137869 2.386458 23.109671

H 27.599983 1.436959 28.002373

H 26.269125 0.969055 26.894461

H 26.158136 2.479843 27.829294

H 30.084229 3.464055 22.165062

O 48.697227 20.089073 33.077934

C 0.381853 18.794392 33.109226

C 0.785218 18.401295 31.706982

C 48.217590 20.427900 34.365284

O 0.704056 16.995138 31.478161

C 1.891173 16.342419 31.896685

H 48.535393 18.052198 33.457832

H 1.233076 18.746477 33.813282

H 1.773831 18.809549 31.427803

H 0.060627 18.859909 31.018568

H 47.789780 21.437536 34.309055

H 0.125654 20.435724 35.125076

H 2.111191 16.521366 32.963036

H 2.765866 16.652399 31.297260

H 1.743028 15.264333 31.750984

H 47.426502 19.737535 34.702152

O 39.068768 29.371861 28.023886

C 39.805584 28.530905 27.151150

C 39.135754 27.193197 27.015717

C 39.788471 30.574623 28.223368

O 39.994312 26.321928 26.296015

C 39.424709 25.029202 26.240767

H 40.833797 28.364180 27.522247

H 39.889191 28.994410 26.155691

H 38.171585 27.304184 26.492075

H 38.918179 26.805965 28.029263

H 39.198338 31.210230 28.896132

H 39.949329 31.126623 27.279737

H 38.430717 25.030180 25.760639

H 39.318123 24.578899 27.242090

H 40.101875 24.398485 25.649164

H 40.769260 30.394207 28.697140

O 32.500511 26.767340 9.493515

C 32.352367 25.371452 9.328746

C 32.652569 25.047781 7.892765

C 31.980291 27.217155 10.728058

O 32.898499 23.655804 7.756353

C 32.918053 23.293507 6.389800

H 31.332951 25.031645 9.588856

H 33.061798 24.832653 9.979020

H 33.535568 25.636450 7.583762

H 31.805256 25.385630 7.264981

H 32.197376 28.291819 10.804331

H 32.454552 26.714046 11.585149

H 33.723808 23.802481 5.833400

H 31.957802 23.516459 5.892560

H 33.084290 22.208576 6.337974

H 30.885094 27.083677 10.787219

O 7.206309 11.078130 42.608608

C 7.100701 10.323226 43.817726

C 8.411028 9.869989 44.423508

C 7.227822 12.485754 42.797821

O 8.109360 9.291099 45.683475

C 9.199181 9.282298 46.586525

H 6.499320 9.434843 43.572773

H 6.545280 10.879136 44.590721

H 9.083304 10.732458 44.533516

H 8.926358 9.137087 43.776657

H 7.994950 12.811380 43.518017

H 6.254367 12.871029 43.145939

H 9.480803 10.300246 46.905796

H 10.094896 8.793859 46.169960

H 8.867198 8.717586 47.469528

H 7.460553 12.940458 41.823879

O 26.467628 10.369674 46.444248

C 27.530069 11.080087 45.831623

C 28.819860 10.712901 46.510742

C 25.322069 10.361362 45.617470

O 29.858343 10.882071 45.561733

C 31.068930 10.309047 46.009594

H 27.343788 12.169907 45.835533

H 27.643499 10.782330 44.776516

H 28.731363 9.653395 46.814369

H 29.006142 11.300593 47.427971

H 24.534407 9.819630 46.159203

H 25.504440 9.838209 44.662594

H 30.947674 9.265675 46.345486

H 31.510920 10.894294 46.831966

H 31.757339 10.309047 45.154457

H 24.966619 11.382244 45.397942

O 1.299571 38.855110 42.757240

C 1.034572 37.457264 42.888275

C 0.761261 36.738049 41.590172

C 2.678837 39.128906 42.575851

O 0.633162 35.352917 41.862015

C 0.283089 34.652283 40.683697

H 0.144234 37.370720 43.530239

H 1.854015 36.937531 43.411427

H 1.588527 36.922863 40.881714

H 48.732433 37.140926 41.132046

H 2.779067 40.217751 42.474152

H 3.087091 38.650246 41.668400

H 1.034572 34.780869 39.885277

H 48.191189 34.963730 40.290112

H 0.227840 33.583977 40.934029

H 3.278262 38.807194 43.444187

O 0.306071 38.701584 1.153381

C 48.771057 37.563847 0.408255

C 48.468410 36.349350 1.241877

C 1.692671 38.644871 1.429625

O 47.174217 36.520966 1.806589

C 46.752762 35.448746 2.636300

H 0.590623 37.343342 48.488453

H 47.830360 37.861118 48.812614

H 0.347628 36.214409 2.016828

H 48.495300 35.437012 0.620450

H 1.924907 39.511738 2.062787

H 2.293074 38.701584 0.506040

H 46.961044 35.671207 3.691895

H 47.236313 34.497292 2.373745

H 45.668808 35.345093 2.501356

H 1.982113 37.730083 1.975269

O 7.287961 28.821815 2.753643

C 7.186264 28.049799 3.940271

C 5.805531 28.229235 4.522584

C 8.580687 28.664869 2.195776

O 5.520975 27.373123 5.630495

C 4.911282 26.161070 5.224684

H 7.948991 28.350977 4.681974

H 7.346632 26.977579 3.724654

H 5.075562 28.094292 3.710474

H 5.684277 29.259407 4.889769

H 8.639358 29.280430 1.288325

H 8.784081 27.617586 1.911219

H 5.532220 25.600269 4.504982

H 3.919247 26.343441 4.778782

H 4.775849 25.548443 6.125779

H 9.374705 29.004187 2.884675

O 22.857874 46.447182 27.321295

C 23.858219 47.441170 27.151150

C 23.214300 48.784744 27.247469

C 23.432364 45.206284 27.679193

O 22.394369 0.124677 26.113153

C 21.645332 1.312281 26.277435

H 24.361326 47.320896 26.176718

H 24.635614 47.354633 27.930500

H 23.972630 0.689386 27.342808

H 22.619764 48.789635 28.179363

H 24.065035 45.298691 28.576862

H 24.035212 44.769180 26.865128

H 22.285828 2.199684 26.414825

H 20.949097 1.247253 27.129637

H 21.050306 1.450649 25.365585

H 22.609497 44.521294 27.919256

O 41.085598 21.311882 25.232597

C 39.759624 21.340729 24.733889

C 38.788124 21.515276 25.866245

C 42.009674 21.132935 24.180424

O 37.529625 21.856548 25.311804

C 36.578659 22.092701 26.333174

H 39.513695 20.414700 24.184824

H 39.616859 22.183151 24.032768

H 39.162151 22.308805 26.536568

H 38.727009 20.588268 26.464693

H 43.012951 21.194050 24.622414

H 41.921665 21.915709 23.408405

H 36.871529 22.931211 26.989315

H 36.412422 21.201874 26.963400

H 35.629162 22.352810 25.844246

H 41.895752 20.147745 23.696875

O 32.545494 36.579147 37.649899

C 31.179426 36.267212 37.867962

C 30.433323 36.633907 36.609463

C 33.328754 36.368420 38.805237

O 29.180201 37.203506 36.938999

C 28.449741 37.514465 35.767525

H 30.762861 36.859791 38.700119

H 31.055729 35.204773 38.143227

H 30.311581 35.763126 35.940609

H 31.048883 37.379032 36.077507

H 34.358437 36.662754 38.554905

H 33.330711 35.309891 39.115215

H 28.218479 36.614353 35.170059

H 28.978762 38.236614 35.122631

H 27.502199 37.968193 36.089241

H 32.984550 36.984959 39.652550

O 24.601391 42.426727 28.941603

C 24.073835 42.978237 30.144367

C 24.409729 42.232624 31.406290

C 23.987785 41.202938 28.577353

O 24.187269 43.085800 32.523979

C 24.636105 42.398857 33.674427

H 22.983526 43.137138 30.072493

H 24.537342 43.971737 30.239706

H 25.470215 41.924107 31.343708

H 23.809814 41.308548 31.494297

H 24.342747 40.952610 27.567228

H 22.887697 41.281170 28.553885

H 24.554453 43.093624 34.513916

H 25.694145 42.088390 33.595222

H 24.026409 41.501675 33.891022

H 24.273319 40.377628 29.253540

O 26.595726 34.346214 10.723658

C 27.397570 34.660595 9.597168

C 26.498430 35.141701 8.491212

C 27.390724 34.095394 11.866282

O 27.269958 35.880959 7.559315

C 26.476919 36.352287 6.490520

H 28.000908 33.792259 9.275942

H 28.110428 35.468792 9.825007

H 25.716145 35.775837 8.948359

H 25.988968 34.286564 8.010596

H 26.712582 33.789326 12.674479

H 27.933434 34.998444 12.195820

H 25.690231 37.046074 6.832769

H 26.002659 35.527462 5.931186

H 27.148706 36.890594 5.806998

H 28.121670 33.284752 11.700536

O 0.410211 7.569583 0.211708

C 48.684517 8.505391 48.219055

C 0.727036 9.617214 47.823513

C 48.872753 6.243122 48.828262

O 0.005378 10.513418 46.994293

C 0.841445 11.521099 46.457939

H 47.780975 8.928314 48.692337

H 48.359379 8.021841 47.285202

H 1.591949 9.181579 47.288139

H 1.117200 10.128143 48.722164

H 0.470349 5.572312 0.655651

H 0.265488 5.923851 47.810310

H 1.655021 11.102088 45.840420

H 1.294192 12.151327 47.242176

H 0.216106 12.159638 45.817444

H 47.779510 6.128223 0.034714

O 12.710662 1.775787 47.904186

C 13.208390 0.834111 48.842438

C 13.640602 1.502965 1.220364

C 12.350322 1.057551 46.738583

O 14.219982 0.493817 2.028562

C 14.860967 1.018437 3.170698

H 14.085526 0.291401 48.441032

H 12.448106 0.069917 0.198994

H 12.777644 1.978203 1.721027

H 14.365194 2.300406 0.978345

H 11.879972 1.752807 46.032570

H 11.620841 0.256687 46.953224

H 14.152998 1.525944 3.846397

H 15.665743 1.725916 2.905699

H 15.312737 0.172103 3.706563

H 13.227458 0.613605 46.237919

O 43.066242 34.310036 47.132168

C 42.827160 33.598152 48.329552

C 42.745995 34.620502 0.534395

C 42.881428 33.492058 45.999813

O 42.932766 34.005920 1.800236

C 42.644299 34.947105 2.818179

H 43.652470 32.893120 48.531479

H 41.892818 33.009972 48.276260

H 41.767651 35.133389 0.465946

H 43.521435 35.387630 0.345669

H 43.070156 34.098328 45.104099

H 41.843925 33.125847 45.934784

H 41.590660 35.276154 2.784933

H 43.288219 35.842823 2.755109

H 42.816891 34.461113 3.788212

H 43.578152 32.637409 45.986122

O 35.520618 39.431553 29.851498

C 36.826057 39.001297 30.234329

C 37.002071 37.500778 30.136543

C 34.583344 39.251629 30.899271

O 37.944725 37.101814 31.113420

C 37.976994 35.698586 31.266457

H 37.068565 39.315189 31.264502

H 37.526203 39.531784 29.572809

H 37.315472 37.187374 29.124952

H 36.024704 37.024075 30.321848

H 33.579086 39.340126 30.464615

H 34.707531 40.025600 31.670311

H 38.241016 35.184235 30.326736

H 37.015270 35.298157 31.630219

H 38.746075 35.473194 32.018917

H 34.660103 38.264484 31.379398

O 16.033417 32.649147 2.395258

C 14.675174 32.762577 2.776622

C 13.823462 32.203732 1.675556

C 16.904686 33.082821 3.418585

O 12.455930 32.350899 2.016828

C 11.633553 31.854637 0.977856

H 14.396485 33.816704 2.944813

H 14.464445 32.212044 3.710964

H 14.092372 31.141291 1.522522

H 14.064991 32.742039 0.741215

H 17.928501 32.896542 3.065579

H 16.758986 32.525444 4.359282

H 11.819835 30.784861 0.778373

H 11.766052 32.418369 0.037159

H 10.588714 31.966112 1.298593

H 16.792233 34.158955 3.628335

O 42.965034 43.872974 34.182423

C 41.903084 43.634377 33.271061

C 40.917892 44.748158 33.362980

C 43.898888 42.820805 34.069969

O 41.559856 45.942120 32.949348

C 40.666100 47.032917 32.898987

H 41.420025 42.661903 33.485210

H 42.282494 43.588909 32.232090

H 40.567333 44.831276 34.405373

H 40.034401 44.536449 32.731773

H 44.734467 43.041309 34.748112

H 44.297852 42.730839 33.043709

H 40.320911 47.341919 33.898354

H 39.787003 46.821209 32.266315

H 41.214676 47.869965 32.449661

H 43.455921 41.850769 34.360882

O 33.605976 19.446133 37.872852

C 34.543743 20.360428 37.327694

C 34.453777 20.364828 35.825710

C 33.918892 19.167444 39.221313

O 35.743572 20.654274 35.319180

C 35.724014 20.745214 33.911068

H 35.570000 20.046047 37.574604

H 34.401951 21.372509 37.749641

H 33.700829 21.085508 35.457058

H 34.142330 19.358126 35.499596

H 34.879635 18.635979 39.325943

H 33.121449 18.524504 39.615879

H 35.129963 21.605728 33.562462

H 35.326515 19.832386 33.436321

H 36.762497 20.888960 33.584953

H 33.975605 20.086140 39.824162

O 25.452124 17.670345 38.482056

C 24.300209 17.038652 37.941792

C 24.270876 17.259647 36.457405

C 25.363630 17.731462 39.894569

O 23.223103 16.493010 35.891712

C 23.071045 16.751650 34.510494

H 23.368315 17.448862 38.372047

H 24.303633 15.955188 38.154472

H 25.251665 16.977535 36.032036

H 24.127131 18.338711 36.274055

H 26.165470 18.391516 40.250996

H 25.483418 16.743340 40.370293

H 24.009787 16.610350 33.947735

H 22.700928 17.773510 34.319321

H 22.329342 16.037817 34.124241

H 24.404352 18.154385 40.237305

O 32.843250 35.653606 15.543999

C 34.007389 34.885502 15.271176

C 34.838074 35.400341 14.126107

C 33.131229 36.933620 16.072041

O 35.903450 34.499249 13.881155

C 36.539547 34.869858 12.675458

H 33.641178 33.882710 15.000311

H 34.634190 34.754955 16.169827

H 35.230686 36.409977 14.346614

H 34.177532 35.493237 13.245548

H 33.602062 37.597588 15.328871

H 33.783947 36.886684 16.957001

H 36.979580 35.879490 12.734130

H 35.843800 34.850788 11.820812

H 37.337967 34.142330 12.479398

H 32.169018 37.371700 16.367353

O 40.608406 5.549821 16.991714

C 41.042080 6.787787 16.458294

C 40.571247 7.857562 17.403391

C 41.159916 4.436533 16.317482

O 41.024967 9.140019 16.991714

C 40.767303 10.079250 18.020418

H 40.632851 6.954512 15.445724

H 42.142662 6.831791 16.382021

H 40.956520 7.592563 18.405695

H 39.467243 7.821870 17.467930

H 40.688099 3.535438 16.736982

H 42.251690 4.360260 16.463673

H 41.303169 9.832831 18.953783

H 39.692642 10.152590 18.257549

H 41.116402 11.061018 17.672302

H 40.953098 4.458535 15.236464

O 45.327049 12.008560 13.206923

C 44.323277 11.873128 14.196024

C 43.065754 12.449574 13.614201

C 46.582615 11.525010 13.634734

O 41.998917 12.406548 14.552942

C 41.003944 13.330622 14.149577

H 44.582897 12.427083 15.117653

H 44.164375 10.818509 14.478624

H 42.802223 11.900018 12.692081

H 43.305332 13.486590 13.311065

H 47.283737 11.683912 12.803069

H 46.556213 10.446436 13.864531

H 40.531639 13.019175 13.205457

H 41.403400 14.352482 14.019032

H 40.235840 13.375113 14.931862

H 46.962025 12.064298 14.520184

O 40.634315 17.835604 11.005769

C 40.960430 19.206070 11.124578

C 42.397392 19.367905 11.540656

C 39.235981 17.669859 10.876692

O 42.808578 20.659164 11.125068

C 44.087124 21.006302 11.613506

H 40.298912 19.710644 11.851126

H 40.831356 19.713577 10.152102

H 43.009041 18.582685 11.064441

H 42.516689 19.231005 12.627543

H 39.051167 16.598616 10.730991

H 38.824306 18.214523 10.010312

H 44.848389 20.256777 11.352419

H 44.092014 21.137825 12.708706

H 44.362881 21.960201 11.142669

H 38.691807 17.993040 11.780720

O 22.837337 19.461779 24.767138

C 21.757296 19.823586 25.608580

C 20.851801 20.712946 24.805273

C 23.892933 18.868221 25.493195

O 19.665663 21.015102 25.526442

C 19.089705 22.212976 25.032135

H 22.099545 20.374607 26.504787

H 21.210186 18.929825 25.959633

H 20.606848 20.217173 23.849907

H 21.428736 21.622841 24.556410

H 24.657618 18.567530 24.764692

H 23.566818 17.975925 26.052530

H 18.786079 22.131815 23.974094

H 19.770782 23.075933 25.132854

H 18.194477 22.427128 25.628628

H 24.352526 19.569832 26.209963

O 23.872889 26.672001 24.410707

C 24.416086 26.718447 23.092558

C 24.684017 25.333803 22.565495

C 23.099403 27.842005 24.628769

O 24.861012 25.425234 21.158358

C 25.619827 24.375505 20.591202

H 25.335760 27.334497 23.074957

H 23.713985 27.186352 22.382633

H 23.818617 24.688908 22.795778

H 25.562622 24.898657 23.061754

H 22.821203 27.887962 25.689255

H 22.167995 27.820980 24.039612

H 25.064405 23.426006 20.567734

H 26.581549 24.217093 21.110443

H 25.839846 24.685486 19.562008

H 23.648958 28.759722 24.366217

O 31.545145 26.907663 22.884764

C 30.987768 28.106514 22.369434

C 30.093031 27.776487 21.213118

C 32.286362 27.150171 24.064547

O 28.843819 27.312984 21.699602

C 27.964237 26.987358 20.644007

H 31.792543 28.801769 22.065321

H 30.381985 28.629669 23.132650

H 30.580978 27.007406 20.592180

H 29.949772 28.659492 20.569201

H 32.706348 26.188450 24.392618

H 31.657600 27.549625 24.879101

H 26.990294 26.777609 21.103109

H 28.298664 26.096531 20.085651

H 27.831736 27.820004 19.933105

H 33.116070 27.852272 23.896357

O 33.095539 45.104584 17.094389

C 32.049721 46.063862 17.109547

C 32.632034 47.418194 16.792233

C 32.536201 43.812347 16.980469

O 31.720671 48.510948 16.866549

C 30.676809 48.379425 15.918518

H 31.542212 46.077065 18.090824

H 31.280146 45.821354 16.354153

H 33.117046 47.361965 15.801664

H 33.435341 47.638699 17.511444

H 33.362980 43.098022 16.900774

H 31.921621 43.710163 16.068130

H 29.947817 47.603497 16.211876

H 31.043995 48.146206 14.905458

H 30.164412 0.452261 15.875981

H 31.913795 43.553215 17.854183

O 47.531136 45.288910 23.865065

C 46.307350 45.023422 24.547607

C 45.554401 46.230587 25.065870

C 48.615086 45.351494 24.776915

O 44.207890 45.804241 25.161213

C 43.304840 46.755207 25.682409

H 46.453049 44.289543 25.359230

H 45.643871 44.540363 23.812750

H 45.654629 47.065189 24.349104

H 45.945053 46.586525 26.036882

H 0.617515 45.618450 24.199492

H 48.796482 44.380974 25.273178

H 43.270126 47.681236 25.084938

H 43.535126 47.019226 26.727249

H 42.310360 46.283882 25.658451

H 48.465477 46.117645 25.556757

O 17.624876 13.196656 22.369434

C 18.422318 13.845952 23.344845

C 19.274519 14.879545 22.654968

C 16.735027 12.261335 22.940990

O 19.564453 15.905316 23.588331

C 20.374607 16.914465 23.024597

H 19.042767 13.128695 23.912491

H 17.790623 14.373506 24.079214

H 18.695139 15.275577 21.802277

H 20.198103 14.436089 22.240356

H 16.169338 11.809078 22.114214

H 16.018749 12.740486 23.627935

H 19.920883 17.370144 22.127903

H 21.375933 16.536522 22.758619

H 20.492928 17.694304 23.789280

H 17.263557 11.459005 23.484678

O 23.355112 32.376324 4.989510

C 23.988764 33.412849 4.261497

C 25.459948 33.131229 4.144643

C 21.970957 32.626656 5.129832

O 26.027592 34.197090 3.403428

C 27.426414 34.068012 3.250393

H 23.863110 34.392662 4.759714

H 23.568773 33.510147 3.242571

H 25.613960 32.157772 3.643003

H 25.903404 33.051044 5.155257

H 21.563190 31.829700 5.766906

H 21.444382 32.606606 4.159799

H 27.706083 33.130249 2.739464

H 27.958858 34.118374 4.216515

H 27.759375 34.909946 2.627010

H 21.770985 33.595707 5.619249

O 5.431990 30.634760 45.160812

C 4.023878 30.734015 45.045425

C 3.518326 30.773619 46.461849

C 6.120401 30.290068 43.979073

O 2.259825 31.410202 46.586525

C 1.913664 31.418512 47.959435

H 3.593132 29.887678 44.483646

H 3.748122 31.661020 44.518360

H 4.273231 31.333927 47.035851

H 3.489968 29.749804 46.873035

H 7.178441 30.188858 44.259716

H 6.041194 31.070885 43.208035

H 2.699372 31.872240 48.589664

H 1.708803 30.400078 48.335423

H 1.007681 32.025272 48.073357

H 5.782063 29.328346 43.555660

O 31.106089 25.838867 42.446774

C 30.502752 26.974157 43.045219

C 30.094496 26.593771 44.440132

C 31.611639 26.185518 41.173603

O 29.468180 27.605854 45.217037

C 30.368784 28.607666 45.656094

H 29.615345 27.304184 42.475616

H 31.205339 27.821959 43.073578

H 30.965765 26.177692 44.963776

H 29.369905 25.772371 44.351147

H 31.930908 25.254597 40.692497

H 32.479000 26.864637 41.248409

H 30.860645 29.127398 44.819050

H 31.146181 28.212122 46.330326

H 29.783539 29.354259 46.209560

H 30.848911 26.661243 40.532619

O 10.825355 18.105003 44.934441

C 11.946466 17.390190 45.425323

C 11.456561 16.422113 46.460869

C 11.156848 18.899023 43.810390

O 11.371977 17.068966 47.723282

C 10.750548 16.214808 48.666912

H 12.685726 18.075178 45.875134

H 12.463264 16.874861 44.601479

H 12.099502 15.527864 46.537144

H 10.467460 16.062752 46.124977

H 10.272377 19.505783 43.567883

H 11.414513 18.284441 42.930325

H 11.249255 15.234507 48.740742

H 9.685663 16.050528 48.433208

H 10.802375 16.702271 0.756373

H 11.997315 19.583033 44.013786

O 15.086851 36.411934 48.861507

C 14.674685 36.332729 1.321572

C 14.863411 37.686569 1.943000

C 14.970487 35.166145 48.202435

O 14.442933 37.649899 3.296352

C 14.837497 38.817951 3.987697

H 15.275577 35.596401 1.884817

H 13.616156 36.027637 1.408113

H 14.289409 38.435608 1.367043

H 15.931230 37.954502 1.850104

H 15.285356 35.323090 47.161507

H 13.931515 34.790649 48.195099

H 14.444400 39.736156 3.518326

H 15.935630 38.899113 4.062014

H 14.430710 38.748032 5.005645

H 15.620272 34.393642 48.650291

O 20.908516 5.938030 31.337841

C 21.814011 5.789885 32.417393

C 21.071331 5.252064 33.606464

C 21.585682 6.172716 30.119919

O 21.988560 4.993422 34.659126

C 21.308949 4.380306 35.738682

H 22.305874 6.748184 32.666260

H 22.605097 5.059916 32.175373

H 20.564312 4.324079 33.283283

H 20.286600 5.966877 33.917912

H 20.834688 6.163426 29.319544

H 22.317608 5.381142 29.886213

H 20.897272 3.394138 35.456570

H 20.485106 5.009067 36.121998

H 22.035986 4.232650 36.550301

H 22.095634 7.149105 30.112587

O 17.950014 11.663378 36.762985

C 18.445787 12.946324 37.093014

C 19.942884 12.901343 37.181507

C 16.550701 11.697602 36.568882

O 20.372652 14.179889 37.601986

C 21.777830 14.285008 37.670437

H 18.051222 13.291507 38.066467

H 18.161718 13.698785 36.335663

H 20.363852 12.621187 36.199741

H 20.253353 12.121503 37.894852

H 16.219696 10.659609 36.422199

H 16.273970 12.287739 35.677078

H 22.260891 14.132464 36.691113

H 22.215910 13.575575 38.393562

H 21.996382 15.309314 38.002419

H 16.022659 12.109768 37.443573

O 40.796642 34.664017 34.943195

C 40.604980 33.574684 35.826199

C 39.423241 33.875374 36.701870

C 41.915794 34.466000 34.105663

O 39.276073 32.807068 37.617630

C 38.166695 32.988461 38.470810

H 41.488964 33.409428 36.470116

H 40.419678 32.630566 35.279579

H 38.525082 34.000053 36.069195

H 39.595345 34.836117 37.222088

H 41.997936 35.356827 33.466633

H 41.796497 33.580551 33.458321

H 37.217201 33.050068 37.910011

H 38.264973 33.890533 39.100060

H 38.124161 32.107414 39.128418

H 42.852585 34.362839 34.680641

O 44.091526 38.724075 6.983358

C 43.043266 37.944725 7.537313

C 42.781200 38.374004 8.955694

C 44.434753 38.241501 5.700901

O 42.275158 37.250446 9.656817

C 42.067364 37.532558 11.025327

H 43.337112 36.880817 7.578873

H 42.124569 38.007309 6.923709

H 42.091320 39.236473 9.010943

H 43.741455 38.702072 9.386929

H 45.246864 38.875641 5.320514

H 43.584511 38.298218 4.997822

H 41.286057 38.297241 11.179338

H 42.989483 37.865028 11.534300

H 41.735870 36.594795 11.491275

H 44.798515 37.200089 5.735614

O 16.312105 15.363585 8.304932

C 17.420994 15.849579 7.570561

C 17.199020 17.329075 7.375479

C 16.273479 13.962317 8.459433

O 17.622433 17.702616 6.078842

C 17.498734 19.098505 5.875937

H 17.481621 15.375808 6.577548

H 18.372936 15.630051 8.086869

H 17.709949 17.932901 8.143096

H 16.115555 17.514868 7.478154

H 15.414922 13.745722 9.111174

H 17.182396 13.569708 8.943471

H 18.303997 19.660772 6.377577

H 16.530167 19.495026 6.222587

H 17.577938 19.274031 4.794917

H 16.119467 13.442098 7.500155

O 0.144234 7.595496 17.167728

C 0.464482 6.214275 17.135460

C 0.812109 5.850512 15.721480

C 48.682560 7.992506 18.475122

O 1.097643 4.462446 15.625649

C 1.134313 4.086460 14.263986

H 48.502636 5.595781 17.465485

H 1.316683 5.979100 17.798935

H 1.678001 6.453849 15.391943

H 48.848797 6.138980 15.083428

H 48.535393 9.081348 18.456543

H 0.580357 7.758799 19.206558

H 1.875039 4.674641 13.694873

H 0.156457 4.205759 13.770658

H 1.408113 3.024020 14.216071

H 47.747242 7.511890 18.809059

O 41.875706 26.398689 17.241556

C 42.800758 27.314939 17.800892

C 42.093281 28.036108 18.910269

C 42.394947 25.651119 16.163471

O 42.933254 29.030100 19.469112

C 42.216976 29.700420 20.488527

H 43.154251 28.052244 17.057232

H 43.693539 26.805479 18.205235

H 41.777431 27.301739 19.671040

H 41.170181 28.482990 18.499567

H 41.588703 24.981287 15.837844

H 43.258396 25.030670 16.457806

H 42.044872 29.040855 21.355885

H 41.241077 30.080318 20.137478

H 42.808578 30.564846 20.816599

H 42.684391 26.289658 15.310780

O 11.277123 35.430656 4.476624

C 11.086931 35.753349 3.107626

C 9.632859 36.123955 2.952147

C 12.445173 34.677708 4.726467

O 9.512094 37.278805 2.138082

C 8.199322 37.795113 2.203110

H 11.384199 34.923149 2.443173

H 11.710803 36.621197 2.836760

H 9.243672 36.312195 3.965695

H 9.030499 35.290825 2.550248

H 12.491132 34.514893 5.811887

H 13.358001 35.206238 4.406219

H 7.964148 38.172565 3.209812

H 7.437084 37.047050 1.929799

H 8.141629 38.631668 1.494164

H 12.409481 33.689583 4.237539

O 5.192415 11.322105 35.555332

C 6.128712 11.499098 34.506580

C 7.488910 11.549458 35.138279

C 3.851286 11.430159 35.125565

O 8.482901 11.439938 34.137440

C 9.770737 11.601772 34.694820

H 5.947809 12.426105 33.933559

H 6.093509 10.663031 33.786880

H 7.558826 10.714857 35.858959

H 7.586206 12.490154 35.713257

H 3.220569 11.344108 36.022259

H 3.576020 10.623427 34.426888

H 9.964353 10.878648 35.504486

H 9.927195 12.620699 35.088898

H 10.498751 11.429669 33.890533

H 3.640558 12.403614 34.650326

O 20.895803 0.771528 3.174609

C 22.178263 0.365229 3.656693

C 23.142918 1.493186 3.973518

C 20.154591 1.541101 4.111395

O 24.291899 1.345530 3.159942

C 25.394920 2.087723 3.637136

H 22.615852 48.669357 2.837249

H 22.064342 48.554462 4.500093

H 23.415741 1.514699 5.041825

H 22.659857 2.454419 3.750078

H 20.377052 2.613809 3.992586

H 20.355539 1.256056 5.154768

H 25.721525 1.745473 4.635037

H 25.181746 3.168253 3.682117

H 26.216318 1.917087 2.928679

H 19.090683 1.368021 3.907024

O 22.168974 36.527809 11.497142

C 21.469807 36.960510 10.345716

C 22.423704 36.888149 9.184512

C 21.422869 36.638798 12.691592

O 21.696669 37.025047 7.975883

C 22.554737 36.916508 6.856726

H 21.090399 37.993126 10.460125

H 20.600981 36.310726 10.135966

H 22.947836 35.915672 9.234383

H 23.198166 37.672390 9.286210

H 22.123013 36.452515 13.518371

H 20.610760 35.897583 12.741953

H 23.045622 35.927898 6.804411

H 23.337023 37.695370 6.856726

H 21.935755 37.046562 5.957588

H 20.995058 37.644032 12.835337

O 7.433661 48.142784 21.422379

C 6.273435 0.004400 21.111912

C 5.972255 48.784260 19.646105

C 7.741197 48.286530 22.793335

O 6.484652 1.036527 18.979206

C 5.839756 1.212052 17.731462

H 6.418158 1.075642 21.339750

H 5.420256 48.556908 21.725025

H 4.887813 48.691853 19.474491

H 6.434293 47.866539 19.249096

H 8.566997 47.600071 23.007975

H 6.891440 48.024464 23.447031

H 4.744557 1.306904 17.840004

H 6.049017 0.383808 17.033764

H 6.220142 2.145905 17.300718

H 8.069757 0.417544 23.039753

O 22.331297 11.109911 5.499951

C 23.677805 10.700190 5.651519

C 24.592100 11.883394 5.530753

C 21.470295 10.112009 4.989510

O 25.913183 11.376376 5.480883

C 26.848503 12.417304 5.287756

H 23.978495 9.986843 4.866300

H 23.827417 10.213706 6.630352

H 24.448845 12.578161 6.378554

H 24.333456 12.434417 4.607169

H 20.475327 10.568178 4.902970

H 21.388645 9.238784 5.655430

H 26.802544 13.178076 6.087642

H 26.711115 12.923344 4.315767

H 27.846407 11.960156 5.304869

H 21.779299 9.779049 3.985252

O 6.984336 6.448471 19.225626

C 5.702367 6.430381 18.608601

C 5.361584 7.727996 17.938768

C 7.209243 5.130810 19.700375

O 4.131930 7.526068 17.267958

C 3.735899 8.629090 16.483719

H 4.907859 6.204008 19.344925

H 5.651519 5.648585 17.828760

H 6.166849 7.989572 17.233734

H 5.280911 8.540594 18.679983

H 8.147007 5.125432 20.270956

H 7.301651 4.402308 18.873110

H 4.442400 8.827595 15.661342

H 3.620512 9.550719 17.076788

H 2.759510 8.371914 16.050528

H 6.397623 4.782205 20.364340

O 47.537979 21.729916 15.577246

C 46.786011 20.537420 15.433011

C 46.813877 20.107164 13.989697

C 47.444595 22.187063 16.913486

O 45.830643 19.136642 13.642070

C 46.152847 17.848804 14.140776

H 45.733837 20.701212 15.720990

H 47.188396 19.739979 16.084753

H 47.831825 19.784472 13.707586

H 46.584084 20.995058 13.381470

H 48.136917 23.032907 17.021540

H 47.730618 21.410645 17.643944

H 46.168980 17.816046 15.244775

H 47.126301 17.489443 13.764790

H 45.369095 17.160883 13.791192

H 46.425671 22.534203 17.161373

O 41.404865 42.008205 12.868096

C 42.034119 41.148178 11.937666

C 43.090202 41.908463 11.192051

C 40.435322 41.309036 13.621534

O 43.768833 40.982433 10.364296

C 44.683128 41.589191 9.478847

H 41.307571 40.737480 11.212097

H 42.511803 40.286201 12.435394

H 43.774700 42.383701 11.916153

H 42.613987 42.718616 10.609737

H 39.898968 42.052208 14.225849

H 40.895893 40.568802 14.298699

H 45.524086 42.061497 10.012267

H 44.201046 42.338230 8.827595

H 45.079159 40.784416 8.843729

H 39.699486 40.791752 12.983482

O 43.448586 37.752087 46.238899

C 42.418415 38.711849 46.325928

C 42.826180 39.609035 47.451439

C 43.253994 36.808453 45.212151

O 41.941708 40.706188 47.560471

C 42.217464 41.421001 48.744656

H 41.437626 38.247860 46.542522

H 42.316231 39.290745 45.392075

H 43.861729 39.943459 47.265156

H 42.851116 38.996895 48.372578

H 44.022591 36.041817 45.364204

H 43.391869 37.248001 44.210827

H 43.220257 41.879616 48.730476

H 42.137772 40.771706 0.738771

H 41.468430 42.218933 48.836082

H 42.263912 36.324417 45.260555

O 25.920029 36.914555 42.710304

C 24.970041 37.963303 42.756264

C 24.499203 38.209236 41.351086

C 26.331219 36.488209 43.993252

O 23.793680 39.439377 41.280678

C 23.311110 39.629570 39.966442

H 25.424255 38.891777 43.144474

H 24.112461 37.713463 43.408005

H 23.872889 37.358498 41.022038

H 25.388075 38.223904 40.692989

H 27.073410 35.690765 43.839241

H 25.492704 36.075062 44.581921

H 22.550337 38.878578 39.694595

H 24.120285 39.583122 39.219360

H 22.849072 40.624050 39.923904

H 26.805479 37.297871 44.572632

O 43.422184 25.915140 31.853168

C 44.304211 26.917440 32.317162

C 44.782379 27.676258 31.109510

C 42.934723 25.094229 32.890675

O 46.058975 28.221903 31.387709

C 46.427624 29.232517 30.475859

H 45.179390 26.480339 32.826138

H 43.810390 27.586296 33.046154

H 44.046059 28.450230 30.827887

H 44.848389 26.970245 30.263174

H 42.139236 24.471823 32.460419

H 42.512287 25.679476 33.724300

H 45.731392 30.089605 30.503241

H 46.490696 28.856531 29.441776

H 47.420147 29.584055 30.789751

H 43.713585 24.421953 33.279861

O 27.604385 9.976576 21.305527

C 27.095900 10.540798 20.107653

C 28.219948 11.041950 19.240784

C 26.569813 9.678819 22.226177

O 27.639099 11.462916 18.020418

C 28.519659 12.169907 17.171640

H 26.422157 11.393489 20.311047

H 26.511633 9.797627 19.533161

H 28.956270 10.237174 19.071125

H 28.744566 11.867749 19.756113

H 27.033806 9.204558 23.104292

H 25.827133 8.981118 21.807655

H 29.391905 11.567547 16.867039

H 28.878532 13.105714 17.632210

H 27.946636 12.428550 16.269567

H 26.036882 10.583336 22.569895

O 39.531784 30.124321 31.409222

C 38.194565 30.330648 31.841925

C 37.929077 31.805256 31.952911

C 39.907280 28.771944 31.577415

O 38.072334 32.553314 30.746725

C 36.933620 32.481930 29.907724

H 38.026867 29.880346 32.839336

H 37.480732 29.848566 31.148136

H 38.683983 32.220844 32.639854

H 36.944866 31.988115 32.420326

H 40.928650 28.662424 31.188717

H 39.248203 28.079624 31.023949

H 37.139950 33.125362 29.039877

H 36.020306 32.852051 30.403988

H 36.745872 31.457138 29.544941

H 39.912170 28.484455 32.643764

O 16.326284 37.203999 13.091537

C 16.342419 35.802242 12.894987

C 17.561316 35.434078 12.099502

C 15.217884 37.638168 13.849863

O 17.679148 34.023521 12.110258

C 18.824217 33.578594 11.414513

H 15.440347 35.451191 12.363033

H 16.390333 35.260021 13.854752

H 18.435030 35.920071 12.566427

H 17.479176 35.828156 11.069818

H 14.257629 37.349209 13.391249

H 15.264821 38.733852 13.879687

H 19.760513 33.950180 11.865304

H 18.800749 33.869019 10.350606

H 18.830084 32.481930 11.473185

H 15.251131 37.263645 14.886879

O 37.858185 45.045425 23.945248

C 37.759911 44.686550 25.314735

C 37.049007 43.365959 25.407143

C 38.785679 46.094173 23.759945

O 36.735115 43.075047 26.758053

C 36.302414 41.734406 26.889572

H 37.208397 45.451237 25.884336

H 38.752922 44.601479 25.789484

H 37.707108 42.595409 24.966131

H 36.133247 43.409470 24.790606

H 38.787636 46.336193 22.687725

H 39.808517 45.794464 24.050858

H 37.052433 41.019104 26.510654

H 35.348518 41.548611 26.365442

H 36.161606 41.534924 27.961792

H 38.508457 46.999668 24.327101

O 7.536336 32.742039 20.032846

C 7.946547 33.328754 21.253700

C 9.357593 32.860363 21.485453

C 6.283214 33.186478 19.557119

O 10.040136 33.727718 22.371878

C 11.417935 33.407471 22.357210

H 7.273782 33.055443 22.084877

H 7.955836 34.430309 21.184761

H 9.845055 32.857918 20.493416

H 9.373727 31.819923 21.858503

H 6.138491 32.721996 18.570463

H 6.243610 34.281677 19.426086

H 11.854059 33.499390 21.346596

H 11.611551 32.387566 22.734663

H 11.932776 34.122284 23.010908

H 5.452036 32.875027 20.212282

O 4.939151 21.454649 20.787262

C 5.770817 22.165062 19.883722

C 6.823479 21.238544 19.348837

C 4.055658 22.347431 21.436558

O 7.545626 21.919130 18.338223

C 8.688251 21.207741 17.911388

H 6.260234 23.028019 20.371185

H 5.184593 22.563049 19.034945

H 6.327217 20.335981 18.948404

H 7.477665 20.917807 20.180504

H 4.576855 22.942457 22.202709

H 3.276795 21.747028 21.922554

H 8.434985 20.228418 17.473797

H 9.413820 21.060574 18.730343

H 9.169845 21.812056 17.130569

H 3.565263 23.039753 20.732992

O 9.634326 32.081989 33.781502

C 10.289001 31.125156 32.963524

C 9.387906 29.941950 32.813915

C 10.514885 33.103359 34.201981

O 9.376172 29.243273 34.047970

C 8.545972 28.101625 34.022057

H 11.232142 30.777040 33.424095

H 10.547154 31.571058 31.985670

H 9.722334 29.280920 31.996914

H 8.377781 30.303268 32.548424

H 9.933062 33.788834 34.833675

H 10.926074 33.672470 33.352222

H 8.951783 27.310053 33.368847

H 7.520201 28.336800 33.696426

H 8.499036 27.723194 35.052711

H 11.352419 32.707817 34.802872

O 23.793192 41.447891 36.785477

C 23.566328 42.831562 36.973713

C 22.280937 43.161587 36.254498

C 25.097164 41.021057 37.127235

O 22.278982 44.525696 35.836956

C 20.949587 44.995556 35.683918

H 24.376482 43.435875 36.534657

H 23.527216 43.088245 38.048374

H 21.414558 42.917122 36.892551

H 22.203199 42.500557 35.371986

H 25.120142 39.930264 36.979092

H 25.350428 41.232765 38.178921

H 20.381453 44.953018 36.630486

H 20.388786 44.418129 34.930481

H 21.003368 46.044304 35.360741

H 25.870646 41.472340 36.481850

O 1.128935 2.928190 0.209750

C 2.470064 2.706705 0.605293

C 3.344268 3.624912 48.690872

C 0.208283 2.140038 0.937764

O 4.617925 3.695807 0.408255

C 5.439324 4.663395 48.680607

H 2.775155 1.653554 0.463504

H 2.615276 2.949214 1.674089

H 2.853873 4.614991 48.664471

H 3.422007 3.270439 47.647499

H 48.095360 2.409437 0.582802

H 0.255220 2.333164 2.023673

H 5.030091 5.681832 48.786701

H 5.601159 4.456090 47.608387

H 6.412780 4.634548 0.295312

H 0.356429 1.059996 0.765661

O 26.822590 2.498422 8.200789

C 27.650835 1.841303 9.154199

C 28.093315 0.433679 8.832973

C 27.522734 2.854851 7.024918

O 28.492279 48.740253 10.061649

C 28.937202 47.412815 9.870478

H 27.035761 1.760141 10.062627

H 28.533836 2.450507 9.422620

H 28.916668 0.421945 8.095181

H 27.254801 48.763233 8.390493

H 26.796677 3.346223 6.362909

H 27.929523 1.975758 6.495409

H 29.778158 47.353165 9.159088

H 28.130472 46.753738 9.509161

H 29.281898 47.043186 10.845889

H 28.349022 3.556951 7.230268

O 17.218576 31.321705 24.162333

C 16.888552 31.013681 25.503950

C 15.515152 30.406433 25.465324

C 18.409117 32.071232 24.034233

O 15.048716 30.073961 26.764408

C 13.691939 29.681839 26.649021

H 16.869970 31.919176 26.138092

H 17.616076 30.308157 25.944965

H 15.539598 29.518051 24.805763

H 14.848743 31.144224 24.981777

H 18.558729 32.246265 22.959570

H 19.289188 31.533899 24.426842

H 13.569220 28.787592 26.011459

H 13.060245 30.491995 26.239786

H 13.319865 29.440800 27.652790

H 18.338223 33.052513 24.533916

O 43.162075 47.034874 0.864425

C 43.911110 46.322506 1.838369

C 44.034813 47.138039 3.096381

C 43.291149 46.388512 48.501656

O 44.455780 46.257965 4.126552

C 44.222557 46.752762 5.431501

H 44.926125 46.074619 1.474118

H 43.416805 45.364697 2.089189

H 43.057442 47.598606 3.318354

H 44.746689 47.971169 2.958992

H 42.858452 47.048077 47.738441

H 42.764576 45.416523 48.476231

H 43.159630 46.993805 5.612893

H 44.824921 47.648479 5.653474

H 44.508583 45.954830 6.132135

H 44.347237 46.222763 48.223946

O 38.534859 47.284229 15.390475

C 39.526405 47.306717 16.400600

C 39.980129 48.741718 16.518921

C 38.131496 45.982700 15.021824

O 40.853355 0.150590 17.599941

C 42.155373 48.537838 17.364767

H 40.377140 46.653023 16.139513

H 39.120598 46.955669 17.365744

H 39.085392 0.466925 16.678312

H 40.406479 0.165255 15.550355

H 37.390278 46.100533 14.219982

H 37.657722 45.437054 15.854957

H 42.585629 0.023955 16.421625

H 42.174442 47.435303 17.344721

H 42.793911 48.872265 18.193989

H 38.969025 45.379852 14.629215

O 37.006962 1.965002 13.883600

C 38.212658 2.663191 14.130997

C 38.133450 3.936848 13.335510

C 37.050476 0.585247 14.181846

O 39.166065 4.826208 13.723231

C 39.334255 5.857846 12.773243

H 39.101040 2.085278 13.820527

H 38.325111 2.887609 15.205660

H 37.139458 4.392040 13.500769

H 38.192608 3.675761 12.262315

H 36.087776 0.159879 13.863065

H 37.178085 0.396521 15.259442

H 38.403339 6.423536 12.600163

H 39.702908 5.466704 11.808100

H 40.086720 6.547724 13.176121

H 37.852318 0.063072 13.632291

O 12.095590 44.533516 40.710590

C 12.834359 43.607487 41.488964

C 11.974825 43.136650 42.624252

C 12.877874 45.023422 39.642284

O 12.691104 42.147549 43.337112

C 11.955267 41.636131 44.427910

H 13.758433 44.056812 41.898193

H 13.136518 42.725952 40.891003

H 11.036083 42.730350 42.207687

H 11.708848 43.995209 43.265728

H 12.230534 45.669785 39.034546

H 13.262173 44.213757 38.996410

H 10.969100 41.252811 44.121838

H 11.811033 42.391525 45.217529

H 12.539536 40.801533 44.839588

H 13.732520 45.620403 40.004578

O 46.132801 5.235441 18.954271

C 45.514797 4.257096 18.135805

C 46.469673 3.124739 17.896233

C 45.183304 6.151692 19.455423

O 45.908382 2.295028 16.897352

C 46.704849 1.150448 16.668043

H 45.218994 4.685886 17.160395

H 44.600010 3.847374 18.601753

H 46.627106 2.574695 18.842796

H 47.449486 3.526638 17.581362

H 45.738235 6.903175 20.033335

H 44.454311 5.663253 20.124765

H 46.810944 0.525109 17.572073

H 47.713017 1.408602 16.298903

H 46.193916 0.558356 15.896027

H 44.630814 6.657732 18.649181

O 39.432529 16.363932 0.364740

C 40.732594 15.879892 0.634140

C 40.674900 14.381329 0.593559

C 39.376793 17.773510 0.317803

O 41.970066 13.851331 0.807709

C 41.934864 12.443706 0.922607

H 41.094398 16.207962 1.626663

H 41.454247 16.237299 48.773014

H 40.271046 14.072814 48.506058

H 39.958130 14.041035 1.361664

H 38.334888 18.039976 0.089474

H 40.022179 18.191545 48.418541

H 41.541767 11.960646 0.012223

H 41.328106 12.116613 1.784098

H 42.967972 12.104390 1.079553

H 39.651085 18.234081 1.282947

O 18.814438 47.782932 7.260092

C 17.491400 47.811779 6.760407

C 16.602528 47.921299 7.967082

C 19.770292 47.359524 6.310594

O 15.254554 48.182877 7.601852

C 14.475692 48.302174 8.779680

H 17.248890 46.901394 6.183961

H 17.344231 48.681095 6.095465

H 17.014206 48.730965 8.599754

H 16.692492 46.986958 8.554773

H 20.732992 47.289604 6.837658

H 19.875412 48.070911 5.479416

H 14.905949 0.127119 9.495470

H 14.352482 47.332142 9.295988

H 13.488546 48.684517 8.492190

H 19.532675 46.367485 5.891582

O 27.286581 40.204060 40.767792

C 27.570650 41.464520 41.344242

C 26.539501 42.404724 40.795662

C 28.067402 39.167534 41.323215

O 26.777121 43.725319 41.243523

C 25.701967 44.573120 40.899315

H 28.587130 41.812145 41.082664

H 27.504154 41.439579 42.447262

H 25.546976 42.031673 41.112488

H 26.577637 42.347031 39.693130

H 27.819023 38.244434 40.778061

H 27.843962 39.011078 42.392502

H 24.771049 44.276340 41.413177

H 25.514217 44.594143 39.813896

H 25.976255 45.586670 41.223965

H 29.147930 39.353813 41.212719

O 16.639196 38.014641 26.060350

C 15.592402 38.962669 26.154715

C 15.786997 39.802162 27.385834

C 16.528212 37.280270 24.857100

O 14.896170 40.905182 27.324230

C 14.926971 41.656174 28.519169

H 14.607213 38.463966 26.197741

H 15.585069 39.633972 25.277578

H 16.838680 40.142456 27.417128

H 15.617338 39.186600 28.288397

H 17.336409 36.536610 24.851721

H 16.646530 37.923210 23.967739

H 15.941498 42.017494 28.763144

H 14.537786 41.075329 29.372351

H 14.273764 42.525490 28.367603

H 15.565022 36.747341 24.786205

O 21.605240 5.146945 46.024750

C 22.296583 6.026038 46.894550

C 23.655313 5.453992 47.178619

C 20.283667 5.582091 45.798862

O 24.468891 6.486119 47.701771

C 25.811487 6.064663 47.804443

H 21.746538 6.191295 47.838669

H 22.438862 7.016605 46.422245

H 24.062592 5.064805 46.229607

H 23.588820 4.600812 47.879250

H 19.859766 4.949418 45.011200

H 20.245043 6.630352 45.459057

H 26.242231 5.828510 46.816811

H 25.916607 5.179214 48.452763

H 26.381086 6.896818 48.242523

H 19.666639 5.460837 46.702404

O 6.002569 36.845615 17.695282

C 6.857704 37.700748 16.957491

C 7.361300 38.751457 17.902588

C 5.441769 35.844292 16.870461

O 8.360669 39.541073 17.281649

C 8.738610 40.608891 18.124561

H 6.328684 38.181854 16.113111

H 7.712839 37.146793 16.532612

H 7.756353 38.241993 18.800749

H 6.501765 39.365551 18.224302

H 4.723534 35.278599 17.480642

H 6.202540 35.143654 16.486162

H 9.105307 40.263710 19.105839

H 7.900098 41.303661 18.292751

H 9.553164 41.147690 17.620964

H 4.899548 36.272099 16.011414

O 13.793148 1.875038 16.456827

C 13.570197 1.846193 15.057027

C 14.839942 1.406155 14.388173

C 12.591851 1.752809 17.190220

O 14.548542 0.968075 13.071489

C 15.580668 0.132988 12.587452

H 13.220124 2.827959 14.693264

H 12.803069 1.101553 14.787138

H 15.230107 0.576448 15.001289

H 15.607560 2.200664 14.389640

H 12.855384 1.814410 18.255104

H 12.100968 0.780818 17.009806

H 15.737615 48.144253 13.234303

H 16.537991 0.672274 12.488199

H 15.270199 48.665447 11.598839

H 11.874105 2.556603 16.956022

O 12.471087 3.676250 20.245043

C 11.429181 4.636015 20.203972

C 10.921185 4.636504 18.789015

C 13.048021 3.479701 21.517721

O 9.891502 5.590891 18.586109

C 9.504272 5.541021 17.223467

H 11.797343 5.638807 20.478748

H 10.607781 4.387640 20.898737

H 10.566712 3.615134 18.553841

H 11.784142 4.841365 18.129450

H 13.811727 2.698883 21.393045

H 12.317075 3.127183 22.264803

H 9.079882 4.558764 16.948200

H 10.347183 5.762016 16.544346

H 8.731277 6.305704 17.065542

H 13.537928 4.390085 21.900063

O 43.405560 45.388165 21.025370

C 42.950859 44.426929 21.961178

C 41.654709 43.867599 21.453182

C 44.687042 45.889317 21.336817

O 41.060661 43.091183 22.474064

C 39.857410 42.500557 22.030117

H 42.777290 44.879677 22.954679

H 43.677895 43.606998 22.098080

H 41.850281 43.267681 20.545244

H 41.001991 44.710018 21.160315

H 44.888477 46.704357 20.628361

H 45.466393 45.116810 21.223875

H 40.014355 41.840992 21.158848

H 39.095657 43.256439 21.770985

H 39.472134 41.889397 22.857874

H 44.736423 46.301968 22.357210

O 35.089382 9.001654 12.312675

C 35.999279 9.009476 11.223342

C 36.244232 7.605275 10.730014

C 34.851276 10.326648 12.744886

O 37.592697 7.536336 10.295357

C 37.910500 6.298370 9.692509

H 36.974201 9.413820 11.548479

H 35.637962 9.659262 10.404387

H 35.543110 7.323164 9.923283

H 36.078976 6.907085 11.568525

H 34.215183 10.269933 13.638157

H 34.327148 10.920207 11.975802

H 37.431839 6.187873 8.706830

H 37.631321 5.433946 10.319314

H 38.998856 6.285169 9.553164

H 35.785618 10.851268 13.011841

O 25.584625 4.300122 39.759624

C 25.287355 4.752380 38.451252

C 24.223450 5.805531 38.553928

C 26.588394 3.306131 39.741047

O 23.852352 6.217697 37.249958

C 22.840759 7.203376 37.288094

H 24.918215 3.928047 37.813690

H 26.178673 5.181170 37.954990

H 24.618992 6.646976 39.152866

H 23.363913 5.384075 39.108376

H 26.770275 3.013263 40.784904

H 27.536425 3.677716 39.313232

H 23.141941 8.096647 37.861118

H 21.905441 6.816634 37.722260

H 22.644211 7.506512 36.249611

H 26.276457 2.408948 39.179756

O 7.369612 2.409437 42.972858

C 7.052298 3.799459 42.823734

C 6.249477 4.393018 43.954628

C 8.611978 2.273515 43.650024

O 5.781085 5.663253 43.533657

C 5.109787 6.327217 44.585342

H 6.462650 3.900667 41.905041

H 7.959748 4.407686 42.675591

H 6.871394 4.480047 44.861099

H 5.408032 3.721720 44.200069

H 8.642291 1.285392 44.117928

H 8.719053 3.008374 44.456757

H 5.782551 6.531100 45.438034

H 4.239495 5.755171 44.951061

H 4.752380 7.286983 44.186378

H 9.467602 2.387924 42.962105

O 29.668152 6.267568 42.173950

C 30.811754 5.475504 41.917263

C 32.048252 6.247522 42.277115

C 28.482500 5.498484 42.151947

O 33.147362 5.361095 42.133369

C 34.431778 5.939986 42.261959

H 30.849890 5.182637 40.853844

H 30.812244 4.546052 42.511803

H 31.946066 6.614707 43.314133

H 32.129414 7.139815 41.633686

H 27.645945 6.201562 42.255112

H 28.440943 4.776826 42.986061

H 34.544720 6.564347 43.161098

H 34.700199 6.559458 41.392155

H 35.142677 5.103920 42.327473

H 28.357822 4.947463 41.205872

O 12.597229 28.602776 46.109333

C 13.208879 27.327652 46.036972

C 13.944226 27.251379 44.730556

C 11.997804 28.848709 47.362946

O 14.710866 26.059862 44.680687

C 15.710724 26.140047 43.686203

H 12.464730 26.514074 46.105911

H 13.941781 27.180485 46.852505

H 14.585212 28.147583 44.667484

H 13.229413 27.302227 43.888622

H 11.591994 29.869589 47.327251

H 12.718483 28.784170 48.196079

H 16.424557 26.959490 43.884712

H 15.281933 26.280367 42.680969

H 16.260767 25.190058 43.707718

H 11.167604 28.157850 47.565361

O 10.154057 38.119270 21.661955

C 10.832688 38.172077 20.420078

C 11.888773 39.239403 20.521286

C 9.145887 37.132126 21.673201

O 12.145460 39.770382 19.231983

C 13.271951 40.623558 19.253496

H 10.143300 38.444408 19.603079

H 11.270768 37.192753 20.154100

H 12.806003 38.843372 20.996035

H 11.498120 40.029026 21.184761

H 8.704385 37.137993 22.679415

H 9.551698 36.124935 21.477140

H 14.199936 40.069607 19.480846

H 13.171230 41.443005 19.984444

H 13.361914 41.062618 18.251194

H 8.346978 37.340897 20.940786

O 46.426647 27.502199 24.450800

C 45.299179 27.539360 23.587841

C 44.414219 26.381577 23.913469

C 47.299870 28.581753 24.189224

O 45.192593 25.197882 23.812750

C 44.420086 24.041567 24.068459

H 45.607693 27.459173 22.529314

H 44.764782 28.500591 23.686117

H 43.548328 26.357132 23.227013

H 44.005474 26.485229 24.933861

H 46.812408 29.560097 24.351547

H 47.699326 28.550463 23.161497

H 43.560551 23.938892 23.383959

H 44.039211 24.007341 25.102541

H 45.081604 23.179098 23.922758

H 48.141808 28.490324 24.888390

O 5.781085 35.136810 44.003517

C 5.244242 35.963589 42.951347

C 6.185428 37.021629 42.425259

C 6.672889 34.095394 43.588421

O 5.779129 37.384903 41.112000

C 6.872372 37.856228 40.344872

H 4.327991 36.412914 43.364491

H 4.933283 35.358295 42.084965

H 7.197020 36.596260 42.416458

H 6.225031 37.913921 43.069668

H 6.361442 33.164474 44.080284

H 7.698172 34.341324 43.903290

H 7.389169 38.705494 40.821575

H 6.467050 38.187721 39.381683

H 7.613586 37.058788 40.158100

H 6.653821 33.935024 42.504467

O 3.177054 13.380004 17.030340

C 1.965002 13.610289 16.334595

C 0.816999 13.105714 17.157949

C 4.267852 13.963784 16.348774

O 48.508503 13.515925 16.529676

C 47.381031 13.152163 17.299250

H 1.961090 13.116961 15.345494

H 1.798277 14.687396 16.161514

H 0.907450 13.533037 18.173943

H 0.872248 12.005137 17.255735

H 5.169925 13.753056 16.939888

H 4.157354 15.058982 16.258812

H 47.395214 13.614689 18.302530

H 47.297424 12.058431 17.418549

H 46.492649 13.516903 16.763386

H 4.406219 13.538905 15.339138

O 12.562027 37.842537 8.354313

C 13.061712 36.531719 8.152386

C 14.198469 36.571815 7.171107

C 11.370509 37.814667 9.116551

O 14.806207 35.292778 7.149594

C 15.928296 35.254154 6.292503

H 12.283826 35.854069 7.758799

H 13.426940 36.094620 9.100417

H 14.911815 37.355080 7.477176

H 13.815639 36.852947 6.173205

H 11.044883 38.856087 9.249540

H 11.521588 37.363388 10.112987

H 16.742851 35.909805 6.646487

H 15.673564 35.536263 5.255487

H 16.293524 34.217136 6.290547

H 10.561823 37.263645 8.602689

O 19.654417 44.720287 8.031131

C 19.909636 44.549164 9.415775

C 18.722031 44.931995 10.240597

C 20.791662 44.292477 7.303607

O 18.502012 46.342064 10.203439

C 18.078602 46.878902 11.443360

H 20.771618 45.162766 9.740912

H 20.158991 43.495033 9.626015

H 18.889244 44.581432 11.272235

H 17.828270 44.411285 9.861678

H 20.590225 44.446980 6.234321

H 20.992613 43.218788 7.458107

H 18.818838 46.705826 12.243735

H 17.107101 46.467716 11.764096

H 17.978371 47.962856 11.297659

H 21.699602 44.863056 7.570561

O 7.625810 9.609879 13.899245

C 7.176974 9.542408 12.558604

C 7.779333 10.634673 11.729872

C 7.276715 8.403695 14.547074

O 7.348588 10.409277 10.399987

C 7.896187 11.333351 9.484715

H 6.074931 9.613791 12.508734

H 7.468375 8.586064 12.084833

H 8.879910 10.580401 11.816412

H 7.464464 11.625731 12.104390

H 7.567627 8.494635 15.599736

H 7.813559 7.540247 14.115352

H 8.996764 11.310371 9.476402

H 7.579851 12.368412 9.694465

H 7.532425 11.049772 8.486813

H 6.193251 8.197856 14.499648

O 46.967892 21.583237 3.188299

C 45.704990 21.290369 3.757411

C 45.650230 19.802073 4.000409

C 47.115055 22.965437 2.925256

O 44.433777 19.363016 4.619392

C 43.493076 18.872622 3.670871

H 44.877724 21.596437 3.090514

H 45.564667 21.823790 4.715222

H 46.472607 19.530230 4.680019

H 45.841400 19.277943 3.048466

H 48.127140 23.112116 2.520424

H 47.018250 23.577576 3.839551

H 43.742920 17.843916 3.359424

H 43.425117 19.505293 2.769777

H 42.509357 18.867243 4.157354

H 46.386555 23.330666 2.179641

O 7.600874 22.717062 1.627152

C 6.441627 23.062244 0.873714

C 6.179072 21.920597 48.818970

C 8.246748 23.855286 2.167418

O 5.203172 22.083900 47.791733

C 3.824395 22.026695 48.127628

H 6.601017 23.978008 0.280645

H 5.597737 23.243147 1.559191

H 6.017237 20.999458 0.509952

H 7.100701 21.801788 48.239590

H 9.077437 23.494457 2.789823

H 7.573984 24.458136 2.799113

H 3.350624 21.307482 47.448994

H 3.378981 23.013351 47.958946

H 3.650826 21.715736 0.269888

H 8.667715 24.502626 1.376821

O 13.428896 17.916277 28.294264

C 13.146297 16.890507 29.228117

C 12.401170 15.793352 28.511837

C 13.898755 19.110239 28.885866

O 12.813336 14.559299 29.068726

C 12.034963 13.486101 28.583708

H 12.574250 17.269426 30.094496

H 14.082105 16.458294 29.621702

H 12.654923 15.838822 27.438150

H 11.306460 15.923407 28.585175

H 13.996053 19.848520 28.076202

H 14.883457 18.985075 29.365015

H 12.098523 13.389782 27.487043

H 10.973499 13.581442 28.872663

H 12.436372 12.568872 29.035477

H 13.190300 19.508226 29.630013

O 11.625241 43.216835 0.145701

C 10.246464 42.905388 48.874706

C 10.041603 41.547634 48.221012

C 12.189953 42.567539 1.269746

O 8.940048 41.477715 47.322365

C 7.733374 41.376511 48.053799

H 9.859233 43.666161 48.181896

H 9.684197 43.034466 0.926030

H 10.012267 40.725258 0.068450

H 10.924118 41.378952 47.588341

H 12.051586 41.474297 1.235521

H 11.778275 42.952812 2.217288

H 7.668346 42.136791 48.849773

H 7.614564 40.374207 48.501656

H 6.901707 41.551056 47.359520

H 13.268528 42.770931 1.255567

O 29.013477 31.600883 33.604507

C 29.909681 30.541866 33.317020

C 29.105394 29.269186 33.260796

C 29.639303 32.865742 33.524815

O 29.652506 28.443384 32.248711

C 28.917645 27.251869 32.072697

H 30.730593 30.492971 34.056770

H 30.379051 30.679743 32.326942

H 28.065933 29.549341 33.013397

H 29.073126 28.752388 34.236206

H 28.870222 33.619175 33.747768

H 30.045115 33.071091 32.519581

H 27.841026 27.440596 31.932377

H 29.039877 26.557100 32.918053

H 29.314167 26.775164 31.167204

H 30.456301 32.976727 34.257717

O 23.926668 12.633899 29.236427

C 23.167364 12.697460 28.042465

C 22.900898 11.304015 27.535448

C 24.345680 13.932492 29.609968

O 22.273603 11.270279 26.256899

C 20.880648 11.508876 26.297970

H 23.724253 13.234792 27.256269

H 22.218843 13.240660 28.203812

H 22.341564 10.722680 28.288883

H 23.871420 10.799441 27.399525

H 25.018444 13.822483 30.469503

H 23.494946 14.573478 29.900879

H 20.638140 12.515579 26.678846

H 20.352118 10.759350 26.907173

H 20.519819 11.432115 25.262911

H 24.909414 14.439022 28.807638

O 4.468313 29.057968 39.014011

C 4.296210 30.414255 39.398304

C 4.374928 30.653341 40.890026

C 5.824599 28.673672 39.119617

O 3.438631 31.655155 41.294861

C 4.013610 32.911209 41.605816

H 3.268972 30.667521 39.108376

H 4.961641 31.088976 38.827728

H 5.398743 30.875313 41.224453

H 4.068370 29.729755 41.399979

H 5.910161 27.661102 38.705009

H 6.170271 28.657537 40.167389

H 4.725000 33.247105 40.834778

H 4.527962 32.881874 42.578297

H 3.188788 33.635311 41.669376

H 6.482696 29.335680 38.538280

O 0.126629 24.170645 38.135895

C 48.510456 23.534060 39.300522

C 47.181549 24.071392 39.741047

C 48.485035 23.723276 36.905750

O 46.827080 23.552149 41.011280

C 45.614048 24.170645 41.397533

H 0.353497 23.751635 40.088673

H 48.454720 22.441795 39.188065

H 46.414913 23.814705 38.996410

H 47.257824 25.168545 39.760605

H 47.398636 23.847462 36.848061

H 48.729988 22.668657 36.700401

H 44.816120 24.013210 40.654362

H 45.730900 25.256065 41.545189

H 45.289890 23.728653 42.348499

H 0.045468 24.348614 36.123466

O 47.389835 23.773634 44.093971

C 48.552017 24.394573 43.555172

C 0.570579 23.327732 43.016373

C 46.521008 24.751001 44.635216

O 1.854015 23.880711 42.816402

C 2.776133 22.914099 42.359257

H 0.214639 24.957329 44.328655

H 48.298752 25.116720 42.756264

H 0.159391 22.913122 42.081055

H 0.613116 22.503889 43.750252

H 45.585201 24.250828 44.918793

H 46.272144 25.542576 43.905735

H 2.556604 22.592873 41.327129

H 2.817203 22.029629 43.016373

H 3.767190 23.383959 42.374413

H 46.947357 25.224285 45.535820

O 34.607300 0.995947 42.802711

C 34.709976 48.752476 43.642693

C 36.045238 48.095844 43.401161

C 33.427029 1.717605 43.086781

O 36.527321 47.606918 44.644505

C 37.444550 46.542034 44.497826

H 33.870487 48.049889 43.477921

H 34.670376 0.146679 44.709045

H 36.746849 48.846352 43.000729

H 35.964077 47.296452 42.644791

H 33.383026 2.562472 42.385170

H 33.431431 2.119992 44.114506

H 38.401871 46.873528 44.066589

H 37.048519 45.718189 43.882267

H 37.619099 46.137691 45.500130

H 32.522022 1.098621 42.953793

O 48.299728 6.032393 10.024001

C 47.667545 6.183961 8.765501

C 46.437401 7.015628 8.992364

C 0.493818 5.130322 9.999556

O 45.857048 7.404325 7.756843

C 44.610279 8.029176 8.000818

H 47.383476 5.209528 8.326933

H 48.330044 6.694402 8.044821

H 46.725872 7.899610 9.591789

H 45.731880 6.423536 9.605479

H 0.923097 5.120054 11.012614

H 1.280994 5.438835 9.290609

H 44.709530 8.928803 8.634468

H 43.895958 7.341254 8.486323

H 44.192734 8.330844 7.030785

H 0.172593 4.104550 9.752646

O 4.617925 35.589558 28.513304

C 4.419420 34.177048 28.496191

C 4.848699 33.500370 29.766426

C 5.807487 36.011990 27.870361

O 4.310389 32.189552 29.771317

C 4.609124 31.558348 30.998524

H 4.881946 33.696915 27.620031

H 3.334978 34.022057 28.404272

H 4.454623 34.091480 30.610804

H 5.949765 33.487656 29.858343

H 6.713959 35.587601 28.329464

H 5.846112 37.103767 27.969614

H 4.098195 32.042873 31.845346

H 5.693566 31.548080 31.207785

H 4.261007 30.518885 30.932030

H 5.808465 35.761662 26.798143

O 27.894320 20.756950 13.100825

C 27.126703 20.304203 14.199936

C 27.212265 18.804171 14.279142

C 27.780399 22.155773 12.930189

O 26.849480 18.425251 15.595336

C 26.843615 17.027405 15.800686

H 26.070618 20.621517 14.119751

H 27.525179 20.716858 15.143566

H 28.252703 18.509346 14.055702

H 26.563457 18.331377 13.517880

H 28.353422 22.425171 12.032029

H 28.191589 22.714127 13.788748

H 27.807291 16.557058 15.541553

H 26.038349 16.534567 15.232552

H 26.651953 16.865082 16.871439

H 26.738005 22.471130 12.761509

O 43.970760 35.539688 39.383636

C 43.573753 34.493870 38.507481

C 44.346260 33.254436 38.805729

C 43.275017 36.718983 39.035522

O 45.715748 33.443165 38.475212

C 46.482384 32.310318 38.827240

H 43.750744 34.768646 37.450905

H 42.491264 34.291943 38.614555

H 43.919426 32.404678 38.241016

H 44.219627 33.018288 39.874035

H 43.630466 37.522778 39.693130

H 42.182262 36.615818 39.167042

H 46.038437 31.370110 38.459076

H 46.615860 32.225735 39.915592

H 47.471973 32.433529 38.369602

H 43.475964 37.008915 37.992149

O 39.636414 44.854256 47.961880

C 38.967072 46.100040 47.944767

C 39.741047 47.027050 47.055897

C 38.821369 43.858307 48.544193

O 39.164112 48.320755 47.083767

C 39.646683 0.217571 46.015457

H 38.900089 46.521008 0.066007

H 37.933479 46.005680 47.565849

H 39.749847 46.599239 46.038437

H 40.788330 47.058342 47.394722

H 39.371906 42.908321 48.492367

H 37.867474 43.727276 48.001972

H 39.316654 48.725098 45.035160

H 40.746769 0.284558 46.009594

H 39.232559 1.225256 46.149422

H 38.596954 44.076370 0.708457

O 29.998177 21.269346 4.139265

C 29.372837 22.380678 4.759714

C 29.750290 23.660692 4.068859

C 29.567919 20.077829 4.770471

O 29.456444 24.722643 4.959685

C 29.726334 25.992392 4.401330

H 29.701887 22.470640 5.810421

H 28.270796 22.276049 4.764114

H 29.207581 23.761412 3.110560

H 30.828867 23.634779 3.839063

H 30.095963 19.240294 4.294744

H 28.480545 19.915993 4.651172

H 29.123974 26.182093 3.496813

H 30.792196 26.123425 4.147088

H 29.457422 26.740940 5.161613

H 29.805540 20.068050 5.848557

O 44.722240 34.818520 14.789582

C 44.885059 35.973366 13.981384

C 44.401016 37.175640 14.733356

C 45.407230 33.713543 14.239050

O 44.523739 38.313866 13.901200

C 44.046059 39.460400 14.575921

H 44.309589 35.888294 13.043133

H 45.941628 36.139111 13.705629

H 44.993111 37.284184 15.661830

H 43.351288 37.011852 15.029158

H 45.217529 32.853027 14.894214

H 46.497051 33.882710 14.199447

H 44.599525 39.655483 15.511241

H 42.969437 39.384129 14.814029

H 44.193222 40.315044 13.902668

H 45.049335 33.469566 13.226480

O 6.534034 14.054235 7.438061

C 6.206941 13.598554 6.137513

C 5.460837 12.298495 6.247033

C 7.339298 15.212016 7.364233

O 4.681485 12.130303 5.075072

C 4.139265 10.829266 4.971420

H 7.108036 13.467521 5.508751

H 5.559112 14.328034 5.623161

H 4.808607 12.342009 7.135904

H 6.168315 11.464873 6.402023

H 7.641944 15.467726 8.390004

H 6.800499 16.070086 6.927621

H 3.644469 10.505596 5.899405

H 4.912748 10.090984 4.703976

H 3.387293 10.852735 4.172023

H 8.249682 15.036981 6.766275

O 28.299154 36.676445 19.092150

C 27.940769 35.397900 18.592953

C 27.786755 35.481506 17.103189

C 28.370535 36.653465 20.502218

O 29.014454 35.606670 16.378111

C 29.560587 34.323723 16.107244

H 28.706430 34.646416 18.852085

H 26.984425 35.059559 19.033478

H 27.183420 34.641037 16.715960

H 27.209330 36.395313 16.903217

H 29.120062 35.931808 20.871359

H 27.395124 36.407043 20.957411

H 30.558489 34.470402 15.674542

H 28.942093 33.761456 15.385097

H 29.674507 33.713051 17.019094

H 28.667315 37.660656 20.825399

O 17.150127 37.458729 36.681335

C 16.170315 37.188351 37.666523

C 16.595684 36.011990 38.501125

C 16.748716 38.563217 35.897583

O 15.579691 35.758724 39.456001

C 15.853002 34.617569 40.242195

H 15.193438 36.966866 37.201065

H 16.022659 38.063046 38.323643

H 17.560825 36.233475 38.992496

H 16.755562 35.143166 37.836182

H 17.579895 38.793011 35.218948

H 16.547279 39.462357 36.506786

H 16.763874 34.745178 40.850914

H 15.954209 33.703762 39.631527

H 14.998356 34.478714 40.919849

H 15.851046 38.335869 35.295712

O 33.489613 14.917192 12.457885

C 33.778076 16.300859 12.466686

C 33.255417 16.843569 11.171026

C 34.028900 14.237094 13.567752

O 33.610867 18.211100 10.995991

C 33.404049 18.516680 9.629437

H 34.866920 16.485186 12.514112

H 33.306751 16.811300 13.326221

H 32.155815 16.709604 11.130445

H 33.686161 16.206985 10.373586

H 33.751190 13.177098 13.466055

H 33.614288 14.615524 14.517250

H 32.402725 18.216478 9.290121

H 34.151619 18.017487 8.986985

H 33.480320 19.601612 9.490092

H 35.129963 14.304078 13.596109

O 47.473442 11.081553 2.614787

C 47.783421 11.797343 3.800437

C 47.252934 11.031194 4.979242

C 47.886585 11.797343 1.468740

O 47.516464 11.761163 6.163426

C 47.070076 11.064441 7.310451

H 47.327251 12.805024 3.799948

H 48.875198 11.936688 3.916313

H 47.735020 10.037692 5.005156

H 46.167515 10.870337 4.838921

H 47.588829 11.208674 0.590136

H 0.086541 11.940111 1.442338

H 47.571228 10.088052 7.429750

H 45.978298 10.898694 7.298717

H 47.316982 11.687335 8.182210

H 47.407436 12.788401 1.397845

O 0.230283 15.020846 11.575371

C 48.299240 14.307989 10.665476

C 48.684517 14.696687 9.269587

C 48.879108 14.624815 12.911610

O 48.009308 15.892604 8.913647

C 48.430275 16.377621 7.654657

H 47.230930 14.545609 10.821444

H 48.413162 13.218657 10.814599

H 48.446899 13.895823 8.546461

H 0.887403 14.832119 9.251985

H 0.740723 15.116675 13.538905

H 0.088004 13.534993 13.054377

H 48.347157 15.615871 6.861126

H 0.575958 16.748716 7.686926

H 47.771198 17.215643 7.388680

H 47.877785 14.932350 13.259727

O 34.540318 8.091269 39.375816

C 33.631401 7.086034 38.953873

C 34.475780 6.027504 38.288929

C 33.932091 9.162999 40.067162

O 33.836750 4.808118 37.913433

C 33.378136 4.094772 39.050190

H 32.858406 7.473265 38.268394

H 33.092602 6.656755 39.809986

H 35.334827 5.836333 38.958271

H 34.891369 6.454339 37.363388

H 34.721222 9.907147 40.249043

H 33.516991 8.849596 41.039639

H 34.150642 3.999431 39.831497

H 33.099934 3.085136 38.719677

H 32.489265 4.569032 39.496582

H 33.129272 9.641660 39.480934

O 22.067276 37.422058 48.404362

C 22.559626 36.983978 0.764683

C 23.892445 37.609318 1.050706

C 21.072796 36.542969 47.917877

O 24.281141 37.185905 2.346854

C 25.670673 37.318893 2.564427

H 22.708750 35.889271 0.788641

H 21.853613 37.236755 1.573370

H 23.827906 38.710384 0.996924

H 24.604813 37.280758 0.271844

H 20.818554 36.873482 46.903351

H 20.160946 36.561058 48.533924

H 26.012436 38.360313 2.458819

H 26.259344 36.686222 1.876994

H 25.869181 36.985939 3.592643

H 21.432648 35.502041 47.852360

O 1.511277 34.233269 24.793537

C 0.283579 33.597668 24.493336

C 48.654202 34.266518 23.258305

C 2.177688 33.682739 25.905849

O 47.449486 33.665138 22.822670

C 46.950287 34.401951 21.723070

H 48.446899 33.698387 25.317669

H 0.420967 32.520069 24.297277

H 0.544665 34.192688 22.481888

H 48.513882 35.340691 23.476856

H 3.095404 34.272385 26.037859

H 2.465667 32.630566 25.741570

H 46.044304 33.895912 21.369087

H 47.666080 34.444977 20.883093

H 46.680401 35.436523 22.003716

H 1.576795 33.753632 26.829435

O 46.550835 16.050528 29.938528

C 46.144047 14.753891 29.524893

C 46.660843 13.689494 30.453859

C 45.975368 16.992203 29.047213

O 46.536167 12.453485 29.778648

C 46.942467 11.361709 30.575111

H 46.546432 14.532407 28.520149

H 45.042980 14.676640 29.453999

H 46.100533 13.681183 31.403845

H 47.713993 13.898755 30.702724

H 46.385090 17.982283 29.286299

H 44.875278 17.034250 29.148420

H 46.338638 11.271256 31.493809

H 48.005882 11.424780 30.856735

H 46.796764 10.454747 29.970798

H 46.214943 16.773653 27.991127

O 20.129654 48.224922 37.821022

C 20.232819 0.059160 36.606525

C 19.094595 48.571575 35.702988

C 21.284014 48.382359 38.623356

O 19.151310 0.471327 34.532986

C 18.310843 48.860039 33.514057

H 21.181339 48.730476 36.089729

H 20.205439 1.150936 36.778141

H 18.135805 48.730476 36.220764

H 19.177223 47.494465 35.474171

H 21.112400 47.815693 39.549873

H 21.474207 0.544666 38.885910

H 17.247423 48.878132 33.805462

H 18.586109 47.832314 33.219234

H 18.443830 0.617027 32.638390

H 22.186087 47.978992 38.134426

O 36.490650 37.824936 5.027647

C 35.273224 37.673859 4.324568

C 34.717800 39.032589 4.014588

C 37.035320 36.568882 5.375274

O 33.405518 38.861950 3.510992

C 32.813423 40.073517 3.094425

H 34.527115 37.129681 4.930350

H 35.414520 37.108170 3.384360

H 35.379807 39.537651 3.288530

H 34.719265 39.637882 4.939151

H 37.946678 36.765427 5.957098

H 37.304226 35.975811 4.483959

H 33.440231 40.625027 2.376679

H 32.595364 40.741879 3.942715

H 31.869305 39.810963 2.595719

H 36.345928 35.972389 5.998168

O 13.557484 12.801112 13.499790

C 12.369390 13.367291 12.967837

C 11.861881 12.488688 11.848680

C 13.856219 13.293952 14.792516

O 10.449859 12.586963 11.695646

C 10.126677 13.655270 10.825844

H 12.541981 14.406753 12.633410

H 11.568525 13.408360 13.725676

H 12.057453 11.444338 12.124926

H 12.400681 12.663236 10.899672

H 14.891279 13.003530 15.017424

H 13.188832 12.866140 15.558666

H 10.492884 13.447966 9.809851

H 10.550089 14.617481 11.159781

H 9.032945 13.746699 10.793574

H 13.780436 14.388173 14.857544

O 42.885342 44.805851 42.657013

C 42.230667 43.982986 41.706047

C 42.690746 42.562649 41.872768

C 42.373924 46.121555 42.615452

O 42.357788 41.841969 40.701298

C 42.510822 40.448524 40.885624

H 42.464375 44.305679 40.674900

H 41.131557 44.023075 41.818989

H 42.229691 42.119678 42.771908

H 43.780079 42.555313 42.032162

H 42.928856 46.705826 43.362045

H 41.300236 46.156757 42.873116

H 41.812145 40.059338 41.643951

H 43.537083 40.172279 41.182896

H 42.278580 39.965462 39.924881

H 42.513268 46.587994 41.624886

O 37.179550 36.093155 18.765057

C 37.503223 34.714375 18.615444

C 37.525223 34.053345 19.972708

C 36.826057 36.647598 17.511444

O 38.581306 33.109226 19.991777

C 38.699139 32.512245 21.266413

H 38.503567 34.609745 18.161230

H 36.794277 34.201000 17.939745

H 36.558613 33.577618 20.217173

H 37.681194 34.834164 20.736414

H 36.466694 37.669456 17.687458

H 36.004169 36.092175 17.033274

H 37.785824 31.967089 21.562214

H 38.930401 33.251015 22.054075

H 39.530319 31.796455 21.207253

H 37.678257 36.677422 16.812279

O 2.352723 36.937042 20.636673

C 0.973455 37.008915 20.981855

C 0.017112 37.320362 19.860744

C 2.651455 35.854069 19.771759

O 47.589806 37.260223 20.376562

C 46.654976 37.360455 19.318035

H 0.893272 37.797070 21.746050

H 0.635607 36.073109 21.452206

H 0.146190 36.570835 19.063791

H 0.236642 38.310440 19.420710

H 2.175240 35.956741 18.783146

H 2.361034 34.881100 20.198103

H 46.776718 36.551769 18.575352

H 46.729294 38.328041 18.790480

H 45.650230 37.277336 19.755135

H 3.737854 35.857491 19.610903

O 43.522903 18.037531 41.079239

C 42.286892 17.507534 40.627960

C 42.114792 17.956858 39.203224

C 43.940937 17.443485 42.286407

O 40.974121 17.453262 38.524105

C 39.776737 18.102070 38.908890

H 41.452293 17.862007 41.259167

H 42.277603 16.404022 40.656807

H 42.980194 17.584784 38.635578

H 42.157814 19.057436 39.170956

H 44.872345 17.939257 42.582207

H 44.153133 16.373220 42.151947

H 39.851051 19.196781 38.806702

H 39.485825 17.867386 39.946396

H 38.986137 17.740753 38.236614

H 43.205097 17.566204 43.097046

O 7.824804 9.246117 19.189445

C 7.119281 10.084629 20.086628

C 6.776542 9.326302 21.333395

C 8.073179 9.948218 17.988640

O 6.445538 10.295846 22.306850

C 6.114044 9.751669 23.563396

H 6.192762 10.488483 19.635349

H 7.736308 10.951987 20.383896

H 7.649767 8.729321 21.639463

H 5.944876 8.620778 21.150536

H 8.625179 9.264697 17.334452

H 8.686784 10.852735 18.144117

H 6.996559 9.352704 24.088015

H 5.353762 8.957650 23.487123

H 5.701878 10.579913 24.156954

H 7.141771 10.242064 17.472332

O 29.585032 17.034250 25.597338

C 28.933781 17.733908 26.644131

C 28.371513 16.736982 27.620522

C 30.013334 17.933880 24.596010

O 27.084656 16.221165 27.282183

C 26.037859 17.086079 27.686525

H 28.120695 18.374893 26.260323

H 29.646639 18.403740 27.157993

H 28.375425 17.141815 28.642868

H 29.046232 15.869625 27.638123

H 30.612759 17.364767 23.872398

H 30.639162 18.746477 24.999866

H 26.072084 18.060022 27.169239

H 26.038837 17.273827 28.773413

H 25.094229 16.588837 27.426414

H 29.156242 18.384670 24.069435

O 38.175495 46.402687 36.499943

C 37.352142 47.351208 37.162926

C 35.904427 46.946377 37.066120

C 39.526897 46.821209 36.574257

O 35.060047 47.867027 37.766266

C 34.269451 47.279339 38.789101

H 37.479263 48.363781 36.740982

H 37.606384 47.419659 38.234657

H 35.810066 45.932343 37.467529

H 35.579288 46.911663 36.015415

H 40.147343 46.048706 36.101955

H 39.876965 46.945889 37.612255

H 34.885014 46.825611 39.584591

H 33.588375 46.507809 38.393070

H 33.667091 48.086559 39.229626

H 39.693619 47.773155 36.042793

O 9.465158 1.063907 9.031966

C 10.240108 2.193820 9.385462

C 10.317848 2.222667 10.882560

C 8.921958 1.114756 7.729951

O 11.196451 3.253327 11.295704

C 11.149025 3.364802 12.703816

H 9.785405 3.137940 9.042234

H 11.253166 2.133193 8.949338

H 10.662053 1.233565 11.239965

H 9.294521 2.375212 11.277614

H 8.318132 0.205350 7.603808

H 9.700332 1.124045 6.953045

H 11.374421 2.405526 13.199589

H 10.164813 3.723675 13.053888

H 11.913219 4.092816 13.004018

H 8.262883 1.986025 7.592563

O 8.414940 7.832138 31.470829

C 7.212177 7.631188 32.190041

C 6.286636 6.857704 31.289925

C 9.390841 8.492190 32.247246

O 5.061382 6.414735 31.869795

C 4.127530 7.465441 32.038475

H 6.762363 8.593887 32.488777

H 7.389658 7.042030 33.105804

H 6.825924 5.943408 31.001457

H 6.127246 7.436106 30.361452

H 10.297313 8.557706 31.630707

H 9.638726 7.929924 33.163010

H 3.895778 7.973927 31.091908

H 4.474180 8.231103 32.749866

H 3.202478 7.018561 32.431084

H 9.078904 9.513072 32.526913

O 19.781538 13.056334 11.965534

C 19.866611 13.656736 10.686499

C 21.305038 13.683628 10.256242

C 18.459965 13.040199 12.462775

O 21.390110 14.484491 9.093572

C 22.718039 14.599389 8.621757

H 19.500404 14.700109 10.700190

H 19.267675 13.108649 9.936973

H 21.667334 12.655902 10.072894

H 21.911308 14.101172 11.079108

H 18.502502 12.600652 13.469477

H 17.785244 12.430016 11.837436

H 23.138517 13.623978 8.322533

H 23.389338 15.063870 9.365905

H 22.691149 15.248198 7.733863

H 18.037531 14.052279 12.557138

O 37.346764 8.074157 47.879742

C 36.563992 8.029176 46.699959

C 37.501266 7.962193 45.529953

C 36.533676 8.021841 0.140810

O 36.760052 7.988105 44.322792

C 37.538425 7.587674 43.211945

H 35.910786 8.916580 46.611950

H 35.911762 7.136393 46.677956

H 38.089447 7.034696 45.631161

H 38.210701 8.808038 45.579334

H 37.203506 8.037976 1.011101

H 35.932297 7.098257 0.182857

H 37.824448 6.523278 43.272083

H 38.453209 8.193455 43.102425

H 36.916019 7.729463 42.316231

H 35.857979 8.889689 0.209260

O 2.136615 39.267273 8.261906

C 2.847028 38.336353 7.469353

C 2.554160 36.943398 7.944591

C 2.329742 40.592270 7.816003

O 3.183410 36.059906 7.034696

C 2.967304 34.702152 7.351521

H 3.938315 38.506992 7.523135

H 2.555138 38.402847 6.405446

H 1.462873 36.789875 7.976860

H 2.918411 36.806988 8.978674

H 1.874550 41.250366 8.568462

H 1.840814 40.770729 6.844014

H 1.896063 34.449379 7.376945

H 3.412228 34.424442 8.320087

H 3.450854 34.107128 6.563859

H 3.398050 40.849934 7.730440

O 27.330097 17.165283 21.567591

C 26.331705 16.972157 20.580936

C 25.218416 16.200140 21.228765

C 28.419430 17.928989 21.097733

O 24.375505 15.643251 20.237707

C 23.427475 14.785672 20.836643

H 26.719427 16.382021 19.730200

H 25.959143 17.929968 20.173658

H 24.656639 16.852371 21.922554

H 25.685345 15.407100 21.837969

H 29.159666 17.969570 21.907396

H 28.122160 18.963072 20.852291

H 22.746397 15.328871 21.511364

H 23.909069 13.961828 21.390110

H 22.828537 14.336836 20.036268

H 28.899067 17.472818 20.216684

O 1.912197 47.832314 15.334249

C 1.363131 48.523170 14.230248

C 48.872753 47.988770 14.009254

C 3.174609 48.329552 15.723435

O 48.309021 48.602375 12.865163

C 47.002113 48.111492 12.646611

H 1.964513 48.373070 13.315954

H 1.304460 0.717748 14.414086

H 48.273327 48.193142 14.914260

H 0.036181 46.889660 13.898755

H 3.534461 47.678303 16.533100

H 3.107626 0.468394 16.109200

H 46.335709 48.283596 13.510547

H 46.997223 47.032917 12.413393

H 46.589458 48.656647 11.787565

H 3.909468 48.304619 14.901058

O 31.706491 38.835060 28.655092

C 32.311787 38.999340 27.385345

C 33.612820 38.248837 27.375078

C 30.407410 39.387550 28.706919

O 34.456711 38.812569 26.385977

C 35.787575 38.372536 26.566391

H 32.538158 40.064228 27.198088

H 31.657600 38.650738 26.567858

H 33.459789 37.167328 27.201019

H 34.052856 38.348091 28.378845

H 30.051470 39.272163 29.740513

H 29.707264 38.864395 28.033663

H 35.866291 37.273426 26.580568

H 36.229076 38.767590 27.496822

H 36.379177 38.755367 25.726414

H 30.388830 40.457813 28.453655

O 41.254280 4.824253 9.094061

C 42.521088 4.314789 9.479337

C 42.336273 3.425430 10.672810

C 41.341797 5.787930 8.062912

O 42.434551 4.221405 11.843303

C 42.383701 3.410273 13.000107

H 43.212433 5.127388 9.761936

H 42.992416 3.776968 8.636913

H 43.092159 2.619676 10.695788

H 41.347664 2.935524 10.603381

H 40.329712 5.907228 7.653190

H 42.008694 5.471104 7.244935

H 43.107315 2.579095 12.959036

H 41.377487 2.986372 13.162919

H 42.649677 4.047346 13.853774

H 41.682087 6.762363 8.450143

O 8.903868 15.785040 29.851988

C 7.755376 15.154813 30.398121

C 7.260581 14.128064 29.410976

C 9.254918 16.951622 30.569246

O 6.831302 12.978104 30.120409

C 6.264146 12.043274 29.225672

H 6.968202 15.887715 30.650408

H 8.021841 14.631659 31.331972

H 8.101537 13.873332 28.741631

H 6.451405 14.527029 28.770969

H 10.222017 17.295828 30.182013

H 9.366393 16.767784 31.649286

H 6.923709 11.836458 28.366623

H 5.288734 12.387968 28.837952

H 6.117467 11.107467 29.782072

H 8.514681 17.755909 30.418165

O 39.492180 5.559600 47.400589

C 38.505035 4.548986 47.313072

C 38.346622 4.027789 45.915230

C 39.621258 5.937541 48.756878

O 37.328186 3.051400 46.007633

C 36.922375 2.500867 44.774071

H 37.516422 4.929372 47.635277

H 38.752922 3.688473 47.963837

H 39.295631 3.596066 45.550976

H 38.066467 4.844788 45.227306

H 40.358562 6.747206 48.809193

H 39.966930 5.097075 0.490396

H 37.725685 1.925887 44.283188

H 36.547367 3.265550 44.072456

H 36.101467 1.811478 45.010712

H 38.672249 6.311082 0.283579

O 14.878568 16.525766 15.142100

C 16.249521 16.190363 14.984177

C 16.913486 16.126801 16.320415

C 14.253229 16.736006 13.889956

O 16.508163 14.961686 17.022028

C 17.044519 14.973420 18.330399

H 16.763874 16.945267 14.366172

H 16.368332 15.217394 14.473736

H 16.642620 17.043053 16.875839

H 18.011129 16.138535 16.194763

H 13.184921 16.909575 14.082593

H 14.340259 15.860335 13.224524

H 16.714005 15.854957 18.906357

H 18.147541 14.948974 18.331377

H 16.677334 14.075747 18.845240

H 14.660505 17.616076 13.363869

O 4.506938 23.746744 25.153877

C 3.556462 24.762737 24.850254

C 2.670036 24.954885 26.050085

C 5.796731 23.978985 24.619480

O 1.478029 25.626673 25.674587

C 0.840956 26.196274 26.801077

H 2.951658 24.453735 23.981918

H 4.039034 25.719080 24.590633

H 3.238170 25.510796 26.816721

H 2.439262 23.963829 26.480339

H 6.388822 23.073490 24.811630

H 6.290547 24.833632 25.112806

H 1.444782 27.012783 27.232311

H 0.641474 25.454569 27.592163

H 48.777905 26.619194 26.463717

H 5.781573 24.164288 23.533081

O 23.164431 44.593655 0.327584

C 23.301331 43.715542 48.103180

C 22.973259 42.275650 48.406319

C 24.424887 44.843498 0.928476

O 22.613409 41.641998 47.189865

C 22.470640 40.246105 47.355122

H 24.299232 43.782524 47.645542

H 22.594830 44.074902 47.346321

H 22.127903 42.251202 0.219530

H 23.827417 41.774006 0.003911

H 24.261097 45.540710 1.758673

H 25.126499 45.317757 0.221972

H 21.700581 39.990398 48.102692

H 23.418184 39.764027 47.652878

H 22.168974 39.830029 46.383133

H 24.894747 43.923824 1.317171

O 2.945302 15.425190 21.004347

C 2.340498 14.145664 21.116800

C 2.580073 13.602955 22.503889

C 2.710128 16.002613 19.735090

O 1.547457 12.678392 22.804579

C 1.608573 12.255958 24.153532

H 2.717462 13.452364 20.343315

H 1.246277 14.213626 20.977455

H 2.574695 14.454179 23.206966

H 3.570152 13.127227 22.601187

H 3.208345 16.983402 19.728733

H 1.634975 16.157116 19.536585

H 1.395401 13.081758 24.852699

H 2.587896 11.817389 24.412174

H 0.839000 11.482963 24.287010

H 3.132562 15.394386 18.916136

O 3.358935 37.639145 35.655563

C 2.669058 36.457893 36.023727

C 3.493391 35.603249 36.940956

C 2.500378 38.456631 34.885502

O 2.669547 34.540806 37.382946

C 3.341823 33.729675 38.321686

H 2.409926 35.863846 35.130943

H 1.723960 36.689648 36.547855

H 3.846397 36.221741 37.786312

H 4.379817 35.212105 36.412914

H 3.082202 39.320080 34.535431

H 1.650621 38.824306 35.482483

H 3.687006 34.315411 39.190510

H 4.209670 33.210434 37.878231

H 2.621632 32.978191 38.675182

H 2.095546 37.930058 34.004944

O 8.696074 45.480572 11.601772

C 8.797771 46.517097 10.644940

C 7.700616 47.508156 10.911406

C 9.632370 44.454311 11.355842

O 7.320719 48.063091 9.666595

C 6.225031 0.050848 9.789805

H 8.637402 46.124977 9.623569

H 9.795672 46.991360 10.663031

H 8.007174 48.290928 11.628175

H 6.865527 46.957134 11.373932

H 9.463202 43.673981 12.110258

H 10.672810 44.811230 11.444826

H 6.402512 0.823843 10.554488

H 5.289712 48.411694 10.028402

H 6.100843 0.544665 8.817817

H 9.498893 44.004498 10.359406

O 30.834732 37.667503 13.965250

C 30.839622 38.757320 13.055355

C 30.971142 40.067162 13.778479

C 30.832777 36.463272 13.220613

O 31.249344 41.069950 12.815781

C 31.184317 42.373432 13.358001

H 29.916037 38.767101 12.457885

H 31.678133 38.683495 12.339565

H 31.794010 39.990398 14.509427

H 30.040714 40.282776 14.335368

H 30.875803 35.626717 13.928581

H 31.710892 36.387001 12.557138

H 31.985180 42.556782 14.092860

H 30.211838 42.582207 13.836662

H 31.308992 43.074554 12.520468

H 29.919460 36.351307 12.610431

O 1.774809 46.538120 0.127609

C 1.767475 47.462685 47.944279

C 2.196265 46.717560 46.706314

C 1.852548 47.155151 1.396380

O 2.895432 47.610344 45.854111

C 3.598999 46.906773 44.849365

H 2.501844 48.269413 48.107090

H 0.777396 47.944767 47.846497

H 1.354819 46.236942 46.175335

H 2.857784 45.902515 47.051495

H 1.703425 46.363575 2.143463

H 1.078575 47.925213 1.540612

H 2.935524 46.254543 44.256298

H 4.412086 46.291214 45.269840

H 4.046857 47.655323 44.182468

H 2.842627 47.609364 1.563594

O 19.450045 17.438595 3.261639

C 19.958530 16.710581 2.152261

C 18.917112 15.669653 1.838858

C 20.099831 18.684872 3.425430

O 19.147398 14.717222 0.809665

C 19.536585 15.311269 48.481121

H 20.938829 16.261257 2.391347

H 20.093962 17.368677 1.277569

H 17.990105 16.227520 1.629597

H 18.762611 15.074627 2.749731

H 19.746334 19.115129 4.371017

H 19.854389 19.385506 2.607942

H 19.424131 14.543163 47.706173

H 20.590225 15.631516 48.520725

H 18.917112 16.179115 48.202435

H 21.195028 18.587088 3.488012

O 18.178831 2.134660 19.821629

C 17.884008 1.142625 20.786283

C 16.437269 0.741704 20.676764

C 19.566898 2.402103 19.802561

O 16.295969 48.323685 21.209698

C 14.930394 47.956017 21.298193

H 18.116737 1.496120 21.807655

H 18.494190 0.241530 20.605381

H 16.146847 0.765661 19.609924

H 15.791396 1.469718 21.200407

H 19.749269 3.135006 19.005610

H 20.162413 1.501009 19.575211

H 14.417509 47.996105 20.320335

H 14.374483 48.597488 22.005182

H 14.890791 46.920952 21.668310

H 19.924793 2.826004 20.757927

O 31.914776 20.795574 15.716102

C 32.371433 21.806189 16.600084

C 31.489408 23.021173 16.535055

C 32.605141 19.583033 15.943941

O 31.799875 23.797104 17.678169

C 31.122223 25.036047 17.706528

H 33.416271 22.094166 16.380066

H 32.345520 21.462961 17.650301

H 30.430387 22.704838 16.542391

H 31.660532 23.583441 15.600714

H 32.178795 18.834484 15.260910

H 32.475574 19.219759 16.979004

H 30.026047 24.916748 17.726574

H 31.396999 25.678988 16.853348

H 31.428289 25.547955 18.628645

H 33.685673 19.677397 15.739080

O 36.799652 13.238214 16.717915

C 37.304718 12.527802 15.602181

C 38.432674 11.683912 16.138046

C 35.702011 14.064503 16.401089

O 39.089794 10.829755 15.203705

C 38.351513 9.640193 14.969997

H 37.674347 13.209368 14.814029

H 36.525364 11.889751 15.147967

H 38.053265 11.122622 17.006872

H 39.200779 12.364500 16.538479

H 35.407677 14.577876 17.326141

H 34.841499 13.478277 16.041239

H 37.381477 9.832831 14.479603

H 38.164249 9.077437 15.901405

H 38.952404 9.008987 14.299188

H 35.962608 14.830653 15.650585

O 11.068841 24.882524 0.445902

C 11.387133 26.253477 0.611649

C 11.676089 26.561501 2.051053

C 11.165648 24.544674 47.967747

O 11.751874 27.971569 2.184530

C 12.320496 28.374935 3.413695

H 10.544710 26.884195 0.276733

H 12.276982 26.538523 0.020046

H 12.620699 26.065727 2.341965

H 10.870825 26.149334 2.682748

H 10.905539 23.486633 47.871429

H 12.188975 24.682552 47.574158

H 13.351157 27.996508 3.535438

H 11.719115 28.050289 4.280076

H 12.356188 29.474535 3.401961

H 10.467460 25.124054 47.343876

O 3.856664 42.508377 39.588989

C 4.643349 41.476250 38.990051

C 4.668284 41.547146 37.475353

C 2.498911 42.122124 39.723934

O 5.852957 40.981457 36.911617

C 5.695522 39.582634 36.733158

H 4.368572 40.471504 39.361149

H 5.674499 41.655685 39.328880

H 4.689308 42.614475 37.213287

H 3.751544 41.130089 37.018208

H 2.027096 41.885971 38.753410

H 1.963046 42.965034 40.181568

H 5.002711 39.359680 35.905403

H 5.328337 39.074146 37.641590

H 6.678757 39.163620 36.482338

H 2.384502 41.247921 40.387409

O 37.669949 42.289825 11.737206

C 36.956600 43.512146 11.591994

C 37.824448 44.730556 11.391045

C 38.311420 41.894283 10.541777

O 37.937878 44.990177 9.996133

C 39.025742 45.858025 9.736023

H 36.360596 43.584511 12.512645

H 36.240322 43.453476 10.753971

H 38.807682 44.536942 11.856014

H 37.410324 45.621872 11.895129

H 38.778835 40.917892 10.730502

H 39.101528 42.606651 10.252332

H 39.996758 45.376431 9.953107

H 38.958271 46.794811 10.315891

H 39.005695 46.109333 8.666738

H 37.606384 41.783787 9.700332

O 48.821415 1.781165 8.825639

C 1.151425 1.149959 8.492680

C 2.077944 2.198220 7.949480

C 48.004417 0.931897 9.605968

O 3.311021 1.600261 7.586695

C 4.353904 2.553671 7.548560

H 0.996435 0.352028 7.745597

H 1.631553 0.688411 9.376172

H 2.207021 2.961437 8.737632

H 1.612484 2.699372 7.082122

H 47.064209 1.468251 9.800562

H 48.475258 0.692811 10.575024

H 4.518672 3.035265 8.527882

H 4.168111 3.340845 6.798544

H 5.269177 2.009494 7.278182

H 47.765820 48.879597 9.089171

O 42.175907 38.929424 23.940847

C 40.948208 38.687405 23.254393

C 40.994167 37.450905 22.397791

C 42.302540 38.206791 25.152901

O 39.850075 37.360455 21.560745

C 40.044670 36.280903 20.670898

H 40.799088 39.555252 22.593363

H 40.090141 38.655136 23.945248

H 41.081684 36.555191 23.037798

H 41.919220 37.516422 21.796410

H 43.253506 38.519215 25.607603

H 42.331875 37.115990 24.990576

H 40.029026 35.309891 21.194050

H 40.989769 36.362064 20.108631

H 39.233047 36.305347 19.938972

H 41.490429 38.436588 25.863314

O 17.959793 10.163836 13.572640

C 18.945471 9.260296 13.108649

C 20.162901 10.024001 12.669102

C 16.802011 9.472980 13.990675

O 21.259079 9.126819 12.642210

C 22.454506 9.807406 12.315118

H 18.560684 8.641803 12.279428

H 19.259361 8.571885 13.914402

H 20.334515 10.841979 13.391249

H 20.001066 10.494840 11.681468

H 16.092577 10.225929 14.360793

H 17.016651 8.757190 14.803272

H 22.709728 10.586758 13.053888

H 22.400726 10.280689 11.320639

H 23.262217 9.062769 12.302896

H 16.326773 8.929781 13.157541

O 37.587807 41.626350 1.101555

C 36.171383 41.679157 1.114756

C 35.612537 40.853844 48.886932

C 38.110470 41.916775 2.381568

O 34.212246 40.818153 0.183837

C 33.548286 39.902878 48.232258

H 35.812019 42.721550 1.037505

H 35.756771 41.265522 2.049586

H 36.059906 39.843719 0.049871

H 35.883892 41.288502 47.908100

H 39.204201 41.931442 2.290628

H 37.829826 41.144268 3.117405

H 33.857773 38.859020 48.416096

H 33.693005 40.133167 47.162971

H 32.477531 39.993332 48.463520

H 37.773109 42.896095 2.757065

O 24.267941 7.589629 28.112383

C 25.482927 6.965757 27.730042

C 25.262911 6.135068 26.493052

C 24.444933 8.417384 29.243759

O 25.626673 6.905619 25.354830

C 25.633028 6.117956 24.180424

H 25.872601 6.357531 28.565617

H 26.257389 7.717239 27.493399

H 24.201937 5.830955 26.445625

H 25.847668 5.199260 26.527765

H 23.460722 8.838351 29.492624

H 25.133833 9.254918 29.043791

H 24.660061 5.630495 23.995607

H 26.414825 5.339582 24.211714

H 25.849623 6.790721 23.340933

H 24.812605 7.855606 30.119431

O 9.117529 41.142799 8.553796

C 9.634815 42.379299 8.097626

C 9.905682 42.234089 6.630352

C 8.861820 41.115910 9.943329

O 10.478705 43.423653 6.123334

C 10.765217 43.281864 4.747491

H 10.579913 42.640388 8.608556

H 8.925381 43.209499 8.263861

H 8.959605 41.985226 6.113555

H 10.592625 41.379444 6.501765

H 8.492680 40.106762 10.177036

H 8.091269 41.846371 10.244997

H 9.855322 43.094112 4.150510

H 11.488831 42.472198 4.558764

H 11.216987 44.223049 4.405730

H 9.770737 41.297791 10.540798

O 29.995243 15.445724 6.109644

C 30.251442 15.728324 7.472775

C 29.732201 17.102213 7.791067

C 30.319891 14.112906 5.776684

O 30.278332 17.495800 9.038812

C 29.764471 18.744522 9.455379

H 31.336372 15.708767 7.682037

H 29.775227 14.990043 8.143584

H 28.625269 17.092434 7.809647

H 30.036802 17.792578 6.983847

H 30.084719 13.987741 4.710821

H 29.720955 13.388315 6.354597

H 30.173702 18.940092 10.454747

H 28.666826 18.738655 9.537519

H 30.054892 19.565922 8.778213

H 31.390154 13.888977 5.928741

O 24.320257 13.528637 33.843105

C 23.303776 13.315954 32.876499

C 22.013004 13.033842 33.607445

C 25.483906 14.087971 33.265194

O 20.961321 13.700251 32.923920

C 19.820652 13.884089 33.737988

H 23.590286 12.518513 32.167061

H 23.137051 14.231228 32.281471

H 22.139637 13.440630 34.624413

H 21.790543 11.957223 33.719898

H 26.241255 14.160821 34.057255

H 25.303001 15.097608 32.858406

H 20.057293 14.450266 34.656193

H 19.350792 12.927257 34.022545

H 19.099483 14.471292 33.153229

H 25.895584 13.463611 32.456505

O 38.120739 14.515295 38.448318

C 37.557983 13.523259 37.605896

C 37.866985 13.878710 36.179207

C 37.731060 14.371550 39.797272

O 37.299828 12.897432 35.334827

C 37.358498 13.267550 33.972183

H 36.458382 13.461165 37.714928

H 37.966728 12.521935 37.831779

H 38.961205 13.946182 36.042793

H 37.447487 14.877102 35.966034

H 38.176968 15.213973 40.345360

H 38.105091 13.432319 40.233395

H 38.395515 13.376581 33.611843

H 36.815300 14.208247 33.771236

H 36.877392 12.459352 33.404049

H 36.634884 14.414576 39.923416

O 45.542175 39.525917 37.557491

C 45.682987 40.445103 36.492119

C 45.810108 39.654995 35.216995

C 45.250286 40.140987 38.792038

O 45.677608 40.529686 34.106152

C 45.240509 39.857899 32.938591

H 46.555233 41.105156 36.642220

H 44.796070 41.097820 36.397266

H 45.018532 38.888355 35.226772

H 46.772320 39.116196 35.183746

H 45.152988 39.331810 39.529827

H 44.305679 40.710098 38.763680

H 44.266563 39.355770 33.084290

H 45.976345 39.114731 32.591938

H 45.114853 40.615738 32.151905

H 46.056530 40.815220 39.118641

O 12.406059 39.938572 33.626022

C 11.877527 41.234722 33.814262

C 12.879830 42.024342 34.604366

C 11.522566 39.088326 32.927345

O 12.295562 43.250084 35.018490

C 13.267061 44.014763 35.702499

H 10.930964 41.204895 34.380928

H 11.672178 41.732449 32.849113

H 13.787280 42.185688 33.991253

H 13.186388 41.413666 35.475151

H 12.039852 38.126606 32.802177

H 11.269790 39.476048 31.925531

H 14.096283 44.298832 35.032669

H 13.686073 43.477921 36.571815

H 12.782534 44.931503 36.062351

H 10.587247 38.906933 33.483746

O 12.935080 5.777662 25.305933

C 12.534158 4.418931 25.283934

C 13.743277 3.549128 25.099609

C 11.812989 6.619107 25.462879

O 13.293952 2.223644 24.892302

C 14.358348 1.314727 24.704554

H 11.834991 4.218960 24.450800

H 12.017850 4.129486 26.218763

H 14.397951 3.625401 25.987011

H 14.318255 3.921691 24.232250

H 12.180663 7.653679 25.467770

H 11.284947 6.426469 26.412380

H 15.017912 1.256545 25.587067

H 14.972931 1.567014 23.823505

H 13.905602 0.325626 24.542719

H 11.093776 6.509587 24.634636

O 28.915203 37.369743 1.954734

C 29.723400 37.175640 3.115938

C 30.784374 36.149868 2.837738

C 28.695183 38.734833 1.648176

O 31.691824 36.074574 3.921691

C 32.820759 35.315269 3.536416

H 29.078503 36.866146 3.953472

H 30.236774 38.101669 3.416629

H 31.298725 36.472073 1.915620

H 30.320871 35.166145 2.641678

H 28.015575 38.765144 0.785707

H 29.631481 39.253094 1.379755

H 33.411381 35.830601 2.759998

H 32.544514 34.315899 3.156030

H 33.454407 35.186192 4.424309

H 28.226789 39.263851 2.492066

O 42.227734 8.498057 28.191099

C 42.408150 9.774649 27.596563

C 41.118847 10.537865 27.485577

C 43.452496 7.793024 28.115805

O 41.430294 11.696136 26.732628

C 40.299404 12.487709 26.442204

H 42.808578 9.685663 26.568346

H 43.129318 10.386297 28.173008

H 40.722321 10.801886 28.482990

H 40.359539 9.914971 26.980024

H 43.315598 6.818590 28.604242

H 44.270473 8.325465 28.632111

H 39.746422 12.783022 27.350634

H 39.609524 11.969446 25.756725

H 40.664143 13.399072 25.946430

H 43.759544 7.614564 27.069500

O 3.740788 19.722866 4.480536

C 4.317723 20.724680 3.652292

C 5.668631 21.041016 4.207225

C 2.438284 19.362526 4.072281

O 6.309127 19.806963 4.499115

C 7.606253 20.012312 5.020312

H 3.683095 21.627729 3.621001

H 4.406219 20.366297 2.608431

H 5.567423 21.650709 5.126410

H 6.267079 21.641420 3.497302

H 2.069143 18.624245 4.798828

H 2.419705 18.900978 3.069979

H 7.607719 20.675787 5.902339

H 8.292707 20.439634 4.269808

H 7.993484 19.030544 5.325892

H 1.753785 20.226952 4.077170

O 11.055151 44.093971 22.258446

C 10.781351 43.062332 21.319706

C 10.651297 43.727764 19.992266

C 11.552879 43.586464 23.482235

O 9.677351 44.759403 20.090540

C 9.736512 45.598400 18.952314

H 9.857766 42.514732 21.588615

H 11.598839 42.321117 21.294281

H 10.393631 43.012463 19.188957

H 11.640886 44.155087 19.746334

H 12.500911 43.036911 23.349733

H 10.830244 42.917610 23.981918

H 9.563432 45.042492 18.014551

H 10.708012 46.114712 18.874086

H 8.953738 46.364552 19.056946

H 11.747473 44.451866 24.132019

O 7.374990 39.791405 30.258287

C 8.188566 39.703396 29.098061

C 9.471513 40.460747 29.358660

C 6.271480 38.910847 30.183969

O 9.634815 41.444469 28.347067

C 10.886471 42.092300 28.443384

H 7.675191 40.169346 28.239502

H 8.374847 38.649757 28.819370

H 10.350606 39.796783 29.406576

H 9.400129 40.933052 30.353138

H 5.701389 39.024277 31.115866

H 6.591238 37.856716 30.102318

H 11.727427 41.379932 28.499123

H 10.937320 42.752842 29.322969

H 11.005769 42.692215 27.530069

H 5.608004 39.145042 29.333237

O 20.440125 39.850075 14.398929

C 20.047026 40.463192 13.179543

C 20.997992 41.526123 12.684258

C 20.336960 40.691032 15.529819

O 20.871359 41.654709 11.274679

C 22.094656 42.097191 10.713390

H 19.008055 40.825977 13.221102

H 20.069515 39.653038 12.434417

H 22.010073 41.192673 12.951702

H 20.850822 42.503975 13.175632

H 20.899717 41.633686 15.416877

H 20.780418 40.144901 16.370287

H 22.926323 41.397045 10.911406

H 22.388012 43.094112 11.083998

H 21.950911 42.148525 9.627481

H 19.288698 40.914963 15.779174

O 33.815239 21.376421 42.121635

C 32.734219 20.466526 42.237999

C 33.270573 19.251051 42.940590

C 33.538506 22.488243 41.292416

O 32.293694 18.225769 42.993393

C 32.754265 17.175062 43.819683

H 32.331341 20.172680 41.254280

H 31.895708 20.907051 42.808090

H 33.594730 19.550764 43.953651

H 34.173622 18.910757 42.399345

H 34.367729 23.197678 41.428333

H 32.597805 22.998196 41.558880

H 32.932236 17.513889 44.854744

H 33.684692 16.722315 43.432453

H 31.970512 16.405489 43.844131

H 33.489124 22.208576 40.226551

O 29.864700 2.851428 45.990036

C 28.598866 2.607942 45.396473

C 27.756443 1.876017 46.392422

C 30.808331 3.218613 45.002399

O 26.517988 1.529367 45.801796

C 25.824200 0.685966 46.697514

H 28.683451 1.961579 44.503693

H 28.110428 3.546684 45.076717

H 27.611719 2.496466 47.295471

H 28.324087 0.975411 46.697514

H 31.803787 3.142340 45.457104

H 30.645517 4.251718 44.655746

H 25.646229 1.183695 47.665592

H 26.363487 48.632690 46.883305

H 24.856121 0.438080 46.249165

H 30.790731 2.539981 44.131618

O 30.116499 47.010914 41.442516

C 29.816298 46.355751 42.666790

C 30.678276 46.806053 43.802570

C 29.303411 46.442291 40.433369

O 30.382965 48.137894 44.204960

C 30.944252 48.354000 45.487415

H 29.928749 45.259087 42.560204

H 28.769012 46.546921 42.950859

H 31.745607 46.706802 43.537083

H 30.487595 46.106396 44.635704

H 29.531250 46.952732 39.488758

H 28.228258 46.573326 40.648006

H 32.038475 48.204388 45.494747

H 30.497862 47.691013 46.250141

H 30.730593 0.496749 45.776863

H 29.505825 45.364204 40.300869

O 35.192547 15.733214 36.902332

C 33.847019 15.718058 37.350677

C 32.959614 15.076095 36.320015

C 35.955765 16.662178 37.646477

O 31.613594 15.278510 36.730228

C 30.683653 15.017912 35.699566

H 33.466145 16.742361 37.508598

H 33.759010 15.186592 38.315331

H 33.193810 14.000942 36.207073

H 33.169853 15.556710 35.346561

H 37.006470 16.543856 37.352634

H 35.879002 16.490564 38.732876

H 30.727657 13.972585 35.354385

H 30.828377 15.685299 34.832699

H 29.685265 15.205172 36.117111

H 35.656540 17.699192 37.422058

O 12.156217 10.818998 20.179525

C 10.844423 11.033149 20.690943

C 10.501684 10.018623 21.733339

C 12.362056 11.705914 19.094105

O 10.248420 8.771368 21.106533

C 10.116898 7.710884 22.032074

H 10.090984 10.952965 19.887636

H 10.761794 12.053053 21.108488

H 9.604502 10.342294 22.290716

H 11.333351 9.957507 22.455975

H 13.394183 11.575859 18.744034

H 12.226133 12.760043 19.385506

H 9.422132 7.953392 22.852005

H 11.088398 7.425349 22.468197

H 9.714510 6.848415 21.482029

H 11.681468 11.489808 18.249725

O 33.351734 6.256322 3.339378

C 33.504768 5.148412 2.472509

C 34.967152 4.900525 2.257380

C 31.991537 6.452872 3.672827

O 35.141701 4.025833 1.157781

C 36.477940 3.579442 1.068308

H 33.010952 5.331759 1.506387

H 33.051044 4.235583 2.885164

H 35.393009 4.474669 3.182921

H 35.464394 5.868114 2.075988

H 31.949976 7.304584 4.365638

H 31.550522 5.575246 4.175934

H 36.769341 2.975616 1.945445

H 37.187862 4.416486 0.969544

H 36.545902 2.951658 0.168191

H 31.375977 6.688046 2.787868

O 29.941460 43.563972 2.472020

C 29.281898 42.435036 3.015219

C 27.909475 42.345074 2.410904

C 31.325615 43.566906 2.762443

O 27.204933 41.294861 3.043577

C 25.871626 41.236187 2.587896

H 29.197802 42.529892 4.112862

H 29.821674 41.494343 2.801558

H 28.000908 42.164661 1.323039

H 27.392679 43.313152 2.542914

H 31.827745 42.641853 2.431928

H 31.527054 43.711140 3.837596

H 25.804152 41.055771 1.502476

H 25.325005 42.160751 2.832360

H 25.386120 40.402077 3.112516

H 31.773476 44.401016 2.203598

O 46.264324 27.542292 1.204230

C 45.922073 28.862396 0.794019

C 45.348560 29.586987 1.987492

C 47.317963 26.996161 0.431723

O 45.048359 30.970655 1.790454

C 46.217384 31.753429 1.985537

H 45.197483 28.835506 48.856621

H 46.807034 29.412931 0.434657

H 46.062397 29.455957 2.816714

H 44.412262 29.093172 2.289650

H 47.529179 25.996792 0.834600

H 48.241547 27.599497 0.507996

H 47.018250 31.497719 1.272191

H 46.625153 31.644888 3.006418

H 45.943100 32.803646 1.824679

H 47.050030 26.899841 48.259148

O 23.626957 27.138439 27.892853

C 23.056377 26.423136 28.987564

C 22.063364 25.442347 28.459520

C 24.171133 28.385204 28.308441

O 22.664745 24.694284 27.409302

C 22.042830 23.441652 27.173639

H 22.564516 27.108126 29.698952

H 23.856264 25.905849 29.550320

H 21.181339 25.987011 28.078644

H 21.693247 24.786205 29.261362

H 23.403027 29.175310 28.304041

H 24.963196 28.657047 27.599983

H 20.943718 23.493969 27.210310

H 22.384102 22.687237 27.899698

H 22.339121 23.124828 26.164982

H 24.612637 28.341690 29.316610

O 41.612663 12.535135 44.493916

C 41.723160 12.033985 45.810108

C 43.187500 11.860904 46.113731

C 40.268108 12.653946 44.073437

O 43.354221 10.938786 47.182041

C 44.666019 11.030704 47.713017

H 41.221519 11.056129 45.900558

H 41.251343 12.715062 46.542522

H 43.633400 12.847073 46.343040

H 43.689140 11.482963 45.204327

H 40.280331 13.102293 43.069668

H 39.676998 13.313997 44.730556

H 44.864521 12.023717 48.155006

H 45.442924 10.829755 46.954197

H 44.754513 10.278244 48.510456

H 39.762070 11.674623 44.010365

O 13.686562 13.923203 22.466242

C 14.321190 14.970487 21.745562

C 13.908534 14.871723 20.303713

C 13.544284 14.218514 23.842573

O 14.229271 16.088177 19.658817

C 13.751590 16.128757 18.330399

H 15.419322 14.927461 21.863392

H 13.997031 15.963499 22.100035

H 12.817737 14.697175 20.288555

H 14.391596 14.008275 19.811363

H 12.954636 13.404449 24.283098

H 12.997173 15.161656 24.015654

H 12.650523 16.118490 18.283463

H 14.135885 15.296602 17.715816

H 14.113395 17.070431 17.897209

H 14.516761 14.273764 24.356438

O 14.823808 18.335289 32.257023

C 14.479113 17.760798 33.508678

C 13.927114 16.376154 33.315063

C 15.342072 19.638281 32.457973

O 13.380004 15.982079 34.557430

C 12.979571 14.630193 34.603878

H 13.707586 18.355335 34.031834

H 15.353317 17.710926 34.185844

H 14.727489 15.691166 32.981617

H 13.148252 16.393267 32.531803

H 15.560133 20.070005 31.470829

H 16.272991 19.626547 33.049576

H 13.835196 13.939338 34.510983

H 12.232001 14.385240 33.831375

H 12.517045 14.483026 35.587112

H 14.629215 20.305180 32.969883

O 15.693611 41.118847 17.184841

C 16.814234 41.102219 18.061489

C 16.708626 42.203285 19.081392

C 15.580179 39.864742 16.537991

O 17.953436 42.285915 19.757580

C 17.870806 42.799778 21.070841

H 17.755421 41.224941 17.497755

H 16.896862 40.144413 18.611044

H 15.878425 41.975445 19.771271

H 16.480295 43.161098 18.585131

H 14.693264 39.905815 15.891137

H 15.440835 39.036991 17.258181

H 17.470863 43.826038 21.096754

H 18.895599 42.805157 21.470783

H 17.250847 42.159283 21.719648

H 16.464161 39.638859 15.916562

O 18.720564 34.956886 19.833853

C 20.046537 35.346561 19.513117

C 21.002880 34.275318 19.947285

C 17.871296 36.086800 19.883722

O 22.304405 34.824875 19.912571

C 23.249504 34.027431 20.590714

H 20.336470 36.264278 20.053871

H 20.153612 35.561203 18.435518

H 20.929541 33.381557 19.303366

H 20.717834 33.977562 20.970610

H 16.856771 35.721569 20.080763

H 17.857607 36.647598 18.934715

H 23.303286 33.010464 20.172680

H 23.032421 33.947250 21.668310

H 24.228827 34.513428 20.471903

H 18.153896 36.781075 20.695345

O 5.991813 41.266991 12.121014

C 6.211341 40.581024 13.346267

C 6.794144 41.509987 14.357861

C 5.521953 40.339985 11.164671

O 8.044821 42.000381 13.895823

C 8.555751 42.973347 14.784694

H 6.902685 39.731754 13.200566

H 5.261843 40.161526 13.726164

H 6.913442 40.983902 15.324471

H 6.083242 42.339211 14.521650

H 5.350339 40.877804 10.223973

H 4.570988 39.874035 11.475140

H 8.709274 42.567539 15.799219

H 7.897654 43.857330 14.851676

H 9.528229 43.295551 14.387195

H 6.263657 39.545963 10.974478

O 43.707226 31.781298 31.393089

C 43.659313 31.150581 32.661858

C 44.773094 31.663956 33.531658

C 42.566071 31.429758 30.636717

O 44.994087 30.695389 34.540806

C 45.783218 31.183826 35.603737

H 43.786438 30.057825 32.563095

H 42.688301 31.311438 33.168388

H 44.507603 32.649632 33.955070

H 45.680542 31.803787 32.919033

H 42.662388 31.912331 29.654461

H 41.630753 31.783743 31.108532

H 45.333893 32.072208 36.080929

H 46.806541 31.431225 35.277134

H 45.836021 30.381006 36.352776

H 42.487843 30.339449 30.484171

O 0.784239 29.584055 26.790812

C 0.693787 30.914919 27.273382

C 48.694298 30.985323 28.479568

C 1.665289 29.527338 25.683876

O 48.511436 32.354813 28.797857

C 47.642120 32.564560 29.894524

H 0.263535 31.582792 26.505278

H 1.688270 31.315350 27.546202

H 0.254734 30.437723 29.323946

H 47.741375 30.484659 28.239992

H 1.677511 28.489836 25.321091

H 2.696929 29.808472 25.960121

H 47.940369 31.995447 30.789263

H 46.594837 32.308361 29.660326

H 47.690525 33.634335 30.139477

H 1.335263 30.177124 24.853676

O 9.856789 20.841534 45.969986

C 9.042234 22.006649 45.925987

C 9.442666 23.026552 44.893860

C 11.171515 21.114355 46.411488

O 8.325465 23.858709 44.644505

C 8.669671 24.889856 43.741455

H 8.950805 22.484819 46.918018

H 8.038465 21.653154 45.645828

H 9.747269 22.488733 43.980049

H 10.311980 23.615223 45.240509

H 11.744050 20.184414 46.311260

H 11.201829 21.428247 47.465130

H 8.973784 24.494802 42.758709

H 9.492537 25.519108 44.120373

H 7.779333 25.519596 43.609932

H 11.666800 21.885883 45.800819

O 47.229954 23.756033 26.721872

C 47.385433 23.386404 28.086470

C 46.129379 23.748211 28.826706

C 48.139851 23.059799 25.895094

O 46.061905 23.017263 30.044134

C 44.827850 23.319910 30.673876

H 48.276260 23.863598 28.534327

H 47.522823 22.297562 28.198435

H 45.283535 23.487612 28.165676

H 46.076088 24.839008 29.006632

H 47.980949 23.408895 24.868834

H 47.970680 21.969980 25.913671

H 43.977119 22.842716 30.158545

H 44.649883 24.405329 30.708590

H 44.860119 22.952724 31.709423

H 0.296780 23.255861 26.165470

O 2.050564 23.022152 5.583069

C 0.685477 22.700928 5.778640

C 0.593559 21.717205 6.908552

C 2.213866 23.946226 4.525517

O 48.177010 21.178894 6.939355

C 48.038643 20.270466 8.009619

H 0.252776 22.229601 4.877546

H 0.079695 23.598598 6.004036

H 0.854157 22.213465 7.862451

H 1.346508 20.926119 6.734494

H 3.295375 24.066015 4.369061

H 1.781165 24.934351 4.763625

H 48.127140 20.770639 8.990408

H 48.776432 19.450533 7.964637

H 47.033894 19.836298 7.926501

H 1.769919 23.588820 3.579442

O 18.229681 6.082264 9.712066

C 19.421686 6.811745 10.020091

C 19.651972 8.111805 9.276920

C 18.412540 5.382608 8.490724

O 21.002392 8.490724 9.498405

C 21.292814 9.712555 8.848619

H 20.323271 6.190806 9.878302

H 19.371326 7.021984 11.095732

H 18.948895 8.892622 9.618192

H 19.475958 7.978817 8.196389

H 17.471842 4.884880 8.230614

H 19.200691 4.614014 8.572864

H 20.749615 10.561823 9.295988

H 21.048840 9.678819 7.775422

H 22.369923 9.903236 8.954227

H 18.654558 6.051462 7.649279

O 25.674587 47.665592 36.327839

C 25.981634 48.587708 37.361919

C 26.876860 0.761263 36.786942

C 24.665440 46.736629 36.670090

O 26.087730 1.862326 36.358150

C 26.888105 2.827959 35.704945

H 26.455406 48.058201 38.209236

H 25.072716 0.181394 37.756973

H 27.422504 0.315845 35.936699

H 27.638609 1.101553 37.510067

H 24.618992 46.015457 35.842823

H 23.677805 47.213818 36.772762

H 27.393169 2.416772 34.812649

H 27.650835 3.260170 36.376244

H 26.221209 3.638111 35.380787

H 24.883501 46.190495 37.601986

O 37.276848 37.852318 8.857908

C 36.743916 36.581104 9.182557

C 35.250240 36.631462 8.978185

C 38.662468 37.946190 9.117529

O 34.634190 35.552399 9.678329

C 33.517971 35.899048 10.481638

H 36.920910 36.339085 10.245485

H 37.203018 35.767525 8.589487

H 34.972042 36.569859 7.913789

H 34.902611 37.607853 9.347815

H 38.964138 38.972450 8.860353

H 39.246738 37.241646 8.501480

H 32.613941 36.102932 9.890524

H 33.719410 36.769829 11.124578

H 33.316044 35.030224 11.120178

H 38.904976 37.767731 10.179970

O 4.389596 13.846931 39.064369

C 4.754825 12.688170 39.794338

C 5.754683 11.931799 38.962669

C 3.292441 14.524584 39.643749

O 6.123823 10.710457 39.592899

C 6.721782 9.834786 38.654160

H 3.881599 12.037407 39.976707

H 5.179703 12.950235 40.779530

H 6.640620 12.567405 38.776390

H 5.273088 11.755296 37.983841

H 3.155541 15.459414 39.081482

H 3.467477 14.781271 40.701298

H 7.681059 10.227884 38.272793

H 6.058307 9.641660 37.795113

H 6.905130 8.875998 39.161667

H 2.361522 13.935426 39.576275

O 19.663218 33.490589 26.649998

C 19.442223 32.735683 27.825380

C 20.082718 31.392111 27.627853

C 19.186512 34.810204 26.794231

O 19.514093 30.465591 28.535305

C 20.116941 29.199759 28.360268

H 18.361691 32.587048 28.001884

H 19.848032 33.244171 28.717186

H 21.179871 31.467896 27.749598

H 19.897902 31.082621 26.583014

H 19.252029 35.282509 25.806597

H 19.789360 35.393009 27.512466

H 21.200407 29.226648 28.565617

H 19.960485 28.797857 27.343298

H 19.649038 28.516724 29.077036

H 18.135805 34.837097 27.115458

O 36.675957 17.224932 44.975506

C 35.839401 17.290449 46.118134

C 35.108940 15.984522 46.259434

C 37.081276 18.521570 44.585342

O 33.915958 16.236809 46.978157

C 33.029041 15.138188 46.968868

H 35.066406 18.071756 45.999813

H 36.420246 17.542248 47.024117

H 35.724014 15.209083 46.750317

H 34.889416 15.633962 45.236107

H 37.702217 18.416451 43.684738

H 37.676304 19.019789 45.369095

H 33.449520 14.261540 47.490067

H 32.736664 14.843365 45.946030

H 32.120613 15.456481 47.498867

H 36.222717 19.169399 44.334034

O 30.060270 35.752857 43.183098

C 30.362917 37.121368 43.360580

C 30.329182 37.463127 44.819538

C 29.998177 35.441902 41.804810

O 30.369762 38.873688 44.922215

C 30.516439 39.318611 46.254055

H 29.619257 37.759911 42.858452

H 31.351528 37.375122 42.938145

H 31.177471 36.982513 45.339760

H 29.396305 37.061234 45.254684

H 29.704332 34.388260 41.723648

H 30.973101 35.570980 41.303169

H 31.462029 38.966583 46.698002

H 29.682329 38.995430 46.899929

H 30.532576 40.418209 46.226189

H 29.241804 36.044750 41.271877

O 48.055756 40.798595 29.183134

C 47.049053 40.507195 28.226789

C 46.296593 41.730495 27.787245

C 48.549572 39.566498 29.675974

O 45.671741 41.395088 26.558081

C 44.546719 42.201820 26.272057

H 46.318596 39.781136 28.623312

H 47.494953 40.056404 27.324230

H 46.986958 42.575851 27.637632

H 45.563202 42.024826 28.559261

H 0.534395 39.781136 30.297890

H 48.861996 38.890800 28.861908

H 44.805851 43.261814 26.131247

H 43.786438 42.144615 27.068520

H 44.107170 41.818012 25.340649

H 47.798088 39.044811 30.296423

O 10.728547 20.362383 0.937764

C 9.431421 19.928217 1.349441

C 8.597799 19.685707 0.142278

C 10.997458 21.718670 1.259479

O 9.187935 18.642824 48.281151

C 8.384626 18.353868 47.152214

H 8.957161 20.648407 2.031496

H 9.559031 18.989964 1.909753

H 8.604644 20.613205 48.441032

H 7.555893 19.450533 0.415100

H 10.281178 22.405615 0.780329

H 12.006116 21.941622 0.889360

H 8.104470 19.260830 46.593372

H 7.464952 17.815557 47.433350

H 8.965472 17.712395 46.478474

H 10.980345 21.902996 2.345388

O 2.162529 37.619099 28.794437

C 1.884328 36.651508 29.793316

C 2.615765 37.079811 31.036171

C 1.946911 37.121368 27.488998

O 2.619676 36.055016 32.030651

C 3.876710 36.031059 32.697548

H 2.264225 35.655075 29.511206

H 0.795975 36.552258 29.954662

H 2.186486 38.008774 31.449316

H 3.640069 37.323784 30.702234

H 2.071099 37.965260 26.797167

H 0.935808 36.701870 27.366766

H 4.114818 36.995224 33.177673

H 4.705932 35.781708 32.011093

H 3.835640 35.259041 33.477390

H 2.678348 36.342995 27.213732

O 45.154945 18.535259 9.153710

C 44.703175 17.259647 8.711719

C 43.236881 17.293871 8.381693

C 46.253078 18.388092 10.044537

O 42.996819 17.705061 7.039585

C 41.695782 17.268448 6.674845

H 44.874786 16.491541 9.487159

H 45.276688 16.942822 7.824804

H 42.725952 17.977394 9.079882

H 42.809559 16.292059 8.557218

H 46.540565 19.387951 10.396565

H 47.129238 17.939745 9.552675

H 40.916916 17.697237 7.329031

H 41.599461 16.168360 6.719826

H 41.500698 17.582829 5.640762

H 46.000301 17.772043 10.927052

O 5.812376 23.752121 40.821575

C 5.766417 24.978842 41.534431

C 4.573432 25.746458 41.033283

C 6.923220 22.968370 41.204407

O 4.127041 26.649021 42.027760

C 2.869030 27.185375 41.671333

H 6.703691 25.557243 41.424911

H 5.616805 24.793537 42.614964

H 3.789192 25.003286 40.814243

H 4.794917 26.272057 40.088184

H 6.835702 22.010073 40.676853

H 6.943756 22.766443 42.290318

H 2.095546 26.404068 41.573547

H 2.916944 27.750574 40.725258

H 2.567361 27.869875 42.476109

H 7.877608 23.437254 40.908607

O 43.814793 8.039443 47.218708

C 45.078671 7.449307 46.954689

C 45.663921 7.914278 45.651695

C 43.286751 7.456641 48.397514

O 46.935131 7.301651 45.555378

C 47.607407 7.570561 44.344791

H 45.003864 6.349219 46.902863

H 45.802284 7.680570 47.757999

H 45.743126 9.015343 45.633118

H 45.013645 7.613098 44.813675

H 42.324051 7.939702 48.614601

H 43.950226 7.603319 0.375010

H 47.784889 8.649137 44.200558

H 47.066162 7.178930 43.468143

H 48.578419 7.057676 44.407372

H 43.103893 6.373176 48.281151

O 19.360081 14.292344 28.589575

C 19.531206 13.753056 29.894524

C 18.168076 13.293952 30.344339

O 17.266981 13.626912 29.295099

C 18.189589 13.600021 28.233147

H 20.263620 12.927257 29.852966

H 19.988842 14.531918 30.523285

H 17.802357 13.763812 31.268412

H 18.128962 12.203643 30.521330

H 17.741730 14.080148 27.351120

H 18.425739 12.554205 27.944191

O 9.074992 0.810154 16.575148

C 9.460756 1.292237 15.294645

C 8.778703 0.416567 14.279631

O 7.924057 48.445919 15.013512

C 8.680917 48.404850 16.199162

H 9.191357 2.356633 15.238907

H 10.558400 1.248722 15.215439

H 9.494982 48.701630 13.698296

H 8.177810 0.952921 13.534993

H 9.566365 47.745773 16.076441

H 8.070246 47.971169 17.003448

O 39.048721 38.960228 2.932590

C 39.224251 38.572506 1.580215

C 40.583958 37.931522 1.516655

O 41.056751 37.833248 2.856806

C 39.817318 37.938370 3.517348

H 38.414585 37.874805 1.296148

H 39.097126 39.459911 0.943631

H 41.326149 38.501125 0.943142

H 40.556580 36.924332 1.069775

H 39.986977 38.174522 4.577833

H 39.274609 36.968334 3.483612

O 32.760620 41.465004 7.297250

C 32.858894 41.975445 8.632513

C 32.739109 43.455433 8.518104

O 33.411873 43.730209 7.301162

C 32.815380 42.678524 6.567770

H 32.110832 41.486519 9.271052

H 33.852886 41.704090 9.028055

H 33.206524 44.019653 9.335102

H 31.685469 43.796215 8.462854

H 33.381069 42.513756 5.640273

H 31.787653 42.963570 6.260234

O 14.139308 42.410591 12.905744

C 12.911610 41.893307 12.387480

C 12.734618 42.489799 11.022392

O 13.579486 43.628021 11.008214

C 14.629704 43.048641 11.745517

H 12.954147 40.793217 12.389435

H 12.107324 42.172485 13.085180

H 11.710803 42.804176 10.781840

H 13.036286 41.792099 10.217617

H 15.347449 43.828484 12.035452

H 15.192948 42.329918 11.114800

O 38.761723 17.114435 35.483952

C 37.878719 18.142162 35.032669

C 38.072823 18.251682 33.563438

O 38.405293 16.932554 33.175232

C 39.283897 16.674400 34.248428

H 36.845615 17.834625 35.261486

H 38.079670 19.061346 35.593468

H 38.885910 18.952314 33.289150

H 37.175148 18.572420 33.020729

H 40.259308 17.170172 34.067036

H 39.481426 15.595336 34.309544

O 37.808311 20.650362 38.898621

C 38.152519 21.132446 37.599052

C 38.001438 22.624655 37.620564

O 37.912945 22.961037 38.993473

C 37.215244 21.816456 39.417862

H 39.174377 20.795574 37.380989

H 37.508598 20.644985 36.857349

H 37.088123 22.960548 37.093498

H 38.836037 23.187899 37.177109

H 36.148891 21.872194 39.118641

H 37.232357 21.772451 40.515507

O 33.071579 47.534557 23.664114

C 32.686790 48.889378 23.852352

C 31.499674 48.867374 24.773005

O 31.371576 47.528202 25.221352

C 31.877617 46.908241 24.064058

H 33.560509 0.538799 24.237139

H 32.465305 0.450303 22.874006

H 30.567778 0.258643 24.255718

H 31.578880 0.631206 25.649651

H 31.125156 46.929752 23.246082

H 32.088345 45.850689 24.278210

O 20.858156 20.770639 47.478821

C 20.265087 21.681513 46.558659

C 19.658327 22.783066 47.383476

O 20.007912 22.495089 48.728031

C 20.075384 21.097242 48.602863

H 21.042482 22.002249 45.850201

H 19.504805 21.149559 45.959721

H 18.556772 22.809958 47.289116

H 20.003511 23.797592 47.144394

H 19.056946 20.653296 48.528549

H 20.538887 20.673342 0.611646

O 47.607899 16.271524 48.080200

C 48.033264 15.052627 47.484688

C 0.476705 14.731400 48.087044

O 0.780328 15.793352 0.084583

C 48.359379 16.191828 0.375010

H 47.286182 14.264475 47.678791

H 48.047932 15.192460 46.394867

H 1.290768 14.652194 47.353165

H 0.474257 13.782880 48.651272

H 48.380402 17.179951 0.857089

H 47.891964 15.487771 1.096663

O 32.546467 25.384653 0.828734

C 32.757687 25.543554 48.324177

C 31.486473 25.101564 47.657280

O 30.678766 24.540762 48.680607

C 31.138845 25.357273 0.834113

H 33.003616 26.598661 48.112957

H 33.647533 24.959286 48.047443

H 31.602348 24.350569 46.864727

H 30.949633 25.954252 47.203552

H 30.791218 24.945595 1.789966

H 30.717390 26.382555 0.754904

O 24.879101 40.137077 6.557991

C 23.821548 40.581512 7.414104

C 23.988274 42.051720 7.565672

O 24.486490 42.436016 6.301793

C 25.420343 41.388245 6.191295

H 22.880852 40.345360 6.901707

H 23.817638 40.015335 8.352845

H 24.708466 42.317696 8.366536

H 23.065178 42.606163 7.769065

H 26.296993 41.600925 6.824945

H 25.791439 41.334461 5.162591

O 17.768621 42.083988 0.955365

C 18.896090 41.289478 1.329884

C 18.360714 40.015823 1.907308

O 17.014206 40.303314 2.243202

C 16.730627 41.152092 1.158759

H 19.550764 41.149647 0.458126

H 19.481825 41.869350 2.057409

H 18.882401 39.665260 2.809380

H 18.392982 39.176334 1.189073

H 15.798731 41.696270 1.366065

H 16.555590 40.562935 0.237619

O 18.338223 38.821369 39.855453

C 19.290653 38.726032 38.809147

C 19.347370 40.088673 38.179409

O 18.603222 40.951141 39.033077

C 18.603710 40.157612 40.195259

H 20.269976 38.429253 39.225716

H 18.993385 37.908054 38.141762

H 18.907824 40.148811 37.173195

H 20.378519 40.470528 38.086514

H 17.825825 40.521374 40.880737

H 19.572277 40.245129 40.735523

O 37.460197 39.211048 21.833569

C 36.887661 40.468082 21.471273

C 36.382599 41.099777 22.728306

O 37.107681 40.467590 23.765812

C 37.126259 39.175842 23.203545

H 37.672878 41.062130 20.981367

H 36.106354 40.303799 20.718813

H 35.299622 40.930607 22.877918

H 36.543945 42.185688 22.787466

H 36.142048 38.682518 23.340445

H 37.862583 38.559795 23.736965

O 2.501844 34.006409 10.603381

C 1.136758 34.401463 10.576490

C 0.431723 33.401604 9.698864

O 1.417891 32.465797 9.280342

C 2.319963 32.636433 10.344738

H 0.735348 34.410755 11.605195

H 1.092265 35.445324 10.232285

H 48.859550 33.826973 8.798748

H 48.522678 32.860851 10.232285

H 3.288530 32.194439 10.069962

H 1.964513 32.103992 11.253655

O 21.610617 39.540096 25.470215

C 20.196148 39.429108 25.574844

C 19.925283 38.176476 26.356642

O 21.183784 37.553581 26.563946

C 21.976336 38.716251 26.551235

H 19.796206 40.328247 26.074043

H 19.782026 39.433022 24.555429

H 19.263763 37.460686 25.854023

H 19.454933 38.385250 27.333031

H 23.034376 38.434143 26.443670

H 21.881483 39.265316 27.512957

O 7.925523 44.201534 35.378830

C 9.293055 43.937023 35.653606

C 9.765359 45.043468 36.557144

O 8.644247 45.894691 36.752716

C 7.969038 45.596935 35.555332

H 9.359549 42.928364 36.076530

H 9.854344 43.901821 34.705574

H 10.586268 45.631161 36.108799

H 10.128633 44.715885 37.542336

H 8.453075 46.099064 34.688953

H 6.943266 45.984653 35.613514

O 46.301968 40.122898 2.433884

C 46.837837 40.970211 1.423758

C 46.317616 40.456837 0.109520

O 45.668808 39.229626 0.397498

C 45.247353 39.541565 1.701469

H 46.528835 42.008694 1.629108

H 47.933521 40.950165 1.518610

H 47.081810 40.267132 48.235191

H 45.589115 41.152580 48.548107

H 44.919769 38.622379 2.208977

H 44.371681 40.223618 1.670178

O 7.783733 45.275711 42.197910

C 8.720520 44.969639 41.180939

C 7.890320 44.555519 40.003113

O 6.563369 45.037113 40.233395

C 6.790721 45.833088 41.374065

H 9.348304 45.855579 40.977055

H 9.409908 44.200558 41.553501

H 7.833604 43.463253 39.886257

H 8.252616 44.948128 39.043346

H 5.854424 45.919140 41.943665

H 7.074789 46.860813 41.073376

O 31.733871 3.386315 35.851624

C 30.979944 4.513294 35.438965

C 30.482214 5.130810 36.706760

O 30.528175 4.106995 37.699280

C 30.857712 3.004462 36.885704

H 31.608706 5.164546 34.815094

H 30.134586 4.181801 34.806782

H 29.452044 5.511196 36.630486

H 31.108532 5.966388 37.054386

H 29.935104 2.543892 36.469627

H 31.340282 2.237334 37.505180

O 0.839978 28.038553 11.949400

C 0.362785 28.911779 12.958548

C 0.757838 28.258574 14.241982

O 0.844379 26.859747 13.970628

C 0.489417 26.847034 12.608963

H 0.774951 29.916527 12.793290

H 48.156963 29.000275 12.880320

H 0.053782 28.422361 15.070716

H 1.745962 28.588598 14.592055

H 48.294842 26.661243 12.502866

H 1.008658 26.010481 12.130793

O 3.341334 3.262616 31.678133

C 3.686028 1.917576 31.983715

C 3.540328 1.782632 33.475922

O 3.223991 3.083669 33.948227

C 2.538514 3.517348 32.803158

H 3.016686 1.243833 31.420958

H 4.698109 1.724938 31.600883

H 4.434577 1.434515 34.012276

H 2.726263 1.086887 33.753632

H 2.344898 4.597390 32.876499

H 1.547457 3.019130 32.716618

O 7.349566 24.308523 31.758806

C 6.239699 23.431385 31.688890

C 6.468029 22.587008 30.467548

O 7.818937 22.800179 30.062716

C 8.314220 23.439697 31.217564

H 6.188850 22.833426 32.617851

H 5.323448 24.036188 31.659065

H 5.821177 22.857874 29.619257

H 6.312060 21.510876 30.644541

H 9.209448 24.020054 30.950609

H 8.626645 22.687237 31.971979

O 29.754204 10.726103 12.188486

C 30.395676 11.849659 12.793778

C 31.127113 12.490154 11.676579

O 31.735826 11.375399 11.050750

C 30.631340 10.498261 11.095243

H 29.642727 12.458862 13.310087

H 31.116846 11.494698 13.553084

H 31.901085 13.209856 11.966513

H 30.443104 12.999128 10.970567

H 31.015636 9.470046 11.136312

H 30.048048 10.591647 10.162368

O 3.425918 27.919256 30.308645

C 2.372768 28.669270 29.731710

C 1.166582 27.822449 29.957108

O 1.619329 26.466652 29.943417

C 3.014241 26.659288 29.831940

H 2.333164 29.659840 30.205482

H 2.574206 28.837463 28.656069

H 0.368652 27.938324 29.211004

H 0.716768 28.021931 30.943764

H 3.317865 26.544388 28.773413

H 3.528104 25.868690 30.395676

O 23.166386 30.870913 37.181995

C 23.395205 32.270226 37.198620

C 23.623045 32.677990 35.768993

O 23.361467 31.524612 34.978886

C 22.584562 30.806374 35.902470

H 22.521980 32.780666 37.644524

H 24.235672 32.459930 37.874321

H 24.644903 33.015842 35.539688

H 22.953703 33.495480 35.451191

H 22.537624 29.758604 35.580269

H 21.540701 31.190672 35.921051

O 40.950653 4.819363 20.931007

C 40.586891 3.849330 19.961464

C 41.819965 3.018152 19.722866

O 42.781200 3.453298 20.676277

C 41.893795 4.023878 21.606216

H 39.753269 3.241104 20.356518

H 40.186947 4.371994 19.082859

H 42.266357 3.120338 18.723497

H 41.635643 1.939577 19.864166

H 42.459976 4.649216 22.309296

H 41.392643 3.235236 22.210043

O 44.488533 12.900853 34.500717

C 45.048359 11.682935 34.960796

C 44.673840 11.584171 36.413891

O 43.761990 12.646123 36.669109

C 43.364491 12.912100 35.345093

H 44.640106 10.842957 34.367729

H 46.126934 11.707380 34.755936

H 45.518219 11.699558 37.109146

H 44.188332 10.625872 36.665688

H 42.880451 13.897778 35.307446

H 42.610565 12.179686 34.992577

O 4.038545 20.844467 32.863785

C 4.661439 20.216196 33.973648

C 3.642514 20.220106 35.079605

O 2.527758 20.957897 34.597523

C 3.185855 21.693735 33.594242

H 5.009067 19.221714 33.658779

H 5.565467 20.788240 34.249405

H 4.021433 20.705122 35.994392

H 3.284618 19.225138 35.382744

H 3.751055 22.545937 34.032810

H 2.438773 22.123991 32.913654

O 37.247025 11.078619 48.767632

C 37.281738 12.341031 48.128117

C 38.403339 12.230045 47.147324

O 39.293674 11.229698 47.647011

C 38.640957 10.900161 48.854664

H 36.296547 12.548337 47.688568

H 37.466064 13.136028 48.875687

H 38.955338 13.168787 46.995758

H 38.065975 11.897085 46.155293

H 39.049702 11.517676 0.787660

H 38.860485 9.851899 0.207307

O 47.753597 41.624886 21.028305

C 0.207305 41.305126 21.349041

C 1.051195 42.308407 20.607826

O 0.137389 43.126873 19.887146

C 47.998550 42.179817 19.760025

H 0.312914 41.318813 22.443750

H 0.415100 40.265663 21.035149

H 1.750362 41.833168 19.894970

H 1.668711 42.958191 21.245876

H 48.257683 41.393135 19.013920

H 47.087677 42.675102 19.393328

O 20.182949 36.420246 3.496324

C 19.708199 35.520130 2.509179

C 18.414495 36.109776 2.032963

O 17.986683 37.024075 3.042599

C 18.928358 36.734627 4.050768

H 20.477772 35.401810 1.735694

H 19.572765 34.517830 2.958014

H 17.624388 35.364162 1.858904

H 18.510324 36.676445 1.094710

H 18.565084 35.900028 4.686375

H 19.032011 37.612740 4.702021

O 24.934351 26.826992 6.897307

C 24.147177 27.989662 7.150572

C 24.712376 29.099529 6.302771

O 25.911716 28.580774 5.755171

C 25.523508 27.231823 5.683788

H 23.100380 27.744707 6.925665

H 24.180912 28.195990 8.230124

H 24.954885 30.025557 6.845481

H 24.044012 29.397774 5.475016

H 26.412380 26.613817 5.489683

H 24.822386 27.069986 4.836476

O 2.816225 17.248890 11.633063

C 4.026811 17.972015 11.486385

C 3.620512 19.343458 11.024348

O 2.252491 19.254961 10.628806

C 2.116081 17.854183 10.573068

H 4.569032 17.937300 12.442240

H 4.674152 17.460108 10.749083

H 4.217493 19.710644 10.173614

H 3.690428 20.114498 11.806144

H 2.475442 17.467442 9.594723

H 1.049728 17.595051 10.646895

O 6.480741 44.003033 7.931390

C 6.146314 44.423996 9.246606

C 4.868256 43.713097 9.604502

O 4.530896 42.913700 8.478500

C 5.824111 42.760666 7.945080

H 6.974557 44.165844 9.929639

H 6.085197 45.522129 9.243184

H 4.012632 44.367771 9.820119

H 4.981687 43.067711 10.492884

H 5.748326 42.382725 6.915886

H 6.407890 42.011627 8.521526

O 15.192460 28.828661 35.523552

C 16.328239 29.491648 36.055996

C 16.315527 30.872868 35.460480

O 15.256509 30.895361 34.508049

C 15.115210 29.511696 34.297321

H 16.260767 29.455467 37.153149

H 17.237156 28.924980 35.783661

H 17.260624 31.127113 34.952972

H 16.132668 31.682045 36.182629

H 15.885760 29.139130 33.589352

H 14.135398 29.318569 33.837238

O 24.186289 2.774666 23.112116

C 23.403027 1.596350 23.052465

C 24.313412 0.532443 23.558996

O 25.237484 1.172938 24.436132

C 24.785227 2.507223 24.356926

H 22.497532 1.708314 23.673405

H 23.054420 1.440382 22.024250

H 24.890347 0.087029 22.736130

H 23.818617 48.602863 24.093884

H 25.640850 3.179988 24.493336

H 24.081169 2.722351 25.186636

O 3.494857 13.176121 9.692997

C 3.836129 14.491337 10.109564

C 4.912260 14.343681 11.153425

O 5.109787 12.947302 11.318683

C 4.712288 12.554205 10.026936

H 4.179846 15.068761 9.232916

H 2.916456 14.979776 10.460614

H 4.672196 14.774916 12.136660

H 5.866158 14.807673 10.840022

H 4.589078 11.461939 9.997600

H 5.494084 12.803557 9.280342

O 42.870186 40.562935 3.730031

C 43.806969 41.440071 4.329947

C 43.751720 42.706882 3.517348

O 42.655056 42.567539 2.621632

C 41.962734 41.553013 3.313465

H 43.528282 41.600437 5.388964

H 44.783360 40.938431 4.351459

H 44.652817 42.900497 2.917434

H 43.596733 43.608463 4.132908

H 41.210762 41.098797 2.653901

H 41.408291 41.977402 4.174468

O 20.805353 16.910551 18.984097

C 21.380821 15.763527 18.355824

C 21.060574 15.854468 16.908108

O 21.027815 17.247913 16.666088

C 20.354073 17.617054 17.847828

H 22.472597 15.816331 18.494190

H 21.012657 14.858521 18.857464

H 20.085651 15.396832 16.657288

H 21.814011 15.389008 16.256857

H 19.268652 17.446417 17.719240

H 20.489994 18.693674 18.020418

O 5.403632 3.166786 25.654051

C 4.337769 2.760488 24.807228

C 4.960175 1.958157 23.702251

O 6.343841 1.876017 24.011255

C 6.242633 2.064254 25.399321

H 3.636157 2.157639 25.405676

H 3.781369 3.649847 24.476713

H 4.854566 2.401125 22.700928

H 4.538719 0.938742 23.640158

H 7.242002 2.260803 25.809532

H 5.866647 1.144580 25.901939

O 1.049240 47.526737 43.028599

C 0.760283 46.389000 42.226269

C 1.911219 46.235966 41.266010

O 2.905210 47.153194 41.697735

C 2.020251 48.123226 42.206711

H 0.618494 45.533375 42.903431

H 48.685493 46.549858 41.723160

H 1.624219 46.481407 40.226551

H 2.357611 45.232685 41.228367

H 1.545990 48.688915 41.378952

H 2.584473 48.849285 42.804668

O 6.350686 36.049149 22.773287

C 5.125432 35.706413 22.145994

C 4.111395 35.577335 23.248526

O 4.836476 35.646763 24.471823

C 6.124312 35.362694 23.978495

H 4.904437 36.468163 21.387667

H 5.249131 34.757401 21.595461

H 3.562330 34.620991 23.208435

H 3.347201 36.368908 23.263683

H 6.254856 34.269939 23.834263

H 6.871394 35.679520 24.719711

O 3.202967 28.121670 21.103109

C 3.562818 27.289026 22.195864

C 4.488848 26.237831 21.643375

O 4.589078 26.483765 20.248953

C 4.301589 27.861073 20.262154

H 4.035123 27.908501 22.977171

H 2.634833 26.899351 22.639322

H 4.140731 25.204727 21.775875

H 5.498484 26.279881 22.088301

H 4.061525 28.183277 19.240294

H 5.188015 28.448275 20.575068

O 46.303436 41.996471 14.047878

C 46.573814 43.030064 13.112559

C 46.291214 44.327190 13.818084

O 45.912296 43.975163 15.140634

C 45.394028 42.700527 14.857544

H 47.610344 42.914677 12.767865

H 45.935272 42.885342 12.224668

H 45.472260 44.895325 13.341378

H 47.146351 45.015602 13.876755

H 44.397594 42.778263 14.368127

H 45.245396 42.150482 15.797753

O 16.825968 4.533340 24.728512

C 17.563761 3.474811 24.128109

C 17.883520 2.494511 25.225262

O 17.168217 2.948236 26.363487

C 17.235689 4.320168 26.057417

H 16.962868 3.068023 23.302797

H 18.469255 3.895289 23.667048

H 18.963562 2.476909 25.463369

H 17.602386 1.451627 25.022358

H 18.257549 4.722066 26.234409

H 16.564880 4.870701 26.725782

O 36.310726 1.727872 11.288858

C 35.657028 2.415793 10.240108

C 35.592491 1.428648 9.118996

O 36.622173 0.464971 9.351236

C 37.231865 1.014037 10.498261

H 34.678684 2.763421 10.602404

H 36.234455 3.319821 9.971198

H 35.743572 1.867705 8.124516

H 34.629795 0.895716 9.086726

H 38.070866 1.675067 10.198060

H 37.662121 0.198016 11.096221

O 21.230721 37.695370 41.397533

C 20.865002 38.095314 42.706394

C 19.462267 37.586830 42.907833

O 19.122463 36.842194 41.745651

C 20.432301 36.540031 41.333973

H 21.577858 37.658699 43.430496

H 20.986744 39.185623 42.781200

H 18.703941 38.371067 43.036911

H 19.382084 36.925800 43.786922

H 20.414700 36.175293 40.298424

H 20.859625 35.728413 41.958332

O 44.560898 7.873696 15.542531

C 44.271938 8.770391 16.609863

C 45.569069 9.434354 16.976068

O 46.489227 9.063747 15.963011

C 45.958252 7.787156 15.696055

H 43.494545 9.464668 16.261257

H 43.825062 8.205679 17.443485

H 45.948475 9.094061 17.957348

H 45.530933 10.531998 17.027895

H 46.220806 7.072832 16.505720

H 46.401222 7.393569 14.769048

O 5.311714 36.370865 8.886267

C 5.456436 37.051453 10.116898

C 5.130810 36.018345 11.140224

O 5.464748 34.749580 10.564755

C 5.908695 35.164192 9.289143

H 6.491008 37.432327 10.219573

H 4.786605 37.921745 10.117876

H 4.053702 36.012478 11.373443

H 5.662764 36.139111 12.094612

H 5.685255 34.378971 8.554284

H 7.006338 35.289845 9.288654

O 25.890203 32.562603 24.402395

C 26.102398 31.674713 25.491728

C 24.806740 30.941319 25.703432

O 23.947205 31.327572 24.641481

C 24.484535 32.612965 24.429775

H 26.953135 31.026394 25.234552

H 26.412868 32.258003 26.373264

H 24.322701 31.195072 26.662220

H 24.905502 29.848076 25.694632

H 24.134953 33.317020 25.215485

H 24.125664 33.000683 23.466587

O 16.032438 44.276829 23.692961

C 15.488261 42.991928 23.979473

C 16.629417 42.145103 24.484045

O 17.799423 42.928856 24.315367

C 17.183865 44.177578 24.495293

H 14.683485 43.102425 24.727533

H 15.004222 42.618385 23.065178

H 16.778542 41.188271 23.965784

H 16.517942 41.896729 25.554798

H 17.889387 44.968174 24.213181

H 16.929132 44.332077 25.565554

O 12.588918 36.221741 36.731205

C 13.088114 35.110409 37.456284

C 12.506289 35.237530 38.832127

O 11.440915 36.179691 38.737762

C 11.320639 36.237385 37.337967

H 12.766888 34.176067 36.960999

H 14.183311 35.104053 37.403969

H 13.202034 35.613514 39.594368

H 12.125414 34.278740 39.214470

H 10.804820 37.166840 37.066120

H 10.694811 35.398876 36.965889

O 45.364697 20.744726 23.816172

C 45.183792 20.570667 22.417349

C 46.418335 19.862700 21.919621

O 47.295959 19.760513 23.033398

C 46.292683 19.710644 24.018589

H 44.262161 19.988842 22.238401

H 44.998489 21.558790 21.971935

H 46.951267 20.360918 21.096754

H 46.180225 18.848175 21.556347

H 46.765472 19.833364 25.000355

H 45.779797 18.724476 24.029345

O 31.918198 9.368349 2.342454

C 33.273506 9.312122 1.912686

C 33.268127 9.648993 0.444925

O 31.929443 9.989777 0.128099

C 31.293837 9.191846 1.094221

H 33.854843 9.996133 2.547804

H 33.674427 8.304442 2.112169

H 33.573219 8.794348 48.707493

H 33.913025 10.489950 0.149612

H 31.305082 8.121583 0.794508

H 30.238729 9.491071 1.170982

O 7.960725 6.233832 1.657955

C 8.679939 7.457619 1.702447

C 8.749855 7.845338 3.157497

O 8.069757 6.823968 3.875732

C 8.279507 5.783041 2.949703

H 9.679796 7.309474 1.254100

H 8.161675 8.171943 1.048262

H 8.279507 8.806082 3.408317

H 9.788339 7.912322 3.528104

H 7.632655 4.930839 3.202967

H 9.329235 5.416344 2.994195

O 23.431385 15.083428 40.612316

C 23.069578 13.953515 41.393135

C 21.562702 13.941781 41.431271

O 21.137335 15.090274 40.706188

C 22.297071 15.866202 40.892960

H 23.533081 13.063178 40.943317

H 23.516947 14.056680 42.398857

H 21.165693 13.998497 42.460953

H 21.095287 13.057799 40.973633

H 22.340588 16.280813 41.924107

H 22.270670 16.723782 40.207970

O 33.880756 12.983972 7.270359

C 33.263725 12.525357 8.467255

C 33.141495 11.029238 8.337200

O 33.628956 10.704590 7.044475

C 34.519295 11.783653 6.904152

H 33.884178 12.825070 9.329235

H 32.307873 13.057799 8.581664

H 32.118168 10.638584 8.424718

H 33.738476 10.487994 9.094061

H 34.837585 11.851615 5.853935

H 35.437012 11.620841 7.511890

O 33.859241 42.418415 31.667377

C 33.415783 42.390057 30.319891

C 34.157974 43.496990 29.625126

O 35.037071 44.052898 30.592224

C 35.148544 42.922989 31.423889

H 33.646557 41.410244 29.868120

H 32.320095 42.460461 30.323803

H 33.532639 44.309101 29.235449

H 34.749580 43.128338 28.769499

H 35.612049 43.215855 32.376812

H 35.810066 42.149994 30.970165

O 2.789334 9.129752 19.483780

C 2.745331 8.730787 20.850334

C 2.891520 9.985865 21.659021

O 3.340356 10.973499 20.746681

C 2.609409 10.516353 19.637793

H 1.796810 8.205190 21.041994

H 3.543261 7.989572 21.007769

H 3.597532 9.934527 22.496555

H 1.924909 10.300246 22.094166

H 2.964859 11.028259 18.731810

H 1.530345 10.763261 19.745846

O 41.736851 23.708118 21.630175

C 42.438950 23.246571 20.486082

C 43.366936 24.361814 20.090052

O 43.056465 25.465324 20.932474

C 41.758362 25.079561 21.322638

H 42.929832 22.297071 20.743748

H 41.709957 23.009930 19.688641

H 43.239815 24.663485 19.037390

H 44.435242 24.138374 20.213751

H 41.018127 25.314249 20.527153

H 41.465004 25.656496 22.211998

O 27.493399 44.165844 20.347717

C 28.267370 42.989971 20.500261

C 29.666197 43.466679 20.766239

O 29.668638 44.869411 20.527641

C 28.505968 44.931995 19.741446

H 27.818537 42.366589 21.286947

H 28.214079 42.402767 19.565432

H 30.413275 42.987526 20.110096

H 30.005510 43.310219 21.796898

H 28.700073 44.583878 18.707851

H 28.188164 45.978298 19.656372

O 21.591549 33.273018 32.297604

C 21.326551 32.544514 31.111465

C 22.360634 31.461050 31.070396

O 23.378092 31.837036 31.992516

C 22.994772 33.172298 32.230133

H 20.288555 32.184662 31.134445

H 21.402334 33.227058 30.245575

H 22.813379 31.311928 30.077873

H 21.968512 30.484171 31.385754

H 23.394228 33.842617 31.440516

H 23.438231 33.515038 33.174255

O 7.738264 19.683752 10.335449

C 8.734209 20.604404 9.896880

C 9.309678 20.049959 8.622734

O 8.784081 18.738655 8.498057

C 7.553448 18.966494 9.138554

H 8.273640 21.598394 9.765359

H 9.469558 20.712456 10.707034

H 10.406833 19.988354 8.595842

H 9.018277 20.647919 7.740219

H 7.087011 17.998419 9.370794

H 6.858193 19.511162 8.463832

O 42.295204 15.178281 23.362936

C 41.923622 16.537500 23.183008

C 43.202656 17.332008 23.194256

O 44.250916 16.400600 23.418184

C 43.616779 15.262865 22.887209

H 41.376022 16.632841 22.228622

H 41.207829 16.801031 23.976051

H 43.262306 18.104515 23.974585

H 43.384537 17.847828 22.234978

H 44.168777 14.363237 23.195232

H 43.633400 15.284867 21.777342

O 15.456969 9.795183 18.706385

C 16.274946 10.953943 18.621311

C 15.879892 11.635998 17.342276

O 15.056537 10.712412 16.640665

C 15.402699 9.534096 17.325163

H 16.139513 11.550435 19.535118

H 17.336897 10.647385 18.604687

H 16.741383 11.905886 16.707647

H 15.299046 12.560071 17.479664

H 16.371265 9.134642 16.957979

H 14.648771 8.761101 17.119814

O 46.582615 47.585403 34.312477

C 45.584713 46.662308 33.881241

C 44.284164 47.144394 34.462090

O 44.639126 48.165276 35.382252

C 45.743614 48.656647 34.665485

H 45.584713 46.623684 32.778709

H 45.882469 45.659031 34.219582

H 43.695004 46.382645 34.992577

H 43.613354 47.567318 33.690071

H 46.302948 0.468883 35.296200

H 45.418476 0.325625 33.762436

O 46.402203 42.890228 7.165240

C 46.846638 41.608749 6.736450

C 47.692482 41.857128 5.517063

O 47.822048 43.270126 5.417811

C 46.561588 43.587929 5.960032

H 47.372723 41.135468 7.578873

H 45.968521 40.971676 6.531100

H 47.213333 41.473804 4.596901

H 48.697227 41.412201 5.545910

H 45.744102 43.356667 5.245220

H 46.508785 44.666992 6.163915

O 17.922146 19.083838 15.435456

C 17.163816 19.819185 14.478624

C 18.139229 20.680677 13.729098

O 19.425110 20.231840 14.114372

C 19.098017 19.858299 15.427633

H 16.622086 19.099483 13.849863

H 16.392778 20.403944 15.005690

H 18.032642 21.743607 14.000942

H 18.077623 20.645473 12.633410

H 18.984585 20.751081 16.080353

H 19.925283 19.267675 15.835889

O 29.223227 21.832102 42.913700

C 29.439331 23.180077 42.521088

C 28.120205 23.698830 42.016029

O 27.187820 22.634920 42.159775

C 28.103092 21.567591 42.105011

H 29.849543 23.720343 43.385025

H 30.221619 23.206966 41.743206

H 28.171541 23.996586 40.956032

H 27.729551 24.578409 42.545536

H 28.413561 21.370554 41.056751

H 27.612700 20.656719 42.469261

O 45.990520 1.119156 5.991323

C 46.584572 2.352233 6.369754

C 46.433983 3.262128 5.179214

O 45.700588 2.526780 4.209181

C 45.047871 1.646221 5.091696

H 46.070217 2.741908 7.268893

H 47.621586 2.153728 6.673378

H 47.380054 3.578464 4.716688

H 45.882469 4.188158 5.420256

H 44.591698 0.829222 4.516717

H 44.218159 2.156173 5.631961

O 20.494394 10.505107 39.648640

C 20.332071 9.140509 39.301987

C 19.143976 8.656959 40.085251

O 18.578285 9.791272 40.727211

C 19.139576 10.786730 39.910706

H 20.174635 9.058369 38.210213

H 21.271791 8.611978 39.512714

H 19.378172 7.919656 40.864601

H 18.379782 8.199812 39.437908

H 19.062325 11.758229 40.418209

H 18.565084 10.867402 38.965115

O 45.314335 7.948503 3.015219

C 45.697655 7.592563 4.336302

C 47.139503 7.158395 4.250251

O 47.520866 7.309962 2.886142

C 46.234989 7.133459 2.333653

H 45.505505 8.455032 4.990977

H 45.035645 6.784854 4.698109

H 47.272980 6.101332 4.544097

H 47.839161 7.731907 4.875590

H 45.925987 6.063685 2.368367

H 46.248188 7.422416 1.274146

O 21.541677 11.800277 1.009147

C 20.153612 11.524522 0.891805

C 19.559565 11.811033 2.241735

O 20.624939 12.280405 3.055800

C 21.476162 12.752220 2.041763

H 19.721888 12.161106 0.099741

H 20.046537 10.487505 0.543199

H 19.119040 10.940742 2.744353

H 18.765547 12.576695 2.206043

H 22.481398 12.911121 2.456374

H 21.137825 13.735943 1.658444

O 9.942840 37.682171 41.105644

C 10.908961 38.552948 40.551689

C 11.055639 39.649616 41.561813

O 10.587735 39.119129 42.801735

C 10.420033 37.771156 42.423794

H 11.854548 38.003395 40.383495

H 10.570134 38.863419 39.559650

H 10.444969 40.534576 41.328594

H 12.087767 40.009468 41.689423

H 9.707665 37.289558 43.108292

H 11.380776 37.225510 42.526470

O 18.608110 48.729988 27.014248

C 17.399969 48.064064 27.353565

C 17.332987 46.840767 26.486696

O 18.475611 46.890640 25.649651

C 19.310699 47.617676 26.516520

H 17.418549 47.812756 28.428717

H 16.570747 48.771545 27.210798

H 16.442160 46.771343 25.844732

H 17.363298 45.909851 27.082699

H 20.189304 47.959435 25.957188

H 19.690109 46.979137 27.345743

O 35.008713 0.408256 0.022002

C 34.413685 48.271374 0.805752

C 35.462437 47.198177 0.935808

O 36.615326 47.713017 0.281131

C 35.934254 48.499210 48.225414

H 34.075836 48.716297 1.754272

H 33.501835 47.908100 0.296291

H 35.155876 46.255524 0.445412

H 35.735748 46.924374 1.965490

H 36.650043 0.250333 47.693459

H 35.437988 47.871918 47.451927

O 22.221777 37.991173 19.548809

C 22.958591 37.581940 18.403740

C 22.016428 37.696350 17.235689

O 20.788240 38.181854 17.763731

C 21.304548 38.833595 18.897556

H 23.841597 38.232704 18.294218

H 23.357557 36.574257 18.585619

H 21.808144 36.754185 16.710093

H 22.386057 38.400402 16.469051

H 20.475817 39.071705 19.580589

H 21.781742 39.798248 18.619356

O 30.284687 29.767891 41.979847

C 31.241034 30.344826 41.100266

C 31.042526 31.835569 41.169693

O 29.933640 32.046295 42.030697

C 30.053915 30.881670 42.805649

H 32.248711 30.031424 41.422466

H 31.087507 29.906260 40.103340

H 30.823000 32.324497 40.209438

H 31.923086 32.359699 41.580879

H 29.122997 30.727657 43.369869

H 30.871893 30.989723 43.547348

O 2.466153 44.681660 5.952209

C 3.021575 45.386696 7.051319

C 2.853873 46.831482 6.691468

O 2.730174 46.878418 5.268199

C 3.015219 45.530441 4.968976

H 2.527758 45.061558 7.976860

H 4.088905 45.116810 7.166217

H 3.689451 47.479309 7.001937

H 1.939577 47.268093 7.118792

H 4.114329 45.377895 4.883902

H 2.593763 45.284023 3.984763

O 2.872941 19.623125 18.029709

C 1.897041 19.925772 19.022722

C 1.738139 21.418959 19.029566

O 2.791779 21.923531 18.228703

C 2.863162 20.845446 17.330051

H 0.959277 19.398706 18.784124

H 2.253469 19.509693 19.976620

H 1.798277 21.885883 20.020624

H 0.772995 21.742138 18.603222

H 3.789681 20.931496 16.748716

H 2.019762 20.880159 16.606928

O 42.824715 7.884452 43.849995

C 42.320629 6.716404 44.485603

C 41.188271 7.175996 45.361275

O 41.174583 8.594377 45.263977

C 41.664486 8.673094 43.946804

H 41.998917 5.997680 43.714561

H 43.147896 6.226987 45.020000

H 41.279213 6.900241 46.420288

H 40.215305 6.774586 45.023914

H 41.916286 9.716955 43.708206

H 40.893448 8.352845 43.209988

O 21.760229 40.630894 2.139060

C 22.225689 41.790634 2.827471

C 22.196842 41.458160 4.285943

O 21.379354 40.305267 4.389596

C 21.816456 39.701931 3.196611

H 23.228970 42.045853 2.458330

H 21.578348 42.635498 2.551715

H 21.800810 42.244846 4.943062

H 23.208435 41.217609 4.657528

H 21.164715 38.850220 2.960459

H 22.846138 39.297100 3.311998

O 46.637375 25.897049 14.185267

C 47.062744 25.779217 15.536176

C 46.030617 26.493542 16.364908

O 45.086494 27.031361 15.448169

C 45.919628 27.100300 14.317278

H 47.185463 24.710911 15.758148

H 48.064064 26.229031 15.649608

H 46.460869 27.319340 16.957491

H 45.491325 25.853533 17.077766

H 46.606571 27.971569 14.385240

H 45.301136 27.250891 13.421074

O 26.542433 10.574046 26.313126

C 26.617239 11.861393 25.716145

C 26.815256 12.844138 26.840189

O 26.759027 12.086789 28.039534

C 27.225466 10.871803 27.506599

H 27.453308 11.869705 24.995955

H 25.701967 12.013939 25.126009

H 26.062796 13.643536 26.896418

H 27.795069 13.353112 26.785431

H 27.046518 10.067027 28.231680

H 28.323599 10.911895 27.333521

O 9.328746 27.730042 41.745163

C 8.771857 29.035477 41.719738

C 9.766337 29.927773 42.411079

O 10.913363 29.128374 42.669724

C 10.683077 28.111404 41.725605

H 8.608067 29.338612 40.670498

H 7.776889 28.991964 42.181774

H 9.426532 30.352652 43.366936

H 10.068494 30.784374 41.789654

H 11.310861 27.245514 41.979359

H 10.983278 28.444853 40.708633

O 36.736584 6.981892 20.822954

C 35.924473 6.778986 21.973890

C 36.293613 5.427101 22.517580

O 37.221111 4.877546 21.593016

C 37.773602 6.088621 21.144669

H 36.111732 7.592073 22.696037

H 34.873768 6.877750 21.665865

H 35.455101 4.724022 22.624655

H 36.771786 5.489683 23.513037

H 38.378891 5.901850 20.246019

H 38.455166 6.523278 21.909353

O 12.136170 34.619038 25.676544

C 10.758861 34.254784 25.745483

C 9.962397 35.514263 25.576801

O 10.875226 36.568882 25.830065

C 12.014427 35.962120 25.268778

H 10.546666 33.496944 24.976887

H 10.599959 33.762924 26.715027

H 9.108239 35.618404 26.261299

H 9.554630 35.614006 24.552984

H 12.912587 36.507763 25.590981

H 11.976780 36.026661 24.161842

O 40.339985 16.724760 18.171009

C 38.966583 16.904686 18.473166

C 38.225368 15.870604 17.670835

O 39.211048 15.140143 16.953089

C 40.282776 15.360162 17.836094

H 38.815994 16.772186 19.558586

H 38.711365 17.949524 18.251194

H 37.498821 16.270056 16.949667

H 37.660168 15.174858 18.310843

H 41.222984 15.068272 17.347164

H 40.182549 14.731400 18.747944

O 8.814394 22.492643 23.544817

C 9.488626 23.290573 24.507027

C 9.131708 24.719221 24.189713

O 8.267283 24.670816 23.063223

C 8.739588 23.460722 22.527357

H 9.183535 22.945391 25.503460

H 10.575512 23.099403 24.436621

H 10.018623 25.318649 23.932047

H 8.618334 25.268778 24.991554

H 9.729667 23.601532 22.050163

H 8.053622 23.115049 21.742628

O 9.299411 8.457965 28.273727

C 9.013876 9.780516 28.727453

C 10.311980 10.377497 29.174334

O 11.155381 9.270075 29.434444

C 10.705078 8.455521 28.376890

H 8.529348 10.335938 27.912411

H 8.276084 9.700821 29.540051

H 10.248420 10.996968 30.080805

H 10.766194 11.010169 28.389116

H 11.047328 7.424860 28.548504

H 11.151958 8.791903 27.418104

O 25.446747 7.507489 6.159026

C 26.117065 6.354108 6.641109

C 27.570650 6.697336 6.582926

O 27.690926 7.838493 5.737570

C 26.400158 7.853162 5.182148

H 25.748415 6.113555 7.648301

H 25.868690 5.483327 6.008925

H 28.210167 5.889138 6.195207

H 27.960814 6.970646 7.570561

H 26.334150 7.163284 4.314301

H 26.188940 8.861331 4.800295

O 42.975792 24.783760 42.915169

C 42.132881 25.235039 43.967827

C 40.803974 25.560177 43.352268

O 40.912518 25.208639 41.985714

C 42.298138 25.399811 41.843437

H 42.095722 24.449821 44.734959

H 42.591984 26.118534 44.438175

H 40.562935 26.634840 43.431965

H 39.948841 25.015022 43.776657

H 42.551891 26.477894 41.774986

H 42.626698 24.947550 40.898827

O 31.242989 2.916944 29.106371

C 32.196884 3.949560 28.910803

C 32.121105 4.299144 27.451351

O 31.274279 3.328622 26.840679

C 31.333927 2.333164 27.832226

H 31.963179 4.776338 29.596767

H 33.197231 3.582864 29.207581

H 33.102379 4.288387 26.954599

H 31.698668 5.293134 27.249914

H 32.264359 1.734717 27.740797

H 30.494440 1.636931 27.695326

O 35.567558 32.112301 39.736645

C 35.189617 33.187943 40.581512

C 35.170547 32.630074 41.978870

O 35.456570 31.242989 41.869350

C 35.067871 31.064529 40.529686

H 35.889759 34.018631 40.412342

H 34.195133 33.556107 40.270065

H 34.184868 32.754753 42.458508

H 35.908829 33.064243 42.667278

H 33.963383 31.005857 40.448524

H 35.465858 30.109653 40.158588

O 34.213715 36.442249 41.832680

C 35.594448 36.753696 41.963223

C 36.101955 35.945011 43.128338

O 34.998936 35.179344 43.589397

C 33.972672 36.035461 43.156696

H 36.084354 36.537102 41.002480

H 35.700546 37.841072 42.117233

H 36.471584 36.572304 43.958538

H 36.920418 35.251709 42.886810

H 33.884178 36.910152 43.835819

H 33.013397 35.501549 43.194344

O 6.722271 47.557045 47.623543

C 7.212666 48.888889 47.662655

C 6.193740 0.800375 48.423428

O 5.216862 48.755901 48.855152

C 5.363051 47.872410 47.770710

H 8.219857 48.864929 48.102203

H 7.330497 0.362296 46.628574

H 5.709212 1.566036 47.788311

H 6.579993 1.342107 0.404345

H 4.955286 48.314884 46.835392

H 4.793939 46.953224 47.959435

O 6.831791 25.854513 16.964825

C 7.685459 24.892792 16.368820

C 8.905824 24.922615 17.222977

O 8.465300 25.248241 18.541616

C 7.099723 25.523998 18.309864

H 7.197998 23.898800 16.372732

H 7.853162 25.167568 15.317625

H 9.601568 25.711258 16.893929

H 9.476892 23.986807 17.263557

H 6.793655 26.358597 18.955250

H 6.495898 24.645393 18.606644

O 15.275089 40.557064 31.621906

C 16.580036 41.086086 31.785698

C 17.184351 40.248550 32.859386

O 16.552658 38.969517 32.775288

C 15.625160 39.195400 31.735826

H 17.141815 41.008347 30.834732

H 16.503765 42.154392 32.029182

H 16.974602 40.661209 33.858753

H 18.273684 40.118500 32.788979

H 14.719177 38.604774 31.930420

H 16.049551 38.828217 30.778996

O 13.867465 47.330185 28.254171

C 14.247361 45.970478 28.379335

C 12.966370 45.205303 28.271284

O 11.919576 46.094173 28.663893

C 12.673502 47.242668 28.998810

H 14.984177 45.732368 27.599983

H 14.752424 45.810596 29.352304

H 12.924323 44.304211 28.900532

H 12.760043 44.881634 27.238668

H 12.906721 47.239246 30.082760

H 12.061853 48.136425 28.810083

O 18.930315 31.885927 16.053951

C 19.294077 31.331972 14.795450

C 20.468481 30.426968 15.057515

O 20.614183 30.380030 16.469051

C 20.140900 31.676178 16.737961

H 18.402271 30.839622 14.382794

H 19.539030 32.148972 14.094817

H 21.398911 30.823977 14.610636

H 20.369717 29.396795 14.685930

H 20.894827 32.444775 16.459272

H 19.961464 31.782763 17.816046

O 2.224133 34.712910 44.732998

C 3.096870 34.646904 45.856556

C 2.365433 35.266865 47.012383

O 1.253611 35.926430 46.431538

C 1.022837 34.981331 45.413586

H 3.386315 33.598644 46.024750

H 4.016055 35.180813 45.587646

H 2.941391 35.997322 47.598118

H 2.008516 34.511471 47.738441

H 0.281623 35.379807 44.706600

H 0.589158 34.046013 45.830154

O 30.839132 35.003330 24.888390

C 29.977640 34.997467 26.013414

C 29.698465 36.439804 26.301880

O 30.036314 37.170750 25.123564

C 30.250952 36.099998 24.229805

H 29.056503 34.433243 25.770906

H 30.472437 34.456711 26.832367

H 30.311581 36.828011 27.127682

H 28.650692 36.646622 26.566391

H 30.915405 36.436867 23.421606

H 29.292654 35.797840 23.755545

O 2.268626 19.953640 23.931068

C 2.564427 21.073286 24.758337

C 3.730031 20.663565 25.618361

O 4.174468 19.408485 25.120632

C 3.583842 19.449068 23.844530

H 2.802046 21.943579 24.120773

H 1.655510 21.346596 25.313269

H 3.485568 20.535954 26.684223

H 4.571966 21.372997 25.583157

H 3.558418 18.429163 23.434809

H 4.200380 20.059250 23.150742

O 48.462055 40.449505 38.564194

C 48.819458 39.375816 37.707592

C 1.060485 38.671268 38.400894

O 1.141647 39.238426 39.702908

C 48.737320 39.778202 39.767937

H 47.948681 38.706474 37.569717

H 0.148634 39.787003 36.714581

H 2.043230 38.792526 37.922722

H 0.891805 37.584385 38.493301

H 48.683540 40.485195 40.607426

H 47.978992 38.988098 39.968887

O 1.226720 43.146431 13.932981

C 2.397214 42.359741 14.089438

C 3.127672 42.941078 15.262376

O 2.234401 43.855862 15.887226

C 1.028705 43.428539 15.298557

H 2.107769 41.311481 14.267897

H 2.958014 42.364143 13.144340

H 4.031211 43.498947 14.981243

H 3.443031 42.185196 15.999680

H 0.283089 44.229404 15.397321

H 0.624850 42.540157 15.831489

O 36.633907 5.939497 33.722832

C 35.382744 5.791841 34.384350

C 34.332035 5.709701 33.312134

O 35.036579 5.502396 32.101055

C 36.170406 6.268056 32.433529

H 35.454613 4.892703 35.009689

H 35.238998 6.633286 35.079117

H 33.739941 6.639153 33.225101

H 33.609398 4.891725 33.435341

H 35.942566 7.352988 32.373878

H 36.966381 6.063196 31.704536

O 22.477976 4.620858 13.153630

C 22.793335 3.484101 12.364989

C 23.019218 4.009210 10.973989

O 23.085712 5.429545 11.076175

C 23.282263 5.547866 12.464241

H 23.689539 2.986861 12.779599

H 21.974380 2.756576 12.459841

H 22.207600 3.770613 10.273355

H 23.946716 3.634691 10.510485

H 23.008463 6.565814 12.776666

H 24.353992 5.405588 12.721418

O 37.814182 41.093906 40.236820

C 39.103485 40.501328 40.079872

C 38.880043 39.146511 39.494137

O 37.591228 38.782745 39.957642

C 36.995224 40.044670 39.764027

H 39.733711 41.165783 39.474579

H 39.563076 40.443146 41.078266

H 39.608543 38.385250 39.804607

H 38.892754 39.161179 38.386715

H 36.035461 40.080364 40.295979

H 36.771297 40.197704 38.688873

O 32.267780 13.134562 26.917440

C 32.725418 13.269016 28.258574

C 31.583281 12.832893 29.139620

O 30.508617 12.511667 28.266394

C 30.894871 13.315465 27.179996

H 33.644112 12.674479 28.363691

H 33.019753 14.318746 28.441429

H 31.246899 13.619578 29.837320

H 31.798899 11.950378 29.759090

H 30.664099 14.386217 27.375078

H 30.311581 13.019664 26.296993

O 10.081206 10.063116 15.738592

C 10.941720 10.959810 15.062893

C 12.213421 10.191704 14.825763

O 11.937666 8.832973 15.141611

C 10.533465 8.890667 15.107387

H 10.456703 11.279569 14.122196

H 11.047817 11.864816 15.677965

H 13.048511 10.514885 15.458437

H 12.578650 10.242553 13.788748

H 10.127654 8.013530 15.629072

H 10.161880 8.843729 14.060102

O 26.265211 18.688784 42.840359

C 26.711601 17.529047 43.524857

C 25.525953 16.600573 43.573261

O 24.429775 17.326141 43.029083

C 24.926527 18.621311 43.260349

H 27.595583 17.141815 43.000240

H 27.056787 17.815557 44.536449

H 25.278555 16.291080 44.603924

H 25.635473 15.673564 42.992905

H 24.835587 18.905378 44.330612

H 24.325636 19.343946 42.699059

O 14.185757 4.368083 10.102719

C 14.953862 3.758878 9.078904

C 16.029993 2.979527 9.789316

O 15.768905 3.130117 11.177382

C 14.392085 3.398538 11.098177

H 14.295765 3.107626 8.473611

H 15.298068 4.551919 8.399294

H 17.063097 3.305153 9.611835

H 16.015326 1.907797 9.532629

H 14.043480 3.784303 12.067232

H 13.808793 2.473487 10.889404

O 31.715780 31.584749 0.621427

C 30.945719 30.705656 48.710918

C 30.614227 29.524406 0.684499

O 31.299215 29.712643 1.915620

C 32.279026 30.625473 1.481452

H 30.077873 31.262056 48.327110

H 31.539766 30.408875 47.829384

H 30.938875 28.571486 0.235663

H 29.546406 29.412441 0.910873

H 33.110691 30.089117 0.976878

H 32.707325 31.137869 2.353210

O 41.950512 36.241299 30.654808

C 41.603863 34.867413 30.535507

C 41.209297 34.649837 29.099039

O 41.488964 35.869225 28.428228

C 41.274323 36.730228 29.520983

H 42.469261 34.278252 30.871403

H 40.786373 34.638592 31.240053

H 40.133167 34.416130 28.995874

H 41.746628 33.844086 28.578817

H 40.189392 36.853924 29.726334

H 41.667423 37.724705 29.276030

O 9.608902 1.015503 38.452229

C 9.066192 48.741234 37.868450

C 8.419340 48.014194 39.008141

O 8.273150 0.053293 40.084274

C 8.588998 1.240410 39.393417

H 8.346978 0.127610 37.074921

H 9.877323 48.189232 37.370235

H 9.037345 47.181065 39.368969

H 7.433661 47.590294 38.759766

H 8.921958 2.001671 40.113609

H 7.683014 1.640354 38.893734

O 0.706012 10.225929 43.416317

C 1.707825 9.221182 43.451519

C 3.016686 9.942350 43.305332

O 2.710128 11.275658 42.904411

C 1.356286 11.120178 42.545048

H 1.596350 8.654514 44.387817

H 1.531322 8.500502 42.631588

H 3.686517 9.485204 42.559715

H 3.597043 10.002977 44.236740

H 1.270235 10.770595 41.495319

H 0.860024 12.099990 42.597363

O 33.678829 4.723044 46.045773

C 33.221680 4.604235 47.382992

C 33.165943 6.008925 47.920811

O 33.412361 6.879217 46.825123

C 33.104336 5.981056 45.789574

H 32.224266 4.128019 47.387878

H 33.886131 3.910446 47.917877

H 33.914490 6.233832 48.690384

H 32.186619 6.257790 48.362312

H 33.511616 6.377088 44.855721

H 32.004738 5.896960 45.649738

O 32.889694 10.922162 16.007502

C 32.593407 9.661218 16.596170

C 31.865395 9.951152 17.881563

O 31.675688 11.357308 17.918232

C 32.815380 11.708359 17.172617

H 32.022339 9.070592 15.866202

H 33.534592 9.111174 16.759964

H 32.450150 9.650461 18.768478

H 30.885094 9.463202 17.981794

H 33.737495 11.599328 17.784756

H 32.747421 12.766399 16.886595

O 6.083242 29.131308 8.475567

C 6.827391 30.155123 7.829692

C 5.839756 31.242008 7.487933

O 4.588100 30.825441 8.010596

C 5.078006 29.961998 9.002631

H 7.608697 30.516439 8.521526

H 7.361300 29.713619 6.975047

H 5.699923 31.433180 6.415713

H 6.113067 32.211067 7.941169

H 4.249273 29.341547 9.355148

H 5.451058 30.531111 9.881234

O 36.846104 21.596437 8.654025

C 36.416336 22.836849 9.196246

C 35.589069 22.485310 10.405365

O 35.391544 21.072796 10.363807

C 35.694675 20.868425 9.002631

H 37.298851 23.465122 9.385951

H 35.825710 23.379070 8.434497

H 34.608768 22.992327 10.413189

H 36.058441 22.733685 11.369042

H 34.833187 21.156403 8.360180

H 35.873135 19.797672 8.821239

O 20.178059 26.093597 39.600231

C 20.510529 24.973953 38.802792

C 19.920393 23.809814 39.530319

O 19.738024 24.219048 40.885624

C 20.370697 25.477060 40.848953

H 20.106676 25.127478 37.793644

H 21.610617 24.903547 38.697674

H 20.537910 22.902855 39.510269

H 18.934225 23.522816 39.133308

H 21.446827 25.361671 41.078266

H 19.949730 26.122444 41.630753

O 23.137539 47.442150 7.994950

C 23.426006 48.484543 7.070388

C 24.528051 0.415589 7.677636

O 24.676195 48.825325 9.002142

C 24.353504 47.490067 8.701941

H 23.722298 48.035221 6.108178

H 22.498022 0.146678 6.876284

H 24.345680 1.498075 7.719195

H 25.479992 0.278200 7.128570

H 24.269897 46.912151 9.629437

H 25.166103 47.009937 8.122071

O 43.297997 13.916357 25.744503

C 44.135529 12.845116 26.142002

C 45.523598 13.406405 26.121466

O 45.391586 14.828208 26.121466

C 44.012318 14.938705 26.394287

H 43.845108 12.505311 27.154572

H 43.965382 11.994871 25.465815

H 46.092220 13.120383 25.225262

H 46.132309 13.095448 26.983938

H 43.653450 15.916562 26.042749

H 43.826527 14.905949 27.488508

O 42.183727 12.570827 32.118660

C 41.304638 13.177588 31.179916

C 42.118214 13.430851 29.937059

O 43.399204 12.871519 30.183481

C 43.389915 13.046065 31.578390

H 40.438747 12.514601 31.038616

H 40.907139 14.107039 31.621906

H 42.237999 14.509427 29.729755

H 41.716805 12.988860 29.014942

H 43.545395 14.114372 31.845346

H 44.225494 12.484288 32.015984

O 5.559600 40.255886 24.509958

C 6.939355 39.964973 24.335413

C 7.297250 38.975384 25.413012

O 6.097910 38.726517 26.133202

C 5.190948 39.035522 25.103029

H 7.096790 39.554276 23.321377

H 7.494777 40.912518 24.368172

H 8.059978 39.319588 26.125866

H 7.676170 38.022465 25.002800

H 4.177890 39.123528 25.521063

H 5.154768 38.221947 24.347149

O 1.158759 11.204762 12.516068

C 2.554160 11.447271 12.651989

C 2.995173 10.721213 13.893867

O 1.813434 10.183393 14.467379

C 1.085909 10.021557 13.274884

H 3.070468 11.078130 11.749917

H 2.710128 12.535625 12.670568

H 3.494857 11.339218 14.652683

H 3.696295 9.897369 13.667004

H 0.035692 9.807406 13.517880

H 1.461406 9.152243 12.691592

O 0.820912 47.394722 33.761456

C 0.653699 46.804588 35.046848

C 48.885464 45.476658 34.832699

O 48.400936 45.491817 33.503300

C 0.522173 46.285347 32.942501

H 1.636444 46.715603 35.535286

H 0.065518 47.503754 35.659962

H 48.040108 45.259575 35.500084

H 0.711389 44.646461 34.951996

H 0.190195 46.639332 31.956333

H 1.433047 45.679077 32.773819

O 36.491138 27.165329 43.195812

C 36.292149 28.226789 42.266357

C 37.648434 28.789547 41.952957

O 38.494766 28.287418 42.973347

C 37.882141 27.025005 43.061844

H 35.743572 27.842493 41.395580

H 35.624271 28.958225 42.735729

H 37.703194 29.885723 41.951488

H 38.029797 28.466366 40.966301

H 38.263016 26.503319 43.947781

H 38.132473 26.398689 42.176884

O 13.004995 47.091587 46.609505

C 12.313652 46.137199 47.409882

C 12.216844 44.885059 46.587994

O 12.586963 45.257130 45.270332

C 13.526682 46.242321 45.616493

H 12.863207 45.998344 48.353024

H 11.340685 46.570393 47.687103

H 11.219431 44.426441 46.545456

H 12.902810 44.099350 46.955177

H 13.759901 46.843704 44.726643

H 14.477158 45.779797 45.958252

O 36.222717 17.889875 23.711052

C 35.500572 18.359245 22.577717

C 34.963242 19.711620 22.939524

O 35.123123 19.819674 24.341280

C 36.326374 19.096062 24.430265

H 36.181160 18.388092 21.712315

H 34.722198 17.617542 22.345476

H 33.906178 19.879324 22.687725

H 35.535286 20.518841 22.450596

H 36.541500 18.874086 25.484882

H 37.179062 19.697443 24.050367

O 34.584812 46.329350 6.575104

C 34.886478 47.621586 7.075766

C 35.849667 48.197544 6.082753

O 35.677563 47.461708 4.870701

C 34.532986 46.714134 5.218328

H 33.960449 48.225414 7.150572

H 35.274197 47.524776 8.099581

H 36.895485 48.076778 6.399579

H 35.702988 0.375983 5.877892

H 34.480667 45.819397 4.580766

H 33.611843 47.302807 5.011512

O 12.331252 8.952271 8.966451

C 11.013103 8.992364 9.494493

C 10.315403 7.764177 8.974274

O 11.253166 7.097768 8.137717

C 12.045719 8.212523 7.803780

H 11.077641 9.073037 10.588714

H 10.522709 9.917904 9.152732

H 9.418709 8.018909 8.380714

H 9.979020 7.054253 9.744335

H 11.539679 8.847641 7.043497

H 12.986417 7.866363 7.351033

O 41.140846 44.782871 18.405205

C 40.164455 43.787415 18.131895

C 40.317490 43.441742 16.673912

O 41.371132 44.257763 16.181072

C 42.055630 44.425953 17.397526

H 40.341938 42.917122 18.788525

H 39.182201 44.186378 18.421339

H 39.426662 43.618732 16.055416

H 40.585915 42.383209 16.516966

H 42.801247 45.223885 17.286539

H 42.616920 43.505302 17.669859

O 30.230906 24.779850 24.817986

C 29.255985 25.672632 24.306078

C 27.952501 25.259975 24.915770

O 28.256615 24.257185 25.874557

C 29.619745 24.552498 26.060841

H 29.526361 26.710625 24.571077

H 29.282875 25.611025 23.212835

H 27.249914 24.829718 24.195580

H 27.423969 26.093597 25.404697

H 30.105742 23.697851 26.550259

H 29.748823 25.432079 26.728228

O 41.171158 20.076361 33.803505

C 40.892960 20.426434 35.151478

C 41.517323 21.783699 35.365631

O 42.073231 22.153816 34.111038

C 41.209785 21.390600 33.304798

H 39.796783 20.453325 35.283489

H 41.254765 19.615791 35.800285

H 42.318676 21.831614 36.115154

H 40.777084 22.545448 35.672188

H 41.582836 21.386688 32.272182

H 40.191837 21.832102 33.265682

O 11.396422 4.401819 39.267273

C 11.615952 5.526842 38.429253

C 12.193376 5.001733 37.141415

O 12.316095 3.597532 37.302761

C 11.307926 3.422007 38.265461

H 10.661564 6.055862 38.268394

H 12.258403 6.221120 38.984673

H 13.182966 5.398743 36.878372

H 11.541145 5.208550 36.275036

H 11.430159 2.440240 38.734344

H 10.308069 3.437164 37.785824

O 1.801211 21.986603 12.108302

C 0.389187 22.127903 12.039852

C 0.133966 23.376625 11.238009

O 1.412513 23.880711 10.866914

C 2.137104 23.342400 11.946956

H 48.866886 21.201874 11.614974

H 48.876175 22.201242 13.065133

H 48.491879 24.141798 11.829613

H 48.434185 23.235813 10.322248

H 1.946911 23.914934 12.881785

H 3.213724 23.425030 11.742095

O 46.821701 19.756113 45.523106

C 45.919628 18.673626 45.351494

C 46.751293 17.529047 44.841053

O 48.112473 17.915789 44.998489

C 47.944279 18.990452 45.891270

H 45.444389 18.436987 46.322018

H 45.104584 18.998764 44.688995

H 46.601192 17.289471 43.778122

H 46.574303 16.591282 45.391586

H 48.840485 19.624102 45.853622

H 47.851871 18.623756 46.936111

O 45.792019 2.726263 12.165996

C 45.983189 2.523357 13.556018

C 46.227165 3.889911 14.123663

O 46.592884 4.727445 13.026998

C 46.744938 3.749100 12.022251

H 45.108009 2.001182 13.965250

H 46.845657 1.848637 13.715407

H 47.026073 3.919247 14.881013

H 45.335850 4.329947 14.596457

H 47.772175 3.324711 12.044742

H 46.617817 4.225316 11.040483

O 18.178831 5.907717 14.060102

C 19.428043 5.498484 13.514460

C 19.115618 4.368572 12.566916

O 17.698704 4.275675 12.550292

C 17.491888 4.696642 13.876265

H 19.908659 6.377088 13.062201

H 20.095428 5.188993 14.336836

H 19.539518 3.405384 12.909655

H 19.469601 4.492270 11.533322

H 17.822403 3.922669 14.606725

H 16.418201 4.850655 14.054724

O 29.391905 40.286686 36.829967

C 30.770193 40.409409 36.484783

C 31.560791 40.028046 37.698792

O 30.659698 39.311279 38.524105

C 29.493114 40.031956 38.212658

H 30.957943 39.750824 35.624271

H 30.956474 41.436161 36.136665

H 31.937265 40.912518 38.245415

H 32.432549 39.386574 37.501266

H 29.473070 40.985367 38.778343

H 28.615978 39.448666 38.525570

O 17.947569 44.843498 15.642273

C 18.487345 43.551262 15.399276

C 17.479664 42.821781 14.550986

O 16.328730 43.651981 14.511382

C 17.018606 44.878212 14.590100

H 19.468624 43.660782 14.906926

H 18.685360 43.082867 16.374199

H 17.186796 41.829258 14.922571

H 17.844404 42.667278 13.517880

H 16.310148 45.691788 14.784204

H 17.522202 45.110939 13.627400

O 20.952520 19.709665 7.563227

C 20.925140 18.299109 7.430727

C 20.919762 17.769110 8.832973

O 20.523731 18.843775 9.686152

C 20.172680 19.817228 8.728342

H 20.026979 17.996462 6.864060

H 21.787119 17.991573 6.820056

H 21.912287 17.431749 9.168866

H 20.232330 16.924244 8.988453

H 20.318871 20.815620 9.162999

H 19.096550 19.725800 8.482412

O 10.307580 3.545706 4.286921

C 10.024980 2.174752 4.070815

C 11.240455 1.429137 4.550453

O 12.061365 2.374723 5.228596

C 11.097688 3.379470 5.438346

H 9.112151 1.900463 4.633082

H 9.782472 2.032963 3.009352

H 11.835480 0.976878 3.743721

H 11.002347 0.610182 5.246686

H 11.600306 4.328969 5.668142

H 10.468437 3.136962 6.319883

O 13.362891 31.135424 36.768852

C 12.384057 30.097431 36.699425

C 12.391391 29.414886 38.033707

O 13.643047 29.754204 38.605755

C 13.671894 31.083597 38.142742

H 11.415491 30.536976 36.423180

H 12.661280 29.428089 35.872158

H 12.300450 28.320665 38.001438

H 11.575371 29.773272 38.690338

H 14.675662 31.503586 38.299683

H 12.967837 31.717249 38.723099

O 3.095892 42.096210 27.019138

C 2.181597 41.348640 27.809246

C 2.870007 41.104176 29.127398

O 4.133398 41.751026 29.044277

C 4.283009 41.677200 27.646435

H 1.243344 41.918240 27.871830

H 1.932732 40.410877 27.281204

H 3.029887 40.028046 29.321501

H 2.340987 41.497272 30.007956

H 4.557298 40.646542 27.333031

H 5.105875 42.332851 27.331076

O 46.132801 21.505009 47.449486

C 45.840420 20.662098 48.556419

C 44.370705 20.771618 48.682560

O 44.123795 22.163595 48.530994

C 45.205303 22.540558 47.700306

H 46.227654 19.655884 48.352535

H 46.339619 21.037104 0.576448

H 43.945339 20.439146 0.745124

H 43.863686 20.219128 47.870941

H 45.720634 23.396671 48.167721

H 44.808292 22.883297 46.734184

O 4.997822 28.787592 34.376038

C 5.308291 30.113564 33.972183

C 6.131646 30.713966 35.075695

O 6.359487 29.661797 36.001232

C 5.175303 28.948938 35.761169

H 5.825577 30.056360 33.005573

H 4.369061 30.661163 33.792747

H 5.609960 31.543680 35.587601

H 7.105590 31.115377 34.761314

H 4.305989 29.466713 36.218319

H 5.241797 27.959837 36.233963

O 44.975021 20.890915 28.423830

C 43.636337 20.609781 28.049311

C 43.718964 19.378172 27.189287

O 45.084541 19.242250 26.806454

C 45.546089 20.511997 27.197598

H 43.030064 20.512974 28.959206

H 43.211945 21.471762 27.497309

H 43.089222 19.434889 26.286724

H 43.434410 18.450186 27.706572

H 45.322647 21.270323 26.414335

H 46.638355 20.467503 27.300762

O 30.829844 44.274876 28.179853

C 30.378075 45.063515 27.084167

C 29.933149 44.104729 26.012924

O 30.164902 42.805649 26.537056

C 31.188227 43.129807 27.443041

H 29.603613 45.751926 27.449883

H 31.207294 45.702545 26.743385

H 30.502752 44.229404 25.074671

H 28.873644 44.171223 25.725435

H 32.153374 43.283817 26.915485

H 31.342728 42.289825 28.132917

O 12.378679 45.413586 7.558338

C 11.444826 45.967545 8.469211

C 11.147558 47.349255 7.951437

O 11.929843 47.497398 6.769697

C 11.952823 46.131336 6.430870

H 10.542265 45.329979 8.483878

H 11.876060 45.920605 9.479825

H 11.381266 48.167721 8.647670

H 10.078761 47.473930 7.700127

H 12.657857 45.953365 5.605560

H 10.959321 45.791527 6.069063

O 44.245049 4.956752 41.274323

C 45.089428 4.966531 40.133167

C 46.481407 4.753358 40.659740

O 46.400246 4.880479 42.076653

C 45.165703 5.550311 42.154392

H 44.731533 4.217493 39.412487

H 44.992134 5.939986 39.623215

H 47.202576 5.493106 40.275444

H 46.907753 3.766212 40.431900

H 45.287933 6.633286 41.931442

H 44.779938 5.486750 43.180164

O 42.766533 33.778076 18.049755

C 41.363308 33.678829 18.240437

C 40.978523 32.297119 17.783779

O 42.170528 31.693781 17.293385

C 42.864315 32.876007 16.977047

H 41.136444 33.894444 19.292610

H 40.864113 34.475780 17.661545

H 40.221172 32.311295 16.980959

H 40.566845 31.650755 18.571440

H 42.476597 33.324356 16.036839

H 43.921379 32.639854 16.795654

O 6.371221 37.491978 6.627908

C 6.965268 36.340549 6.053418

C 6.144358 36.048172 4.837453

O 5.525864 37.278313 4.455112

C 6.061729 38.152027 5.425634

H 8.022330 36.548347 5.807976

H 6.980914 35.536263 6.800988

H 5.343005 35.319668 5.040359

H 6.720804 35.654587 3.986230

H 5.328337 38.944580 5.627072

H 6.967713 38.648781 5.028135

O 20.335981 37.912945 29.626102

C 21.356863 37.645008 30.578533

C 21.761208 38.977341 31.153027

O 20.988701 39.954708 30.470972

C 19.889591 39.138683 30.151213

H 20.961321 36.954643 31.344196

H 22.164083 37.100834 30.067116

H 22.819736 39.237450 31.022968

H 21.562214 39.047745 32.238445

H 19.270119 39.649616 29.400705

H 19.247139 38.970985 31.042038

O 45.945541 25.960609 5.611916

C 47.185951 26.599640 5.872514

C 47.210888 27.822449 5.003200

O 45.868290 28.020952 4.573432

C 45.206284 27.148706 5.453992

H 47.218220 26.873928 6.941310

H 48.004906 25.885803 5.705790

H 47.841114 27.730042 4.105528

H 47.559006 28.725498 5.531242

H 44.215717 26.919886 5.038892

H 45.034668 27.641544 6.435760

O 19.954130 7.053276 17.440062

C 19.937994 5.630984 17.477221

C 18.495169 5.223218 17.467442

O 17.774977 6.378554 17.065542

C 18.598822 7.314363 17.715816

H 20.508574 5.272600 16.609863

H 20.481682 5.286290 18.370491

H 18.155363 4.908837 18.472189

H 18.249725 4.394485 16.789787

H 18.408628 7.308496 18.809549

H 18.358269 8.325954 17.362322

O 45.109474 46.990868 17.772533

C 46.138180 46.065331 18.083002

C 47.134129 46.182671 16.966290

O 46.477497 46.875481 15.907762

C 45.149567 46.826099 16.374687

H 46.531765 46.300014 19.081392

H 45.706947 45.047871 18.150475

H 47.490067 45.208729 16.597149

H 48.035706 46.756672 17.226400

H 44.665527 45.872688 16.077908

H 44.575077 47.631367 15.896515

O 3.675761 26.429003 11.847703

C 4.072770 26.569326 13.217191

C 4.422354 28.009218 13.425962

O 3.708030 28.697628 12.417304

C 3.907024 27.749109 11.402289

H 3.227902 26.247122 13.843018

H 4.899058 25.877981 13.436719

H 5.507285 28.198435 13.311554

H 4.139753 28.418940 14.404307

H 4.937684 27.835649 10.998924

H 3.213235 27.965704 10.579424

O 1.946911 16.253922 42.073231

C 2.246135 16.477362 43.440765

C 1.428159 15.471148 44.202515

O 0.532932 14.877590 43.265236

C 0.611160 15.849091 42.249245

H 3.332533 16.394245 43.578640

H 1.983581 17.519268 43.704784

H 0.844379 15.928296 45.017555

H 2.016828 14.663440 44.663082

H 48.854176 16.719870 42.484909

H 0.238597 15.418344 41.309036

O 47.342896 3.449387 23.395205

C 45.938210 3.474811 23.613266

C 45.400875 4.597879 22.769377

O 46.530788 5.249619 22.211020

C 47.372234 4.123130 22.159683

H 45.512840 2.493533 23.337511

H 45.762196 3.593621 24.691353

H 44.801937 5.345939 23.308176

H 44.766247 4.221405 21.948467

H 48.399960 4.454134 21.955313

H 47.077900 3.442542 21.330462

O 34.003475 19.766382 27.173151

C 33.003616 18.948404 26.588882

C 32.626163 18.036064 27.690435

O 33.870487 17.650789 28.265415

C 34.645927 18.802704 27.983305

H 32.196884 19.592323 26.221209

H 33.418228 18.392004 25.728857

H 32.073677 17.133505 27.398546

H 32.023808 18.579754 28.440943

H 35.562668 18.468277 27.476776

H 34.947105 19.286253 28.924490

O 36.088753 3.003485 37.996552

C 35.861889 1.737650 38.597931

C 36.119556 1.923932 40.064716

O 36.770809 3.181943 40.198681

C 37.141415 3.379959 38.854618

H 34.845409 1.402734 38.349556

H 36.554214 0.999369 38.153496

H 36.753696 1.134802 40.501816

H 35.201839 1.959135 40.667076

H 38.069889 2.816714 38.612602

H 37.371700 4.442889 38.711365

O 8.793859 5.813843 24.189713

C 7.441973 6.252411 24.158421

C 6.729605 5.365985 23.177143

O 7.719195 4.499605 22.640789

C 8.597310 4.496671 23.736965

H 7.010738 6.196184 25.172459

H 7.448329 7.315341 23.890488

H 6.243122 5.894516 22.344498

H 5.938030 4.762648 23.652380

H 9.562943 4.070815 23.427475

H 8.208611 3.853730 24.552984

O 48.722164 42.649677 33.332664

C 48.504101 41.580395 32.422771

C 0.113431 40.337048 33.107761

O 0.654186 40.765839 34.348171

C 48.734386 41.899174 34.519783

H 47.428459 41.529545 32.176842

H 0.124677 41.831699 31.484028

H 0.897183 39.789940 32.566025

H 48.201454 39.603657 33.296974

H 0.247398 42.509842 35.337761

H 47.704216 41.617550 34.816563

O 10.638584 1.408602 19.812828

C 10.522220 0.021024 19.547829

C 10.740770 48.225903 20.866959

O 10.759350 0.354962 21.856548

C 10.115432 1.364598 21.116800

H 9.517472 48.710430 19.131752

H 11.238499 48.641979 18.761633

H 11.684401 47.665592 20.938829

H 9.939906 47.508644 21.107512

H 10.279712 2.327297 21.615505

H 9.015343 1.211564 21.089420

O 23.115049 26.145914 46.978157

C 21.802767 26.229521 47.531136

C 21.867306 27.242090 48.641979

O 23.249504 27.467976 48.867867

C 23.651403 27.327652 47.526737

H 21.091375 26.502342 46.734673

H 21.519188 25.220861 47.864582

H 21.396467 26.929176 0.692813

H 21.383266 28.198435 48.365738

H 24.748068 27.275826 47.479797

H 23.342400 28.211145 46.926819

O 31.917707 42.138260 22.824625

C 30.885094 41.321751 23.363424

C 31.519722 40.502308 24.449335

O 32.859386 40.965321 24.580854

C 32.728840 42.224312 23.970184

H 30.075428 41.966156 23.751635

H 30.444078 40.737968 22.542025

H 31.556391 39.421772 24.245449

H 30.998524 40.615246 25.414478

H 33.726254 42.586117 23.680738

H 32.315697 42.963081 24.684998

O 22.646656 28.006773 12.370367

C 22.984993 27.129637 13.437209

C 24.349104 27.547180 13.920268

O 24.784737 28.552904 13.021620

C 23.514013 29.063835 12.701371

H 22.209555 27.206398 14.219492

H 22.932678 26.104353 13.045088

H 25.106941 26.752672 13.944226

H 24.326612 27.968637 14.939683

H 23.596643 29.744423 11.844280

H 23.102337 29.661306 13.542816

O 6.332595 19.522896 42.668259

C 6.937399 19.085304 43.878353

C 5.806998 18.835461 44.843987

O 4.608635 19.063303 44.114017

C 5.134233 20.012312 43.217815

H 7.558338 18.206211 43.651981

H 7.637055 19.863190 44.232338

H 5.835845 19.522896 45.710365

H 5.765928 17.820448 45.264954

H 5.296068 20.991634 43.722874

H 4.412086 20.185881 42.407658

O 45.915714 19.856833 36.691601

C 46.024261 21.212629 37.102791

C 44.989197 21.414068 38.173054

O 44.429867 20.133078 38.414585

C 44.593655 19.629971 37.113544

H 47.056385 21.379843 37.434284

H 45.863403 21.869261 36.229565

H 44.193714 22.107368 37.844494

H 45.364204 21.812056 39.125484

H 43.864174 20.086628 36.409489

H 44.395149 18.550417 37.115013

O 0.466926 39.056057 12.150839

C 48.818485 40.365406 12.299961

C 0.635606 41.186317 11.290325

O 1.968913 40.701298 11.334329

C 1.674578 39.325943 11.468295

H 47.727684 40.316513 12.188486

H 0.126632 40.726723 13.322310

H 0.642451 42.263912 11.495186

H 0.208772 41.035725 10.281178

H 2.506245 38.827728 11.985580

H 1.600261 38.864395 10.471371

O 15.696545 25.509331 39.723934

C 16.985847 26.122444 39.771847

C 16.780987 27.562828 40.153214

O 15.425677 27.647900 40.556580

C 14.910837 26.680311 39.677975

H 17.587229 25.561644 40.499371

H 17.480154 26.000214 38.793991

H 16.960423 28.253683 39.307365

H 17.410725 27.925611 40.979500

H 14.863411 27.081722 38.643402

H 13.880666 26.437803 39.974754

O 34.796516 46.008125 25.527418

C 34.988663 47.240711 26.200186

C 34.027920 47.223110 27.359434

O 33.472500 45.914249 27.405392

C 34.468445 45.251263 26.665155

H 36.041817 47.316982 26.529232

H 34.846390 48.057709 25.479992

H 33.198212 47.939388 27.272892

H 34.516361 47.435795 28.325554

H 34.097351 44.266563 26.349796

H 35.366116 45.069382 27.297337

O 15.033069 36.036438 18.319155

C 14.796429 34.633705 18.198389

C 13.423517 34.383369 18.729855

O 13.205457 35.435547 19.654905

C 13.820527 36.458870 18.901957

H 14.943596 34.333504 17.151594

H 15.566489 34.118374 18.792437

H 13.298352 33.419693 19.243717

H 12.658345 34.414665 17.932411

H 14.026365 37.317429 19.556143

H 13.134072 36.815300 18.107937

O 8.489745 18.108427 20.364828

C 9.808384 17.971527 19.846075

C 9.707665 17.067499 18.648691

O 8.386093 16.551189 18.661892

C 7.757820 17.664968 19.250072

H 10.201482 18.973339 19.607479

H 10.444480 17.577938 20.651831

H 10.404387 16.217741 18.648691

H 9.877323 17.608253 17.698704

H 6.745739 17.383835 19.576187

H 7.641455 18.480499 18.507881

O 20.107164 33.303329 0.267444

C 21.455139 32.845695 0.295312

C 21.461494 31.595016 1.136758

O 20.137478 31.456648 1.631553

C 19.488670 32.069767 0.543688

H 21.793478 32.664299 48.152073

H 22.083410 33.661224 0.680099

H 22.152349 31.606749 1.992382

H 21.722582 30.695879 0.547599

H 18.430630 32.235512 0.791085

H 19.510183 31.415092 48.538815

O 7.145682 30.586357 17.396545

C 7.663946 31.008303 16.140980

C 7.597941 29.810427 15.229618

O 7.153995 28.719141 16.024126

C 6.416202 29.464756 16.962868

H 7.071365 31.863926 15.772818

H 8.678472 31.396999 16.312593

H 8.554284 29.525383 14.768070

H 6.885573 29.957108 14.399419

H 6.181517 28.828661 17.828270

H 5.452525 29.783539 16.527233

O 28.106028 25.388563 11.321617

C 27.249914 26.418736 11.783653

C 26.035418 26.322416 10.918740

O 26.431448 25.660896 9.717933

C 27.820492 25.542576 9.949685

H 27.047987 26.270590 12.851472

H 27.753998 27.400015 11.701024

H 25.590492 27.294895 10.662541

H 25.243353 25.716635 11.384688

H 28.352446 26.420691 9.532629

H 28.201857 24.668861 9.403552

O 24.973463 24.223936 26.127821

C 25.563112 23.632824 27.287560

C 25.669209 22.159195 27.022562

O 24.790606 21.916197 25.936165

C 25.025780 23.121405 25.252642

H 26.530210 24.116373 27.484598

H 24.927015 23.879732 28.149054

H 25.379763 21.507942 27.860094

H 26.698402 21.861927 26.743385

H 24.256207 23.246082 24.479158

H 26.005104 23.086691 24.733400

O 45.407719 16.268591 1.524966

C 44.603436 16.933043 0.564223

C 44.478268 15.987945 48.291908

O 45.117786 14.777848 48.675224

C 45.059113 14.950439 1.175872

H 43.622154 17.170172 1.014526

H 45.075249 17.897697 0.334916

H 44.944706 16.338018 47.360008

H 43.430496 15.763527 48.044510

H 45.761215 14.252739 1.651599

H 44.048988 14.701088 1.562614

O 24.058680 20.191259 34.599964

C 25.107430 19.582056 33.859241

C 26.016346 20.700722 33.438766

O 25.597338 21.850193 34.163357

C 24.851721 21.210186 35.165169

H 25.641829 18.859909 34.504139

H 24.671795 18.985561 33.047131

H 25.995323 20.951054 32.368500

H 27.068031 20.488527 33.676872

H 24.202425 21.952868 35.642849

H 25.517641 20.806820 35.959675

O 31.633152 40.107742 42.601765

C 31.867838 39.814873 41.229832

C 33.350758 39.894569 41.013237

O 33.876842 40.492531 42.185688

C 32.942993 39.964485 43.098022

H 31.450293 38.821861 40.997101

H 31.291391 40.543865 40.642632

H 33.663670 40.494972 40.146858

H 33.807415 38.897644 40.876335

H 33.022686 40.497906 44.055836

H 33.163498 38.898136 43.305820

O 48.372093 23.226524 48.787682

C 0.182859 23.983873 47.815201

C 0.596492 22.998684 46.756672

O 0.264510 21.706448 47.250980

C 0.198505 22.030117 48.618511

H 48.428318 24.802340 47.469040

H 1.051195 24.468403 48.293861

H 1.672134 23.031443 46.516121

H 0.073828 23.127762 45.805710

H 1.215475 22.113235 0.167213

H 48.583797 21.218987 0.264508

O 20.419100 12.025673 46.402203

C 21.103109 13.171721 45.913269

C 22.392902 12.677415 45.319714

O 22.394857 11.268324 45.494747

C 21.548523 11.215519 46.616352

H 20.438656 13.693894 45.211174

H 21.284014 13.869909 46.748852

H 23.273951 13.107671 45.830154

H 22.523445 12.888631 44.249451

H 22.091234 11.535278 47.530155

H 21.226320 10.177036 46.779163

O 24.886925 2.345876 20.236240

C 24.441021 3.690917 20.189304

C 22.945879 3.612689 20.125254

O 22.624655 2.291605 19.698420

C 23.913469 1.837391 19.356659

H 24.835587 4.229227 21.062529

H 24.868343 4.185224 19.295053

H 22.494110 4.330924 19.425598

H 22.464285 3.775013 21.102133

H 24.160866 2.114125 18.310354

H 23.911512 0.742193 19.407507

O 26.948244 26.776630 27.693861

C 27.830759 27.885519 27.578960

C 27.768177 28.606201 28.900045

O 26.922819 27.823425 29.733667

C 27.091990 26.590839 29.079971

H 28.846750 27.511490 27.357475

H 27.528603 28.480545 26.704760

H 27.348188 29.620235 28.851641

H 28.757767 28.708876 29.381638

H 26.328283 25.885313 29.436399

H 28.081089 26.144934 29.321011

O 8.566997 48.084114 43.082378

C 8.517125 47.351696 44.303722

C 9.798117 46.563545 44.384884

O 10.510974 46.843212 43.188965

C 9.971198 48.117359 42.947437

H 8.420807 48.060154 45.143700

H 7.595985 46.750317 44.309589

H 9.676863 45.473240 44.465557

H 10.412210 46.869617 45.251263

H 10.222995 48.428806 41.924599

H 10.424434 48.867867 43.632423

O 43.728252 24.286030 2.016828

C 43.547840 24.548098 3.398050

C 42.527935 23.546284 3.873777

O 42.092300 22.835381 2.721862

C 42.427704 23.815193 1.768453

H 44.528141 24.490891 3.892356

H 43.201679 25.590981 3.520771

H 41.650311 24.021521 4.344125

H 42.909786 22.816313 4.600812

H 41.691380 24.648817 1.768942

H 42.391525 23.374670 0.765661

O 7.058165 16.930599 27.765732

C 5.817754 17.034740 27.078787

C 5.853446 16.011904 25.972343

O 7.065498 15.292201 26.143469

C 7.804269 16.332640 26.734583

H 5.014934 16.892952 27.816092

H 5.706768 18.064913 26.699379

H 5.848557 16.480295 24.970531

H 5.024713 15.291712 25.969898

H 8.095670 17.090967 25.978701

H 8.734699 15.922918 27.150661

O 1.171960 8.761101 8.703407

C 1.804633 9.914971 9.231450

C 1.800233 10.932430 8.124027

O 1.314727 10.265043 6.963312

C 1.572392 8.942004 7.369123

H 1.268768 10.213706 10.142323

H 2.832849 9.653883 9.542408

H 2.807425 11.331395 7.915744

H 1.159737 11.805655 8.312754

H 2.648034 8.690696 7.240046

H 1.005236 8.252127 6.729116

O 5.617783 14.887368 34.466000

C 5.330782 14.708421 35.843311

C 4.059569 15.463815 36.096577

O 3.796526 16.225075 34.921684

C 5.081429 16.183027 34.347191

H 5.267221 13.636691 36.062351

H 6.182006 15.073649 36.442249

H 4.114818 16.143913 36.960999

H 3.194166 14.810608 36.278946

H 5.751260 16.934509 34.820961

H 5.008090 16.449493 33.282795

O 14.256652 45.013645 1.155337

C 14.572498 46.017902 2.110702

C 15.883803 46.611462 1.670178

O 16.180094 46.035503 0.404832

C 15.544488 44.800961 0.630228

H 13.728120 46.722446 2.152261

H 14.632149 45.552933 3.110560

H 16.689068 46.362106 2.382546

H 15.906783 47.703728 1.561147

H 16.148314 44.164375 1.312283

H 15.455503 44.257271 48.572060

O 1.750851 2.007538 43.912090

C 2.359566 2.429483 45.123653

C 1.549413 3.604377 45.607204

O 0.482083 3.754967 44.684105

C 0.444436 2.427039 44.221092

H 2.351743 1.589505 45.842377

H 3.417607 2.645100 44.920258

H 2.095546 4.557787 45.656094

H 1.126001 3.442053 46.613415

H 48.709938 2.380590 43.320976

H 48.870796 1.749873 44.971107

O 24.179934 18.228212 17.177019

C 23.763369 19.269140 18.058556

C 23.205502 20.351139 17.199999

O 22.645189 19.645126 16.109688

C 23.713985 18.739632 15.945409

H 22.984993 18.848663 18.711273

H 24.605791 19.559565 18.698072

H 23.986807 21.049328 16.841125

H 22.421749 20.957411 17.673281

H 24.545652 19.239805 15.411988

H 23.394228 17.900633 15.315180

O 7.609187 44.476311 29.014454

C 6.257300 44.885059 29.149397

C 5.851001 44.469955 30.529152

O 6.849881 43.577175 31.004881

C 7.485976 43.300442 29.779627

H 6.187873 45.962654 28.947958

H 5.626583 44.394661 28.385204

H 4.866789 43.976627 30.561420

H 5.800153 45.309448 31.229786

H 6.947177 42.502510 29.224205

H 8.489257 42.914188 29.987911

O 4.047346 44.086147 22.721462

C 3.592154 45.162277 23.528193

C 4.714243 46.163113 23.587841

O 5.806998 45.572979 22.897476

C 5.052582 44.773094 22.018383

H 2.681281 45.588623 23.075445

H 3.290974 44.741314 24.497736

H 5.048671 46.436913 24.598455

H 4.439466 47.114079 23.095982

H 5.710679 44.037258 21.538746

H 4.614502 45.392563 21.209208

O 28.504992 24.044502 31.118311

C 29.880836 23.968718 30.778505

C 29.925817 23.842573 29.286299

O 28.591530 23.577576 28.859465

C 28.028776 23.206478 30.094986

H 30.400564 24.846344 31.188227

H 30.334070 23.089136 31.267923

H 30.578533 23.025574 28.940138

H 30.276377 24.754913 28.781725

H 28.248302 22.142570 30.322336

H 26.935532 23.297419 30.032892

O 40.945274 36.539547 13.843995

C 41.018127 35.715702 15.005199

C 39.814384 34.820477 14.974398

O 39.332302 34.908482 13.645980

C 39.618813 36.272591 13.459700

H 41.973003 35.173481 14.962174

H 41.073376 36.358643 15.895537

H 39.023788 35.149036 15.675520

H 40.006042 33.763901 15.205172

H 38.904491 36.906727 14.028811

H 39.490711 36.529278 12.398725

O 34.955910 34.817051 23.778036

C 34.711929 34.784294 25.177345

C 34.240116 33.391338 25.496618

O 34.312477 32.661858 24.280165

C 34.116417 33.749233 23.410362

H 35.637474 35.078629 25.691700

H 33.958492 35.552399 25.425724

H 33.197231 33.382538 25.862823

H 34.835632 32.864273 26.255922

H 33.048599 34.063614 23.408405

H 34.358437 33.445606 22.383614

O 14.207758 35.506927 32.465797

C 14.080638 34.152107 32.875519

C 14.413108 34.127663 34.343281

O 14.646327 35.477104 34.722198

C 13.897778 36.095596 33.705227

H 13.051933 33.808880 32.666744

H 14.729934 33.530682 32.244801

H 15.308336 33.548771 34.609745

H 13.589753 33.715984 34.952484

H 14.150064 37.164883 33.678337

H 12.806980 36.027145 33.914982

O 13.425962 11.914686 38.188698

C 12.236890 11.821790 37.407391

C 11.397889 12.945836 37.880184

O 12.324897 14.013654 37.956947

C 13.455298 13.311065 38.433163

H 12.491132 11.963579 36.340549

H 11.805655 10.820466 37.530602

H 10.973989 12.747332 38.878578

H 10.575512 13.235281 37.212311

H 13.555040 13.468499 39.520050

H 14.346614 13.737898 37.953037

O 29.624147 12.776666 37.629364

C 29.110771 11.472695 37.411793

C 30.023111 10.537865 38.162296

O 31.094843 11.327973 38.650246

C 30.976032 12.406059 37.755997

H 28.062511 11.461939 37.738396

H 29.094149 11.264901 36.325882

H 30.428432 9.725266 37.536469

H 29.562542 10.046981 39.028679

H 31.417048 12.153771 36.766895

H 31.547100 13.258260 38.143227

O 15.690188 20.806820 9.812296

C 15.468703 21.425802 11.077152

C 16.352198 22.634434 11.124578

O 17.299250 22.443262 10.088540

C 16.424070 21.826725 9.174245

H 14.399907 21.660488 11.173961

H 15.690677 20.685566 11.858949

H 16.889528 22.790890 12.070166

H 15.778195 23.559484 10.936341

H 17.004915 21.388645 8.349423

H 15.737125 22.575272 8.722964

O 27.551092 40.454880 46.742004

C 27.151638 40.502308 45.371052

C 25.706858 40.878780 45.368114

O 25.551376 41.655685 46.543991

C 26.359087 40.856289 47.378590

H 27.773066 41.261612 44.870388

H 27.379969 39.537163 44.897770

H 25.045826 39.991375 45.410164

H 25.385143 41.472340 44.500759

H 25.797796 39.958130 47.708618

H 26.616753 41.422958 48.283104

O 1.189563 41.009811 46.742985

C 0.578400 40.227039 45.730415

C 0.083120 41.202454 44.697308

O 0.229793 42.502510 45.259575

C 0.356429 42.135326 46.613415

H 1.307881 39.487782 45.370075

H 48.642467 39.650105 46.178272

H 47.917877 41.040615 44.435242

H 0.635607 41.184849 43.746834

H 48.251328 41.939266 47.060299

H 0.796951 42.972858 47.172264

O 11.032660 44.605881 30.968700

C 12.113191 44.891899 31.843882

C 11.560214 45.867802 32.840313

O 10.140855 45.761215 32.773819

C 10.024490 44.624458 31.950956

H 12.465219 43.957561 32.320587

H 12.961969 45.263977 31.253256

H 11.821301 46.912643 32.614433

H 11.880950 45.683475 33.875866

H 9.042234 44.651836 31.465939

H 10.060183 43.693539 32.556248

O 37.750618 14.895679 20.941763

C 36.381130 15.192948 21.168137

C 35.595913 14.254696 20.293446

O 36.540031 13.389782 19.677397

C 37.596119 13.541839 20.595114

H 36.152313 15.062405 22.241335

H 36.226631 16.257345 20.947142

H 35.016533 14.748512 19.500893

H 34.874744 13.643536 20.856201

H 38.521660 13.182477 20.125254

H 37.424992 12.920900 21.500608

O 1.798277 30.829844 33.599133

C 1.830544 32.026741 34.355015

C 1.845214 33.098469 33.319954

O 1.204227 32.567005 32.161194

C 0.868338 31.281614 32.642788

H 0.939230 32.086880 35.010178

H 2.706703 32.015003 35.019958

H 2.868543 33.378136 33.035885

H 1.337705 34.021076 33.615753

H 0.843888 30.579512 31.798410

H 48.738297 31.292860 33.076958

O 44.288074 15.965454 33.747276

C 44.548672 16.379576 35.079117

C 45.993458 16.801521 35.108452

O 46.510254 16.602039 33.799591

C 45.599873 15.615871 33.383514

H 43.821148 17.162840 35.335804

H 44.347237 15.541553 35.768993

H 46.585548 16.201607 35.818378

H 46.167027 17.850273 35.377853

H 45.887360 14.627258 33.796169

H 45.642406 15.536176 32.291737

O 29.426620 2.439262 32.486820

C 30.046579 1.346019 31.831656

C 31.517277 1.558213 32.025761

O 31.690355 2.915967 32.423260

C 30.398121 3.397072 32.144081

H 29.660816 0.409722 32.260933

H 29.755669 1.342596 30.764328

H 32.126480 1.365087 31.129068

H 31.923086 0.924074 32.824181

H 30.303268 3.678694 31.076752

H 30.229929 4.320657 32.712704

O 16.858236 15.250642 46.847614

C 16.247566 16.283747 47.606918

C 14.785182 16.238766 47.256844

O 14.612591 15.123521 46.383621

C 15.903360 15.164102 45.823307

H 16.480295 16.109688 48.665939

H 16.720360 17.249868 47.353653

H 14.472269 17.158928 46.732227

H 14.102150 16.132668 48.111980

H 15.989412 16.005547 45.104584

H 16.090620 14.247361 45.247841

O 24.733889 26.738495 35.046848

C 25.141655 27.483622 36.197784

C 26.632397 27.452328 36.213432

O 26.992735 27.305162 34.850788

C 25.991411 26.376686 34.512939

H 24.757359 28.510368 36.089241

H 24.667885 27.052387 37.091545

H 27.021093 26.599640 36.803566

H 27.111546 28.356846 36.614841

H 26.277435 25.366562 34.869366

H 25.909271 26.314106 33.418716

O 17.187775 36.090710 16.183517

C 17.840004 34.826832 16.192806

C 19.150331 35.016045 15.476037

O 19.158644 36.357174 15.011556

C 18.321110 36.903797 15.999680

H 17.968103 34.492889 17.236668

H 17.166262 34.096859 15.721969

H 19.303366 34.357948 14.610146

H 20.019646 34.858612 16.140003

H 18.001350 37.905609 15.683343

H 18.868708 37.025539 16.956022

O 15.613915 4.064458 17.153061

C 15.394386 4.052235 18.553841

C 15.043825 5.469637 18.909779

O 14.732867 6.137513 17.689415

C 14.622859 5.014445 16.846992

H 14.581299 3.341823 18.790970

H 16.292547 3.655715 19.047657

H 15.874024 6.016259 19.377684

H 14.185757 5.560578 19.593300

H 14.744601 5.337138 15.806553

H 13.610778 4.564143 16.923754

O 14.540718 3.178032 29.314655

C 14.710377 1.815390 28.948938

C 15.849579 1.294192 29.785982

O 16.298903 2.391836 30.564846

C 15.866691 3.420540 29.713133

H 13.749145 1.301526 29.092684

H 14.929417 1.763563 27.867430

H 16.693956 0.932875 29.176779

H 15.593870 0.468393 30.466572

H 16.539455 3.517837 28.834038

H 15.908251 4.376395 30.249487

O 9.568810 39.809006 4.419420

C 10.023024 38.937737 5.450080

C 11.525989 38.992496 5.423189

O 11.865304 40.018757 4.501560

C 10.751038 39.863277 3.658159

H 9.563432 39.259941 6.396156

H 9.639215 37.923702 5.263309

H 11.966023 38.036152 5.083873

H 12.002693 39.225227 6.385400

H 10.868869 38.959740 3.025975

H 10.688944 40.720367 2.974149

O 46.513187 31.915266 19.972219

C 46.072174 31.511410 18.689274

C 46.187561 30.016266 18.696608

O 46.254055 29.613880 20.067560

C 45.953854 30.844999 20.689966

H 45.028313 31.846813 18.540638

H 46.678936 32.025761 17.929480

H 47.101368 29.656416 18.197901

H 45.341225 29.503870 18.212568

H 46.361130 30.841578 21.710848

H 44.857189 30.976032 20.784328

O 30.264154 9.221182 42.865295

C 28.886354 9.355637 42.545536

C 28.718163 10.734414 41.963223

O 30.013334 11.321617 41.937309

C 30.779974 10.141833 41.938778

H 28.600821 8.566997 41.829746

H 28.307955 9.149798 43.457878

H 28.056156 11.403756 42.531845

H 28.315287 10.712901 40.935986

H 31.813566 10.388741 42.219421

H 30.816641 9.693486 40.923275

O 16.834768 0.118321 40.164455

C 17.269915 0.821399 39.007652

C 18.445297 1.649154 39.440353

O 18.666782 1.331840 40.805927

C 18.088381 0.050360 40.801533

H 17.533447 0.100230 38.218521

H 16.424070 1.394912 38.612110

H 18.295685 2.731641 39.352348

H 19.361061 1.436470 38.868797

H 17.954903 48.604332 41.838058

H 18.754299 48.201454 40.306244

O 0.937767 37.941792 4.251718

C 0.565689 36.739029 4.905415

C 48.336887 37.106701 5.838289

O 48.067001 38.486454 5.617294

C 48.574505 38.571041 4.309412

H 0.258644 35.991455 4.154910

H 1.460430 36.319038 5.381630

H 48.567665 36.966381 6.900730

H 47.416725 36.524876 5.659831

H 48.689896 39.625656 4.026322

H 47.865074 38.125626 3.579442

O 32.796310 31.849747 38.252747

C 33.084290 32.669682 37.124790

C 33.236835 31.743162 35.947941

O 32.815380 30.466082 36.400200

C 33.221680 30.610315 37.738396

H 32.265335 33.397205 37.023582

H 33.994186 33.257862 37.331608

H 34.285587 31.677645 35.603249

H 32.640831 32.008648 35.062984

H 34.324703 30.505684 37.822002

H 32.780174 29.806026 38.343689

O 40.832821 8.731277 1.920020

C 42.109413 8.810971 2.544381

C 41.869350 9.024144 4.013610

O 40.463680 8.990408 4.195980

C 40.061783 9.378128 2.905699

H 42.655056 7.886409 2.307251

H 42.682926 9.631882 2.082344

H 42.257557 9.993199 4.371994

H 42.310852 8.259949 4.669262

H 40.137566 10.478705 2.793735

H 39.003742 9.115085 2.765377

O 31.804276 20.813665 11.063463

C 31.076262 20.991634 12.270626

C 31.373041 22.399746 12.713594

O 32.163151 22.974237 11.680001

C 31.730936 22.149904 10.627828

H 30.005999 20.825399 12.060876

H 31.364241 20.200548 12.979083

H 31.939711 22.479931 13.650381

H 30.464615 23.011395 12.853917

H 32.395882 22.287292 9.764380

H 30.704189 22.418325 10.288511

O 2.119503 16.059818 5.829489

C 2.445618 14.796916 6.394689

C 1.494653 13.801459 5.785485

O 0.712857 14.521161 4.839409

C 1.603684 15.586047 4.608146

H 3.497302 14.559786 6.162448

H 2.391836 14.884923 7.488421

H 0.805753 13.323776 6.496387

H 2.018295 12.976149 5.273088

H 1.066352 16.398155 4.099172

H 2.427039 15.278510 3.931959

O 18.936182 45.632137 3.026464

C 19.683264 45.132454 1.928821

C 19.705753 46.242809 0.912829

O 19.109261 47.375656 1.530833

C 19.296032 46.983047 2.869519

H 20.699255 44.859146 2.272537

H 19.216337 44.199089 1.586571

H 19.132242 46.036972 -0.000488

H 19.132242 46.036972 48.892311

H 20.730547 46.498520 0.591114

H 18.660427 47.606430 3.514414

H 20.347717 47.144882 3.190744

O 31.430735 40.763393 18.594910

C 30.357052 39.940529 19.065258

C 29.928259 39.078060 17.925077

O 30.279800 39.823673 16.775608

C 31.506519 40.319935 17.253780

H 29.544941 40.604004 19.396263

H 30.690008 39.378750 19.946796

H 28.850174 38.861950 17.894764

H 30.450436 38.102646 17.919701

H 31.821878 41.158447 16.617683

H 32.287338 39.538628 17.167240

O 39.937595 36.747829 41.615105

C 39.223270 37.324764 42.694656

C 40.193794 38.262039 43.351780

O 41.485050 37.930058 42.845249

C 41.183384 36.683292 42.261959

H 38.309464 37.792179 42.303028

H 38.892265 36.521942 43.380138

H 40.205036 38.182343 44.449909

H 40.010445 39.321545 43.121983

H 41.189739 35.878025 43.029575

H 41.962246 36.426601 41.531498

O 44.729576 26.049105 47.063232

C 43.514591 25.390520 47.406944

C 42.479042 26.456871 47.627453

O 43.197277 27.677725 47.620609

C 44.196644 27.302717 46.708759

H 43.251549 24.687929 46.601192

H 43.715542 24.779360 48.296307

H 41.929977 26.385000 48.577442

H 41.709957 26.479851 46.831966

H 44.992619 28.057131 46.724892

H 43.803551 27.279737 45.673206

O 8.216435 26.756096 6.684624

C 8.979651 25.701477 6.118934

C 8.191500 24.437111 6.343352

O 7.017095 24.814562 7.047897

C 7.499667 26.003147 7.629721

H 9.163977 25.949852 5.063827

H 9.971686 25.676054 6.604928

H 8.743988 23.690027 6.940333

H 7.880053 23.916891 5.425634

H 8.135762 25.782152 8.511747

H 6.647465 26.595726 7.990551

O 35.415989 26.649021 3.662071

C 34.885502 26.938953 4.946485

C 33.630421 27.742752 4.723044

O 33.462719 27.829781 3.313465

C 34.209312 26.697914 2.942858

H 34.692375 25.991901 5.479416

H 35.672676 27.449396 5.512174

H 33.653893 28.766567 5.126410

H 32.737152 27.266047 5.164546

H 34.436668 26.756582 1.869172

H 33.620644 25.769928 3.107626

O 6.547724 39.335236 45.225349

C 5.370874 39.304432 46.032570

C 5.357184 37.976013 46.720493

O 6.206941 37.140926 45.950432

C 7.155461 38.121227 45.604759

H 5.389453 40.156143 46.727337

H 4.505472 39.463333 45.371540

H 4.370039 37.500778 46.789921

H 5.743926 38.054245 47.754086

H 7.773466 37.752575 44.773094

H 7.841916 38.317287 46.447670

O 21.278635 23.990231 20.997013

C 21.183784 25.409098 21.050795

C 19.898392 25.728371 21.764629

O 19.250563 24.485025 21.970469

C 20.409809 23.695894 22.065321

H 22.065809 25.806107 21.579327

H 21.244900 25.795841 20.023069

H 19.211937 26.391844 21.220942

H 20.075384 26.211428 22.742975

H 20.125254 22.635899 22.015938

H 20.918295 23.847952 23.041710

O 15.924873 42.826180 44.370705

C 15.216907 42.813957 45.610138

C 15.190503 41.396553 46.075596

O 15.214951 40.648987 44.872833

C 16.172270 41.443981 44.208382

H 15.690677 43.524372 46.300991

H 14.202869 43.185543 45.406254

H 14.297722 41.116890 46.653511

H 16.068619 41.140846 46.699467

H 16.165426 41.215652 43.135181

H 17.186796 41.195606 44.578499

O 20.296867 27.816580 7.882497

C 20.448925 29.124462 7.345166

C 20.918295 28.951384 5.926296

O 21.048840 27.551582 5.722413

C 20.095919 27.132080 6.668978

H 19.479870 29.649084 7.396992

H 21.136356 29.681839 7.996906

H 21.880995 29.424175 5.690633

H 20.202017 29.365992 5.196327

H 20.207394 26.051550 6.839125

H 19.063303 27.292940 6.290059

O 31.796944 31.063063 24.969551

C 31.878595 30.439678 26.240767

C 30.496881 29.916037 26.517988

O 29.631481 30.491505 25.544041

C 30.474394 31.526077 25.096674

H 32.670658 29.677929 26.206051

H 32.200798 31.181871 26.996161

H 30.128721 30.175167 27.525179

H 30.414745 28.824751 26.424112

H 30.430387 32.385612 25.796820

H 30.117964 31.891794 24.124195

O 5.696011 43.224171 18.515215

C 5.375763 44.161446 17.493843

C 6.180538 45.398918 17.774977

O 6.768230 45.192593 19.050100

C 6.903664 43.796215 18.946938

H 5.616805 43.715542 16.515009

H 4.287410 44.316921 17.508511

H 5.603115 46.334240 17.796978

H 6.980914 45.552933 17.026428

H 7.171107 43.379158 19.928217

H 7.730930 43.532192 18.252661

O 30.841578 21.906418 20.851313

C 31.857569 22.883785 21.020481

C 31.166714 24.133974 21.491808

O 29.775717 23.843552 21.527498

C 29.764959 22.782578 20.609781

H 32.605141 22.481398 21.719648

H 32.382191 23.031443 20.060226

H 31.333441 24.986664 20.815620

H 31.468874 24.472313 22.492643

H 29.796740 23.167854 19.567877

H 28.821327 22.231556 20.726147

O 37.748173 39.762558 45.704010

C 37.658699 40.713036 44.651836

C 36.558613 40.246105 43.742920

O 36.083862 39.025253 44.289543

C 36.430027 39.273632 45.629696

H 37.453350 41.709469 45.081604

H 38.640957 40.776104 44.168289

H 36.862724 40.063736 42.702484

H 35.729389 40.971188 43.698429

H 36.357662 38.339291 46.203693

H 35.716190 39.990887 46.087330

O 10.577957 41.082176 36.905750

C 9.551208 40.218727 37.363876

C 8.943959 40.912029 38.555885

O 9.774649 42.035583 38.835552

C 10.935853 41.627331 38.152027

H 8.867198 40.026089 36.526344

H 9.990754 39.236473 37.611763

H 8.886755 40.273487 39.452576

H 7.924545 41.288990 38.396492

H 11.520611 40.897850 38.752922

H 11.585149 42.500557 37.993618

O 15.993323 39.106415 42.100613

C 15.808998 37.900719 42.826180

C 14.414086 37.432816 42.518158

O 13.883600 38.367157 41.591148

C 15.102008 38.818439 41.054794

H 16.578571 37.172218 42.513268

H 15.991367 38.107048 43.887157

H 13.741322 37.392723 43.386982

H 14.393063 36.426113 42.065895

H 14.924038 39.726864 40.462215

H 15.537642 38.063046 40.369316

O 22.522957 37.663589 37.429882

C 21.503054 37.211334 36.556168

C 21.059595 35.880959 37.099857

O 22.009094 35.513287 38.095314

C 23.048555 36.394821 37.738888

H 20.724192 37.984325 36.499943

H 21.913752 37.118435 35.534798

H 21.023415 35.093296 36.328815

H 20.067072 35.894157 37.573627

H 23.630379 35.991455 36.880817

H 23.744787 36.487228 38.583752

O 48.567665 27.082699 45.666851

C 47.549713 28.008240 45.312866

C 47.685638 29.170910 46.256012

O 48.746609 28.837952 47.137547

C 0.562757 27.999439 46.258457

H 47.684170 28.300129 44.260696

H 46.584572 27.488022 45.348560

H 46.793343 29.391905 46.857391

H 47.937435 30.109653 45.733345

H 1.324993 27.443527 46.819744

H 1.099111 28.594954 45.485950

O 48.463520 14.032723 35.735748

C 0.338338 15.042359 35.098183

C 0.482572 16.151737 36.106842

O 48.861996 15.651564 37.336987

C 0.004400 14.273764 37.052433

H 1.313261 14.617481 34.795536

H 48.723141 15.323981 34.164822

H 48.818970 17.068476 35.861404

H 1.525455 16.469051 36.261345

H 48.233234 13.748167 37.753551

H 1.027727 13.868443 37.208397

O 1.144580 9.882702 35.098183

C 48.748074 10.468437 35.270779

C 47.752617 9.476402 34.748112

O 48.455700 8.247726 34.684551

C 0.808687 8.770879 34.300743

H 48.625843 10.686988 36.342506

H 48.725098 11.437493 34.749092

H 47.381031 9.748735 33.741409

H 46.860325 9.331680 35.373940

H 0.803798 9.045168 33.225101

H 1.578260 7.997396 34.428352

O 2.901788 24.063570 15.588491

C 3.839551 24.686462 16.451939

C 3.241592 26.016346 16.792721

O 1.850592 25.927853 16.486162

C 1.747429 24.546143 16.231920

H 3.991608 24.062101 17.353521

H 4.810563 24.734869 15.942475

H 3.661093 26.849970 16.211386

H 3.367736 26.290148 17.850273

H 0.873714 24.367193 15.589958

H 1.569459 23.991207 17.175550

O 43.703808 12.382589 8.393916

C 42.499577 13.066112 8.713675

C 42.723507 13.701718 10.060672

O 44.007431 13.269994 10.492884

C 44.090549 12.099502 9.716466

H 42.259022 13.761856 7.898632

H 41.676224 12.333208 8.728342

H 41.968601 13.399561 10.806287

H 42.718616 14.799851 10.069962

H 43.461300 11.288858 10.147700

H 45.127075 11.734272 9.724778

O 8.668694 15.005199 35.447769

C 9.132687 16.300859 35.797352

C 10.108098 16.701290 34.722687

O 10.078761 15.660852 33.753143

C 8.805593 15.146011 34.054810

H 8.266795 16.984869 35.844776

H 9.548763 16.259790 36.814812

H 11.143646 16.824989 35.068359

H 9.841632 17.653234 34.232296

H 8.690206 14.163754 33.575665

H 8.007663 15.800686 33.640202

O 32.679947 34.611214 34.281185

C 34.041122 34.924614 34.047970

C 34.023521 35.618404 32.723461

O 32.742531 36.244232 32.599766

C 32.163639 35.839890 33.823547

H 34.423466 35.574398 34.860565

H 34.636635 34.000542 34.077793

H 34.129131 34.902611 31.892773

H 34.804829 36.377220 32.589985

H 31.075285 35.756283 33.694473

H 32.344540 36.625107 34.585300

O 1.907797 28.687363 37.497353

C 0.882026 28.690294 38.481567

C 48.730476 29.666197 38.011707

O 0.218551 30.045115 36.698448

C 1.606128 29.906260 36.865170

H 0.527064 27.655235 38.591576

H 1.321083 28.974361 39.453068

H 48.698208 30.564846 38.653183

H 47.702259 29.278475 37.971123

H 2.017317 30.768728 37.430370

H 2.090656 29.909191 35.882427

O 47.052475 27.400015 39.803139

C 45.782242 27.908010 40.191349

C 45.786152 27.980371 41.691380

O 46.987446 27.350634 42.108921

C 47.759953 27.686525 40.982433

H 45.631649 28.890755 39.715622

H 45.012665 27.238178 39.784073

H 44.938839 27.474821 42.176884

H 45.775394 29.022764 42.056610

H 48.690384 27.101768 40.995636

H 48.047932 28.757767 40.998077

O 25.034090 23.695406 17.367701

C 24.492361 24.019566 16.093554

C 25.562134 23.683182 15.091251

O 26.671511 23.197187 15.834421

C 26.393312 23.862131 17.038652

H 23.550684 23.465611 15.968877

H 24.222471 25.090807 16.072529

H 25.880426 24.557875 14.496715

H 25.280510 22.910189 14.367149

H 26.660755 24.939238 16.964334

H 27.016203 23.438231 17.838539

O 25.763571 5.212950 44.276829

C 25.037516 4.510361 43.280396

C 23.734520 5.245708 43.119537

O 23.831327 6.424025 43.911602

C 25.232107 6.492964 44.036766

H 25.625692 4.503027 42.345566

H 24.952440 3.460144 43.594776

H 22.845160 4.693220 43.454941

H 23.536995 5.528309 42.072254

H 25.485861 7.154483 44.878212

H 25.682899 6.941800 43.126381

O 8.165586 37.058296 36.897930

C 8.466277 36.793297 35.532845

C 7.155461 36.464252 34.870342

O 6.172716 36.628040 35.877537

C 6.993137 36.285793 36.968334

H 8.998719 37.664082 35.124588

H 9.177178 35.949898 35.476128

H 7.119770 35.418434 34.515385

H 6.882639 37.090569 34.008854

H 7.221467 35.195480 36.980556

H 6.460694 36.501896 37.903652

O 11.140224 21.671734 33.721855

C 11.772408 22.190487 32.567493

C 12.056965 20.996035 31.715294

O 12.011495 19.858789 32.576294

C 11.932776 20.514441 33.822571

H 11.127023 22.948814 32.103500

H 12.701860 22.705816 32.868183

H 13.039220 21.042482 31.221966

H 11.308905 20.844957 30.924696

H 12.944857 20.773573 34.200516

H 11.496163 19.826031 34.559875

O 20.330605 25.236507 13.292974

C 20.851801 24.579388 14.438532

C 20.533508 23.124338 14.268386

O 19.903770 22.996729 13.000107

C 20.416656 24.157442 12.394814

H 21.938200 24.762737 14.508449

H 20.406878 25.042402 15.326914

H 19.854876 22.719017 15.030135

H 21.431181 22.486776 14.296742

H 19.828962 24.383816 11.493231

H 21.466383 24.004410 12.066743

O 45.261040 34.735401 43.868576

C 44.907059 34.404396 42.530380

C 45.098717 32.919521 42.395927

O 45.290379 32.419350 43.706741

C 45.940655 33.554642 44.215225

H 43.876884 34.749580 42.360722

H 45.543156 34.979378 41.836590

H 45.991501 32.687771 41.793076

H 44.269009 32.371922 41.932907

H 46.988914 33.610378 43.856354

H 45.971455 33.468098 45.306023

O 30.148767 12.239824 0.790597

C 31.311928 13.052911 0.772506

C 31.924063 12.934100 2.142971

O 31.008791 12.183108 2.932101

C 29.848566 12.374278 2.158128

H 31.953890 12.717505 48.838039

H 31.018082 14.090416 0.532443

H 32.090302 13.911957 2.623588

H 32.893608 12.416327 2.168885

H 29.396795 13.367780 2.366411

H 29.101973 11.617907 2.439262

O 41.897217 0.569599 22.853474

C 40.747749 0.076271 22.174353

C 41.245964 48.210747 20.974522

O 42.638435 48.473789 20.911451

C 42.848183 48.587708 22.297562

H 40.162014 48.348625 22.871073

H 40.116055 0.937277 21.921087

H 40.788330 48.507034 20.021112

H 41.083153 47.119946 21.062040

H 43.856842 0.089963 22.484333

H 42.796356 47.589806 22.786978

O 22.455975 33.487167 8.131850

C 23.492012 32.718571 8.727854

C 22.805069 31.686445 9.580055

O 21.407713 31.929443 9.471025

C 21.476652 33.290131 9.122418

H 24.130064 32.313251 7.927967

H 24.142286 33.379604 9.330214

H 23.103804 31.767120 10.640051

H 22.992817 30.645027 9.282298

H 21.692757 33.913513 10.016667

H 20.498306 33.612335 8.736654

O 34.005920 2.265203 25.925407

C 34.921192 1.917087 26.962425

C 36.267212 2.184530 26.410423

O 36.079464 3.381426 25.674099

C 34.824875 3.076335 25.103519

H 34.732468 2.557093 27.839561

H 34.736870 0.882026 27.267027

H 36.598217 1.365087 25.746458

H 37.051453 2.340498 27.164349

H 34.972042 2.554160 24.141798

H 34.297321 4.015077 24.881544

O 4.005787 9.576143 25.609560

C 4.295722 10.154546 26.872459

C 5.288245 9.231450 27.529579

O 5.400699 8.087847 26.690578

C 4.198424 8.232570 25.973810

H 4.651661 11.181783 26.704760

H 3.356491 10.248420 27.448418

H 4.979242 8.912668 28.539217

H 6.296415 9.651928 27.644478

H 3.333511 7.870763 26.563946

H 4.249273 7.610653 25.069294

O 0.358384 15.236464 23.616199

C 47.865562 15.070229 23.377115

C 47.305252 16.463182 23.337023

O 48.419518 17.355965 23.351202

C 0.518264 16.451448 22.934145

H 47.719372 14.524096 22.426638

H 47.458775 14.425332 24.166733

H 46.666710 16.706669 24.199003

H 46.698978 16.659245 22.437393

H 1.509810 16.866549 23.165897

H 0.475727 16.302326 21.833569

O 29.046722 13.317420 23.153675

C 29.188023 14.580321 23.787813

C 27.792133 15.038937 24.122240

O 26.907663 14.087481 23.543839

C 27.784313 13.556018 22.580650

H 29.859324 14.465424 24.649794

H 29.701397 15.272644 23.095491

H 27.560383 16.041239 23.725231

H 27.573093 15.090274 25.196413

H 27.870361 14.236606 21.704981

H 27.370678 12.608963 22.211020

O 20.675299 43.641224 46.011059

C 19.953640 43.682293 47.230930

C 18.524994 43.416317 46.860325

O 18.545038 42.987526 45.504040

C 19.904259 42.638920 45.404785

H 20.344294 42.911743 47.920811

H 20.160456 44.645969 47.709106

H 17.862984 44.291008 46.932198

H 18.058556 42.626698 47.470509

H 20.180504 42.544071 44.345768

H 20.105207 41.652264 45.872204

O 23.160030 33.236347 28.192078

C 22.862272 34.624905 28.026331

C 24.110506 35.368561 28.367113

O 25.146545 34.448399 28.070824

C 24.501158 33.326801 28.624289

H 22.569405 34.779896 26.976603

H 21.992960 34.885502 28.646292

H 24.144243 35.645294 29.438845

H 24.272341 36.296059 27.800446

H 24.548098 33.367382 29.731222

H 25.032625 32.412014 28.332888

O 15.793840 33.786392 28.482990

C 15.530797 34.962265 27.730530

C 16.126801 36.100975 28.522591

O 16.570747 35.545555 29.753223

C 15.760105 34.396572 29.751268

H 15.940518 34.825363 26.719915

H 14.438532 35.064938 27.596073

H 15.403187 36.904285 28.736744

H 16.990248 36.593815 28.053711

H 14.715265 34.647881 30.040224

H 16.140980 33.693005 30.501772

O 2.780045 4.950396 40.311626

C 1.399801 5.260376 40.397186

C 1.033105 5.078495 41.839523

O 2.258847 4.998800 42.564606

C 3.139406 5.464259 41.570126

H 1.247744 6.303749 40.074005

H 0.846334 4.634059 39.683838

H 0.463015 4.159310 42.034119

H 0.430257 5.908206 42.242401

H 4.158333 5.137167 41.811165

H 3.154563 6.575593 41.554482

O 20.382431 44.711975 25.789974

C 21.376909 44.089085 24.974932

C 21.541189 42.702969 25.497595

O 21.205296 42.794891 26.872459

C 20.130632 43.695496 26.738983

H 21.059595 44.134064 23.923735

H 22.302450 44.680195 25.057560

H 22.553759 42.285915 25.405188

H 20.855713 42.004784 24.982754

H 19.934572 44.165844 27.712929

H 19.212915 43.144962 26.455894

O 27.727596 23.921780 45.350029

C 28.168121 24.310966 46.636887

C 26.987358 24.953419 47.306717

O 25.991411 25.126987 46.300014

C 26.804499 24.965641 45.160812

H 28.581753 23.427475 47.139503

H 28.999296 25.028713 46.527855

H 27.255291 25.929808 47.746754

H 26.535101 24.360348 48.114426

H 27.312008 25.926874 44.941280

H 26.186983 24.713842 44.286606

O 34.461601 2.356633 35.045872

C 34.478222 1.007681 35.493237

C 34.248917 0.152057 34.279232

O 34.240116 1.035550 33.168388

C 34.951508 2.100435 33.749233

H 35.458523 0.800375 35.958210

H 33.727718 0.892294 36.287746

H 33.303818 48.487968 34.281185

H 35.035114 48.287994 34.130108

H 34.824875 2.992239 33.121449

H 36.041328 1.883351 33.778076

O 48.886444 29.811407 42.911743

C 1.137246 30.610804 42.675102

C 0.926028 31.867350 43.474499

O 48.485523 31.811611 43.962452

C 47.973125 30.870424 43.052555

H 2.029050 30.027512 42.939121

H 1.210096 30.812731 41.590172

H 1.056085 32.785557 42.879475

H 1.588038 31.977846 44.341370

H 47.757507 31.341263 42.068340

H 47.018250 30.481728 43.434410

O 16.102356 33.734077 45.206284

C 17.455708 33.440231 45.527020

C 18.259016 34.633213 45.087471

O 17.315384 35.592491 44.632771

C 16.346819 34.675262 44.193222

H 17.512423 33.207500 46.600704

H 17.753464 32.520069 44.994087

H 18.936182 34.376038 44.253361

H 18.886801 35.095253 45.862911

H 16.663645 34.177048 43.250568

H 15.415899 35.211128 43.969292

O 24.162821 0.286023 0.433679

C 23.091581 1.216453 0.302157

C 21.934776 0.452258 48.643444

O 22.521490 48.269905 47.930588

C 23.557039 47.998550 48.847328

H 22.887697 1.688757 1.271213

H 23.444586 2.020739 48.535881

H 21.283525 1.016970 47.963348

H 21.287926 0.058671 0.557867

H 24.323679 47.382992 48.355957

H 23.162964 47.414280 0.809176

O 10.976922 5.586002 30.692453

C 10.439590 4.956752 31.843882

C 11.633063 4.526495 32.645233

O 12.757109 4.595923 31.768585

C 12.077010 4.724511 30.540398

H 9.758513 5.657875 32.344540

H 9.817675 4.092328 31.540255

H 11.547501 3.499747 33.033440

H 11.843303 5.171880 33.511124

H 11.752851 3.729054 30.161970

H 12.763465 5.138144 29.789894

O 8.131850 15.161656 46.651554

C 8.459921 13.936893 47.285202

C 7.767110 12.877385 46.483852

O 6.822012 13.537928 45.645340

C 6.833258 14.814518 46.239388

H 9.555609 13.842529 47.315029

H 8.122071 13.962317 48.337868

H 7.242002 12.130793 47.098434

H 8.455521 12.322941 45.832108

H 6.123823 14.847766 47.090611

H 6.483185 15.554755 45.507462

O 9.538985 26.901796 46.183163

C 8.358713 27.279249 46.867172

C 8.345023 28.782701 46.838814

O 9.352704 29.189978 45.919628

C 9.556097 27.963747 45.260555

H 7.491354 26.837257 46.345486

H 8.374847 26.827967 47.869965

H 8.572374 29.251585 47.806400

H 7.384768 29.201223 46.505363

H 10.532976 27.980371 44.759892

H 8.787014 27.813646 44.473377

O 46.521988 9.421153 23.808838

C 46.646175 8.314220 22.920944

C 47.648479 8.704874 21.874640

O 48.249859 9.901280 22.342052

C 47.125813 10.416121 23.015797

H 46.942467 7.440017 23.514503

H 45.657562 8.069267 22.503889

H 47.176174 8.897511 20.894339

H 48.436142 7.960237 21.693735

H 46.393402 10.833178 22.291695

H 47.439705 11.246322 23.665092

O 14.344169 7.095812 29.299988

C 14.910837 6.446027 28.165186

C 15.340606 7.521180 27.211288

O 15.363585 8.710252 27.981350

C 14.254207 8.405161 28.789547

H 15.752770 5.839267 28.526014

H 14.173533 5.744904 27.743729

H 14.636059 7.639500 26.366909

H 16.333130 7.372056 26.762453

H 13.319376 8.525927 28.207722

H 14.204824 9.114106 29.627081

O 47.978016 43.397247 9.496449

C 47.121902 44.507114 9.732111

C 47.827915 45.703033 9.147353

O 0.119787 45.195038 8.545484

C 0.290421 44.112061 9.429955

H 46.135735 44.286121 9.297455

H 46.952244 44.610279 10.818509

H 48.101715 46.439846 9.926705

H 47.258312 46.261879 8.391471

H 0.609694 44.455780 10.438124

H 1.085420 43.450050 9.058369

O 45.607204 15.848600 38.011707

C 45.273754 15.045293 39.128418

C 46.380199 15.278999 40.115074

O 47.019718 16.491053 39.728821

C 46.113243 16.934509 38.746567

H 44.294922 15.365051 39.531784

H 45.145164 14.008765 38.786171

H 47.147816 14.490849 40.112633

H 46.036484 15.377764 41.155514

H 46.632973 17.627810 38.070377

H 45.279621 17.500200 39.211048

O 3.858131 7.741686 3.734432

C 4.162733 6.388333 3.440097

C 2.850939 5.767883 3.039665

O 1.839347 6.734494 3.322755

C 2.568339 7.514823 4.242428

H 4.954796 6.364865 2.676881

H 4.592501 5.906250 4.339236

H 2.629944 4.840387 3.596066

H 2.781023 5.509730 1.973802

H 2.609898 7.023939 5.241308

H 2.057898 8.478500 4.385195

O 29.405596 31.824814 30.209883

C 29.585522 32.902412 29.306833

C 28.284483 33.024155 28.561707

O 27.369211 32.135773 29.195356

C 28.028776 32.004738 30.431856

H 29.829985 33.818172 29.877411

H 30.465103 32.685814 28.682961

H 28.344135 32.743019 27.500734

H 27.868406 34.045525 28.588108

H 27.630299 31.129068 30.964300

H 27.839561 32.888721 31.080175

O 22.430059 21.372997 20.769173

C 21.627729 20.200548 20.886515

C 22.530779 19.120018 21.421892

O 23.828394 19.691574 21.481541

C 23.426006 21.019991 21.700581

H 20.774061 20.409809 21.554880

H 21.186228 19.987865 19.901815

H 22.582117 18.201811 20.818554

H 22.239866 18.797325 22.435928

H 24.293365 21.682001 21.576881

H 23.065668 21.162758 22.742975

O 47.404015 44.395149 42.014069

C 46.117153 44.372173 41.404865

C 46.155293 43.287727 40.364429

O 47.515980 42.893654 40.268600

C 47.862629 43.127850 41.610706

H 45.356873 44.194202 42.180305

H 45.912296 45.378387 41.016171

H 45.805222 43.579128 39.364082

H 45.544621 42.413525 40.657787

H 0.064049 43.096558 41.713383

H 47.457306 42.330406 42.268314

O 16.892952 2.431439 0.609693

C 18.050734 2.618698 1.413491

C 19.120508 3.149674 0.495284

O 18.525482 3.231325 48.099270

C 17.193153 3.391694 48.521214

H 17.813114 3.320799 2.234401

H 18.285908 1.656977 1.889218

H 20.021112 2.524335 0.422434

H 19.474491 4.151977 0.796464

H 16.521366 3.247949 47.664124

H 17.012739 4.421376 0.007823

O 46.956642 11.368065 41.638088

C 46.315659 11.594439 40.380074

C 45.168633 10.651297 40.321404

O 44.739357 10.585780 41.672310

C 46.017414 10.517330 42.263424

H 45.945053 12.629010 40.374207

H 47.055897 11.504476 39.573833

H 45.461014 9.648016 39.959595

H 44.333546 10.984256 39.687263

H 46.390465 9.475913 42.225288

H 45.939186 10.789174 43.325375

O 41.663021 5.658853 2.995173

C 42.075188 4.531874 2.238801

C 43.247639 3.944182 2.976594

O 43.414360 4.729401 4.150999

C 42.089855 5.192415 4.251718

H 41.232765 3.819017 2.161062

H 42.289337 4.866789 1.214008

H 44.196644 3.952494 2.420682

H 43.071625 2.892498 3.265550

H 42.048298 6.016259 4.977287

H 41.413666 4.395952 4.635037

O 13.276839 13.443075 31.711382

C 14.636059 13.142384 31.415092

C 14.694242 11.666311 31.134933

O 13.347244 11.217964 31.102175

C 12.826048 12.146438 32.019894

H 14.942618 13.782880 30.575602

H 15.264821 13.435741 32.273159

H 15.238907 11.127512 31.928953

H 15.180726 11.378332 30.192770

H 13.106693 11.870683 33.059845

H 11.727916 12.129814 31.968557

O 48.746609 45.728947 28.991964

C 48.709938 44.402485 29.504360

C 47.486153 44.318878 30.381006

O 46.904331 45.613068 30.370251

C 47.375168 46.024750 29.111752

H 48.677670 43.694031 28.659004

H 0.770548 44.212780 30.019690

H 47.678303 44.048500 31.427315

H 46.745430 43.587440 30.010889

H 47.227020 47.108700 29.016897

H 46.794319 45.545597 28.293283

O 4.568054 2.845561 3.608778

C 4.017033 1.709781 4.252696

C 4.210648 0.604804 3.275329

O 5.393854 0.922607 2.539003

C 5.701389 2.196754 3.075357

H 4.559254 1.505409 5.192904

H 2.969260 1.913175 4.516717

H 3.371648 0.553955 2.564427

H 4.322124 48.502148 3.727587

H 6.135068 2.822581 2.283294

H 6.481229 2.095056 3.855686

O 20.492439 46.737118 21.486917

C 21.177916 47.931568 21.840414

C 20.293446 48.627312 22.840271

O 19.093126 47.864582 22.878407

C 19.706732 46.620750 22.644211

H 22.166529 47.670479 22.258936

H 21.374954 48.497257 20.917807

H 20.053871 0.782286 22.611452

H 20.747171 48.641979 23.848440

H 18.942049 45.848736 22.479443

H 20.308601 46.292683 23.521347

O 16.549725 26.335619 34.530052

C 17.308050 25.559198 35.440926

C 18.548950 25.300068 34.677216

O 18.105494 24.993999 33.356625

C 16.816679 25.578270 33.369827

H 17.433216 26.120979 36.376244

H 16.783432 24.614103 35.676586

H 19.179668 24.481604 35.045380

H 19.162066 26.215828 34.634682

H 16.075953 24.765669 33.279861

H 16.700802 26.226585 32.490730

O 35.027290 9.336569 19.007565

C 35.583691 9.926705 20.173658

C 37.076878 9.921816 19.975153

O 37.307163 9.367372 18.686829

C 36.059418 9.678329 18.118204

H 35.176414 10.947587 20.286110

H 35.229218 9.361505 21.046883

H 37.641590 9.329723 20.709524

H 37.513489 10.933408 20.007912

H 35.939629 9.110684 17.184841

H 36.001724 10.755927 17.849293

O 2.104346 17.173107 16.299393

C 2.400636 17.311474 14.916705

C 1.437937 18.335289 14.376438

O 0.629250 18.735231 15.474082

C 0.755394 17.570604 16.252943

H 3.462099 17.571583 14.821852

H 2.294539 16.329706 14.424355

H 0.788641 17.925077 13.582419

H 1.902419 19.237362 13.951560

H 0.114898 16.754095 15.852511

H 0.403366 17.777422 17.273336

O 44.133087 39.146999 21.518700

C 45.467369 39.246250 21.982691

C 45.384743 40.325314 23.000639

O 44.478268 41.289478 22.469175

C 43.869553 40.532619 21.440960

H 45.791527 38.267414 22.362589

H 46.144535 39.516628 21.150536

H 46.330818 40.825001 23.245104

H 44.957417 39.933681 23.939871

H 44.231850 40.919849 20.470438

H 42.782665 40.692989 21.471762

O 40.267132 16.967268 14.152998

C 39.275097 17.833160 14.681530

C 39.964973 19.142508 14.951418

O 41.344242 18.939116 14.667840

C 41.373085 17.535402 14.809628

H 38.439030 17.879608 13.970140

H 38.858532 17.386280 15.598759

H 39.855942 19.475958 15.998214

H 39.615391 19.976131 14.328034

H 41.386776 17.244490 15.881360

H 42.300095 17.143770 14.365682

O 32.481441 37.677769 8.035532

C 31.963667 36.632439 7.222933

C 30.633785 37.138969 6.736938

O 30.433813 38.389160 7.388191

C 31.253256 38.170120 8.505391

H 32.705860 36.403133 6.445048

H 31.867350 35.707390 7.821870

H 29.791359 36.465717 6.974069

H 30.581957 37.317429 5.656897

H 30.777040 37.474865 9.231938

H 31.418024 39.120598 9.033923

O 21.087465 29.287766 20.402964

C 21.179871 30.711521 20.403454

C 21.427269 31.130533 21.826725

O 21.086487 29.999643 22.608519

C 21.576881 29.044277 21.700092

H 21.975847 31.018082 19.706732

H 20.238197 31.103155 19.992266

H 20.835667 31.989582 22.171907

H 22.487753 31.395535 22.007627

H 21.249788 28.048334 22.017895

H 22.686258 29.031565 21.707426

O 26.772230 45.577869 43.676426

C 26.593285 44.475822 44.548672

C 25.555777 44.927105 45.536797

O 25.388563 46.329350 45.346607

C 26.577148 46.584572 44.638149

H 27.556471 44.230385 45.037113

H 26.322906 43.594288 43.950226

H 25.857445 44.744736 46.579193

H 24.573032 44.449909 45.410164

H 27.451839 46.654976 45.320202

H 26.498920 47.558025 44.135040

O 8.345512 31.950956 3.254305

C 7.347610 31.992516 4.271275

C 7.908411 32.812935 5.388964

O 8.905334 33.612335 4.783183

C 9.396708 32.622253 3.909957

H 6.439181 32.433037 3.831728

H 7.098257 30.963320 4.561698

H 8.364091 32.182709 6.175650

H 7.181863 33.462719 5.895494

H 10.025958 31.891794 4.459512

H 10.041114 33.091137 3.156030

O 16.158581 33.107269 21.786142

C 15.835889 34.398041 22.276537

C 15.043337 34.141354 23.514992

O 14.430710 32.862804 23.348267

C 14.920615 32.501488 22.074612

H 16.764853 34.965687 22.434462

H 15.246243 34.951996 21.520164

H 14.262030 34.888435 23.713497

H 15.682365 34.092949 24.411198

H 14.175979 32.771866 21.299660

H 15.035514 31.409222 22.041363

O 48.728519 37.083233 25.163168

C 47.470509 36.914063 25.799751

C 46.595814 38.028820 25.298601

O 47.440685 38.885910 24.537342

C 48.688915 38.482544 25.039957

H 47.108700 35.899540 25.580225

H 47.612785 36.960510 26.893972

H 46.126934 38.612110 26.109243

H 45.769527 37.704170 24.649303

H 0.003911 38.968540 26.016836

H 0.582801 38.809639 24.345680

O 36.025681 1.545990 19.412886

C 36.826546 2.720884 19.439777

C 37.529133 2.793246 18.112827

O 36.975182 1.748407 17.331032

C 36.686222 0.856602 18.380270

H 36.163559 3.571619 19.655394

H 37.526203 2.650968 20.287088

H 38.619442 2.633855 18.202299

H 37.402992 3.739321 17.565716

H 37.612740 0.371096 18.757233

H 36.032528 0.054760 18.006239

O 32.059986 33.302841 44.109127

C 33.084290 33.254436 45.086987

C 32.491711 32.541092 46.265789

O 31.102175 32.396858 45.993946

C 30.979944 33.392803 45.005821

H 33.972672 32.774799 44.654282

H 33.386936 34.286564 45.344646

H 32.630566 33.096512 47.205997

H 32.909744 31.544167 46.455982

H 30.930075 34.403908 45.464924

H 30.034847 33.250038 44.462135

O 30.239220 8.506370 30.659698

C 31.631197 8.611488 30.400564

C 31.714804 8.863775 28.930357

O 30.465591 9.431910 28.538237

C 29.856388 9.558053 29.801140

H 32.063408 9.450489 30.978968

H 32.141636 7.699149 30.743792

H 31.841925 7.929434 28.367603

H 32.527401 9.542408 28.632601

H 28.766079 9.541919 29.669617

H 30.110630 10.539332 30.252909

O 5.415367 18.343601 37.193241

C 4.769493 19.129309 38.190163

C 5.007601 18.430141 39.499516

O 5.532709 17.162840 39.150421

C 6.264635 17.606787 38.038109

H 5.183125 20.152145 38.167187

H 3.712430 19.218294 37.907566

H 4.126552 18.268795 40.132675

H 5.738059 18.975784 40.127785

H 6.666045 16.744806 37.493931

H 7.145194 18.209635 38.352001

O 22.754709 27.209330 17.455708

C 23.633801 26.715027 18.452143

C 24.941683 27.413214 18.217947

O 24.671795 28.479568 17.316362

C 23.269550 28.518681 17.426373

H 23.228970 26.949223 19.454445

H 23.660204 25.619337 18.382227

H 25.717613 26.785921 17.759331

H 25.378298 27.820004 19.143976

H 22.862272 29.063835 16.562435

H 22.955170 29.078991 18.332844

O 17.762266 47.338009 18.740610

C 16.560968 46.986469 18.062956

C 16.292059 48.094379 17.079721

O 17.364767 0.123208 17.213198

C 18.325998 48.119804 17.709949

H 16.692980 46.008614 17.566204

H 15.773306 46.832947 18.812971

H 15.352340 48.641491 17.242535

H 16.258322 47.730129 16.037817

H 19.175756 48.690872 18.110382

H 18.727898 47.478329 16.894417

O 43.093136 26.391356 28.383736

C 41.945133 26.028082 29.134729

C 41.907974 24.524630 29.126907

O 42.932766 24.097305 28.234125

C 43.077492 25.293222 27.508556

H 42.006248 26.502832 30.122364

H 41.047951 26.460295 28.666338

H 40.945274 24.120773 28.775856

H 42.086433 24.060637 30.106718

H 42.272224 25.396385 26.749741

H 44.028454 25.264378 26.960466

O 30.566311 27.395124 15.071694

C 29.230072 26.968290 15.281445

C 29.191446 25.531330 14.830653

O 30.449945 25.259487 14.222426

C 30.843044 26.584969 13.958405

H 28.978273 27.137459 16.338995

H 28.547527 27.613186 14.696198

H 28.395962 25.325981 14.099706

H 29.043791 24.801849 15.638851

H 30.334558 26.975622 13.049976

H 31.924063 26.604527 13.760879

O 27.997972 5.366474 1.098621

C 27.272892 4.510361 1.970380

C 27.819023 4.764114 3.348668

O 28.655582 5.911628 3.245015

C 28.192078 6.418158 2.016828

H 26.195295 4.747980 1.903886

H 27.371166 3.478234 1.607106

H 28.425297 3.945649 3.765234

H 27.025494 4.958219 4.087927

H 28.938671 7.120258 1.618352

H 27.252356 6.994604 2.158617

O 45.510395 41.781342 43.677895

C 45.295265 42.876541 44.559429

C 45.367630 42.318184 45.952877

O 45.699120 40.946251 45.805710

C 45.110939 40.746769 44.542809

H 46.048218 43.646603 44.338924

H 44.317410 43.331245 44.328655

H 44.409817 42.399837 46.497051

H 46.122532 42.788044 46.598259

H 44.002541 40.699833 44.618591

H 45.443901 39.782116 44.132595

O 30.501772 25.495640 36.514610

C 31.364731 26.180138 37.412769

C 31.605284 27.545715 36.828991

O 31.039595 27.506111 35.530399

C 29.994267 26.622618 35.846245

H 30.890961 26.213873 38.406273

H 32.268761 25.571424 37.513981

H 32.659412 27.840536 36.739029

H 31.112932 28.342178 37.419128

H 29.497025 26.295525 34.925594

H 29.227627 27.133549 36.466694

O 41.819477 3.606333 28.900045

C 40.434345 3.815105 28.671227

C 40.159569 5.228596 29.109306

O 41.364777 5.711657 29.691620

C 41.949043 4.468313 30.004044

H 40.218239 3.605355 27.613186

H 39.857410 3.071935 29.252562

H 39.350883 5.298512 29.856878

H 39.878922 5.911628 28.293774

H 41.484074 4.023878 30.911493

H 43.014908 4.613036 30.232374

O 28.499613 0.747082 40.391808

C 29.757135 1.042883 39.802162

C 30.349226 2.160084 40.616714

O 29.417818 2.433884 41.654709

C 28.807638 1.166093 41.699692

H 30.394207 0.140811 39.820251

H 29.587967 1.271213 38.741188

H 30.526220 3.092469 40.064716

H 31.318769 1.881884 41.066040

H 27.881117 1.229165 42.288361

H 29.463779 0.431723 42.213066

O 8.742033 38.519215 14.411641

C 10.020091 39.117661 14.243450

C 10.407810 38.888355 12.806490

O 9.289143 38.267906 12.189464

C 8.784570 37.619587 13.332089

H 9.935506 40.173748 14.538274

H 10.727570 38.659538 14.956307

H 11.286414 38.223412 12.709683

H 10.647874 39.797272 12.237867

H 9.399641 36.730228 13.586820

H 7.766621 37.258755 13.126249

O 27.836136 3.801904 16.267612

C 29.150866 3.351601 15.982567

C 29.548851 4.017521 14.697664

O 28.551439 4.994888 14.431198

C 27.961792 5.082407 15.704368

H 29.823139 3.631757 16.814234

H 29.134729 2.255425 15.956165

H 29.601168 3.344757 13.828350

H 30.529642 4.515250 14.757803

H 26.966822 5.536131 15.603159

H 28.555840 5.747838 16.367353

O 26.412868 34.374084 47.910053

C 25.287846 34.040634 48.714828

C 25.401766 32.570427 0.128588

O 26.534122 32.105457 48.303642

C 27.256269 33.311153 48.282131

H 25.302023 34.663040 0.733391

H 24.380884 34.327148 48.160873

H 24.535385 31.961712 48.723141

H 25.557243 32.376324 1.205206

H 28.076689 33.228035 47.554604

H 27.719774 33.505745 0.379894

O 28.282042 5.279444 26.067684

C 29.240339 6.184450 25.551376

C 29.401686 7.242490 26.605017

O 28.725008 6.782409 27.774042

C 28.591530 5.422212 27.432283

H 30.185438 5.645651 25.350428

H 28.881954 6.545280 24.576942

H 28.974852 8.220346 26.335619

H 30.459726 7.423394 26.847034

H 27.785288 4.976798 28.031218

H 29.513651 4.866300 27.687990

O 42.776310 8.611978 12.726307

C 42.822269 7.185775 12.628521

C 44.202999 6.830813 12.179196

O 44.639614 7.970504 11.459495

C 44.101307 8.920491 12.348365

H 42.536736 6.746228 13.595621

H 42.062477 6.874327 11.893662

H 44.269985 5.945853 11.531856

H 44.878212 6.641109 13.034821

H 44.114506 9.909103 11.871660

H 44.739845 8.992364 13.251416

O 4.401330 7.162796 35.049294

C 5.451547 8.069267 35.388607

C 6.579993 7.239556 35.888783

O 6.450427 6.021637 35.176414

C 5.045248 5.918962 35.235573

H 5.733170 8.622245 34.482624

H 5.065783 8.806082 36.102444

H 6.495409 7.056209 36.974689

H 7.580339 7.658568 35.706413

H 4.737224 5.493595 36.210987

H 4.703487 5.221262 34.458668

O 21.281569 0.385274 16.248543

C 21.904463 0.145211 14.994443

C 23.387871 0.121745 15.258465

O 23.542372 0.290421 16.661688

C 22.281427 48.699184 17.051363

H 21.537277 48.076286 14.596946

H 21.569059 0.914296 14.284031

H 23.955517 0.916254 14.753403

H 23.862131 48.063576 14.955818

H 22.098568 0.069429 18.102558

H 22.251114 47.590294 16.988293

O 17.344231 17.675236 25.790464

C 16.396688 17.518290 26.841169

C 15.578223 16.299393 26.502832

O 16.169827 15.748371 25.338203

C 16.644087 16.957979 24.799894

H 16.947712 17.456684 27.790668

H 15.779661 18.432096 26.901796

H 14.525561 16.555590 26.281347

H 15.546444 15.520531 27.278759

H 15.805575 17.560339 24.385773

H 17.315384 16.748716 23.956982

O 29.283365 20.815620 34.365772

C 29.535164 21.952377 33.548286

C 28.849686 23.118471 34.206871

O 28.098202 22.560606 35.271267

C 28.966051 21.492786 35.553867

H 30.623514 22.103945 33.466145

H 29.181665 21.709869 32.537666

H 28.167631 23.698830 33.570774

H 29.576233 23.845997 34.614635

H 28.468323 20.797531 36.242275

H 29.888168 21.848724 36.062351

O 2.761954 10.467949 32.124523

C 3.813149 11.128001 31.435625

C 3.576020 12.604074 31.624842

O 2.336098 12.723862 32.311787

C 1.793388 11.474162 31.959757

H 4.769493 10.753483 31.826769

H 3.780880 10.834155 30.370251

H 3.512459 13.148252 30.667030

H 4.343147 13.124784 32.216934

H 1.413002 11.480518 30.916384

H 0.930919 11.264901 32.606606

O 22.042830 17.527092 13.229903

C 20.772594 17.449841 12.611898

C 20.078806 16.360998 13.355557

O 21.099688 15.476527 13.822483

C 22.249157 16.139513 13.337467

H 20.283178 18.431608 12.657368

H 20.892382 17.200975 11.540656

H 19.363016 15.790418 12.753198

H 19.533161 16.744316 14.231716

H 22.544470 15.721969 12.353744

H 23.086203 15.951275 14.021967

O 47.070076 39.342567 9.869989

C 46.329838 38.436588 10.677698

C 46.205162 37.170261 9.873900

O 46.897484 37.396633 8.654025

C 47.779022 38.385735 9.123885

H 46.867661 38.269375 11.628175

H 45.380833 38.918179 10.954432

H 45.175968 36.876419 9.626992

H 46.662308 36.305347 10.386297

H 48.258659 38.882000 8.269239

H 48.592110 37.928589 9.728689

O 15.933185 18.201323 20.849846

C 15.691166 19.211447 19.876879

C 16.905663 19.260830 18.992407

O 17.717773 18.167587 19.387951

C 17.333475 18.132872 20.739347

H 14.760246 18.953295 19.352747

H 15.497062 20.167791 20.390741

H 17.478197 20.198103 19.115129

H 16.698847 19.165977 17.917255

H 17.815557 18.959160 21.302103

H 17.681103 17.192175 21.185249

O 12.896455 6.447982 13.746211

C 12.196309 6.898285 12.594786

C 13.219635 6.985803 11.496653

O 14.455645 6.562881 12.062343

C 14.162289 6.941800 13.385382

H 11.354375 6.216720 12.408015

H 11.742095 7.882008 12.801601

H 13.334044 8.021353 11.130935

H 13.000595 6.375132 10.610227

H 14.205803 8.047755 13.505169

H 14.923549 6.529145 14.059612

O 45.989056 31.819923 25.653074

C 45.221439 32.812935 24.989599

C 43.990318 33.010464 25.832510

O 44.248470 32.373390 27.079277

C 45.638496 32.192974 26.963400

H 45.816952 33.742386 24.909903

H 45.040047 32.478508 23.958450

H 43.076023 32.559669 25.420343

H 43.744389 34.070457 26.007547

H 45.937717 31.401400 27.660122

H 46.187073 33.108734 27.267027

O 38.596954 20.169258 21.746538

C 39.375328 18.977741 21.647776

C 40.603516 19.324879 20.858646

O 40.684677 20.739347 20.900206

C 39.302475 21.003368 20.856201

H 39.596321 18.637445 22.671103

H 38.755856 18.192032 21.191116

H 40.536530 18.988985 19.807449

H 41.542744 18.912712 21.252234

H 38.916714 20.871847 19.823586

H 39.124996 22.051630 21.135380

O 29.083393 17.594563 38.020508

C 30.191303 17.490911 38.906445

C 29.809452 18.290306 40.126320

O 28.544594 18.868708 39.832966

C 28.660959 18.871643 38.433651

H 30.402519 16.425047 39.080017

H 31.089464 17.897697 38.405293

H 30.527685 19.099483 40.346825

H 29.706778 17.711906 41.054794

H 29.363550 19.658817 38.081623

H 27.679193 19.097038 37.991661

O 17.520735 26.370331 17.745153

C 18.778746 26.689114 18.322577

C 19.678862 27.052387 17.175062

O 18.866264 27.017183 16.009459

C 17.625364 27.228888 16.633331

H 18.655048 27.528112 19.030544

H 19.111706 25.831533 18.922491

H 20.523241 26.366909 17.022028

H 20.117920 28.059088 17.283606

H 16.817656 27.012293 15.919007

H 17.507534 28.293774 16.934509

O 27.389746 9.504760 10.154546

C 26.534611 8.377781 10.283134

C 25.332827 8.690696 9.444622

O 25.716635 9.737490 8.556240

C 27.106169 9.766337 8.796304

H 27.062654 7.468864 9.935506

H 26.309706 8.217902 11.346063

H 24.475248 9.048590 10.033780

H 24.964176 7.837027 8.859375

H 27.498777 10.754950 8.519081

H 27.624432 9.035389 8.144073

O 15.557689 25.784107 23.089624

C 16.217253 26.526300 24.102194

C 17.227377 25.582180 24.669350

O 16.813255 24.269897 24.299723

C 15.531287 24.570097 23.806881

H 16.641642 27.441572 23.671938

H 15.485328 26.855347 24.864922

H 17.315384 25.640850 25.765039

H 18.227724 25.743038 24.246918

H 14.787138 24.627304 24.630726

H 15.210060 23.744299 23.159540

O 22.825603 35.426743 23.625002

C 21.496208 35.167614 24.054768

C 20.595602 36.044258 23.226524

O 21.433626 36.690624 22.282404

C 22.622698 36.689156 23.034864

H 21.313349 34.088551 23.946226

H 21.428736 35.382744 25.132854

H 20.081249 36.813835 23.827906

H 19.805494 35.512794 22.676481

H 22.598742 37.484642 23.809326

H 23.467077 36.911129 22.367479

O 14.479113 16.534567 3.388760

C 15.818776 16.424557 2.952636

C 16.482252 15.531287 3.966673

O 15.526397 15.325448 5.000755

C 14.750957 16.473452 4.763625

H 15.806553 16.056395 1.918553

H 16.278858 17.431261 2.912544

H 17.392635 15.983056 4.395952

H 16.788809 14.544141 3.592643

H 15.256999 17.395081 5.125921

H 13.804882 16.406958 5.312202

O 47.563892 19.042767 41.766674

C 46.514652 19.677885 41.058216

C 45.891758 20.611738 42.048786

O 46.874508 20.850334 43.057442

C 47.974102 20.193705 42.463394

H 46.928284 20.215216 40.182056

H 45.837978 18.915157 40.658272

H 44.999466 20.194193 42.539669

H 45.573956 21.566614 41.610706

H 48.694782 19.913059 43.244701

H 48.501656 20.887981 41.779385

O 6.615685 28.623800 31.117332

C 6.926643 29.112240 29.825584

C 6.448471 28.049799 28.881954

O 6.288592 26.857306 29.650061

C 6.902196 27.272404 30.850380

H 6.449938 30.091074 29.689175

H 8.016952 29.288254 29.752245

H 7.140794 27.854717 28.050776

H 5.475994 28.285952 28.423830

H 8.000818 27.107145 30.801973

H 6.522789 26.658310 31.678623

O 37.773602 7.918189 5.078984

C 37.061718 7.205332 4.075215

C 37.950592 6.061729 3.659137

O 39.179756 6.255345 4.340703

C 38.646336 6.888996 5.477460

H 36.093155 6.873838 4.484936

H 36.813835 7.914766 3.273862

H 38.158386 6.000124 2.582029

H 37.534512 5.077517 3.946138

H 39.463333 7.324142 6.069552

H 38.131004 6.153648 6.132624

O 44.467510 36.829967 27.620522

C 45.025867 35.571468 27.959837

C 46.082928 35.839890 28.996365

O 46.104443 37.248001 29.173355

C 44.759892 37.490997 28.825727

H 44.225002 34.907993 28.332888

H 45.399899 35.120678 27.032829

H 47.097458 35.513287 28.726963

H 45.852158 35.350960 29.960041

H 44.596588 38.568596 28.692739

H 44.072948 37.166840 29.636858

O 43.840218 33.130249 10.906516

C 42.722042 33.242702 10.039647

C 43.196789 34.035255 8.849596

O 44.541832 34.402927 9.118996

C 44.490005 34.315899 10.521730

H 42.358765 32.228664 9.815719

H 41.900639 33.752167 10.576980

H 42.594917 34.946129 8.687761

H 43.190922 33.493034 7.894231

H 43.979073 35.204773 10.951009

H 45.515285 34.309544 10.918740

O 29.404617 47.860672 35.436523

C 30.086184 46.884285 36.210987

C 31.279657 47.575138 36.812366

O 31.226366 0.027872 36.362064

C 30.503727 48.695271 35.175926

H 29.376261 46.480919 36.945354

H 30.371719 46.040882 35.557289

H 32.235023 47.127281 36.487720

H 31.303616 47.580029 37.911964

H 31.153027 48.248882 34.393642

H 30.144367 0.764195 34.783806

O 23.484678 24.381372 3.336445

C 23.611799 23.059799 2.823559

C 22.328854 22.758131 2.093590

O 21.485453 23.880222 2.304806

C 22.494110 24.848299 2.451485

H 24.493826 23.017263 2.161062

H 23.830839 22.389969 3.668427

H 21.793964 21.859972 2.435350

H 22.486776 22.623676 1.007192

H 22.055054 25.771397 2.856317

H 22.938057 25.104010 1.464339

O 0.082630 24.220516 31.424870

C 0.018092 23.820084 32.787510

C 47.545315 24.225893 33.279373

O 46.910198 24.853676 32.177326

C 48.067978 25.292736 31.512877

H 0.231262 22.743954 32.832981

H 0.832644 24.312922 33.348312

H 47.585403 24.937773 34.122776

H 46.899441 23.403027 33.615753

H 48.522194 26.165960 32.029671

H 47.800045 25.632050 30.502262

O 7.032740 31.858059 27.992105

C 6.683157 31.191650 26.787876

C 6.727160 32.232578 25.700989

O 7.034207 33.463211 26.338551

C 7.742175 32.936634 27.433750

H 7.395036 30.365362 26.615284

H 5.700901 30.720324 26.938467

H 5.786952 32.358723 25.144100

H 7.507978 32.025272 24.948040

H 7.871252 33.715008 28.196966

H 8.759145 32.620296 27.123281

O 6.974069 44.461647 1.089332

C 5.827044 44.405418 0.255709

C 4.661928 44.828831 1.109867

O 5.199749 45.120232 2.390858

C 6.315972 44.264606 2.315563

H 6.021148 45.033691 48.268440

H 5.706768 43.377201 48.766167

H 3.897734 44.035789 1.206185

H 4.125085 45.717213 0.755883

H 6.014303 43.203144 2.436328

H 7.007316 44.482670 3.138918

O 35.028267 43.719452 37.261200

C 34.571121 43.560062 38.594997

C 35.782684 43.740475 39.472134

O 36.878372 43.957069 38.594021

C 36.347397 43.282841 37.479752

H 33.754124 44.274384 38.768078

H 34.122284 42.556782 38.709404

H 35.984612 42.849159 40.092094

H 35.726944 44.586323 40.170811

H 36.391888 42.181286 37.621056

H 36.952690 43.511169 36.591858

O 24.691843 32.467751 41.523678

C 23.753099 33.054466 40.639206

C 23.049044 34.123264 41.422958

O 23.691496 34.183887 42.688301

C 24.918705 33.595222 42.335297

H 24.292875 33.472500 39.774292

H 23.103804 32.258492 40.247574

H 21.983181 33.925735 41.599949

H 23.109671 35.112850 40.937943

H 25.444792 33.285728 43.249592

H 25.571913 34.332035 41.821434

O 15.899938 8.442809 10.537375

C 15.715124 9.813763 10.193171

C 15.885270 9.899815 8.700962

O 16.350241 8.623222 8.295641

C 15.658897 7.886409 9.267141

H 14.714288 10.142323 10.525641

H 16.438736 10.410744 10.768150

H 16.600573 10.654719 8.351868

H 14.931372 10.120320 8.183677

H 16.003592 6.845481 9.251985

H 14.572498 7.850717 9.043212

O 4.479558 38.919643 21.942600

C 5.289712 39.143085 20.798508

C 5.152323 40.607914 20.475327

O 4.198914 41.133022 21.390110

C 3.534950 39.928791 21.686890

H 6.310594 38.807194 21.030748

H 4.935728 38.495747 19.974174

H 4.793450 40.783928 19.446623

H 6.078842 41.190716 20.578979

H 2.854362 39.634457 20.856691

H 2.908633 40.073029 22.578695

O 46.594349 29.876923 7.713328

C 47.164436 31.150581 7.418993

C 46.286324 32.124035 8.103004

O 46.069729 31.507500 9.361993

C 45.945541 30.164902 8.937115

H 48.175541 31.203384 7.859518

H 47.252934 31.259121 6.330640

H 45.328514 32.261425 7.569583

H 46.724892 33.119007 8.260439

H 44.877232 29.912127 8.829551

H 46.359661 29.513651 9.718422

O 27.633232 43.475964 31.192139

C 28.149054 43.738522 32.487801

C 29.624636 43.957561 32.303474

O 29.932173 43.555660 30.970165

C 28.765589 42.814449 30.682676

H 27.603897 44.591698 32.916100

H 27.938814 42.879963 33.154697

H 30.235306 43.375248 33.013885

H 29.940973 45.005333 32.413479

H 28.831596 41.778896 31.080175

H 28.673182 42.722042 29.591387

O 40.547775 1.186628 6.231388

C 39.165089 0.958788 6.463140

C 38.542194 2.316052 6.666045

O 39.601704 3.257238 6.554080

C 40.648495 2.398192 6.937399

H 38.766613 0.394565 5.610449

H 39.049213 0.303135 7.346143

H 38.075756 2.417260 7.661991

H 37.762356 2.583007 5.937053

H 40.638229 2.220711 8.036510

H 41.611195 2.872941 6.700758

O 6.746228 28.842350 22.301962

C 7.634122 28.091356 23.112116

C 7.012694 28.078644 24.481604

O 5.956610 29.036945 24.461557

C 6.308149 29.738068 23.291063

H 7.773466 27.107635 22.643234

H 8.629090 28.574417 23.110649

H 7.722618 28.345600 25.281488

H 6.574615 27.112036 24.768114

H 7.096790 30.490040 23.501303

H 5.428567 30.287622 22.923878

O 0.835578 20.573601 39.678463

C 0.751482 19.674461 38.586689

C 0.994968 18.330889 39.192955

O 1.736183 18.548950 40.393276

C 1.896063 19.948261 40.357582

H 48.660069 19.789360 38.110958

H 1.505409 19.938972 37.821514

H 1.546968 17.628298 38.552948

H 0.056227 17.835115 39.477024

H 2.860718 20.217173 39.883812

H 1.937133 20.319847 41.390198

O 32.288803 14.089438 40.346340

C 33.077934 12.906721 40.400120

C 34.037701 13.074424 41.538834

O 33.925247 14.429243 41.944645

C 32.562603 14.593034 41.628796

H 32.415436 12.037896 40.535065

H 33.560997 12.786935 39.421772

H 35.089382 12.866140 41.293880

H 33.777103 12.413881 42.386147

H 32.310806 15.661830 41.639065

H 31.921621 14.095795 42.389080

O 31.604794 3.051888 0.695256

C 30.598091 3.274351 1.682890

C 29.802607 2.022206 1.754763

O 30.760416 1.011592 1.498075

C 31.437580 1.669200 0.452747

H 31.083107 3.471389 2.649501

H 30.048536 4.181312 1.413980

H 29.005165 1.996782 0.989590

H 29.328346 1.831035 2.728707

H 30.887535 1.529367 48.393604

H 32.424725 1.209119 0.318292

O 4.805184 2.845072 40.789795

C 4.756292 2.030029 39.630547

C 4.856033 0.609693 40.119473

O 5.020312 0.695256 41.530033

C 4.305500 1.898507 41.697735

H 3.806793 2.224622 39.108860

H 5.549821 2.358588 38.945557

H 5.687211 0.024446 39.705841

H 3.942226 0.030314 39.888210

H 4.432132 2.267159 42.725460

H 3.212746 1.750362 41.551544

O 19.954130 5.897449 38.729942

C 20.087118 5.189482 39.952751

C 18.725941 5.228107 40.597160

O 17.840982 5.816288 39.652550

C 18.598331 5.614360 38.482544

H 20.429367 4.160288 39.739090

H 20.893360 5.661786 40.535065

H 18.675583 5.819221 41.523678

H 18.336756 4.232161 40.858246

H 18.228703 6.288103 37.696350

H 18.475611 4.577833 38.104115

O 31.143736 14.691309 17.535892

C 31.611151 16.021193 17.348145

C 32.181240 16.462694 18.671181

O 32.119148 15.328382 19.523384

C 30.999012 14.712332 18.935692

H 32.332809 16.008970 16.517942

H 30.770193 16.651421 17.010294

H 31.600883 17.287516 19.124908

H 33.224613 16.807877 18.636469

H 30.060760 15.233529 19.227583

H 30.919319 13.679228 19.304832

O 24.832653 40.096985 12.087277

C 24.937283 40.562443 13.423030

C 23.853819 39.854477 14.193090

O 23.179588 39.025742 13.255816

C 24.250828 38.848755 12.363522

H 25.948875 40.334114 13.803905

H 24.854656 41.658131 13.398094

H 23.111626 40.514530 14.660505

H 24.241539 39.227673 15.013512

H 23.883154 38.415073 11.423802

H 24.995466 38.133942 12.776178

O 36.393845 11.259522 31.267433

C 36.663242 9.978043 30.726679

C 35.357803 9.519428 30.154144

O 34.579922 10.691388 29.901859

C 35.515732 11.687335 30.255354

H 37.446018 10.062138 29.948795

H 37.076878 9.340969 31.521677

H 34.790161 8.877954 30.845490

H 35.452168 8.956672 29.216869

H 34.973507 12.580117 30.596136

H 36.097553 11.983625 29.362082

O 7.717728 6.167827 7.472775

C 6.678267 5.199749 7.501622

C 6.898774 4.342659 6.286148

O 8.165097 4.719622 5.761039

C 8.700474 5.343005 6.900241

H 6.742317 4.622814 8.443297

H 5.710679 5.720946 7.534380

H 6.151203 4.477114 5.490662

H 6.914909 3.264083 6.508121

H 9.564409 5.952698 6.598083

H 9.071570 4.596901 7.633633

O 8.981118 3.954450 29.254517

C 8.442320 5.160146 28.718163

C 8.599754 5.071161 27.233778

O 9.544852 4.038545 27.012293

C 9.127308 3.207368 28.069357

H 7.392102 5.264777 29.025700

H 8.979163 6.002569 29.176779

H 8.954716 5.991813 26.750717

H 7.644878 4.801273 26.744360

H 9.875367 2.418727 28.233635

H 8.174876 2.695460 27.815603

O 15.276555 10.332026 25.432079

C 15.799708 11.301082 26.338551

C 14.628238 11.897573 27.060209

O 13.544772 11.011147 26.830412

C 13.902668 10.633206 25.523018

H 16.506699 10.784285 27.003492

H 16.390333 12.042296 25.779217

H 14.371060 12.901833 26.673466

H 14.763669 12.011983 28.143673

H 13.642070 11.432604 24.800383

H 13.323776 9.744824 25.235529

O 11.882417 19.120996 23.938892

C 12.793778 18.362669 23.151718

C 12.802580 18.989964 21.787119

O 11.697114 19.878345 21.764629

C 11.776809 20.260199 23.117495

H 13.783857 18.376358 23.635267

H 12.453485 17.317829 23.163940

H 12.717017 18.287863 20.945675

H 13.726654 19.569832 21.616974

H 10.868869 20.812687 23.393738

H 12.636832 20.945187 23.286173

O 35.871181 28.847240 13.390270

C 36.694534 29.962486 13.715898

C 36.723873 30.034847 15.218862

O 36.121510 28.829638 15.666720

C 35.192547 28.727943 14.616502

H 37.673859 29.801140 13.242615

H 36.279434 30.871403 13.247503

H 36.143513 30.896826 15.601692

H 37.718838 30.116987 15.675520

H 34.397060 29.498003 14.715755

H 34.689930 27.751553 14.660505

O 20.103252 42.824715 40.825001

C 20.793617 43.223679 39.650105

C 21.084042 44.692909 39.807541

O 20.531553 45.074272 41.060661

C 20.599026 43.815281 41.686489

H 21.711336 42.617409 39.552807

H 20.163879 42.970905 38.785679

H 20.646940 45.336338 39.030144

H 22.167995 44.907547 39.806561

H 19.981020 43.828484 42.591496

H 21.638487 43.582554 42.007229

O 40.277401 46.554256 40.136101

C 39.218868 45.604759 40.090630

C 38.160831 46.102486 41.035725

O 38.683495 47.283737 41.628307

C 39.529827 47.661678 40.573200

H 38.849728 45.522621 39.054592

H 39.629082 44.614681 40.335094

H 37.890942 45.396965 41.834145

H 37.217201 46.349884 40.516975

H 40.216282 48.438587 40.929630

H 38.951427 48.111980 39.739090

O 3.523215 25.527418 37.982372

C 3.199056 24.191179 38.323154

C 2.893965 23.508146 37.024075

O 2.675903 24.534407 36.058929

C 2.631410 25.664808 36.902817

H 4.035612 23.759945 38.889336

H 2.341965 24.195091 39.018410

H 2.016828 22.848583 37.065144

H 3.710964 22.879875 36.648087

H 1.600261 25.841801 37.268047

H 2.914500 26.552214 36.321484

O 30.876780 36.763962 48.051353

C 31.766140 35.668278 48.230301

C 30.943274 34.548141 48.810658

O 29.661797 35.095737 0.182373

C 29.662283 36.053059 48.045979

H 32.224266 35.417942 47.259289

H 32.591450 36.008568 48.873241

H 31.339796 34.115440 0.847798

H 30.816154 33.702297 48.115894

H 28.832573 36.756142 48.202435

H 29.490669 35.568535 47.060299

O 16.446070 42.116257 4.516228

C 17.380411 41.400955 5.314158

C 18.414495 42.402283 5.753705

O 18.013086 43.656868 5.221751

C 17.288982 43.170876 4.121174

H 16.829390 40.913494 6.131646

H 17.818981 40.581512 4.718644

H 19.421198 42.154884 5.373319

H 18.514725 42.516197 6.841569

H 17.982283 42.845249 3.321777

H 16.677334 43.982986 3.704607

O 23.923248 32.358723 13.166342

C 23.551662 33.731140 13.181009

C 23.091091 34.022057 14.587167

O 23.212835 32.800713 15.302468

C 24.246428 32.239910 14.530452

H 24.421465 34.347679 12.888631

H 22.796268 33.888088 12.397747

H 22.050653 34.363815 14.676640

H 23.710073 34.794071 15.081472

H 24.354969 31.176495 14.788116

H 25.223307 32.723953 14.755358

O 7.659546 13.372670 21.512344

C 7.315829 13.965250 20.268511

C 5.884737 13.588287 20.007421

O 5.443724 12.903788 21.170582

C 6.377088 13.418629 22.086346

H 7.438551 15.058982 20.355049

H 8.039932 13.631801 19.511650

H 5.724858 12.923344 19.146421

H 5.240330 14.468358 19.840698

H 6.360464 12.809425 23.000639

H 6.109155 14.455645 22.389479

O 21.130489 1.297615 29.652016

C 19.798161 1.718582 29.911636

C 19.702332 3.138429 29.421732

O 20.953987 3.430319 28.816927

C 21.739695 2.563938 29.599213

H 19.121485 1.006703 29.419287

H 19.595745 1.632042 30.992655

H 19.525339 3.851286 30.248508

H 18.914669 3.326666 28.679050

H 21.890774 2.979038 30.619118

H 22.736130 2.466153 29.145487

O 7.453707 23.837685 37.592205

C 6.846458 25.116720 37.513000

C 6.146802 25.133833 36.185562

O 5.983012 23.773146 35.785130

C 6.417180 23.129717 36.961491

H 6.142891 25.242374 38.356892

H 7.619943 25.887758 37.642567

H 6.712492 25.651119 35.395943

H 5.164057 25.619337 36.209518

H 6.757963 22.123991 36.695023

H 5.578669 22.988417 37.669456

O 19.489649 40.803486 8.317643

C 18.674116 39.712688 8.717586

C 18.754789 38.683495 7.621899

O 19.537563 39.274117 6.597594

C 20.317892 40.099918 7.427306

H 19.041302 39.332302 9.686642

H 17.664480 40.107250 8.890178

H 17.789156 38.385250 7.189197

H 19.238338 37.748173 7.959748

H 20.857180 40.825977 6.806856

H 21.080620 39.508316 7.979794

O 9.091127 25.139210 35.674141

C 10.452303 25.529863 35.702011

C 11.006258 25.167568 34.350124

O 9.945284 24.552498 33.630913

C 9.206514 24.101217 34.736378

H 10.496306 26.599640 35.955276

H 10.961765 24.997421 36.525364

H 11.844280 24.453735 34.422977

H 11.373932 26.017815 33.758034

H 9.673441 23.203056 35.196457

H 8.204211 23.802971 34.398041

O 13.910979 30.995100 47.791733

C 15.184637 30.361938 47.798580

C 15.645696 30.288113 46.369930

O 14.534852 30.675831 45.576889

C 13.938848 31.551500 46.501942

H 15.867181 30.937897 48.445431

H 15.058982 29.380173 48.277729

H 15.980124 29.293142 46.042839

H 16.489586 30.970165 46.169472

H 12.905744 31.748539 46.185608

H 14.471779 32.522999 46.523453

O 37.364857 14.918660 26.874418

C 36.048172 15.396343 27.098345

C 35.426743 15.539110 25.738636

O 36.463272 15.287801 24.795494

C 37.569229 15.537642 25.629606

H 35.535774 14.703531 27.780888

H 36.103912 16.361486 27.630787

H 35.012623 16.545813 25.562134

H 34.605347 14.836519 25.541599

H 37.738396 16.630398 25.751839

H 38.475700 15.125476 25.163658

O 11.789032 20.865980 16.554613

C 12.042296 19.842653 15.602181

C 13.363379 19.234428 15.980124

O 13.811727 19.930172 17.134970

C 12.554205 20.343315 17.612654

H 11.215030 19.110729 15.629560

H 12.020294 20.289045 14.598901

H 14.144198 19.311678 15.210060

H 13.267061 18.161718 16.216274

H 12.693549 21.121691 18.376358

H 12.024206 19.499914 18.105982

O 10.709479 34.752510 44.007919

C 12.036429 34.562809 43.549305

C 12.841205 34.321766 44.789227

O 11.912242 33.988316 45.817444

C 10.774017 33.773678 45.018047

H 12.066743 33.696915 42.861385

H 12.347387 35.427235 42.950367

H 13.410317 35.199394 45.128544

H 13.566285 33.503300 44.672375

H 9.875856 33.830395 45.644363

H 10.784774 32.748886 44.582897

O 37.470463 26.520920 11.883394

C 36.953178 27.276314 10.784774

C 35.580757 26.739471 10.490439

O 35.542622 25.466305 11.111867

C 36.292637 25.831045 12.243245

H 36.958069 28.346581 11.048306

H 37.658211 27.153107 9.950173

H 35.333359 26.607462 9.428487

H 34.793091 27.397079 10.911406

H 36.561058 24.925550 12.803557

H 35.685387 26.458826 12.923834

O 40.475903 15.650096 45.155922

C 40.199169 16.560480 44.105705

C 40.762905 17.870317 44.573120

O 41.715336 17.566694 45.595470

C 41.744671 16.162004 45.479595

H 40.679298 16.206985 43.172344

H 39.117172 16.559504 43.914047

H 40.001644 18.529882 45.013157

H 41.254280 18.455076 43.780567

H 42.066387 15.731747 46.435448

H 42.493710 15.854468 44.719311

O 33.387913 28.512323 43.789368

C 33.784435 27.405882 44.599033

C 34.547161 27.982817 45.734325

O 33.849953 29.189491 45.991501

C 33.708652 29.588455 44.644016

H 32.876984 26.922819 44.984798

H 34.304653 26.671021 43.972229

H 35.603249 28.190121 45.476170

H 34.550583 27.364811 46.641773

H 34.644459 30.067606 44.296387

H 32.919521 30.346781 44.562855

O 3.189766 6.815167 10.839045

C 4.198914 6.640131 9.849455

C 5.484305 7.149105 10.438612

O 5.230552 7.331964 11.821301

C 3.870354 7.675191 11.721560

H 4.221893 5.571334 9.588856

H 3.899690 7.178930 8.936137

H 5.793797 8.113760 9.994177

H 6.343841 6.470962 10.333982

H 3.766701 8.728342 11.389578

H 3.407339 7.602341 12.716040

O 3.934893 41.557411 2.262270

C 4.597390 40.837711 3.296352

C 5.840245 40.256378 2.677370

O 5.899405 40.777573 1.358242

C 4.513294 40.902252 1.159248

H 4.781716 41.531498 4.129486

H 3.916313 40.061291 3.688473

H 5.794286 39.153355 2.626032

H 6.782898 40.501816 3.189277

H 4.053213 39.905323 0.999369

H 4.322612 41.487007 0.247886

O 2.974638 45.590092 13.541839

C 4.296699 45.755348 13.065623

C 4.156866 46.625637 11.854059

O 2.793735 46.555233 11.437493

C 2.344898 45.523598 12.286271

H 4.912260 46.177780 13.873821

H 4.732334 44.765759 12.826048

H 4.794917 46.318104 11.014570

H 4.391551 47.680260 12.059409

H 2.527758 44.534496 11.814945

H 1.257523 45.616493 12.419260

O 10.440569 19.236872 26.472517

C 10.394120 20.622982 26.766853

C 10.681610 20.741302 28.238035

O 10.663031 19.418753 28.763634

C 9.969730 18.784124 27.715860

H 11.103555 21.144669 26.107777

H 9.389862 21.008747 26.513098

H 9.933062 21.349041 28.774389

H 11.660933 21.184761 28.472233

H 8.875510 18.956228 27.809736

H 10.131077 17.700661 27.785778

O 42.175415 39.616859 35.186680

C 41.380913 38.492809 34.832699

C 40.912029 37.882141 36.132267

O 41.427849 38.703049 37.169285

C 42.548958 39.172424 36.466694

H 40.588848 38.840927 34.155045

H 41.995003 37.791199 34.239628

H 41.289478 36.852947 36.269657

H 39.825630 37.822983 36.269169

H 43.327820 38.383781 36.395313

H 42.989483 40.007023 37.022118

O 40.995636 44.580944 45.147610

C 41.507053 43.422672 45.798862

C 40.800064 42.239956 45.199436

O 40.108719 42.746975 44.070503

C 39.825630 44.015743 44.603924

H 42.594917 43.417782 45.660007

H 41.341797 43.514103 46.883305

H 40.074493 41.790142 45.903984

H 41.451317 41.418556 44.869411

H 39.021832 43.953159 45.370560

H 39.453068 44.666504 43.801105

O 34.870342 18.874086 19.156687

C 36.182137 19.144464 18.674604

C 36.159161 20.545732 18.124561

O 34.805317 20.960833 18.193989

C 34.435688 20.202505 19.318523

H 36.899887 19.026144 19.504316

H 36.430027 18.369024 17.936323

H 36.498966 20.649385 17.083633

H 36.780586 21.240499 18.718121

H 33.341469 20.214727 19.426086

H 34.853722 20.646452 20.246508

O 36.087776 44.466534 18.585131

C 36.206585 43.406052 17.644922

C 35.579781 42.198887 18.289330

O 34.946617 42.663368 19.473513

C 35.872158 43.695004 19.741446

H 35.725479 43.729229 16.709114

H 37.271957 43.253994 17.403881

H 36.330772 41.442024 18.569975

H 34.842476 41.670841 17.668390

H 36.831924 43.278927 20.119875

H 35.481506 44.349190 20.532532

O 8.272173 28.619398 19.400663

C 8.885777 29.704332 20.077339

C 9.803006 30.365852 19.083838

O 9.817185 29.536629 17.930456

C 9.309678 28.350491 18.491257

H 8.087358 30.346781 20.472881

H 9.431910 29.319056 20.955942

H 10.835133 30.478794 19.454933

H 9.482269 31.368641 18.765057

H 10.118854 27.772577 18.978718

H 8.917069 27.726616 17.678659

O 39.977200 32.428638 3.590198

C 40.072048 32.082478 2.211910

C 39.367996 33.158607 1.449183

O 39.256516 34.240604 2.355655

C 39.020367 33.461254 3.502191

H 41.138401 32.003757 1.962557

H 39.634457 31.083597 2.055453

H 38.361290 32.826138 1.134313

H 39.882835 33.505745 0.543199

H 37.990196 33.042240 3.491435

H 39.093216 34.096859 4.395463

O 39.371414 9.973153 36.030571

C 39.714642 9.653395 34.689442

C 41.219563 9.691042 34.630768

O 41.659599 10.160901 35.902470

C 40.471504 10.801886 36.301437

H 39.260918 8.684339 34.435688

H 39.248695 10.393142 34.012276

H 41.594570 10.369674 33.845062

H 41.698711 8.718564 34.444000

H 40.359539 11.784631 35.792953

H 40.515999 11.004302 37.380501

O 26.397223 44.208382 11.873616

C 27.481667 44.943237 12.416327

C 26.857792 46.191471 12.961969

O 25.611025 46.360641 12.284805

C 25.717125 45.318737 11.338729

H 28.003349 44.323277 13.156563

H 28.225813 45.148098 11.622796

H 27.466995 47.096966 12.826537

H 26.638266 46.117645 14.037612

H 26.239786 45.682499 10.430301

H 24.706511 45.018532 11.026304

O 28.515749 44.291988 38.098248

C 27.290981 44.651352 37.478775

C 27.499266 44.485603 35.995857

O 28.832083 44.023567 35.833534

C 28.980719 43.421696 37.096924

H 26.488651 43.996185 37.861118

H 27.044073 45.670277 37.805382

H 27.383389 45.405277 35.403767

H 26.808899 43.745365 35.553867

H 30.043648 43.205589 37.274403

H 28.433119 42.456551 37.137505

O 10.839045 38.959740 46.696533

C 11.414023 38.572506 47.947701

C 12.159150 37.292496 47.711060

O 11.704936 36.827034 46.455006

C 11.561680 38.087978 45.855579

H 12.066743 39.382172 48.312931

H 10.597514 38.477654 48.678650

H 11.996337 36.505322 48.461567

H 13.251416 37.458729 47.664612

H 11.011636 37.969170 44.916836

H 12.555182 38.516281 45.597912

O 20.047026 43.536102 18.594421

C 19.670551 44.908527 18.601753

C 20.950563 45.647785 18.594421

O 21.721115 44.920750 19.535118

C 21.369087 43.625580 19.089216

H 19.003164 45.100674 17.755909

H 19.114639 45.117786 19.531206

H 20.886515 46.699467 18.904890

H 21.421892 45.615025 17.593586

H 21.494741 42.914188 19.916969

H 22.053585 43.310219 18.285418

O 8.597799 34.495335 17.446417

C 9.664150 34.534451 16.514032

C 10.305624 35.882915 16.693468

O 9.793227 36.427090 17.905521

C 9.213848 35.265396 18.449697

H 10.362829 33.703274 16.727203

H 9.258830 34.344746 15.509284

H 10.072894 36.591858 15.887226

H 11.403756 35.846245 16.758495

H 8.457477 35.561687 19.188957

H 9.981465 34.668419 18.986542

O 37.915386 25.954742 46.676491

C 37.525711 25.866245 48.039619

C 36.980068 24.476225 48.223946

O 37.000114 23.863110 46.941975

C 36.996693 25.026758 46.152847

H 38.399426 26.114134 48.660557

H 36.768852 26.643152 48.253773

H 35.945496 24.473289 48.610199

H 37.563362 23.839640 0.010754

H 35.975323 25.461414 46.094173

H 37.288582 24.765179 45.126099

O 28.359291 21.281569 18.477077

C 29.412441 20.317892 18.429653

C 28.815950 19.110729 17.814091

O 27.507090 19.087261 18.358757

C 27.228401 20.465548 18.252661

H 30.259264 20.751572 17.889387

H 29.745890 20.103741 19.458357

H 29.318569 18.164654 18.053667

H 28.786125 19.200201 16.712536

H 26.453938 20.731037 18.985075

H 26.818190 20.689476 17.250847

O 1.913175 19.281364 47.800533

C 2.523846 18.452143 46.823654

C 3.203456 17.345699 47.579540

O 2.952636 17.595541 0.063072

C 2.785912 18.984097 48.862484

H 1.749873 18.115273 46.121067

H 3.234748 19.058414 46.235474

H 4.292299 17.327608 47.403034

H 2.842627 16.337528 47.338497

H 3.774035 19.478401 48.733898

H 2.355655 19.370838 0.903050

O 35.382744 39.509293 14.205803

C 36.650043 39.654995 13.575086

C 36.536610 39.003254 12.223200

O 35.257088 38.391117 12.193376

C 34.605347 39.293674 13.052422

H 36.896461 40.727703 13.512992

H 37.406902 39.202736 14.231716

H 37.295425 38.235634 12.016384

H 36.604572 39.733223 11.396422

H 33.641178 38.866840 13.354091

H 34.388752 40.252953 12.532203

O 43.074554 31.653200 3.759367

C 43.773724 31.897173 4.973864

C 43.860264 30.568756 5.679876

O 43.224171 29.626591 4.827675

C 42.355343 30.519375 4.180335

H 44.742290 32.355297 4.725978

H 43.220257 32.654034 5.558133

H 43.347866 30.574623 6.659199

H 44.880169 30.209883 5.872025

H 41.513901 30.805399 4.847232

H 41.914330 30.022621 3.304664

O 11.769475 40.475903 23.586376

C 12.764443 40.129253 24.537828

C 12.027140 39.800205 25.808552

O 10.650807 40.019245 25.534754

C 10.708501 39.750332 24.156466

H 13.350180 39.276073 24.150599

H 13.469477 40.968746 24.618992

H 12.311207 40.407944 26.676401

H 12.171863 38.750965 26.119511

H 9.767315 40.057381 23.680250

H 10.821932 38.659046 23.969694

O 41.946598 33.634823 21.577858

C 43.136162 34.365284 21.308460

C 43.540504 35.011646 22.606075

O 42.601765 34.575031 23.580019

C 42.159775 33.399651 22.945391

H 43.897423 33.667091 20.919273

H 42.927879 35.070316 20.490484

H 43.535126 36.111732 22.594830

H 44.550629 34.723667 22.937567

H 41.217609 33.075001 23.408895

H 42.889252 32.574337 23.087181

O 0.320737 30.634272 20.511997

C 0.887404 31.895708 20.192238

C 2.115591 32.010605 21.049818

O 1.981136 31.044481 22.091234

C 0.642451 30.661163 21.881483

H 1.063418 31.943134 19.108774

H 0.155968 32.696083 20.410788

H 2.245646 33.004597 21.504030

H 3.049933 31.791075 20.511997

H 48.845863 31.341751 22.423704

H 0.501151 29.659351 22.306362

O 22.705328 22.014473 11.343129

C 22.323963 20.676764 11.623774

C 22.846138 20.376076 13.003530

O 23.457787 21.569059 13.471922

C 23.809814 22.079500 12.209510

H 21.231697 20.608315 11.522078

H 22.747864 20.008890 10.852245

H 23.597132 19.565432 13.001084

H 22.075588 20.079784 13.727632

H 24.675217 21.518209 11.800277

H 24.124685 23.128250 12.308762

O 23.569262 20.322292 40.697876

C 24.389194 21.189650 39.935150

C 23.617178 22.473576 39.846653

O 22.502422 22.345966 40.719391

C 23.002596 21.311394 41.525143

H 25.358250 21.329973 40.452435

H 24.627304 20.706589 38.976852

H 23.232391 22.734663 38.853642

H 24.225405 23.327244 40.160057

H 22.179241 20.875269 42.109413

H 23.746744 21.698624 42.257557

O 23.805414 10.173124 48.569618

C 24.533916 10.920207 0.640496

C 24.185801 10.326648 1.980158

O 23.225546 9.306255 1.734717

C 23.625490 8.994808 0.421456

H 25.611515 10.849801 0.409722

H 24.265007 11.980692 0.520219

H 23.752611 11.036083 2.697905

H 25.063425 9.881234 2.482287

H 22.848583 8.386581 48.831196

H 24.557875 8.390004 0.430257

O 14.752913 8.881866 21.172537

C 14.067436 8.544994 22.370899

C 14.609657 7.214622 22.818270

O 15.541065 6.820546 21.823790

C 15.908251 8.107404 21.392067

H 14.235627 9.344881 23.110649

H 12.991796 8.545972 22.154795

H 13.856709 6.419625 22.915567

H 15.125966 7.270848 23.794659

H 16.475895 8.026242 20.453814

H 16.578571 8.594865 22.133282

O 15.278510 16.570259 10.912384

C 15.720013 17.877163 10.555956

C 17.126659 18.009172 11.059551

O 17.253780 17.000027 12.045230

C 16.496429 16.029015 11.363665

H 15.609515 18.012596 9.471513

H 15.029158 18.593443 11.022392

H 17.366722 18.980673 11.507409

H 17.883520 17.844894 10.270421

H 16.275434 15.191483 12.037896

H 17.085100 15.611470 10.522220

O 39.842743 3.137940 40.963856

C 40.305759 4.282032 40.260288

C 40.593735 5.323448 41.307571

O 40.252953 4.742113 42.560204

C 39.318123 3.804838 42.085945

H 41.164803 3.982319 39.641792

H 39.528362 4.614991 39.550850

H 39.998222 6.242633 41.162357

H 41.642487 5.643207 41.360863

H 38.352001 4.299144 41.844414

H 39.111794 3.070468 42.876541

O 4.354393 10.357450 46.593861

C 5.394342 9.421642 46.811432

C 4.984132 8.636913 48.022511

O 3.936359 9.377639 48.642467

C 4.106018 10.597025 47.956017

H 5.543466 8.835907 45.895184

H 6.335041 9.972664 46.992825

H 5.806509 8.495613 48.743187

H 4.595434 7.635588 47.789780

H 4.928883 11.193518 48.406803

H 3.194166 11.196451 48.054779

O 19.272074 13.387827 5.380652

C 18.338223 12.359611 5.696011

C 18.838884 11.675112 6.932999

O 20.202017 12.043274 7.044475

C 20.091028 13.349201 6.527678

H 17.340321 12.807958 5.802109

H 18.283951 11.689290 4.826697

H 18.772390 10.577469 6.910019

H 18.304485 11.999760 7.842405

H 21.090887 13.722741 6.263657

H 19.685219 14.028322 7.302629

O 36.170406 30.178102 20.490971

C 35.062004 31.058662 20.326204

C 35.043427 31.460560 18.875555

O 36.245701 30.952074 18.315243

C 36.377708 29.824120 19.145441

H 34.140377 30.545288 20.646940

H 35.196457 31.893753 21.027327

H 35.005291 32.543537 18.690739

H 34.179977 31.035194 18.334311

H 37.392235 29.414886 19.038857

H 35.674633 29.021788 18.838396

O 18.099136 32.208622 40.444614

C 18.372936 32.032116 39.058502

C 19.202158 33.212391 38.634113

O 19.177711 34.112019 39.730289

C 19.101439 33.147850 40.752151

H 18.889244 31.069906 38.913288

H 17.408770 31.941175 38.537304

H 18.838884 33.742386 37.742306

H 20.250908 32.932236 38.420452

H 18.853064 33.641178 41.703114

H 20.082718 32.647678 40.895893

O 2.737997 9.883191 38.447830

C 2.587896 8.730787 37.631809

C 3.369692 7.634611 38.304085

O 3.884533 8.184655 39.509293

C 2.939435 9.213848 39.667217

H 1.514210 8.486323 37.545269

H 2.918900 8.984541 36.616306

H 4.218471 7.241513 37.731060

H 2.739464 6.760407 38.534370

H 3.312487 9.930127 40.409409

H 1.982603 8.809994 40.060314

O 15.197349 39.900925 11.618884

C 14.870256 39.388527 10.337893

C 15.828066 38.263016 10.137922

O 16.996603 38.589619 10.891360

C 16.598127 39.794827 11.507898

H 13.809771 39.100060 10.334472

H 14.990533 40.178146 9.571254

H 16.127289 38.086025 9.096994

H 15.430079 37.311073 10.524175

H 16.995138 40.654362 10.933408

H 17.046474 39.845188 12.509711

O 9.328746 21.241966 14.348082

C 10.069472 22.443262 14.233183

C 9.845543 22.873518 12.826537

O 8.547440 22.408548 12.461798

C 8.203234 21.690802 13.627400

H 9.700821 23.188387 14.962664

H 11.122133 22.253069 14.477158

H 10.572090 22.398281 12.149861

H 9.903236 23.957960 12.665680

H 7.595985 20.825888 13.334533

H 7.577406 22.335697 14.276209

O 10.564267 23.302797 4.901014

C 11.731828 24.053791 4.605702

C 12.728262 23.659225 5.649563

O 12.286271 22.410992 6.180538

C 11.218452 22.125458 5.310247

H 12.079944 23.812750 3.582864

H 11.479540 25.123077 4.605702

H 12.786445 24.375505 6.482696

H 13.753545 23.550684 5.269666

H 10.503640 21.473719 5.831933

H 11.581238 21.557325 4.431155

O 28.547527 8.275595 14.859989

C 29.794294 7.984684 14.238071

C 29.478447 7.360322 12.904766

O 28.065447 7.234668 12.850984

C 27.734930 8.296130 13.711496

H 30.374165 8.918535 14.144198

H 30.363407 7.338320 14.922571

H 29.918482 6.365354 12.738530

H 29.809940 7.977838 12.055009

H 26.682266 8.195900 14.008765

H 27.829781 9.268608 13.184432

O 45.940655 14.905458 5.251087

C 44.784824 14.624325 4.468313

C 43.654423 14.402352 5.429545

O 44.151176 14.823319 6.688046

C 45.478127 14.404797 6.481719

H 44.625923 15.477015 3.795059

H 44.984798 13.745722 3.833195

H 43.361557 13.336978 5.490662

H 42.733772 14.958263 5.201705

H 45.550976 13.296397 6.512521

H 46.113731 14.783227 7.292850

O 8.729810 13.157053 24.885456

C 8.626645 11.741606 24.827763

C 9.971198 11.200363 25.237974

O 10.805798 12.329786 25.448702

C 9.799095 13.251904 25.791929

H 7.811603 11.415491 25.497595

H 8.311776 11.476118 23.809326

H 10.461593 10.547644 24.503603

H 9.921816 10.612183 26.169382

H 10.213706 14.269854 25.753794

H 9.445600 13.081758 26.832367

O 47.919830 46.882328 2.538025

C 47.775112 47.842094 3.578464

C 48.142296 47.145859 4.860433

O 48.647358 45.870735 4.489337

C 47.887562 45.706947 3.312487

H 48.407291 48.706520 3.327644

H 46.741028 48.220524 3.582376

H 47.268093 47.010914 5.523420

H 0.011733 47.661678 5.461814

H 46.840279 45.428745 3.555484

H 48.308041 44.880657 2.720395

O 20.342339 11.975313 21.467361

C 20.026979 10.709479 22.050163

C 19.096550 10.027424 21.105066

O 19.437820 10.559378 19.838253

C 19.625080 11.892685 20.254332

H 20.969145 10.151611 22.159195

H 19.626059 10.866914 23.060778

H 18.037043 10.248420 21.329483

H 19.193357 8.933693 21.073774

H 18.643803 12.394325 20.361897

H 20.174635 12.445173 19.480358

O 21.775385 10.610227 15.427145

C 21.556347 9.383995 16.102842

C 21.476652 9.750202 17.554470

O 21.249300 11.157825 17.608742

C 20.967188 11.411579 16.253433

H 20.619560 8.923914 15.736148

H 22.364056 8.687273 15.838334

H 22.401215 9.534585 18.108427

H 20.668943 9.244651 18.101580

H 21.167160 12.471576 16.039282

H 19.890568 11.234098 16.041239

O 14.146153 42.838402 8.039443

C 15.465281 43.339066 8.157763

C 15.517597 44.463112 7.178441

O 14.564187 44.167801 6.156581

C 14.064503 42.934235 6.635731

H 15.647162 43.643669 9.196735

H 16.195251 42.539181 7.927967

H 16.501808 44.628857 6.723249

H 15.217884 45.412121 7.646833

H 14.614546 42.094254 6.166360

H 13.016241 42.831070 6.328684

O 11.276146 24.170155 10.415144

C 12.490154 23.439697 10.320292

C 12.296539 22.445217 9.211404

O 10.990613 22.673058 8.697540

C 10.862024 24.019566 9.081837

H 13.322310 24.132509 10.098808

H 12.702349 23.012863 11.309393

H 12.369878 21.388645 9.501338

H 13.044110 22.587008 8.411517

H 9.808384 24.316833 8.999698

H 11.442871 24.686462 8.407605

O 8.508814 10.652274 0.929452

C 7.261069 10.933408 1.550880

C 7.574472 11.470250 2.922812

O 8.986985 11.404735 3.050911

C 9.300877 11.536256 1.685824

H 6.719337 11.658978 0.925052

H 6.655777 10.015690 1.541590

H 7.125637 10.914340 3.758389

H 7.250313 12.518023 3.049444

H 10.361852 11.291792 1.533278

H 9.157621 12.585007 1.343085

O 17.659590 38.551971 45.435589

C 18.955738 38.979782 45.815975

C 18.836929 39.378262 47.257336

O 17.531981 38.996895 47.678303

C 17.259647 38.043488 46.682846

H 19.673485 38.154472 45.666367

H 19.268652 39.776737 45.127075

H 18.942049 40.455860 47.431885

H 19.581076 38.885910 47.905163

H 16.179115 37.840096 46.661823

H 17.767155 37.079323 46.905796

O 17.283115 20.207882 40.700809

C 18.175409 21.232187 41.136932

C 17.320763 22.334232 41.684044

O 16.041239 22.117636 41.112976

C 16.073509 20.713434 41.216629

H 18.880444 20.813175 41.870327

H 18.777769 21.526033 40.267132

H 17.638567 23.356579 41.445938

H 17.257202 22.285828 42.789513

H 15.237441 20.285624 40.647518

H 15.937098 20.397099 42.273689

O 34.261627 10.960299 44.821495

C 35.608624 11.242410 44.466534

C 35.747971 12.741953 44.477291

O 34.465023 13.253860 44.800472

C 33.975605 12.147904 45.516262

H 36.281391 10.748104 45.187214

H 35.800774 10.765706 43.495033

H 36.062351 13.190788 43.524372

H 36.469139 13.102781 45.229752

H 32.888229 12.257915 45.623825

H 34.399017 12.114657 46.542522

O 39.235493 34.908970 26.516520

C 39.462357 34.660103 25.140676

C 38.394535 35.424297 24.416574

O 37.887032 36.385532 25.339672

C 38.834084 36.251076 26.375219

H 40.475414 35.011646 24.871767

H 39.453556 33.574684 24.971508

H 37.553581 34.794071 24.091438

H 38.753410 35.949898 23.519392

H 38.387203 36.595772 27.316895

H 39.711708 36.902817 26.181118

O 40.412834 25.769928 7.186264

C 40.266155 27.186352 7.232223

C 41.656666 27.757910 7.144704

O 42.544071 26.654888 7.222933

C 41.691380 25.729836 6.599550

H 39.630547 27.519312 6.391756

H 39.715134 27.444017 8.148474

H 41.935352 28.478588 7.925034

H 41.811165 28.268839 6.179561

H 42.123589 24.728512 6.715915

H 41.619995 25.928829 5.508751

O 19.598679 43.422184 32.868183

C 18.716164 44.232826 32.092747

C 18.902933 43.823593 30.666540

O 19.407997 42.501045 30.734015

C 20.247486 42.694172 31.849747

H 17.693815 44.048012 32.453083

H 18.928846 45.293312 32.290760

H 19.624102 44.475338 30.137032

H 17.982283 43.820660 30.066137

H 21.175470 43.217815 31.543190

H 20.550621 41.719738 32.258003

O 32.879917 2.266181 15.961054

C 32.766979 1.920998 14.586189

C 33.942848 2.581051 13.911957

O 34.696774 3.164831 14.969997

C 34.285587 2.288183 15.989901

H 32.796310 0.821888 14.494760

H 31.773476 2.217288 14.224382

H 33.703762 3.369203 13.183944

H 34.571609 1.864771 13.354091

H 34.631260 2.670036 16.960911

H 34.722198 1.269257 15.870604

O 45.468838 19.322924 33.163498

C 44.093479 19.642195 33.000683

C 43.867107 19.758070 31.516296

O 45.157391 19.656860 30.929585

C 45.682007 18.775812 31.889351

H 43.479389 18.848175 33.461742

H 43.895958 20.556978 33.575665

H 43.398716 20.693876 31.179916

H 43.224655 18.943026 31.128088

H 46.759605 18.649670 31.725071

H 45.224861 17.765686 31.796455

O 17.938280 7.372056 28.988539

C 18.838884 8.200300 28.268351

C 18.712252 9.580544 28.854574

O 17.710926 9.501338 29.857855

C 17.863962 8.136250 30.166857

H 19.859278 7.795957 28.367603

H 18.585131 8.123539 27.201509

H 18.412050 10.355494 28.137806

H 19.654905 9.932083 29.312212

H 16.995138 7.802802 30.750147

H 18.766033 7.974905 30.797573

O 15.160191 30.402033 41.392643

C 13.889956 29.979109 41.863483

C 13.226969 31.211208 42.411079

O 14.223892 32.228664 42.441395

C 15.365051 31.408735 42.352409

H 13.353112 29.487738 41.040615

H 14.029788 29.202204 42.636967

H 12.826537 31.065994 43.429028

H 12.388947 31.579859 41.800411

H 15.616361 30.966745 43.340530

H 16.224098 32.025272 42.057095

O 40.387897 21.824280 39.361637

C 41.746628 21.870237 38.984184

C 42.497131 21.956289 40.282776

O 41.538834 22.270182 41.287525

C 40.509151 22.718039 40.438255

H 41.963711 20.997013 38.356400

H 41.920685 22.758131 38.343689

H 43.263771 22.743464 40.267620

H 43.006107 21.028793 40.581024

H 40.696411 23.752611 40.071072

H 39.566498 22.760576 40.997589

O 33.269592 21.730404 3.467966

C 32.678970 22.210533 2.268626

C 33.202614 21.330462 1.168538

O 33.975605 20.316914 1.798766

C 33.376183 20.385853 3.069979

H 31.578390 22.149416 2.347832

H 32.920498 23.277374 2.168885

H 33.847507 21.850681 0.448836

H 32.395393 20.857668 0.580846

H 34.002499 19.842165 3.790170

H 32.378281 19.893991 3.068512

O 42.163193 8.850574 22.160662

C 42.940590 8.303953 21.109467

C 43.808437 9.431910 20.621517

O 43.697449 10.480171 21.580303

C 43.122471 9.758513 22.645189

H 43.532681 7.456641 21.503054

H 42.259514 7.877119 20.359451

H 43.509212 9.842120 19.645615

H 44.868923 9.153221 20.519819

H 42.643810 10.462570 23.339956

H 43.902798 9.228516 23.229458

O 21.422869 8.726387 24.038143

C 20.827354 8.065356 25.145567

C 21.916687 7.896676 26.170361

O 23.074957 8.498547 25.610538

C 22.747374 8.307864 24.259140

H 19.965864 8.664293 25.470215

H 20.415678 7.097768 24.809185

H 22.125458 6.832769 26.385487

H 21.728449 8.371426 27.143326

H 22.885252 7.245424 23.960894

H 23.426985 8.904845 23.634779

O 23.708118 14.546097 23.689539

C 23.679272 15.593870 24.646862

C 22.478954 15.344027 25.520086

O 21.819880 14.211670 24.971022

C 22.935612 13.594644 24.375505

H 24.629749 15.593870 25.208149

H 23.648470 16.541901 24.091438

H 21.754852 16.170315 25.564579

H 22.758619 15.134277 26.567369

H 22.587494 12.831426 23.664114

H 23.557528 13.072467 25.129919

O 40.201614 46.985493 4.560720

C 38.844353 46.726360 4.239006

C 38.857552 45.960697 2.943835

O 40.228020 45.859493 2.571272

C 40.736015 45.877579 3.884533

H 38.389648 46.151867 5.064805

H 38.315331 47.686615 4.195491

H 38.300175 46.440826 2.129281

H 38.416050 44.951061 3.049444

H 41.827301 45.960697 3.880621

H 40.517464 44.927593 4.411108

O 40.806908 44.082237 37.741329

C 39.836384 43.156208 37.247025

C 40.574177 41.916775 36.864681

O 41.728050 41.938778 37.686081

C 41.981804 43.318531 37.581940

H 39.120598 42.963570 38.060600

H 39.279007 43.625580 36.425137

H 40.868027 41.912865 35.797352

H 40.020710 40.982433 37.039230

H 42.449219 43.549793 36.604084

H 42.701992 43.609444 38.358356

O 36.287258 29.678907 4.190113

C 35.618893 30.527685 3.271906

C 36.132755 30.138988 1.913175

O 37.277828 29.314167 2.128793

C 37.501266 29.599213 3.489968

H 34.536407 30.409855 3.418096

H 35.848202 31.583281 3.513437

H 36.422691 31.006836 1.297615

H 35.413544 29.561563 1.313749

H 38.075268 30.541376 3.604866

H 38.110958 28.798838 3.928047

Li 21.897617 20.825888 34.255272

Li 18.914179 18.484413 34.542274

Li 25.088362 25.303492 8.158253

Li 15.140143 26.610395 21.302103

Li 29.016409 6.993137 31.052307

Li 24.076769 25.005732 45.602802

Li 12.335165 33.130737 10.926563

Li 14.370572 42.894630 14.951418

Li 44.110107 19.174778 6.545280

Li 5.804064 32.964016 12.104390

Li 3.078291 25.895584 9.626015

Li 46.917042 22.044785 44.695351

Li 40.873894 10.728058 14.528984

Li 48.433697 16.007502 30.312557

Li 22.113724 23.039265 19.408485

Li 32.121105 38.355911 32.564072

Li 46.153824 21.437536 31.046438

Li 9.742867 44.980888 28.201366

Li 33.472988 19.996666 11.682446

Li 2.720395 3.437653 16.402067

Li 6.184450 34.575520 45.806686

Li 14.779316 23.464632 40.270065

Li 42.406193 9.246606 46.753250

Li 5.881804 29.499470 1.553324

Li 28.948938 8.751323 21.707914

Li 28.761679 45.080627 3.030376

Li 16.836723 5.594803 11.073241

Li 46.848103 25.681919 24.870298

Li 23.618156 19.846075 46.383621

Li 7.305562 41.477715 35.780239

Li 37.841560 45.656094 34.743710

Li 23.613266 25.928829 26.254454

Li 27.758400 11.503009 11.880462

Li 27.478241 39.607079 6.621552

Li 8.975739 40.263218 45.809132

Li 31.809166 39.634457 6.433803

Li 37.591717 0.375986 1.226719

Li 9.332658 45.143211 21.950422

Li 27.611719 0.533909 21.915220

Li 27.494377 16.502298 38.231236

Li 7.472775 10.415633 40.930119

Li 18.226257 19.730200 25.375853

Li 36.663242 17.558870 31.265966

Li 15.731747 29.886213 28.572462

Li 31.660532 32.871609 7.575939

Li 18.382713 47.638210 46.873528

Li 5.066761 18.375381 4.440933

Li 35.200859 44.917812 8.315687

Li 36.453003 26.533632 35.463413

Li 43.703808 32.306404 1.934199

Li 19.237362 15.864736 40.501816

Li 33.096512 4.264919 15.472616

Li 42.564114 20.730059 14.748024

Li 13.393206 9.306255 19.565922

Li 0.538310 7.768088 2.097501

Li 39.058014 26.439270 13.262173

Li 48.077759 44.665039 22.109324

Li 17.620476 24.275764 17.652746

Li 31.485987 44.484135 30.123344

Li 14.362749 10.590180 39.457466

Li 29.152821 4.619881 48.039131

Li 37.380013 37.458729 20.323271

Li 6.313528 47.876320 1.642798

Li 4.522095 43.563972 41.029858

Li 33.297951 41.242542 19.421686

Li 8.865731 7.413126 19.043257

Li 47.120434 27.876230 20.304203

Li 41.015682 7.706483 29.508270

Li 15.765484 17.386280 30.931051

Li 38.734833 21.063507 8.697540

Li 0.118810 44.309101 6.682668

Li 23.634291 8.143096 6.386378

Li 37.165371 29.831453 27.476776

Li 34.562809 3.655715 36.561058

Li 4.253674 4.914704 30.801973

Li 19.464712 8.472633 2.368856

Li 0.301180 19.073082 23.983385

Li 43.580109 2.700349 43.676426

Li 13.961828 47.495934 6.380510

Li 15.113742 1.033594 40.667076

Li 4.129974 4.005787 26.968290

Li 19.230028 20.364340 30.700766

Li 41.290947 47.666080 1.525458

Li 16.538479 5.519019 44.542316

Li 26.933577 42.426727 14.290387

Li 38.466412 31.392599 44.564808

Li 25.629606 30.844023 38.363247

Li 7.096301 0.657609 42.233601

Li 23.101847 45.001419 33.887600

Li 14.615524 13.665049 4.315767

Li 23.742344 4.229227 34.302208

Li 33.769279 11.731828 25.935184

Li 14.451244 4.385684 32.743507

Li 7.533403 7.421438 5.779618

Li 12.419749 4.213093 47.523312

Li 0.709435 35.415497 32.377300

Li 29.318569 37.173683 15.207617

Li 11.154403 18.880932 48.560329

Li 17.724129 47.077412 20.854246

Li 14.014143 13.362402 44.961819

N 13.204477 4.035123 42.319653

S 13.625935 2.902766 43.195812

S 14.333902 4.823763 41.696270

O 14.733845 2.154217 42.625717

O 12.504823 2.053498 43.517525

O 15.505373 4.904437 42.557758

O 13.874310 6.114044 41.247433

C 14.150064 3.802882 44.741798

C 14.722600 3.795059 40.204548

F 15.444747 3.561840 45.027332

F 14.022454 5.139122 44.639126

F 13.431830 3.446942 45.824776

F 15.997235 3.363336 40.240730

F 14.551475 4.508405 39.086861

F 13.963294 2.677370 40.097473

N 10.990123 35.387630 30.935452

S 9.816208 36.085331 31.560303

S 11.051240 35.445324 29.431509

O 9.497426 37.333565 30.897316

O 10.017646 36.258900 32.976238

O 9.734556 35.460480 28.826706

O 11.900996 34.400482 28.917158

C 8.418851 34.875721 31.327572

C 11.883883 37.076385 29.103930

F 7.405304 35.447769 30.646496

F 8.762568 33.743855 30.679253

F 7.904010 34.501202 32.516644

F 12.215377 37.758930 30.219662

F 11.095243 37.879208 28.362713

F 13.023575 36.908195 28.401829

N 34.294876 43.074554 44.608326

S 34.545696 43.969784 43.435383

S 33.335110 43.581085 45.644363

O 35.720589 43.568375 42.724483

O 34.586769 45.378872 43.816261

O 32.322052 44.457733 45.057159

O 32.788490 42.516689 46.415401

C 33.065224 43.699894 42.338230

C 34.438133 44.601479 46.743473

F 32.131371 42.876541 42.846230

F 32.463840 44.871368 42.050739

F 33.449032 43.179676 41.153557

F 35.734772 44.603924 46.359177

F 34.421997 44.133575 48.012238

F 34.037701 45.887360 46.800678

N 39.247719 12.910144 9.910571

S 38.969517 11.515721 9.391329

S 38.490368 13.273907 11.164182

O 39.400749 11.397400 8.015486

O 37.599541 11.124578 9.595212

O 39.103485 14.419464 11.798322

O 38.338799 12.161595 12.074566

C 40.118500 10.476260 10.429323

C 36.831436 13.820039 10.522709

F 41.010792 9.821586 9.654860

F 40.848469 11.202807 11.309393

F 39.456978 9.554142 11.150981

F 36.716045 13.760389 9.180601

F 36.600170 15.108364 10.866425

F 35.836956 13.090069 11.061506

N 11.887306 15.082940 6.261701

S 12.503356 13.840085 6.844014

S 11.087909 15.935630 7.219022

O 12.906721 13.989697 8.222302

O 13.573618 13.392715 5.975678

O 10.330560 15.139166 8.153363

O 10.264554 16.865570 6.480741

C 11.136802 12.582561 6.734005

C 12.377212 16.911041 8.143096

F 9.968264 13.074424 6.277835

F 11.489808 11.581726 5.898427

F 10.897227 12.019317 7.932368

F 13.638647 16.633331 7.766132

F 12.289205 16.698357 9.472490

F 12.195820 18.235058 7.950947

N 0.657608 30.342381 4.224338

S 1.480474 31.157913 5.185570

S 1.087865 30.440659 2.785423

O 2.876852 31.224897 4.813985

O 1.286370 30.678766 6.533545

O 1.543057 31.762720 2.422149

O 0.051826 29.941460 1.914642

C 0.696722 32.839336 5.027647

C 2.508689 29.246695 2.728707

F 1.600261 33.758522 4.641394

F 48.564728 32.883831 4.146109

F 0.189215 33.249550 6.208408

F 3.665004 29.825098 2.326319

F 2.240268 28.263948 1.853037

F 2.750220 28.641401 3.912402

N 40.972164 39.902878 17.248402

S 39.875988 38.887863 17.157461

S 41.701645 39.945904 18.561172

O 40.186459 37.659191 17.857607

O 39.489246 38.674206 15.787974

O 40.832821 39.661350 19.683752

O 42.408150 41.193649 18.698563

C 38.460056 39.732735 18.013086

C 42.964550 38.593044 18.376358

F 38.742653 40.966789 18.479523

F 37.418636 39.872566 17.166262

F 38.013172 38.998363 19.056458

F 42.860893 37.668968 19.351282

F 44.211803 39.103973 18.447252

F 42.880451 37.940815 17.200487

N 8.985030 47.970192 4.475647

S 9.661218 47.018250 3.520281

S 7.502600 47.757999 4.664373

O 9.134152 45.682007 3.615134

O 11.098665 47.087189 3.660115

O 6.854282 47.352188 3.431297

O 6.898774 0.046937 5.227618

C 9.214827 47.717903 1.855971

C 7.385746 46.394375 5.919451

F 8.435474 48.822395 1.923932

F 10.313447 48.037178 1.150936

F 8.514681 46.827568 1.120623

F 8.580197 45.958252 6.355086

F 6.716893 45.341717 5.410966

F 6.694402 46.815346 6.995093

N 14.251761 26.666620 31.045950

S 13.416183 25.430124 31.274769

S 13.733009 27.593142 29.995243

O 12.004160 25.642805 31.054262

O 13.714919 24.853186 32.560162

O 13.196656 26.886150 28.857019

O 14.751935 28.554861 29.612413

C 14.055214 24.252783 29.988886

C 12.382102 28.522591 30.872868

F 13.071979 23.816172 29.174824

F 15.023290 24.781315 29.217360

F 14.573965 23.145851 30.570223

F 12.226623 28.169096 32.163151

F 11.197918 28.365648 30.252909

F 12.659812 29.843187 30.856735

N 20.508574 17.791601 43.524857

S 20.984301 18.355824 44.845455

S 19.312166 16.892462 43.634377

O 19.894970 18.569485 45.769039

O 21.821346 19.518005 44.654770

O 19.353725 16.044172 44.803894

O 19.150820 16.134134 42.415970

C 22.088789 17.019094 45.497684

C 17.889875 18.088869 43.744877

F 21.649731 16.554125 46.676491

F 22.240847 15.966433 44.666992

F 23.315510 17.538336 45.721123

F 18.254126 19.383551 43.679848

F 17.178486 17.921167 44.872833

F 17.042562 17.869341 42.715195

N 32.902412 17.944147 33.232437

S 31.798410 18.964050 33.406494

S 33.305286 17.694792 31.811611

O 30.632805 18.646248 32.609051

O 32.283428 20.309580 33.238792

O 34.673794 17.204887 31.745607

O 33.109715 18.836439 30.951097

C 31.359840 18.722031 35.191570

C 32.109367 16.353662 31.299702

F 32.169994 17.820936 35.766548

F 31.456160 19.858299 35.914696

F 30.093031 18.270750 35.355358

F 31.220497 16.011904 32.260933

F 32.754265 15.220329 30.942297

F 31.376953 16.736982 30.231884

N 13.800482 46.460384 15.562578

S 13.345289 45.775883 14.300655

S 12.950725 46.178761 16.784899

O 12.946324 44.406399 14.565165

O 14.366172 45.851177 13.284663

O 11.554346 46.018879 16.463182

O 13.196656 47.155640 17.818003

C 11.907352 46.805569 13.725676

C 13.616644 44.552586 17.392147

F 11.675601 47.876804 14.507961

F 12.127859 47.279827 12.478420

F 10.780373 46.076572 13.675805

F 12.649056 43.616287 17.424416

F 14.106061 44.667973 18.642824

F 14.610636 44.057301 16.623062

N 26.529232 6.535501 31.928463

S 27.072432 5.155746 31.744627

S 25.778238 6.747206 33.230968

O 27.465530 4.557298 32.995796

O 28.152964 5.210505 30.787796

O 25.045338 5.567912 33.661716

O 24.940706 7.915255 33.154697

C 25.629606 4.237050 31.018568

C 27.182440 7.101190 34.394127

F 25.256552 3.212746 31.808187

F 24.547119 5.020802 30.850380

F 25.943010 3.734432 29.811897

F 28.386181 7.042519 33.780037

F 27.197107 6.227965 35.416965

F 27.073900 8.335733 34.913368

N 5.213439 26.071108 48.090958

S 4.542630 25.579735 46.819256

S 4.363682 26.941399 0.080672

O 3.147718 25.253620 47.026562

O 5.290689 24.491381 46.240856

O 3.410273 27.736887 48.243504

O 5.205616 27.737373 0.953900

C 4.698598 27.034296 45.676632

C 3.464055 25.698055 1.133825

F 5.287267 28.089401 46.267746

F 5.443724 26.728714 44.595123

F 3.491924 27.433260 45.230732

F 3.762301 24.418531 0.837045

F 2.122925 25.811977 1.049726

F 3.792614 25.862335 2.432905

N 22.075588 20.578001 29.741001

S 23.028019 19.418753 29.871546

S 21.896151 21.401846 30.984833

O 24.105618 19.685219 30.782415

O 23.457787 18.959160 28.571486

O 21.895662 20.611738 32.199329

O 20.667963 22.146482 30.857712

C 21.902018 18.121628 30.576578

C 23.337999 22.571362 30.986300

F 22.332275 17.678659 31.771521

F 20.646452 18.592953 30.751616

F 21.803255 17.053808 29.760559

F 24.197535 22.370899 29.972263

F 24.011742 22.449129 32.139683

F 22.920944 23.847462 30.917850

N 25.024313 20.960833 9.129264

S 26.248098 21.792498 9.336569

S 25.252642 19.607967 8.493168

O 27.389746 21.031727 9.796162

O 25.945452 22.914099 10.196104

O 26.452471 19.554188 7.686926

O 24.064058 19.184067 7.795468

C 26.561501 22.492643 7.643900

C 25.444792 18.501524 9.977065

F 25.718100 22.063854 6.684624

F 26.423626 23.840130 7.678614

F 27.818537 22.234489 7.247869

F 25.418388 19.165977 11.152937

F 26.603550 17.816536 9.936973

F 24.451777 17.587717 10.028402

N 32.083942 46.537636 11.414023

S 32.732262 47.591763 10.565734

S 32.628120 46.402687 12.805024

O 32.419838 47.381031 9.177668

O 34.163357 47.673412 10.791618

O 31.689379 45.731392 13.661138

O 33.089668 47.666080 13.346267

C 31.903040 0.266466 11.149513

C 34.089527 45.285488 12.556648

F 31.017591 0.081651 12.156217

F 32.811958 1.161201 11.590038

F 31.226366 0.866869 10.153079

F 34.294388 44.930527 11.267835

F 33.950672 44.141396 13.258750

F 35.217484 45.876114 12.994239

N 4.143176 31.906464 15.756193

S 2.972682 32.038963 16.723293

S 3.769146 31.880060 14.312389

O 1.830546 32.700481 16.142937

O 3.415162 32.615406 17.971037

O 2.633855 31.028839 14.050323

O 4.917638 31.567148 13.476322

C 2.499889 30.275400 17.060654

C 3.362847 33.648514 13.945204

F 1.201785 30.045603 16.764364

F 3.227902 29.373327 16.374687

F 2.656835 29.990843 18.368048

F 3.520771 34.483112 14.980754

F 2.121459 33.778568 13.459700

F 4.218960 34.061169 12.982505

N 11.747962 22.796757 42.281025

S 12.713105 23.839640 42.797821

S 11.785121 22.541536 40.800552

O 12.025673 24.797939 43.624111

O 13.527171 24.435154 41.749073

O 10.944164 21.418959 40.460747

O 13.138963 22.382633 40.302822

C 13.775546 22.804090 43.913071

C 11.022882 24.085571 40.083782

F 13.471922 21.486917 43.869553

F 15.058494 22.923388 43.537571

F 13.689494 23.174210 45.205791

F 10.649341 24.982754 41.013237

F 9.929150 23.793680 39.356258

F 11.889751 24.704065 39.257984

N 40.468082 22.245735 12.430506

S 40.591778 23.037310 13.692429

S 39.776737 22.941969 11.303038

O 40.747749 24.463022 13.452854

O 41.626842 22.454506 14.518718

O 38.692295 23.776079 11.767030

O 39.370438 21.985136 10.290468

C 38.950447 22.768398 14.519205

C 41.107597 24.020544 10.586758

F 38.115360 21.992470 13.799992

F 39.111794 22.171417 15.716102

F 38.336845 23.947205 14.734822

F 42.298630 23.872398 11.199385

F 40.763393 25.318649 10.666943

F 41.294861 23.732565 9.283765

N 44.070992 27.592651 37.407391

S 43.511658 26.223654 37.221111

S 43.033485 28.630646 37.794132

O 42.411079 26.186005 36.292637

O 43.271591 25.539644 38.475212

O 43.683273 29.776203 38.389160

O 41.996956 28.067402 38.626289

C 44.965240 25.383675 36.441757

C 42.328941 29.164066 36.153294

F 46.075596 26.145914 36.336151

F 45.292824 24.306078 37.181019

F 44.696819 24.909903 35.210640

F 42.921032 28.555349 35.101608

F 42.496155 30.491995 35.971409

F 41.008347 28.919600 36.065285

N 43.825062 18.324043 19.419241

S 43.858307 16.963356 18.761145

S 44.438175 19.432932 18.611532

O 43.668602 17.044029 17.324675

O 42.935211 16.062262 19.402618

O 45.582268 18.986052 17.846361

O 44.722240 20.580936 19.429020

C 45.582268 16.360020 19.130774

C 43.073578 19.908659 17.441528

F 46.326904 17.228846 19.845587

F 45.543156 15.212996 19.843142

F 46.258942 16.091599 17.995974

F 43.462276 19.758070 16.153692

F 42.745018 21.214098 17.564739

F 41.935352 19.208515 17.599451

N 43.844616 4.053213 47.241688

S 42.931789 3.074379 46.564526

S 45.195526 3.513437 47.633320

O 43.634377 2.219244 45.627739

O 41.869839 3.780391 45.895184

O 45.153481 2.131237 48.043041

O 45.825264 4.388129 48.597488

C 42.221378 2.086745 47.973125

C 46.140137 3.652292 46.040394

F 42.702484 2.449040 0.284068

F 40.884647 2.235379 48.021530

F 42.470730 0.772995 47.811779

F 46.616840 2.457352 45.650230

F 47.190350 4.480047 46.190002

F 45.400387 4.132908 45.012665

N 33.782967 27.909966 27.990637

S 32.770889 27.770622 29.105885

S 35.182281 27.488022 28.341690

O 33.003128 26.603062 29.919947

O 31.427315 27.806801 28.571972

O 35.521595 27.717329 29.725845

O 36.125423 28.129496 27.449396

C 33.062778 29.330791 30.084229

C 35.155876 25.665297 27.968637

F 34.051880 30.101341 29.571344

F 31.953402 30.097431 30.090586

F 33.381557 29.074591 31.367176

F 35.505459 24.935818 29.042812

F 36.040348 25.373898 26.987848

F 33.955563 25.209129 27.541315

N 28.099182 30.384920 8.061934

S 27.649855 29.392885 9.102861

S 29.392885 31.064039 8.383648

O 28.743589 28.806170 9.849455

O 26.789831 28.389116 8.531304

O 29.523918 31.354954 9.792739

O 29.624147 32.232578 7.561272

C 26.616262 30.451414 10.222995

C 30.624495 29.775717 7.860006

F 26.539011 31.735826 9.832831

F 25.350428 29.981066 10.248909

F 27.070477 30.424032 11.490786

F 30.075428 28.667315 7.313874

F 31.384289 29.380173 8.888222

F 31.455673 30.299358 6.937399

N 1.403712 15.194415 26.611862

S 0.565690 16.331661 27.126213

S 2.310185 14.555386 27.629810

O 0.232730 16.209919 28.531391

O 48.302662 16.554613 26.296503

O 2.850450 15.519552 28.563175

O 3.323244 13.739366 27.003981

C 1.678490 17.789156 26.876373

C 1.143603 13.424007 28.526014

F 2.874897 17.463530 26.362019

F 1.100088 18.663361 26.015860

F 1.884817 18.454586 28.029263

F 1.061463 13.774080 29.828030

F 1.568481 12.146927 28.482500

F 48.783279 13.450409 28.035131

N 10.313936 3.906534 46.677467

S 10.018623 5.369896 46.875481

S 9.231938 2.951169 47.135593

O 11.206718 5.979100 47.404991

O 8.842752 5.637829 47.666565

O 9.495470 1.626663 46.632484

O 7.895209 3.386315 46.805077

C 9.761936 5.950743 45.128544

C 9.503782 2.947747 0.080672

F 9.857766 4.956263 44.223049

F 8.548906 6.512032 44.968666

F 10.684054 6.869927 44.787270

F 10.500707 3.777458 0.462035

F 9.851410 1.716626 0.490886

F 8.394894 3.294397 0.755883

N 33.218746 37.225510 19.408976

S 34.090996 38.251770 20.038713

S 33.368847 35.858959 20.027468

O 34.196602 39.421772 19.174290

O 35.386654 37.747688 20.446968

O 34.559875 35.202816 19.578632

O 33.218746 35.908829 21.456116

C 33.121937 38.821861 21.507454

C 31.907930 34.956886 19.296032

F 31.917707 38.240036 21.671734

F 33.815727 38.692783 22.649590

F 32.888721 40.146366 21.335840

F 31.169159 35.735748 18.493702

F 31.108532 34.490936 20.271444

F 32.320587 33.896889 18.573885

N 26.027592 14.536807 10.816554

S 26.688623 14.615524 12.163062

S 26.229031 13.271461 10.063604

O 26.975136 13.299331 12.735596

O 25.936165 15.457947 13.050956

O 27.550602 12.698438 10.289979

O 25.885313 13.396626 8.676517

C 28.299641 15.456481 11.772897

C 24.975418 12.169907 10.883048

F 28.504992 15.654007 10.460614

F 28.410629 16.643597 12.399702

F 29.322969 14.698153 12.221245

F 25.618851 11.138268 11.477096

F 24.108061 11.649199 9.997110

F 24.250828 12.773243 11.847215

N 17.533936 9.089661 4.061036

S 17.344721 7.868807 4.937195

S 16.289125 9.508672 3.298797

O 15.998701 7.738264 5.443235

O 18.347511 7.822848 5.970300

O 15.540088 8.375337 2.817692

O 16.648975 10.440080 2.257380

C 17.712395 6.505676 3.730031

C 15.297580 10.443013 4.570010

F 16.654844 5.713613 3.510503

F 18.123093 6.981892 2.535581

F 18.699051 5.716057 4.189624

F 14.068414 9.917416 4.734779

F 15.122543 11.730849 4.188158

F 15.887715 10.495817 5.780596

N 17.199020 4.083527 35.051739

S 17.184351 3.812660 33.575172

S 15.844200 4.375416 35.639427

O 15.955188 3.165320 33.159588

O 18.378313 3.150163 33.126339

O 14.947995 5.025691 34.694328

O 15.970833 5.093163 36.883259

C 17.184351 5.532220 32.883831

C 15.190992 2.670525 35.989010

F 16.026083 5.733170 32.213020

F 17.262581 6.478296 33.835285

F 18.187143 5.740015 32.018917

F 14.050323 2.468597 35.295223

F 14.898125 2.505267 37.292496

F 16.044172 1.684846 35.650673

N 0.598934 25.120142 21.412601

S 0.811131 25.176369 19.918438

S 48.065044 25.077116 21.838947

O 48.709450 24.412174 19.201181

O 2.162528 24.813585 19.582544

O 47.222622 25.946430 21.034170

O 47.951126 25.362162 23.252926

C 0.544665 26.976603 19.541473

C 47.614254 23.290085 21.587149

F 48.368668 27.130613 18.724476

F 0.252286 27.709503 20.641562

F 1.595860 27.535934 18.938137

F 48.626823 22.517090 21.142714

F 46.587994 23.161009 20.729080

F 47.227020 22.754709 22.761065

N 13.042154 31.866859 14.352970

S 11.796366 31.623375 13.575575

S 14.002897 32.829559 13.713452

O 11.483452 32.732754 12.705771

O 10.696278 31.235653 14.427287

O 14.010720 32.734707 12.261335

O 15.314202 32.721016 14.307499

C 12.275515 30.174681 12.525357

C 13.267061 34.460136 14.209226

F 12.215377 30.511063 11.219431

F 13.526194 29.724377 12.755643

F 11.431625 29.141575 12.708706

F 12.129326 34.339371 14.921594

F 12.974194 35.199394 13.117450

F 14.117308 35.175926 14.964619

N 26.754627 30.228464 19.777626

S 27.973038 31.115866 19.841187

S 25.460436 30.955500 19.979065

O 29.092684 30.469013 19.191891

O 27.715860 32.436947 19.330746

O 24.397995 30.012356 20.268511

O 25.553331 31.993982 20.974033

C 28.326532 31.199963 21.668800

C 25.124542 31.696712 18.299597

F 28.286442 32.468731 22.124481

F 29.564987 30.734015 21.930864

F 27.473352 30.470482 22.423216

F 25.037516 33.040775 18.355335

F 23.934982 31.259121 17.832670

F 26.043728 31.387709 17.359879

N 36.378201 26.808411 39.217403

S 35.092319 26.712582 38.451252

S 37.625942 26.627995 38.390625

O 35.223351 27.092968 37.058788

O 34.027920 27.411259 39.142597

O 37.417660 25.753794 37.256313

O 38.736790 26.234900 39.228161

C 34.740780 24.895237 38.569576

C 37.904629 28.360268 37.770664

F 35.661919 24.221004 39.281944

F 33.559040 24.657618 39.172909

F 34.676239 24.329546 37.348232

F 36.971756 29.234962 38.201900

F 37.845470 28.360758 36.421227

F 39.100548 28.838928 38.138828

N 6.752096 34.037212 47.624031

S 7.049364 34.928528 48.800880

S 7.542203 32.739594 47.599586

O 7.338809 34.181442 1.111823

O 5.993768 35.895138 0.072361

O 8.887733 32.895565 48.109535

O 7.506512 32.227200 46.259434

C 8.567485 35.821796 48.208302

C 6.567770 31.627287 48.731941

F 9.661706 35.558266 0.044495

F 8.847641 35.541645 46.918018

F 8.358224 37.154617 48.259148

F 5.465726 32.230625 0.331005

F 7.285516 31.196539 0.894741

F 6.143381 30.506662 48.113937

N 26.810856 37.562382 32.215954

S 25.409588 38.043488 31.990070

S 27.214220 36.356197 31.404335

O 24.984219 37.906097 30.612759

O 25.241884 39.390972 32.484375

O 26.119511 35.430656 31.202894

O 28.394003 35.734280 31.954866

C 24.390661 36.921398 33.069622

C 27.694347 37.110611 29.772781

F 23.463655 36.263790 32.342587

F 25.097652 36.003193 33.755100

F 23.708609 37.652344 33.979519

F 27.574074 38.456631 29.729265

F 26.956066 36.615326 28.758743

F 28.981207 36.824589 29.483337

N 39.036991 17.365744 28.274216

S 39.802650 16.262722 28.974361

S 38.675182 18.540638 29.142065

O 40.733570 16.760450 29.955153

O 40.434834 15.410032 27.987705

O 38.311909 18.138741 30.475372

O 37.648922 19.328791 28.504013

C 38.471786 15.280955 29.848076

C 40.239265 19.529251 29.207092

F 37.236755 15.826110 29.750290

F 38.395515 14.018544 29.382128

F 38.729942 15.185125 31.169159

F 41.234722 18.958183 28.503523

F 40.664631 19.643171 30.480259

F 40.080364 20.774549 28.721098

N 16.404512 8.593398 47.205997

S 17.459129 7.642923 46.749828

S 15.373363 8.023798 48.148163

O 18.158785 8.173409 45.607693

O 16.924730 6.320861 46.451092

O 14.713310 9.079392 48.874706

O 15.887226 6.953534 0.078719

C 18.631578 7.607231 48.200478

C 14.132464 7.314363 46.969357

F 18.283463 8.410050 0.339316

F 18.753323 6.359487 48.687939

F 19.862700 8.004729 47.822048

F 14.505516 7.414593 45.676144

F 12.952192 7.948991 47.083767

F 13.920758 6.000613 47.219200

N 13.046556 26.351751 21.153959

S 12.834359 24.865902 21.234142

S 11.830591 27.216175 21.453671

O 12.133726 24.480135 22.441307

O 14.108994 24.222960 21.094799

O 10.577957 26.494520 21.343662

O 11.885351 28.416006 20.673342

C 11.840369 24.439066 19.715044

C 12.110746 27.695326 23.222614

F 11.513276 25.509817 18.968451

F 12.559582 23.622066 18.910757

F 10.712412 23.774124 20.015245

F 13.215234 27.129148 23.744299

F 11.068841 27.355520 23.995119

F 12.267693 29.032055 23.336533

N 41.199028 40.056404 28.346090

S 39.885277 40.520397 27.752529

S 41.872768 41.054794 29.254030

O 39.156288 41.407310 28.633091

O 39.099083 39.393417 27.303696

O 41.735870 42.410591 28.773901

O 43.251549 40.687611 29.495560

C 40.427010 41.463047 26.247122

C 40.945274 40.855312 30.855267

F 41.759338 41.462074 26.062796

F 39.875988 40.933540 25.135788

F 40.030491 42.748932 26.306280

F 40.358070 42.011627 31.229786

F 41.795033 40.514530 31.848770

F 39.990887 39.906303 30.812244

N 8.821239 33.679314 38.711365

S 9.742379 32.521534 38.425831

S 8.027220 33.551216 39.973286

O 10.324693 31.960733 39.611481

O 10.715835 32.899475 37.433796

O 7.577406 32.209110 40.237797

O 6.945711 34.513428 39.972309

C 8.602200 31.277702 37.645500

C 9.242207 34.063126 41.286549

F 8.633491 30.117964 38.329021

F 7.323653 31.695736 37.586830

F 8.992852 31.002926 36.391888

F 10.474304 34.350616 40.835266

F 9.355148 33.096512 42.218933

F 8.806571 35.166634 41.923622

N 5.061382 4.502049 12.666658

S 4.884391 4.532362 14.166200

S 5.494573 3.186344 12.091189

O 4.239495 3.337911 14.677618

O 4.191580 5.732681 14.565165

O 6.337974 2.440729 12.993751

O 6.086176 3.372137 10.783795

C 6.634264 4.652639 14.795450

C 3.887955 2.280849 11.866771

F 6.949623 3.616111 15.592891

F 7.571050 4.713755 13.821505

F 6.791698 5.769351 15.537642

F 2.804002 3.010330 12.192397

F 3.860087 1.152403 12.598207

F 3.729543 1.916598 10.578446

N 8.098603 46.594837 25.497105

S 8.644247 45.212639 25.251665

S 8.874043 47.475883 26.445625

O 9.761936 44.871368 26.114134

O 8.913157 45.027824 23.844040

O 9.171801 46.794319 27.692392

O 8.184655 48.724606 26.653908

C 7.198487 44.134064 25.684855

C 10.452791 47.846497 25.525953

F 6.140447 44.822964 26.149334

F 6.799033 43.431473 24.612637

F 7.545626 43.243237 26.635330

F 11.516699 47.435303 26.240767

F 10.597025 0.278689 25.325491

F 10.538843 47.249512 24.320257

N 21.412601 5.397276 4.572944

S 22.293161 4.750914 5.610449

S 22.009094 6.584394 3.865465

O 23.680738 4.761181 5.224195

O 21.792988 3.448409 5.982034

O 22.868139 7.366678 4.735756

O 20.964254 7.386235 3.247460

C 22.056030 5.858824 7.081633

C 23.014818 5.811398 2.505756

F 21.207253 6.879217 6.848415

F 21.582258 5.172369 8.135273

F 23.222124 6.426959 7.459574

F 24.304123 6.190317 2.593274

F 22.555227 6.222587 1.308860

F 22.979126 4.466357 2.489132

N 28.524549 45.586178 1.065375

S 28.187677 45.138321 48.557392

S 29.151352 46.948822 1.170982

O 27.718794 46.233521 47.736485

O 27.260181 44.036278 48.605801

O 30.036802 47.255871 0.069429

O 29.776203 47.052963 2.463219

C 29.806026 44.502716 47.902233

C 27.684080 48.091446 1.126493

F 30.832287 44.628368 48.768124

F 29.707264 43.195320 47.591274

F 30.138012 45.161301 46.780628

F 26.509188 47.449970 0.975902

F 27.791155 0.090452 0.120766

F 27.606340 48.804302 2.266671

N 35.857491 39.660370 33.886623

S 34.596546 39.433022 34.662548

S 37.128704 39.572365 34.693844

O 34.755447 38.503567 35.751392

O 33.511124 39.074146 33.770744

O 36.977623 40.113609 36.027145

O 38.221947 40.192814 33.981476

C 34.257717 41.134487 35.322605

C 37.421082 37.733997 34.763268

F 34.182423 41.127155 36.665688

F 35.184727 42.054165 34.968132

F 33.076466 41.595551 34.863987

F 36.470608 37.016247 34.130108

F 37.469975 37.297382 36.037903

F 38.593529 37.408371 34.186825

N 2.371301 5.071161 29.703352

S 1.014526 4.417465 29.373817

S 2.837249 6.057817 28.685406

O 0.127121 5.337627 28.717676

O 0.465459 3.818038 30.559956

O 2.687148 5.548355 27.340853

O 4.211137 6.380510 28.969961

C 1.456028 3.046021 28.195009

C 1.806589 7.587185 28.993429

F 0.837534 3.209812 27.014738

F 2.782978 2.982461 27.936855

F 1.109867 1.840325 28.680517

F 0.946565 7.473265 30.026047

F 1.119156 7.947036 27.899210

F 2.611853 8.628112 29.302433

N 32.792892 4.493737 6.252411

S 33.565884 4.923016 7.468864

S 32.863785 3.018152 5.935586

O 33.522369 3.950538 8.534238

O 33.160072 6.240188 7.899610

O 34.162376 2.452463 6.228454

O 32.442818 2.771244 4.579789

C 35.305000 5.074584 6.834724

C 31.577415 2.283294 7.052298

F 35.455101 4.755313 5.534176

F 35.738190 6.346286 6.973580

F 36.156223 4.301589 7.538781

F 32.133327 1.345041 7.833604

F 30.606892 1.684846 6.330151

F 30.961365 3.183899 7.847784

N 37.435749 33.989296 47.981926

S 38.310932 34.067528 46.767918

S 36.095112 34.627834 47.805912

O 37.566784 33.991253 45.531418

O 39.337681 33.052021 46.837837

O 35.187660 33.729187 47.137547

O 36.181160 35.940121 47.210888

C 39.124996 35.729881 46.959087

C 35.535286 34.793583 0.676185

F 38.890800 36.527809 45.902515

F 38.740700 36.391399 48.070911

F 40.464172 35.586624 47.054432

F 36.432472 34.336926 1.575326

F 34.395107 34.101749 0.877139

F 35.283001 36.079952 0.985187

N 39.052147 42.362186 7.383791

S 40.531639 42.053673 7.482554

S 38.508945 42.263424 6.005013

O 40.999054 41.133514 6.470962

O 40.879269 41.633198 8.816349

O 38.273285 40.894424 5.623161

O 39.287319 43.020775 5.051604

C 41.298771 43.724831 7.192131

C 36.861748 43.103405 6.192273

F 40.397675 44.707088 6.972113

F 42.044872 44.106682 8.248215

F 42.130436 43.709671 6.126268

F 35.853577 42.266357 5.881315

F 36.765427 44.156063 5.349361

F 36.599686 43.590866 7.431216

N 5.144500 41.905529 43.799637

S 4.918615 43.348358 44.028454

S 6.110133 41.597992 42.701504

O 4.114818 43.918934 42.962105

O 6.148270 44.066589 44.257763

O 6.118934 42.618385 41.674267

O 5.855890 40.292557 42.163685

C 3.935870 43.316086 45.599873

C 7.749509 41.568169 43.573753

F 2.724307 43.871021 45.419941

F 3.756923 42.080563 46.102978

F 4.562187 44.039700 46.548389

F 8.597799 42.462421 43.039841

F 8.340134 40.359051 43.478901

F 7.676659 41.825832 44.898746

N 27.026960 40.683212 10.238153

S 28.378845 41.256721 10.604360

S 27.064610 39.355770 9.549742

O 29.427109 40.254910 10.618538

O 28.292797 42.020428 11.824723

O 28.044420 39.339146 8.479478

O 25.767483 38.979782 9.055924

C 28.720608 42.396412 9.179623

C 27.514421 38.181366 10.913363

F 27.717819 42.422325 8.285863

F 28.925470 43.661270 9.594234

F 29.830965 42.015049 8.512725

F 28.668781 37.538914 10.652763

F 26.555145 37.247025 11.049284

F 27.640568 38.789101 12.107813

N 26.391356 27.203465 42.052696

S 26.778095 27.764755 40.690052

S 24.907459 27.142349 42.311340

O 25.792910 27.477753 39.666241

O 28.104071 27.340853 40.311626

O 24.206825 28.304041 41.808231

O 24.616058 26.826502 43.697449

C 26.786411 29.598722 40.972164

C 24.415598 25.657475 41.318813

F 26.517988 29.963953 42.237511

F 27.974995 30.121876 40.633827

F 25.869181 30.186415 40.168369

F 23.531616 25.961098 40.346825

F 23.817638 24.748556 42.118214

F 25.461905 25.037516 40.736992

N 4.020455 15.331315 1.523011

S 4.239984 14.068903 0.708946

S 5.137167 15.677965 2.471042

O 5.631473 13.790215 0.455681

O 3.451343 14.081615 48.390671

O 5.724858 14.517250 3.085136

O 4.707399 16.673912 3.444009

C 3.512948 12.761999 1.808545

C 6.365354 16.518921 1.361664

F 4.408175 11.794410 2.077944

F 3.050911 13.246526 2.973171

F 2.463219 12.152794 1.208630

F 5.966877 16.597639 0.078717

F 7.568605 15.920473 1.379266

F 6.548213 17.785734 1.795344

N 15.406610 21.957756 1.484874

S 16.290104 21.481052 2.608920

S 14.974398 23.397160 1.553813

O 17.213688 22.484819 3.079757

O 16.921799 20.233307 2.249069

O 14.730422 23.834263 2.910589

O 13.830795 23.599577 0.697700

C 15.059472 21.099199 3.951516

C 16.414780 24.314388 0.824333

F 13.783857 21.393045 3.619534

F 15.065827 19.780561 4.247317

F 15.346472 21.754852 5.093652

F 16.898329 25.221352 1.690224

F 16.033417 24.975908 48.605801

F 17.426373 23.501791 0.466926

N 16.453403 15.459903 38.150074

S 16.300859 16.191339 36.847569

S 16.575148 16.339973 39.361637

O 15.405143 17.315384 36.942913

O 15.959099 15.264333 35.790020

O 17.242535 17.584295 39.086861

O 17.237646 15.592891 40.408920

C 18.025309 16.798100 36.502388

C 14.814029 16.653866 39.866699

F 18.053177 18.140205 36.378201

F 18.933247 16.455849 37.435749

F 18.478544 16.326284 35.319180

F 13.915380 16.081820 39.042854

F 14.539741 17.970060 39.909237

F 14.587167 16.154671 41.099777

N 41.972511 32.043854 43.345913

S 41.268456 32.281960 42.016029

S 41.652756 30.753571 44.040188

O 41.051373 31.025414 41.306103

O 41.919220 33.284264 41.244007

O 40.222637 30.456791 43.967339

O 42.126526 30.736948 45.389629

C 39.608543 32.927834 42.546513

C 42.656521 29.493603 43.108292

F 39.525917 33.062290 43.886665

F 39.357723 34.132061 42.006248

F 38.599884 32.110348 42.193508

F 43.411915 30.007467 42.121147

F 41.876682 28.530905 42.600296

F 43.519970 28.879019 43.956097

N 25.494173 17.704082 1.808545

S 24.578899 17.358900 0.666409

S 24.922615 17.450329 3.191233

O 23.864574 16.119467 0.883982

O 25.302023 17.376013 48.307064

O 23.490057 17.633677 3.237192

O 25.634493 18.224792 4.176912

C 23.394228 18.794392 0.609204

C 25.311804 15.652541 3.483612

F 23.601044 19.738512 1.548435

F 23.498857 19.420710 48.303154

F 22.119593 18.379782 0.706501

F 24.190201 14.929905 3.690917

F 26.085285 15.493639 4.578811

F 25.976255 15.065827 2.463219

N 2.272048 36.983002 15.945409

S 0.792552 36.729736 16.114578

S 2.637278 38.376450 15.512707

O 0.110009 37.861607 16.706181

O 0.555422 35.504974 16.841125

O 1.593905 39.024277 14.756825

O 3.905557 38.387203 14.819407

C 0.221973 36.462780 14.360793

C 2.864140 39.259941 17.135948

F 48.153053 37.341385 14.020010

F 1.209608 36.560570 13.442587

F 48.577930 35.230686 14.210203

F 2.037852 40.321404 17.242535

F 4.122641 39.743492 17.234711

F 2.660257 38.486946 18.219902

N 23.075445 48.871777 31.852682

S 22.305874 0.405324 33.068645

S 23.332132 47.390812 31.728004

O 21.575415 48.205364 33.680294

O 21.506475 1.568484 32.785557

O 23.727186 46.769875 32.986504

O 24.245939 47.118969 30.652363

C 23.645046 0.965633 34.225937

C 21.676622 46.697021 31.254234

F 24.891325 0.784729 33.754608

F 23.521837 2.292095 34.489471

F 23.545305 0.333447 35.406212

F 20.679209 47.599586 31.181871

F 21.329483 45.771484 32.173908

F 21.724049 46.061905 30.068583

N 35.568535 6.520833 25.854023

S 34.279232 7.197509 26.205563

S 36.712135 6.800988 26.787386

O 34.056278 7.284538 27.628342

O 33.178654 6.594172 25.496128

O 36.723873 8.172432 27.242579

O 37.964283 6.408380 26.186495

C 34.514893 8.907290 25.519596

C 36.387486 5.662764 28.219948

F 35.714233 9.103839 24.940218

F 33.592777 9.170823 24.576454

F 34.346214 9.873412 26.455894

F 36.299480 6.358509 29.368437

F 37.405926 4.791984 28.363691

F 35.263443 4.929372 28.101137

N 1.818812 47.832802 21.167648

S 2.056920 0.233710 20.408833

S 1.062930 46.778675 20.404921

O 2.309207 0.012222 19.003164

O 3.095403 0.998389 21.059107

O 48.890842 47.334587 19.607967

O 0.584758 45.753391 21.313349

C 0.451769 1.153869 20.610760

C 2.358100 46.021816 19.303366

F 48.433697 0.497729 21.363708

F 0.665431 2.339521 21.218496

F 48.771545 1.423267 19.422665

F 3.576508 46.587502 19.428043

F 1.997271 46.098087 18.011129

F 2.517490 44.715401 19.594769

N 34.465023 32.698528 10.906516

S 33.211414 31.871260 10.883537

S 35.594936 32.206665 10.044047

O 33.344402 30.650408 10.126188

O 32.088833 32.681412 10.469904

O 35.973854 30.844509 10.371140

O 36.724846 33.101894 10.117876

C 33.006062 31.442471 12.677415

C 34.915325 32.313251 8.311776

F 34.006897 31.899130 13.458720

F 31.856104 31.936775 13.180032

F 32.963524 30.102318 12.843650

F 34.933414 31.100710 7.727507

F 35.674633 33.129272 7.552471

F 33.646069 32.772354 8.234526

N 43.851952 48.553482 11.402779

S 43.978584 47.500332 10.346206

S 43.206078 48.066509 12.680836

O 42.784622 46.707779 10.192682

O 45.190636 46.728317 10.502662

O 43.480366 0.092405 13.762346

O 43.567883 46.705334 13.003039

C 44.194691 48.556419 8.833951

C 41.388245 48.190697 12.296050

F 44.182957 0.993015 9.077927

F 45.375450 48.283104 8.234036

F 43.232479 48.316841 7.920633

F 41.129601 48.654202 11.052217

F 40.790775 0.162323 13.144829

F 40.763393 47.007496 12.434417

N 47.306717 36.562035 34.712421

S 47.196220 35.965054 33.348801

S 46.749340 35.704456 35.847221

O 47.600071 34.578941 33.313110

O 48.022995 36.715069 32.422771

O 45.663921 34.857143 35.419899

O 46.449139 36.530746 36.990337

C 45.405766 36.164051 32.887253

C 48.221500 34.654728 36.273571

F 44.652817 36.738049 33.842129

F 45.311401 36.943398 31.795475

F 44.863544 34.974976 32.568962

F 47.966282 33.340000 36.156715

F 48.641491 34.881100 37.534023

F 0.379410 34.909946 35.463905

N 25.264378 42.577805 16.265167

S 24.715311 41.827789 17.446907

S 24.452755 43.784477 15.871581

O 23.581486 41.018127 17.076300

O 24.454222 42.692215 18.574863

O 23.026552 43.586464 16.011414

O 24.829231 44.191269 14.542674

C 26.139069 40.705700 17.852716

C 24.985199 45.123165 17.049898

F 25.789484 39.413952 17.727062

F 27.217646 40.914963 17.072876

F 26.530210 40.878292 19.132242

F 25.933720 44.739357 17.921167

F 23.943781 45.604267 17.768131

F 25.471682 46.180717 16.368332

N 12.084833 12.114657 41.509987

S 11.670222 12.672036 42.832047

S 11.846236 10.645430 41.359398

O 12.662257 12.405081 43.853909

O 10.326648 12.297517 43.192875

O 11.860904 9.925238 42.607632

O 12.770800 10.113476 40.385941

C 11.740139 14.486937 42.513268

C 10.152590 10.549110 40.610359

F 12.704794 15.018889 43.290665

F 12.020783 14.794473 41.235207

F 10.584803 15.085874 42.843784

F 9.531162 11.739650 40.470528

F 9.318479 9.776604 41.338863

F 10.200994 9.995155 39.380219

N 42.722527 3.560374 35.629162

S 44.007431 3.514903 34.846390

S 41.469406 3.358447 34.806293

O 43.991787 2.528736 33.793724

O 45.126587 3.358447 35.749928

O 41.559856 3.977918 33.503792

O 40.305759 3.771591 35.554844

C 44.122818 5.214906 34.098816

C 41.377975 1.514699 34.612190

F 43.085312 6.023104 34.392174

F 45.232197 5.841223 34.553028

F 44.225983 5.156235 32.757687

F 42.375389 0.853668 35.233128

F 41.395580 1.170005 33.312618

F 40.228508 1.043372 35.136810

N 27.030384 32.295158 38.354935

S 27.642033 33.552197 38.984673

S 27.453796 32.035538 36.932156

O 28.965561 33.835773 38.488411

O 27.569183 33.468098 40.419678

O 27.494865 33.242214 36.147423

O 26.555145 31.029816 36.408512

C 26.463717 34.888435 38.449299

C 29.145487 31.274769 37.093014

F 27.072432 35.859447 37.755508

F 25.453592 34.421021 37.685593

F 25.886293 35.476128 39.520050

F 29.553741 31.162313 38.371067

F 30.076405 31.970024 36.422199

F 29.133265 30.021156 36.591370

N 8.684829 33.393784 9.129264

S 8.601710 32.903385 10.537865

S 10.080718 33.767323 8.663804

O 9.693486 32.016472 10.851756

O 7.297739 32.326942 10.814110

O 10.949542 34.186825 9.750202

O 10.007378 34.719265 7.585718

C 8.691673 34.475780 11.536256

C 10.757882 32.170486 8.000328

F 9.728201 34.510983 12.386991

F 8.755722 35.593956 10.786241

F 7.573006 34.562809 12.289205

F 11.885839 31.852682 8.670650

F 11.089865 32.274136 6.700758

F 9.912526 31.128088 8.114738

N 26.143469 46.489719 5.889627

S 25.741570 46.373352 4.450712

S 26.993225 45.336338 6.368776

O 26.748272 45.758282 3.624912

O 25.311804 47.672924 3.951516

O 26.704760 44.110596 5.706279

O 26.931131 45.248817 7.822848

C 24.204868 45.324604 4.562676

C 28.702518 45.910828 5.894027

F 24.333456 44.190777 3.845907

F 23.876308 44.978931 5.824111

F 23.127272 45.975368 4.063480

F 28.700563 47.113590 5.287756

F 29.256962 45.041023 5.017868

F 29.523428 45.992481 6.951089

N 33.929649 10.963721 35.153923

S 34.208336 10.344738 36.502388

S 33.679314 9.968753 34.046013

O 33.392315 9.176690 36.729248

O 34.079258 11.330906 37.552116

O 34.519295 8.804127 34.153088

O 33.750698 10.603870 32.751820

C 35.996346 9.836253 36.434425

C 31.906464 9.458312 34.311501

F 36.121510 8.507835 36.642220

F 36.602127 10.122276 35.268822

F 36.701870 10.437634 37.418636

F 31.302637 10.093919 35.333847

F 31.804766 8.128917 34.538853

F 31.156937 9.707176 33.216301

N 32.346500 7.922100 22.230577

S 31.388199 6.799033 21.932821

S 31.808678 9.310656 22.006649

O 30.006489 7.186264 22.167017

O 31.750984 5.577201 22.603630

O 30.795128 9.358571 20.969145

O 32.870628 10.264066 21.803743

C 31.658087 6.550169 20.109119

C 30.972122 9.694465 23.624512

F 30.518394 6.799033 19.430975

F 32.607098 7.352010 19.585966

F 32.014027 5.280423 19.826031

F 31.001945 8.691673 24.519249

F 29.668152 9.950663 23.377604

F 31.487942 10.788197 24.220516

N 37.724705 24.771536 31.917221

S 37.908054 24.499203 33.382538

S 38.536816 23.919334 30.983368

O 38.077713 23.094513 33.679806

O 36.854904 25.128942 34.153088

O 39.836876 23.580507 31.521187

O 38.621887 24.531961 29.678907

C 39.476048 25.400785 33.788834

C 37.527180 22.365034 30.822510

F 40.040268 26.006569 32.727371

F 39.236473 26.354687 34.710464

F 40.381538 24.556898 34.315899

F 38.239059 21.282547 31.214140

F 37.181019 22.148439 29.536629

F 36.390423 22.368944 31.540255

N 30.567778 40.152721 32.700481

O 31.358376 40.365406 31.749029

O 29.855898 41.076305 33.158607

O 30.566311 39.011566 33.222656

N 22.044785 35.123608 45.124142

O 22.629543 36.228588 45.257130

O 21.144669 34.999908 44.257271

O 22.249645 34.194645 45.946030

N 20.417143 25.931274 25.542086

O 19.678862 25.294691 26.331219

O 21.368109 25.346518 24.961241

O 20.219616 27.154572 25.349939

N 11.728404 8.223769 18.535749

O 12.379169 9.084771 17.899654

O 10.491417 8.088824 18.368536

O 12.304851 7.715284 19.526806

N 37.454327 31.245920 25.666763

O 37.087631 31.648798 26.796677

O 37.697327 30.021156 25.550400

O 37.592205 32.025272 24.694775

N 16.613283 20.174147 23.889511

O 17.742708 19.763449 23.524771

O 16.394245 20.191259 25.125523

O 15.746414 20.536442 23.063223

N 5.714591 3.199056 29.415373

O 5.277489 3.774035 28.386671

O 6.015770 1.985537 29.383104

O 5.721436 3.827328 30.503727

N 21.175470 4.268830 24.277719

O 20.768684 3.431297 23.431385

O 22.327873 4.753847 24.181890

O 20.403944 4.663395 25.186636

N 27.219110 37.766754 14.751447

O 27.730042 36.775700 14.175979

O 26.000214 37.783867 15.036002

O 28.008240 38.610641 15.241352

N 39.868164 0.652230 1.709781

O 39.500004 48.464500 1.200807

O 39.010098 1.557724 1.845214

O 41.049908 0.742682 2.110213

N 32.823692 32.545494 5.175792

O 32.374367 33.350269 6.029949

O 34.055790 32.496601 4.949907

O 32.037498 31.764185 4.590056

N 40.188904 9.137087 30.969677

O 39.682865 9.006054 29.828520

O 40.052494 10.191704 31.626797

O 41.007858 8.255549 31.320238

N 37.584385 48.192654 33.548286

O 37.340897 46.963490 33.664646

O 38.059132 48.639534 32.480465

O 37.409348 0.053293 34.534451

N 16.129246 39.480934 21.684444

O 17.284582 39.496094 22.174841

O 15.250154 40.268108 22.122524

O 15.843223 38.667850 20.772104

N 29.239359 40.217262 5.367940

O 28.718163 39.085880 5.256465

O 28.569530 41.071907 5.979589

O 30.486616 40.361008 5.248642

N 24.241049 22.507801 45.844334

O 24.152065 23.149273 44.766247

O 24.490402 21.271791 45.846291

O 24.064547 23.136072 46.910198

N 3.210301 3.866443 18.573397

O 2.065721 3.465522 18.253149

O 4.067881 3.914846 17.657635

O 3.454765 4.312834 19.719933

N 29.323946 6.465095 46.798233

O 29.711178 7.272315 45.927452

O 29.795271 6.472428 47.962372

O 28.578817 5.500440 46.506340

N 18.007708 28.621355 48.626823

O 16.963846 28.356358 0.379897

O 18.982141 27.830759 48.646381

O 18.091803 29.703842 47.995125

N 20.329136 23.184477 18.001350

O 21.320683 23.950138 17.976906

O 20.423012 22.159683 18.714697

O 19.245184 23.506680 17.456196

N 19.123440 20.735926 34.722687

O 18.022375 20.142855 34.706554

O 19.195803 21.977314 34.880611

O 20.153612 20.064627 34.467957

N 5.933141 8.628601 6.748673

O 5.620227 7.655146 6.020170

O 5.092185 9.184512 7.490866

O 7.134437 8.991385 6.716893

N 18.081535 17.924101 31.660042

O 17.387747 16.890995 31.493809

O 18.648691 18.136786 32.758175

O 18.021397 18.838884 30.800997

N 16.749207 42.901966 35.491283

O 16.311617 43.337112 34.396084

O 17.943169 43.115627 35.818378

O 15.949320 42.395927 36.318062

N 28.698605 44.151665 16.067640

O 28.096247 43.348358 15.313225

O 28.331909 45.354916 16.127779

O 29.628548 43.739986 16.802011

H 28.247816 -0.037647 18.610556

H 29.060413 -0.527061 20.124765

C 49.316700 1.770408 39.524937

O -0.194592 0.541732 39.839809

H -0.316338 2.572739 39.355770

H 47.294495 -0.366207 40.971188

C 45.573467 48.974941 43.338085

H 45.395008 -0.597958 42.485889

O 46.570393 -0.412655 44.206425

H 15.572845 -0.449326 45.324116

H 15.904828 -0.472794 47.074474

H 41.868370 28.904446 49.340168

H 41.160404 30.542843 49.288342

H 43.308754 -0.016624 41.426868

H 48.977383 6.610796 36.674000

H 49.146553 4.169578 36.306324

C -0.843399 46.387043 38.631668

O 49.216473 45.927452 37.954502

H -0.273800 43.994232 37.382458

H 48.912842 5.681343 32.665279

H 12.659325 49.673130 38.686916

H 12.318541 49.401283 41.201473

C 49.507381 27.487532 34.401951

O -0.195572 28.362223 33.639225

H 45.514797 -0.158413 29.547386

H 45.300156 -0.352028 31.303127

C 48.951469 0.630717 31.570080

O -1.114269 0.056716 31.035194

H -0.112453 1.054129 32.574825

H 0.811131 -0.164280 31.644398

C -0.371586 26.367399 9.232428

C 49.445778 27.411747 8.665760

H 48.977383 25.942520 10.138900

C 23.342888 -0.566669 43.938004

C 22.719505 49.688286 44.076370

C 13.921247 19.756603 -0.484528

C 13.767724 19.287720 49.838875

O 33.320442 49.542099 20.095428

C 32.386589 -0.409233 20.198593

C 28.329464 15.695077 49.183224

O 28.077179 14.704998 -0.696720

C 29.264297 16.732094 -0.279667

O 29.993288 17.480154 49.580723

H 27.173639 13.404940 49.572899

C -0.241528 6.177605 25.289801

H 49.372925 5.528798 25.817842

O 49.223804 7.432194 24.966131

H 36.713600 -0.684990 23.180565

H 39.490227 21.975357 49.737175

H 40.409409 20.631783 48.993031

C 26.548302 21.473719 49.320122

H 25.701477 21.739204 -0.228329

O 27.775999 21.392067 -0.270378

H 26.288679 -0.021511 16.552658

H 26.899841 -0.914295 15.135745

C 21.160803 -0.356430 11.062485

O 21.897129 49.547962 10.394608

H 20.477772 48.979832 11.808589

C 26.931131 49.347504 31.493809

O 27.545715 -0.753437 31.086531

O 1.686802 49.934216 25.203259

C 1.892640 -0.321712 24.883501

C 8.679450 49.523518 31.897173

O 8.588998 -0.599426 31.202894

H 9.431910 -0.430260 33.571262

H 7.869296 49.136284 29.422709

C 36.969311 -0.595028 29.525383

O 36.134712 49.427197 29.335680

C 47.958946 -0.012222 26.824547

O 46.634441 49.099617 27.256269

H 48.514370 49.825672 26.704760

C 49.274651 18.794392 33.109226

H -0.357407 18.052198 33.457832

O -0.195572 20.089073 33.077934

H 49.018456 20.435724 35.125076

H -0.160366 37.140926 41.132046

H -0.701611 34.963730 40.290112

C -0.121742 37.563847 0.408255

H 47.830360 37.861118 -0.080185

H 49.483421 37.343342 -0.404346

O 49.198872 38.701584 1.153381

H 49.240429 36.214409 2.016828

O 22.394369 49.017475 26.113153

H 23.972630 49.582184 27.342808

C 23.214300 -0.108055 27.247469

C -0.020046 6.243122 -0.064537

C -0.208282 8.505391 -0.673744

O 49.303009 7.569583 49.104507

C 49.619835 9.617214 47.823513

C -0.208282 8.505391 48.219055

H 47.779510 6.128223 48.927513

H 49.158287 5.923851 47.810310

H 49.363148 5.572312 49.548450

H 12.448106 0.069917 49.091793

C 13.640602 1.502965 50.113163

C 13.208390 0.834111 -0.050362

C 42.745995 34.620502 49.427197

C 42.827160 33.598152 -0.563248

H 30.164412 49.345058 15.875981

H 49.510315 45.618450 24.199492

H 10.802375 16.702271 49.649174

C 14.674685 36.332729 50.214371

O 15.086851 36.411934 -0.031292

C -0.210239 7.992506 18.475122

H -0.390163 5.595781 17.465485

H -0.044003 6.138980 15.083428

O 49.037033 7.595496 17.167728

H 49.473156 7.758799 19.206558

H 22.064342 -0.338337 4.500093

H 22.615852 -0.223442 2.837249

C 6.273435 48.897202 21.111912

C 5.972255 -0.108540 19.646105

H 5.420256 -0.335892 21.725025

O 7.433661 -0.750015 21.422379

O 6.484652 49.929325 18.979206

H 8.069757 49.310345 23.039753

H 42.137772 40.771706 49.631569

C 3.344268 3.624912 -0.201927

C 2.470064 2.706705 49.498093

O 4.617925 3.695807 49.301056

H -0.797440 2.409437 0.582802

C 5.439324 4.663395 -0.212193

H 6.412780 4.634548 49.188110

H 27.254801 -0.129566 8.390493

O 28.492279 -0.152546 10.061649

C 28.093315 49.326477 8.832973

C 43.291149 46.388512 -0.391144

O 43.162075 47.034874 49.757225

H 39.085392 49.359726 16.678312

H 40.406479 49.058056 15.550355

O 40.853355 49.043388 17.599941

C 39.980129 -0.151081 16.518921

C 42.155373 -0.354961 17.364767

H 42.585629 48.916756 16.421625

H 41.454247 16.237299 -0.119785

H 40.271046 14.072814 -0.386742

H 40.022179 18.191545 -0.474258

H 14.905949 49.019920 9.495470

H 15.270199 -0.227352 11.598839

H 15.737615 -0.748547 13.234303

O -0.384296 13.515925 16.529676

C 49.709797 13.105714 17.157949

C 6.179072 21.920597 -0.073830

H 6.017237 20.999458 49.402752

C 6.441627 23.062244 49.766514

H 3.650826 21.715736 49.162689

C 10.246464 42.905388 -0.018093

H 9.684197 43.034466 49.818829

O 11.625241 43.216835 49.038502

H 10.012267 40.725258 48.961250

C -0.407764 23.723276 36.905750

C -0.382343 23.534060 39.300522

O 49.019428 24.170645 38.135895

H 49.246296 23.751635 40.088673

H 48.938267 24.348614 36.123466

C 49.463379 23.327732 43.016373

H 49.107437 24.957329 44.328655

C -0.340782 24.394573 43.555172

C 34.709976 -0.140324 43.642693

O 34.607300 49.888744 42.802711

H 34.670376 49.039478 44.709045

C 49.386616 5.130322 9.999556

O -0.593071 6.032393 10.024001

H 38.900089 46.521008 48.958805

H 38.596954 44.076370 49.601257

C 39.646683 49.110371 46.015457

H 39.316654 -0.167702 45.035160

O 39.164112 -0.572044 47.083767

H 48.979340 11.940111 1.442338

C -0.593559 14.307989 10.665476

C -0.013691 14.624815 12.911610

O 49.123081 15.020846 11.575371

H 49.780201 14.832119 9.251985

H 48.980804 13.534993 13.054377

H 49.633522 15.116675 13.538905

H 49.468758 16.748716 7.686926

C 22.559626 36.983978 49.657482

O 22.067276 37.422058 -0.488438

H -0.445900 33.698387 25.317669

C -0.238598 34.266518 23.258305

C 49.176376 33.597668 24.493336

H 49.437466 34.192688 22.481888

C 20.232819 48.951962 36.606525

C 19.094595 -0.321224 35.702988

H 21.181339 -0.162323 36.089729

O 20.129654 -0.667877 37.821022

O 19.151310 49.364124 34.532986

H 21.474207 49.437466 38.885910

C 18.310843 -0.032761 33.514057

H 18.443830 49.509827 32.638390

O -1.302994 37.260223 20.376562

C 48.909912 37.320362 19.860744

C 36.533676 8.021841 49.033611

O 37.346764 8.074157 -1.013058

C -0.020046 47.988770 14.009254

H 1.304460 49.610546 14.414086

C 50.255932 48.523170 14.230248

H 48.928982 46.889660 13.898755

H 3.107626 49.361195 16.109200

H 39.966930 5.097075 49.383194

H 38.672249 6.311082 49.176376

H -0.114895 26.619194 26.463717

C 23.301331 43.715542 -0.789619

O 23.164431 44.593655 49.220383

H 22.127903 42.251202 49.112328

H 23.827417 41.774006 48.896709

C 6.225031 48.943645 9.789805

H 5.289712 -0.481106 10.028402

O 7.320719 -0.829708 9.666595

C 1.767475 47.462685 -0.948521

O 1.774809 46.538120 49.020409

C 19.536585 15.311269 -0.411678

O 19.147398 14.717222 49.702465

O 16.295969 -0.569115 21.209698

C 16.437269 49.634502 20.676764

C 11.165648 24.544674 -0.925053

O 11.068841 24.882524 49.338703

C 50.044224 1.149959 8.492680

O -0.071384 1.781165 8.825639

H 47.765820 -0.013203 9.089171

C 35.612537 40.853844 -0.005867

O 34.212246 40.818153 49.076637

H 36.059906 39.843719 48.942669

C 36.171383 41.679157 50.007557

C 33.548286 39.902878 -0.660542

H 26.363487 -0.260109 46.883305

H 30.730593 49.389549 45.776863

H 45.197483 28.835506 -0.036179

H 47.050030 26.899841 -0.633652

C -0.198502 30.985323 28.479568

C 49.586586 30.914919 27.273382

H 49.147533 30.437723 29.323946

H 49.189579 23.255861 26.165470

O -0.715790 21.178894 6.939355

C 49.486359 21.717205 6.908552

H 25.072716 49.074192 37.756973

C 26.876860 49.654064 36.786942

C 25.981634 -0.305092 37.361919

H 49.427197 39.781136 30.297890

H 8.604644 20.613205 -0.451767

O 9.187935 18.642824 -0.611649

C 8.597799 19.685707 49.035076

H 43.950226 7.603319 49.267811

C 8.680917 -0.487949 16.199162

O 7.924057 -0.446880 15.013512

H 9.494982 -0.191170 13.698296

C 8.778703 49.309364 14.279631

O 9.074992 49.702953 16.575148

H 32.465305 49.343102 22.874006

H 33.560509 49.431599 24.237139

H 30.567778 49.151443 24.255718

H 31.578880 49.524006 25.649651

H 20.538887 20.673342 49.504444

C 48.359379 16.191828 49.267811

C 49.369503 14.731400 48.087044

C -0.859535 15.052627 47.484688

O 0.780328 15.793352 48.977383

C -0.533421 16.191828 0.375010

C 0.476705 14.731400 -0.805756

O 47.607899 16.271524 -0.812599

O 49.673126 15.793352 0.084583

C 32.757687 25.543554 -0.568623

O 32.546467 25.384653 49.721535

C 31.138845 25.357273 49.726913

O 30.678766 24.540762 -0.212193

H -0.033249 33.826973 8.798748

H -0.370121 32.860851 10.232285

H 45.589115 41.152580 -0.344692

H 47.081810 40.267132 -0.657608

H -0.735836 29.000275 12.880320

H -0.597958 26.661243 12.502866

H 38.860485 9.851899 49.100105

H 39.049702 11.517676 49.680458

C 49.100105 41.305126 21.349041

O -1.139202 41.624886 21.028305

C -0.894249 42.179817 19.760025

O 49.030190 43.126873 19.887146

H 23.818617 -0.289936 24.093884

H -0.207306 46.549858 41.723160

H 33.573219 8.794348 -0.185307

H 45.418476 49.218426 33.762436

H 46.302948 49.361683 35.296200

C 34.413685 -0.621426 0.805752

C 35.934254 -0.393589 -0.667385

O 35.008713 49.301056 0.022002

C 35.934254 48.499210 -0.667385

O 35.008713 49.301056 48.914803

O 36.615326 47.713017 49.173931

H 36.650043 49.143131 47.693459

C 6.193740 49.693176 48.423428

H 7.330497 49.255096 46.628574

O 5.216862 -0.136898 48.855152

H 6.579993 1.342107 49.297146

C 7.212666 -0.003910 47.662655

H 49.041435 39.787003 36.714581

C 49.953285 38.671268 38.400894

C -0.073341 39.375816 37.707592

C -0.155479 39.778202 39.767937

O 50.034447 39.238426 39.702908

C 30.945719 30.705656 -0.181881

C 30.614227 29.524406 49.577297

O 31.715780 31.584749 49.514225

C 9.066192 -0.151566 37.868450

H 8.346978 49.020409 37.074921

O 9.608902 49.908302 38.452229

O 8.273150 48.946091 40.084274

C 8.419340 -0.878605 39.008141

H 22.498022 49.039478 6.876284

C 24.528051 49.308388 7.677636

C 23.426006 -0.408257 7.070388

O 24.676195 -0.067474 9.002142

C -0.007336 45.476658 34.832699

H 49.604187 44.646461 34.951996

C 49.546497 46.804588 35.046848

C 49.414974 46.285347 32.942501

O -0.491863 45.491817 33.503300

H 35.702988 49.268784 5.877892

H -0.025913 21.201874 11.614974

H -0.016624 22.201242 13.065133

H -0.458614 23.235813 10.322248

H -0.400921 24.141798 11.829613

H 46.339619 21.037104 49.469246

H 43.945339 20.439146 49.637924

H -0.038624 16.719870 42.484909

C 49.006229 40.337048 33.107761

H 49.017475 41.831699 31.484028

H -0.691345 39.603657 33.296974

C -0.388699 41.580395 32.422771

C -0.158413 41.899174 34.519783

O 49.546986 40.765839 34.348171

H 49.140198 42.509842 35.337761

H 9.517472 -0.182369 19.131752

C 10.740770 -0.666897 20.866959

H 11.238499 -0.250820 18.761633

C 10.522220 48.913822 19.547829

O 10.759350 49.247761 21.856548

H 21.396467 26.929176 49.585613

C -0.074314 40.365406 12.299961

O 49.359726 39.056057 12.150839

C 49.528404 41.186317 11.290325

H 49.019432 40.726723 13.322310

H 21.793478 32.664299 -0.740726

H 19.510183 31.415092 -0.353985

C 44.478268 15.987945 -0.600891

C 44.603436 16.933043 49.457024

C 45.059113 14.950439 50.068672

O 45.117786 14.777848 -0.217575

C 49.091305 22.030117 48.618511

C 49.075657 23.983873 47.815201

O -0.520706 23.226524 48.787682

H -0.464481 24.802340 47.469040

H -0.309002 21.218987 49.157307

H 1.215475 22.113235 49.060013

H 15.455503 44.257271 -0.320740

H -0.022003 1.749873 44.971107

H -0.182861 2.380590 43.320976

H -0.250332 39.650105 46.178272

H -0.974922 41.040615 44.435242

H -0.641472 41.939266 47.060299

H -0.154503 31.292860 33.076958

H 18.754299 -0.691345 40.306244

H 17.954903 -0.288467 41.838058

C -0.318295 38.571041 4.309412

C -0.555912 37.106701 5.838289

C 49.458488 36.739029 4.905415

O 49.830566 37.941792 4.251718

C 49.455555 27.999439 46.258457

O -0.325134 27.082699 45.666851

O -0.146191 28.837952 47.137547

C 48.897202 14.273764 37.052433

C 49.231136 15.042359 35.098183

O -0.429279 14.032723 35.735748

H -0.169659 15.323981 34.164822

O -0.030804 15.651564 37.336987

H -0.073830 17.068476 35.861404

C 49.375370 16.151737 36.106842

H -0.659565 13.748167 37.753551

C -0.144726 10.468437 35.270779

O 50.037380 9.882702 35.098183

C 49.701485 8.770879 34.300743

O -0.437099 8.247726 34.684551

C -0.162323 29.666197 38.011707

C 49.774826 28.690294 38.481567

O 49.111351 30.045115 36.698448

H 31.953890 12.717505 -0.054760

C 42.848183 -0.305092 22.297562

C 41.245964 -0.682053 20.974522

H 40.162014 -0.544174 22.871073

C 40.747749 48.969070 22.174353

O 41.897217 49.462399 22.853474

H 43.856842 48.982761 22.484333

C -1.027237 15.070229 23.377115

O 49.251183 15.236464 23.616199

C 49.411064 16.451448 22.934145

O -0.473282 17.355965 23.351202

H 33.303818 -0.404831 34.281185

H 35.035114 -0.604805 34.130108

C 50.030045 30.610804 42.675102

O -0.006355 29.811407 42.911743

O -0.407276 31.811611 43.962452

C 49.818829 31.867350 43.474499

C 23.557039 -0.894249 -0.045471

C 21.934776 0.452258 -0.249355

H 23.444586 2.020739 -0.356918

H 21.287926 0.058671 49.450665

O 22.521490 -0.622894 47.930588

C 23.091581 1.216453 49.194958

C 21.934776 49.345058 48.643444

H 23.162964 47.414280 49.701977

O 24.162821 49.178822 49.326477

C 49.183220 44.112061 9.429955

O 49.012585 45.195038 8.545484

C -1.064884 45.703033 9.147353

O -0.914783 43.397247 9.496449

H 48.956848 43.096558 41.713383

C 17.193153 3.391694 -0.371586

O 18.525482 3.231325 -0.793530

C 19.120508 3.149674 49.388084

O 16.892952 2.431439 49.502491

H 17.012739 4.421376 48.900623

H 49.663345 44.212780 30.019690

H 4.322124 -0.390652 3.727587

H 20.053871 49.675087 22.611452

H 49.475601 38.809639 24.345680

H 48.896709 38.968540 26.016836

O 17.364767 49.016006 17.213198

C 16.292059 -0.798420 17.079721

C 18.325998 -0.772995 17.709949

C 25.401766 32.570427 49.021389

H 25.302023 34.663040 49.626190

O 26.534122 32.105457 -0.589157

H 24.535385 31.961712 -0.169659

C 25.287846 34.040634 -0.177971

H 27.719774 33.505745 49.272694

C 22.281427 -0.193615 17.051363

H 21.537277 -0.816513 14.596946

H 23.862131 -0.829224 14.955818

O 21.281569 49.278072 16.248543

H 22.098568 48.962227 18.102558

O 23.542372 49.183220 16.661688

O 31.226366 48.920670 36.362064

C 30.503727 -0.197529 35.175926

C 31.279657 -1.317661 36.812366

H 30.144367 49.656994 34.783806

C -0.824821 25.292736 31.512877

C -1.347485 24.225893 33.279373

C 48.910892 23.820084 32.787510

O 48.975430 24.220516 31.424870

H 5.706768 43.377201 -0.126633

H 6.021148 45.033691 -0.624359

H -0.232731 19.789360 38.110958

H 30.887535 1.529367 -0.499195

O 29.661797 35.095737 49.075172

H 31.339796 34.115440 49.740597

C 30.943274 34.548141 -0.082142

C 29.662283 36.053059 -0.846821

H 37.563362 23.839640 48.903553

O 2.952636 17.595541 48.955872

C 3.203456 17.345699 -1.313259

C 2.785912 18.984097 -0.030315

H 2.355655 19.370838 49.795849

H -0.046936 31.341751 22.423704

C 23.625490 8.994808 49.314255

C 24.533916 10.920207 49.533295

O 23.805414 10.173124 -0.323181

H 22.848583 8.386581 -0.061604

H 48.904533 47.661678 5.461814

O 49.125530 16.209919 28.531391

O 49.596855 16.995138 31.478161

O 36.615326 -1.179783 0.281131

O 39.500004 -0.428299 1.200807

O 26.378645 -0.841934 20.796553

O 27.550602 -1.102531 23.477345

O 17.491888 49.013565 45.948963

O -1.371933 7.309962 2.886142

O 49.477558 45.753391 21.313349

O 27.997972 5.366474 49.991421

O 5.216862 48.755901 -0.037647

O 5.393854 49.815407 2.539003

F 49.145084 27.709503 20.641562

O -1.070751 43.270126 5.417811

O -1.596840 19.760513 23.033398

O 13.824440 -0.333450 40.009468

N 39.868164 49.545029 1.709781

O 41.049908 49.635483 2.110213

O 8.566997 -0.808685 43.082378

O -1.292728 34.578941 33.313110

O -0.869804 36.715069 32.422771

O 10.728547 20.362383 49.830563

O 12.611898 18.467789 49.997288

F -0.328072 32.883831 4.146109

C 49.589523 32.839336 5.027647

O 6.898774 48.939735 5.227618

O 3.410273 27.736887 -0.649296

N 5.213439 26.071108 -0.801842

F 42.702484 2.449040 49.176868

C 42.221378 2.086745 -0.919674

O -0.590137 16.554613 26.296503

F -0.109520 13.450409 28.035131

C 50.036404 13.424007 28.526014

O -0.183350 24.412174 19.201181

N 49.491734 25.120142 21.412601

F -0.524132 27.130613 18.724476

C 49.437466 26.976603 19.541473

O 5.993768 35.895138 48.965160

O 7.338809 34.181442 50.004623

F 9.661706 35.558266 48.937294

F 5.465726 32.230625 49.223804

F 7.285516 31.196539 49.787540

C 8.567485 35.821796 -0.684498

C 6.567770 31.627287 -0.160858

O 15.887226 6.953534 48.971519

F 18.283463 8.410050 49.232117

C 18.631578 7.607231 -0.692322

F 10.597025 49.171490 25.325491

C 10.452791 -1.046303 25.525953

N 28.524549 45.586178 49.958176

F 27.791155 48.983250 0.120766

C 27.684080 -0.801353 1.126493

O 3.451343 14.081615 -0.502129

F 16.033417 24.975908 -0.286999

C 16.414780 24.314388 49.717133

O 25.302023 17.376013 -0.585735

F 23.498857 19.420710 -0.589645

C 23.394228 18.794392 49.502003

F -0.314869 35.230686 14.210203

F -0.739746 37.341385 14.020010

C 49.114773 36.462780 14.360793

O 21.575415 -0.687435 33.680294

N 23.075445 -0.021023 31.852682

N 1.818812 -1.059998 21.167648

O -0.001957 47.334587 19.607967

F -0.121254 1.423267 19.422665

F -0.459103 0.497729 21.363708

C 49.344570 1.153869 20.610760

O 43.480366 48.985203 13.762346

F 44.182957 49.885815 9.077927

F 40.790775 49.055122 13.144829

C 44.194691 -0.336380 8.833951

C 41.388245 -0.702103 12.296050

F 49.272209 34.909946 35.463905

C -0.671299 34.654728 36.273571

O 37.409348 48.946091 34.534451

O 16.963846 28.356358 49.272697

C 50.092628 1.554791 38.259594

H 49.994843 2.108258 40.329227

C -1.090797 0.608715 40.927185

H 44.616634 49.142155 43.861240

H 45.930382 49.939594 42.951836

C 46.143066 -1.620308 44.812695

C -2.181595 46.200760 37.960857

H -0.921143 45.950432 39.642773

H -0.697212 47.466599 38.768078

C 49.358261 44.519337 38.010731

C 50.913052 27.553047 33.859730

H 49.152420 26.449049 34.312477

H 49.453598 27.732973 35.476616

C -1.503941 28.428228 34.170689

H 49.703930 -0.164280 31.644398

H 49.361195 1.415447 30.919319

C -2.094566 1.051195 30.790731

O -1.608086 26.990294 9.522850

H -0.493816 25.538664 8.510281

O 50.746815 26.865128 8.461388

H 49.013565 27.798491 7.724573

H 49.486847 28.274216 9.358571

O 22.707283 -1.260944 42.879963

H 24.434175 -0.493816 43.774212

H 23.204521 -1.096176 44.898746

H 21.678089 49.636459 43.717983

H 23.244614 50.437813 43.457386

O 22.754709 50.015865 45.452213

H 13.139451 19.297010 -1.103020

O 15.194415 19.352259 -0.968079

H 13.794126 20.850822 -0.564224

H 14.678596 18.731810 50.124409

H 13.705629 20.148722 50.520439

C 32.761597 50.723831 19.561520

C 33.129761 -1.672623 20.524708

H 31.652710 -0.219528 21.000435

H 31.809166 -0.550533 19.270119

H 28.798347 15.242330 50.073559

H 27.396101 16.175205 49.522537

C 26.996649 13.882133 -0.299225

H 28.719631 17.392635 -0.979324

H 30.023111 16.201118 -0.872250

C 29.166510 18.352402 50.333179

H -0.560802 5.631961 24.383816

C -1.440872 6.367798 26.174273

C 50.305801 7.271337 24.072859

H 26.307261 20.533508 49.846699

H 26.654888 22.268715 50.070629

C 27.693861 20.518841 -1.383175

H 20.546711 -0.946564 10.359406

C 22.114214 -1.293213 11.751384

C 21.132935 50.201660 9.401107

H 25.833490 49.310345 31.384289

H 27.305162 50.219749 30.932030

H 27.166307 49.484890 32.559181

C 27.304672 -1.033108 29.715088

C 1.142136 50.654892 24.118330

H 0.941186 -0.816021 24.615568

H 2.570294 -0.445900 24.018099

C 2.451485 -0.986168 26.111198

H 7.712350 49.802204 32.354813

H 8.973784 50.350292 31.223431

C 9.701798 49.360214 32.983574

C 7.626299 -0.529999 30.171747

H 36.928242 -1.192009 28.604733

H 36.627552 -1.225742 30.364874

H 38.022953 -0.319271 29.702864

C 36.112221 50.262775 30.491016

H 47.989262 -0.562267 25.868690

H 48.466454 -0.612629 27.591187

C 45.963142 49.919548 26.318993

C 49.678017 18.401295 31.706982

H 50.125877 18.746477 33.813282

C -0.675209 20.427900 34.365284

H -1.062439 37.861118 -0.080185

C -0.424389 36.349350 1.241877

H 0.590623 37.343342 -0.404346

C 50.585472 38.644871 1.429625

C 21.645332 50.205082 26.277435

C 23.858219 -1.451630 27.151150

H 22.619764 -0.103165 28.179363

H -1.113289 6.128223 0.034714

H 0.265488 5.923851 -1.082489

H -0.533421 8.021841 -1.607597

H -1.111824 8.928314 -0.200462

C 0.727036 9.617214 -1.069286

O 48.898178 10.513418 46.994293

H 50.484749 9.181579 47.288139

H 50.009998 10.128143 48.722164

H -0.533421 8.021841 47.285202

H -1.111824 8.928314 48.692337

O 0.410211 7.569583 49.104507

H 12.777644 1.978203 50.613827

O 14.219982 0.493817 50.921360

H 14.365194 2.300406 49.871143

O 12.710662 1.775787 -0.988613

H 14.085526 0.291401 -0.451767

H 41.767651 35.133389 49.358746

O 42.932766 34.005920 50.693035

H 43.521435 35.387630 49.238468

H 41.892818 33.009972 -0.616539

H 43.652470 32.893120 -0.361320

O 43.066242 34.310036 -1.760632

H 15.275577 35.596401 50.777618

H 13.616156 36.027637 50.300911

C 14.863411 37.686569 50.835800

C 14.970487 35.166145 -0.690365

H -1.145557 7.511890 18.809059

H -0.357407 9.081348 18.456543

C 49.357281 6.214275 17.135460

H 6.418158 49.968441 21.339750

H 4.887813 -0.200947 19.474491

H 6.434293 -1.026260 19.249096

C 7.741197 -0.606270 22.793335

C 5.839756 50.104851 17.731462

H 3.422007 3.270439 -1.245300

H 2.853873 4.614991 -0.228329

O 1.128935 2.928190 49.102551

H 2.775155 1.653554 49.356304

H 2.615276 2.949214 50.566887

H 5.030091 5.681832 -0.106098

H 5.601159 4.456090 -1.284412

C 28.937202 -1.479984 9.870478

C 27.650835 50.734100 9.154199

H 28.916668 49.314743 8.095181

H 42.764576 45.416523 -0.416569

H 44.347237 46.222763 -0.668854

H 42.858452 47.048077 -1.154358

C 43.911110 46.322506 50.731167

C 39.526405 -1.586082 16.400600

H 42.174442 -1.457497 17.344721

H 42.793911 -0.020535 18.193989

C -1.511768 13.152163 17.299250

H 49.765045 12.005137 17.255735

C 50.857800 13.610289 16.334595

H 49.800251 13.533037 18.173943

O 5.203172 22.083900 -1.101067

H 7.100701 21.801788 -0.653210

H 5.597737 23.243147 50.451992

H 6.601017 23.978008 49.173443

O 7.600874 22.717062 50.519951

C 10.041603 41.547634 -0.671787

H 9.859233 43.666161 -0.710903

C 12.189953 42.567539 50.162544

H -0.162811 22.668657 36.700401

H -1.494164 23.847462 36.848061

H -0.438080 22.441795 39.188065

C -1.711250 24.071392 39.741047

H 49.052189 22.913122 42.081055

H 49.505917 22.503889 43.750252

O 50.746815 23.880711 42.816402

O -1.502964 23.773634 44.093971

H -0.594048 25.116720 42.756264

H 33.870487 -0.842911 43.477921

C 36.045238 -0.796955 43.401161

C 33.427029 50.610405 43.086781

H 49.065392 4.104550 9.752646

H 50.173794 5.438835 9.290609

H 49.815895 5.120054 11.012614

C -1.225254 6.183961 8.765501

H 39.232559 50.118057 46.149422

H 40.746769 49.177357 46.009594

C 39.741047 -1.865749 47.055897

C -0.208282 14.696687 9.269587

H -1.661869 14.545609 10.821444

H -0.479637 13.218657 10.814599

H -1.015015 14.932350 13.259727

H 21.853613 37.236755 50.466171

H 22.708750 35.889271 49.681438

C 23.892445 37.609318 49.943504

C 21.072796 36.542969 -0.974922

O -1.443314 33.665138 22.822670

H -0.378918 35.340691 23.476856

H 49.313766 32.520069 24.297277

O 50.404076 34.233269 24.793537

H 20.205439 50.043736 36.778141

H 19.177223 -1.398335 35.474171

H 18.135805 -0.162323 36.220764

C 21.284014 -0.510441 38.623356

H 17.247423 -0.014668 33.805462

H 18.586109 -1.060486 33.219234

C -2.237823 37.360455 19.318035

H 49.038990 36.570835 19.063791

C 49.866253 37.008915 20.981855

H 49.129440 38.310440 19.420710

H 37.203506 8.037976 49.903900

H 35.932297 7.098257 49.075657

H 35.857979 8.889689 49.102058

C 36.563992 8.029176 -2.192841

O -0.583778 48.602375 12.865163

H -0.619473 48.193142 14.914260

H 50.857311 48.373070 13.315954

O 50.804996 47.832314 15.334249

H 50.197258 49.610546 14.414086

C 22.973259 42.275650 -0.486481

H 24.299232 43.782524 -1.247257

H 22.594830 44.074902 -1.546478

C 24.424887 44.843498 49.821274

H 6.402512 49.716644 10.554488

H 6.100843 49.437466 8.817817

C 7.700616 -1.384644 10.911406

C 2.196265 46.717560 -2.186485

H 0.777396 47.944767 -1.046303

H 2.501844 48.269413 -0.785709

C 1.852548 47.155151 50.289181

H 19.424131 14.543163 -1.186626

H 18.917112 16.179115 -0.690365

H 20.590225 15.631516 -0.372074

C 18.917112 15.669653 50.731659

C 14.930394 -0.936783 21.298193

H 16.146847 49.658459 19.609924

H 15.791396 50.362518 21.200407

C 17.884008 50.035423 20.786283

H 10.905539 23.486633 -1.021370

H 10.467460 25.124054 -1.548923

H 12.188975 24.682552 -1.318642

C 11.387133 26.253477 49.504448

H 49.889236 0.352028 7.745597

H 50.524353 0.688411 9.376172

C 50.970745 2.198220 7.949480

C -0.888382 0.931897 9.605968

H 35.883892 41.288502 -0.984699

O 37.587807 41.626350 49.994354

H 35.756771 41.265522 50.942387

H 35.812019 42.721550 49.930305

H 32.477531 39.993332 -0.429279

H 33.857773 38.859020 -0.476704

H 33.693005 40.133167 -1.729828

H -1.151424 30.484659 28.239992

O -0.381363 32.354813 28.797857

O 49.677040 29.584055 26.790812

H 49.156334 31.582792 26.505278

H 50.581070 31.315350 27.546202

C -0.854156 20.270466 8.009619

H 50.239307 20.926119 6.734494

C 49.578278 22.700928 5.778640

H 49.746956 22.213465 7.862451

O 26.087730 50.755127 36.358150

H 27.422504 49.208645 35.936699

H 27.638609 49.994350 37.510067

O 25.674587 -1.227207 36.327839

H 26.455406 -0.834599 38.209236

C 8.384626 18.353868 -1.740585

H 7.555893 19.450533 49.307899

C 9.431421 19.928217 50.242241

H 8.070246 -0.921631 17.003448

H 9.566365 -1.147026 16.076441

H 8.177810 49.845718 13.534993

C 9.460756 50.185036 15.294645

H 47.891964 15.487771 49.989464

H 48.380402 17.179951 49.749889

O 49.673126 15.793352 48.977383

H 50.183567 14.652194 47.353165

H 49.367058 13.782880 48.651272

H -1.606617 14.264475 47.678791

H -0.844868 15.192460 46.394867

O -1.284901 16.271524 48.080200

C -0.533421 16.191828 49.267811

O -1.284901 16.271524 -0.812599

H -1.000835 15.487771 1.096663

H -0.512398 17.179951 0.857089

C -0.859535 15.052627 -1.408112

H 1.290768 14.652194 -1.539635

H 0.474257 13.782880 -0.241528

C 48.033264 15.052627 -1.408112

C 49.369503 14.731400 -0.805756

C 31.486473 25.101564 -1.235519

H 33.003616 26.598661 -0.779842

H 33.647533 24.959286 -0.845356

H 30.791218 24.945595 50.682766

H 30.717390 26.382555 49.647705

H 49.307899 40.265663 21.035149

C 49.943996 42.308407 20.607826

H 49.205711 41.318813 22.443750

H -0.635117 41.393135 19.013920

H -1.805122 42.675102 19.393328

C 35.462437 -1.694622 0.935808

H 34.075836 -0.176502 1.754272

H 33.501835 -0.984699 0.296291

H 35.437988 -1.020882 -1.440872

H 36.650043 0.250333 -1.199341

H 35.437988 47.871918 -1.440872

H 36.650043 49.143131 -1.199341

C 34.413685 48.271374 49.698551

C 35.462437 47.198177 49.828609

H 5.709212 50.458836 47.788311

H 6.579993 50.234905 49.297146

C 5.363051 -1.020390 47.770710

O 6.722271 -1.335754 47.623543

H 8.219857 -0.027870 48.102203

H 49.784603 37.584385 38.493301

H 50.936028 38.792526 37.922722

H -0.944118 38.706474 37.569717

O -0.430744 40.449505 38.564194

H -0.913807 38.988098 39.968887

H -0.209259 40.485195 40.607426

H 30.077873 31.262056 -0.565689

H 31.539766 30.408875 -1.063416

H 30.938875 28.571486 49.128464

H 29.546406 29.412441 49.803673

O 31.299215 29.712643 50.808418

C 32.279026 30.625473 50.374252

H 9.877323 -0.703568 37.370235

C 8.588998 50.133209 39.393417

H 7.433661 -1.302505 38.759766

H 9.037345 -1.711735 39.368969

H 24.345680 50.390873 7.719195

H 25.479992 49.170998 7.128570

H 23.722298 -0.857578 6.108178

O 23.137539 -1.450649 7.994950

C 24.353504 -1.402733 8.701941

H -0.852692 45.259575 35.500084

H 50.529243 46.715603 35.535286

O 49.713711 47.394722 33.761456

H 48.958317 47.503754 35.659962

H 49.082993 46.639332 31.956333

H 50.325848 45.679077 32.773819

H 49.789982 39.789940 32.566025

H -1.464340 41.529545 32.176842

O -0.170635 42.649677 33.332664

H -1.188583 41.617550 34.816563

H 9.939906 -1.384155 21.107512

H 11.684401 -1.227207 20.938829

O 10.638584 50.301399 19.812828

C 10.115432 50.257397 21.116800

H -1.165115 40.316513 12.188486

C 50.567379 39.325943 11.468295

H 49.101570 41.035725 10.281178

O 50.861713 40.701298 11.334329

H 49.535252 42.263912 11.495186

H 43.430496 15.763527 -0.848289

H 44.944706 16.338018 -1.532791

H 43.622154 17.170172 49.907326

O 45.407719 16.268591 50.417767

H 45.075249 17.897697 49.227715

H 44.048988 14.701088 50.455414

H 45.761215 14.252739 50.544399

H 48.583797 21.218987 49.157307

O 49.157310 21.706448 47.250980

H 50.108273 22.113235 49.060013

C 49.489292 22.998684 46.756672

H 49.943996 24.468403 48.293861

H -1.027725 38.125626 3.579442

H -0.202904 39.625656 4.026322

O -0.825798 38.486454 5.617294

H -1.476074 36.524876 5.659831

H -0.325134 36.966381 6.900730

H 49.151443 35.991455 4.154910

H 50.353230 36.319038 5.381630

H 50.217793 27.443527 46.819744

H 49.991909 28.594954 45.485950

C -1.343086 28.008240 45.312866

C -1.207161 29.170910 46.256012

H 49.920525 13.868443 37.208397

H 50.206059 14.617481 34.795536

H 50.418255 16.469051 36.261345

C -1.140182 9.476402 34.748112

H -0.167702 11.437493 34.749092

H -0.266956 10.686988 36.342506

H 50.471058 7.997396 34.428352

H 49.696598 9.045168 33.225101

H -1.190540 29.278475 37.971123

H -0.194592 30.564846 38.653183

O 50.800598 28.687363 37.497353

H 49.419865 27.655235 38.591576

H 50.213882 28.974361 39.453068

C 50.498928 29.906260 36.865170

O 42.638435 -0.419010 20.911451

H 42.796356 -1.302994 22.786978

H 40.788330 -0.385765 20.021112

H 41.083153 -1.772854 21.062040

H 40.116055 49.830078 21.921087

H -1.173428 14.524096 22.426638

H -1.434025 14.425332 24.166733

C -1.587547 16.463182 23.337023

H 50.402611 16.866549 23.165897

H 49.368526 16.302326 21.833569

H 50.102898 30.812731 41.590172

H 50.921848 30.027512 42.939121

C -0.919674 30.870424 43.052555

H 49.948883 32.785557 42.879475

H 50.480839 31.977846 44.341370

O 22.521490 -0.622894 -0.962212

H 24.323679 -1.509808 -0.536842

H 23.162964 -1.478519 0.809176

H 21.283525 1.016970 -0.929451

C 23.557039 -0.894249 48.847328

O 24.162821 0.286023 49.326477

H 22.887697 1.688757 50.164013

H 21.283525 49.909771 47.963348

H 21.287926 48.951469 49.450665

C 23.091581 50.109253 49.194958

H 49.502495 44.455780 10.438124

H 49.978218 43.450050 9.058369

C -1.770897 44.507114 9.732111

H -1.634487 46.261879 8.391471

H -0.791084 46.439846 9.926705

H 16.521366 3.247949 -1.228676

C 18.050734 2.618698 50.306290

H 19.474491 4.151977 49.689262

H 20.021112 2.524335 49.315231

H 15.352340 -0.251308 17.242535

H 16.258322 -1.162670 16.037817

C 16.560968 -1.906330 18.062956

O 17.762266 -1.554790 18.740610

H 18.727898 -1.414471 16.894417

H 19.175756 -0.201927 18.110382

H 25.557243 32.376324 50.098007

C 27.256269 33.311153 -0.610668

H 24.380884 34.327148 -0.731926

O 26.412868 34.374084 -0.982746

H 22.251114 -1.302505 16.988293

C 21.904463 49.038010 14.994443

C 23.387871 49.014545 15.258465

O 29.404617 -1.032127 35.436523

H 31.153027 -0.643917 34.393642

C 30.086184 -2.008514 36.210987

H 32.235023 -1.765518 36.487720

H 31.303616 -1.312771 37.911964

O -1.982601 24.853676 32.177326

H -1.092754 25.632050 30.502262

H -0.370605 26.165960 32.029671

H -1.993359 23.403027 33.615753

H -1.307396 24.937773 34.122776

H 49.124062 22.743954 32.832981

H 49.725445 24.312922 33.348312

H 30.816154 33.702297 -0.776905

C 31.766140 35.668278 -0.662498

H 28.832573 36.756142 -0.690365

H 29.490669 35.568535 -1.832500

O 30.876780 36.763962 -0.841446

H 2.842627 16.337528 -1.554302

C 2.523846 18.452143 -2.069145

H 4.292299 17.327608 -1.489765

O 1.913175 19.281364 -1.092266

H 3.774035 19.478401 -0.158901

O 23.225546 9.306255 50.627518

H 24.557875 8.390004 49.323055

C 24.185801 10.326648 50.872959

H 24.265007 11.980692 49.413017

H 25.611515 10.849801 49.302521

C 50.783974 16.342419 31.896685

C 25.812464 -1.077106 19.514093

C 26.305794 -2.023182 21.594482

C 27.526157 -2.103367 22.460375

C 26.998604 -1.638401 24.673262

C 16.087198 48.975430 46.143066

C 17.885963 49.731800 44.773094

C -2.657810 7.133459 2.333653

C -1.753296 7.158395 4.250251

C 27.272892 4.510361 50.863178

C 28.192078 6.418158 50.909626

C 5.363051 47.872410 -1.122089

C 6.193740 49.693176 -0.469372

C 4.210648 49.497604 3.275329

C 5.701389 51.089554 3.075357

C -2.331211 43.587929 5.960032

C -1.200317 41.857128 5.517063

C -2.474464 19.862700 21.919621

C -2.600117 19.710644 24.018589

C 13.035799 -0.246910 38.827240

C 12.969304 -0.381363 41.137913

O 39.010098 50.450523 1.845214

C 8.517125 -1.541103 44.303722

C 9.971198 -0.775440 42.947437

C 10.997458 21.718670 50.152279

C 12.260358 18.432096 51.375576

F 50.493061 33.758522 4.641394

F 49.082016 33.249550 6.208408

F 40.884647 2.235379 -0.871269

F 42.470730 0.772995 -1.081020

F 50.461281 12.146927 28.482500

F 49.954262 13.774080 29.828030

F 50.488659 27.535934 18.938137

F 8.847641 35.541645 -1.974781

F 8.358224 37.154617 -0.633652

F 6.143381 30.506662 -0.778862

F 18.753323 6.359487 -0.204861

F 19.862700 8.004729 -1.070751

F 10.538843 -1.643288 24.320257

F 11.516699 -1.457497 26.240767

F 26.509188 -1.442829 0.975902

F 27.606340 -0.088497 2.266671

F 16.898329 25.221352 50.583023

F 17.426373 23.501791 49.359726

F 22.119593 18.379782 49.599300

F 23.601044 19.738512 50.441235

F 50.102406 36.560570 13.442587

F 49.558231 2.339521 21.218496

F 43.232479 -0.575958 7.920633

F 45.375450 -0.609695 8.234036

F 41.129601 -0.238598 11.052217

F 40.763393 -1.885303 12.434417

F -0.926517 33.340000 36.156715

F -0.251308 34.881100 37.534023

H 49.386127 1.242855 37.466064

H 50.803532 0.720680 38.404804

O 50.758060 2.759021 37.931522

H -1.598305 -0.366207 40.971188

H -1.856461 1.390511 40.796642

H -0.571556 0.779351 41.884018

H 45.959232 -2.412373 44.061703

H 45.200901 -1.475582 45.373497

C 47.190350 -2.072567 45.788597

H -2.333656 45.144680 37.678257

O -3.135494 46.612926 38.925514

H -2.251514 46.805077 37.037762

H 50.360073 44.277321 37.630341

H 49.271229 44.134552 39.038456

H 50.914516 28.188164 32.957169

O 51.331573 26.236855 33.537037

H 51.605373 28.029263 34.573078

H -2.021717 27.455263 34.130596

H -2.066700 29.149397 33.562462

H -1.515678 28.781725 35.216507

C -3.412228 0.390653 30.505196

H -2.243690 1.700492 31.673733

H -1.802677 1.706359 29.948305

C -2.561005 26.125866 10.104675

C 51.476784 27.747643 7.621899

C 23.207945 -2.581051 42.816891

C 22.463797 51.366287 45.722103

C 15.688233 20.189304 -2.001183

H 32.390503 50.576180 18.532814

H 31.940199 51.119377 20.179035

H 33.565395 51.472874 19.540985

O 32.194439 -2.615276 21.012657

H 33.663181 -2.049587 19.632416

H 33.897869 -1.441360 21.284990

H 26.048616 14.446845 -0.243973

H 26.880774 13.093492 -1.054131

H 28.518194 18.977741 49.698551

H 28.526991 17.806269 51.047993

H 29.833410 19.018322 50.897404

O -1.949844 5.079473 26.468607

H -2.194309 6.993137 25.663342

H -1.137737 6.909531 27.088568

H 49.988487 6.834236 23.110159

H 50.717480 8.273150 23.884623

H 51.101284 6.635731 24.497736

H 27.030384 20.929052 -2.166927

H 27.294405 19.527784 -1.098133

C 29.070190 20.352606 -1.961090

O 21.376909 -2.180618 12.575716

H 22.846138 -0.706013 12.336143

H 22.683815 -1.846191 10.984745

H 21.814011 50.884205 8.872577

H 20.305180 50.786907 9.835276

H 20.718813 49.493690 8.661359

H 26.301880 -0.698677 29.397285

C 27.383389 -2.514557 29.482357

H 28.040998 -0.510929 29.080462

H 1.828102 50.670544 23.254881

H 0.166724 50.252018 23.795637

H 0.992035 51.689957 24.455690

H 1.895574 -0.597469 26.984425

O 2.292583 -2.385967 25.988968

H 3.511970 -0.704056 26.251032

O 9.719889 50.521904 33.794704

H 10.687966 49.164646 32.522514

H 6.611285 -0.335407 30.560934

H 7.625810 -1.504921 29.669617

H 35.222370 50.904251 30.393721

H 35.977764 49.668240 31.410690

C 37.327209 51.139423 30.674366

H 45.930382 49.439911 25.322559

C 44.545250 50.123920 26.764408

H 46.473583 50.893494 26.196760

H 50.666630 18.809549 31.427803

H 48.953426 18.859909 31.018568

H -1.466297 19.737535 34.702152

H -1.103020 21.437536 34.309055

H -0.397499 35.437012 0.620450

O -1.718582 36.520966 1.806589

H 50.874912 37.730083 1.975269

H 50.817707 39.511738 2.062787

H 51.185875 38.701584 0.506040

H 21.050306 50.343449 25.365585

H 20.949097 50.140053 27.129637
[truncated: 18,757 more chars]
