# Supplementary material for: Unlocking Li‒S chemistry via acoustic-induced entropy-driven electrolyte
Source: Nat Commun. 2026 Apr 30;17:5915. doi: 10.1038/s41467-026-72486-6 (PMC13338023; doi:10.1038/s41467-026-72486-6)
Supplement: Supplementary file 3 — Supplementary Data [file 41467_2026_72486_MOESM3_ESM.zip › Supplementary Data files/Supplementary Data 2.docx]

model-US

O 12.173329 6.838147 16.324816

C 12.044252 5.926296 17.401924

C 11.455583 4.671707 16.797611

C 12.752220 8.092247 16.625019

O 11.086931 3.678205 17.751509

C 10.306602 2.663191 17.132036

H 11.374421 6.327706 18.180298

H 13.023087 5.723880 17.872274

H 12.192397 4.292788 16.069107

H 10.572579 4.971420 16.212362

H 12.760531 8.661848 15.683343

H 13.791680 8.005218 16.983892

H 10.831711 2.125370 16.325306

H 9.373239 3.072423 16.714005

H 10.054804 1.912197 17.893787

H 12.165017 8.665271 17.362322

O 14.213137 42.567051 28.764612

C 13.418139 43.636337 29.241804

C 13.490012 44.768204 28.256615

C 14.549520 41.662533 29.792828

O 13.194700 45.957275 28.965071

C 13.201056 47.096478 28.131941

H 13.804393 44.022591 30.199125

H 12.377212 43.308754 29.421244

H 12.801601 44.608326 27.406370

H 14.517250 44.798027 27.848362

H 15.036981 40.807888 29.307322

H 13.664560 41.297302 30.342381

H 12.492599 47.000648 27.293427

H 14.202380 47.298405 27.719774

H 12.891564 47.949169 28.752876

H 15.257977 42.103546 30.514975

O 5.361584 14.871234 3.977918

C 4.349504 14.675174 3.011796

C 4.651172 13.406895 2.259825

C 5.188015 16.081820 4.683930

O 3.465522 12.959036 1.607595

C 3.731498 12.312675 0.375497

H 4.278609 15.526886 2.308718

H 3.361869 14.571033 3.490457

H 4.997333 12.636344 2.971215

H 5.480394 13.583397 1.553324

H 6.005991 16.145868 5.416344

H 4.228738 16.118979 5.228107

H 4.392529 11.440915 0.500662

H 4.193536 13.002552 48.541752

H 2.767821 11.971401 48.862976

H 5.249131 16.955534 4.014099

O 17.991573 6.808322 27.357475

C 19.158155 6.479274 28.090380

C 19.679352 7.693771 28.808125

C 17.673281 5.794775 26.424603

O 21.062529 7.472775 29.010052

C 21.667334 8.530816 29.716555

H 19.969286 6.156092 27.414192

H 18.967962 5.647607 28.794437

H 19.154732 7.881031 29.760069

H 19.507738 8.578241 28.167141

H 16.780008 6.131646 25.879448

H 17.447886 4.831586 26.914999

H 21.292326 8.595842 30.752106

H 21.514299 9.502805 29.220291

H 22.746885 8.327910 29.746868

H 18.481968 5.634406 25.689255

O 31.025904 7.468375 5.636851

C 32.429127 7.527047 5.833889

C 33.041264 8.164120 4.610591

C 30.396652 6.743295 6.673378

O 33.795681 7.175996 3.933426

C 34.333988 7.660524 2.722840

H 32.860363 6.514966 5.939497

H 32.663322 8.064867 6.770185

H 33.663670 9.046635 4.843321

H 32.220844 8.522993 3.970584

H 29.316124 6.756985 6.472428

H 30.576092 7.195064 7.663946

H 34.994534 8.528371 2.883209

H 33.545349 7.940680 2.005094

H 34.929996 6.846947 2.287205

H 30.729612 5.691611 6.702225

O 37.301296 7.410192 46.975224

C 36.283836 6.679245 47.627941

C 34.975952 7.399926 47.483219

C 38.522636 6.697336 46.999180

O 34.046013 6.785343 48.354000

C 32.746441 7.319252 48.228832

H 36.504833 6.575104 48.704563

H 36.185562 5.664231 47.203064

H 34.644947 7.352499 46.430069

H 35.122631 8.464322 47.738441

H 39.277054 7.337342 46.524921

H 38.464455 5.749793 46.433983

H 32.347477 7.201910 47.207462

H 32.706841 8.385604 48.508011

H 32.103500 6.751606 0.022982

H 38.862930 6.471451 48.023975

O 12.941436 5.242286 27.354544

C 14.155932 4.508405 27.302717

C 14.455156 3.907024 28.637980

C 12.815781 6.027504 26.182583

O 13.298352 3.242571 29.128374

C 13.635714 2.239290 30.072004

H 14.995421 5.156235 26.992249

H 14.075747 3.705096 26.544878

H 14.780293 4.676107 29.361105

H 15.305402 3.218124 28.521614

H 11.810056 6.452872 26.183559

H 12.929702 5.438835 25.255087

H 14.134419 2.649501 30.963320

H 14.283053 1.460917 29.633438

H 12.699415 1.765030 30.389807

H 13.539393 6.861126 26.163515

O 3.008374 19.719933 48.830708

C 4.346081 19.593300 0.386742

C 4.437510 20.212282 1.762097

C 2.845561 19.152287 47.547268

O 5.092674 19.287720 2.612342

C 5.195838 19.778116 3.932448

H 5.052582 20.045071 48.557884

H 4.625259 18.528416 0.475727

H 3.410273 20.405899 2.118036

H 4.960175 21.187206 1.759652

H 1.802678 19.320969 47.250004

H 3.046510 18.067356 47.542381

H 4.211137 19.825542 4.423332

H 5.659341 20.777973 3.975473

H 5.838289 19.084326 4.493737

H 3.492413 19.630947 46.794811

O 25.467770 46.637863 35.932785

C 24.070414 46.795788 35.759216

C 23.825462 47.276894 34.353062

C 25.762594 46.169472 37.232357

O 22.774265 48.229813 34.366749

C 22.653011 48.804790 33.082821

H 23.680250 47.558514 36.454960

H 23.526726 45.859001 35.973854

H 23.592731 46.443760 33.663670

H 24.765179 47.733551 33.993698

H 26.855347 46.113731 37.321339

H 25.346027 45.162766 37.410816

H 22.468685 48.049400 32.301029

H 23.552639 0.484527 32.807068

H 21.798855 0.596492 33.107269

H 25.391985 46.850548 38.018063

O 18.880444 45.724056 25.830065

C 17.916767 46.378735 26.636309

C 17.852228 45.700588 27.966682

C 18.966984 46.356239 24.567652

O 17.963215 44.304699 27.753998

C 17.614609 43.538059 28.888800

H 16.923754 46.341084 26.148359

H 18.177366 47.444107 26.758053

H 16.904686 45.961678 28.463921

H 18.658470 46.046749 28.635534

H 19.699398 45.795929 23.972139

H 19.312656 47.400105 24.649794

H 16.578571 43.728252 29.211981

H 18.289330 43.716518 29.741488

H 17.695282 42.481976 28.592999

H 18.002329 46.347931 24.029833

O 41.102711 33.035885 1.599772

C 42.094254 33.819637 2.240268

C 41.782318 33.812794 3.706074

C 41.239120 33.083801 0.195082

O 42.723507 34.596546 4.415509

C 42.465839 34.539341 5.802109

H 42.088390 34.863010 1.874550

H 43.107803 33.411381 2.069143

H 41.790142 32.762577 4.049790

H 40.754593 34.200024 3.838085

H 40.382519 32.546959 48.657627

H 42.165638 32.590473 48.752964

H 42.515713 33.508190 6.189828

H 41.489452 34.983288 6.061240

H 43.248123 35.116276 6.304727

H 41.228367 34.116909 48.698208

O 0.420478 27.082699 35.588581

C 1.567503 26.426067 36.093639

C 2.116569 25.513241 35.036579

C 0.012712 28.154919 36.410957

O 3.080246 24.677172 35.647739

C 3.694829 23.816660 34.709976

H 1.311305 25.817842 36.978603

H 2.355655 27.139416 36.396778

H 2.554649 26.111198 34.215183

H 1.286370 24.922615 34.609257

H 48.022995 28.603266 35.931808

H 0.793530 28.928402 36.496029

H 4.243895 24.372572 33.929157

H 2.964370 23.149761 34.220558

H 4.415509 23.197187 35.263443

H 48.626331 27.830271 37.426460

O 44.658684 6.151203 15.142589

C 44.743755 6.979447 13.985297

C 46.045284 6.842058 13.239681

C 44.183933 4.845276 14.870256

O 46.027191 7.725552 12.130303

C 47.318451 7.841427 11.568036

H 44.670906 8.017930 14.346614

H 43.896442 6.822990 13.296397

H 46.191963 5.798686 12.906721

H 46.868637 7.076744 13.937381

H 44.254341 4.283498 15.811931

H 44.787270 4.324568 14.108994

H 47.746754 6.859660 11.312816

H 48.014683 8.361157 12.247158

H 47.227512 8.430585 10.645918

H 43.130783 4.853099 14.541697

O 1.822235 21.990025 31.948511

C 3.067534 22.182663 31.299702

C 4.132908 21.367132 31.972469

C 0.786196 22.573317 31.184317

O 5.383097 21.904953 31.582792

C 6.473896 21.153469 32.072208

H 3.371648 23.242659 31.355440

H 3.020108 21.905441 30.229929

H 4.035123 20.300289 31.696226

H 3.995031 21.439981 33.064735

H 48.736832 22.385569 31.716269

H 0.707968 22.120081 30.180548

H 6.465584 20.114986 31.696712

H 6.506654 21.131468 33.175232

H 7.386235 21.645821 31.707468

H 0.921140 23.662159 31.065508

O 17.479176 13.794614 20.479727

C 18.800749 13.640602 20.961321

C 19.424131 12.546869 20.142855

C 16.720848 14.792516 21.127556

O 20.751081 12.278937 20.558933

C 21.372509 11.427225 19.614813

H 18.817371 13.367291 22.031586

H 19.390884 14.566632 20.838600

H 19.389418 12.870541 19.086771

H 18.799282 11.639909 20.230864

H 15.730280 14.786650 20.650850

H 17.157461 15.797753 21.001902

H 21.462473 11.903440 18.623266

H 20.829309 10.473816 19.493559

H 22.382633 11.208185 19.983465

H 16.586393 14.585212 22.203199

O 16.766808 6.609328 48.614601

C 15.517108 6.799033 47.972637

C 14.494269 6.069063 48.801857

C 17.818981 7.281116 47.955524

O 13.217191 6.039728 48.186298

C 12.374278 5.157701 0.012222

H 15.524442 6.390778 46.945396

H 15.273622 7.873696 47.900276

H 14.428754 6.545768 0.900115

H 14.881013 5.048671 0.072361

H 18.712252 7.165240 48.584778

H 17.618031 8.358713 47.833782

H 12.240802 5.467193 1.063417

H 12.760531 4.123619 0.007332

H 11.391533 5.162102 48.412674

H 18.029221 6.844992 46.965446

O 35.767525 29.124462 24.500181

C 36.552258 30.273932 24.238115

C 36.188496 30.762373 22.863251

C 35.993900 28.625755 25.808552

O 37.164394 30.292513 21.944555

C 37.010872 30.889980 20.668943

H 37.631321 30.032892 24.256697

H 36.388954 31.047417 25.012577

H 36.102444 31.860014 22.821692

H 35.189125 30.364384 22.611452

H 35.200859 27.895298 26.023191

H 35.951366 29.413908 26.580082

H 37.021137 31.993492 20.714413

H 36.077019 30.573647 20.173658

H 37.863071 30.568266 20.051916

H 36.971268 28.123138 25.887758

O 34.029877 10.134010 10.241575

C 35.099651 9.765359 11.106977

C 35.927898 11.000879 11.366598

C 33.619175 9.083794 9.385462

O 37.267071 10.658141 11.696136

C 38.035175 11.844280 11.795877

H 35.753349 9.012410 10.640540

H 34.701664 9.306255 12.028606

H 35.465370 11.627196 12.151327

H 35.902958 11.592483 10.432745

H 32.818314 9.484225 8.747411

H 33.230480 8.215457 9.941373

H 37.647453 12.528292 12.570338

H 38.077225 12.391880 10.838555

H 39.061924 11.570481 12.073588

H 34.441555 8.743499 8.733232

O 0.475238 38.157894 25.936165

C 0.348117 39.355770 26.680801

C 0.698678 39.081482 28.118250

C 0.125654 38.381824 24.584278

O 0.869314 40.270065 28.884399

C 48.549572 40.769749 29.420753

H 48.218567 39.770870 26.606482

H 1.046306 40.127785 26.310682

H 1.673112 38.569576 28.134382

H 48.859550 38.393070 28.573441

H 0.261088 37.435749 24.040588

H 0.762728 39.143574 24.111973

H 48.786701 41.709957 29.940485

H 48.121273 40.070091 30.155123

H 47.793690 40.983902 28.646292

H 47.967747 38.699627 24.478180

O 0.417056 31.866369 1.948378

C 1.298593 31.390644 0.947542

C 1.375843 32.445263 48.779858

C 0.296779 30.928606 2.993706

O 2.503800 32.214489 47.954544

C 2.721862 33.341957 47.132656

H 0.966122 30.425501 0.527553

H 2.316541 31.242498 1.353842

H 1.464339 33.403561 0.425367

H 0.438080 32.487309 48.192165

H 48.547615 31.374512 3.766701

H 1.275124 30.687567 3.442542

H 2.944324 34.246960 47.723282

H 1.856949 33.552197 46.478474

H 3.592643 33.126827 46.499985

H 48.719719 29.994267 2.646078

O 27.610741 12.009538 30.345806

C 28.954315 11.597861 30.199125

C 29.029121 10.715835 28.984629

C 27.462109 12.968815 31.370110

O 30.337004 10.182903 28.864841

C 30.506662 9.582989 27.594606

H 29.302921 11.051240 31.091908

H 29.635393 12.457397 30.068583

H 28.748476 11.320149 28.102114

H 28.277641 9.909103 29.067259

H 26.394287 13.222569 31.418024

H 28.029263 13.893867 31.164268

H 30.327713 10.298779 26.772718

H 29.840252 8.713675 27.451351

H 31.549057 9.241228 27.522247

H 27.772577 12.578161 32.352856

O 6.038750 8.161675 47.374191

C 6.057817 9.395729 48.067490

C 6.270990 9.055435 0.620447

C 5.442257 8.193455 46.096130

O 6.386867 10.229352 1.402244

C 6.713959 9.869012 2.731154

H 5.114187 9.950173 47.948681

H 6.868460 10.049426 47.703236

H 7.174041 8.423251 0.691345

H 5.419767 8.438408 0.961232

H 5.329315 7.143727 45.791527

H 6.072486 8.718076 45.365185

H 7.737774 9.463691 2.806446

H 6.022126 9.122418 3.149184

H 6.645020 10.773528 3.348180

H 4.442889 8.657937 46.098087

O 45.304066 26.150803 11.726449

C 45.348560 27.045542 10.627339

C 44.572632 28.283997 10.973989

C 45.810596 24.883989 11.357308

O 44.514938 29.129843 9.834298

C 43.779102 30.303755 10.122276

H 44.889946 26.601595 9.724289

H 46.391445 27.310053 10.374563

H 45.048847 28.789057 11.832546

H 43.562996 27.973038 11.304503

H 45.692276 24.222471 12.225155

H 46.880371 24.926527 11.089865

H 44.263138 30.908562 10.907495

H 42.743065 30.086674 10.439102

H 43.737541 30.900738 9.200158

H 45.250774 24.441511 10.513907

O 12.405081 44.390751 36.322952

C 12.990328 44.609791 35.049782

C 14.139308 43.668118 34.859589

C 11.687335 45.535332 36.757607

O 13.686073 42.324543 34.698730

C 14.485958 41.600925 33.773678

H 13.365336 45.645340 34.961773

H 12.246181 44.465065 34.245007

H 14.811095 43.734612 35.733791

H 14.721622 43.997650 33.987831

H 11.451182 45.395496 37.820538

H 10.746148 45.668320 36.201206

H 15.506841 41.423935 34.156021

H 14.555876 42.119190 32.801689

H 13.986763 40.636272 33.608421

H 12.282360 46.460869 36.670578

O 32.654034 3.016686 15.568445

C 32.572384 2.779067 16.962868

C 33.095047 3.991119 17.658123

C 32.146038 1.944955 14.805229

O 32.274624 5.098052 17.313429

C 32.870628 6.316949 17.704082

H 33.161541 1.886773 17.241556

H 31.528522 2.596697 17.274803

H 34.139397 4.154421 17.335430

H 33.116070 3.851286 18.754299

H 32.353344 2.199687 13.759901

H 31.057194 1.818323 14.936750

H 33.799103 6.516921 17.141327

H 33.097980 6.336996 18.783146

H 32.149948 7.115858 17.478687

H 32.646702 0.989101 15.031114

O 7.384768 33.416763 48.159897

C 6.381488 33.106293 0.226863

C 6.945222 32.354321 1.410068

C 6.794144 34.196114 47.139503

O 6.163915 32.737640 2.530691

C 6.413758 31.987625 3.701674

H 5.547377 32.540112 48.661537

H 5.941453 34.035744 0.631206

H 7.998862 32.644257 1.558213

H 6.934955 31.261078 1.253122

H 7.546114 34.356480 46.356239

H 6.466073 35.177391 47.523800

H 7.469841 32.028206 4.014099

H 6.116001 30.931051 3.588243

H 5.800153 32.438419 4.495693

H 5.927763 33.692028 46.677467

O 35.172504 13.058778 22.648613

C 34.089527 12.925789 21.741650

C 34.518803 12.893519 20.301268

C 35.681965 11.774853 22.952236

O 33.354179 12.789867 19.512627

C 33.671001 12.525846 18.162697

H 33.448051 13.791192 21.930864

H 33.473965 12.034963 21.948956

H 35.191570 12.031540 20.140900

H 35.091339 13.803905 20.054848

H 36.067242 11.267835 22.051630

H 34.931461 11.120178 23.429430

H 34.303677 11.628663 18.039976

H 34.179489 13.380004 17.689415

H 32.719551 12.342009 17.648346

H 36.495541 11.937177 23.656292

O 5.584047 22.200264 11.627196

C 6.749651 21.413090 11.405712

C 7.496244 20.970121 12.638789

C 5.917984 23.544329 11.916641

O 8.596332 20.191748 12.201687

C 9.287188 19.625570 13.296885

H 7.459574 21.923042 10.732458

H 6.407890 20.511507 10.874737

H 6.822012 20.385365 13.290529

H 7.840939 21.842369 13.221592

H 4.985110 24.118330 11.954778

H 6.557991 23.992674 11.137779

H 8.633001 18.996319 13.924669

H 9.741402 20.390253 13.944226

H 10.090984 18.998276 12.886676

H 6.414735 23.638691 12.894987

O 3.878177 41.716805 34.345726

C 3.686028 42.960636 35.040981

C 4.981198 43.701363 35.267841

C 3.336934 40.620628 35.074226

O 4.723534 44.757935 36.173336

C 5.797708 45.658051 36.350330

H 3.209812 42.813469 36.023727

H 2.991750 43.582554 34.452309

H 5.372830 44.065125 34.304653

H 5.727303 43.003662 35.683918

H 3.409784 39.721977 34.446445

H 2.274493 40.771706 35.333359

H 6.080308 46.166050 35.412567

H 6.691957 45.170101 36.774231

H 5.449102 46.420288 37.062210

H 3.901157 40.443146 36.003677

O 46.608528 35.282509 29.877411

C 47.972637 35.130455 29.515606

C 48.831196 35.967010 30.424522

C 45.790554 34.365284 29.178246

O 1.219877 36.121025 29.831940

C 2.195773 36.506786 30.779974

H 48.142296 35.465370 28.476143

H 48.293373 34.073883 29.571833

H 48.887424 35.495682 31.421448

H 48.359379 36.954643 30.561911

H 44.748646 34.657173 29.359150

H 45.935764 33.334133 29.541029

H 2.409440 35.690277 31.492342

H 1.898020 37.404945 31.345663

H 3.115938 36.731205 30.222593

H 45.968033 34.382397 28.089891

O 26.153248 0.325626 14.286965

C 27.016203 1.233076 13.619089

C 26.541456 1.428648 12.208043

C 26.672977 0.090941 15.583114

O 27.424950 2.289161 11.496163

C 27.239645 2.094568 10.101253

H 28.053221 0.846334 13.585353

H 27.041142 2.206532 14.140776

H 25.520086 1.850104 12.208531

H 26.487185 0.428790 11.742095

H 25.971365 48.330532 16.112133

H 26.768808 1.020393 16.170805

H 26.184538 2.195776 9.795183

H 27.596563 1.101066 9.774159

H 27.820980 2.856317 9.565387

H 27.658657 48.482098 15.556710

O 13.213768 3.701185 19.779581

C 13.408360 4.753358 20.724192

C 12.084345 5.309758 21.184271

C 14.489381 3.317376 19.300432

O 12.291650 6.440160 22.026695

C 11.151958 7.277693 22.121548

H 13.987741 5.576224 20.265087

H 13.985297 4.407197 21.604261

H 11.509854 4.527962 21.710848

H 11.504965 5.576713 20.287088

H 14.398929 2.436328 18.648203

H 15.148945 3.013752 20.118898

H 10.257220 6.751606 22.493622

H 10.904072 7.753420 21.156893

H 11.398868 8.074646 22.834892

H 14.985154 4.142687 18.758701

O 35.708858 8.579708 24.706511

C 34.761803 7.703061 25.291756

C 33.398670 8.187099 24.892302

C 37.029449 8.092247 24.821896

O 32.397346 7.351033 25.448702

C 31.144224 7.592073 24.840475

H 34.853230 7.687415 26.392822

H 34.887455 6.665066 24.935328

H 33.360535 8.194433 23.786346

H 33.276440 9.230472 25.233084

H 37.685593 8.830040 24.337858

H 37.165863 7.122214 24.311945

H 31.144714 7.304584 23.775103

H 30.835222 8.647181 24.918705

H 30.405943 6.977003 25.370472

H 37.340897 7.980283 25.873091

O 9.137575 10.367719 25.352385

C 8.162653 11.307926 24.926527

C 8.220835 12.487709 25.843267

C 9.342925 9.345369 24.397995

O 7.949480 12.119547 27.195642

C 8.026731 13.301775 27.975481

H 7.157417 10.850779 24.923105

H 8.354313 11.654088 23.891956

H 7.517757 13.272439 25.502485

H 9.239272 12.916010 25.758194

H 10.137433 8.700474 24.796963

H 9.684686 9.741402 23.425518

H 9.027078 13.756968 27.919743

H 7.840939 13.045577 29.027655

H 7.280138 14.057169 27.665503

H 8.439875 8.731765 24.230293

O 43.936539 19.849010 20.241129

C 42.522068 19.814295 20.134544

C 42.035095 21.158848 19.663218

C 44.437199 18.813948 21.062040

O 40.619160 21.147602 19.569832

C 40.110188 22.426638 19.236383

H 42.049763 19.595745 21.108976

H 42.206711 19.026144 19.428532

H 42.499088 21.391577 18.686829

H 42.388592 21.921576 20.382919

H 45.528976 18.936182 21.106043

H 44.212292 17.816536 20.655741

H 40.520885 22.806534 18.284441

H 40.305759 23.174698 20.025513

H 39.020367 22.327873 19.123440

H 44.038723 18.868221 22.090256

O 12.530735 18.014551 2.125859

C 11.396912 18.183722 1.296637

C 10.176059 17.731462 2.043719

C 13.710030 18.453121 1.486341

O 9.035389 18.082512 1.279036

C 7.837027 17.820448 1.978692

H 11.488341 17.608253 0.356429

H 11.254145 19.245672 1.024304

H 10.174103 18.224792 3.032820

H 10.224463 16.641642 2.222667

H 14.533873 18.306931 2.196754

H 13.667004 19.524361 1.232099

H 7.754887 18.426229 2.897876

H 7.731907 16.755074 2.248580

H 7.012205 18.093760 1.305927

H 13.929070 17.872763 0.573513

O 12.747332 27.539846 20.070005

C 12.660790 26.328773 20.799974

C 13.404449 26.474962 22.097589

C 11.776320 27.607809 19.042767

O 12.994239 25.432566 22.974728

C 13.026020 25.829088 24.330034

H 13.058290 25.484882 20.213751

H 11.615952 26.083820 21.056173

H 13.155585 27.472864 22.496555

H 14.498671 26.448071 21.955801

H 11.990470 28.512323 18.456543

H 10.752015 27.691904 19.448088

H 12.397747 26.712091 24.518274

H 14.045435 26.056440 24.672285

H 12.627055 24.995466 24.921150

H 11.815434 26.735559 18.368536

O 19.990311 0.260107 13.290529

C 20.304203 0.487948 14.653172

C 21.711336 0.046937 14.938705

C 18.743055 0.858558 12.982017

O 21.809610 48.754433 16.338018

C 23.035843 48.208790 16.772675

H 19.628502 48.812126 15.317136

H 20.201527 1.553324 14.918660

H 22.430550 0.804289 14.575433

H 21.897617 48.002953 14.384749

H 18.514236 0.660541 11.923488

H 18.779236 1.949847 13.134562

H 23.889511 48.872265 16.555590

H 23.228479 47.213333 16.339973

H 22.954679 48.091934 17.862984

H 17.922146 0.463014 13.600021

O 34.881588 14.605745 14.443911

C 35.207703 15.002755 13.122828

C 34.631260 16.372242 12.903788

C 35.348026 13.296397 14.708421

O 34.898701 16.836235 11.585637

C 34.051880 17.940235 11.320639

H 34.774025 14.310922 12.376723

H 36.300457 15.015469 12.963926

H 35.029736 17.074345 13.658692

H 33.545349 16.289614 13.092514

H 35.195480 13.101315 15.777218

H 36.424156 13.182966 14.494760

H 34.170200 18.213057 10.267488

H 34.296833 18.817371 11.945978

H 32.987484 17.686970 11.460961

H 34.788204 12.537091 14.135885

O 42.202801 17.019094 29.912615

C 42.093281 16.761919 28.514280

C 41.762764 18.034109 27.767687

C 42.175415 15.789440 30.613737

O 40.895405 17.732929 26.686178

C 40.660229 18.907333 25.928829

H 41.269924 16.056395 28.309910

H 43.017841 16.288147 28.136337

H 42.670700 18.543571 27.396591

H 41.264545 18.721542 28.472233

H 42.106968 16.016304 31.686445

H 43.082378 15.188060 30.427946

H 41.596527 19.356659 25.553331

H 40.117031 19.674952 26.507719

H 40.049068 18.624245 25.062939

H 41.293880 15.180236 30.350695

O 46.884773 26.227076 38.550503

C 45.581779 26.802544 38.476677

C 45.442924 27.976460 37.538425

C 47.118969 25.357761 37.452374

O 44.109615 28.442898 37.646969

C 43.920403 29.633926 36.910641

H 45.342693 27.156038 39.492180

H 44.813183 26.049595 38.222923

H 45.671741 27.676748 36.498966

H 46.168495 28.761679 37.818092

H 48.119804 24.924082 37.584873

H 47.097458 25.887272 36.483318

H 44.157532 29.510225 35.839401

H 44.528629 30.459236 37.313519

H 42.861870 29.912127 37.003048

H 46.385090 24.534407 37.409348

O 26.916954 12.945836 9.805940

C 28.011663 13.201056 10.668898

C 29.046232 12.140571 10.420522

C 25.920517 13.943737 9.877812

O 30.141432 12.315607 11.303038

C 31.016613 11.206230 11.242410

H 27.706083 13.169275 11.731339

H 28.452187 14.197001 10.486527

H 29.377237 12.193376 9.367372

H 28.568062 11.155381 10.553021

H 25.114765 13.634246 9.197713

H 26.293079 14.926971 9.543874

H 31.932865 11.474651 11.786588

H 31.295792 10.947587 10.207350

H 30.573156 10.317848 11.721071

H 25.502485 14.047878 10.894294

O 19.415331 1.028216 5.646141

C 19.097038 2.105813 4.771448

C 19.303856 3.429341 5.464259

C 18.953295 48.700161 5.096585

O 19.319990 4.456090 4.484936

C 19.410442 5.735125 5.083873

H 18.049755 2.050564 4.423820

H 19.727757 2.073055 3.864976

H 20.258732 3.403917 6.018215

H 18.498590 3.591665 6.206941

H 19.170378 47.897343 5.816776

H 19.454445 48.462055 4.141220

H 20.324247 5.851001 5.692589

H 18.534771 5.957588 5.719969

H 19.444668 6.475851 4.271764

H 17.861029 48.708965 4.928394

O 5.631961 22.285337 6.381000

C 4.294744 21.853127 6.555546

C 3.461610 22.407570 5.431501

C 6.494919 21.710848 7.342232

O 2.279382 21.632618 5.304379

C 1.464339 22.120569 4.257096

H 3.892845 22.157728 7.539270

H 4.228738 20.754015 6.506654

H 4.064948 22.363565 4.506449

H 3.233770 23.472944 5.608493

H 7.509934 22.070698 7.122214

H 6.507632 20.607338 7.292850

H 1.975758 22.082922 3.279240

H 1.131868 23.158564 4.436533

H 0.574001 21.477629 4.212114

H 6.236766 22.012028 8.372403

O 35.764595 27.959347 5.229574

C 36.988380 28.258574 4.580277

C 37.498333 27.013762 3.896756

C 35.263443 29.083393 5.924829

O 37.961346 27.377523 2.606964

C 38.606243 26.293570 1.974780

H 37.731552 28.660469 5.291179

H 36.834366 29.030590 3.805327

H 36.666180 26.294548 3.815105

H 38.291862 26.516520 4.485915

H 35.921051 29.381149 6.758940

H 34.289497 28.793947 6.343352

H 37.930058 25.432566 1.839347

H 39.498539 25.965498 2.537047

H 38.934315 26.638266 0.984701

H 35.119209 29.955153 5.263309

O 20.676277 47.245602 42.003803

C 20.391720 47.384457 43.388447

C 19.405552 46.329350 43.797680

C 21.871216 47.924721 41.674755

O 19.321945 46.310768 45.212151

C 18.625711 45.168633 45.666367

H 20.004490 48.395069 43.615311

H 21.296236 47.236313 44.004986

H 19.781538 45.367138 43.404095

H 18.417429 46.515144 43.337601

H 21.984159 47.897343 40.583469

H 22.744930 47.423573 42.119678

H 19.153265 44.235271 45.406742

H 17.597986 45.117786 45.267887

H 18.568996 45.236107 46.761562

H 21.857527 0.084583 42.000870

O 46.656933 16.088177 23.487123

C 48.033752 15.983056 23.803459

C 48.136917 15.598759 25.254597

C 46.409534 16.244633 22.106390

O 0.556889 15.137700 25.514217

C 0.696722 14.693264 26.848503

H 48.576462 16.924730 23.599577

H 48.531971 15.199305 23.206478

H 47.393257 14.803761 25.441366

H 47.867519 16.450472 25.904871

H 45.324604 16.380066 22.002249

H 46.694576 15.350872 21.527988

H 0.095341 13.790703 27.049942

H 0.419011 15.472126 27.579451

H 1.756229 14.440488 26.997625

H 46.923889 17.122259 21.680534

O 39.036991 11.517188 32.432549

C 40.417721 11.208185 32.511757

C 40.682720 10.157969 31.474741

C 38.617977 12.368412 33.478367

O 42.016029 9.700332 31.550522

C 42.246799 8.718076 30.557510

H 40.688587 10.813131 33.506233

H 41.046970 12.099011 32.331341

H 40.456837 10.571112 30.475859

H 39.972797 9.334125 31.658087

H 37.561893 12.609942 33.291107

H 39.192955 13.309597 33.504768

H 42.154884 9.123396 29.537119

H 41.550568 7.866363 30.642586

H 43.272083 8.343556 30.691477

H 38.692295 11.879972 34.466492

O 33.312618 24.081659 5.309269

C 34.601925 24.400928 4.816919

C 35.527462 23.277863 5.174325

C 32.421307 25.162678 5.142056

O 36.803566 23.529171 4.616458

C 37.713463 22.506823 4.972398

H 34.991108 25.336737 5.260376

H 34.593609 24.533428 3.719275

H 35.105518 22.327873 4.800784

H 35.579781 23.210390 6.277347

H 31.448826 24.836563 5.529287

H 32.299561 25.434034 4.080593

H 37.545761 21.590572 4.383239

H 37.665546 22.252581 6.044617

H 38.723099 22.884764 4.769003

H 32.737640 26.057907 5.706279

O 36.764942 34.019119 47.160526

C 37.150215 33.945293 48.527569

C 36.216362 32.976238 0.316824

C 37.695862 34.781361 46.414913

O 35.976788 33.379604 1.654046

C 34.962265 32.595852 2.248579

H 37.055851 34.933907 0.118808

H 38.208256 33.646069 48.640514

H 36.612396 31.947044 0.281620

H 35.278599 32.972816 48.629757

H 37.283695 34.942219 45.408211

H 38.653183 34.247936 46.327881

H 35.225307 31.524122 2.278893

H 33.993206 32.708305 1.731296

H 34.844433 32.952278 3.280220

H 37.884098 35.770462 46.863258

O 17.444462 36.936066 7.373523

C 16.941355 37.567760 8.546950

C 15.685299 36.918953 9.055435

C 18.855019 37.033360 7.333920

O 15.391453 37.544292 10.290468

C 14.249318 37.002071 10.920695

H 16.757519 38.637047 8.350890

H 17.662035 37.512512 9.382039

H 15.872559 35.839401 9.190868

H 14.857055 37.024563 8.331822

H 19.194336 36.470116 6.456783

H 19.332703 36.584526 8.218880

H 14.357861 35.925453 11.138268

H 13.337955 37.151680 10.317848

H 14.124641 37.531578 11.876060

H 19.194824 38.080158 7.255202

O 35.961632 0.252286 17.636122

C 34.949551 48.226879 17.259647

C 34.968132 48.035706 15.767927

C 36.206585 0.177483 19.026144

O 33.684204 47.602516 15.340116

C 33.760967 46.766453 14.203847

H 33.958008 48.611668 17.553982

H 35.075695 47.248535 17.758354

H 35.760685 47.318451 15.493150

H 35.210148 0.095832 15.274111

H 36.852459 1.024302 19.290165

H 36.722404 48.132030 19.300432

H 34.310036 45.835533 14.426310

H 34.235226 47.265648 13.341866

H 32.731285 46.500965 13.942760

H 35.277622 0.248375 19.611879

O 42.164173 2.164484 32.150436

C 43.494545 1.739606 31.900108

C 44.349190 2.955570 31.667377

C 41.310993 1.066352 32.401749

O 45.568089 2.595719 31.034704

C 46.212498 3.750567 30.529642

H 43.538059 1.116223 30.988258

H 43.893021 1.134802 32.735683

H 44.541832 3.488501 32.616386

H 43.764923 3.633713 31.027859

H 40.298912 1.467762 32.550381

H 41.603371 0.513863 33.312134

H 46.378242 4.513294 31.308504

H 45.640453 4.214070 29.705797

H 47.188396 3.438631 30.136055

H 41.279701 0.357406 31.556391

O 31.745117 39.066814 0.840467

C 31.210718 38.867332 2.141994

C 29.959553 39.689220 2.308718

C 33.112160 38.705986 0.783752

O 29.155266 39.055569 3.286085

C 28.007750 39.807049 3.621001

H 31.936775 39.123528 2.935035

H 30.948652 37.804890 2.282805

H 29.431509 39.734200 1.340641

H 30.210371 40.729168 2.592296

H 33.460766 38.888355 48.651272

H 33.262260 37.645500 1.033594

H 27.352589 39.984039 2.751198

H 28.265905 40.780018 4.075704

H 27.452818 39.214958 4.361727

H 33.740921 39.307858 1.461406

O 39.608543 19.193357 8.837862

C 39.804115 19.527784 10.209794

C 39.845676 21.036615 10.276777

C 39.956661 17.878630 8.455032

O 39.415909 21.547546 11.531856

C 39.340126 22.957615 11.396422

H 40.714012 19.071613 10.629784

H 38.961205 19.159132 10.814599

H 39.182690 21.395000 9.466623

H 40.861179 21.403801 10.037203

H 39.615879 17.745153 7.416549

H 39.462357 17.116880 9.074015

H 40.336559 23.414272 11.255122

H 38.901558 23.360491 12.316586

H 38.693272 23.267595 10.555467

H 41.048439 17.724619 8.487301

O 22.632965 14.493782 7.763688

C 22.116169 14.166689 6.482696

C 22.314184 15.344027 5.565956

C 22.297071 13.487079 8.699007

O 21.806189 15.070716 4.262963

C 22.176796 16.107244 3.371648

H 21.042973 13.914890 6.541368

H 22.638344 13.285641 6.067596

H 23.396183 15.568445 5.540043

H 21.820856 16.227520 6.010880

H 22.722441 13.778479 9.670018

H 22.714617 12.506779 8.416407

H 23.263195 16.304771 3.400494

H 21.639954 17.048429 3.586287

H 21.913263 15.790908 2.350766

H 21.203829 13.378536 8.816349

O 44.345280 34.482136 42.547493

C 44.629837 35.850647 42.337254

C 44.951061 36.515587 43.649536

C 43.849995 33.838707 41.390198

O 45.395496 37.839115 43.371334

C 45.988079 38.472279 44.493916

H 43.769325 36.371353 41.888416

H 45.488392 35.984119 41.652264

H 45.723080 35.928875 44.174644

H 44.057789 36.509232 44.300297

H 43.667625 32.790932 41.668888

H 44.569210 33.856796 40.553154

H 46.729294 37.839115 45.013157

H 45.236107 38.793011 45.234642

H 46.518078 39.359192 44.117439

H 42.893654 34.272877 41.048927

O 5.123477 5.984478 26.876860

C 6.062707 6.868949 27.469440

C 7.178930 7.000471 26.461271

C 3.793103 6.043639 27.357965

O 8.288307 7.810625 26.877350

C 9.484715 7.050342 26.796677

H 6.444560 6.448471 28.414051

H 5.624139 7.853651 27.696306

H 6.769697 7.425349 25.530354

H 7.497221 5.973722 26.214853

H 3.205412 5.356206 26.733604

H 3.348179 7.047409 27.279249

H 9.642638 6.641598 25.783129

H 9.487159 6.214764 27.519312

H 10.326159 7.718706 27.022562

H 3.692873 5.716057 28.398893

O 10.703612 22.064342 42.597851

C 10.957366 22.402681 43.952671

C 9.982443 21.672222 44.834206

C 11.796855 22.421259 41.773029

O 10.445457 21.732849 46.171425

C 9.633348 20.950077 47.024609

H 10.898205 23.495434 44.112061

H 11.968957 22.085365 44.260208

H 9.931595 20.626894 44.477779

H 8.970362 22.100523 44.740822

H 11.544568 22.116169 40.748238

H 12.724351 21.899084 42.065895

H 9.579566 19.894970 46.704357

H 8.608556 21.348551 47.096478

H 10.087563 20.979900 48.024952

H 11.989004 23.508146 41.780853

O 37.981396 38.655628 27.050432

C 36.984959 38.454185 26.065727

C 36.053551 39.630058 26.126846

C 38.978806 37.657722 26.992249

O 35.260998 39.670643 24.952440

C 34.451336 40.826954 24.937283

H 36.440781 37.505180 26.229031

H 37.424503 38.409203 25.054138

H 36.682312 40.536041 26.215828

H 35.431145 39.574318 27.039675

H 39.636902 37.809292 27.859118

H 39.586544 37.733505 26.073063

H 35.051739 41.749561 25.007200

H 33.708652 40.820599 25.754284

H 33.916935 40.835754 23.977518

H 38.550503 36.642712 27.050432

O 33.227058 5.588936 28.911289

C 32.420326 6.672889 29.323456

C 33.327774 7.766621 29.817274

C 32.481441 4.560720 28.293774

O 32.569450 8.953738 29.989866

C 33.364937 10.030357 30.447500

H 31.712358 6.368776 30.116499

H 31.828234 7.067943 28.478100

H 34.120819 7.908411 29.058949

H 33.826485 7.451263 30.753082

H 33.194786 3.768657 28.024862

H 31.984692 4.906881 27.370678

H 34.269939 10.177525 29.833410

H 33.668556 9.894436 31.498697

H 32.751820 10.941231 30.384430

H 31.722626 4.130953 28.969961

O 35.566090 24.720198 43.863686

C 35.219929 25.856468 43.094601

C 36.224674 26.015860 41.989624

C 34.781361 24.617035 45.036137

O 35.750416 26.990780 41.074352

C 36.767872 27.388279 40.175701

H 35.204285 26.776140 43.707226

H 34.216648 25.739126 42.648212

H 36.364998 25.042402 41.488472

H 37.199596 26.295525 42.430149

H 35.057114 23.676338 45.532398

H 33.701317 24.591610 44.817097

H 37.113544 26.548302 39.549873

H 37.641590 27.820492 40.696411

H 36.343483 28.166653 39.524452

H 34.975952 25.445768 45.737747

O 39.153843 1.547457 20.001556

C 39.769402 0.473282 19.310699

C 40.102852 0.879093 17.903076

C 38.881508 1.172449 21.336329

O 40.716946 48.658604 17.277248

C 41.550568 0.094363 16.184984

H 39.119617 48.471344 19.277943

H 40.711567 0.169169 19.802561

H 40.777084 1.747918 17.938768

H 39.193447 1.185161 17.355965

H 38.342709 2.008516 21.801298

H 39.806072 0.981767 21.909353

H 42.421837 0.696722 16.494965

H 41.014702 0.629739 15.383630

H 41.922642 48.035221 15.786017

H 38.241993 0.274289 21.395000

O 1.997760 37.144348 8.332312

C 1.905841 38.287460 7.482554

C 3.152119 38.341732 6.640131

C 1.381222 37.294941 9.601568

O 3.041621 39.354301 5.661297

C 4.198914 39.397820 4.850655

H 1.789477 39.222290 8.051666

H 1.022348 38.207767 6.827879

H 3.295375 37.348721 6.175161

H 4.015077 38.521660 7.304584

H 1.612484 36.384064 10.173124

H 0.284067 37.389301 9.540941

H 4.384217 38.438053 4.337280

H 5.100986 39.669662 5.426123

H 4.025344 40.170811 4.089883

H 1.773831 38.165230 10.154546

O 34.765713 22.110790 13.154119

C 35.863358 22.936590 12.800624

C 35.440434 23.958450 11.797343

C 35.172012 21.134401 14.089438

O 34.911903 23.315510 10.642985

C 35.055157 24.116373 9.484225

H 36.676445 22.331297 12.359121

H 36.281391 23.438719 13.691939

H 36.308281 24.589165 11.539679

H 34.676239 24.624369 12.231511

H 34.305634 20.486572 14.251273

H 35.468304 21.579815 15.055559

H 36.114666 24.222471 9.190379

H 34.617569 25.118677 9.613791

H 34.522717 23.608377 8.669183

H 36.007103 20.512484 13.719320

O 11.794410 48.791592 23.650425

C 11.751874 1.046306 22.818270

C 10.532976 0.941675 21.943579

C 12.923344 48.768124 24.500181

O 10.518797 2.014872 21.015102

C 9.250518 2.156173 20.405899

H 12.649056 1.136758 22.182175

H 11.700536 1.976247 23.409382

H 9.639215 0.953898 22.592873

H 10.544221 48.857109 21.425802

H 12.811380 47.886585 25.147036

H 12.985439 0.772506 25.139210

H 8.455521 2.359078 21.144180

H 8.964005 1.267301 19.816252

H 9.313590 3.020108 19.729712

H 13.869421 48.669357 23.938402

O 48.158428 12.815291 16.264679

C 46.742493 12.890097 16.341928

C 46.281925 11.945978 17.424416

C 48.671314 13.386359 15.078540

O 45.269356 12.568872 18.197901

C 44.885059 11.726449 19.265230

H 46.266769 12.667146 15.370917

H 46.442783 13.910490 16.625019

H 47.162971 11.710803 18.047798

H 45.922073 10.987190 17.012251

H 0.874201 13.345289 15.154813

H 48.363781 14.436577 14.958263

H 45.727478 11.516211 19.945328

H 44.478756 10.764238 18.906845

H 44.099838 12.246668 19.829943

H 48.360847 12.821159 14.182824

O 41.266502 46.146004 33.370312

C 42.540157 45.762684 32.877964

C 43.121006 46.889172 32.072208

C 40.718410 45.103607 34.154552

O 44.343815 46.464783 31.499674

C 44.945683 47.449486 30.686588

H 42.458508 44.869411 32.233555

H 43.237370 45.513329 33.698872

H 43.269638 47.758488 32.737152

H 42.390545 47.177639 31.295301

H 41.305126 44.920750 35.072269

H 40.641651 44.159000 33.590332

H 45.169125 48.377468 31.237608

H 44.325722 47.707150 29.810427

H 45.894691 47.023628 30.329182

H 39.705353 45.404301 34.451820

O 7.175018 39.323990 28.726475

C 6.913930 40.507683 27.991617

C 7.799379 41.597507 28.545082

C 6.455316 38.229771 28.193054

O 7.624343 42.883385 27.960327

C 6.455805 43.551262 28.401339

H 5.851001 40.792240 28.086960

H 7.119770 40.375675 26.912554

H 8.848130 41.324196 28.347067

H 7.679592 41.626842 29.642239

H 6.660177 37.360943 28.835018

H 6.771653 37.987751 27.163372

H 6.506165 43.804035 29.473070

H 5.535643 42.969437 28.223858

H 6.385889 44.481201 27.818537

H 5.365007 38.407249 28.185232

O 22.337652 42.089855 9.906170

C 21.907396 43.295063 9.280831

C 20.625427 43.829464 9.853366

C 23.629400 41.782806 9.414798

O 20.288555 44.984798 9.106284

C 19.052057 45.524086 9.525295

H 21.739695 43.131271 8.202256

H 22.664745 44.096416 9.373727

H 20.747660 44.064148 10.926563

H 19.828962 43.075535 9.761936

H 23.985340 40.882202 9.931595

H 24.355459 42.590027 9.616724

H 19.079437 45.853622 10.578935

H 18.219902 44.807808 9.402575

H 18.852573 46.397800 8.889689

H 23.616199 41.588703 8.328400

O 31.636574 31.576435 35.084007

C 30.545776 32.353832 35.542130

C 30.606892 32.397346 37.041676

C 31.725559 31.551991 33.675404

O 29.599213 33.266171 37.524734

C 29.608013 33.285240 38.937737

H 29.577209 31.930908 35.216995

H 30.596624 33.389870 35.161747

H 31.610661 32.747910 37.340408

H 30.490526 31.374022 37.442596

H 32.651588 31.017103 33.421654

H 31.777388 32.564560 33.240257

H 30.564846 33.662693 39.336700

H 29.412441 32.287338 39.367504

H 28.806660 33.961918 39.263363

H 30.877270 31.014658 33.223637

O 10.104186 17.572073 37.123814

C 10.126188 17.571095 38.538773

C 8.786036 17.138393 39.065834

C 11.399357 17.748087 36.586483

O 8.775768 17.344721 40.468082

C 7.626299 16.794188 41.079239

H 10.364296 18.575352 38.933334

H 10.893805 16.885128 38.926979

H 8.616867 16.077419 38.808659

H 7.989572 17.717773 38.566639

H 11.306949 17.723640 35.491772

H 12.084833 16.936954 36.889130

H 7.562249 15.701433 40.935005

H 6.691957 17.251335 40.709126

H 7.712350 16.997581 42.155861

H 11.837924 18.718121 36.872017

O 41.358418 32.750843 25.057070

C 41.620483 31.611151 25.854023

C 42.039497 30.481728 24.957329

C 41.022038 33.865108 25.858423

O 42.506420 29.417332 25.766994

C 42.874096 28.283506 25.007689

H 40.733570 31.310947 26.442694

H 42.436993 31.810146 26.570791

H 42.833027 30.848911 24.282120

H 41.186317 30.176147 24.323679

H 40.922783 34.731979 25.192993

H 41.802364 34.098328 26.604040

H 43.660290 28.510368 24.266962

H 42.011139 27.837116 24.484045

H 43.270126 27.541803 25.715658

H 40.057381 33.717941 26.372776

O 29.329811 42.884365 46.617329

C 30.728634 42.687817 46.459896

C 30.974567 42.129948 45.080139

C 29.049658 43.345913 47.925213

O 32.007671 41.167736 45.166191

C 32.303474 40.562935 43.924801

H 31.102175 41.948555 47.191818

H 31.292370 43.622646 46.643242

H 31.221966 42.918587 44.345768

H 30.038269 41.653728 44.747181

H 27.959347 43.431965 48.023975

H 29.488226 44.341370 48.105625

H 32.475574 41.304638 43.128826

H 31.506031 39.880878 43.589886

H 33.223145 39.978176 44.067570

H 29.417818 42.655548 48.705051

O 5.884737 18.348490 9.215804

C 5.017379 17.429794 8.578731

C 4.115307 18.224302 7.678614

C 6.743784 17.698215 10.131566

O 3.017175 17.434195 7.252758

C 2.219733 18.157318 6.336507

H 4.404263 16.889038 9.317989

H 5.579646 16.671955 8.001307

H 4.706910 18.598331 6.822501

H 3.768168 19.106329 8.249193

H 7.391613 18.464365 10.580891

H 7.393080 16.954067 9.639705

H 2.771244 18.387604 5.410477

H 1.843747 19.103884 6.760897

H 1.360198 17.523178 6.079819

H 6.188850 17.199999 10.945142

O 43.865154 46.992336 26.291124

C 44.202999 45.637028 26.540478

C 44.079304 45.365673 28.014107

C 44.003517 47.297424 24.917725

O 43.878353 43.977119 28.205276

C 43.898399 43.638290 29.577700

H 45.228287 45.402832 26.199696

H 43.521435 44.951553 26.003633

H 43.221725 45.941628 28.404760

H 44.984306 45.719170 28.539707

H 43.708206 48.346664 24.789627

H 43.354713 46.668179 24.282610

H 43.104870 44.151665 30.145344

H 44.869411 43.866619 30.051960

H 43.727276 42.554825 29.647125

H 45.046402 47.186440 24.571077

O 3.208345 23.552639 15.988923

C 3.247460 23.100380 14.639482

C 3.666471 21.653154 14.557831

C 2.689104 24.871279 15.993813

O 3.019619 21.059595 13.440630

C 3.543750 19.774693 13.161453

H 3.916313 23.736965 14.031256

H 2.243202 23.169809 14.186735

H 3.354535 21.145647 15.486794

H 4.765581 21.557325 14.483514

H 2.632877 25.207661 17.038162

H 1.676534 24.918705 15.556710

H 3.439120 19.084326 14.016588

H 4.608635 19.810385 12.871029

H 2.974149 19.371326 12.313163

H 3.328622 25.578270 15.434968

O 15.088806 44.904125 19.635349

C 15.619294 43.870045 20.440125

C 14.982221 43.912579 21.798367

C 15.666230 44.846432 18.347023

O 15.420300 42.774845 22.519045

C 14.870744 42.733772 23.821548

H 16.714493 43.969784 20.554533

H 15.419322 42.880451 19.989822

H 13.883110 43.915512 21.676134

H 15.262376 44.853764 22.309296

H 15.270199 45.683964 17.758842

H 15.413943 43.917465 17.819960

H 13.770658 42.637943 23.810305

H 15.140634 43.618244 24.426353

H 15.289267 41.847836 24.312433

H 16.763874 44.929550 18.377825

O 42.473175 45.321178 40.831356

C 41.096844 45.527508 40.538975

C 40.614273 46.846149 41.072884

C 42.785110 43.975651 40.519417

O 39.208603 46.904331 40.880737

C 38.631180 48.033752 41.502163

H 40.469059 44.753044 41.016171

H 40.909584 45.468838 39.450130

H 41.125687 47.685638 40.567822

H 40.871449 46.894062 42.145592

H 42.214531 43.266216 41.142799

H 43.850971 43.811371 40.712055

H 39.031124 0.088004 41.100266

H 38.775414 48.022018 42.596386

H 37.551624 47.995617 41.299259

H 42.591007 43.744877 39.458443

O 24.434666 46.609997 21.150047

C 24.940218 47.915920 20.938829

C 24.416574 48.793545 22.037451

C 24.799404 45.720634 20.113520

O 24.939238 1.212052 21.893707

C 24.369638 2.062298 22.865696

H 24.625835 48.322220 19.960485

H 26.044704 47.921787 20.963766

H 24.701620 48.351067 23.010908

H 23.311110 48.787682 21.989048

H 24.598455 44.705620 20.480705

H 25.869181 45.780773 19.854876

H 24.655172 1.769430 23.891466

H 23.267595 2.081856 22.805069

H 24.740246 3.078291 22.672081

H 24.201937 45.895672 19.202646

O 37.915874 14.000942 21.028305

C 38.006817 13.391249 19.737535

C 38.323643 11.921042 19.867100

C 38.366669 15.346472 20.993589

O 37.339432 11.177382 19.165977

C 37.813202 9.878790 18.861864

H 38.759766 13.889956 19.108774

H 37.052917 13.507614 19.201670

H 38.310440 11.643332 20.932474

H 39.339146 11.696625 19.492580

H 38.549038 15.656941 22.030117

H 37.618610 16.010435 20.530087

H 38.095802 9.314078 19.765404

H 38.688873 9.909103 18.189100

H 37.000603 9.344392 18.350935

H 39.316166 15.465281 20.449413

O 3.143318 31.077242 34.111038

C 3.031842 32.170971 35.006756

C 2.465175 33.349777 34.266518

C 3.403917 29.858343 34.775005

O 2.329253 34.427864 35.176903

C 1.551368 35.481506 34.647392

H 4.012632 32.438904 35.440434

H 2.351743 31.947044 35.848690

H 1.491230 33.048599 33.842617

H 3.123272 33.617222 33.418716

H 3.538861 29.089258 34.002007

H 2.562472 29.558632 35.419899

H 0.514841 35.164677 34.437153

H 1.986515 35.898560 33.724300

H 1.523988 36.272591 35.409630

H 4.319679 29.899414 35.386162

O 8.692651 9.635793 40.992214

C 9.341948 9.588856 42.260002

C 10.501684 8.643270 42.213066

C 7.313385 9.944306 41.123734

O 11.603239 9.259318 41.565235

C 12.807468 8.580687 41.895752

H 8.651092 9.258340 43.051086

H 9.698376 10.593114 42.547001

H 10.193660 7.723595 41.680622

H 10.761794 8.350401 43.246658

H 6.830813 9.730156 40.160545

H 7.154483 11.006746 41.366730

H 12.872497 7.581807 41.432735

H 12.918945 8.451120 42.982639

H 13.641091 9.197713 41.535412

H 6.815167 9.322879 41.887436

O 41.509987 46.594349 19.510183

C 42.613495 46.200275 18.689762

C 42.288849 45.090897 17.724619

C 41.870327 46.742493 20.875269

O 43.505302 44.663082 17.132036

C 43.315598 43.483299 16.379087

H 43.456898 45.840420 19.298477

H 42.959167 47.077412 18.123583

H 41.562302 45.427769 16.965311

H 41.820942 44.259716 18.266350

H 40.957497 47.017761 21.421402

H 42.624744 47.532600 21.022436

H 42.499577 43.582554 15.643251

H 43.097046 42.615452 17.023006

H 44.251896 43.288708 15.837844

H 42.261467 45.801796 21.299660

O 41.094398 29.925327 32.730793

C 42.228222 29.084858 32.838360

C 41.902107 27.999928 33.825016

C 41.330551 31.020037 31.870281

O 43.071625 27.245514 34.088551

C 42.896095 26.366419 35.180813

H 43.109760 29.640770 33.209454

H 42.502510 28.646782 31.861483

H 41.089508 27.362856 33.428009

H 41.518787 28.489346 34.739799

H 40.400120 31.602348 31.840456

H 41.572567 30.701744 30.840599

H 42.104034 25.621296 34.995510

H 42.659946 26.906687 36.113197

H 43.849995 25.839355 35.322113

H 42.139236 31.676178 32.237465

O 11.300104 33.686649 41.480160

C 12.259870 33.152252 42.373432

C 11.995359 31.688400 42.501045

C 11.544079 35.051739 41.211254

O 10.954921 31.477673 43.440273

C 10.558888 30.123344 43.453476

H 12.191420 33.619667 43.371334

H 13.281240 33.332664 41.998917

H 12.904276 31.140800 42.805157

H 11.710315 31.320728 41.498741

H 10.766194 35.388607 40.515018

H 12.526335 35.209171 40.741879

H 10.128143 29.803585 42.487843

H 9.790295 30.022621 44.229404

H 11.390555 29.450089 43.715054

H 11.489808 35.673164 42.118702

O 31.724094 48.108559 45.975368

C 30.873848 47.302807 46.771828

C 29.550320 47.116035 46.089287

C 32.971348 48.266972 46.624172

O 28.651182 46.558167 47.034386

C 27.448908 46.119114 46.440338

H 30.689522 47.768265 47.756531

H 31.323660 46.309795 46.958111

H 29.681839 46.465759 45.205303

H 29.185579 48.094379 45.726501

H 33.615265 48.873730 45.972923

H 33.471035 47.298893 46.803612

H 27.622477 45.264954 45.765614

H 26.944332 46.915573 45.870247

H 26.784941 45.794464 47.254398

H 32.871609 48.791100 47.587849

O 33.279373 42.779732 28.896132

C 34.287052 43.108780 27.936855

C 35.375408 43.990807 28.508904

C 33.194786 41.381886 29.147930

O 35.216995 45.311401 27.991617

C 36.392868 46.060440 28.236570

H 33.781013 43.670559 27.143326

H 34.691887 42.203285 27.461618

H 36.377708 43.590866 28.267370

H 35.300110 43.993252 29.611435

H 32.445751 41.232277 29.936573

H 34.153088 40.963856 29.498493

H 37.248978 45.706947 27.632254

H 36.685246 46.033062 29.296566

H 36.182137 47.100880 27.969124

H 32.871117 40.824020 28.251726

O 37.760887 48.374538 32.612965

C 36.360107 48.232746 32.767952

C 35.735748 48.143272 31.406290

C 38.439030 48.042553 33.807907

O 34.323723 48.190697 31.524612

C 33.682739 47.712036 30.359497

H 35.944519 0.196058 33.322399

H 36.098042 47.319427 33.331200

H 36.076042 47.197197 30.953054

H 36.109287 0.075292 30.774595

H 39.514183 48.115894 33.599133

H 38.219501 47.011406 34.135975

H 33.961426 46.667198 30.136055

H 33.894932 48.334442 29.476980

H 32.602695 47.743332 30.551643

H 38.192608 48.727543 34.633213

O 28.220434 41.545681 41.222008

C 27.145771 40.941853 41.919220

C 25.958652 40.931583 41.000523

C 29.313189 41.851257 42.062477

O 25.970388 39.716110 40.273487

C 24.989109 39.706821 39.258476

H 26.936512 41.489941 42.851604

H 27.392191 39.902878 42.204754

H 26.047150 41.807743 40.331181

H 25.001331 41.042572 41.538345

H 30.061737 42.358276 41.439579

H 29.764959 40.943317 42.493710

H 25.186636 40.465637 38.481079

H 23.971161 39.864254 39.654995

H 25.026758 38.714294 38.789593

H 29.036945 42.536736 42.880451

O 35.233616 24.394085 22.755198

C 35.880959 25.613470 22.436417

C 37.133102 25.766506 23.251461

C 34.073883 24.245449 21.960690

O 37.749153 27.002516 22.917032

C 38.952404 27.156527 23.648470

H 35.220905 26.475939 22.641768

H 36.156715 25.648186 21.365175

H 37.805382 24.914793 23.037798

H 36.873974 25.721525 24.326124

H 33.622112 23.276884 22.215422

H 34.312965 24.246918 20.883093

H 39.677975 26.352732 23.429430

H 38.777859 27.175594 24.739756

H 39.399284 28.116293 23.352179

H 33.332176 25.039957 22.153816

O 24.742201 6.603950 44.079304

C 24.227360 7.903521 43.853416

C 25.146545 8.914135 44.473869

C 23.864086 5.617783 43.574730

O 24.694284 10.202460 44.106197

C 25.464836 11.231165 44.690952

H 23.219679 8.022330 44.291988

H 24.146198 8.124027 42.772888

H 26.171827 8.732254 44.109127

H 25.153391 8.779680 45.571022

H 24.305099 4.636993 43.804035

H 23.735010 5.686721 42.481976

H 26.514565 11.209163 44.351635

H 25.438435 11.193028 45.793972

H 25.020889 12.186041 44.374615

H 22.866673 5.670098 44.045567

O 44.272430 37.248978 47.867519

C 43.976627 38.279152 48.820927

C 43.678383 37.737907 1.312281

C 43.204121 37.020161 46.957134

O 44.821495 37.875786 2.155195

C 44.780426 36.894505 3.178035

H 43.164516 38.921604 48.448853

H 44.876743 38.903023 48.882042

H 43.390892 36.680355 1.192984

H 42.816402 38.243462 1.784096

H 43.350803 36.033016 46.508297

H 43.207058 37.768711 46.152359

H 44.747669 35.873135 2.756578

H 43.908180 37.024075 3.841997

H 45.697655 36.987892 3.776479

H 42.217953 37.020649 47.447041

O 5.573779 0.678143 24.364748

C 4.802740 0.321225 25.495640

C 5.024224 47.745285 25.764061

C 5.455947 2.056921 24.086548

O 5.066272 47.420635 27.161417

C 3.840529 46.852505 27.621986

H 3.731498 0.544665 25.339672

H 5.125921 0.891314 26.378645

H 5.997680 47.457306 25.344072

H 4.269319 47.163952 25.221840

H 6.054884 2.254938 23.188387

H 5.843668 2.679327 24.907948

H 3.745188 45.788597 27.340853

H 2.957036 47.389347 27.239157

H 3.829773 46.920464 28.717186

H 4.415998 2.359076 23.878754

O 42.868229 35.950874 34.346214

C 42.084476 36.890106 33.628956

C 41.280190 37.670437 34.628815

C 43.745365 35.213573 33.520416

O 40.271534 38.418495 33.969738

C 39.695576 39.353813 34.863499

H 41.392155 36.390911 32.928810

H 42.713238 37.579494 33.036865

H 41.963223 38.321686 35.205750

H 40.830376 36.956600 35.336781

H 44.352127 34.585300 34.187801

H 44.426929 35.862869 32.944458

H 40.448036 40.024136 35.311848

H 39.148464 38.863907 35.684898

H 38.993473 39.973286 34.288033

H 43.203632 34.556454 32.820271

O 20.853268 45.065960 47.890495

C 20.930519 45.443901 0.356429

C 19.568365 45.918159 0.768106

C 22.083900 44.638634 47.349743

O 19.557608 46.145023 2.166440

C 18.240437 46.398777 2.613809

H 21.670755 46.247700 0.517775

H 21.212141 44.587299 0.995946

H 18.841330 45.136364 0.477683

H 19.306301 46.834412 0.206817

H 21.878550 44.361416 46.305882

H 22.490688 43.758080 47.875340

H 17.531492 45.615513 2.292094

H 17.863472 47.375656 2.265692

H 18.264883 46.396332 3.712430

H 22.845160 45.437546 47.352188

O 2.673458 7.509445 40.531639

C 2.148350 6.519855 39.657925

C 3.254794 5.528309 39.424709

C 1.687291 8.369469 41.065548

O 2.794224 4.386173 38.732876

C 3.797015 3.389249 38.710876

H 1.802678 6.956467 38.703049

H 1.286858 6.001591 40.109695

H 3.657670 5.252064 40.417233

H 4.061036 6.020170 38.852173

H 2.208488 9.057879 41.747116

H 0.924074 7.821870 41.645420

H 3.992586 2.989306 39.719532

H 4.748958 3.761323 38.292351

H 3.432275 2.570294 38.075268

H 1.176361 8.954227 40.282288

O 6.979936 28.320665 10.253309

C 7.835071 27.196133 10.258687

C 8.044332 26.764898 11.679023

C 6.578038 28.675137 8.948849

O 8.630069 25.475105 11.662889

C 8.787014 25.016979 12.989350

H 8.801682 27.422993 9.778071

H 7.384768 26.342951 9.718422

H 7.055231 26.747295 12.176263

H 8.670160 27.494865 12.225645

H 5.812376 29.450577 9.071081

H 6.123334 27.830271 8.402227

H 7.852184 25.100096 13.563841

H 9.570765 25.577780 13.524237

H 9.068148 23.957472 12.950725

H 7.412148 29.083393 8.352845

O 9.283765 42.689770 39.053612

C 9.392796 42.168571 40.366383

C 8.009130 41.907486 40.879757

C 10.551555 42.872143 38.468365

O 8.090780 41.193161 42.097679

C 6.809300 40.755569 42.502510

H 9.923772 42.862850 41.039639

H 9.952129 41.216141 40.380566

H 7.477176 41.323215 40.108719

H 7.466908 42.861870 41.007370

H 10.376518 43.226124 37.444061

H 11.126045 41.932907 38.425339

H 6.338462 40.090141 41.758850

H 6.124312 41.598972 42.698082

H 6.939844 40.182056 43.429520

H 11.152937 43.620689 39.011078

O 20.228907 38.519703 41.644932

C 19.178200 39.444756 41.429802

C 18.115759 39.274609 42.479530

C 21.189650 38.601357 40.609871

O 16.960911 39.970352 42.039497

C 15.826110 39.655972 42.822758

H 18.700029 39.264832 40.453903

H 19.536585 40.487640 41.414646

H 18.452143 39.640327 43.465210

H 17.918232 38.191631 42.581718

H 21.951399 37.833740 40.806419

H 21.684935 39.587032 40.577110

H 15.893582 40.072048 43.841194

H 15.659385 38.567619 42.898540

H 14.952395 40.099430 42.325031

H 20.751572 38.400402 39.618324

O 16.100887 11.919576 23.545794

C 16.579060 11.163692 24.643927

C 15.984522 11.772408 25.883848

C 16.384466 11.343129 22.289249

O 16.660709 11.304994 27.036741

C 16.011414 11.768007 28.204790

H 17.681103 11.197918 24.700644

H 16.290592 10.099297 24.574497

H 14.907904 11.522566 25.921007

H 16.059818 12.872006 25.796820

H 15.966433 12.017850 21.527988

H 15.906783 10.357450 22.167507

H 14.978309 11.385178 28.283018

H 15.983056 12.870541 28.260529

H 16.580526 11.391533 29.065304

H 17.467442 11.237521 22.110302

O 37.663589 30.091560 44.627392

C 39.011566 30.445059 44.380486

C 39.390484 31.653200 45.187214

C 37.314495 28.906403 43.944359

O 40.626984 32.098122 44.663082

C 41.228851 33.157143 45.382298

H 39.168510 30.698322 43.316086

H 39.704865 29.618771 44.622501

H 39.456978 31.403357 46.261879

H 38.604286 32.419838 45.071827

H 36.259388 28.708876 44.172199

H 37.911964 28.040508 44.280743

H 41.583324 32.840805 46.376774

H 40.562443 34.022057 45.511864

H 42.098167 33.486187 44.794117

H 37.423527 29.006142 42.851604

O 38.519215 19.153265 23.155140

C 37.513000 18.379293 22.528336

C 37.069542 17.344721 23.537483

C 39.104462 20.097385 22.278004

O 36.194359 16.354643 22.987438

C 35.205750 15.966922 23.931068

H 37.901211 17.899654 21.615505

H 36.659332 19.011477 22.222755

H 36.587460 17.889387 24.364748

H 37.966728 16.860682 23.962851

H 39.832966 20.679699 22.862272

H 38.365204 20.799486 21.854103

H 34.639080 16.835258 24.307055

H 35.636982 15.425677 24.790117

H 34.493870 15.304914 23.421606

H 39.637882 19.609924 21.443892

O 15.419811 7.550515 2.693015

C 16.032927 8.742033 2.183552

C 15.938563 9.871945 3.167765

C 16.360020 6.512521 2.945791

O 16.670979 10.979856 2.682748

C 16.460249 12.050119 3.579442

H 15.513685 9.011921 1.250678

H 17.090967 8.585576 1.925398

H 16.340952 9.534585 4.141220

H 14.872701 10.131077 3.318354

H 15.788463 5.602137 3.168742

H 17.005405 6.751606 3.807282

H 16.882195 11.829613 4.574899

H 15.388030 12.282848 3.701674

H 16.950155 12.941924 3.168742

H 17.004427 6.297882 2.077455

O 47.978504 20.623960 25.564089

C 0.495284 20.717344 25.696587

C 0.893272 22.133282 25.399321

C 47.575626 19.270609 25.508841

O 2.301384 22.233023 25.351894

C 2.728707 23.512058 24.930927

H 1.019904 20.060715 24.978354

H 0.819932 20.416656 26.710136

H 0.465459 22.808491 26.158136

H 0.442969 22.398281 24.427332

H 46.477497 19.247139 25.492704

H 47.927166 18.697094 26.381577

H 2.450018 24.301678 25.649164

H 2.330231 23.779991 23.936937

H 3.824884 23.471966 24.863457

H 47.943790 18.776791 24.595034

O 38.444897 24.647348 33.202614

C 38.052288 25.887272 33.764881

C 38.505035 25.928829 35.195972

C 38.244434 24.637081 31.802320

O 37.910011 27.039675 35.844292

C 38.563705 27.331076 37.062698

H 36.955135 26.013905 33.724300

H 38.502590 26.741428 33.226566

H 39.609524 25.980165 35.213085

H 38.213634 24.989599 35.695656

H 38.577396 23.657759 31.429758

H 38.837017 25.416922 31.289436

H 39.608059 27.653280 36.901840

H 38.562241 26.470562 37.752575

H 38.017086 28.160786 37.532558

H 37.183464 24.771536 31.526567

O 9.030989 45.088451 21.590572

C 10.105653 44.865501 20.698277

C 9.527740 44.730556 19.317545

C 9.414798 45.623825 22.839294

O 10.551555 44.445023 18.377825

C 10.054316 44.640106 17.066031

H 10.667431 43.957069 20.976479

H 10.811665 45.710857 20.700722

H 9.028055 45.686897 19.079437

H 8.746922 43.945824 19.316566

H 8.506370 45.647293 23.459253

H 9.804962 46.651066 22.741018

H 9.748246 45.686409 16.886595

H 9.192335 43.984451 16.851393

H 10.862024 44.397594 16.360508

H 10.173614 45.012177 23.351690

O 46.172890 6.756985 8.816838

C 46.756672 5.486750 9.061791

C 48.242523 5.602137 8.802171

C 44.803406 6.795121 9.157132

O 48.648335 4.523562 7.977838

C 1.064396 4.726467 7.483532

H 46.341084 4.744557 8.363602

H 46.525898 5.121521 10.078761

H 48.850262 5.629028 9.723800

H 48.405827 6.566792 8.289774

H 44.435242 7.794490 8.891155

H 44.647926 6.666045 10.239130

H 1.824190 4.642371 8.277551

H 1.178314 5.708723 6.994115

H 1.247743 3.951516 6.727160

H 44.209846 6.039728 8.613444

O 2.344410 29.476490 16.783920

C 1.798277 29.562054 15.471637

C 1.829080 30.993635 15.015956

C 3.072423 28.281061 17.004915

O 1.482430 31.042038 13.642557

C 1.900952 32.258492 13.056822

H 0.773973 29.154287 15.455014

H 2.392325 28.987074 14.740690

H 2.854362 31.367664 15.189526

H 1.148492 31.620930 15.617826

H 3.645936 28.425297 17.930456

H 3.794081 28.065447 16.198183

H 3.000062 32.322052 12.983972

H 1.537678 33.133183 13.620067

H 1.486341 32.292717 12.040340

H 2.398192 27.419573 17.118347

O 4.246829 25.109386 3.496324

C 4.863367 25.256065 2.229512

C 3.838085 24.988132 1.161693

C 5.177258 25.302513 4.544097

O 4.403775 25.304468 48.795990

C 3.531038 25.027245 47.720840

H 5.717035 24.562275 2.108258

H 5.254509 26.281836 2.092612

H 2.947747 25.610048 1.368021

H 3.522237 23.929604 1.214497

H 4.619881 25.267309 5.491150

H 5.684277 26.280857 4.483470

H 2.613809 25.638895 47.765331

H 3.247460 23.960894 47.675858

H 4.072281 25.277578 46.796764

H 5.941453 24.506538 4.563165

O 11.787076 44.063656 9.025611

C 11.668266 42.976772 9.922305

C 11.543591 43.525837 11.312816

C 11.924465 43.625092 7.692305

O 11.035105 42.506912 12.152794

C 11.127512 42.893166 13.507124

H 12.538070 42.296673 9.861678

H 10.763261 42.379791 9.708643

H 10.859091 44.393684 11.291303

H 12.527802 43.889111 11.660443

H 11.886329 44.517384 7.054253

H 11.109911 42.946457 7.385257

H 10.549110 43.806480 13.725676

H 12.173329 43.067711 13.803905

H 10.726591 42.065411 14.106551

H 12.891564 43.121006 7.531935

O 12.342009 26.945799 12.555671

C 12.452996 25.806107 13.392715

C 12.172840 24.568632 12.585495

C 12.239334 28.138296 13.310576

O 11.989982 23.484190 13.476322

C 11.508876 22.330318 12.818225

H 11.713737 25.835445 14.214114

H 13.452854 25.751839 13.857687

H 12.989350 24.362793 11.871660

H 11.261968 24.759314 11.992914

H 12.170885 28.964094 12.590873

H 13.118916 28.307465 13.955960

H 12.160617 22.024740 11.987536

H 10.487017 22.456951 12.422194

H 11.497142 21.522121 13.561884

H 11.331395 28.157364 13.941293

O 14.162289 32.445263 46.074619

C 15.043825 32.301517 44.973553

C 15.236464 33.635803 44.308609

C 14.099217 31.257166 46.835392

O 15.637384 33.413338 42.968460

C 15.748371 34.643482 42.285427

H 14.624325 31.610174 44.223049

H 16.016304 31.885437 45.286953

H 15.978656 34.252338 44.849854

H 14.274252 34.175579 44.347725

H 13.453343 31.462029 47.699814

H 15.090762 30.954519 47.214310

H 16.528700 35.290333 42.724483

H 14.795450 35.202328 42.273689

H 16.022169 34.413685 41.247433

H 13.667004 30.419632 46.261879

O 1.476074 46.708271 18.000374

C 1.030660 46.030125 19.164021

C 1.593905 44.639126 19.123440

C 0.896205 47.987793 17.873741

O 1.267790 43.947781 20.312513

C 1.920020 42.695637 20.352606

H 48.820438 45.980743 19.215359

H 1.377799 46.532745 20.085651

H 2.686659 44.721756 18.995831

H 1.197385 44.111572 18.237015

H 1.238944 48.406319 16.917887

H 1.205696 48.662514 18.687805

H 3.019619 42.795868 20.390253

H 1.650621 42.060520 19.492092

H 1.593905 42.186665 21.267389

H 48.688427 47.945744 17.850273

O 8.485345 44.639614 9.806918

C 8.443297 45.404785 8.616378

C 9.497915 46.469181 8.678472

C 7.488421 43.640247 9.768292

O 9.631882 47.051006 7.393569

C 10.802864 47.838669 7.319741

H 7.449796 45.862911 8.467255

H 8.651092 44.776024 7.730440

H 10.437634 45.996391 9.009476

H 9.237317 47.233376 9.430932

H 7.622876 42.998772 10.649341

H 7.565183 43.003662 8.869642

H 11.711292 47.216263 7.352499

H 10.871803 48.571087 8.138206

H 10.772061 48.385292 6.366820

H 6.479763 44.085659 9.804962

O 39.207623 44.471912 26.211918

C 40.441189 45.145164 26.002659

C 40.250996 46.623196 26.112179

C 39.394886 43.072109 26.113644

O 39.402706 47.090122 25.071739

C 39.418842 48.500191 24.979332

H 40.857758 44.915367 25.006222

H 41.199028 44.813675 26.736540

H 41.239609 47.115055 26.065241

H 39.817318 46.857391 27.099813

H 38.406761 42.601273 26.205563

H 40.046139 42.681946 26.915485

H 39.052635 0.085562 25.901449

H 38.749989 48.776432 24.152065

H 40.427502 48.889866 24.755892

H 39.831009 42.774845 25.143610

O 31.142759 1.768942 36.910152

C 30.476837 0.577421 37.362900

C 30.154144 48.499702 36.250099

C 30.525730 2.933081 37.445042

O 29.499470 47.379078 36.819702

C 29.249630 46.374821 35.855045

H 29.524893 0.806731 37.869431

H 31.116356 0.081651 38.111446

H 31.074797 48.183853 35.735748

H 29.511696 0.102185 35.500084

H 31.078707 3.806304 37.076385

H 30.550665 2.944814 38.548550

H 30.173702 46.009594 35.373451

H 28.553885 46.719025 35.070316

H 28.788080 45.526043 36.379665

H 29.473557 3.027928 37.121368

O 18.659937 9.064236 22.818758

C 19.859278 8.841285 22.098568

C 20.917807 9.759980 22.637856

C 17.656166 8.139185 22.452063

O 22.160662 9.413820 22.049675

C 23.243637 10.172636 22.548382

H 19.719933 9.011432 21.014614

H 20.219616 7.804758 22.221777

H 20.932964 9.646549 23.734032

H 20.642052 10.809710 22.436417

H 16.753607 8.391960 23.025574

H 17.938280 7.102657 22.701904

H 23.498857 9.896392 23.582952

H 23.054911 11.257078 22.503889

H 24.110996 9.949196 21.913752

H 17.410725 8.194922 21.377398

O 10.586268 32.482910 34.035744

C 10.891360 33.777103 33.552681

C 9.599612 34.400974 33.097980

C 11.669245 31.699158 34.495335

O 9.869500 35.480038 32.213997

C 8.708297 35.763615 31.456648

H 11.546035 33.724785 32.667725

H 11.397400 34.399017 34.312965

H 8.979651 34.739315 33.948227

H 9.034901 33.602062 32.585094

H 11.230676 30.752106 34.837585

H 12.220756 32.160706 35.328960

H 7.866363 36.078976 32.095676

H 8.387072 34.895279 30.853800

H 8.951294 36.577682 30.763838

H 12.388947 31.459093 33.701317

O 11.394956 12.771288 7.744131

C 12.381612 13.506146 8.450631

C 11.728404 14.543163 9.321412

C 11.930820 11.569992 7.224889

O 12.716528 15.124498 10.158457

C 12.119547 15.885760 11.189607

H 13.094959 13.979918 7.752932

H 12.960014 12.850006 9.125352

H 10.948075 14.032723 9.915460

H 11.224320 15.310780 8.707319

H 11.091821 10.993057 6.813700

H 12.409481 10.954921 8.007663

H 11.484429 15.266288 11.847215

H 11.506920 16.714005 10.794064

H 12.934100 16.307217 11.794899

H 12.665191 11.756763 6.423536

O 37.495399 16.259790 35.082050

C 37.990196 16.337040 33.758522

C 38.453697 17.750042 33.519928

C 37.156570 14.940661 35.455101

O 38.886890 17.953924 32.172440

C 38.063534 18.842306 31.434647

H 37.208889 16.076441 33.021217

H 38.839951 15.649118 33.595707

H 39.308342 17.969082 34.179001

H 37.647453 18.443342 33.803993

H 36.815300 14.982709 36.498474

H 38.021976 14.259584 35.410610

H 38.083088 19.865145 31.849258

H 37.019672 18.490278 31.389666

H 38.467876 18.879955 30.413765

H 36.345440 14.522139 34.834652

O 10.951987 22.592873 24.846830

C 10.051381 23.229458 23.953560

C 8.787502 23.577085 24.687443

C 12.148883 22.214443 24.198023

O 7.909877 24.242027 23.793192

C 6.706625 24.598455 24.441021

H 9.788339 22.574295 23.102337

H 10.484083 24.156954 23.534060

H 9.041745 24.216116 25.553331

H 8.336222 22.649099 25.084938

H 12.759066 21.678089 24.937773

H 12.720440 23.081802 23.830839

H 6.892418 25.253620 25.305933

H 6.142891 23.715942 24.786692

H 6.089598 25.145567 23.714964

H 11.963579 21.542168 23.344357

O 37.706615 19.389418 16.979980

C 37.434284 19.631926 18.353380

C 36.187027 20.473372 18.471210

C 39.068768 19.081882 16.746761

O 35.496174 20.089563 19.649529

C 34.367237 20.908028 19.875902

H 38.284527 20.119875 18.858931

H 37.262180 18.672649 18.869198

H 35.566090 20.297846 17.575005

H 36.431980 21.552435 18.476099

H 39.172909 18.900000 15.669164

H 39.397820 18.181767 17.291428

H 33.619175 20.819044 19.069658

H 34.643482 21.970957 19.987865

H 33.906670 20.573112 20.815620

H 39.736156 19.918438 17.020561

O 41.672310 0.135433 43.150829

C 42.370010 1.058040 42.324051

C 43.389427 1.919531 43.009041

C 40.696899 0.748060 43.970272

O 44.430351 1.108889 43.528282

C 45.488392 1.922465 43.987873

H 41.663021 1.676045 41.742718

H 42.908321 0.436124 41.594082

H 42.926899 2.522379 43.810883

H 43.782524 2.633366 42.261959

H 41.144268 1.417402 44.725178

H 40.178146 48.826794 44.492935

H 45.176949 2.584473 44.814163

H 45.903492 2.539492 43.174789

H 46.288280 1.262412 44.350170

H 39.952263 1.316194 43.387470

O 34.818520 39.633480 8.582153

C 35.725479 39.289764 7.546114

C 36.084839 37.834713 7.662968

C 34.446934 40.990746 8.444276

O 36.855392 37.410816 6.538434

C 37.981396 36.596748 6.840591

H 36.647106 39.895546 7.600874

H 35.280067 39.463825 6.548701

H 35.155392 37.249470 7.698660

H 36.616306 37.671413 8.609533

H 33.720387 41.223476 9.232428

H 33.967293 41.197071 7.472775

H 37.683147 35.584671 7.158884

H 38.630688 37.037762 7.617009

H 38.571529 36.516079 5.915540

H 35.312332 41.667423 8.551839

O 12.542959 45.963142 48.887424

C 11.189117 45.860466 0.402388

C 11.157825 45.826733 1.902419

C 12.686704 46.092220 47.486156

O 9.809362 45.847755 2.337565

C 9.699353 46.022305 3.736388

H 10.707034 44.952038 48.887424

H 10.593603 46.725872 0.063561

H 11.717649 46.706802 2.264225

H 11.690757 44.929058 2.267648

H 13.741322 45.895672 47.250004

H 12.428550 47.105770 47.139503

H 10.070450 47.009449 4.061036

H 10.234730 45.240509 4.302567

H 8.630069 45.960209 3.983785

H 12.072610 45.369095 46.929264

O 16.160536 44.867947 13.274395

C 15.821221 44.064148 12.156217

C 15.515640 42.684391 12.664213

C 16.298903 46.234989 12.947302

O 15.056049 41.851746 11.613506

C 14.614546 40.619160 12.151816

H 16.639196 44.033833 11.413535

H 14.922083 44.444042 11.641376

H 14.742156 42.787556 13.440142

H 16.413801 42.260979 13.151185

H 16.540434 46.763515 13.880666

H 15.364562 46.660843 12.543937

H 13.711985 40.741394 12.776666

H 15.394876 40.119473 12.752220

H 14.364704 39.962040 11.310371

H 17.111502 46.413933 12.222222

O 42.353874 18.187632 35.121651

C 42.822758 18.968939 34.037212

C 41.806274 18.967962 32.925880

C 43.265236 18.257549 36.200718

O 42.385170 19.526318 31.750008

C 41.452293 20.235264 30.954029

H 43.782524 18.578775 33.652424

H 42.996819 20.018179 34.339371

H 40.927673 19.547829 33.244659

H 41.455227 17.940723 32.727371

H 42.875072 17.616564 37.004517

H 43.358624 19.284788 36.593815

H 41.119823 21.157381 31.459093

H 40.567333 19.630947 30.698322

H 41.955402 20.517374 30.018225

H 44.271938 17.897697 35.919094

O 45.502575 21.908863 5.723880

C 44.563343 21.426292 4.781716

C 45.262020 20.469460 3.858131

C 44.870388 22.675991 6.728138

O 44.312035 19.947773 2.947258

C 44.859146 18.961605 2.097012

H 44.122818 22.253069 4.195002

H 43.729721 20.896782 5.276999

H 45.724056 19.671040 4.468313

H 46.080486 21.001413 3.338889

H 45.656586 23.061266 7.389169

H 44.177578 22.069721 7.335876

H 45.232197 18.087402 2.659279

H 45.674675 19.360571 1.470695

H 44.046059 18.625223 1.438915

H 44.315456 23.534060 6.313528

O 25.583647 10.676721 2.367878

C 26.278412 9.455379 2.560516

C 27.321785 9.353682 1.485363

C 24.581833 10.867891 3.347201

O 28.243416 8.329866 1.811967

C 29.188511 8.093225 0.787174

H 25.597338 8.586064 2.520424

H 26.783476 9.431910 3.542772

H 27.825380 10.333493 1.434515

H 26.834812 9.177178 0.508974

H 24.098772 11.832057 3.131095

H 24.999376 10.908473 4.368572

H 29.784027 8.987474 0.541243

H 28.713762 7.724084 48.754433

H 29.872034 7.317297 1.162182

H 23.810793 10.078273 3.313954

O 10.040136 1.532789 3.067534

C 9.540452 2.693993 2.430461

C 8.906801 3.560863 3.478723

C 10.679654 0.661520 2.161062

O 8.566018 4.811051 2.905210

C 7.914766 5.613382 3.870843

H 8.796793 2.445618 1.650621

H 10.351095 3.268484 1.945445

H 9.623569 3.683095 4.312345

H 8.018419 3.046021 3.890400

H 11.146091 48.761768 2.760488

H 11.471718 1.166093 1.584127

H 8.567485 5.847090 4.730867

H 6.998516 5.128855 4.251229

H 7.630210 6.557014 3.384848

H 9.962886 0.200460 1.459450

O 6.126757 37.151680 35.752369

C 5.893538 37.743286 34.487514

C 6.481719 39.139664 34.475292

C 5.516574 35.873135 35.809574

O 6.292015 39.856922 33.252480

C 7.190664 39.441330 32.234043

H 4.810073 37.796577 34.275318

H 6.353620 37.128212 33.692516

H 7.548560 39.085880 34.747623

H 5.985456 39.742023 35.254154

H 5.696500 35.459015 36.810898

H 5.933141 35.167614 35.066895

H 8.196877 39.870125 32.356766

H 6.776542 39.809986 31.290901

H 7.280627 38.343689 32.173416

H 4.424798 35.932297 35.657520

O 1.859393 45.825264 32.748398

C 1.603195 46.709736 33.830883

C 2.906188 46.949310 34.545696

C 0.731436 45.707432 31.900595

O 2.829915 47.628429 35.792953

C 2.414326 0.081162 35.658985

H 0.871270 46.272633 34.535431

H 1.188095 47.668526 33.475433

H 3.613667 47.446552 33.858753

H 3.326177 45.961185 34.770115

H 0.930430 44.887501 31.196053

H 0.547599 46.631996 31.326595

H 1.365087 0.162323 35.323090

H 3.054822 0.645877 34.961773

H 2.490599 0.536354 36.656399

H 48.705540 45.468838 32.458462

O 8.506370 41.654709 2.280849

C 7.248357 41.180450 2.724307

C 6.778986 40.095028 1.803167

C 8.830528 42.884365 2.896409

O 5.410966 39.872566 2.079411

C 4.785627 39.032589 1.135291

H 6.488074 41.975933 2.685682

H 7.284050 40.826466 3.769635

H 7.373523 39.171444 1.926865

H 6.930065 40.458302 0.772017

H 9.806918 43.193855 2.498422

H 8.908268 42.798313 3.994053

H 5.298512 38.061577 1.042883

H 4.731845 39.505383 0.140322

H 3.759856 38.857063 1.492697

H 8.096159 43.674473 2.656835

O 34.910927 4.151487 38.984184

C 33.723808 4.520628 39.661839

C 33.073044 3.280218 40.202106

C 35.758240 5.257932 38.743145

O 31.860992 3.638602 40.840645

C 31.344196 2.532158 41.554482

H 33.027573 5.047692 38.985161

H 33.930622 5.191437 40.514530

H 33.776123 2.801069 40.908115

H 32.898987 2.559049 39.383148

H 36.670090 4.867767 38.269859

H 36.054039 5.771795 39.674553

H 32.028694 2.198220 42.354855

H 31.125156 1.671156 40.897362

H 30.408875 2.857295 42.024342

H 35.295712 5.994257 38.065975

O 9.557565 38.151539 3.177543

C 9.969241 38.371067 4.526006

C 9.215315 39.474579 5.221262

C 10.290468 38.898621 2.223155

O 9.613791 39.493156 6.579504

C 8.890178 40.448036 7.330009

H 11.054662 38.540237 4.606191

H 9.757536 37.436729 5.066761

H 8.134295 39.267273 5.122010

H 9.410398 40.444614 4.733312

H 9.804473 38.734833 1.251656

H 11.337750 38.560284 2.149328

H 7.801824 40.270554 7.293828

H 9.088683 41.480652 6.993626

H 9.219715 40.351227 8.374847

H 10.280689 39.978664 2.437795

O 1.943489 11.770942 20.459681

C 2.662213 10.979856 19.530230

C 1.953756 11.013592 18.207678

C 2.605986 11.809078 21.706936

O 2.695460 10.234730 17.287027

C 2.053987 10.195615 16.029505

H 2.744353 9.929150 19.866611

H 3.691406 11.357308 19.387951

H 1.870639 12.062832 17.868851

H 0.926030 10.624894 18.336267

H 2.016339 12.462775 22.364056

H 3.625890 12.226133 21.627729

H 1.987981 11.191562 15.561601

H 1.042883 9.767315 16.103333

H 2.649012 9.551698 15.370430

H 2.668569 10.809710 22.169462

O 18.099136 5.526842 37.029449

C 18.708830 5.849535 38.272305

C 19.082371 4.562187 38.966095

C 17.661057 6.713470 36.393356

O 18.593931 4.634059 40.292557

C 18.657982 3.382893 40.942341

H 17.990105 6.377088 38.926979

H 19.562498 6.538434 38.129539

H 20.163879 4.340214 38.959248

H 18.592953 3.741277 38.417030

H 17.103678 6.422069 35.492264

H 18.509346 7.349076 36.090710

H 19.694019 3.039176 41.091953

H 18.105982 2.602564 40.394253

H 18.176388 3.504636 41.922153

H 16.988781 7.309474 37.035805

O 40.999054 37.650391 11.339218

C 40.476883 36.337128 11.269301

C 40.343403 35.835487 12.678392

C 41.311481 38.190655 10.069962

O 39.855942 34.509518 12.651989

C 39.709751 33.932579 13.932003

H 41.137913 35.656540 10.707034

H 39.494625 36.315617 10.764238

H 39.663795 36.511677 13.226969

H 41.335926 35.896114 13.161942

H 41.722183 39.196869 10.243041

H 40.423100 38.282574 9.421642

H 38.971470 34.468445 14.552453

H 40.666100 33.876354 14.478624

H 39.346481 32.907299 13.774080

H 42.073723 37.598072 9.535074

O 23.315020 9.452445 7.984684

C 24.502138 9.132197 7.278671

C 24.153042 8.180743 6.167337

C 23.557039 9.983420 9.273008

O 25.349939 7.739730 5.552266

C 25.138233 6.675334 4.647749

H 25.242865 8.634957 7.927478

H 24.981777 10.042092 6.875305

H 23.486633 8.675050 5.438346

H 23.587841 7.340276 6.611285

H 22.578205 10.277755 9.679796

H 24.212692 10.870825 9.249540

H 24.443954 6.945222 3.833195

H 24.755402 5.770817 5.151834

H 26.117556 6.438693 4.207225

H 24.002453 9.234383 9.950173

O 7.892276 16.225075 19.866123

C 6.779476 15.958611 19.032501

C 5.661297 16.885618 19.409952

C 9.025611 15.474082 19.485735

O 4.526495 16.582481 18.617401

C 3.454765 17.441528 18.943516

H 6.432826 14.916215 19.147886

H 7.022473 16.114578 17.964682

H 5.994746 17.929968 19.257896

H 5.446658 16.763874 20.487061

H 9.848966 15.794331 20.138454

H 9.319457 15.663298 18.437963

H 3.714875 18.505436 18.800259

H 3.115938 17.299250 19.984444

H 2.626032 17.193642 18.266350

H 8.868176 14.390617 19.618725

O 6.443093 36.867126 5.659831

C 7.069899 36.540524 6.887529

C 6.501765 37.418148 7.963659

C 6.958424 36.082397 4.604235

O 7.365211 37.375610 9.083304

C 6.935932 38.265949 10.090984

H 8.159719 36.724358 6.842547

H 6.924687 35.476128 7.146661

H 5.481371 37.087631 8.233059

H 6.422558 38.441963 7.556871

H 6.482207 36.431492 3.678694

H 6.734983 35.009201 4.735268

H 5.895983 38.067448 10.401454

H 7.019051 39.319588 9.775137

H 7.591096 38.112915 10.959810

H 8.048244 36.200230 4.492270

O 41.358910 13.019175 44.756470

C 42.491264 12.557138 44.038723

C 43.167454 13.753056 43.418762

C 40.763882 11.988026 45.517731

O 42.932766 13.744744 42.020916

C 43.546371 14.848253 41.381886

H 42.189110 11.861393 43.237858

H 43.173809 12.000249 44.705620

H 44.252384 13.780924 43.630955

H 42.733772 14.652683 43.883732

H 39.966442 12.447618 46.118622

H 41.485050 11.511321 46.202721

H 44.611748 14.946039 41.652264

H 43.034466 15.796774 41.618038

H 43.481834 14.675662 40.298424

H 40.325314 11.204762 44.879677

O 28.363203 47.681236 2.614787

C 28.774879 46.590439 1.813434

C 30.264154 46.664753 1.654043

C 27.044561 47.536022 3.091981

O 30.649918 45.695210 0.703078

C 32.053631 45.616982 0.576935

H 28.301109 46.629551 0.815532

H 28.513304 45.616493 2.264714

H 30.747215 46.488255 2.631899

H 30.536976 47.684658 1.327439

H 26.804010 48.454231 3.641047

H 26.962425 46.680401 3.778435

H 32.537182 45.258110 1.501987

H 32.510288 46.579681 0.290423

H 32.251156 44.897770 48.666424

H 26.308237 47.405972 2.283783

O 39.517117 42.507401 41.035725

C 38.476189 41.656666 41.497272

C 38.143227 41.899174 42.947922

C 39.916569 42.102566 39.734200

O 36.962955 41.188271 43.297020

C 36.865662 41.028393 44.699753

H 38.759766 40.600578 41.384823

H 37.573627 41.815079 40.887093

H 38.024422 42.980682 43.137138

H 38.972450 41.548122 43.573261

H 40.482258 42.925922 39.279499

H 39.060459 41.880596 39.077080

H 36.858326 41.997448 45.228771

H 37.690971 40.413319 45.103119

H 35.919586 40.507683 44.899727

H 40.561954 41.210274 39.765980

O 35.115295 31.667868 17.704082

C 34.789181 32.955704 18.191545

C 35.865803 33.911068 17.770088

C 34.079750 30.728634 17.906988

O 35.576355 35.184235 18.318176

C 36.595284 36.109287 18.002329

H 34.733932 32.968414 19.294565

H 33.817192 33.312618 17.802357

H 35.914207 33.944805 16.665600

H 36.836811 33.522369 18.130426

H 34.420040 29.776203 17.477709

H 33.142960 31.022482 17.401436

H 36.705292 36.247166 16.912508

H 37.571182 35.810551 18.423784

H 36.311214 37.073944 18.443830

H 33.869511 30.560934 18.974808

O 24.250340 38.160831 12.201198

C 25.662363 38.219013 12.058920

C 26.091642 39.663307 11.955267

C 23.874844 36.922375 12.776666

O 27.377033 39.794338 12.545892

C 27.748619 41.156490 12.646123

H 25.995323 37.601009 11.205252

H 26.157160 37.805382 12.952681

H 25.346518 40.261753 12.511667

H 26.090176 40.033424 10.913363

H 22.787956 36.941933 12.930679

H 24.347635 36.762009 13.758924

H 27.026474 41.740761 13.244082

H 27.847872 41.635643 11.656532

H 28.724518 41.196095 13.152653

H 24.133486 36.065773 12.129814

O 45.837490 46.438381 44.849365

C 45.756329 46.677956 43.453476

C 45.476658 45.352474 42.786091

C 46.243301 47.571228 45.590092

O 45.698631 45.397942 41.374554

C 46.456959 44.293941 40.895405

H 46.713158 47.063717 43.060379

H 44.978443 47.425037 43.212433

H 44.444534 45.019512 42.979702

H 46.138180 44.617611 43.262794

H 46.268234 47.273956 46.648132

H 45.530933 48.407291 45.500130

H 45.931850 43.329777 41.005413

H 47.424549 44.202999 41.411713

H 46.649109 44.464581 39.827099

H 47.252445 47.915432 45.304558

O 48.359379 2.044208 13.369736

C 47.742840 2.148350 12.097056

C 47.752617 0.803798 11.421847

C 48.223946 3.251371 14.096772

O 46.981583 0.907939 10.238153

C 46.965935 48.607265 9.482269

H 48.259148 2.890542 11.458516

H 46.689201 2.472509 12.185553

H 47.329208 0.054760 12.115635

H 48.793056 0.498707 11.204762

H 48.631714 3.078291 15.101519

H 47.169327 3.553040 14.216071

H 46.439846 47.788799 10.002000

H 47.981926 48.263058 9.223138

H 46.429092 48.826305 8.546950

H 48.781322 4.083037 13.631313

O 28.721586 0.277222 31.114887

C 27.353565 0.019068 31.373531

C 26.595240 1.308371 31.251299

C 29.474047 47.975082 31.108532

O 25.226240 1.005236 31.079685

C 24.464977 2.173774 30.867981

H 27.202488 48.456184 32.369968

H 26.934067 48.216610 30.625473

H 26.996161 1.846681 30.372694

H 26.753162 1.947889 32.137238

H 30.502752 48.250835 30.842554

H 29.105394 47.251469 30.359983

H 24.821896 2.741908 29.992798

H 24.469868 2.834316 31.752449

H 23.430895 1.853526 30.674854

H 29.486269 47.485176 32.098122

O 28.605711 39.609035 33.383514

C 28.335333 40.579556 32.386589

C 29.562054 41.430294 32.208130

C 27.512957 38.741188 33.602062

O 29.383104 42.308895 31.109510

C 30.422565 43.265236 31.073820

H 27.478241 41.217609 32.668701

H 28.087446 40.111164 31.418024

H 30.433813 40.773174 32.041409

H 29.749804 41.984734 33.143940

H 27.814137 38.039577 34.392662

H 27.256269 38.157894 32.700481

H 31.419493 42.802711 30.982878

H 30.419632 43.899380 31.976379

H 30.250952 43.906223 30.198149

H 26.613327 39.282429 33.942360

O 48.740742 28.907867 40.859226

C 47.460243 29.545919 40.815708

C 46.708271 29.301943 42.087410

C 0.800862 29.801140 41.421494

O 46.784054 27.909475 42.384190

C 46.388020 27.626877 43.715542

H 46.919975 29.110771 39.962040

H 47.555580 30.625959 40.601559

H 45.661964 29.643215 42.002338

H 47.165417 29.902349 42.895119

H 0.419988 30.318426 42.317207

H 1.102042 30.568266 40.689075

H 45.331936 27.875740 43.911110

H 47.003094 28.161764 44.458710

H 46.509762 26.548302 43.875420

H 1.681422 29.220291 41.721695

O 41.084129 19.238338 46.486786

C 40.653385 20.001066 47.599098

C 40.821575 19.165977 48.836082

C 41.222008 20.026003 45.322647

O 40.193302 19.819185 1.032130

C 40.486660 19.170378 2.253469

H 39.591923 20.288555 47.494465

H 41.238632 20.931496 47.711548

H 41.901619 19.012453 0.124187

H 40.377140 18.170033 48.657135

H 41.517323 19.349325 44.509071

H 42.010647 20.790197 45.434124

H 41.568657 19.180645 2.476910

H 40.132675 18.124561 2.266671

H 39.968399 19.723356 3.049931

H 40.280823 20.521774 45.036137

O 43.879330 3.345245 22.740040

C 44.896301 4.000898 23.480278

C 44.673351 5.485772 23.394228

C 43.944359 1.940066 22.875963

O 45.347092 6.096443 24.478668

C 45.204327 7.501133 24.496761

H 45.906429 3.730521 23.118471

H 44.852299 3.727587 24.548098

H 43.585484 5.664231 23.457298

H 45.021957 5.881804 22.422728

H 43.163052 1.516166 22.228622

H 43.750252 1.617374 23.913469

H 44.162418 7.807691 24.677662

H 45.551464 7.971971 23.561928

H 45.824287 7.870274 25.325981

H 44.918793 1.530345 22.557671

O 4.219449 48.658112 48.760300

C 3.692873 47.382500 48.423916

C 4.837943 46.545944 47.926678

C 3.349646 0.558356 0.652230

O 4.437999 45.194546 47.726704

C 5.590403 44.386841 47.593231

H 2.898365 47.455841 47.661190

H 3.255772 46.874016 0.407766

H 5.630984 46.629063 48.695271

H 5.250598 46.980602 47.000648

H 3.866443 1.512746 0.831176

H 3.132562 0.101206 1.633022

H 6.333084 44.579967 48.385780

H 6.084709 44.546719 46.620262

H 5.269177 43.340046 47.674881

H 2.393303 0.771527 0.146679

O 6.449938 40.554134 16.544346

C 7.027362 39.277054 16.720360

C 5.943408 38.300175 17.068966

C 7.406281 41.564259 16.312593

O 6.466073 37.001579 16.901752

C 5.552266 36.053551 17.412682

H 7.774933 39.271187 17.531492

H 7.531935 38.932358 15.799708

H 5.068717 38.459076 16.413801

H 5.608004 38.470810 18.109404

H 6.842547 42.485889 16.118979

H 8.044821 41.347664 15.439369

H 6.014303 35.072762 17.270893

H 4.579789 36.066753 16.890018

H 5.373319 36.214409 18.489302

H 8.047266 41.733917 17.193153

O 40.187439 9.306745 11.160270

C 40.964832 8.337200 10.479683

C 40.798595 8.437430 8.998231

C 40.367363 9.079882 12.548337

O 39.447689 8.177810 8.610022

C 39.289764 6.889484 8.031131

H 40.628448 7.332453 10.778907

H 42.032650 8.418362 10.745171

H 41.495319 7.726040 8.526416

H 41.049908 9.445110 8.636913

H 39.743977 9.798117 13.094959

H 41.418068 9.229005 12.856850

H 39.694111 6.842058 7.005360

H 38.215103 6.683646 8.000328

H 39.769402 6.109155 8.636913

H 40.060806 8.059000 12.844627

O 1.364109 34.532986 6.363398

C 2.064743 33.650471 7.239556

C 3.216168 32.899475 6.612262

C 0.583291 33.822083 5.415855

O 3.658648 31.903530 7.516779

C 4.552409 31.019548 6.865038

H 1.382200 32.909252 7.696215

H 2.444151 34.279720 8.061444

H 4.041479 33.583485 6.342863

H 2.868541 32.420815 5.681343

H 0.053293 34.563297 4.799806

H 48.742210 33.152252 5.897449

H 5.543954 31.479141 6.719826

H 4.170556 30.682186 5.885715

H 4.652639 30.128721 7.495755

H 1.203741 33.215324 4.735268

O 4.797851 48.850262 11.836946

C 4.990488 47.453396 11.650665

C 6.438693 47.232399 11.359263

C 3.454276 0.291400 12.129326

O 7.192131 47.696880 12.472064

C 8.568462 47.408901 12.307784

H 4.360260 47.067631 10.828777

H 4.708376 46.885262 12.557138

H 6.717871 47.786842 10.442035

H 6.638175 46.162624 11.164182

H 3.367736 1.384642 12.046697

H 3.173143 48.882534 13.151185

H 9.021210 47.992195 11.488341

H 8.757678 46.339127 12.114657

H 9.070103 47.675858 13.247015

H 2.730663 48.734879 11.429181

O 29.846121 39.956173 23.531616

C 30.381985 39.324970 22.385078

C 29.394350 39.323990 21.255167

C 30.808819 39.987465 24.566187

O 30.032402 38.677135 20.165834

C 29.311724 38.795456 18.955738

H 30.667521 38.277683 22.597763

H 31.285524 39.851051 22.034517

H 29.114685 40.363937 21.012657

H 28.469297 38.794971 21.550480

H 30.309135 40.372738 25.465815

H 31.651731 40.655342 24.321722

H 29.360125 39.822208 18.557262

H 28.252703 38.509438 19.066725

H 29.781094 38.117805 18.228212

H 31.204363 38.986137 24.801849

O 24.853186 12.290184 40.171303

C 25.453102 11.015059 40.046623

C 25.565067 10.743215 38.575439

C 24.711887 12.699415 41.514877

O 26.285257 9.545831 38.347599

C 26.311171 9.224605 36.971756

H 24.852211 10.228862 40.539951

H 26.462252 10.993057 40.496441

H 26.080399 11.608128 38.122692

H 24.546629 10.704590 38.145672

H 24.225893 13.685583 41.501675

H 25.684855 12.799157 42.024826

H 26.886641 8.294175 36.867126

H 26.803034 10.008356 36.371841

H 25.298113 9.054458 36.566925

H 24.081659 12.004649 42.093765

O 17.622919 5.809442 17.649323

C 16.519897 6.323794 16.922775

C 16.146847 5.326382 15.854468

C 18.131895 6.746228 18.574375

O 15.870113 6.049506 14.670774

C 15.450126 5.194371 13.627400

H 16.793211 7.264492 16.410868

H 15.674542 6.559947 17.593586

H 15.292689 4.686863 16.142937

H 17.012251 4.659973 15.698010

H 19.042278 6.306193 19.004631

H 17.415615 6.948156 19.388439

H 14.490359 4.697620 13.857687

H 16.200140 4.420398 13.389782

H 15.309314 5.823132 12.739018

H 18.404226 7.702083 18.094248

O 2.053009 19.636816 42.604694

C 2.083811 18.351423 42.009182

C 2.607453 18.470234 40.607914

C 1.809034 19.525829 43.991787

O 2.674436 17.174086 40.040268

C 3.300753 17.197063 38.774925

H 1.075153 17.901121 41.995003

H 2.747286 17.659101 42.560692

H 3.603400 18.948404 40.648987

H 1.946422 19.133707 40.020710

H 1.836902 20.539375 44.412754

H 2.579584 18.920048 44.501736

H 4.369061 17.467442 38.846794

H 2.804980 17.894764 38.078690

H 3.226436 16.182049 38.360313

H 0.822377 19.082859 44.200558

O 45.849220 3.540817 35.123123

C 46.420780 2.269115 35.377361

C 46.911663 1.687291 34.083168

C 45.078186 3.986719 36.217831

O 47.556072 0.445413 34.332523

C 47.404503 48.436142 33.252972

H 45.683964 1.577282 35.819355

H 47.262714 2.337565 36.085331

H 47.611809 2.384991 33.592777

H 46.042839 1.573370 33.417252

H 44.189804 3.350624 36.371353

H 44.739357 5.005156 35.987545

H 47.865074 48.813103 32.324986

H 46.343529 48.205856 33.058868

H 47.894409 47.498867 33.544861

H 45.659519 4.016055 37.152660

O 32.655014 46.365044 18.711763

C 32.927834 44.980888 18.599310

C 33.865597 44.577030 19.700375

C 31.671778 46.753250 17.773022

O 34.048946 43.165985 19.637304

C 35.169079 42.730839 20.389275

H 33.389870 44.741798 17.623409

H 32.005714 44.378040 18.682917

H 33.440720 44.885059 20.673832

H 34.816074 45.127075 19.570320

H 31.514341 47.832802 17.897209

H 30.709566 46.237919 17.940723

H 34.937328 42.657990 21.463940

H 36.041328 43.390404 20.260199

H 35.444347 41.732449 20.021601

H 31.991537 46.562080 16.735518

O 12.776666 42.977261 43.370846

C 13.840574 43.856842 43.679359

C 15.079517 43.011486 43.667137

C 11.510832 43.592819 43.339558

O 16.223120 43.816261 43.449078

C 17.322229 42.993393 43.117580

H 13.696830 44.364838 44.650860

H 13.950094 44.639614 42.907833

H 14.946039 42.288361 42.844757

H 15.169970 42.423794 44.599525

H 10.791618 42.795376 43.108292

H 11.439448 44.366791 42.558739

H 17.184351 42.500557 42.139725

H 17.497267 42.218441 43.880798

H 18.213057 43.632912 43.059887

H 11.238988 44.040188 44.309101

O 43.966850 15.435456 32.931255

C 43.906223 15.039914 34.295853

C 43.088737 13.781902 34.427864

C 45.207748 16.007502 32.560162

O 43.322445 13.247993 35.720589

C 42.977261 11.880950 35.823753

H 43.486721 15.852023 34.910927

H 44.907547 14.813540 34.696289

H 43.418274 13.089581 33.633846

H 42.013092 13.974541 34.262608

H 45.126099 16.262722 31.495274

H 46.046261 15.298068 32.674080

H 43.610909 11.250722 35.179344

H 41.920685 11.690269 35.572445

H 43.148384 11.587105 36.868591

H 45.442436 16.921309 33.132694

O 26.274990 14.822830 26.247122

C 26.177204 15.785529 27.284140

C 25.608093 15.161168 28.525036

C 26.484739 15.470659 25.008177

O 25.385630 16.194273 29.467201

C 24.522671 15.798731 30.513508

H 25.488794 16.599106 27.003006

H 27.154572 16.253922 27.498289

H 26.279881 14.387685 28.936716

H 24.663973 14.667840 28.239016

H 26.546345 14.690331 24.239584

H 27.421528 16.054440 24.999866

H 24.937283 14.973908 31.116356

H 23.529659 15.497550 30.139477

H 24.392130 16.675867 31.160847

H 25.650629 16.145868 24.745625

O 14.919638 7.519224 30.317936

C 16.300371 7.183330 30.294958

C 16.923265 7.497221 31.628262

C 14.449288 7.952414 29.055035

O 18.164654 6.817612 31.731426

C 18.968451 7.412637 32.729328

H 16.841614 7.739241 29.512182

H 16.424557 6.110133 30.076405

H 16.244143 7.187730 32.440861

H 17.045498 8.592421 31.713823

H 13.386847 8.201279 29.173845

H 14.550008 7.170129 28.286928

H 18.484413 7.399436 33.720387

H 19.220737 8.459433 32.481930

H 19.903280 6.838636 32.784088

H 14.976843 8.856931 28.704473

O 15.868647 40.357582 20.535954

C 14.810118 39.468224 20.839579

C 13.587310 40.264198 21.233654

C 16.912508 39.682373 19.860744

O 12.461308 39.523472 20.799974

C 11.200363 39.991375 21.232676

H 15.121566 38.748520 21.621374

H 14.537295 38.877110 19.947285

H 13.622512 41.237167 20.711479

H 13.552106 40.476883 22.316139

H 17.715816 40.407944 19.669085

H 16.560480 39.306389 18.889734

H 11.070797 41.076305 21.092844

H 11.003814 39.745445 22.288271

H 10.456214 39.464798 20.618093

H 17.329075 38.841908 20.444035

O 1.168538 4.965064 17.038162

C 2.398681 5.146456 17.717285

C 2.727729 3.848841 18.429163

C 0.798419 6.116489 16.304281

O 3.244526 4.036590 19.747313

C 2.238312 4.032678 20.746681

H 2.317519 5.950253 18.468277

H 3.198078 5.450080 17.018116

H 3.493391 3.270928 17.892809

H 1.820279 3.224969 18.439919

H 48.694294 5.925807 15.881360

H 1.497097 6.319883 15.474571

H 1.830546 3.023042 20.915850

H 1.404690 4.706910 20.496349

H 2.706216 4.376395 21.679068

H 0.735348 7.019051 16.936466

O 14.001920 20.034803 6.004525

C 13.281240 20.215706 4.792472

C 13.985297 21.273746 3.983785

C 13.940315 18.721052 6.531100

O 13.269994 21.534834 2.787378

C 13.985297 22.412460 1.941533

H 12.245690 20.521774 5.012979

H 13.234303 19.287720 4.201358

H 15.009112 20.917807 3.771101

H 14.084548 22.182663 4.604724

H 14.315812 17.973482 5.813843

H 12.922856 18.426718 6.834236

H 14.916705 21.957268 1.564081

H 14.241005 23.356579 2.449040

H 13.334533 22.643234 1.086398

H 14.595968 18.706873 7.412637

O 38.031261 2.226578 7.100701

C 39.034546 2.161062 6.093998

C 39.128418 3.376537 5.210505

C 36.752228 2.284272 6.487097

O 40.280823 3.206390 4.414042

C 40.540443 4.328479 3.601933

H 38.918179 1.259479 5.462792

H 40.000668 2.051542 6.613240

H 39.195400 4.277631 5.846601

H 38.224880 3.480678 4.580277

H 36.552746 3.281685 6.058796

H 36.000748 2.070121 7.251780

H 40.784904 5.223707 4.198424

H 39.701443 4.567076 2.926234

H 41.412201 4.067392 2.991750

H 36.629509 1.542079 5.685255

O 7.449796 32.395393 8.636424

C 8.445253 33.400627 8.683361

C 9.074015 33.493034 7.323164

C 6.905130 32.138702 9.916438

O 9.947240 34.606323 7.287472

C 10.619515 34.689930 6.049995

H 9.224605 33.179630 9.435332

H 8.006685 34.379459 8.950316

H 8.270217 33.589352 6.570214

H 9.606946 32.547939 7.112925

H 6.018215 31.506519 9.772204

H 6.581948 33.058376 10.431767

H 9.919371 34.823895 5.210017

H 11.242899 33.800571 5.852957

H 11.275168 35.569511 6.101332

H 7.617009 31.599903 10.566712

O 47.517445 24.392130 27.562338

C 47.387878 23.201101 28.323109

C 46.116669 22.511711 27.965704

C 48.726074 25.036047 27.932457

O 45.020000 23.415251 28.060066

C 43.798660 22.707771 28.208212

H 47.376144 23.436274 29.404617

H 48.239590 22.514156 28.154428

H 45.976833 21.660976 28.645802

H 46.182671 22.096123 26.943354

H 48.723629 26.037373 27.482153

H 0.724590 24.491381 27.571629

H 43.697449 22.279959 29.219805

H 43.692074 21.883928 27.482643

H 42.980194 23.421606 28.045399

H 48.818970 25.154858 29.025209

O 13.514460 3.108115 10.512930

C 14.324612 3.412717 9.390350

C 14.987110 2.161062 8.892134

C 12.916988 4.293277 11.003814

O 15.777706 2.469086 7.753909

C 16.066664 1.328906 6.972602

H 15.094185 4.159799 9.643126

H 13.720786 3.832707 8.568462

H 14.189668 1.439404 8.648647

H 15.609026 1.721027 9.692019

H 12.299473 4.021433 11.871172

H 12.270137 4.776826 10.249887

H 15.149922 0.845357 6.591727

H 16.664623 0.584269 7.523135

H 16.645554 1.680934 6.110133

H 13.669938 5.028625 11.333351

O 0.914785 15.657430 10.972522

C 48.740742 15.172903 10.174103

C 47.745777 14.514317 11.068841

C 1.930286 16.245123 10.184370

O 47.203552 15.512707 11.924953

C 46.036972 15.029158 12.564960

H 0.199485 14.475202 9.396218

H 48.241058 16.006035 9.647527

H 48.230301 13.711007 11.656532

H 46.959579 14.028811 10.464526

H 2.713062 16.595194 10.873758

H 1.565546 17.110523 9.603523

H 46.256500 14.193581 13.252394

H 45.273754 14.688864 11.842325

H 45.603783 15.862290 13.135540

H 2.386947 15.522975 9.483247

O 0.330515 10.306602 33.943336

C 1.350908 11.150492 33.443165

C 0.760283 12.476464 33.075489

C 0.741704 8.956183 33.918892

O 1.816368 13.352623 32.736176

C 1.325973 14.467868 32.021362

H 1.825168 10.733925 32.536201

H 2.146394 11.296192 34.195625

H 0.166724 12.875919 33.915466

H 0.073339 12.327342 32.225243

H 48.848797 8.365069 34.406353

H 1.689246 8.791903 34.459156

H 0.570579 15.033558 32.593407

H 0.889360 14.167666 31.052307

H 2.177685 15.132810 31.820900

H 0.854157 8.592910 32.883831

O 33.996143 33.852886 21.881483

C 33.028553 32.885296 22.257469

C 32.793865 31.898149 21.137825

C 34.054325 34.843452 22.893564

O 31.479630 31.354954 21.262989

C 31.200939 30.447014 20.207882

H 32.072697 33.387913 22.455975

H 33.337067 32.369480 23.185944

H 33.569309 31.116356 21.154938

H 32.892139 32.426193 20.175613

H 34.777451 35.612049 22.581139

H 34.387772 34.420532 23.856264

H 31.867838 29.566942 20.200060

H 31.272812 30.926651 19.215849

H 30.175657 30.073473 20.354073

H 33.078423 35.334335 23.051489

O 36.808945 25.704901 16.500830

C 35.707390 24.858076 16.234854

C 34.853230 24.804785 17.473797

C 37.554562 25.931763 15.322025

O 33.567841 24.291409 17.152573

C 32.740086 24.194113 18.296175

H 36.041817 23.846975 15.939053

H 35.104542 25.260466 15.402699

H 34.785759 25.836910 17.866407

H 35.354385 24.196068 18.247770

H 38.404316 26.579103 15.581646

H 36.955624 26.442694 14.547074

H 32.606121 25.159258 18.817371

H 33.127804 23.458277 19.019789

H 31.750984 23.853331 17.957348

H 37.951569 24.994976 14.895679

O 32.241867 28.693718 44.815140

C 33.372269 29.492138 44.513962

C 32.995796 30.927629 44.777985

C 32.493664 27.313963 44.665039

O 33.936005 31.869795 44.252872

C 34.737354 32.499043 45.241486

H 33.661716 29.387995 43.452988

H 34.247936 29.207092 45.121212

H 32.820759 31.072840 45.854603

H 32.036518 31.123201 44.275852

H 31.564703 26.782009 44.910480

H 33.278885 26.961935 45.351982

H 35.304024 31.773476 45.848244

H 34.133530 33.128292 45.917671

H 35.453636 33.143940 44.710999

H 32.768932 27.055809 43.630466

O 3.380937 43.567883 16.931576

C 2.791779 43.621666 15.645207

C 2.264225 45.016090 15.449636

C 3.800926 42.264404 17.273336

O 2.185997 45.305534 14.064013

C 1.543057 46.546432 13.857687

H 1.987492 42.872143 15.528354

H 3.541306 43.419739 14.861944

H 2.960459 45.711834 15.953232

H 1.281480 45.125122 15.945409

H 4.283498 42.324543 18.258528

H 4.525517 41.862991 16.549725

H 2.050564 47.368809 14.389151

H 0.486972 46.522964 14.180867

H 1.573859 46.750317 12.778133

H 2.957036 41.559368 17.349121

O 21.472250 13.706119 39.789940

C 21.403801 12.297517 39.637394

C 20.789707 11.676089 40.859711

C 22.289249 14.254207 38.773457

O 19.363993 11.652621 40.847000

C 18.849640 10.491417 40.215794

H 22.410015 11.873128 39.475071

H 20.789707 12.043763 38.759766

H 21.075241 12.301918 41.712402

H 21.199919 10.670364 41.050396

H 22.259424 15.344027 38.884445

H 21.935265 13.994098 37.760887

H 17.781334 10.669875 40.043201

H 18.958183 9.601079 40.852867

H 19.329769 10.284600 39.245274

H 23.339956 13.930047 38.869286

O 45.140766 28.594954 15.491684

C 46.302460 28.871199 14.720156

C 47.096966 27.638609 14.395996

C 44.468979 29.829008 15.679920

O 48.097801 28.072289 13.495879

C 0.116366 27.069986 13.098380

H 46.971802 29.589432 15.234018

H 46.028172 29.329811 13.752567

H 46.442291 26.878328 13.934447

H 47.535534 27.198574 15.308336

H 43.545395 29.643703 16.246588

H 44.194202 30.304245 14.720156

H 48.516323 26.255434 12.542469

H 0.667384 26.640707 13.952538

H 0.845356 27.554026 12.430994

H 45.087471 30.546265 16.248543

O 44.184914 48.453255 6.724716

C 43.742920 47.205997 7.230756

C 42.921032 47.405972 8.463832

C 44.917812 48.268929 5.530264

O 43.607979 48.084114 9.525784

C 43.277462 0.565199 9.648993

H 44.603924 46.546921 7.456641

H 43.105850 46.678444 6.496387

H 42.647232 46.419800 8.862798

H 41.979359 47.915920 8.198833

H 45.168633 0.370609 5.141567

H 44.329147 47.743816 4.762648

H 42.322098 0.711389 10.182903

H 43.216835 1.071239 8.673582

H 44.087124 1.017460 10.236197

H 45.856556 47.712036 5.690144

O 40.516483 41.011280 8.795326

C 41.172138 42.087898 8.142118

C 42.642345 41.985226 8.375825

C 39.148464 40.978031 8.451120

O 42.925922 42.217464 9.745802

C 44.313988 42.107944 9.983910

H 40.811310 43.060379 8.525437

H 40.972164 42.066875 7.054742

H 43.182610 42.708351 7.735818

H 42.966991 40.973633 8.072201

H 38.702072 40.128765 8.984052

H 39.001785 40.821087 7.368145

H 44.893860 42.853561 9.411863

H 44.695843 41.100266 9.742867

H 44.475338 42.293739 11.053195

H 38.620422 41.900154 8.750344

O 44.306656 32.917080 36.420734

C 45.282555 33.082336 37.436729

C 46.557190 32.442818 36.952690

C 43.118561 33.617710 36.723381

O 47.285694 31.911352 38.047890

C 48.552017 31.475716 37.595142

H 44.967197 32.576294 38.366669

H 45.436077 34.149178 37.684124

H 47.161015 33.171810 36.381618

H 46.282410 31.633642 36.252056

H 42.394455 33.389870 35.927898

H 43.281864 34.709976 36.741474

H 0.264018 32.307384 37.192261

H 48.460587 30.715921 36.805035

H 0.190195 31.040571 38.452721

H 42.688793 33.308220 37.690971

O 39.569431 42.099636 13.450409

C 38.529972 41.197563 13.771146

C 38.435608 41.111019 15.266288

C 39.741047 42.260490 12.055986

O 37.414238 40.200638 15.640317

C 37.270981 40.212860 17.048429

H 38.736790 40.192326 13.363379

H 37.560917 41.538834 13.364847

H 38.238079 42.120171 15.663298

H 39.415417 40.796642 15.670141

H 40.639206 42.876541 11.918109

H 38.885910 42.776798 11.586127

H 36.825081 41.156490 17.409260

H 38.232704 40.065205 17.567673

H 36.609951 39.379726 17.322718

H 39.908260 41.303169 11.532834

O 31.374022 11.968957 42.018471

C 31.127602 10.679165 41.497272

C 29.822163 10.176547 42.049763

C 32.608562 12.483798 41.565723

O 29.846611 8.753767 42.064922

C 28.599842 8.260927 42.517178

H 31.917221 9.981465 41.819477

H 31.118311 10.683566 40.391319

H 28.966539 10.563290 41.464520

H 29.710688 10.587247 43.069668

H 32.718082 13.482678 42.009674

H 32.641811 12.588918 40.467102

H 27.764755 8.583131 41.867882

H 28.376890 8.583131 43.549305

H 28.649223 7.162796 42.500065

H 33.465164 11.866771 41.888905

O 48.216122 47.840137 6.476340

C 0.141300 48.029842 5.332738

C 0.034225 0.561288 4.864833

C 48.122738 46.472118 6.825924

O 1.071239 0.805752 3.929025

C 1.029192 2.142973 3.474811

H 48.743187 47.358055 4.504004

H 1.201295 47.823025 5.569379

H 0.122719 1.229167 5.740015

H 47.928631 0.737792 4.421865

H 47.471973 46.400246 7.707461

H 0.208770 46.026703 7.066965

H 1.086888 2.860715 4.309900

H 0.113918 2.350275 2.894943

H 1.900952 2.296985 2.823559

H 47.671947 45.868290 6.026038

O 41.435181 42.481487 46.383621

C 41.256721 41.727558 47.571716

C 42.534290 41.722668 48.357422

C 40.190369 42.896587 45.853134

O 42.232624 41.298283 0.783752

C 43.368889 41.343750 1.618352

H 40.910561 40.699833 47.352188

H 40.500351 42.190575 48.225414

H 42.924454 42.757732 48.355957

H 43.298485 41.083641 47.883652

H 40.393764 43.417782 44.908035

H 39.666241 43.599667 46.518078

H 43.948269 42.268314 1.482919

H 44.034813 40.492531 1.424736

H 43.020287 41.303661 2.658790

H 39.520538 42.043407 45.655117

O 48.797947 6.084709 33.377647

C 0.746104 5.236419 32.620789

C 1.804144 6.126268 32.044830

C 47.776577 5.379675 34.044544

O 2.771733 5.386031 31.326595

C 3.750567 6.290547 30.866022

H 0.194104 4.720600 31.813566

H 1.216453 4.462446 33.253948

H 2.266670 6.672400 32.888721

H 1.310327 6.876284 31.396511

H 47.195732 6.118934 34.612682

H 48.180431 4.633571 34.750069

H 4.251229 6.795610 31.705023

H 3.324221 7.057186 30.192770

H 4.503027 5.716057 30.313046

H 47.091587 4.870701 33.346355

O 43.195812 17.030340 4.283498

C 42.684391 15.708767 4.258563

C 43.718475 14.789582 3.663537

C 42.395435 17.882542 5.078984

O 43.330753 13.460188 3.958850

C 44.253853 12.486243 3.518326

H 41.737335 15.656452 3.689939

H 42.461441 15.343049 5.276511

H 44.696331 15.034046 4.117263

H 43.815281 14.958752 2.576162

H 42.815914 18.893156 4.990977

H 42.412548 17.591629 6.144358

H 45.248817 12.610431 3.979874

H 44.368259 12.485266 2.420682

H 43.850971 11.509854 3.825861

H 41.346195 17.912367 4.740646

O 40.921806 7.990062 1.916598

C 41.089020 8.625668 0.663964

C 42.546028 8.977207 0.534398

C 39.605614 7.546114 2.152750

O 42.816891 9.366393 48.093891

C 44.202999 9.540452 47.884140

H 40.457325 9.527250 0.574001

H 40.816685 7.956814 48.719719

H 43.130783 8.082957 0.818954

H 42.812004 9.776115 1.251656

H 39.612946 7.065010 3.140385

H 39.275585 6.807833 1.403712

H 44.777985 8.625668 48.114914

H 44.605389 10.374563 48.482590

H 44.342346 9.775626 46.819744

H 38.885422 8.378270 2.178174

O 22.545448 37.089100 33.374226

C 21.135868 36.992779 33.279861

C 20.576046 38.384270 33.183540

C 23.160030 35.816422 33.386936

O 19.210958 38.317776 32.808044

C 18.681450 39.608543 32.587540

H 20.704634 36.460827 34.146732

H 20.834688 36.439312 32.373390

H 21.173515 38.926491 32.426682

H 20.704145 38.917202 34.143307

H 24.243494 35.983635 33.470543

H 22.967882 35.249264 32.458950

H 19.272564 40.183525 31.852682

H 18.613977 40.191837 33.522369

H 17.671814 39.473602 32.180264

H 22.838804 35.205257 34.247452

O 21.539232 35.801750 21.634575

C 20.196148 35.371986 21.500608

C 20.075384 34.057747 22.214933

C 21.746050 37.026028 20.965721

O 19.777138 34.175579 23.604954

C 18.382227 34.169220 23.861153

H 19.937017 35.248775 20.433767

H 19.486713 36.099998 21.931843

H 21.065462 33.581532 22.159683

H 19.375238 33.373245 21.709869

H 22.813379 37.271957 21.052261

H 21.158848 37.843025 21.417002

H 18.251682 34.385815 24.930437

H 17.936323 33.183540 23.648470

H 17.841959 34.935860 23.279329

H 21.491808 36.958553 19.893991

O 4.020455 17.725595 34.345234

C 3.780391 16.749695 35.346073

C 3.583842 15.433990 34.674774

C 4.560231 18.909779 34.889416

O 4.852121 14.995911 34.221539

C 4.800295 13.772123 33.521393

H 2.900810 17.036207 35.946964

H 4.640904 16.647999 36.033504

H 2.880275 15.580668 33.839195

H 3.138918 14.692286 35.359760

H 3.871821 19.398706 35.599827

H 4.745535 19.592323 34.050900

H 4.252207 13.866976 32.569939

H 4.342659 12.966370 34.120331

H 5.839267 13.491969 33.297951

H 5.523420 18.715185 35.387142

O 34.938305 14.155443 46.956154

C 34.563297 13.483168 45.763168

C 35.425278 14.023922 44.653793

C 33.973648 14.147132 47.988285

O 34.914837 13.578997 43.411915

C 35.608624 14.128551 42.309383

H 33.508678 13.664070 45.493282

H 34.681129 12.389435 45.859978

H 36.471096 13.708074 44.808784

H 35.415497 15.126944 44.721756

H 34.420040 14.700598 48.826794

H 33.720387 13.128695 48.320267

H 36.670578 13.832261 42.292271

H 35.547508 15.230107 42.279072

H 35.127522 13.735943 41.402912

H 33.042732 14.662462 47.697861

O 2.348810 42.444817 47.104790

C 0.985190 42.429661 47.507664

C 0.424390 43.826527 47.517933

C 2.834316 41.121292 46.981583

O 47.914455 43.709187 47.652878

C 47.259781 44.956928 47.561935

H 0.855624 41.971535 48.505081

H 0.369141 41.828770 46.813389

H 0.690366 44.325233 46.570393

H 0.857580 44.428398 48.336399

H 3.879155 41.185825 46.649597

H 2.274004 40.542889 46.226189

H 47.477352 45.467369 46.610973

H 47.515980 45.633118 48.397026

H 46.179249 44.755001 47.601540

H 2.804491 40.564888 47.936943

O 31.971003 11.802722 7.249824

C 32.243824 11.945489 5.866647

C 31.167204 11.226275 5.105875

C 32.984058 12.401170 8.035532

O 31.367664 11.317217 3.699718

C 30.383451 10.541287 3.044555

H 33.230480 11.522566 5.602137

H 32.245293 13.011352 5.583558

H 30.193748 11.661910 5.396787

H 31.164268 10.175080 5.448124

H 32.654034 12.359611 9.082815

H 33.152252 13.459700 7.770533

H 29.364037 10.924118 3.231325

H 30.407898 9.479825 3.348668

H 30.589291 10.587247 1.967446

H 33.942360 11.858949 7.954858

O 33.953606 18.185677 35.618404

C 33.745323 16.821079 35.290333

C 33.414318 16.074486 36.551277

C 34.211269 18.935204 34.446934

O 32.756706 14.862923 36.220764

C 32.027718 14.388173 37.336987

H 34.623436 16.381533 34.782825

H 32.883339 16.708626 34.606323

H 32.758175 16.724272 37.150703

H 34.313457 15.881847 37.163906

H 34.314922 19.985422 34.750069

H 33.385468 18.867731 33.716965

H 31.165737 15.040892 37.562870

H 32.649147 14.311900 38.245903

H 31.650755 13.385870 37.091057

H 35.146099 18.619356 33.950180

O 24.718733 38.685452 24.435154

C 24.223936 39.906303 23.916401

C 24.551029 39.966442 22.452063

C 24.400928 38.534370 25.801706

O 24.329056 41.287525 21.991493

C 24.637571 41.407310 20.618584

H 24.701130 40.770729 24.413643

H 23.131672 40.002621 24.062101

H 23.937426 39.231091 21.898596

H 25.608580 39.669170 22.323963

H 24.879101 37.605408 26.137602

H 23.313065 38.449299 25.967455

H 23.951117 40.814243 19.993242

H 25.674099 41.101734 20.392698

H 24.520229 42.464863 20.350649

H 24.791582 39.361637 26.416290

O 47.878273 34.132061 16.755074

C 46.776230 33.286705 17.029850

C 45.600845 33.831375 16.271034

C 0.112944 33.835285 17.554960

O 44.492447 32.954235 16.381533

C 43.383556 33.464188 15.667209

H 46.538612 33.258350 18.108915

H 46.980602 32.250668 16.703247

H 45.907406 33.964363 15.217394

H 45.368607 34.834164 16.673912

H 0.939719 34.461601 17.189241

H 0.418520 32.778221 17.479176

H 43.613842 33.626511 14.599879

H 43.017841 34.414173 16.094532

H 42.578297 32.719063 15.738103

H 48.832661 34.076813 18.618378

O 38.503082 28.742611 16.921309

C 38.687405 29.763491 15.955676

C 39.965954 29.479425 15.217394

C 37.198132 28.746521 17.465485

O 40.316025 30.595160 14.412619

C 41.225922 30.236286 13.389782

H 38.747055 30.756992 16.435314

H 37.856228 29.796740 15.227663

H 39.820744 28.564152 14.616502

H 40.763882 29.262831 15.949320

H 37.156570 27.931479 18.201811

H 36.427582 28.555349 16.697870

H 40.766327 29.555208 12.651011

H 42.134834 29.756645 13.787280

H 41.513901 31.163292 12.876408

H 36.960510 29.696020 17.972992

O 20.096897 10.803353 31.046438

C 20.971590 11.604706 31.821388

C 22.330318 10.962743 31.856104

C 18.776791 11.308905 31.067463

O 23.265638 11.866282 32.416416

C 24.524630 11.259034 32.625675

H 20.598047 11.732805 32.851070

H 21.071819 12.610431 31.384289

H 22.616831 10.705567 30.824955

H 22.272625 10.019601 32.428150

H 18.161230 10.613648 30.480259

H 18.707363 12.314140 30.617159

H 25.006222 10.961765 31.678623

H 24.459112 10.374074 33.281818

H 25.156813 12.005628 33.125847

H 18.359735 11.356819 32.087856

O 44.503693 18.466810 43.833374

C 44.367283 17.150127 44.341370

C 43.768345 17.232267 45.726013

C 45.100674 18.465343 42.552383

O 42.966015 16.107244 46.068260

C 43.706741 15.043337 46.636887

H 45.329979 16.608395 44.337948

H 43.669090 16.577103 43.710651

H 43.079445 18.085936 45.719658

H 44.524719 17.450329 46.497540

H 45.181347 19.515072 42.238491

H 44.493423 17.922634 41.807255

H 44.099838 15.308336 47.632832

H 44.544273 14.719666 45.997856

H 43.015396 14.197491 46.745914

H 46.116177 18.028242 42.564114

O 13.776524 1.548435 33.336086

C 12.539536 1.865749 33.947735

C 11.488341 1.926865 32.877472

C 14.801317 1.352375 34.286564

O 10.221040 1.975269 33.499878

C 9.173267 1.994337 32.553802

H 12.584517 2.825515 34.496315

H 12.240313 1.089821 34.674286

H 11.588082 1.023326 32.247734

H 11.656043 2.807425 32.229645

H 15.702411 1.065374 33.726254

H 14.561742 0.546622 35.002846

H 9.172289 1.093732 31.915266

H 9.217271 2.890054 31.913307

H 8.228169 2.020251 33.115093

H 15.014001 2.276449 34.846874

O 41.116402 22.317608 41.453270

C 42.026783 23.281773 40.953098

C 41.268948 24.330034 40.190369

C 41.803345 21.254189 42.083500

O 42.204266 25.254597 39.668198

C 41.586262 26.231964 38.858532

H 42.766041 22.826092 40.269089

H 42.591496 23.768745 41.771076

H 40.544842 24.824831 40.864113

H 40.694454 23.836706 39.385105

H 41.040615 20.585337 42.504955

H 42.455574 21.599861 42.905388

H 40.796642 26.784941 39.395374

H 41.153557 25.795351 37.941792

H 42.367569 26.947754 38.569576

H 42.418903 20.677254 41.371132

O 12.016384 3.481656 38.970005

C 11.303038 4.576855 38.418495

C 11.791476 5.847579 39.076103

C 11.862371 2.307740 38.198479

O 12.012472 6.791698 38.041042

C 12.670081 7.959748 38.481567

H 10.212238 4.425776 38.506500

H 11.519632 4.672196 37.341385

H 12.735107 5.608493 39.597301

H 11.099643 6.245077 39.840298

H 12.338098 1.490252 38.758301

H 12.349342 2.394280 37.212311

H 13.679228 7.746575 38.877598

H 12.090212 8.496591 39.249184

H 12.771288 8.616867 37.606384

H 10.803353 2.052520 38.038109

O 34.428352 46.051147 37.010384

C 33.225101 45.845802 36.295082

C 33.560997 45.597912 34.854698

C 34.158466 46.256989 38.378891

O 32.429127 45.901047 34.052856

C 32.642788 45.413101 32.742531

H 32.578739 46.737602 36.352287

H 32.648167 44.995556 36.702358

H 33.895424 44.551609 34.722198

H 34.413685 46.243786 34.577965

H 35.117741 46.411003 38.890800

H 33.668556 45.381805 38.831150

H 32.637901 44.308609 32.716125

H 33.600597 45.759750 32.319607

H 31.826279 45.785172 32.110348

H 33.526772 47.145859 38.547081

O 14.830653 17.707506 41.593594

C 15.547421 16.539455 41.951488

C 16.801031 16.455849 41.125202

C 13.612733 17.795023 42.305962

O 17.494331 15.283401 41.507542

C 18.557262 14.914260 40.654854

H 14.949462 15.627605 41.770584

H 15.817799 16.543367 43.022728

H 17.417570 17.358410 41.288013

H 16.515499 16.437759 40.061291

H 13.115493 18.723009 41.988159

H 13.772613 17.838539 43.396759

H 19.304344 15.713167 40.541420

H 18.207190 14.598901 39.656948

H 19.054991 14.060102 41.133514

H 12.938501 16.948200 42.088390

O 5.138144 5.805042 48.566196

C 3.716342 5.680366 48.533436

C 2.964859 6.922243 0.025424

C 5.686233 5.543954 0.955365

O 2.978549 7.840939 47.839161

C 2.480332 9.091616 48.263058

H 3.393160 4.818385 0.246909

H 3.459654 5.447147 47.489574

H 3.431786 7.358855 0.924074

H 1.922465 6.664088 0.290912

H 5.350339 6.269524 1.715648

H 6.777031 5.625606 0.855135

H 3.149674 9.559031 0.108542

H 1.476563 9.006543 48.707985

H 2.422638 9.742379 47.379078

H 5.444213 4.527962 1.312772

O 9.871456 36.847569 14.640460

C 10.701655 36.590393 13.510058

C 10.008845 36.556660 12.171373

C 9.050547 35.750904 15.006667

O 11.016525 36.531719 11.173471

C 10.456214 36.364998 9.887101

H 11.290325 35.664364 13.632779

H 11.422336 37.424015 13.478767

H 9.374216 37.453842 12.067720

H 9.347815 35.673653 12.095590

H 8.448187 35.362694 14.167666

H 8.356268 36.113197 15.775750

H 9.778560 37.191776 9.615746

H 9.907147 35.414032 9.802517

H 11.283969 36.347397 9.164466

H 9.631392 34.916306 15.434479

O 36.903305 4.308434 28.226303

C 38.021976 3.665004 27.640078

C 38.570061 2.631899 28.591530

C 36.127380 4.992444 27.261646

O 39.348923 1.728360 27.826849

C 39.921459 0.695745 28.603756

H 38.791058 4.405730 27.356010

H 37.741817 3.134028 26.715515

H 37.719818 2.113147 29.069214

H 39.159710 3.102248 29.400219

H 35.377361 5.577691 27.808758

H 35.597382 4.297677 26.587414

H 39.155312 0.032758 29.038410

H 40.557556 1.081509 29.418308

H 40.556580 0.101208 27.930988

H 36.731205 5.681832 26.649021

O 15.646185 10.168235 12.325874

C 15.144545 11.419402 11.893173

C 15.752770 11.720582 10.554977

C 15.097119 9.778071 13.568240

O 15.395853 13.035309 10.175080

C 15.715612 13.290529 8.823195

H 15.403187 12.227111 12.600652

H 14.042500 11.412558 11.797343

H 15.393898 10.973989 9.824030

H 16.849436 11.610085 10.623427

H 15.408077 8.740077 13.753056

H 13.997031 9.820607 13.581442

H 15.291712 12.536603 8.136250

H 16.804455 13.342356 8.668694

H 15.273622 14.263986 8.566018

H 15.464792 10.408299 14.387685

O 21.594482 8.540105 42.402767

C 21.094309 7.529980 41.545681

C 22.110302 7.312407 40.462700

C 20.684097 8.923914 43.412895

O 21.711826 6.220142 39.655483

C 22.666702 5.958076 38.648781

H 20.131121 7.812580 41.086575

H 20.934429 6.581460 42.090836

H 23.086203 7.123192 40.941360

H 22.211998 8.242348 39.870125

H 21.207741 9.658772 44.039700

H 20.378519 8.078069 44.050945

H 23.619133 5.605071 39.074635

H 22.865696 6.843525 38.022953

H 22.261379 5.162102 38.009262

H 19.778116 9.403064 43.003174

O 0.241042 38.401382 3.390227

C 48.782299 38.421917 2.019273

C 48.763721 37.009892 1.505409

C 0.307534 39.711224 3.915824

O 0.011733 37.060253 0.096808

C 0.072850 35.776817 48.400936

H 0.634628 38.987606 1.431092

H 47.801514 38.903999 1.853037

H 47.830849 36.495052 1.804144

H 0.706988 36.465717 1.975758

H 0.617026 39.621258 4.964086

H 48.223946 40.224594 3.886488

H 48.179451 35.095253 48.761276

H 1.048263 35.301579 48.574505

H 48.833149 35.915672 47.318939

H 1.049242 40.338516 3.392671

O 32.324497 47.272491 27.283159

C 31.305573 47.837204 26.460295

C 29.960041 48.106602 27.096878

C 31.948999 45.989544 27.748129

O 29.306833 0.176993 26.277925

C 27.914856 0.253265 26.511633

H 31.165249 47.250004 25.533289

H 31.675688 48.825325 26.152760

H 30.105253 48.511925 28.113359

H 29.365992 47.179596 27.192707

H 31.023458 46.007633 28.350491

H 32.748886 45.622849 28.404272

H 27.670879 0.463014 27.568205

H 27.398060 48.221992 26.198229

H 27.532024 1.077598 25.896070

H 31.822369 45.274242 26.919399

O 18.903423 44.091038 36.474030

C 17.851250 43.840218 37.383923

C 18.214523 44.490490 38.687893

C 18.718607 43.465698 35.222370

O 17.056742 44.569210 39.499516

C 17.302183 45.353447 40.651917

H 17.678659 42.758709 37.531094

H 16.903708 44.281231 37.026028

H 18.618378 45.494263 38.458588

H 19.020277 43.920891 39.188065

H 19.621170 43.681316 34.636635

H 17.843916 43.863197 34.680641

H 17.513401 46.407089 40.403053

H 18.146563 44.968666 41.247921

H 16.393267 45.325092 41.270412

H 18.620823 42.370010 35.308422

O 43.259369 39.805096 21.496208

C 44.074413 38.740208 21.048349

C 43.235413 37.713463 20.346249

C 44.050945 40.784904 22.141106

O 44.113529 36.693069 19.906704

C 43.457878 35.559242 19.380129

H 44.586323 38.246880 21.894684

H 44.856209 39.093704 20.351627

H 42.701015 38.183807 19.501383

H 42.473175 37.331608 21.049818

H 43.374271 41.559856 22.527357

H 44.757446 41.266502 21.445848

H 42.898056 35.781708 18.459476

H 42.761642 35.102585 20.102762

H 44.246517 34.832207 19.136642

H 44.620056 40.375675 22.994284

O 36.913574 21.574924 35.871670

C 36.498966 22.516602 36.853436

C 37.470955 22.485310 38.007793

C 36.472073 21.857037 34.555965

O 36.787430 22.811424 39.207138

C 37.642567 22.635410 40.322380

H 35.503506 22.233511 37.233334

H 36.418289 23.532595 36.436867

H 38.328533 23.163452 37.840580

H 37.882141 21.463940 38.059132

H 36.911129 22.788445 34.158466

H 35.374916 21.930376 34.484089

H 38.476677 23.358536 40.322872

H 38.067448 21.616974 40.367851

H 37.044605 22.793823 41.230808

H 36.813835 21.022436 33.927204

O 38.715763 1.079064 9.904215

C 37.802937 0.504574 10.832200

C 38.466412 48.869331 12.080433

C 39.604145 0.123699 9.355637

O 37.570206 0.091918 13.176121

C 38.295773 0.078228 14.394040

H 37.140926 48.653225 10.352561

H 37.159992 1.337218 11.156360

H 39.364571 0.594536 12.236402

H 38.824795 47.828403 11.978248

H 40.278866 0.656141 8.670650

H 39.080990 48.241058 8.778703

H 38.791058 1.045328 14.577876

H 39.062412 48.178474 14.420442

H 37.581940 48.792080 15.207128

H 40.220684 48.524147 10.127166

O 11.201829 7.058165 34.346703

C 12.520956 6.732539 34.746647

C 13.162431 5.983990 33.613800

C 10.552044 7.923567 35.256111

O 14.439022 5.507285 34.005920

C 15.011067 4.743091 32.964016

H 12.519490 6.095954 35.649208

H 13.116470 7.634611 34.977421

H 13.235281 6.657732 32.738129

H 12.488688 5.153790 33.332176

H 9.574188 8.171943 34.818520

H 11.107955 8.865242 35.408165

H 15.068272 5.302424 32.015003

H 14.440977 3.819994 32.780666

H 16.030972 4.475158 33.272530

H 10.385809 7.446862 36.237385

O 1.206185 3.384848 25.974789

C 1.817834 2.616254 26.993225

C 0.945098 2.680792 28.218967

C 1.913175 3.286085 24.756868

O 1.056573 1.436470 28.887821

C 0.051826 1.259967 29.865189

H 2.851428 2.951169 27.186352

H 1.871616 1.553813 26.693514

H 48.805283 2.852895 27.867920

H 1.200807 3.522726 28.889290

H 1.292237 3.760345 23.987297

H 2.085767 2.241246 24.452755

H 47.930588 1.319617 29.438354

H 0.129077 1.996293 30.683653

H 0.184815 0.251309 30.281267

H 2.886142 3.803860 24.804785

O 6.176138 28.741144 22.311739

C 6.805878 28.218967 21.147114

C 7.214622 26.771254 21.264946

C 4.789050 28.477610 22.359655

O 8.305420 26.577148 20.387320

C 8.839818 25.270733 20.436701

H 7.731907 28.801769 21.025370

H 6.208897 28.394003 20.234774

H 6.381488 26.089685 21.031727

H 7.511890 26.579103 22.310274

H 4.570499 27.399525 22.439350

H 4.266386 28.886354 21.480074

H 8.128917 24.514360 20.069515

H 9.169845 24.988621 21.450737

H 9.713043 25.265352 19.769804

H 4.403286 28.982674 23.256350

O 12.609453 11.601772 24.723623

C 12.943879 12.858317 24.158911

C 13.035309 13.889466 25.250196

C 12.650523 10.586758 23.740877

O 13.867953 14.947507 24.804785

C 13.810261 16.060307 25.675564

H 12.197775 13.166832 23.404493

H 13.922225 12.823114 23.650913

H 13.455298 13.416183 26.155205

H 12.024695 14.243939 25.508350

H 12.413393 9.641171 24.246918

H 13.650381 10.490928 23.283239

H 14.154955 15.813398 26.693514

H 12.791333 16.481274 25.741570

H 14.471779 16.833302 25.258997

H 11.911753 10.754950 22.937080

O 48.159409 36.356686 12.517535

C 48.291908 35.040001 13.019175

C 47.170307 34.179977 12.520468

C 0.383811 37.112568 12.938013

O 47.455353 32.857426 12.932634

C 46.524433 31.904018 12.469131

H 48.304619 35.022404 14.126107

H 0.342738 34.583344 12.670081

H 47.130703 34.262608 11.418425

H 46.204185 34.533470 12.919434

H 0.266466 38.131985 12.547359

H 1.333794 36.703335 12.551760

H 46.318104 32.005714 11.390066

H 45.575912 31.956333 13.027486

H 46.982555 30.919806 12.636344

H 0.453234 37.162926 14.037612

O 38.498680 10.299268 15.714635

C 38.818924 11.676089 15.587025

C 40.283756 11.853082 15.818776

C 37.144840 10.028402 15.417366

O 40.580048 11.609595 17.184351

C 41.977402 11.558257 17.390190

H 38.259594 12.286271 16.319439

H 38.555885 12.047674 14.581789

H 40.603516 12.872985 15.535687

H 40.821575 11.146091 15.159701

H 36.989357 8.955206 15.600226

H 36.903305 10.241086 14.361771

H 42.486374 12.486243 17.081188

H 42.450684 10.721703 16.849926

H 42.135815 11.411579 18.465832

H 36.453495 10.596047 16.063240

O 9.878790 30.602491 48.017620

C 10.683077 29.816784 47.156128

C 9.911549 28.597887 46.732227

C 10.572579 31.742672 48.481121

O 10.627339 27.951036 45.691788

C 9.941861 26.795698 45.247353

H 11.620352 29.499960 47.647499

H 10.965677 30.385897 46.252102

H 8.908268 28.917645 46.392422

H 9.762915 27.929033 47.600563

H 9.877323 32.302006 0.228820

H 10.896249 32.399303 47.654835

H 8.932714 27.027451 44.862564

H 9.845543 26.038837 46.044792

H 10.531998 26.358597 44.429867

H 11.456072 31.473272 0.193127

O 23.233370 8.442809 15.045293

C 23.268572 7.630210 13.882621

C 23.938402 6.348730 14.310922

C 22.599230 9.694465 14.885413

O 24.408264 5.511196 13.252394

C 23.513037 4.457556 12.938991

H 23.867020 8.101048 13.082246

H 22.261869 7.443929 13.468011

H 23.288618 5.805531 15.016934

H 24.826786 6.647954 14.884436

H 22.716572 10.225440 15.841757

H 21.522121 9.591789 14.677618

H 22.532736 4.832564 12.601629

H 23.358046 3.776968 13.793636

H 23.970673 3.884533 12.121503

H 23.055889 10.303180 14.086993

O 47.639187 9.466623 41.746628

C 46.356239 8.889200 41.922642

C 46.231075 7.646833 41.087063

C 47.741863 10.674765 42.474152

O 44.914394 7.146661 41.237652

C 44.699265 5.962477 40.497417

H 45.560757 9.592767 41.623421

H 46.168980 8.625179 42.980194

H 46.992336 6.910508 41.404865

H 46.438869 7.895698 40.033424

H 48.766167 11.048795 42.339211

H 47.567802 10.533465 43.555172

H 45.360783 5.140100 40.820110

H 44.833721 6.119912 39.413464

H 43.660290 5.652497 40.679787

H 47.035362 11.440426 42.106968

O 36.360107 12.395791 37.984814

C 36.659332 12.199243 39.356747

C 37.541847 10.991101 39.518097

C 35.186680 13.169275 37.840096

O 37.288582 10.484572 40.816685

C 37.728130 9.156155 40.995144

H 35.742592 11.976291 39.931236

H 37.103279 13.109137 39.799229

H 38.614555 11.210141 39.371414

H 37.246044 10.266021 38.739235

H 35.014088 13.304219 36.762985

H 35.282509 14.161799 38.314354

H 38.825771 9.080371 40.981941

H 37.305206 8.472144 40.241219

H 37.368767 8.825150 41.979359

H 34.304653 12.659812 38.265949

O 33.496456 17.288494 16.566347

C 34.891369 17.280670 16.854815

C 35.271751 15.969367 17.496777

C 33.114117 18.605667 16.213341

O 36.425137 16.055416 18.327953

C 37.620564 15.946386 17.573538

H 35.138279 18.066868 17.588696

H 35.495193 17.491888 15.952254

H 35.363674 15.158724 16.757029

H 34.450356 15.694589 18.169052

H 32.023315 18.625223 16.082308

H 33.585442 18.915157 15.267265

H 37.637676 16.625998 16.705204

H 37.783379 14.916215 17.213198

H 38.450764 16.221653 18.237503

H 33.375202 19.343946 16.993670

O 0.385275 4.130953 47.615719

C 48.742210 3.549128 46.440826

C 47.959435 4.603746 45.711346

C 1.131868 3.199056 48.369648

O 47.578560 4.111395 44.440620

C 46.846638 5.078006 43.712608

H 48.086067 2.689593 46.672577

H 0.651741 3.178521 45.778328

H 48.599930 5.497995 45.617470

H 47.077900 4.891236 46.312725

H 1.430603 3.700207 0.408256

H 2.042252 2.874897 47.839649

H 47.445084 5.981545 43.511169

H 45.912785 5.373319 44.223049

H 46.583103 4.622814 42.747952

H 0.543688 2.303340 48.632198

O 42.404724 39.694595 29.546406

C 42.568027 39.368969 28.178387

C 43.219280 40.534084 27.474821

C 41.891350 38.602821 30.282732

O 43.357159 40.339493 26.067196

C 42.230667 40.780994 25.330872

H 41.592125 39.158733 27.705103

H 43.190922 38.466896 28.051266

H 44.238693 40.671474 27.864985

H 42.663368 41.456692 27.712929

H 41.609238 38.984184 31.272324

H 42.639412 37.802937 30.412300

H 42.133369 41.880104 25.355316

H 41.289478 40.330204 25.687790

H 42.388100 40.466125 24.291409

H 40.990257 38.167675 29.820698

O 12.392368 18.333332 23.856752

C 12.314630 18.481478 22.449129

C 13.533527 17.861517 21.824768

C 11.203296 18.785591 24.475735

O 13.581931 18.261950 20.468481

C 14.708421 17.760798 19.782026

H 12.283338 19.548319 22.163595

H 11.403756 18.009663 22.034029

H 13.493924 16.760941 21.927443

H 14.422398 18.212568 22.379213

H 11.316728 18.632557 25.557734

H 10.319314 18.224792 24.130064

H 14.691797 16.660709 19.703310

H 15.657430 18.070778 20.252377

H 14.674196 18.181767 18.767012

H 11.005280 19.854389 24.298744

O 15.302468 37.434284 39.294655

C 16.431402 37.419128 40.152233

C 17.605320 36.788410 39.480446

C 14.141753 37.709061 40.059338

O 18.034109 37.596607 38.396492

C 19.026144 36.904285 37.660168

H 16.214808 36.880817 41.089508

H 16.679777 38.453209 40.420166

H 17.296316 35.790508 39.125484

H 18.427696 36.646130 40.208462

H 13.306664 37.854271 39.361637

H 14.247361 38.619442 40.672455

H 18.660915 35.927898 37.296406

H 19.938972 36.729248 38.255192

H 19.284298 37.521801 36.787922

H 13.895334 36.870548 40.729656

O 24.848785 36.908195 8.726387

C 23.890978 37.940815 8.867687

C 22.674524 37.299828 9.476892

C 26.119024 37.325741 8.272662

O 21.753384 38.268883 9.962397

C 20.742281 37.580959 10.680632

H 23.659714 38.425831 7.903032

H 24.252783 38.724564 9.554142

H 23.040243 36.649067 10.290468

H 22.191952 36.626575 8.745944

H 26.776140 36.447624 8.355290

H 26.550259 38.132473 8.888711

H 21.121691 37.164883 11.629152

H 20.303713 36.750763 10.102719

H 19.937994 38.289417 10.912872

H 26.099466 37.655281 7.220000

O 47.272003 34.786736 20.685566

C 46.996250 35.819843 21.616974

C 47.389347 37.141415 21.019503

C 47.142437 33.508190 21.270813

O 47.028027 38.175495 21.922554

C 47.630386 39.413464 21.602306

H 47.562916 35.684898 22.557671

H 45.924026 35.843311 21.878550

H 46.885750 37.269028 20.044580

H 48.475258 37.126747 20.824421

H 47.432861 32.772842 20.507107

H 46.107864 33.295506 21.585682

H 47.426018 39.723446 20.564800

H 48.723141 39.386082 21.753384

H 47.207462 40.165924 22.283871

H 47.806889 33.381557 22.143549

O 19.432444 45.050800 16.435804

C 18.493212 46.092709 16.216274

C 18.287863 46.962513 17.430283

C 20.761839 45.530441 16.466606

O 16.976068 47.492996 17.387257

C 16.631374 48.088512 18.620335

H 17.536869 45.586178 16.010925

H 18.734743 46.682354 15.313225

H 19.044235 47.764355 17.502645

H 18.426229 46.319572 18.315731

H 21.413090 44.650372 16.550701

H 20.952520 46.187073 17.332987

H 17.349609 48.863464 18.924446

H 16.556568 47.346321 19.434889

H 15.656941 48.570595 18.482944

H 21.022926 46.077553 15.546444

O 27.523224 46.286324 6.168315

C 28.184254 46.089775 7.404814

C 29.549341 45.488880 7.238090

C 26.149334 46.508297 6.418647

O 29.983511 45.191124 8.553796

C 31.292860 44.667973 8.621757

H 27.625410 45.386208 8.043354

H 28.268351 47.041229 7.962193

H 30.234819 46.179737 6.718359

H 29.476980 44.574585 6.620574

H 25.662851 46.736629 5.463282

H 25.978701 47.358543 7.100701

H 32.045807 45.439499 8.408094

H 31.459583 43.821636 7.937257

H 31.438070 44.321812 9.653395

H 25.666277 45.611603 6.842547

O 37.830803 15.396343 2.545848

C 37.159508 15.431545 1.300060

C 37.665058 16.600084 0.501640

C 37.468994 14.268874 3.319821

O 37.059765 16.549234 48.113449

C 37.621056 17.482597 47.211864

H 36.066261 15.520041 1.430603

H 37.351166 14.514805 0.712857

H 38.765144 16.521366 0.436613

H 37.433796 17.547626 1.022837

H 38.084068 14.298699 4.231183

H 37.666523 13.315954 2.800091

H 38.697674 17.302183 47.040741

H 37.488552 18.525482 47.548248

H 37.095943 17.355965 46.254543

H 36.409000 14.289899 3.617578

O 37.903652 4.542630 22.984993

C 36.829967 3.946138 23.690027

C 35.544086 4.412575 23.068111

C 39.151398 4.208203 23.557528

O 34.462090 4.146109 23.944759

C 33.258835 4.627214 23.379559

H 36.832901 4.264430 24.744646

H 36.896461 2.842627 23.681227

H 35.397900 3.933426 22.082922

H 35.638451 5.496040 22.888676

H 39.922436 4.742602 22.984016

H 39.365551 3.127183 23.504236

H 33.021217 4.123619 22.426149

H 33.291107 5.715568 23.196211

H 32.454060 4.418931 24.098282

H 39.216423 4.519650 24.614103

O 23.187899 40.789307 3.734432

C 22.984505 41.203918 2.396725

C 24.260607 41.004925 1.632042

C 22.028162 41.053326 4.494715

O 24.258162 41.891350 0.527064

C 25.537199 41.939266 48.823860

H 22.742975 42.282005 2.354188

H 22.141106 40.664143 1.929310

H 24.386261 39.955685 1.304460

H 25.093252 41.232765 2.320452

H 22.239378 40.784904 5.538576

H 21.162271 40.462700 4.154910

H 25.894114 40.935986 48.545662

H 26.285257 42.396412 0.601381

H 25.455059 42.553848 47.916901

H 21.743116 42.117233 4.452667

O 22.999660 3.988186 24.594545

C 22.222267 5.048182 24.067970

C 21.325573 4.466357 23.017263

C 23.782925 4.358304 25.705389

O 20.390253 5.437857 22.588963

C 19.528761 4.870212 21.621374

H 22.853962 5.844645 23.633801

H 21.586660 5.513152 24.841454

H 20.817575 3.589220 23.460232

H 21.941133 4.102106 22.174841

H 24.289944 3.445964 26.051550

H 23.173231 4.747491 26.537546

H 18.939116 4.036590 22.036474

H 20.076849 4.506938 20.734947

H 18.832041 5.652497 21.295259

H 24.540762 5.110275 25.436966

O 26.715515 2.853384 41.571102

C 25.546486 2.072077 41.740761

C 24.655172 2.230489 40.544353

C 27.634699 2.584473 42.610565

O 23.332623 1.890196 40.912029

C 22.451084 1.990915 39.813408

H 24.985199 2.412371 42.628654

H 25.798286 1.006703 41.883526

H 25.023335 1.602706 39.715622

H 24.698685 3.280707 40.218239

H 28.418451 3.351112 42.553360

H 28.096247 1.587549 42.499088

H 22.679415 1.242366 39.035522

H 22.459396 2.995173 39.355770

H 21.441448 1.796322 40.200150

H 27.167284 2.641189 43.607979

O 8.348935 21.633598 29.261852

C 9.398663 22.489710 28.851151

C 8.823195 23.874353 28.781235

C 8.720520 20.274376 29.329325

O 9.850922 24.820431 28.545082

C 9.312612 26.127335 28.519659

H 9.805940 22.201731 27.863518

H 10.234241 22.482376 29.573299

H 8.300042 24.072371 29.735132

H 8.062912 23.897821 27.980371

H 7.818447 19.710644 29.606544

H 9.488626 20.095919 30.098896

H 8.875510 26.411890 29.492624

H 8.544505 26.243212 27.735907

H 10.134500 26.820635 28.296219

H 9.080859 19.892525 28.358313

O 10.386297 45.994434 29.104416

C 9.230961 45.905937 28.284483

C 9.531651 46.659378 27.010338

C 10.119832 45.809620 30.479282

O 8.411517 47.437260 26.610884

C 8.806082 48.287506 25.546486

H 8.363602 46.379707 28.767057

H 8.966451 44.847897 28.119715

H 9.864611 45.990520 26.194805

H 10.382386 47.323830 27.240623

H 11.091331 45.774418 30.991190

H 9.580544 44.869411 30.677786

H 9.178156 47.727196 24.672285

H 9.593256 0.104143 25.857445

H 7.928945 48.868843 25.231129

H 9.540452 46.650578 30.895849

O 19.172333 15.760105 2.208488

C 18.769457 14.925504 1.130890

C 19.385994 13.567263 1.306904

C 18.643312 17.060654 2.021228

O 19.160599 12.797690 0.139833

C 19.499914 11.438959 0.334427

H 19.105839 15.334249 0.160857

H 17.665945 14.828697 1.077597

H 18.945959 13.091046 2.198709

H 20.467993 13.686073 1.496609

H 19.007074 17.691372 2.845561

H 17.537357 17.064566 2.029540

H 18.918091 10.977411 1.151914

H 20.576534 11.304015 0.536354

H 19.252518 10.905050 48.299240

H 18.976273 17.514380 1.071241

O 8.314709 5.916028 14.502583

C 8.323021 6.577059 13.245060

C 8.279507 5.592847 12.112702

C 8.762079 6.828368 15.488261

O 8.252616 6.314505 10.889893

C 8.844707 5.656408 9.785893

H 9.251006 7.156439 13.116470

H 7.479620 7.285516 13.157053

H 7.387213 4.951374 12.191420

H 9.176201 4.956752 12.193865

H 8.712697 6.323794 16.462206

H 8.135273 7.733374 15.530797

H 8.778213 6.352642 8.937115

H 8.316176 4.725978 9.524806

H 9.911059 5.427101 9.955552

H 9.805940 7.144216 15.306869

O 47.197685 9.488626 29.497515

C 47.469040 10.698234 30.181524

C 46.687737 10.684544 31.466915

C 47.921299 9.398174 28.287907

O 46.604130 12.004160 31.978334

C 46.089775 12.009049 33.293552

H 47.158081 11.573903 29.584055

H 48.549572 10.818021 30.387365

H 47.169327 9.993689 32.183685

H 45.679565 10.282156 31.258635

H 47.632832 8.447208 27.818048

H 0.120276 9.386929 28.454142

H 46.739559 11.457049 33.993698

H 45.075741 11.579282 33.339512

H 46.048706 13.056334 33.619667

H 47.678791 10.221529 27.593142

O 36.185074 20.445990 44.237717

C 36.390423 19.348347 43.366936

C 37.798046 19.415331 42.846714

C 34.899193 20.421057 44.822475

O 37.972103 18.406183 41.867882

C 39.288784 18.415474 41.353043

H 35.691254 19.376705 42.510334

H 36.239342 18.382227 43.883732

H 38.502102 19.290653 43.691093

H 37.970150 20.422522 42.425751

H 34.823895 21.300636 45.478127

H 34.741756 19.518982 45.438034

H 40.047115 18.270262 42.140701

H 39.519070 19.352747 40.817177

H 39.364082 17.579895 40.642632

H 34.095882 20.477772 44.067570

O 5.934608 30.120409 29.792339

C 5.239353 29.086817 29.116152

C 6.075419 28.624779 27.958368

C 5.085340 30.693922 30.766283

O 5.248642 28.013130 26.983448

C 5.951231 27.873785 25.763571

H 4.292299 29.468180 28.696650

H 4.989999 28.261015 29.805052

H 6.883617 27.944679 28.283506

H 6.546257 29.528807 27.544247

H 5.627561 31.526077 31.236143

H 4.807629 29.970308 31.551012

H 6.879705 27.293427 25.886293

H 6.208897 28.850174 25.314249

H 5.294113 27.334007 25.068806

H 4.160288 31.092888 30.316957

O 17.484554 17.096344 18.386627

C 17.547136 16.322372 17.199999

C 16.156137 16.118000 16.669512

C 18.775812 17.297295 18.923958

O 16.228010 15.355272 15.476037

C 14.947017 15.157745 14.908881

H 18.013086 15.339626 17.389702

H 18.154385 16.820101 16.420156

H 15.698010 17.107592 16.489586

H 15.544976 15.610493 17.439083

H 18.660427 17.923611 19.818207

H 19.444178 17.817514 18.215990

H 14.470313 16.104799 14.601346

H 14.265452 14.636059 15.601204

H 15.076584 14.532407 14.013165

H 19.252518 16.348284 19.221714

O 35.837444 14.553431 6.126757

C 36.648087 15.577246 6.668000

C 36.575237 16.757519 5.749793

C 36.147423 13.312531 6.721293

O 37.336498 17.805292 6.314994

C 37.576561 18.844263 5.388475

H 37.708084 15.269221 6.749162

H 36.307304 15.858869 7.680570

H 35.518661 17.045498 5.601648

H 36.966866 16.455360 4.763625

H 35.499104 12.560071 6.251433

H 35.965542 13.308131 7.810625

H 36.642712 19.309233 5.028135

H 38.153984 18.492233 4.515739

H 38.171585 19.604546 5.914562

H 37.198620 13.021620 6.543323

O 31.225876 33.189411 8.623712

C 30.277355 34.138420 9.070592

C 29.787449 33.742878 10.434701

C 31.569103 33.363468 7.264003

O 28.677582 34.569164 10.735392

C 28.048820 34.229359 11.950868

H 29.394350 34.174110 8.408584

H 30.708590 35.155876 9.099928

H 30.594179 33.845062 11.184717

H 29.501915 32.675545 10.403898

H 32.346500 32.621277 7.035184

H 31.973446 34.369194 7.057676

H 28.732344 34.288521 12.813336

H 27.608299 33.218254 11.922998

H 27.240623 34.960308 12.096079

H 30.713480 33.180611 6.591727

O 2.713061 34.880611 23.919334

C 2.737019 34.988178 25.333317

C 1.363620 34.758869 25.871626

C 3.997475 35.101120 23.372225

O 1.029193 33.392315 25.697567

C 48.622913 33.103848 26.166937

H 3.106648 35.982655 25.643784

H 3.411740 34.230824 25.770416

H 0.661031 35.415012 25.326960

H 1.318639 35.041958 26.940422

H 3.894800 35.048801 22.279472

H 4.721089 34.333504 23.696384

H 47.849426 33.672958 25.624228

H 48.519257 33.298443 27.247955

H 48.453255 32.032608 25.991901

H 4.400352 36.095112 23.631357

O 34.738335 8.022330 21.357841

C 34.311501 9.069614 20.506618

C 32.818802 9.048590 20.370207

C 36.150356 8.007663 21.420424

O 32.461887 10.121299 19.520451

C 31.102175 10.131566 19.137131

H 34.754467 8.983563 19.496004

H 34.607300 10.051870 20.909494

H 32.353344 9.153710 21.367132

H 32.506866 8.075624 19.957552

H 36.451050 7.201910 22.099545

H 36.563015 8.955206 21.808144

H 30.424032 10.048449 20.001066

H 30.873848 9.325324 18.418896

H 30.917362 11.097688 18.643312

H 36.603104 7.801335 20.437191

O 48.422939 41.759830 40.047604

C 48.869331 42.508865 38.930401

C 0.784240 43.676918 39.430088

C 47.625011 40.657787 39.667217

O 1.638398 44.146778 38.394047

C 1.971847 45.509418 38.593529

H 48.023975 42.863338 38.309956

H 0.624850 41.904064 38.274750

H 1.387089 43.350311 40.295979

H 0.109031 44.471424 39.788471

H 47.328720 40.142944 40.591778

H 48.171143 39.941017 39.031124

H 2.384502 45.694721 39.601212

H 1.096666 46.166534 38.444897

H 2.734085 45.770996 37.846935

H 46.710712 40.972656 39.136730

O 11.274191 24.455200 7.976371

C 12.352277 25.302023 7.611142

C 12.879340 24.843409 6.292992

C 10.538843 24.972486 9.065703

O 11.950378 25.222816 5.289712

C 12.334188 24.707487 4.033656

H 13.131627 25.290779 8.394404

H 12.025673 26.348330 7.491844

H 13.004507 23.745275 6.334551

H 13.870887 25.284420 6.081286

H 9.852387 24.184334 9.403064

H 9.938439 25.844732 8.765012

H 12.348854 23.604465 4.035123

H 13.323776 25.082006 3.718786

H 11.588572 25.036047 3.297331

H 11.183249 25.261932 9.911059

O 31.807211 37.278313 33.686161

C 31.551012 38.283550 34.650326

C 32.350410 39.511250 34.316875

C 31.137379 36.068707 33.974628

O 32.159729 40.454391 35.357803

C 33.083313 41.522209 35.279579

H 31.847792 37.955482 35.662895

H 30.479282 38.549038 34.692863

H 32.038963 39.921947 33.340000

H 33.410408 39.223270 34.217628

H 31.451761 35.339226 33.214344

H 30.040224 36.177738 33.920357

H 33.002151 42.078609 34.329590

H 34.127174 41.179958 35.395454

H 32.852051 42.206219 36.108799

H 31.405802 35.669743 34.967640

O 18.957205 1.840325 31.119287

C 17.939745 2.083322 30.155123

C 17.676214 0.966122 29.176289

C 20.258242 1.932243 30.566801

O 16.722803 1.491719 28.272261

C 16.366865 0.626317 27.217155

H 18.125050 3.004462 29.571833

H 17.018116 2.254936 30.733034

H 17.302183 0.067472 29.698465

H 18.607622 0.672276 28.661449

H 20.951054 1.518121 31.310947

H 20.539375 2.976105 30.355095

H 15.906294 48.587219 27.580429

H 17.229334 0.371096 26.582527

H 15.636894 1.168049 26.598661

H 20.367273 1.343085 29.642239

O 2.649501 0.082140 7.745108

C 2.465664 47.738930 8.408584

C 3.585798 46.826591 8.017442

C 1.668222 1.038972 8.091759

O 4.866789 47.309650 8.446720

C 5.734636 47.584431 7.357388

H 1.501987 47.282757 8.127939

H 2.451974 47.866539 9.502805

H 3.553529 46.717072 6.923220

H 3.454276 45.822823 8.450143

H 1.853037 1.917576 7.460553

H 1.739117 1.345041 9.147842

H 5.289712 48.297287 6.644043

H 6.000124 46.664753 6.807833

H 6.648932 48.028866 7.771999

H 0.643918 0.684010 7.892765

O 24.900612 0.774948 48.484543

C 26.084309 0.364740 0.260110

C 27.279249 1.088351 48.584286

C 23.832306 48.762257 48.700653

O 28.204300 1.277567 0.748060

C 29.502893 1.630085 0.316336

H 26.232941 48.165276 0.191171

H 26.011946 0.619963 1.331840

H 26.946289 2.061322 48.184834

H 27.726130 0.524621 47.745773

H 22.913122 0.354470 48.341290

H 23.700785 48.513393 0.872737

H 29.518538 2.552202 48.605309

H 29.978621 0.824823 48.624378

H 30.098408 1.799746 1.224765

H 23.975073 47.824493 48.135448

O 34.267986 21.480562 30.425501

C 33.799591 21.305527 29.097572

C 32.873074 22.439350 28.743589

C 35.408657 20.680187 30.657742

O 31.930420 21.990025 27.782354

C 31.318769 23.064690 27.093454

H 33.251995 20.352606 29.004675

H 34.636150 21.264458 28.376404

H 33.465164 23.290085 28.365158

H 32.356277 22.780132 29.656906

H 35.702988 20.829800 31.705513

H 36.256943 20.975988 30.013334

H 32.050697 23.646025 26.503319

H 30.792686 23.756033 27.772577

H 30.584402 22.629055 26.401133

H 35.201347 19.608946 30.496395

O 14.087481 17.836094 38.306042

C 15.296112 17.696749 37.581940

C 15.532754 16.220675 37.375610

C 13.891912 19.159620 38.764168

O 16.887573 15.880870 37.079323

C 17.166752 15.859847 35.688808

H 16.145380 18.098648 38.160339

H 15.259442 18.248749 36.624619

H 14.822830 15.805087 36.638798

H 15.305891 15.726369 38.332443

H 12.929212 19.175268 39.295143

H 13.846440 19.886658 37.934944

H 16.465628 15.217884 35.127033

H 18.172964 15.435946 35.576843

H 17.160395 16.870461 35.246330

H 14.681041 19.475958 39.469200

O 27.109591 42.524513 27.723194

C 25.934208 43.314617 27.802891

C 26.326328 44.758911 27.620522

C 26.902287 41.209297 28.195499

O 25.433546 45.573467 28.358313

C 25.689255 46.945396 28.148073

H 25.452124 43.220257 28.790525

H 25.190058 42.988018 27.054829

H 26.346375 45.040535 26.553679

H 27.354053 44.876743 28.007263

H 27.831736 40.649963 28.018507

H 26.082354 40.700321 27.663057

H 25.473150 47.246090 27.108126

H 26.729694 47.215290 28.393517

H 25.021379 47.504242 28.818882

H 26.689114 41.193649 29.278475

O 42.299118 31.167204 20.090540

C 43.376225 31.735826 19.356171

C 44.533028 30.783396 19.490625

O 44.034321 29.659351 20.201038

C 43.053043 30.342381 20.944698

H 43.031044 31.904509 18.325998

H 43.606998 32.730793 19.770292

H 45.372028 31.227831 20.056805

H 44.960354 30.432346 18.542593

H 43.517036 30.936918 21.762674

H 42.383701 29.612900 21.423357

O 46.942467 25.146545 41.396065

C 47.476376 23.953072 40.832821

C 46.304413 23.139496 40.358070

O 45.152988 23.931559 40.598137

C 45.655117 24.655172 41.694309

H 48.183365 24.237629 40.041248

H 48.068466 23.422585 41.598484

H 46.209076 22.191463 40.916916

H 46.324463 22.873030 39.291229

H 45.674187 24.023478 42.610565

H 44.983334 25.497595 41.905041

O 29.321989 9.710599 33.638733

C 27.952501 9.538496 33.305286

C 27.935879 8.776258 32.005714

O 29.299011 8.544994 31.662001

C 29.823629 8.582153 32.969883

H 27.450373 8.992364 34.123264

H 27.481667 10.531020 33.269592

H 27.445482 9.296966 31.171114

H 27.419573 7.805735 32.090790

H 30.920784 8.645714 32.924900

H 29.574764 7.654657 33.525791

O 7.612609 30.775084 23.609354

C 8.539127 31.416067 22.751287

C 8.401250 32.871117 23.071533

O 7.093856 33.042240 23.623045

C 6.587816 31.731426 23.485655

H 9.538496 30.995100 22.923388

H 8.289286 31.188227 21.698135

H 8.519081 33.540951 22.207109

H 9.125841 33.206032 23.829372

H 6.073952 31.618975 22.508289

H 5.830955 31.558836 24.264030

O 24.003920 13.695850 47.567802

C 23.080824 14.171089 48.537350

C 22.096123 15.036002 47.795643

O 22.567450 15.130855 46.457451

C 23.912491 14.770025 46.661823

H 22.641277 13.299820 0.152543

H 23.630867 14.733845 0.420478

H 22.010073 16.050039 48.220036

H 21.071819 14.637037 47.760445

H 24.500671 15.638851 47.022163

H 24.353014 14.468846 45.700588

O 40.157612 3.395605 37.765774

C 41.257702 4.021922 37.118927

C 42.057095 4.689308 38.205811

O 41.362820 4.428710 39.417377

C 40.755081 3.221058 39.026722

H 41.843437 3.261150 36.574257

H 40.855801 4.697131 36.352287

H 42.176884 5.776684 38.104115

H 43.080425 4.288387 38.284527

H 39.981598 2.947747 39.759136

H 41.492874 2.386946 39.010586

O 4.850655 33.915958 19.878834

C 6.138002 34.037212 20.482660

C 7.088478 33.243679 19.638281

O 6.423047 33.077934 18.400805

C 5.122499 32.924900 18.915157

H 6.386378 35.107964 20.528620

H 6.068574 33.692028 21.522121

H 7.307029 32.249203 20.068539

H 8.061444 33.721855 19.454445

H 4.991955 31.913795 19.354216

H 4.394974 33.015842 18.096203

O 11.128001 40.165924 34.922661

C 10.716813 39.096149 34.076813

C 10.487017 37.907566 34.974487

O 10.701655 38.386227 36.294594

C 10.417100 39.739578 36.060394

H 9.793717 39.391949 33.552681

H 11.470740 38.955338 33.291107

H 11.160270 37.055363 34.802383

H 9.461246 37.500778 34.901146

H 10.734414 40.328247 36.929710

H 9.322879 39.911190 35.944519

O 29.640284 6.141424 36.782055

C 30.002089 7.425838 36.295570

C 30.599560 8.171453 37.458241

O 30.577557 7.273293 38.560772

C 29.492138 6.486608 38.137360

H 30.685612 7.285516 35.446301

H 29.103439 7.925523 35.893181

H 30.021646 9.076460 37.720306

H 31.636574 8.503924 37.303738

H 28.534327 7.024918 38.303596

H 29.460358 5.567912 38.739719

O 44.699753 15.484838 18.099136

C 44.252872 15.677477 16.765341

C 45.343182 16.442160 16.070574

O 46.306858 16.775608 17.062120

C 45.494263 16.639196 18.201811

H 44.029922 14.690331 16.332640

H 43.297020 16.233389 16.774630

H 44.976486 17.367701 15.594358

H 45.862423 15.872069 15.289267

H 44.855721 17.538336 18.335289

H 46.133781 16.561947 19.092150

O 27.979881 42.981171 36.085331

C 27.446463 42.803204 37.389790

C 28.557795 42.226753 38.220478

O 29.670107 42.072742 37.351166

C 28.957739 41.970558 36.143024

H 27.070965 43.771278 37.744751

H 26.569813 42.133369 37.335522

H 28.292307 41.244007 38.646336

H 28.872663 42.852093 39.067791

H 28.497168 40.963364 36.034481

H 29.656906 42.094254 35.305492

O 48.700161 17.263069 35.797352

C 48.439564 17.821915 34.519295

C 47.803471 19.162066 34.781849

O 47.851871 19.359104 36.190449

C 0.014179 18.473656 36.480385

H 0.501640 17.909922 33.968273

H 47.824001 17.111992 33.948715

H 46.754230 19.245184 34.461601

H 48.338352 19.992754 34.289009

H 0.042048 18.281017 37.562870

H 0.997413 18.918579 36.209030

O 28.535793 36.677910 5.097563

C 28.622334 35.488838 5.860780

C 27.595097 34.560856 5.271622

O 27.170218 35.150009 4.041479

C 28.235102 36.057461 3.871821

H 29.646639 35.077652 5.776684

H 28.477123 35.740147 6.920776

H 26.705246 34.419556 5.901850

H 27.997972 33.553661 5.074095

H 27.939791 36.822636 3.141362

H 29.131798 35.544086 3.457699

O 12.807958 48.221500 41.818501

C 13.301286 0.500660 42.449219

C 13.858665 0.060628 43.776657

O 13.634734 47.552158 43.863197

C 12.543448 47.482243 42.982639

H 14.017566 0.974923 41.765205

H 12.475975 1.224767 42.571941

H 13.348712 0.553466 44.621525

H 14.932350 0.246422 43.919910

H 11.618884 47.853336 43.470100

H 12.368412 46.433002 42.712749

O 41.157471 0.260597 0.938742

C 41.092442 0.430258 2.350277

C 42.510822 0.553956 2.831382

O 43.326355 0.520221 1.670178

C 42.469261 48.639534 0.865892

H 40.455368 1.300549 2.562472

H 40.584934 48.449341 2.794713

H 42.788536 48.607754 3.493391

H 42.735237 1.474121 3.388760

H 42.497620 47.569763 1.169516

H 42.814449 48.681580 48.715321

O 41.737335 28.981697 40.533112

C 43.061844 29.204647 40.984386

C 43.262794 28.239992 42.119678

O 42.030697 27.552561 42.293247

C 41.198540 28.549484 41.756409

H 43.745365 29.078503 40.132675

H 43.165497 30.258287 41.306103

H 43.515572 28.762167 43.057442

H 44.049480 27.492908 41.956375

H 41.078751 29.399731 42.465351

H 40.196239 28.132917 41.583817

O 2.113636 24.834608 44.495869

C 0.927007 25.448214 44.971107

C 48.690384 24.570097 44.505646

O 0.393098 23.414761 43.928711

C 1.663822 23.501791 44.527649

H 0.978345 25.519108 46.071686

H 0.898650 26.482784 44.598545

H 48.050377 25.026758 43.739986

H 48.018108 24.257185 45.321671

H 2.367389 22.866673 43.969292

H 1.633508 23.117495 45.570045

O 23.024597 4.485425 18.357290

C 22.852495 3.510014 19.374260

C 23.437740 2.232934 18.837906

O 23.908091 2.523357 17.527092

C 24.118818 3.903112 17.696749

H 23.357557 3.854219 20.293446

H 21.780764 3.459654 19.612858

H 22.726841 1.396867 18.773857

H 24.282610 1.871127 19.449068

H 24.234205 4.373950 16.710093

H 25.056093 4.094283 18.260483

O 24.425865 12.312675 27.351120

C 25.630093 11.563636 27.442062

C 25.375853 10.478705 28.456097

O 24.021032 10.619515 28.851151

C 23.908579 12.001716 28.620869

H 25.877003 11.205252 26.432913

H 26.451494 12.238845 27.737862

H 26.011459 10.575024 29.351324

H 25.526930 9.451957 28.093315

H 24.428310 12.577673 29.418798

H 22.851028 12.295073 28.659492

O 46.455006 8.239415 4.863856

C 46.455494 6.821524 4.848210

C 47.854317 6.409357 5.178236

O 48.490898 7.551493 5.743926

C 47.378101 8.396849 5.914562

H 45.755348 6.441138 5.611916

H 46.076572 6.478785 3.875732

H 48.449829 6.086176 4.312345

H 47.880718 5.580624 5.899894

H 47.724262 9.439733 5.938519

H 46.893085 8.196877 6.891929

O 6.226987 35.797840 12.314630

C 5.738059 35.735256 13.650381

C 4.235583 35.755302 13.558463

O 3.930492 35.969944 12.187509

C 5.071161 35.341671 11.653111

H 6.123823 34.821449 14.133930

H 6.171249 36.578171 14.208247

H 3.748122 36.540524 14.152998

H 3.784303 34.800426 13.886044

H 5.151345 35.585155 10.583824

H 4.982176 34.235714 11.726938

O 33.496944 19.280874 47.821068

C 32.175861 19.105350 48.334934

C 32.182217 17.807735 0.168682

O 33.495968 17.729019 0.692813

C 34.167267 18.201323 48.439072

H 31.963667 19.934082 0.135430

H 31.459583 19.175268 47.506687

H 31.986156 16.952112 48.386761

H 31.456160 17.741241 0.991057

H 34.288521 17.377968 47.708618

H 35.177391 18.526459 48.719719

O 34.244518 31.124668 29.030100

C 33.294529 30.762861 30.015779

C 32.688747 29.486269 29.530273

O 32.969883 29.400219 28.135851

C 33.491570 30.692944 27.921701

H 33.796658 30.690498 30.991190

H 32.541092 31.563723 30.105742

H 31.600883 29.423687 29.685751

H 33.135139 28.607666 30.014801

H 32.668701 31.408245 27.721729

H 34.127174 30.680721 27.025494

O 13.616156 36.965401 24.083614

C 13.248481 35.600803 23.917379

C 11.818368 35.594448 23.442631

O 11.393978 36.947800 23.475389

C 12.649056 37.533047 23.236792

H 13.940315 35.124096 23.201590

H 13.408360 35.097698 24.877634

H 11.121645 35.003330 24.053791

H 11.723026 35.207703 22.411970

H 12.589407 38.609665 23.442631

H 12.941924 37.414726 22.170439

O 34.809227 45.572979 25.231129

C 36.028614 45.035645 24.733400

C 35.704945 44.440620 23.388849

O 34.311989 44.635216 23.176653

C 33.921337 44.741314 24.521206

H 36.782055 45.838467 24.722643

H 36.410469 44.276829 25.440392

H 35.930828 43.359600 23.351690

H 36.232498 44.892879 22.538115

H 33.858265 43.734612 24.984219

H 32.918545 45.182323 24.574011

O 47.610344 27.039675 17.806757

C 47.423080 25.803665 18.470722

C 45.932343 25.632050 18.541128

O 45.362740 26.920864 18.291286

C 46.526390 27.703638 18.413029

H 47.961880 25.017958 17.920189

H 47.891964 25.851089 19.472046

H 45.571999 25.251665 19.510672

H 45.543156 24.945107 17.776443

H 46.748360 27.926588 19.480846

H 46.370907 28.669760 17.912855

O 39.366035 14.216071 28.809105

C 39.063389 13.010373 29.498003

C 38.712341 11.995359 28.441429

O 38.731407 12.699905 27.209330

C 39.703888 13.652826 27.565273

H 39.943951 12.717996 30.094006

H 38.262527 13.221592 30.221619

H 37.725685 11.522566 28.554861

H 39.444756 11.168094 28.392048

H 39.736156 14.444888 26.803034

H 40.719879 13.199589 27.597542

O 47.594208 48.839508 41.452782

C 48.567173 48.106117 40.727703

C 0.404834 0.241042 39.921459

O 0.217082 1.492206 40.585915

C 48.398983 1.067328 41.728050

H 0.347138 47.582474 41.432247

H 48.062599 47.326275 40.139523

H 0.000979 0.339316 38.901066

H 48.893780 0.339316 38.901066

H 1.483405 0.047916 39.820744

H 47.762398 1.894598 42.070786

H 0.212681 0.838514 42.553360

O 25.198372 1.353842 37.395660

C 26.384510 1.020393 36.681824

C 26.226585 1.588038 35.302067

O 25.139700 2.496466 35.383717

C 24.384794 1.783120 36.331749

H 27.233778 1.440871 37.240669

H 26.520433 48.820438 36.691113

H 25.992392 0.807709 34.554008

H 27.107145 2.125859 34.925106

H 23.881199 0.918207 35.854557

H 23.594687 2.432417 36.733158

O 11.471718 46.681866 14.144687

C 12.774221 46.225208 14.523116

C 13.493435 47.405972 15.080006

O 12.449574 48.164295 15.663298

C 11.538701 48.016151 14.600367

H 12.638300 45.462967 15.303935

H 13.253860 45.739216 13.662603

H 14.009254 48.000507 14.300165

H 14.245895 47.168350 15.846156

H 11.825212 48.681580 13.759411

H 10.540798 48.330532 14.935283

O 23.984852 38.181854 43.250568

C 22.811424 37.444550 43.568863

C 22.984016 36.963444 44.983334

O 24.248873 37.448952 45.398430

C 24.845854 37.496376 44.126728

H 21.942110 38.088470 43.407024

H 22.702883 36.607994 42.856495

H 22.982550 35.860912 45.049335

H 22.223244 37.314495 45.695210

H 25.071739 36.471584 43.757587

H 25.806597 38.027840 44.187359

O 8.804615 26.058395 35.654095

C 8.494635 26.423136 34.317856

C 8.227192 25.145567 33.568329

O 8.378270 24.095839 34.512447

C 9.260785 24.751982 35.390076

H 7.641455 27.113501 34.353062

H 9.345859 26.986870 33.895912

H 8.938581 24.993999 32.735195

H 7.220000 25.070271 33.131229

H 10.288023 24.779360 34.966663

H 9.317989 24.184334 36.330772

O 20.731524 44.418129 19.494537

C 21.581770 44.411285 20.630806

C 20.814154 43.732655 21.730404

O 19.505783 43.482323 21.225832

C 19.498938 44.414707 20.171701

H 22.513666 43.900352 20.353094

H 21.859972 45.449280 20.883093

H 20.741793 44.345768 22.642744

H 21.248322 42.771908 22.042830

H 19.256430 45.427277 20.552088

H 18.707851 44.142864 19.460312

O 21.474207 46.561104 5.941453

C 21.256144 45.273754 5.364518

C 22.414904 44.409328 5.775218

O 23.095982 45.129520 6.786809

C 22.833916 46.414425 6.278813

H 20.283178 44.905102 5.719969

H 21.164715 45.375942 4.270297

H 23.108692 44.216202 4.937684

H 22.141594 43.423161 6.172227

H 23.468544 46.623196 5.392387

H 23.098425 47.155640 7.043008

O 20.163391 40.477859 47.492020

C 20.171213 39.330345 48.328087

C 21.593016 38.838974 48.343735

O 22.344009 39.762558 47.568783

C 21.493275 40.872913 47.720348

H 19.825052 39.610989 0.445902

H 19.428043 38.616512 47.947701

H 21.742628 37.834225 47.923256

H 22.010561 38.808659 0.471815

H 21.777342 41.644444 46.992336

H 21.603771 41.333485 48.725586

O 18.622780 5.806509 10.402921

C 19.642195 4.832075 10.256731

C 19.050100 3.533483 10.725124

O 17.693327 3.801904 11.054662

C 17.513401 4.954307 10.265532

H 19.956085 4.801762 9.199181

H 20.517374 5.163080 10.834644

H 19.545874 3.130117 11.617418

H 19.086283 2.736530 9.962397

H 16.612307 5.487239 10.602892

H 17.353521 4.681974 9.199181

O 46.427135 41.595551 45.928917

C 45.067913 41.782806 45.576889

C 45.061073 41.928020 44.080284

O 46.415890 42.096210 43.679359

C 46.957134 42.436016 44.933460

H 44.487560 40.936962 45.969009

H 44.676285 42.685856 46.082928

H 44.469955 42.791466 43.734612

H 44.673351 41.049416 43.548817

H 46.755207 43.502857 45.174503

H 48.047443 42.315739 44.896790

O 46.251610 30.852335 22.756664

C 46.179737 30.656763 24.160866

C 46.975712 29.415373 24.454712

O 47.351208 28.872663 23.196211

C 46.385574 29.501915 22.391436

H 46.543011 31.568613 24.654194

H 45.120720 30.548710 24.455690

H 46.394867 28.658514 25.008667

H 47.893917 29.586987 25.035557

H 45.409187 28.976807 22.469175

H 46.701912 29.440800 21.341217

O 29.945862 12.704306 48.201454

C 28.737720 11.952333 48.102203

C 28.455610 11.755784 46.638355

O 29.601658 12.245201 45.965588

C 29.929728 13.246526 46.898460

H 28.883421 11.015548 48.654202

H 27.926100 12.498466 48.611668

H 27.568205 12.326852 46.305882

H 28.283997 10.713390 46.331306

H 29.212469 14.095304 46.837837

H 30.922739 13.651359 46.656933

O 45.543644 29.273586 27.417614

C 46.800678 29.255007 28.088915

C 47.114079 30.677298 28.460987

O 46.185116 31.472784 27.742264

C 45.110939 30.564846 27.776978

H 46.730270 28.585175 28.960670

H 47.533577 28.800303 27.405882

H 48.130562 31.015148 28.214079

H 46.989891 30.855757 29.544451

H 44.343815 30.893892 27.064610

H 44.636681 30.552624 28.782701

O 35.852112 5.062850 19.326834

C 36.951221 4.168600 19.445154

C 38.181366 4.963597 19.096062

O 37.730576 6.270502 18.765057

C 36.425137 5.925318 18.373915

H 36.950245 3.746166 20.459192

H 36.796230 3.314443 18.762611

H 38.722122 4.539207 18.231636

H 38.920624 5.051115 19.905725

H 36.422199 5.458881 17.366234

H 35.819843 6.839125 18.299109

O 1.426692 29.895014 27.553537

C 2.250047 30.115030 26.418245

C 2.196754 28.840395 25.617870

O 1.356286 27.946636 26.337574

C 1.557236 28.497656 27.616119

H 1.881884 31.007326 25.893627

H 3.272395 30.363407 26.755608

H 3.190744 28.377380 25.498083

H 1.791432 28.952360 24.603346

H 2.549759 28.206257 28.021931

H 0.802820 28.098202 28.307955

O 18.307421 35.349983 13.161453

C 19.014898 36.349350 13.887021

C 18.476589 37.668480 13.416183

O 17.709461 37.380989 12.257915

C 17.234222 36.131290 12.697460

H 18.887777 36.181160 14.967552

H 20.088583 36.215385 13.689006

H 19.251541 38.394535 13.148252

H 17.833160 38.160339 14.170600

H 16.744806 35.607647 11.864816

H 16.466118 36.255966 13.489034

O 33.434853 35.540176 30.302290

C 34.148197 34.340836 30.062716

C 34.198071 33.667091 31.396999

O 34.059212 34.691399 32.380726

C 34.034767 35.827663 31.544167

H 33.642647 33.771236 29.269672

H 35.161747 34.577965 29.684284

H 35.124588 33.106293 31.581326

H 33.363956 32.967926 31.545145

H 35.066895 36.210987 31.397980

H 33.468590 36.625599 32.040916

O 0.516310 16.112133 19.212915

C 0.176504 15.011556 18.386137

C 48.434673 14.007788 19.304344

O 48.731453 14.425332 20.633251

C 0.856599 15.356251 20.351627

H 48.426849 15.367496 17.568649

H 1.092268 14.613568 17.907476

H 48.814571 12.984462 19.155220

H 47.340450 13.950583 19.211447

H 1.831523 14.839454 20.209839

H 0.974923 16.029993 21.212141

O 6.439670 9.263229 16.476385

C 5.447636 8.367024 16.959446

C 4.876079 7.677636 15.749837

O 5.578669 8.191500 14.627258

C 5.939497 9.437778 15.170947

H 5.920429 7.693771 17.687458

H 4.694686 8.940537 17.525623

H 3.798481 7.872230 15.621739

H 4.980710 6.583416 15.742993

H 5.073117 10.133033 15.168992

H 6.719826 9.892969 14.543652

O 13.415696 13.709541 19.485735

C 12.717505 13.670916 18.250216

C 11.517188 12.789378 18.475122

O 11.498609 12.479887 19.864655

C 12.299473 13.541839 20.324736

H 12.431972 14.700598 17.963215

H 13.411783 13.318398 17.474287

H 11.535278 11.842325 17.915300

H 10.568178 13.282707 18.202789

H 12.651501 13.308131 21.336329

H 11.710803 14.482047 20.395144

O 14.001920 47.476864 5.823621

C 14.994443 46.467228 5.745882

C 14.835542 45.845802 4.385684

O 13.604910 46.323483 3.855686

C 13.031886 46.829521 5.035470

H 15.975722 46.927311 5.932163

H 14.834076 45.738235 6.562881

H 14.805717 44.747669 4.407686

H 15.628583 46.114712 3.674294

H 12.543448 46.012035 5.606049

H 12.247646 47.550205 4.766070

O 13.253371 28.386671 42.287380

C 12.492599 27.204443 42.084965

C 12.860272 26.691557 40.720856

O 13.753545 27.645454 40.159081

C 13.432808 28.765589 40.944786

H 12.712128 26.511633 42.912231

H 11.419402 27.450863 42.169060

H 11.988026 26.574213 40.057381

H 13.370225 25.717125 40.720856

H 12.528292 29.276520 40.558044

H 14.258607 29.489693 40.890514

O 8.018419 7.951925 21.245876

C 8.393427 7.734841 19.893013

C 8.896045 9.063258 19.387463

O 8.717586 9.983420 20.457237

C 7.672258 9.308701 21.112888

H 9.122418 6.912953 19.862209

H 7.511890 7.379390 19.329279

H 8.328888 9.416265 18.507881

H 9.955552 9.084282 19.093616

H 6.710047 9.427999 20.563824

H 7.524113 9.747757 22.109814

O 42.982639 12.187018 39.427643

C 43.862709 11.328462 40.133167

C 43.262794 9.989287 39.909725

O 41.851257 10.193171 39.986000

C 41.780361 11.600795 39.878922

H 44.884079 11.455094 39.750332

H 43.881775 11.584660 41.210762

H 43.547348 9.213848 40.630405

H 43.506279 9.617214 38.900089

H 41.513901 12.014916 40.869003

H 40.970211 11.862860 39.186111

O 0.379408 28.643356 20.864025

C 1.295170 28.534327 19.788382

C 2.146883 27.334986 20.105207

O 1.875528 26.990294 21.459049

C 1.263390 28.192566 21.861927

H 0.729970 28.475655 18.847685

H 1.895574 29.462799 19.725311

H 3.225947 27.531048 19.998133

H 1.932732 26.451981 19.485735

H 2.025629 28.965561 22.090256

H 0.712368 28.019976 22.794800

O 39.731266 36.867126 37.517891

C 38.366180 36.551277 37.318409

C 38.346622 35.275665 36.532211

O 39.683838 34.784782 36.521942

C 40.204060 35.546532 37.586342

H 37.879208 36.412422 38.295773

H 37.879208 37.413746 36.845123

H 38.020508 35.398876 35.490791

H 37.684612 34.513916 36.976646

H 41.299747 35.554844 37.521313

H 39.942486 35.086941 38.562241

O 10.283622 30.016266 8.257994

C 11.007236 28.794437 8.179276

C 11.864816 28.715231 9.414309

O 11.735250 29.975199 10.049915

C 10.416121 30.242641 9.641171

H 10.286556 27.964724 8.115227

H 11.563147 28.791012 7.229290

H 12.933123 28.524549 9.235849

H 11.524033 27.924143 10.108586

H 10.184370 31.293837 9.853854

H 9.689575 29.629526 10.217128

O 8.958138 1.600261 14.457112

C 9.495960 1.908286 13.181499

C 8.298574 2.085278 12.289694

O 7.161328 2.165951 13.147762

C 7.831649 2.435839 14.356881

H 10.109564 2.828449 13.254349

H 10.187793 1.109378 12.881297

H 8.129406 1.254589 11.591016

H 8.351379 2.988328 11.663378

H 7.156439 2.239779 15.201260

H 8.121094 3.507081 14.423375

O 3.185855 3.913380 43.479389

C 2.077455 3.197100 42.960636

C 1.242855 4.217982 42.251202

O 1.720049 5.496529 42.662388

C 2.488154 5.099519 43.773235

H 2.438284 2.384991 42.314274

H 1.533767 2.708172 43.789860

H 0.169658 4.141220 42.475616

H 1.329395 4.150999 41.157471

H 1.842770 4.965552 44.667973

H 3.205901 5.895005 44.020145

O 44.314476 26.611862 5.720458

C 43.589886 25.817842 4.785627

C 43.303864 26.697424 3.604866

O 44.172199 27.805826 3.755456

C 44.067081 27.875252 5.156723

H 44.207401 24.940706 4.542630

H 42.673145 25.437456 5.262332

H 42.254135 27.050432 3.599977

H 43.476944 26.230986 2.626032

H 43.068199 28.262484 5.459370

H 44.804871 28.583220 5.549821

O 33.098957 39.897503 15.167035

C 34.130596 39.167534 15.811931

C 33.411873 38.006817 16.397179

O 32.163639 38.532417 16.852371

C 32.157284 39.796295 16.215296

H 34.915813 38.912312 15.086363

H 34.593609 39.793358 16.597639

H 33.915466 37.495888 17.229334

H 33.210922 37.245068 15.625160

H 32.358723 40.578579 16.971668

H 31.151558 39.996265 15.821221

O 48.624378 11.223342 23.214790

C 47.752617 11.393000 22.101013

C 46.527855 12.082388 22.630520

O 46.618797 11.988514 24.043034

C 48.017620 12.121503 24.113440

H 48.274796 11.971401 21.322149

H 47.555580 10.401454 21.665865

H 45.570534 11.650176 22.308317

H 46.505363 13.149230 22.338631

H 48.361824 11.904419 25.134809

H 48.328087 13.160964 23.883642

O 46.779163 9.033434 1.271213

C 47.104298 10.357450 0.851713

C 48.383823 10.726591 1.538167

O 48.481121 9.838209 2.633366

C 47.961391 8.710252 1.967935

H 47.168839 10.379453 48.646381

H 46.262367 11.006746 1.133335

H 48.431740 11.750896 1.924421

H 0.368161 10.602404 0.877137

H 47.744797 7.924057 2.700838

H 48.715321 8.287819 1.269746

O 23.207945 6.771653 20.374607

C 22.538603 7.362278 19.267675

C 23.589310 7.596963 18.213057

O 24.821896 7.152528 18.766523

C 24.487959 7.296762 20.124765

H 21.720625 6.692446 18.964539

H 22.057497 8.301020 19.594769

H 23.676338 8.663316 17.940235

H 23.426006 7.055720 17.270893

H 24.537828 8.362624 20.429855

H 25.224285 6.755029 20.734459

O 37.213776 3.530060 36.192406

C 37.923702 2.595230 35.400829

C 37.464108 2.869519 34.005432

O 37.061234 4.238517 33.981960

C 37.384903 4.608146 35.304024

H 39.012543 2.753643 35.516216

H 37.724220 1.584616 35.770950

H 36.588436 2.264225 33.730164

H 38.225857 2.700838 33.230480

H 36.747341 5.448124 35.609116

H 38.431206 4.973864 35.355850

O 29.917992 0.562267 6.182983

C 29.946350 48.672783 4.997822

C 31.390644 48.555439 4.599346

O 32.146038 0.347138 5.594314

C 31.212185 0.269888 6.645998

H 29.479425 47.690525 5.187037

H 29.311724 0.275757 4.251718

H 31.617996 0.116855 3.624423

H 31.728981 47.506687 4.535296

H 31.484028 0.998389 7.422416

H 31.238098 48.160385 7.122214

O 46.831966 18.238970 39.416885

C 46.722446 19.651484 39.518097

C 45.280113 19.981508 39.230118

O 44.624458 18.740610 39.005211

C 45.744102 18.016018 38.555885

H 47.082787 19.946306 40.515018

H 47.413792 20.119387 38.794971

H 45.168633 20.616138 38.331955

H 44.748646 20.503685 40.038803

H 46.002747 18.292751 37.511536

H 45.501106 16.943800 38.547573

O 38.771011 30.008934 1.079553

C 40.141476 29.683308 1.257034

C 40.443634 29.906260 2.714039

O 39.229137 30.332113 3.316888

C 38.617977 30.878248 2.174752

H 40.293045 28.651669 0.906961

H 40.751659 30.321358 0.594536

H 41.214676 30.682676 2.865607

H 40.795174 29.015923 3.255282

H 39.027210 31.883482 1.943978

H 37.546246 31.011236 2.375212

O 5.742948 5.848557 20.969145

C 5.843179 4.835009 21.960201

C 5.720946 5.531242 23.290085

O 5.658853 6.919798 23.002106

C 5.048182 6.800011 21.739695

H 5.040848 4.093794 21.799833

H 6.787787 4.297188 21.799343

H 6.544790 5.362562 23.993652

H 4.809585 5.225663 23.832796

H 5.071650 7.771022 21.225342

H 3.978896 6.518388 21.844814

O 3.190744 23.527216 20.971100

C 4.351948 24.342747 21.048840

C 5.531242 23.412317 20.925140

O 4.982666 22.117147 20.744236

C 3.778435 22.334719 21.434114

H 4.285454 25.107430 20.260687

H 4.343147 24.886925 22.010073

H 6.173205 23.412806 21.825256

H 6.199118 23.620602 20.078806

H 3.947116 22.367479 22.534203

H 3.100292 21.495230 21.239521

O 36.874462 40.266155 21.680044

C 37.462154 39.544495 20.607338

C 38.323154 38.484989 21.237566

O 38.226833 38.681049 22.641768

C 37.903164 40.049561 22.615366

H 36.664223 39.164597 19.960485

H 38.033222 40.238773 19.970753

H 39.384129 38.555393 20.944698

H 38.018551 37.455307 21.005323

H 38.798882 40.664143 22.377745

H 37.564827 40.365406 23.612288

O 42.810535 11.269301 28.604733

C 43.283329 12.141060 29.619745

C 44.450397 12.862718 29.009565

O 44.812206 12.129814 27.841026

C 44.073925 10.953454 28.066912

H 42.454105 12.782534 29.947817

H 43.584019 11.547991 30.505196

H 45.322159 12.929212 29.679396

H 44.222557 13.892889 28.697628

H 44.630325 10.264066 28.740166

H 43.943378 10.425411 27.111546

O 4.639926 1.856949 15.587025

C 3.400983 1.207163 15.871581

C 2.315074 2.070610 15.309314

O 2.903254 3.348668 15.139166

C 4.190602 2.917922 14.772470

H 3.426407 0.188237 15.458925

H 3.319821 1.097643 16.963356

H 1.440382 2.170351 15.968388

H 1.938110 1.703914 14.336348

H 4.891236 3.759856 14.865368

H 4.200380 2.606475 13.709052

O 14.109484 40.675877 4.581744

C 13.181499 40.729656 3.512459

C 12.173818 41.783787 3.894800

O 12.629498 42.350456 5.112720

C 13.987741 42.024342 4.958708

H 13.724209 40.980476 2.582518

H 12.772756 39.722466 3.363825

H 11.155869 41.409756 4.056635

H 12.086789 42.590519 3.148207

H 14.510405 42.190083 5.908695

H 14.469824 42.687325 4.208692

O 4.768515 0.558845 18.951826

C 3.783814 0.316336 19.948261

C 4.434088 0.627784 21.269346

O 5.760550 1.052662 20.975500

C 5.541999 1.497097 19.659306

H 2.909122 0.961232 19.753180

H 3.433741 48.172119 19.838253

H 4.494226 48.671803 21.967535

H 3.905557 1.432070 21.809610

H 6.510076 1.615418 19.153265

H 5.057471 2.494511 19.658817

O 31.316328 33.981960 48.662514

C 30.392252 33.769279 0.833624

C 29.051613 34.205891 0.325136

O 29.344479 34.898701 48.017620

C 30.394697 34.060680 47.605942

H 30.433323 32.716125 1.154359

H 30.727169 34.361858 1.687291

H 28.493746 34.875233 0.995457

H 28.379826 33.352222 0.110986

H 30.886070 34.487514 46.719517

H 30.022621 33.052513 47.310627

O 8.456010 11.825212 4.857500

C 8.286840 12.902321 3.950538

C 8.908757 12.447618 2.658302

O 9.591789 11.229209 2.941391

C 9.676863 11.352908 4.341681

H 7.216088 13.138963 3.895289

H 8.778213 13.804882 4.358793

H 9.623081 13.178566 2.247113

H 8.188077 12.247158 1.852059

H 10.513418 12.025673 4.630637

H 9.893947 10.365762 4.774871

O 38.340263 10.222507 43.759056

C 37.946190 9.279365 44.742290

C 36.465225 9.458312 44.929550

O 36.077019 10.552044 44.111084

C 37.335522 11.174938 44.004498

H 38.233189 8.277551 44.394661

H 38.516769 9.462712 45.670761

H 36.189964 9.677841 45.974876

H 35.874599 8.580687 44.631794

H 37.567760 11.753340 44.924660

H 37.312538 11.888773 43.171364

O 41.250854 35.591026 29.293631

C 40.069115 34.832207 29.098549

C 40.477859 33.394760 29.268698

O 41.800411 33.406006 29.799671

C 41.843925 34.741268 30.244106

H 39.309322 35.148056 29.837320

H 39.651085 35.084007 28.115805

H 40.501328 32.813915 28.336800

H 39.814873 32.832981 29.945372

H 42.889740 35.040001 30.396652

H 41.343262 34.842964 31.229298

O 31.833124 4.468802 47.109188

C 32.423260 3.178521 47.073498

C 32.086388 2.610387 45.721123

O 31.507986 3.670871 44.965729

C 31.966112 4.753358 45.738235

H 33.515038 3.274840 47.218708

H 32.051674 2.600119 47.931076

H 31.364241 1.781654 45.743614

H 32.974281 2.230978 45.186726

H 31.367664 5.642229 45.493771

H 33.021709 4.996356 45.493282

O 31.774942 31.250811 48.271374

C 30.817621 30.241661 48.544682

C 31.454693 28.979738 48.059666

O 32.862804 29.206114 48.048420

C 32.916588 30.498840 48.605801

H 30.602491 30.214773 0.736813

H 29.873501 30.498350 48.042553

H 31.157427 28.744076 47.029984

H 31.234676 28.090380 48.667892

H 33.812794 31.014168 48.232258

H 33.020241 30.428923 0.813090

O 0.726547 14.702065 36.899395

C 48.273815 14.404307 36.547855

C 48.003437 13.011841 37.050964

O 0.288468 12.607985 37.733997

C 1.201785 13.382449 36.997181

H 47.630386 15.183659 36.981049

H 48.162338 14.490359 35.452656

H 47.802002 12.302406 36.227608

H 47.156128 12.922856 37.746220

H 1.374866 12.944368 35.989990

H 2.174263 13.387338 37.508598

O 4.331413 8.647181 10.002488

C 5.668631 8.164120 10.009823

C 5.624628 6.806366 9.355637

O 4.264919 6.574126 9.020232

C 3.884533 7.920145 8.884311

H 6.312060 8.873554 9.457824

H 6.030927 8.170964 11.048306

H 5.960521 5.971766 9.985865

H 6.236766 6.764318 8.435964

H 2.788356 7.981750 8.821728

H 4.287899 8.355780 7.943613

O 35.448746 11.322595 48.623890

C 35.800774 10.584803 0.891314

C 37.134567 9.951152 0.599913

O 37.521313 10.409277 48.204880

C 36.755161 11.587105 48.170166

H 35.853577 11.270768 1.756720

H 34.982800 9.882702 1.104001

H 37.130657 8.852041 0.581338

H 37.906097 10.247931 1.332331

H 36.711647 11.953800 47.136082

H 37.231865 12.386012 48.778877

O 33.891510 13.497835 3.844441

C 33.537529 14.877102 3.858131

C 32.867695 15.149434 2.543403

O 32.577763 13.876265 1.981136

C 33.727230 13.225502 2.475442

H 32.901432 15.051648 4.739179

H 34.445465 15.483373 4.008721

H 33.544373 15.697522 1.864771

H 31.942154 15.740059 2.600608

H 34.632236 13.532060 1.907797

H 33.620155 12.140082 2.340987

O 33.664162 1.957179 34.513916

C 33.124382 0.649785 34.396572

C 32.072208 0.737792 33.331688

O 32.302006 1.963046 32.640831

C 33.576641 2.286716 33.145405

H 33.921337 48.828262 34.119839

H 32.777245 0.334427 35.387142

H 31.041061 0.761750 33.713051

H 32.114258 48.795502 32.615406

H 33.746788 3.365291 33.017307

H 34.366261 1.767964 32.562115

O 45.917183 28.739189 1.365087

C 44.707088 28.526991 0.652719

C 43.737057 29.557652 1.160718

O 44.456757 30.357052 2.084298

C 45.748013 30.120899 1.582169

H 44.899727 28.614021 48.463032

H 44.388794 27.489977 0.814063

H 42.869698 29.133265 1.683380

H 43.332710 30.209393 0.368651

H 46.487762 30.472437 2.313608

H 45.926472 30.700766 0.651251

O 19.348347 1.028703 39.014988

C 19.706244 0.876160 37.654789

C 20.467014 48.478676 37.570206

O 20.262154 47.808846 38.809147

C 19.186024 48.558372 39.321545

H 18.783146 0.850736 37.052917

H 20.265577 1.770895 37.349209

H 21.551456 48.606777 37.435261

H 20.131121 47.821556 36.751740

H 19.145441 48.434185 40.412834

H 18.225769 48.177498 38.916222

O 17.589672 47.159061 33.660736

C 18.392982 48.014194 32.852539

C 19.119040 48.874706 33.814262

O 18.134829 0.276246 34.795052

C 17.602386 47.860184 34.886971

H 19.017344 47.405972 32.187107

H 17.728529 48.625843 32.218399

H 19.500893 0.914296 33.382050

H 19.974665 48.340309 34.272385

H 16.572214 47.924721 35.256111

H 18.178831 47.266624 35.622314

O 37.929077 3.778435 48.553482

C 37.470955 4.111885 0.964166

C 35.967499 4.051746 0.911851

O 35.635517 3.699229 48.468899

C 36.820679 3.014241 48.145226

H 37.904629 3.400983 1.687779

H 37.880676 5.097075 1.225254

H 35.453148 4.992444 1.151425

H 35.546532 3.301731 1.601239

H 36.866146 2.865118 47.056877

H 36.836811 2.004605 48.609711

O 47.915920 1.793388 18.136786

C 48.208790 1.636442 19.517517

C 47.279339 2.556116 20.259708

O 46.499008 3.214212 19.272074

C 47.403526 3.102737 18.199856

H 48.108559 0.574001 19.776159

H 0.376963 1.887751 19.683752

H 47.827915 3.305153 20.854246

H 46.593861 2.058876 20.959854

H 48.225414 3.842974 18.304976

H 46.881348 3.333511 17.260138

O 40.942341 28.819370 47.080830

C 41.383842 27.495844 46.842724

C 41.957355 27.555002 45.465412

O 42.449219 28.882444 45.293312

C 42.076653 29.453022 46.526390

H 42.139236 27.213243 47.601540

H 40.538975 26.802053 46.955669

H 41.176537 27.388279 44.706108

H 42.770931 26.847034 45.261040

H 41.868858 30.519863 46.379707

H 42.925922 29.385063 47.234844

O 11.056618 15.085874 43.613354

C 11.799299 14.881990 44.809761

C 11.309882 13.587310 45.400875

O 10.220551 13.170253 44.590721

C 10.644940 13.759411 43.387470

H 12.874942 14.863411 44.565300

H 11.645776 15.760105 45.454166

H 10.958832 13.658692 46.440826

H 12.086300 12.801112 45.393055

H 9.811318 13.759411 42.670212

H 11.464873 13.164875 42.929344

O 2.935035 37.510067 37.518867

C 1.781654 38.090916 38.097755

C 2.122925 39.541073 38.241501

O 3.546684 39.632015 38.195053

C 3.864487 38.262527 38.260082

H 0.913806 37.870895 37.462639

H 1.578748 37.619099 39.078060

H 1.762097 39.988441 39.177799

H 1.720538 40.162014 37.428905

H 3.887955 37.927612 39.319099

H 4.873145 38.104115 37.853783

O 35.927406 30.485149 34.574543

C 35.126053 29.445688 35.113831

C 35.230198 29.564987 36.611416

O 36.170891 30.596136 36.868591

C 36.847080 30.577068 35.635029

H 34.110550 29.554720 34.707531

H 35.503994 28.473700 34.748112

H 35.592491 28.639936 37.088612

H 34.290962 29.829496 37.119411

H 37.578030 29.741001 35.595425

H 37.422058 31.505541 35.538219

O 14.167177 25.077116 37.126259

C 13.813193 23.834263 37.713947

C 12.340543 23.921291 38.002419

O 11.884373 25.126009 37.399570

C 12.937036 25.316204 36.486740

H 14.054724 23.018730 37.006470

H 14.448800 23.674871 38.596954

H 12.098523 23.971649 39.073170

H 11.765563 23.073490 37.591717

H 12.911121 26.352242 36.119068

H 12.816269 24.652727 35.603249

O 11.624752 2.786401 47.073498

C 12.119547 1.509321 47.466106

C 10.953454 0.569112 47.416237

O 9.801539 1.389533 47.317474

C 10.385809 2.398681 46.529808

H 12.935568 1.210586 46.789433

H 12.572295 1.616396 48.461567

H 10.846378 48.803326 48.287018

H 11.010658 48.800392 46.533234

H 9.719399 3.272884 46.503895

H 10.509995 2.054964 45.479103

O 43.167942 3.744699 1.372909

C 43.250568 5.151345 1.152890

C 42.822758 5.387987 48.627800

O 42.136791 4.209670 48.243015

C 42.963570 3.310531 0.047426

H 42.603718 5.647607 1.891661

H 44.273895 5.487728 1.373888

H 43.682781 5.551289 47.950146

H 42.150482 6.241655 48.483566

H 43.936047 3.184877 48.417072

H 42.483444 2.325341 0.061117

O 44.002541 32.930279 23.432364

C 43.363514 33.899822 22.610964

C 43.261814 35.159302 23.426985

O 43.735096 34.823895 24.722153

C 44.627392 33.808392 24.335901

H 43.967339 34.041611 21.699602

H 42.400322 33.482765 22.282404

H 42.248756 35.574398 23.524281

H 43.883732 35.972878 23.012375

H 44.937862 33.246613 25.228197

H 45.549019 34.240116 23.888042

O 13.367291 21.040039 46.219341

C 13.528149 19.827007 45.491817

C 12.880808 18.739143 46.304905

O 12.208043 19.394796 47.366364

C 13.062201 20.506128 47.485664

H 13.078335 19.973686 44.498806

H 14.602812 19.653439 45.320202

H 13.618112 18.031176 46.726360

H 12.144972 18.129450 45.759750

H 13.991163 20.223530 48.019085

H 12.568382 21.278147 48.092422

O 7.120258 12.226133 43.518017

C 6.470473 11.255611 44.329147

C 5.051115 11.161248 43.849995

O 4.965064 12.018339 42.723995

C 5.984968 12.924323 43.070644

H 7.031274 10.319314 44.225002

H 6.547235 11.555325 45.388653

H 4.326524 11.484429 44.617123

H 4.734779 10.154546 43.537571

H 5.634895 13.637179 43.844131

H 6.256811 13.518371 42.186172

O 46.430557 48.539791 28.187189

C 45.832108 0.822866 27.641054

C 46.483852 1.048262 26.319971

O 46.766941 48.631222 25.863314

C 47.215290 48.108559 27.093945

H 44.754513 0.621427 27.526157

H 45.936253 1.646709 28.360758

H 47.418682 1.633997 26.408955

H 45.859978 1.554791 25.571424

H 48.270393 48.399960 27.267513

H 47.183018 47.012871 27.047987

O 41.866413 25.003286 29.879366

C 42.933254 25.944965 29.943905

C 42.969925 26.652443 28.615978

O 42.071278 25.939585 27.783823

C 41.164314 25.557734 28.791012

H 42.755775 26.627508 30.791218

H 43.849506 25.388563 30.180548

H 43.953651 26.678356 28.124605

H 42.635990 27.705103 28.689808

H 40.468082 24.807228 28.389116

H 40.551197 26.425091 29.120552

O 7.893253 4.823763 44.365818

C 6.645020 5.206105 43.793770

C 6.804900 6.623019 43.323421

O 8.031620 7.074299 43.869553

C 8.706830 5.840245 43.831905

H 6.393222 4.512316 42.975304

H 5.869580 5.071650 44.562855

H 6.005503 7.311918 43.628021

H 6.855259 6.694402 42.224312

H 9.627970 5.914073 44.428398

H 9.013388 5.591870 42.792446

O 23.161009 41.532967 27.595097

C 22.141106 40.791260 28.243416

C 20.856201 41.540791 28.017530

O 21.189161 42.673634 27.228401

C 22.354765 42.158306 26.627995

H 22.118124 39.775761 27.811691

H 22.419794 40.674900 29.300966

H 20.368740 41.892818 28.938181

H 20.100319 40.941360 27.482153

H 22.915567 42.986061 26.177204

H 22.098568 41.452782 25.807087

O 8.527882 35.944519 42.496643

C 8.869642 34.912392 43.400669

C 7.870274 33.819637 43.165009

O 6.879217 34.334969 42.281025

C 7.142749 35.705433 42.475616

H 9.914481 34.624413 43.239815

H 8.826617 35.302067 44.432312

H 7.379879 33.512100 44.101307

H 8.295153 32.912678 42.709324

H 6.664577 36.062840 43.411427

H 6.701247 36.291168 41.663021

O 47.427971 27.627853 47.417702

C 48.410717 28.223858 48.257683

C 48.570595 27.320318 0.556398

O 47.492020 26.398199 0.488438

C 47.414280 26.338551 47.975082

H 0.453234 28.337778 47.688080

H 48.068954 29.241318 48.499702

H 48.542236 27.826359 1.531811

H 0.635607 26.765873 0.525600

H 46.485809 25.833490 47.675369

H 48.252304 25.739126 47.566338

O 32.789467 4.519162 3.604866

C 32.156796 3.704607 4.581255

C 30.772150 3.422985 4.062503

O 30.677298 4.055658 2.793246

C 31.636087 5.061382 3.008863

H 32.785065 2.815736 4.734779

H 32.147018 4.239495 5.546399

H 29.989376 3.828306 4.727445

H 30.537952 2.359078 3.922669

H 31.212185 5.874959 3.635180

H 31.910864 5.509241 2.044208

O 12.492109 43.083847 48.265503

C 12.517045 41.876682 0.119297

C 11.678534 40.885624 48.253773

O 11.163204 41.591148 47.138523

C 12.194353 42.536247 47.007004

H 13.564329 41.552525 0.242022

H 12.142037 42.105503 1.122576

H 10.827311 40.473949 48.812614

H 12.261335 40.017780 47.897831

H 11.858949 43.332710 46.331795

H 13.102293 42.089855 46.548878

O 22.256491 3.195633 44.525204

C 21.309927 2.984905 43.490631

C 20.066584 3.705585 43.932625

O 20.329626 4.201358 45.239529

C 21.339750 3.286574 45.584225

H 21.744583 3.340845 42.545536

H 21.141735 1.898996 43.363514

H 19.183090 3.044066 43.969292

H 19.789850 4.555831 43.294086

H 20.914873 2.287205 45.829666

H 21.858992 3.638602 46.486294

O 36.919930 39.763046 30.752106

C 37.118927 40.925720 29.954662

C 38.573975 40.933052 29.566454

O 39.102016 39.708286 30.044624

C 38.254704 39.577744 31.158403

H 36.424156 40.874382 29.104906

H 36.828011 41.814587 30.540888

H 39.129887 41.766186 30.033869

H 38.772480 40.999546 28.486900

H 38.532417 40.301357 31.957312

H 38.369602 38.571041 31.585726

O 42.829601 23.184477 15.444258

C 42.625717 23.945738 14.264475

C 43.087269 25.346518 14.580812

O 43.511658 25.332827 15.940518

C 42.727905 24.239584 16.368332

H 43.165009 23.460722 13.437696

H 41.556435 23.889511 14.004854

H 42.287380 26.099466 14.465424

H 43.926758 25.696587 13.961828

H 41.665955 24.545652 16.512077

H 43.099491 23.889999 17.341299

O 11.091821 29.540051 4.233627

C 11.026304 30.879713 4.691753

C 10.498261 31.669333 3.531038

O 9.930127 30.733524 2.618209

C 9.939417 29.579655 3.426407

H 10.355006 30.933496 5.569379

H 12.023717 31.183340 5.041825

H 11.279569 32.220352 2.988817

H 9.731133 32.404678 3.816083

H 9.922793 28.693228 2.776622

H 9.023655 29.541519 4.054680

O 46.575279 47.621586 19.170378

C 45.530441 48.087536 20.009378

C 44.728111 0.154501 19.172823

O 45.204815 0.026403 17.837559

C 45.874161 47.685638 17.951969

H 44.930527 47.224575 20.350161

H 45.981236 48.518280 20.914873

H 44.833721 1.209117 19.462757

H 43.646114 48.840485 19.189936

H 46.583103 47.588829 17.117857

H 45.165211 46.835880 17.865917

O 11.752362 14.016588 15.328871

C 12.145949 12.999617 14.414086

C 10.965677 12.078477 14.274742

O 10.023024 12.503844 15.246731

C 10.357450 13.869909 15.199794

H 12.431972 13.468988 13.455787

H 13.052911 12.519001 14.807673

H 11.181294 11.012125 14.433643

H 10.509507 12.162084 13.271951

H 9.859722 14.396974 16.025593

H 9.993689 14.331946 14.255674

O 10.622938 48.336887 35.452168

C 9.894925 48.481121 36.662266

C 8.683850 47.595673 36.531231

O 8.757678 47.019226 35.235573

C 9.530673 48.022995 34.625881

H 10.569157 48.237637 37.496864

H 9.615258 0.649298 36.787922

H 7.738264 48.159897 36.624619

H 8.620778 46.774273 37.257782

H 8.908757 0.029335 34.423950

H 9.899815 47.661678 33.655361

O 19.291632 8.141629 7.297250

C 18.027752 8.737144 7.563227

C 18.262438 9.778071 8.626157

O 19.664684 9.797139 8.857420

C 19.915504 8.454054 8.519570

H 17.628788 9.125352 6.614707

H 17.326141 7.952903 7.896187

H 17.741730 9.525784 9.568810

H 17.941214 10.796019 8.363113

H 19.566898 7.775911 9.326791

H 20.998478 8.297597 8.419340

O 43.326843 35.181305 9.860211

C 43.378670 33.794212 10.132544

C 43.251060 33.690559 11.622307

O 43.584995 34.969597 12.159150

C 44.047523 35.610584 10.992080

H 42.583183 33.294529 9.560498

H 44.338924 33.384003 9.764380

H 43.909645 32.936146 12.072122

H 42.231155 33.449520 11.954778

H 45.131966 35.420387 10.850779

H 43.927738 36.696980 11.112356

O 10.588225 4.487870 24.048901

C 9.199181 4.397907 23.757011

C 8.693628 3.163364 24.453245

O 9.837231 2.526291 24.993509

C 10.608759 3.682117 25.199839

H 8.706341 5.318069 24.110996

H 9.080859 4.390574 22.663767

H 8.180743 2.439751 23.805414

H 7.992017 3.401472 25.274155

H 11.645776 3.387293 25.414478

H 10.241575 4.249762 26.081863

O 29.811407 13.872844 33.408936

C 30.376608 12.748797 34.068012

C 31.455673 12.227600 33.155186

O 31.478651 13.096425 32.028694

C 30.937407 14.237094 32.652077

H 30.777040 13.068068 35.045380

H 29.564009 12.044742 34.294388

H 31.303127 11.203296 32.788490

H 32.450642 12.233467 33.636292

H 30.634272 14.963641 31.883972

H 31.696712 14.739713 33.291595

O 40.390343 38.824306 6.094488

C 39.986977 39.178288 4.779271

C 41.141335 39.929771 4.172512

O 42.198887 39.889679 5.122988

C 41.776451 38.766121 5.858335

H 39.752777 38.259102 4.211626

H 39.045300 39.741531 4.849188

H 40.939896 40.985855 3.940760

H 41.486519 39.472134 3.229369

H 42.304005 38.749500 6.822501

H 42.043407 37.828850 5.323937

O 42.194973 9.891013 20.453325

C 43.030064 8.745456 20.521286

C 42.458996 7.891787 21.620884

O 41.254765 8.523481 22.042830

C 41.009327 9.261763 20.871359

H 43.019798 8.228169 19.544409

H 44.067081 9.076460 20.678232

H 43.111713 7.789601 22.498022

H 42.226753 6.866505 21.284990

H 40.249043 10.025469 21.080130

H 40.597649 8.609044 20.070005

O 0.911851 24.058680 11.652132

C 2.247113 24.368172 11.260012

C 2.690571 23.266127 10.339360

O 1.502965 22.595808 9.949196

C 0.835089 22.734663 11.178850

H 2.240268 25.362162 10.786730

H 2.866096 24.470358 12.163062

H 3.355513 22.543003 10.844912

H 3.225947 23.602999 9.439733

H 1.257523 22.030117 11.925443

H 48.668385 22.468685 11.052707

O 29.123974 5.218328 27.643990

C 27.872807 4.890258 28.229725

C 26.915485 4.688331 27.087587

O 27.662567 4.890258 25.892649

C 28.935247 4.569521 26.409935

H 27.987217 3.982807 28.851151

H 27.593142 5.703345 28.914713

H 26.069153 5.389453 27.086613

H 26.478874 3.673316 27.079767

H 29.703842 4.913237 25.704901

H 29.066769 3.468944 26.513098

O 0.680588 20.999458 20.088583

C 0.805264 19.649038 20.515419

C 0.911851 19.686197 22.016916

O 0.849757 21.057152 22.381657

C 0.128588 21.524567 21.270323

H 48.817505 19.077971 20.170725

H 1.672134 19.213404 20.002045

H 1.841303 19.265230 22.429573

H 0.083118 19.147886 22.508778

H 0.185793 22.621721 21.240988

H 47.946724 21.256144 21.356863

O 30.644541 44.627880 40.338028

C 30.084229 45.721123 41.047951

C 30.378075 46.952244 40.232906

O 31.153513 46.512699 39.128906

C 30.634272 45.206772 39.057034

H 29.004187 45.539242 41.149647

H 30.496395 45.721615 42.067364

H 30.935452 47.740887 40.756546

H 29.456934 47.431885 39.853989

H 31.257166 44.600010 38.385735

H 29.607035 45.209705 38.629711

O 47.885605 20.763306 8.599265

C 47.614742 19.972708 7.454196

C 47.598118 18.549438 7.938235

O 48.089005 18.566063 9.275454

C 48.706028 19.832386 9.260296

H 46.668179 20.310558 7.014161

H 48.394093 20.156546 6.690491

H 48.226391 17.876184 7.332453

H 46.592884 18.103537 7.955836

H 0.816022 19.779581 8.783102

H 48.862976 20.162413 10.294868

O 27.326185 46.041370 10.535420

C 26.112667 46.773785 10.500707

C 25.135788 45.922562 9.743846

O 25.747925 44.646950 9.582500

C 26.770763 44.748158 10.546666

H 25.783129 46.976692 11.535767

H 26.315083 47.749687 10.038670

H 24.889370 46.308815 8.746922

H 24.166733 45.805222 10.250375

H 27.557938 44.018188 10.306602

H 26.386467 44.491470 11.555813

O 31.704536 44.571655 14.997376

C 30.288113 44.568722 15.045293

C 29.922392 43.614334 16.148314

O 31.132488 43.001705 16.582972

C 31.911840 43.253994 15.439857

H 29.944395 45.603783 15.188548

H 29.892078 44.247982 14.063525

H 29.221272 42.834980 15.811442

H 29.457912 44.088104 17.022028

H 31.670799 42.525490 14.636549

H 32.975262 43.118561 15.686766

O 37.241158 37.390770 3.796037

C 36.033993 38.034687 3.396583

C 35.156857 36.959534 2.855829

O 35.610584 35.774860 3.489479

C 36.982025 36.075554 3.359424

H 35.605202 38.523125 4.284476

H 36.251568 38.830662 2.671503

H 35.270287 36.863216 1.760141

H 34.087082 37.100834 3.057756

H 37.304718 35.945984 2.305784

H 37.577538 35.372471 3.960317

O 3.393649 24.460091 29.206604

C 4.236072 23.723276 28.323109

C 5.148412 24.740246 27.752529

O 5.501418 25.521063 28.884399

C 4.240473 25.549911 29.522451

H 4.813496 22.978148 28.900532

H 3.610244 23.179588 27.604385

H 4.636015 25.363630 26.996161

H 6.060751 24.340303 27.294895

H 3.709008 26.473494 29.240828

H 4.404263 25.581202 30.609337

O 38.580330 33.014374 34.102238

C 37.421082 33.376183 33.357113

C 37.907074 34.081703 32.124035

O 39.308834 33.874401 32.101055

C 39.517117 33.866085 33.490589

H 36.852947 32.457485 33.156162

H 36.774231 34.007874 33.985386

H 37.700260 35.168102 32.154839

H 37.487576 33.713543 31.175514

H 39.451111 34.897724 33.898354

H 40.531155 33.501347 33.708164

O 34.011299 16.080353 20.869402

C 32.686790 15.800197 20.431812

C 31.892773 17.055763 20.628361

O 32.786045 18.002817 21.186716

C 33.973648 17.470863 20.652319

H 32.723461 15.460392 19.384039

H 32.267292 14.966086 21.006302

H 31.036661 16.929621 21.307970

H 31.492342 17.454241 19.683264

H 34.830742 17.942190 21.150047

H 34.062637 17.711416 19.569832

O 11.639909 29.440800 29.499960

C 10.713879 29.695042 30.536976

C 11.140714 31.012213 31.113911

O 12.485754 31.244944 30.686588

C 12.753687 29.957108 30.185438

H 9.693975 29.652016 30.134100

H 10.770106 28.886354 31.293837

H 11.092798 30.989723 32.206177

H 10.536887 31.878595 30.808819

H 13.042643 29.279943 31.020525

H 13.610289 30.002579 29.497025

O 2.770755 13.075890 41.029858

C 3.817061 13.522771 40.182549

C 4.515739 12.279428 39.698997

O 3.779902 11.168582 40.202595

C 2.556116 11.830102 40.412834

H 3.385337 14.106551 39.349903

H 4.446311 14.226337 40.747261

H 5.554222 12.173818 40.039780

H 4.549964 12.227111 38.599400

H 1.920020 11.218452 41.065548

H 2.008027 11.951845 39.455021

O 20.701700 4.332391 14.766114

C 19.967329 5.452036 14.293322

C 20.143833 6.535989 15.325936

O 21.025860 6.011370 16.306726

C 20.668453 4.662417 16.132668

H 20.335005 5.703834 13.288574

H 18.909779 5.159168 14.167177

H 19.187490 6.806856 15.807530

H 20.561867 7.479132 14.950439

H 19.663706 4.471246 16.567326

H 21.373976 4.017521 16.670488

O 3.731009 44.825405 1.911708

C 2.914011 43.756611 1.448694

C 3.352090 42.526470 2.195287

O 4.331902 42.959660 3.128161

C 4.807629 44.071480 2.408948

H 1.863794 44.040676 1.610529

H 3.040643 43.656868 0.359362

H 3.797504 41.771076 1.523988

H 2.556116 42.010647 2.750709

H 5.475016 43.749767 1.581193

H 5.409988 44.700733 3.079269

O 33.016819 25.735212 28.228745

C 32.308849 25.904871 29.451557

C 33.309196 25.716145 30.559465

O 34.485069 25.226240 29.938040

C 34.335457 25.885803 28.702030

H 31.829214 26.895931 29.450577

H 31.487942 25.174414 29.461824

H 33.011440 24.995466 31.333441

H 33.543396 26.657820 31.088976

H 35.022404 25.439411 27.970594

H 34.603878 26.960466 28.790037

O 11.863349 31.425848 12.982994

C 10.448880 31.489897 13.064156

C 10.141345 32.746441 13.827861

O 11.389089 33.275948 14.262518

C 12.136660 32.085899 14.195536

H 10.034269 31.463005 12.048653

H 10.071917 30.580978 13.566285

H 9.506717 32.567982 14.710377

H 9.626992 33.515526 13.234792

H 11.914686 31.427803 15.066316

H 13.208390 32.326942 14.242962

O 38.759766 44.504669 37.855247

C 37.617630 44.545742 38.705494

C 36.694046 43.452007 38.243462

O 37.444061 42.673634 37.324276

C 38.248837 43.709187 36.810898

H 37.968681 44.433777 39.742512

H 37.169773 45.549019 38.644871

H 35.796375 43.846085 37.733505

H 36.327347 42.786091 39.037479

H 37.671902 44.332569 36.094620

H 39.089794 43.270618 36.254013

O 23.580507 6.382466 31.208275

C 22.661812 5.378208 30.809309

C 23.025087 5.040848 29.389950

O 24.287987 5.651030 29.127398

C 24.679617 5.945364 30.447500

H 22.755688 4.509383 31.486473

H 21.640442 5.762994 30.951097

H 22.311251 5.414877 28.642380

H 23.106249 3.957872 29.209049

H 25.438923 6.740361 30.424522

H 25.154858 5.059427 30.919806

O 26.454916 8.810482 13.269506

C 26.126356 9.948707 14.054724

C 27.422993 10.657164 14.339280

O 28.446320 9.846520 13.782880

C 27.678703 9.273986 12.754665

H 25.416922 10.578935 13.490990

H 25.587557 9.601079 14.947995

H 27.647409 10.809220 15.404165

H 27.458685 11.655555 13.866976

H 28.235102 8.431563 12.321474

H 27.501221 10.002488 11.935710

O 7.085055 4.370527 35.951851

C 8.168031 5.269666 35.793438

C 9.267141 4.408175 35.274197

O 9.054458 3.108115 35.829132

C 7.831160 3.309554 36.504341

H 7.867340 6.081286 35.116276

H 8.421296 5.733659 36.766895

H 10.283622 4.742113 35.520618

H 9.213359 4.323590 34.177532

H 8.028687 3.511481 37.575584

H 7.240046 2.384502 36.451538

O 28.618423 17.985704 6.861616

C 29.484312 18.568996 5.905761

C 29.715088 17.507534 4.873634

O 28.717676 16.514521 5.091207

C 27.862049 17.216133 5.961010

H 29.011522 19.474979 5.483816

H 30.393232 18.906845 6.417180

H 30.698322 17.020561 4.955774

H 29.641748 17.874229 3.838085

H 27.247955 16.495453 6.520344

H 27.160929 17.858095 5.385542

O 16.316017 12.472553 45.802776

C 15.711701 12.669102 47.070076

C 15.902873 11.386643 47.834270

O 16.714493 10.546177 47.023628

C 17.279205 11.528433 46.190002

H 16.186939 13.531082 47.573181

H 14.666862 12.953658 46.889172

H 14.975375 10.836600 48.041576

H 16.396200 11.543591 48.809193

H 17.681103 11.040972 45.290867

H 18.122606 12.035452 46.701912

O 10.463548 15.344027 26.815744

C 10.726591 15.782597 28.151009

C 9.700332 16.818146 28.434587

O 9.615746 17.490911 27.192707

C 9.536541 16.327261 26.401623

H 10.743704 14.912304 28.817905

H 11.733783 16.229475 28.170565

H 9.957019 17.531492 29.226160

H 8.718564 16.384466 28.709850

H 9.721355 16.575638 25.348959

H 8.509792 15.909717 26.445625

O 30.911983 31.543680 25.907806

C 31.428780 32.856941 25.779217

C 30.520840 33.452454 24.771536

O 30.300335 32.426193 23.798571

C 30.721792 31.287479 24.533916

H 31.420467 33.354179 26.758541

H 32.480465 32.830536 25.436480

H 30.888025 34.357948 24.271364

H 29.553741 33.693497 25.245796

H 31.662977 30.903671 24.097794

H 29.972754 30.492971 24.422932

O 31.747562 27.526648 25.231617

C 32.553314 27.724661 24.076281

C 31.608706 28.030243 22.945391

O 30.315981 28.115314 23.526237

C 30.540398 27.211775 24.582323

H 33.268616 28.529926 24.296299

H 33.155186 26.817211 23.897333

H 31.609684 27.243559 22.168484

H 31.813566 28.973873 22.424683

H 30.554089 26.163025 24.210247

H 29.714600 27.284140 25.303978

O 36.842678 37.056343 10.951009

C 37.358498 38.333908 11.325039

C 36.516079 38.813061 12.465219

O 35.319668 38.060600 12.377212

C 35.925941 36.846592 11.999270

H 38.424850 38.237103 11.576838

H 37.302761 38.989563 10.443013

H 36.268681 39.883324 12.439306

H 36.996693 38.621887 13.445030

H 35.156368 36.135693 11.664845

H 36.437847 36.388466 12.871029

O 2.651945 27.851784 31.246410

C 1.595861 26.904728 31.360819

C 0.577424 27.515402 32.284893

O 1.168049 28.703495 32.777245

C 1.928821 28.996365 31.631197

H 1.188584 26.701824 30.356071

H 2.025140 25.955721 31.713823

H 0.287001 26.891041 33.139538

H 48.531971 27.775023 31.760763

H 2.624565 29.814829 31.859037

H 1.280013 29.350838 30.800997

O 29.232517 13.390760 2.278404

C 27.825869 13.600999 2.377168

C 27.517847 13.779458 3.840040

O 28.756788 13.654292 4.519650

C 29.596277 14.053746 3.466988

H 27.553047 14.492804 1.784098

H 27.312984 12.749775 1.907308

H 26.819166 13.042154 4.261497

H 27.084656 14.772960 4.056147

H 30.630360 13.795103 3.729543

H 29.567432 15.154323 3.317376

O 41.362331 7.730930 15.236952

C 40.390831 6.973580 15.942964

C 40.501816 7.390147 17.386280

O 41.515854 8.382670 17.438595

C 42.242401 7.958770 16.310148

H 39.410530 7.161817 15.481905

H 40.600090 5.899405 15.794331

H 40.787842 6.554080 18.048777

H 39.585567 7.817470 17.814581

H 42.831562 7.046430 16.545813

H 42.963081 8.737144 16.023148

O 27.135014 43.670559 32.335255

C 26.352732 44.143841 31.254721

C 25.919540 45.522621 31.657110

O 26.753162 45.915230 32.746929

C 27.728085 44.901680 32.661858

H 26.972691 44.152153 30.338959

H 25.532309 43.434895 31.072351

H 24.875189 45.572487 31.997892

H 26.012436 46.259922 30.847445

H 28.239502 44.806828 33.629932

H 28.499613 45.174988 31.911352

O 24.097305 36.536610 36.735115

C 24.146198 37.856228 36.204632

C 22.911654 38.560284 36.695023

O 22.333252 37.703194 37.667503

C 22.738085 36.482338 37.096924

H 25.091784 38.311420 36.533676

H 24.207314 37.803913 35.106010

H 22.181686 38.737274 35.884380

H 23.090113 39.540096 37.160484

H 22.107368 36.232010 36.217831

H 22.597273 35.677078 37.831295

O 8.496101 36.954643 46.475536

C 8.508326 37.809780 47.605453

C 7.338320 38.722607 47.413303

O 6.969179 38.629223 46.042347

C 8.102026 37.951569 45.557331

H 8.462365 37.205952 48.519749

H 9.468091 38.362759 47.641632

H 7.557360 39.770382 47.661678

H 6.464117 38.430229 48.007839

H 8.930758 38.668823 45.366650

H 7.854628 37.495888 44.590721

O 5.685744 47.680260 2.297473

C 6.608351 48.759811 2.360544

C 7.136882 48.773502 3.770613

O 6.477807 47.711548 4.447289

C 5.317092 47.668526 3.653759

H 7.365211 48.608246 1.577771

H 6.084220 0.805262 2.102391

H 6.920287 0.830202 4.292299

H 8.220835 48.618511 3.865954

H 4.636504 48.516811 3.891378

H 4.759225 46.747875 3.875243

O 37.780441 35.595913 22.628077

C 37.380501 34.369194 23.218212

C 38.617489 33.517971 23.306221

O 39.683838 34.317368 22.813379

C 39.117172 35.580269 23.064199

H 36.942421 34.575520 24.210247

H 36.573772 33.948227 22.605097

H 38.590599 32.591450 22.715595

H 38.835552 33.208477 24.343235

H 39.689709 36.341530 22.515623

H 39.183178 35.843800 24.142775

O 40.866066 23.399117 3.328622

C 39.870609 23.049532 2.380101

C 40.425056 23.412317 1.032127

O 41.678177 24.031788 1.278058

C 41.979359 23.354624 2.472998

H 39.649616 21.974380 2.475931

H 38.945557 23.575619 2.651945

H 39.808517 24.109039 0.447858

H 40.578579 22.522957 0.396032

H 42.826672 23.847462 2.965837

H 42.289825 22.304895 2.272537

O 13.795103 41.742718 37.749153

C 14.178423 42.814934 38.604286

C 13.871376 42.370499 40.008488

O 13.256794 41.096844 39.886745

C 13.885066 40.704235 38.694252

H 15.251131 43.021751 38.453697

H 13.646958 43.722385 38.286972

H 13.193721 43.028599 40.571735

H 14.787627 42.275158 40.618671

H 13.378536 39.813408 38.296753

H 14.946529 40.423588 38.875641

O 26.145424 5.254998 35.702011

C 24.941193 5.930208 35.388119

C 25.265352 6.761874 34.181934

O 26.489630 6.265612 33.648026

C 26.583504 5.067739 34.378483

H 24.146198 5.187037 35.186192

H 24.610678 6.503231 36.266235

H 25.417898 7.825293 34.413685

H 24.486004 6.719337 33.407471

H 27.629810 4.740157 34.396084

H 25.998257 4.268342 33.876842

O 27.695816 10.624405 8.017930

C 28.690294 9.805451 7.429750

C 28.198921 9.631392 6.037772

O 27.568693 10.864470 5.682321

C 27.553537 11.519143 6.935932

H 29.677439 10.305135 7.474731

H 28.779280 8.873066 8.001796

H 27.440105 8.834929 6.000124

H 28.960670 9.383995 5.287267

H 26.606972 12.067232 7.041541

H 28.365648 12.273560 6.962335

O 12.193865 33.117538 8.838351

C 13.388315 33.841640 9.102372

C 14.301633 33.591797 7.929924

O 13.533037 32.867207 6.983358

C 12.720928 32.196884 7.915744

H 13.113538 34.894791 9.251495

H 13.812705 33.496456 10.061649

H 15.181704 32.983574 8.207146

H 14.695220 34.494358 7.441484

H 13.291018 31.395044 8.435964

H 11.893662 31.707958 7.386235

O 14.536318 31.963667 33.034420

C 15.561111 31.982246 34.017654

C 16.147825 33.370800 33.980007

O 15.439369 34.066547 32.971348

C 14.244918 33.337067 33.051533

H 15.127921 31.733871 35.003822

H 16.259300 31.168182 33.782967

H 17.217112 33.430450 33.737011

H 16.022169 33.911556 34.934395

H 13.618600 33.581039 32.183197

H 13.671894 33.616734 33.963383

O 25.878471 48.862484 44.260696

C 25.651119 0.578890 45.521152

C 24.276741 1.186631 45.455147

O 23.795147 0.933366 44.145309

C 24.530985 48.652248 43.913555

H 25.731791 48.701138 46.306370

H 26.464207 1.295169 45.705479

H 24.246428 2.269113 45.640453

H 23.573662 0.731923 46.173382

H 24.477203 48.397026 42.848183

H 24.094860 47.799068 44.477291

O 3.031842 10.759838 37.120880

C 4.264430 10.534443 36.453495

C 4.872168 9.319457 37.106701

O 3.931470 8.862309 38.073311

C 2.779067 9.428487 37.493443

H 4.061525 10.383364 35.379807

H 4.866789 11.453138 36.513142

H 5.825577 9.500360 37.622032

H 5.068717 8.507835 36.385532

H 1.961090 9.411375 38.225368

H 2.438773 8.829551 36.620708

O 44.286121 14.479603 6.632308

C 44.638149 15.650585 7.353477

C 45.720146 15.234018 8.309820

O 45.962654 13.855730 8.067801

C 44.687531 13.522281 7.577406

H 44.932484 16.423580 6.628886

H 43.742920 16.038794 7.871252

H 45.418476 15.366029 9.363949

H 46.672089 15.766950 8.185633

H 43.949249 13.450898 8.407117

H 44.732513 12.534158 7.096301

O 35.525997 29.295588 47.190842

C 36.826057 29.584545 47.670479

C 37.645988 28.357336 47.382500

O 36.733650 27.348188 46.959579

C 35.553375 27.917788 47.470997

H 37.181507 30.513018 47.200130

H 36.760052 29.790871 48.753944

H 38.197010 27.997482 48.265995

H 38.395515 28.488855 46.590439

H 35.467815 27.732973 48.563751

H 34.685040 27.436686 47.002605

O 16.140003 0.868336 2.720884

C 15.842734 2.232445 2.477398

C 14.536318 2.229023 1.721515

O 14.023922 0.902561 1.827124

C 14.805229 0.466926 2.908633

H 15.765484 2.758043 3.448409

H 16.699825 2.674925 1.952778

H 14.621880 2.489132 0.656630

H 13.782392 2.919389 2.124392

H 14.778826 48.265503 2.971704

H 14.402352 0.858069 3.871821

O 37.946190 28.228258 31.308014

C 38.056198 29.642239 31.215609

C 36.711647 30.146322 30.766283

O 35.884872 29.001251 30.598581

C 36.554214 28.138783 31.483541

H 38.349068 30.045115 32.201286

H 38.884445 29.871546 30.529642

H 36.721916 30.701254 29.816784

H 36.246185 30.817621 31.509455

H 36.234943 27.105680 31.286015

H 36.279926 28.370535 32.534737

O 17.948057 16.250011 44.364349

C 17.989616 17.330542 45.280598

C 18.571440 16.772186 46.551811

O 18.734743 15.374830 46.341084

C 17.798935 15.212505 45.301136

H 18.566551 18.146074 44.820518

H 16.965311 17.720217 45.422878

H 17.911388 16.927176 47.423080

H 19.551743 17.187284 46.830013

H 16.761919 15.186592 45.701565

H 17.974461 14.249318 44.801937

O 31.393089 20.241129 4.483959

C 31.926022 19.968309 3.188788

C 33.379112 20.280245 3.265061

O 33.720875 19.937506 4.596412

C 32.578739 20.496349 5.206105

H 31.770540 18.898535 2.977082

H 31.352509 20.535954 2.446596

H 33.587887 21.352465 3.080735

H 34.004452 19.699886 2.573228

H 32.704395 21.590572 5.325404

H 32.475086 20.070494 6.210363

O 44.863056 31.308014 6.264146

C 45.519684 32.572872 6.360464

C 45.542664 33.167896 4.982666

O 44.595612 32.423260 4.241450

C 44.828831 31.184317 4.860922

H 44.965729 33.168873 7.099235

H 46.531765 32.435974 6.768230

H 46.537636 33.067665 4.507916

H 45.279621 34.233761 4.926438

H 45.778328 30.739391 4.493737

H 44.018188 30.497862 4.582233

O 8.109848 12.732175 9.284253

C 8.110337 12.719461 7.861473

C 8.372892 14.131974 7.413615

O 8.677005 14.871234 8.586064

C 7.928945 14.110951 9.503782

H 8.867687 11.989982 7.538292

H 7.142260 12.332231 7.502600

H 7.492821 14.583744 6.922243

H 9.211892 14.246873 6.711515

H 6.853793 14.378394 9.437288

H 8.256527 14.348570 10.525153

O 40.089649 46.053593 29.540051

C 39.285854 46.350376 30.677298

C 38.555885 45.085026 31.030794

O 38.751945 44.220112 29.925327

C 40.058849 44.647926 29.630013

H 39.931728 46.724892 31.485006

H 38.620911 47.183018 30.410343

H 37.477795 45.208237 31.191650

H 38.948494 44.604412 31.946556

H 40.375183 44.215717 28.673182

H 40.779530 44.281719 30.394697

O 0.764195 43.793278 10.820954

C 48.608730 44.285629 11.645776

C 48.193142 45.606228 11.056618

O 0.173572 45.838467 9.960930

C 0.429279 44.490978 9.647038

H 47.778042 43.560551 11.646753

H 0.084583 44.322792 12.680836

H 48.253773 46.467716 11.736716

H 47.152706 45.579334 10.685032

H 1.272193 44.437687 8.945427

H 48.447872 44.023075 9.139042

O 33.943336 1.183206 27.117413

C 35.236061 1.354331 27.694347

C 36.206097 0.660053 26.780540

O 35.398876 48.712383 25.974789

C 34.349636 0.747571 25.843267

H 35.426254 2.428505 27.814623

H 35.214062 0.939231 28.711807

H 36.980068 0.061605 27.281204

H 36.750763 1.367043 26.125866

H 33.505745 0.269399 25.333803

H 34.648861 1.611018 25.206684

O 25.347494 5.182148 1.411535

C 26.321438 5.294113 0.382342

C 27.623453 4.830120 0.978345

O 27.370190 4.629659 2.358588

C 26.022215 4.254163 2.223155

H 26.003633 4.676596 48.417072

H 26.320948 6.336018 0.030314

H 28.469788 5.520975 0.875181

H 27.960814 3.879155 0.533420

H 25.546976 4.239495 3.212746

H 25.938120 3.224480 1.809034

O 16.426025 46.594349 47.131191

C 16.369797 47.437748 48.267948

C 15.217394 48.363781 48.014194

O 14.907904 48.262573 46.625637

C 16.045639 47.559982 46.180717

H 16.272991 46.809967 0.272336

H 17.327608 47.982903 48.366222

H 15.434479 0.521200 48.254745

H 14.311412 48.101715 48.580376

H 16.881706 48.262081 45.974388

H 15.812908 47.058830 45.229752

O 8.710741 44.455288 33.215324

C 8.823195 43.874931 34.514893

C 8.303464 42.481487 34.398041

O 7.358855 42.545048 33.341469

C 8.112293 43.391869 32.503445

H 9.867545 43.947781 34.847855

H 8.219368 44.480225 35.208195

H 7.814047 42.093281 35.303047

H 9.108239 41.768631 34.136463

H 7.458107 43.809414 31.725559

H 8.899467 42.810047 31.981270

O 17.346188 40.581024 45.897137

C 16.524300 39.641792 46.582615

C 15.567957 40.437279 47.423573

O 15.764016 41.795033 47.064697

C 17.125681 41.697735 46.727337

H 16.036348 39.002766 45.833088

H 17.161861 38.973915 47.185463

H 15.765484 40.313580 48.504589

H 14.509427 40.187439 47.269070

H 17.753464 41.635643 47.639679

H 17.428816 42.609585 46.194893

O 30.756502 20.313492 9.962397

C 31.016127 21.668310 10.282644

C 32.267292 21.943089 9.545831

O 32.065365 21.339262 8.271684

C 31.095819 20.360918 8.595842

H 30.195215 22.321518 9.929639

H 31.085062 21.763651 11.371488

H 33.120960 21.452694 10.048449

H 32.510288 23.003084 9.405996

H 31.478161 19.375729 8.301997

H 30.190815 20.556978 7.995929

O 24.994976 45.834064 24.282610

C 26.088709 45.106052 23.743320

C 25.760639 43.651489 23.939871

O 24.425865 43.606022 24.428797

C 24.000498 44.878212 24.003920

H 26.195786 45.356873 22.673546

H 27.009848 45.449280 24.233227

H 26.400158 43.125404 24.663485

H 25.824688 43.075535 23.001617

H 23.078379 45.146145 24.538807

H 23.752121 44.865986 22.920456

O 3.686028 40.357094 43.283329

C 2.915967 39.163132 43.285774

C 2.011450 39.223270 42.086922

O 2.328275 40.437279 41.425404

C 2.799602 41.155514 42.538200

H 3.611711 38.314354 43.292130

H 2.347343 39.115707 44.227940

H 0.942164 39.230606 42.367077

H 2.136126 38.397472 41.371132

H 1.956690 41.509987 43.168919

H 3.333022 42.046341 42.185196

O 5.943408 12.165017 18.345556

C 5.421234 12.770800 17.165773

C 6.583905 13.426940 16.471497

O 7.661502 13.382936 17.393614

C 7.309962 12.167951 18.006729

H 4.932305 11.997804 16.551189

H 4.625748 13.467521 17.470375

H 6.420602 14.473247 16.174227

H 6.877261 12.885698 15.553778

H 7.903521 12.040830 18.922981

H 7.553448 11.312816 17.341299

O 48.742210 25.817842 23.426985

C 47.809826 25.691700 22.368944

C 46.452560 25.682899 23.017752

O 46.664753 25.621296 24.425865

C 47.985348 25.136278 24.394573

H 47.977528 26.506252 21.651688

H 48.014683 24.752468 21.820366

H 45.863403 24.809673 22.700928

H 45.829666 26.562969 22.805557

H 47.992683 24.045479 24.194603

H 48.450317 25.281979 25.374872

O 26.699379 4.974843 14.637037

C 27.224001 4.661439 15.916073

C 28.647757 5.151345 15.890160

O 28.801769 5.826066 14.648771

C 27.453308 6.142403 14.433154

H 26.620663 5.173836 16.690046

H 27.091990 3.584331 16.085732

H 29.415863 4.366616 15.943941

H 28.866796 5.859802 16.708626

H 27.326185 6.479763 13.397116

H 27.119858 6.973580 15.099074

O 15.036491 11.190095 43.356667

C 15.699967 11.278591 42.111366

C 16.287169 12.652966 42.080563

O 15.556223 13.432319 43.026154

C 14.550497 12.510201 43.377693

H 16.432871 10.465014 42.033627

H 14.967552 11.122622 41.298283

H 16.226542 13.132117 41.091465

H 17.345699 12.672036 42.382233

H 13.684606 12.608476 42.692215

H 14.187224 12.748797 44.385860

O 29.657394 5.451058 32.742039

C 30.074938 4.200380 33.268616

C 29.243273 3.154563 32.579227

O 28.353912 3.859597 31.723604

C 29.166023 4.986088 31.511896

H 31.152536 4.076682 33.071091

H 29.961018 4.234117 34.362347

H 28.651669 2.519935 33.253948

H 29.853943 2.464197 31.970512

H 28.568062 5.780107 31.044971

H 29.999153 4.749935 30.813219

O 43.426094 46.706314 46.717560

C 42.673145 47.324810 45.682007

C 42.336273 46.245255 44.688019

O 43.001217 45.072315 45.134407

C 43.014908 45.375942 46.509762

H 41.781830 47.798580 46.124977

H 43.274529 48.147182 45.268375

H 42.660435 46.454025 43.658825

H 41.251835 46.045773 44.627880

H 43.717499 44.702198 47.019226

H 42.014561 45.201393 46.960068

O 9.613791 47.767776 44.751091

C 8.342089 47.916901 45.362740

C 8.332312 47.003582 46.558659

O 9.606458 46.379707 46.588970

C 9.896392 46.472118 45.216549

H 8.207634 0.088494 45.590580

H 7.560783 47.655811 44.629349

H 7.556871 46.222763 46.477982

H 8.168520 47.500332 47.526245

H 9.325324 45.717701 44.637169

H 10.961765 46.256989 45.060093

O 20.342827 39.834919 14.775893

C 19.308256 40.788818 15.009112

C 19.965374 42.135815 15.051159

O 21.355885 41.885483 15.195882

C 21.416513 40.688587 14.456622

H 18.539661 40.694454 14.226337

H 18.810528 40.518440 15.953232

H 19.635349 42.763107 15.888694

H 19.789360 42.726929 14.133442

H 22.361610 40.174725 14.680062

H 21.426292 40.901272 13.370225

O 34.505604 15.616849 30.489059

C 33.598152 16.632353 30.093517

C 33.078911 16.219696 28.742121

O 33.576641 14.906436 28.495213

C 33.870487 14.551475 29.828030

H 32.787510 16.702271 30.843534

H 34.121307 17.598963 30.117476

H 33.392315 16.868505 27.911432

H 31.976870 16.204540 28.707407

H 34.536896 13.676783 29.830965

H 32.943478 14.251761 30.362917

O 5.905272 2.566872 6.029949

C 5.929230 3.554018 7.052786

C 4.496182 3.933914 7.312896

O 3.713897 3.172165 6.407890

C 4.600812 2.092123 6.258768

H 6.406913 3.126695 7.949969

H 6.578038 4.377861 6.721293

H 4.258074 4.993422 7.154972

H 4.182779 3.700696 8.346978

H 4.279098 1.475096 5.408032

H 4.578811 1.442827 7.159862

O 31.645374 18.354845 13.386847

C 30.725212 17.299250 13.155585

C 29.394840 17.792578 13.661138

O 29.594812 19.151310 14.034189

C 30.727169 19.409462 13.244082

H 30.695389 17.071409 12.074566

H 31.122711 16.398643 13.637669

H 28.991964 17.261114 14.533873

H 28.606201 17.737818 12.890587

H 31.194582 20.349184 13.568240

H 30.438213 19.542940 12.180174

O 35.829132 38.975384 35.572933

C 35.152454 37.767242 35.909317

C 35.141209 36.926285 34.667931

O 35.442879 37.811245 33.602554

C 36.349350 38.622379 34.311012

H 35.661430 37.292004 36.761517

H 34.148689 38.038109 36.268188

H 34.183403 36.433449 34.454754

H 35.904915 36.127865 34.700199

H 36.545902 39.540096 33.737988

H 37.319874 38.095802 34.426395

O 41.353527 11.081064 5.899894

C 40.399143 10.430301 5.067250

C 39.515160 11.507898 4.504982

O 40.162498 12.733641 4.807140

C 40.722813 12.332231 6.034838

H 40.949188 9.870478 4.298166

H 39.853497 9.682730 5.665209

H 38.508457 11.509365 4.963597

H 39.362125 11.451182 3.418096

H 39.940041 12.293605 6.823479

H 41.468430 13.072467 6.356553

O 12.134704 7.923078 2.301873

C 10.777928 7.511401 2.468597

C 10.102230 7.723595 1.143603

O 11.147558 8.008640 0.227352

C 11.971401 8.695584 1.137246

H 10.779395 6.464117 2.805469

H 10.317848 8.096647 3.283151

H 9.402085 8.580197 1.172938

H 9.526762 6.864549 0.767617

H 11.544568 9.692509 1.383666

H 12.955125 8.864754 0.678143

O 29.285809 34.341816 20.663565

C 29.886213 35.551422 21.120222

C 31.209740 35.639919 20.407366

O 31.369131 34.381905 19.772247

C 30.010399 34.223003 19.461779

H 29.213936 36.398731 20.900206

H 29.963463 35.500572 22.215422

H 32.076122 35.828156 21.051773

H 31.225876 36.432957 19.633881

H 29.834385 33.231949 19.020277

H 29.685751 34.969109 18.700518

O 48.493343 24.235672 6.935932

C 48.074333 24.390661 8.280973

C 0.008801 25.523018 8.766479

O 1.289792 25.355316 8.161186

C 0.984701 24.407774 7.157906

H 46.992825 24.577923 8.308353

H 48.264526 23.460232 8.846663

H 0.127610 25.605160 9.853366

H 48.475742 26.474962 8.416407

H 1.435493 23.437740 7.440506

H 1.457983 24.727043 6.219164

O 14.859010 10.377985 31.422424

C 14.517738 11.757252 31.478161

C 13.890934 11.997804 32.825645

O 13.782880 10.724147 33.442677

C 14.872211 10.106141 32.803158

H 15.429590 12.359121 31.321705

H 13.857197 11.967979 30.624983

H 12.889608 12.451041 32.801689

H 14.509916 12.658834 33.458321

H 14.802295 9.020232 32.955704

H 15.832955 10.439590 33.252480

O 40.424076 31.147646 39.598766

C 39.895546 32.380726 39.140640

C 38.780300 32.025761 38.194077

O 38.760746 30.605915 38.114384

C 40.103832 30.386387 38.461521

H 39.603657 32.982594 40.012398

H 40.700809 32.947392 38.642426

H 38.945557 32.433037 37.181507

H 37.781422 32.366055 38.500145

H 40.780994 30.630850 37.613232

H 40.244640 29.319544 38.681538

O 8.634468 20.145788 8.942492

C 9.550230 19.103395 8.644247

C 10.683566 19.757580 7.900098

O 10.414655 21.154448 7.899121

C 9.570276 21.191605 9.022677

H 9.881724 18.631578 9.588367

H 9.014365 18.321598 8.086380

H 10.794064 19.431953 6.856237

H 11.661910 19.583033 8.377781

H 9.036367 22.151371 9.039790

H 10.154057 21.133425 9.967775

O 43.679848 39.168510 38.006817

C 42.857960 38.017086 38.073803

C 43.070156 37.468018 39.452576

O 43.683758 38.514324 40.204548

C 43.474010 39.593876 39.325943

H 41.807255 38.312397 37.895344

H 43.133717 37.337967 37.255825

H 43.749767 36.602615 39.478489

H 42.143639 37.152660 39.960087

H 44.182957 40.398651 39.570900

H 42.453617 40.015335 39.453556

O 24.434666 14.372038 15.246243

C 24.168200 14.806695 16.571726

C 24.161356 13.567752 17.426861

O 24.392618 12.472553 16.549725

C 23.912001 13.072957 15.370917

H 24.921638 15.556710 16.851881

H 23.201590 15.337183 16.581993

H 23.202566 13.409340 17.948547

H 24.930927 13.552595 18.210123

H 22.799200 13.090069 15.358695

H 24.230783 12.475487 14.506004

O 27.150661 9.328746 18.440897

C 26.264725 10.376518 18.075178

C 25.631561 10.848334 19.356171

O 26.314592 10.185837 20.415678

C 27.479221 9.817185 19.716999

H 26.835302 11.181783 17.575005

H 25.560667 9.989777 17.324186

H 24.560320 10.616583 19.444668

H 25.718590 11.937666 19.501383

H 27.998951 9.032456 20.284155

H 28.178877 10.676721 19.636326

O 0.482574 17.892319 13.721764

C 0.134457 19.242739 13.443564

C 47.564873 19.366926 13.780436

O 47.181549 18.104515 14.307011

C 48.447388 17.693815 14.759758

H 0.758326 19.912571 14.064991

H 0.401897 19.462757 12.400681

H 46.904816 19.601124 12.932145

H 47.372234 20.143343 14.541697

H 48.409737 16.627462 15.021335

H 48.750519 18.235548 15.683832

O 41.193161 47.142437 37.203506

C 41.444962 48.020550 36.121510

C 40.834778 0.430748 36.523899

O 39.894569 0.142769 37.556026

C 39.904346 47.625984 37.487087

H 42.522556 48.066509 35.923985

H 40.983902 47.617184 35.205257

H 40.322872 0.947541 35.699078

H 41.576481 1.132356 36.930199

H 39.183666 47.271515 36.718983

H 39.559650 47.218708 38.445873

O 47.580029 39.532764 10.797486

C 46.870106 39.635925 12.016872

C 47.593716 40.689568 12.815781

O 48.617046 41.196583 11.968469

C 48.020550 40.863136 10.739792

H 45.820377 39.907768 11.801744

H 46.835880 38.636070 12.468153

H 48.070911 40.327759 13.734965

H 46.936111 41.521233 13.122338

H 48.752476 40.967278 9.929639

H 47.182041 41.555458 10.499729

O 5.811398 44.318878 14.481070

C 6.251922 43.620201 13.322310

C 7.753909 43.659805 13.342356

O 8.119627 44.199581 14.603302

C 6.990203 45.013645 14.804250

H 5.829489 42.605186 13.365825

H 5.815799 44.098862 12.429528

H 8.169009 44.299324 12.543937

H 8.243325 42.680969 13.228925

H 7.068921 45.942120 14.204824

H 6.945222 45.314335 15.861313

O 24.984219 14.198469 34.997955

C 25.466305 13.721764 36.248142

C 26.903263 13.324754 36.024216

O 27.143326 13.479256 34.633705

C 26.217787 14.512362 34.400974

H 25.351404 14.516273 37.005005

H 24.805763 12.905744 36.573772

H 27.154572 12.290671 36.296059

H 27.609274 13.963294 36.585995

H 26.068663 14.629215 33.319466

H 26.605507 15.484350 34.775982

O 11.475140 38.815506 27.753510

C 10.254776 38.269859 28.253683

C 10.147700 38.724564 29.672062

O 10.828777 39.967419 29.698465

C 11.896596 39.572365 28.867777

H 9.435822 38.662468 27.629322

H 10.263577 37.179062 28.119715

H 10.636628 38.015617 30.367807

H 9.121930 38.869286 30.035337

H 12.627055 38.979782 29.453999

H 12.420238 40.465637 28.503523

O 5.524886 2.795690 45.656094

C 6.505187 2.159106 46.455982

C 6.174182 2.547804 47.861652

O 4.808607 2.960948 47.851383

C 4.450223 2.593763 46.540077

H 6.435270 1.063907 46.316635

H 7.499667 2.463708 46.104443

H 6.778009 3.390715 48.230301

H 6.295926 1.728360 48.586731

H 3.597532 3.206390 46.216408

H 4.115796 1.533767 46.515144

O 6.343841 8.012063 32.346500

C 5.711168 9.044679 31.602839

C 4.518184 9.463202 32.419350

O 4.636504 8.780169 33.661224

C 6.019192 8.526416 33.615753

H 6.420114 9.882702 31.466915

H 5.489194 8.669671 30.593204

H 3.543750 9.204070 31.978825

H 4.490314 10.551066 32.608074

H 6.279302 7.790578 34.389729

H 6.607373 9.445110 33.844574

O 6.060751 27.198574 44.149223

C 4.659973 27.115946 43.940937

C 4.326524 28.152473 42.905876

O 5.558133 28.736744 42.509842

C 6.395178 27.664036 42.865295

H 4.407686 26.107777 43.571796

H 4.158333 27.227911 44.911457

H 3.662071 28.958715 43.239815

H 3.844930 27.701683 42.020916

H 7.441973 27.997972 42.856495

H 6.307661 26.845079 42.124081

O 3.002507 6.972113 13.344312

C 3.510992 5.954165 12.496022

C 2.347343 5.483816 11.663378

O 1.238455 6.289569 12.039363

C 1.951800 7.405304 12.516558

H 4.328479 6.373666 11.883883

H 3.974007 5.186059 13.129672

H 2.065232 4.431155 11.810544

H 2.524824 5.603115 10.580401

H 1.276102 8.048244 13.098870

H 2.337076 8.020374 11.673644

O 38.749012 24.445421 45.075249

C 38.279152 24.407286 46.417358

C 38.214611 22.954191 46.804096

O 38.692783 22.220798 45.686897

C 38.358845 23.149761 44.686062

H 38.953384 25.020889 47.033405

H 37.291515 24.896702 46.467228

H 37.184929 22.626610 47.031448

H 38.824306 22.678925 47.674881

H 37.271473 23.108692 44.467022

H 38.882000 22.882318 43.756611

O 9.293055 16.171782 31.561768

C 10.688944 16.163960 31.851213

C 11.070308 14.736778 32.111813

O 9.849943 14.047390 32.324009

C 9.063747 14.789095 31.423889

H 11.232142 16.612795 31.005857

H 10.853224 16.830858 32.710751

H 11.718627 14.584722 32.986992

H 11.594439 14.282565 31.250811

H 7.999840 14.583255 31.611639

H 9.273008 14.468358 30.384430

O 15.422745 37.535004 31.065020

C 14.801806 36.665199 31.996914

C 13.549662 37.370720 32.453575

O 13.592688 38.664425 31.863438

C 14.983199 38.722607 31.671288

H 14.651706 35.694675 31.508965

H 15.501950 36.481850 32.832493

H 13.503213 37.484154 33.545841

H 12.598697 36.890106 32.179771

H 15.510752 38.904491 32.634476

H 15.229129 39.565033 31.018568

O 8.018909 25.656986 6.366820

C 8.722475 26.749741 5.799175

C 8.932714 26.394777 4.353415

O 8.565530 25.026758 4.212604

C 8.595354 24.650282 5.569868

H 8.146029 27.668436 5.980567

H 9.684686 26.874418 6.327706

H 9.980487 26.522877 4.035123

H 8.323510 26.977579 3.647403

H 9.633838 24.430265 5.897449

H 8.021841 23.721319 5.690144

O 15.722946 38.464943 28.042465

C 15.013512 38.818439 26.863661

C 15.513197 40.184502 26.492561

O 16.591770 40.471992 27.375566

C 16.911530 39.154819 27.743729

H 15.217884 38.072334 26.072084

H 13.936403 38.748520 27.064119

H 14.774426 40.993191 26.590349

H 15.876470 40.214329 25.453592

H 17.556425 39.174870 28.633091

H 17.486021 38.636555 26.946777

O 29.526361 1.568481 21.996382

C 28.305996 1.370954 22.687237

C 27.987217 2.714039 23.266617

O 29.220291 3.432275 23.347778

C 30.114054 2.408459 22.960548

H 27.554026 0.976389 21.990025

H 28.438007 0.597959 23.469521

H 27.527624 2.670036 24.264519

H 27.305653 3.296352 22.627588

H 30.435278 1.819301 23.846975

H 31.020037 2.863162 22.537624

O 10.594092 18.286886 19.249096

C 11.091331 18.338711 17.915300

C 10.326648 19.424131 17.212221

O 9.582500 20.087606 18.222347

C 9.381551 18.978228 19.059879

H 12.174796 18.517660 17.970549

H 10.960788 17.350100 17.443974

H 9.628948 19.019299 16.455360

H 10.946609 20.168280 16.690535

H 8.604644 18.308397 18.633535

H 9.010454 19.324390 20.034313

O 35.615959 27.298317 8.532771

C 35.388119 27.955437 9.781982

C 36.725338 28.150518 10.406833

O 37.571182 28.331423 9.286210

C 37.028961 27.282671 8.515659

H 34.928528 28.932316 9.567832

H 34.668419 27.370190 10.369674

H 37.051941 27.271914 10.996480

H 36.798187 29.024723 11.069818

H 37.398102 26.305305 8.891155

H 37.379032 27.372147 7.478154

O 20.487061 13.924181 26.493052

C 20.155079 13.508101 27.814137

C 20.082718 12.003671 27.776978

O 20.411766 11.625731 26.448561

C 19.979553 12.800624 25.811487

H 20.907539 13.921736 28.501081

H 19.192379 13.962806 28.111404

H 19.073568 11.624752 28.018507

H 20.769173 11.487364 28.460987

H 18.869686 12.834359 25.758680

H 20.344294 12.808447 24.774960

O 3.689939 30.363407 37.738888

C 3.615623 29.251095 38.609177

C 4.288877 29.679884 39.885277

O 4.668774 31.040081 39.721489

C 3.731498 31.367176 38.722122

H 2.552204 28.991964 38.752922

H 4.083527 28.390581 38.108513

H 3.619534 29.612900 40.756546

H 5.188504 29.099039 40.132187

H 4.015077 32.315205 38.243946

H 2.729196 31.504564 39.178288

O 43.680828 18.482944 9.754602

C 44.160954 17.920189 10.966655

C 43.563972 18.740120 12.080433

O 42.847202 19.801584 11.462428

C 43.496990 19.801584 10.212729

H 43.887157 16.855791 10.979366

H 45.265930 17.955393 10.966655

H 44.328655 19.172823 12.747820

H 42.864315 18.190565 12.729239

H 44.469955 20.334026 10.281667

H 42.879475 20.348694 9.486181

O 33.395248 35.634052 41.906509

C 34.553520 36.459850 41.859570

C 34.533962 37.309608 43.100960

O 33.269108 37.074432 43.698429

C 33.131229 35.737701 43.282349

H 35.449722 35.823753 41.800411

H 34.515385 37.025539 40.918873

H 34.647392 38.390137 42.932766

H 35.334335 37.028473 43.811371

H 32.102032 35.404743 43.477436

H 33.804482 35.067871 43.862217

O 16.202585 3.037221 38.449299

C 15.487284 3.451832 37.296894

C 14.905458 4.799806 37.614700

O 15.275577 5.090718 38.953873

C 15.466748 3.770124 39.403194

H 16.180584 3.446942 36.445671

H 14.707443 2.702794 37.073944

H 13.808305 4.803718 37.527180

H 15.254065 5.621694 36.975182

H 14.492804 3.277773 39.614899

H 16.015814 3.794570 40.354160

O 8.064867 21.030260 3.941249

C 8.364091 21.783699 2.769777

C 9.859233 21.767075 2.618698

O 10.314425 20.762815 3.508547

C 9.349281 20.961811 4.510850

H 7.825782 21.318727 1.929799

H 7.946058 22.796268 2.886142

H 10.316870 22.734663 2.897387

H 10.227396 21.531412 1.610040

H 9.572232 21.881971 5.092674

H 9.377150 20.120365 5.216372

O 25.948387 38.312397 29.057968

C 24.868343 38.759277 29.863234

C 24.609701 37.662613 30.859667

O 25.494663 36.600170 30.527685

C 25.708323 36.931664 29.177265

H 25.138233 39.732246 30.299358

H 23.994631 38.944092 29.213936

H 23.571218 37.291515 30.815176

H 24.791582 37.941299 31.908419

H 24.840963 36.629509 28.553394

H 26.579103 36.374775 28.803724

O 34.564766 33.299908 8.425696

C 35.504486 32.790447 7.496244

C 35.353405 33.645088 6.279791

O 34.763268 34.871323 6.716404

C 34.739799 34.660103 8.113271

H 36.521942 32.873562 7.914278

H 35.312824 31.719694 7.344187

H 34.682598 33.200169 5.529776

H 36.306816 33.862663 5.773262

H 33.913513 35.242420 8.547440

H 35.676098 35.043427 8.568953

O 3.585309 34.367729 1.464828

C 3.001040 34.339371 2.760488

C 3.436675 35.605202 3.449876

O 4.285943 36.278458 2.531180

C 3.736388 35.758724 1.346508

H 1.903397 34.274342 2.654879

H 3.316888 33.407963 3.253327

H 3.992586 35.458523 4.387151

H 2.583984 36.263298 3.700207

H 4.413553 35.974342 0.507507

H 2.767821 36.248142 1.109867

O 21.259079 42.856007 43.634377

C 20.535465 41.830235 44.288074

C 21.372021 40.596668 44.146778

O 22.369434 40.884159 43.168919

C 21.804722 42.052696 42.618385

H 20.312025 42.145592 45.316780

H 19.560053 41.695782 43.788879

H 20.771618 39.739578 43.811371

H 21.884417 40.273487 45.062538

H 21.037104 41.805790 41.857128

H 22.585539 42.609585 42.088879

O 27.097368 6.930065 8.541573

C 26.160091 5.870559 8.684339

C 25.363630 6.167827 9.928172

O 25.835932 7.415571 10.416121

C 27.134525 7.361300 9.879767

H 25.566534 5.819221 7.759776

H 26.708670 4.915193 8.747900

H 25.506395 5.400699 10.710457

H 24.277231 6.251433 9.777094

H 27.780399 6.694402 10.488483

H 27.579451 8.365069 9.921327

O 34.037212 32.574337 36.341038

C 34.472359 33.091625 37.588295

C 33.934536 34.495335 37.673859

O 33.315552 34.761803 36.421715

C 33.988316 33.795681 35.649208

H 34.126194 32.410061 38.375957

H 35.576355 33.066200 37.623009

H 34.728558 35.241928 37.850849

H 33.185009 34.654728 38.462986

H 35.013111 34.136951 35.393497

H 33.451477 33.655846 34.701664

O 8.664293 15.545466 46.326904

C 8.738610 15.769883 47.728172

C 7.524602 15.106896 48.327599

O 6.781920 14.567610 47.240711

C 7.856584 14.395019 46.348907

H 9.684197 15.346961 48.113449

H 8.802171 16.855791 47.890987

H 6.861616 15.776729 0.000979

H 6.861616 15.776729 48.893780

H 7.795957 14.287943 0.125656

H 7.463974 14.218514 45.338291

H 8.453566 13.499302 46.626129

O 46.887218 30.690989 32.557224

C 46.713646 32.054119 32.197865

C 47.204041 32.859386 33.369827

O 47.816666 31.935799 34.262608

C 48.025440 30.873358 33.365913

H 47.299385 32.267292 31.284060

H 45.659519 32.212044 31.928953

H 46.414425 33.386448 33.923782

H 47.940369 33.628468 33.082821

H 48.208302 29.956617 33.943825

H 0.036667 31.054752 32.744976

O 34.406841 42.889740 46.778183

C 34.335457 41.611195 47.390324

C 33.887600 41.852238 48.805283

O 33.851418 43.259861 0.083607

C 34.737354 43.610909 47.941833

H 33.675892 40.967766 46.796276

H 35.331406 41.137424 47.346809

H 34.583344 41.417091 0.649785

H 32.889210 41.463047 0.156946

H 35.787083 43.415829 48.247906

H 34.657173 44.688995 47.741863

O 34.921684 47.410370 42.386635

C 33.926712 47.189865 43.370846

C 32.846668 48.196079 43.079933

O 33.125847 48.738789 41.792099

C 34.044544 47.768753 41.349129

H 33.559528 46.148445 43.293594

H 34.392174 47.273472 44.361416

H 32.812935 0.142769 43.788391

H 31.836056 47.754574 43.077000

H 34.623924 48.184341 40.515018

H 33.509171 46.874016 40.964344

O 26.181604 41.412201 7.586206

C 25.575335 40.912029 6.404957

C 25.355316 42.102566 5.513641

O 25.897539 43.224655 6.193251

C 26.809879 42.531357 7.005849

H 26.244186 40.163967 5.946342

H 24.661039 40.370293 6.683157

H 24.302654 42.323563 5.301446

H 25.856958 41.993046 4.536763

H 27.165329 43.196789 7.802802

H 27.701683 42.228710 6.414735

O 37.452374 32.400280 10.044537

C 38.451252 31.548567 10.587247

C 39.769890 32.229156 10.344250

O 39.478981 33.463699 9.701309

C 38.152027 33.617222 10.141345

H 38.248348 31.391623 11.661422

H 38.355911 30.563866 10.109564

H 40.464172 31.666397 9.703265

H 40.312599 32.432060 11.281525

H 37.655766 34.373104 9.517962

H 38.121227 33.996632 11.181294

O 19.806473 15.086363 32.129902

C 20.236729 14.763669 33.454407

C 21.720137 14.903993 33.465652

O 21.973402 15.906294 32.495621

C 21.028793 15.477505 31.542700

H 19.773226 15.489239 34.138908

H 19.857321 13.768212 33.723808

H 22.237423 13.967207 33.190388

H 22.138660 15.218373 34.432266

H 21.440960 14.636059 30.952564

H 20.839579 16.292547 30.830332

O 9.349770 39.264339 38.859020

C 8.029176 38.912804 38.458099

C 7.373034 38.268883 39.647171

O 8.242837 38.506992 40.741394

C 9.447556 38.469833 40.017288

H 7.535358 39.828075 38.106556

H 8.084425 38.245903 37.584385

H 7.251780 37.179550 39.509293

H 6.376110 38.655628 39.902393

H 9.715487 37.425972 39.758160

H 10.263088 38.856575 40.641651

O 4.637482 14.244918 13.754033

C 5.882293 14.129042 13.075401

C 6.075419 12.663723 12.788401

O 4.924483 12.002693 13.298842

C 4.031211 13.087136 13.235281

H 5.852468 14.741179 12.155728

H 6.660666 14.573478 13.711496

H 6.962823 12.216355 13.259237

H 6.167337 12.451529 11.708848

H 3.135984 12.850984 13.827861

H 3.691895 13.259237 12.192397

O 31.962202 39.856922 38.893246

C 32.854496 38.996895 39.581165

C 32.112301 38.524593 40.804955

O 30.869448 39.213493 40.828423

C 31.234676 40.298424 40.011909

H 33.168388 38.202389 38.888355

H 33.772701 39.556232 39.839809

H 32.655987 38.754875 41.734406

H 31.897173 37.446995 40.826466

H 31.813078 41.039150 40.599113

H 30.327227 40.807888 39.668198

O 24.683529 45.476170 3.455254

C 23.808350 45.591068 2.345876

C 24.621435 45.212639 1.136758

O 25.888248 44.783360 1.619818

C 25.504930 44.460178 2.934057

H 22.947836 44.913902 2.496955

H 23.396183 46.609997 2.336098

H 24.796473 46.026215 0.419989

H 24.158421 44.394661 0.560800

H 26.405045 44.362881 3.559396

H 24.982264 43.480854 2.962904

O 14.637527 36.533192 2.888098

C 14.846298 35.128986 3.014241

C 14.050813 34.687485 4.212114

O 13.248971 35.800774 4.574410

C 14.156421 36.800632 4.183268

H 14.542674 34.657661 2.067188

H 15.926829 34.933907 3.122294

H 14.702554 34.424442 5.064805

H 13.397116 33.818172 4.050768

H 14.995911 36.876904 4.908837

H 13.646470 37.773109 4.184246

O 4.738201 35.368561 27.757420

C 4.336791 34.296833 28.599356

C 5.602626 33.744343 29.195847

O 6.673378 34.465023 28.598375

C 6.005991 34.860565 27.424950

H 3.607311 34.679661 29.323946

H 3.794570 33.540459 28.001394

H 5.729258 32.670170 28.980719

H 5.685744 33.848972 30.287622

H 5.915051 34.017166 26.705246

H 6.594172 35.639427 26.920376

O 47.192307 14.122196 41.189739

C 48.575485 13.887511 41.435181

C 0.390164 15.196860 41.226410

O 48.258659 16.170315 41.113464

C 47.307205 15.340606 40.494972

H 48.672783 13.489524 42.455574

H 0.032756 13.095448 40.753616

H 0.998878 15.198326 40.302334

H 1.068797 15.491684 42.039497

H 47.572693 15.162634 39.430088

H 46.332771 15.846645 40.501328

O 46.864727 5.845623 1.558213

C 47.454372 4.594945 1.868683

C 46.738094 3.608778 0.998391

O 46.188049 4.353904 48.803814

C 46.744450 5.621205 0.175036

H 48.540283 4.634059 1.655999

H 47.363434 4.414531 2.947747

H 45.903004 3.111049 1.514699

H 47.379566 2.809869 0.598448

H 46.096619 6.391756 48.625843

H 47.735504 5.713613 48.580376

O 19.675930 2.929657 34.780869

C 19.664194 4.319679 34.492405

C 20.848377 4.892213 35.225796

O 21.593016 3.779902 35.710323

C 21.077686 2.799602 34.845898

H 19.742424 4.465379 33.398182

H 18.685360 4.728912 34.775494

H 20.593647 5.525375 36.087776

H 21.498165 5.504840 34.577477

H 21.340240 1.803655 35.231171

H 21.536301 2.881253 33.834793

O 0.794019 7.720173 46.104931

C 48.521214 8.222791 46.737118

C 47.485664 8.316176 45.655609

O 48.181412 8.150430 44.422531

C 0.607251 8.354313 44.866966

H 48.272350 7.565183 47.580029

H 48.740253 9.214337 47.174217

H 46.961533 9.285720 45.645828

H 46.705334 7.542692 45.706455

H 0.835576 9.440222 44.941280

H 1.304460 7.929924 44.131130

O 37.789734 19.227583 38.854618

C 37.921257 18.322088 37.773109

C 36.603104 18.344578 37.058788

O 35.723034 19.142998 37.841560

C 36.411446 19.054014 39.068768

H 38.782745 18.621801 37.163418

H 38.155453 17.316362 38.163765

H 36.178715 17.332497 36.957581

H 36.643684 18.771412 36.045727

H 36.208542 18.079580 39.566498

H 36.047195 19.841187 39.744469

O 20.122320 11.750406 45.206772

C 20.594625 12.870541 44.480713

C 22.064342 12.634389 44.371193

O 22.434462 11.846236 45.505505

C 21.180361 11.703469 46.133781

H 20.052893 12.934590 43.526325

H 20.369717 13.801949 45.034180

H 22.674524 13.547707 44.348705

H 22.323963 12.055498 43.473034

H 21.051283 12.496022 46.897976

H 21.155914 10.740770 46.662800

O 4.624281 14.604280 23.547260

C 4.965552 15.982079 23.494946

C 3.665982 16.725739 23.320887

O 2.639722 15.742014 23.321865

C 3.326666 14.765625 24.066502

H 5.484305 16.257833 24.430754

H 5.699923 16.125824 22.689194

H 3.584331 17.302673 22.388992

H 3.477745 17.441528 24.142286

H 2.785912 13.811727 24.000008

H 3.360402 15.036002 25.145567

O 28.232658 13.472411 22.917522

C 27.909966 12.643678 21.802277

C 28.332888 13.390760 20.581423

O 28.273239 14.753403 20.961811

C 28.818396 14.572010 22.247202

H 28.398893 11.666800 21.926954

H 26.822102 12.467664 21.818901

H 27.687504 13.224524 19.709665

H 29.363550 13.132117 20.268511

H 28.657047 15.481905 22.841738

H 29.917503 14.431688 22.177773

O 14.135885 4.786605 45.416523

C 14.282565 4.828164 44.003033

C 15.159213 3.659137 43.635845

O 15.535198 3.049933 44.862564

C 14.394040 3.417607 45.595959

H 13.283195 4.769493 43.536102

H 14.683485 5.814820 43.734612

H 16.073019 3.906046 43.077980

H 14.620902 2.920856 43.015884

H 14.562721 3.217146 46.663776

H 13.515925 2.811336 45.285488

O 44.347237 36.578171 15.285846

C 44.990665 37.321831 14.265941

C 45.835533 38.344666 14.974886

O 45.491325 38.292843 16.356108

C 44.265099 37.610298 16.237787

H 45.552444 36.627552 13.625445

H 44.226959 37.781910 13.613711

H 45.669296 39.369461 14.606235

H 46.915089 38.154964 14.888347

H 43.452496 38.315331 15.971810

H 44.005474 37.181995 17.213198

O 31.313883 9.635304 46.692135

C 32.380726 10.311980 46.045284

C 32.736176 9.474447 44.848389

O 31.732895 8.469211 44.729088

C 30.743305 9.059835 45.541199

H 32.049229 11.325528 45.751438

H 33.195278 10.450836 46.770851

H 33.705719 8.966451 44.931995

H 32.775780 10.062627 43.916981

H 30.020666 8.288307 45.842865

H 30.180548 9.825986 44.969639

O 7.707461 36.285793 22.744930

C 7.969038 36.693558 24.080681

C 8.437430 38.123184 23.997562

O 8.346001 38.492809 22.628077

C 7.377434 37.552116 22.229113

H 7.041052 36.593327 24.672775

H 8.689717 35.988522 24.520716

H 9.472980 38.292843 24.324656

H 7.816003 38.810127 24.597479

H 7.352988 37.500778 21.130489

H 6.361442 37.863071 22.557671

O 46.648621 25.553331 33.649490

C 45.926472 26.169870 32.585094

C 46.548878 25.691210 31.304592

O 47.782444 25.101074 31.677645

C 47.369301 24.587210 32.920990

H 44.861588 25.903894 32.673592

H 45.987099 27.259691 32.729816

H 46.745914 26.476919 30.560934

H 45.927940 24.930927 30.795618

H 48.253281 24.288965 33.501835

H 46.759605 23.671448 32.777245

O 26.943844 34.072903 46.304413

C 26.137112 35.034626 46.965446

C 26.914019 36.318062 46.921444

O 27.999928 36.099022 46.028172

C 27.540335 34.942219 45.376919

H 25.172459 35.124588 46.434956

H 25.904385 34.661572 47.972637

H 27.331076 36.635864 47.888050

H 26.320461 37.168308 46.556702

H 28.394981 34.435200 44.906082

H 26.830412 35.206238 44.564808

O 21.396955 41.589680 19.681307

C 20.709034 41.449848 18.447742

C 20.691921 39.977200 18.145584

O 21.443892 39.360661 19.177711

C 21.174982 40.299404 20.188326

H 19.693041 41.872284 18.545528

H 21.231211 42.074207 17.709461

H 21.138802 39.694111 17.185329

H 19.669085 39.559650 18.142651

H 21.849215 40.125832 21.038084

H 20.136499 40.190369 20.572622

O 43.198257 15.212996 25.890694

C 43.993252 14.135885 25.416922

C 43.908669 14.187224 23.914934

O 43.132740 15.334738 23.594198

C 43.359112 16.061285 24.778870

H 45.028801 14.260563 25.782639

H 43.618244 13.207900 25.870646

H 43.437340 13.311554 23.445564

H 44.900215 14.282565 23.444586

H 42.634033 16.884640 24.840475

H 44.370216 16.526257 24.775450

O 17.404860 8.119139 42.559223

C 17.597008 6.715915 42.575359

C 17.778889 6.372199 44.021122

O 17.183374 7.428772 44.770157

C 16.616219 8.185143 43.725807

H 16.717426 6.213786 42.127502

H 18.454586 6.467540 41.935844

H 18.838884 6.329173 44.311054

H 17.326632 5.417322 44.325722

H 16.533100 9.228027 44.057301

H 15.587513 7.830182 43.508724

O 26.251522 2.078434 4.271764

C 25.529375 1.411045 5.300957

C 26.516033 1.182231 6.379533

O 27.224487 2.408950 6.416202

C 27.332541 2.605008 5.018846

H 24.737310 2.088209 5.658853

H 25.053648 0.512399 4.889280

H 27.197107 0.347138 6.128712

H 26.081375 0.982256 7.368634

H 28.259548 2.135151 4.650683

H 27.415173 3.682116 4.823275

O 33.813770 42.171017 40.677830

C 34.849319 42.094254 41.642487

C 35.305492 43.508236 41.895752

O 34.561829 44.330124 41.008347

C 33.430939 43.505302 40.886604

H 34.469421 41.613640 42.558739

H 35.603737 41.403889 41.244499

H 36.371841 43.703316 41.717293

H 35.108452 43.825062 42.936680

H 32.847649 43.827503 40.017780

H 32.777245 43.596733 41.780853

O 11.545056 41.369663 24.069435

C 10.366251 42.157814 24.110018

C 10.338871 42.770931 25.484882

O 11.378332 42.142170 26.230009

C 11.541145 40.999054 25.425724

H 10.397054 42.868229 23.272972

H 9.485204 41.513901 23.926180

H 9.375684 42.609585 25.998747

H 10.524175 43.853909 25.504930

H 10.738814 40.255398 25.628628

H 12.496022 40.514038 25.673609

O 2.606475 13.117938 45.952385

C 3.544728 14.192113 45.927452

C 3.162386 15.076095 44.773582

O 1.853037 14.672239 44.405907

C 2.002649 13.303731 44.692909

H 3.498280 14.698643 46.903839

H 4.561209 13.775546 45.847755

H 3.836129 14.954841 43.905243

H 3.151141 16.152714 44.999954

H 2.599141 12.808447 43.898399

H 1.014037 12.822625 44.697796

O 31.994471 8.598288 13.319865

C 32.508823 7.731907 14.319723

C 31.794010 6.418647 14.145664

O 30.956964 6.563369 13.004018

C 31.663956 7.607719 12.378191

H 33.601578 7.631677 14.186245

H 32.361168 8.213013 15.297090

H 31.167204 6.133113 15.001289

H 32.486820 5.573779 13.984807

H 31.034216 8.047755 11.591994

H 32.581184 7.224400 11.879972

O 8.514193 41.792587 20.967188

C 7.967082 40.767792 20.140411

C 6.492475 41.031815 20.037737

O 6.302282 42.313786 20.607338

C 7.316807 42.215023 21.576393

H 8.501480 40.791260 19.182602

H 8.185143 39.785049 20.584356

H 5.900872 40.287666 20.604404

H 6.082264 41.035725 19.019299

H 7.028829 41.516830 22.392412

H 7.465931 43.197765 22.043808

O 33.991253 29.228117 2.776622

C 32.688747 29.277985 3.341823

C 31.925043 28.126560 2.745331

O 32.829559 27.450863 1.880906

C 34.033302 27.851784 2.489621

H 32.768932 29.196337 4.439466

H 32.269249 30.274910 3.142829

H 31.039595 28.405249 2.157150

H 31.562258 27.424950 3.515881

H 34.871323 27.651323 1.807567

H 34.220558 27.258713 3.409784

O 26.025637 6.261701 38.404316

C 25.981146 6.873838 39.695087

C 26.350286 5.811398 40.665119

O 27.284626 5.026180 39.944439

C 26.582037 5.003200 38.722607

H 26.731159 7.679103 39.715134

H 24.998890 7.341743 39.838829

H 25.482437 5.198283 40.970699

H 26.819166 6.179072 41.586262

H 25.777262 4.244873 38.749989

H 27.264582 4.697620 37.917347

O 39.011566 37.671413 17.200975

C 39.452087 37.388325 15.884781

C 40.737480 36.650532 16.076441

O 41.256233 37.059765 17.340321

C 40.272018 37.984325 17.743687

H 38.666382 36.824589 15.362118

H 39.594856 38.330978 15.322514

H 41.494343 36.841213 15.301980

H 40.589333 35.560711 16.122400

H 40.575157 39.008633 17.445929

H 40.206505 37.970638 18.839375

O 43.313152 4.891236 18.616423

C 43.262794 4.304522 19.907192

C 41.869350 3.756923 20.062672

O 41.201473 4.030233 18.838396

C 42.335789 4.086949 18.008684

H 44.033344 3.516370 19.976131

H 43.547840 5.070673 20.641073

H 41.282635 4.202336 20.878693

H 41.863968 2.667102 20.240641

H 42.055141 4.524540 17.040607

H 42.733284 3.069001 17.798935

O 7.668346 21.302593 37.172218

C 6.434782 20.698767 37.533047

C 6.525233 20.368252 38.998363

O 7.779822 20.863047 39.445244

C 8.459921 20.784819 38.210701

H 5.624628 21.390600 37.258755

H 6.296904 19.795227 36.921398

H 6.483185 19.277943 39.174870

H 5.735125 20.809265 39.621746

H 8.739588 19.739000 37.977482

H 9.393773 21.360287 38.267906

O 6.080798 43.330753 38.550018

C 5.308780 42.140701 38.475212

C 4.566587 42.035583 39.777718

O 4.946973 43.171364 40.537998

C 5.245708 44.013786 39.454044

H 4.615480 42.209641 37.621056

H 5.981545 41.303661 38.249325

H 4.784649 41.134487 40.367851

H 3.470900 42.049274 39.632015

H 5.755171 44.912437 39.825630

H 4.320168 44.362392 38.948982

O 23.154652 5.829978 48.584778

C 23.199144 6.378066 47.272491

C 21.915220 7.141771 47.084255

O 21.272278 7.137371 48.350090

C 21.766586 5.899894 48.801857

H 24.108551 6.991671 47.198177

H 23.322844 5.557156 46.546432

H 21.250278 6.666533 46.338638

H 22.037941 8.186610 46.762543

H 21.248810 5.057960 48.294842

H 21.564169 5.797219 0.985187

O 3.780880 15.657919 28.216034

C 4.315767 16.881706 27.732485

C 3.251860 17.926544 27.944679

O 2.176219 17.265514 28.595930

C 2.907655 16.210407 29.169933

H 5.236419 17.109056 28.298174

H 4.621347 16.735518 26.686668

H 2.862185 18.387604 27.024519

H 3.598999 18.760656 28.580774

H 2.207021 15.433501 29.502893

H 3.465522 16.554613 30.068583

O 40.184994 21.379354 34.739315

C 40.152233 20.693388 35.978256

C 40.979988 21.543146 36.873974

O 40.719879 22.891121 36.477451

C 40.033424 22.682837 35.257576

H 39.111794 20.613205 36.338596

H 40.522839 19.669573 35.838421

H 42.053185 21.340729 36.722404

H 40.769749 21.438026 37.947170

H 40.387409 23.409872 34.515385

H 38.962185 22.895519 35.417942

O 9.637749 1.154359 9.624548

C 10.715835 2.079900 9.540941

C 10.722680 2.608920 8.130383

O 9.694954 1.909753 7.443929

C 8.870621 1.647199 8.551839

H 10.562800 2.876852 10.289001

H 11.633553 1.552835 9.839676

H 11.664355 2.461752 7.581317

H 10.511952 3.692873 8.089314

H 8.118649 0.896205 8.271684

H 8.320087 2.561005 8.857908

O 2.735552 44.789715 5.882782

C 1.766497 44.677261 4.850655

C 0.822866 43.584019 5.266243

O 1.236988 43.180653 6.563369

C 2.598163 43.501389 6.427936

H 1.300548 45.659519 4.704954

H 2.281338 44.451866 3.907513

H 0.863447 42.715683 4.584189

H 48.659092 43.882267 5.315136

H 3.125228 42.746975 5.802598

H 3.069979 43.487701 7.418993

O 28.121183 37.711502 24.333946

C 28.647757 36.449093 24.709442

C 28.172522 36.192894 26.114624

O 27.405882 37.327694 26.490606

C 28.001884 38.261059 25.623251

H 28.316753 35.710323 23.966272

H 29.749313 36.488697 24.639526

H 29.007608 36.071152 26.826012

H 27.539846 35.303047 26.233431

H 28.994410 38.570061 26.016346

H 27.380947 39.167042 25.575825

O 29.757624 5.333715 19.025656

C 29.450089 6.487586 19.797184

C 30.255354 6.381488 21.063995

O 31.144714 5.293134 20.872826

C 30.306690 4.527962 20.039692

H 29.680374 7.370100 19.182112

H 28.362713 6.513988 19.979553

H 29.619745 6.174671 21.944555

H 30.845980 7.271826 21.318727

H 29.498983 4.050768 20.633739

H 30.886070 3.714386 19.581076

O 35.939140 8.474588 5.428567

C 37.152660 8.119139 4.793939

C 37.192753 6.626930 4.869234

O 36.403622 6.256811 6.001102

C 36.024704 7.532425 6.472428

H 37.158527 8.535215 3.777458

H 38.001438 8.580197 5.332249

H 38.204346 6.213786 4.985110

H 36.748318 6.148270 3.982319

H 36.744896 7.887386 7.234179

H 35.053692 7.448818 6.977491

O 29.668638 44.022591 27.394146

C 29.386040 44.117439 26.005590

C 30.436256 43.298977 25.308868

O 31.277702 42.772888 26.325348

C 30.330158 42.780708 27.366276

H 28.372490 43.725319 25.811487

H 29.357193 45.180859 25.733747

H 31.065994 43.844131 24.594545

H 29.982040 42.470730 24.738289

H 30.839132 42.623764 28.325554

H 29.608500 41.943665 27.249914

O 36.777164 18.053667 28.616957

C 37.829826 17.164795 28.277641

C 37.216709 16.142937 27.359434

O 35.917141 16.621597 27.028919

C 36.100975 17.971037 27.388279

H 38.635090 17.737818 27.780399

H 38.265461 16.760450 29.202204

H 37.099369 15.145522 27.807291

H 37.799511 16.001637 26.433403

H 35.121166 18.459476 27.478241

H 36.661289 18.509836 26.595240

O 37.256802 3.091003 16.271034

C 36.103912 2.757554 15.514663

C 35.761662 3.981830 14.709887

O 36.805523 4.918615 14.946529

C 37.799511 4.008721 15.355761

H 36.338104 1.899974 14.859989

H 35.327003 2.409437 16.208452

H 34.807762 4.460979 14.972931

H 35.706902 3.766701 13.628378

H 38.619934 4.558764 15.835889

H 38.234169 3.478723 14.484003

O 47.866539 35.465370 42.310360

C 48.783279 35.954296 43.280396

C 0.460081 34.747623 43.974674

O 48.811150 33.619175 43.310219

C 47.609852 34.207355 42.886318

H 0.628272 36.583549 42.762623

H 48.247414 36.621685 43.973694

H 0.178948 34.709000 45.042492

H 1.553324 34.659126 43.941425

H 46.904816 34.296345 43.737057

H 47.128746 33.557571 42.140701

O 47.106258 19.579121 44.801453

C 46.302460 20.245043 45.763660

C 45.776371 21.483986 45.091381

O 46.328861 21.504520 43.782036

C 47.459263 20.703655 44.031876

H 45.528488 19.545874 46.110798

H 46.921444 20.475817 46.646664

H 46.079998 22.404636 45.618938

H 44.681175 21.522121 44.998978

H 48.250347 21.288414 44.547207

H 47.879250 20.368740 43.072601

O 48.032288 23.106737 0.615073

C 48.843418 23.134607 48.347645

C 0.375983 21.715248 48.138870

O 48.337868 20.896782 48.850754

C 47.399124 21.880505 0.332958

H 48.249859 23.511568 47.493977

H 0.771527 23.848930 48.504101

H 1.379268 21.518209 48.542728

H 0.390654 21.408201 47.081810

H 46.854458 21.538746 1.223298

H 46.645195 22.011049 48.420006

O 34.779404 1.236989 37.612740

C 35.403767 0.236642 36.807480

C 36.546387 48.573040 37.604427

O 36.323929 0.112455 38.931381

C 35.779751 1.365087 38.601845

H 34.640060 48.383335 36.553722

H 35.723526 0.685965 35.855534

H 37.515934 0.073829 37.255337

H 36.640266 47.478821 37.593674

H 36.576214 2.061322 38.259102

H 35.339226 1.799746 39.506847

O 25.602224 42.123589 34.561829

C 24.497736 41.261612 34.803848

C 23.327732 42.145103 35.141701

O 23.852352 43.456409 35.263931

C 24.898657 43.321465 34.335457

H 24.324656 40.639694 33.909599

H 24.792562 40.573692 35.608624

H 22.814358 41.897217 36.079464

H 22.553270 42.134834 34.352570

H 25.589025 44.168289 34.449863

H 24.504093 43.353733 33.296974

O 4.739668 5.797219 34.365772

C 4.667306 4.412575 34.027920

C 3.778435 3.764745 35.043427

O 2.986861 4.822786 35.551910

C 4.000409 5.798197 35.569023

H 4.265408 4.349992 33.008018

H 5.683788 3.994542 33.985386

H 4.358793 3.299286 35.862869

H 3.111049 2.983439 34.654240

H 4.679041 5.635384 36.432472

H 3.540328 6.786809 35.703476

O 4.779271 42.564606 9.314568

C 3.831728 42.177372 10.301713

C 2.951658 41.146225 9.652416

O 3.361380 41.075821 8.295641

C 4.705443 41.430294 8.487301

H 4.368572 41.789165 11.183249

H 3.306620 43.081890 10.641028

H 1.879439 41.376999 9.674907

H 3.062156 40.146858 10.107120

H 5.160146 41.663509 7.513845

H 5.278955 40.583469 8.921469

O 16.934999 10.945142 16.082308

C 16.108221 12.053541 16.424070

C 16.572702 12.524379 17.771555

O 17.471842 11.528433 18.230658

C 17.993040 11.173471 16.975090

H 16.205030 12.819692 15.638851

H 15.060938 11.717159 16.398643

H 15.782106 12.660790 18.523037

H 17.106613 13.488056 17.711416

H 18.582685 10.251353 17.073366

H 18.679005 11.959179 16.587372

O 0.351539 0.375497 24.692329

C 48.730965 0.585247 23.384937

C 0.889849 0.063072 22.442284

O 1.933710 48.437607 23.256838

C 1.158270 48.155499 24.396528

H 47.771687 0.044981 23.281284

H 48.492855 1.653554 23.276884

H 1.324017 0.819443 21.774408

H 0.506040 48.150608 21.791033

H 1.821746 47.953571 25.248241

H 0.541243 47.242176 24.251318

O 11.140714 36.836811 20.210817

C 10.148679 35.872646 19.890568

C 10.024980 35.877045 18.391516

O 10.987190 36.808945 17.911388

C 11.868238 36.778141 19.010010

H 10.461593 34.888435 20.281221

H 9.229493 36.137157 20.431322

H 9.038323 36.184582 18.016018

H 10.228374 34.884525 17.949524

H 12.543937 37.643055 18.956228

H 12.507756 35.870689 18.979696

O 30.536976 32.763065 30.736948

C 29.445200 31.858549 30.765793

C 29.208069 31.438560 29.342524

O 30.051960 32.252625 28.540682

C 30.288601 33.293060 29.457422

H 29.676952 31.035194 31.455183

H 28.579308 32.380234 31.198496

H 28.163719 31.578390 29.018364

H 29.457422 30.387852 29.132774

H 29.427599 33.996143 29.479912

H 31.165737 33.868042 29.127398

O 33.344887 43.672028 12.753198

C 34.517338 44.099838 12.075543

C 35.076183 42.878983 11.395934

O 34.214691 41.797966 11.741606

C 33.050068 42.565582 11.938154

H 34.240116 44.895813 11.361709

H 35.193527 44.566765 12.805512

H 36.102444 42.614964 11.688313

H 35.096230 43.000240 10.298290

H 32.284893 41.949535 12.431483

H 32.619808 42.892185 10.965188

O 32.177818 36.229076 25.764551

C 32.236000 36.133736 27.181952

C 32.780174 37.451881 27.670391

O 33.018772 38.231724 26.507719

C 33.221191 37.161953 25.617382

H 31.234676 35.873627 27.552561

H 32.891655 35.287399 27.456240

H 33.728210 37.333565 28.226789

H 32.110832 38.020020 28.329954

H 34.212246 36.684265 25.783619

H 33.228035 37.542336 24.586723

O 2.408948 21.176451 37.042652

C 2.539492 21.805700 38.308487

C 1.140669 22.166529 38.737274

O 0.279667 21.786142 37.671902

C 1.232099 21.832590 36.636353

H 3.074379 21.120712 38.982227

H 3.187322 22.695059 38.202389

H 1.022837 23.249016 38.910358

H 0.794019 21.668310 39.654507

H 1.441849 22.883785 36.343483

H 0.828733 21.333885 35.744057

O 28.329954 5.871536 46.804588

C 27.287071 6.238232 45.908382

C 27.303207 5.221262 44.800472

O 28.481033 4.458535 44.997997

C 28.480545 4.530896 46.401711

H 26.328283 6.251922 46.453537

H 27.470909 7.272804 45.585690

H 27.316408 5.630984 43.781548

H 26.424603 4.548986 44.847897

H 29.428089 4.139265 46.791874

H 27.676748 3.891867 46.830013

O 14.348570 10.176059 18.635002

C 13.529614 9.952618 19.769314

C 13.843995 8.556240 20.219128

O 15.050181 8.169497 19.566898

C 15.456481 9.418709 19.058903

H 13.759901 10.708501 20.543776

H 12.481353 10.112497 19.480358

H 13.070024 7.829692 19.931639

H 13.980896 8.461388 21.307970

H 16.127289 9.251006 18.204744

H 16.038305 9.973642 19.823586

O 18.083002 41.777431 11.224809

C 17.589184 40.858734 10.268955

C 18.786570 40.537998 9.435822

O 19.924305 40.700321 10.285089

C 19.282831 41.094398 11.479052

H 17.179462 39.963997 10.779395

H 16.751650 41.321259 9.732111

H 18.896578 41.237652 8.592421

H 18.804659 39.524937 9.017299

H 19.955595 41.751026 12.043274

H 19.085793 40.202595 12.104879

O 8.384626 24.404352 47.368809

C 9.001654 23.889999 48.540283

C 8.979163 25.006222 0.659072

O 8.302975 26.092621 0.038625

C 7.553937 25.346027 48.001972

H 8.437430 23.004063 48.883022

H 10.002488 23.525261 48.266972

H 9.968264 25.359230 0.985677

H 8.436942 24.727043 1.579237

H 7.128081 26.023682 47.248043

H 6.702225 24.837053 48.500679

O 33.433384 20.736902 23.316977

C 32.742039 21.224852 24.454222

C 33.349289 20.517374 25.632050

O 34.192200 19.493069 25.114765

C 33.680294 19.442223 23.804926

H 32.829559 22.321030 24.471334

H 31.664442 21.000923 24.354969

H 32.595364 20.058271 26.292593

H 33.963871 21.172537 26.263744

H 32.754753 18.826662 23.772167

H 34.418087 18.951338 23.157097

O 11.228721 14.226828 39.312256

C 12.353744 13.721764 38.614555

C 13.394671 13.476811 39.671616

O 12.897432 14.054235 40.873894

C 11.944510 14.916215 40.305759

H 12.042786 12.833871 38.043976

H 12.681815 14.467868 37.865517

H 14.370083 13.932981 39.432529

H 13.589265 12.414859 39.871101

H 12.439306 15.824155 39.894077

H 11.244367 15.259932 41.079239

O 14.637527 46.587013 32.968414

C 15.427633 45.720634 32.168041

C 15.813398 46.510742 30.949142

O 15.176813 47.775600 31.071865

C 14.127574 47.375168 31.919176

H 16.267122 45.349537 32.773819

H 14.829185 44.830296 31.909395

H 15.467237 46.024261 30.022621

H 16.892952 46.676979 30.823486

H 13.346267 46.821701 31.353485

H 13.637669 48.263550 32.336231

O 6.084709 46.029148 21.482517

C 5.047692 45.298199 20.836643

C 5.360118 45.305046 19.365459

O 6.661644 45.855579 19.242739

C 6.600528 46.705334 20.362873

H 5.025202 44.292477 21.277657

H 4.074726 45.759750 21.077686

H 4.649216 45.925495 18.789015

H 5.358162 44.313499 18.890221

H 5.983990 47.593231 20.130144

H 7.608697 47.073990 20.600492

O 12.451041 21.850681 18.440897

C 12.472553 21.403801 19.786917

C 12.023717 22.572340 20.622982

O 11.666800 23.598598 19.709665

C 11.409624 22.782089 18.595398

H 11.791476 20.540354 19.893501

H 13.476811 21.019991 20.001066

H 12.780088 22.980593 21.308460

H 11.142669 22.326408 21.240988

H 11.331883 23.405962 17.693815

H 10.439102 22.259914 18.719097

O 40.958965 40.341938 44.352615

C 41.562302 41.326641 43.535614

C 42.187153 40.521862 42.464863

O 42.692703 39.343056 43.092159

C 42.065411 39.469200 44.357502

H 40.819130 42.037540 43.157185

H 42.289825 41.918732 44.119884

H 42.992416 41.027905 41.920685

H 41.410244 40.225574 41.740761

H 42.809067 39.837852 45.088940

H 41.743694 38.478142 44.699265

O 8.492190 9.250029 45.736279

C 9.284742 10.405855 45.958740

C 10.600448 9.912037 46.484341

O 10.500217 8.492680 46.544968

C 9.103350 8.401738 46.674046

H 9.345859 10.954921 45.009247

H 8.768435 11.074220 46.670132

H 10.823888 10.301713 47.492996

H 11.462428 10.176059 45.855091

H 8.784570 8.657449 47.706661

H 8.783591 7.365700 46.489719

O 38.288441 46.184139 15.500484

C 38.186745 44.769180 15.507329

C 37.445042 44.416664 16.771208

O 37.102791 45.652184 17.380411

C 38.195053 46.389000 16.888062

H 37.694881 44.458225 14.573965

H 39.203712 44.336967 15.476527

H 38.074287 43.831417 17.468418

H 36.523411 43.834351 16.631863

H 39.142109 46.108841 17.403391

H 38.034687 47.457798 17.086567

O 12.950725 0.273309 19.827986

C 12.759554 48.693806 18.503481

C 12.628033 47.202576 18.584154

O 12.671547 46.875481 19.962440

C 13.432808 47.973125 20.401009

H 11.877039 0.305581 18.086424

H 13.621534 0.101206 17.884008

H 13.450898 46.692135 18.057577

H 11.698580 46.803612 18.155363

H 14.506004 47.826447 20.167301

H 13.362891 48.040596 21.494741

O 35.828156 20.607338 1.150936

C 35.108940 21.566124 0.393098

C 35.748947 22.873030 0.730947

O 37.085678 22.574295 1.132846

C 37.105236 21.171560 0.974922

H 35.190102 21.330462 48.207321

H 34.043568 21.493275 0.651741

H 35.251221 23.377115 1.574348

H 35.775352 23.593710 48.793056

H 37.793644 20.739838 1.714670

H 37.502735 20.911451 48.865910

O 31.931889 17.160395 45.772949

C 33.131718 16.614752 45.265442

C 32.890675 16.416246 43.803551

O 31.873705 17.343742 43.435383

C 31.653687 17.990595 44.673351

H 33.976093 17.303650 45.455147

H 33.340488 15.703878 45.826244

H 32.523979 15.403677 43.569351

H 33.776123 16.594706 43.176743

H 30.600046 18.289818 44.720776

H 32.276093 18.906357 44.726643

O 15.047248 46.063374 9.638726

C 13.693894 46.443760 9.859722

C 13.715898 47.881207 10.302691

O 15.086851 48.227859 10.424434

C 15.587513 47.347298 9.449512

H 13.125761 46.297081 8.925381

H 13.261683 45.751926 10.594581

H 13.222569 48.084114 11.264901

H 13.235281 48.548107 9.564409

H 16.681734 47.296940 9.539474

H 15.360651 47.719372 8.427652

O 29.266253 39.636414 46.181206

C 29.296076 38.726517 45.082096

C 30.352161 37.725685 45.405277

O 30.347761 37.649410 46.825611

C 30.189348 39.032101 47.060299

H 28.298664 38.268883 44.995556

H 29.460358 39.291721 44.155579

H 31.343708 38.058643 45.047382

H 30.186415 36.723873 44.984306

H 31.168669 39.543518 46.974247

H 29.835854 39.184155 48.088024

O 44.986263 7.084566 18.273193

C 45.740192 8.046777 17.552515

C 46.776230 8.563085 18.514236

O 46.579681 7.850717 19.727266

C 46.002258 6.698313 19.163044

H 46.201740 7.557360 16.675379

H 45.047871 8.799237 17.151594

H 46.720005 9.632370 18.747454

H 47.801514 8.392939 18.141184

H 45.571999 6.078842 19.961464

H 46.769386 6.072975 18.652603

O 2.623099 24.546629 40.204548

C 2.876364 25.784594 39.566010

C 1.752807 26.691557 39.977684

O 0.997902 26.001190 40.967278

C 1.982114 25.063425 41.342285

H 3.857642 26.170847 39.898968

H 2.967304 25.609070 38.485477

H 1.068797 26.981979 39.167534

H 2.133682 27.626877 40.413319

H 1.503454 24.236160 41.879128

H 2.720884 25.520573 42.035095

O 47.420635 21.020481 18.260483

C 46.765472 19.841677 17.829737

C 45.430698 20.291489 17.299250

O 45.274731 21.651197 17.694792

C 46.266769 21.697647 18.693184

H 47.410370 19.330257 17.102701

H 46.665733 19.152777 18.689762

H 44.585342 19.707708 17.696749

H 45.344162 20.248463 16.203562

H 45.891270 21.257612 19.639259

H 46.518078 22.744930 18.906845

O 43.318043 23.766788 44.963284

C 43.621178 24.100239 46.313213

C 42.406681 23.747232 47.125324

O 41.481140 23.161497 46.223251

C 41.913349 23.807859 45.052273

H 44.529114 23.557039 46.609997

H 43.880310 25.170990 46.373844

H 41.943665 24.632681 47.595673

H 42.592472 23.028019 47.935478

H 41.544701 24.854656 45.022934

H 41.479183 23.297907 44.181000

O 3.761812 39.164597 12.211466

C 2.887120 39.473114 13.297375

C 3.714386 40.209927 14.282565

O 4.972398 39.561123 14.206780

C 5.027158 39.350883 12.815291

H 2.013406 40.014843 12.911121

H 2.531669 38.528015 13.740343

H 3.347690 40.151745 15.317136

H 3.819017 41.278233 14.019032

H 5.647607 38.465919 12.625587

H 5.525375 40.208950 12.330765

O 48.243992 18.495169 2.034918

C 0.725080 18.768967 1.792899

C 1.502966 17.797956 2.637278

O 0.549066 17.115412 3.443520

C 48.388226 18.044865 3.362358

H 0.907453 18.681450 0.713346

H 0.939719 19.821629 2.055453

H 2.240757 18.299109 3.285596

H 2.064744 17.044029 2.065232

H 48.561306 18.899023 4.053702

H 47.456818 17.556425 3.684072

O 39.347946 13.691451 37.877251

C 39.708778 14.668818 38.846794

C 40.632359 15.636406 38.154472

O 40.603516 15.281445 36.781075

C 40.404522 13.902668 36.972736

H 38.780788 15.114232 39.233536

H 40.187923 14.164244 39.703888

H 41.671333 15.555733 38.527527

H 40.361008 16.697380 38.246391

H 41.334461 13.418139 37.340897

H 40.157124 13.430851 36.011505

O 10.490439 28.848709 36.768364

C 10.679654 27.918278 37.819069

C 10.229352 28.676115 39.014011

O 10.697744 30.007467 38.813549

C 11.057107 29.951241 37.447487

H 10.117876 27.002026 37.602962

H 11.746984 27.633232 37.892406

H 10.598982 28.313332 39.978664

H 9.128775 28.703005 39.075127

H 12.160129 29.915060 37.365833

H 10.739304 30.880203 36.961491

O 45.546089 37.921745 36.367443

C 46.906773 37.872364 36.756142

C 47.537003 39.006187 36.023235

O 46.815834 39.137707 34.799450

C 45.752903 38.228790 35.007732

H 46.965935 37.940815 37.851337

H 47.343876 36.899887 36.461315

H 48.601398 38.872707 35.788551

H 47.439217 39.953239 36.578659

H 45.966076 37.298363 34.447422

H 44.828831 38.653183 34.590187

O 29.735622 41.971535 8.177321

C 29.176779 40.779530 8.712208

C 29.079971 39.807049 7.564205

O 29.559118 40.498886 6.419136

C 30.408388 41.406822 7.076744

H 29.823139 40.423100 9.530185

H 28.217503 41.036217 9.184024

H 28.065933 39.447201 7.344676

H 29.688684 38.899113 7.711372

H 30.691967 42.205730 6.376110

H 31.351040 40.913006 7.398458

O 42.598339 4.023388 26.425091

C 41.659599 4.116774 27.481667

C 42.390057 3.680650 28.715721

O 43.598198 3.070468 28.277641

C 43.277950 2.896899 26.919399

H 41.262589 5.140100 27.528112

H 40.806908 3.462588 27.241600

H 41.830235 2.964370 29.337637

H 42.659946 4.520139 29.369415

H 42.675591 1.972825 26.774675

H 44.206913 2.763910 26.353218

O 29.559118 37.455795 39.175842

C 30.196680 36.834858 38.075756

C 29.132774 36.777164 37.027004

O 28.181808 37.795113 37.340408

C 28.853106 38.409695 38.416050

H 31.070885 37.435749 37.759911

H 30.589779 35.857979 38.389160

H 28.608156 35.812996 37.042652

H 29.488226 36.932640 36.001232

H 28.120205 38.915733 39.059479

H 29.540541 39.193935 38.032242

O 13.963784 33.305775 39.640812

C 14.286476 34.287540 38.668339

C 14.014143 33.652916 37.335030

O 13.415206 32.391479 37.605408

C 12.953170 32.660389 38.907421

H 13.659182 35.182281 38.829681

H 15.324959 34.605347 38.831150

H 14.911815 33.476410 36.728760

H 13.329644 34.253319 36.711159

H 12.693060 31.712358 39.397327

H 12.026650 33.272530 38.894222

O 18.720564 2.251024 16.502298

C 19.051079 2.069632 17.867874

C 17.892809 2.646078 18.615932

O 16.786366 2.658790 17.711906

C 17.340809 1.998738 16.597639

H 19.196781 0.994968 18.078602

H 20.019156 2.551715 18.063444

H 18.068333 3.682117 18.941561

H 17.609720 2.081366 19.515072

H 16.846992 2.354188 15.682365

H 17.145727 0.911362 16.655821

O 46.617329 10.190237 38.455166

C 46.041862 9.461246 37.381966

C 47.148304 9.233894 36.391888

O 48.347157 9.708155 36.996204

C 47.934990 9.710110 38.341244

H 45.625294 8.512237 37.767242

H 45.190147 10.037692 36.995224

H 47.020206 9.766337 35.438965

H 47.270046 8.170964 36.128357

H 48.602863 10.363317 38.920135

H 48.026909 8.692651 38.773945

O 21.113867 11.671689 4.839898

C 20.431812 11.058084 3.755456

C 20.415678 9.580544 4.039034

O 21.116800 9.408442 5.260865

C 20.870380 10.680632 5.809442

H 19.412886 11.477585 3.686517

H 20.952032 11.345574 2.831382

H 20.902161 8.955694 3.275818

H 19.388439 9.187447 4.151977

H 21.540701 10.841490 6.665555

H 19.830919 10.753483 6.195695

O 4.271764 0.198994 31.820900

C 4.379328 1.615418 31.906950

C 2.984905 2.153728 31.758806

O 2.136126 1.037994 31.957802

C 2.963392 0.103653 31.312414

H 4.857011 1.848637 32.869164

H 5.071161 1.969891 31.129068

H 2.806447 2.578117 30.751616

H 2.696438 2.936013 32.469707

H 2.956059 0.255220 30.209883

H 2.580073 47.984371 31.489897

O 43.860264 10.807264 12.143015

C 43.950714 10.195615 10.864958

C 45.126587 10.825844 10.171658

O 45.643383 11.794410 11.073241

C 45.213127 11.173471 12.262315

H 44.079792 9.107262 10.998924

H 42.990948 10.341805 10.350606

H 44.883591 11.324550 9.224115

H 45.916206 10.091475 9.933062

H 45.324116 11.874105 13.101315

H 45.843845 10.288511 12.496511

O 9.713532 20.294912 32.969883

C 10.297801 21.583727 32.853027

C 11.217475 21.732849 34.036232

O 11.064441 20.552578 34.815586

C 9.777094 20.193214 34.371639

H 10.788685 21.653154 31.872240

H 9.496449 22.344498 32.846668

H 10.963721 22.606564 34.659618

H 12.280893 21.840414 33.777592

H 9.003119 20.831755 34.852253

H 9.563432 19.156687 34.669884

O 39.157757 16.732582 44.425953

C 38.512367 15.563557 43.945824

C 39.411507 14.992488 42.880451

O 40.567333 15.817799 42.856983

C 40.475414 16.314550 44.171711

H 38.373024 14.862923 44.789227

H 37.508110 15.848600 43.605534

H 38.980762 14.981243 41.868858

H 39.713665 13.952049 43.093624

H 41.155025 17.169195 44.280251

H 40.796150 15.550844 44.913414

O 14.124641 20.140411 29.234472

C 12.772265 20.479727 29.498003

C 12.011983 19.184067 29.470135

O 12.961481 18.160742 29.210024

C 13.879687 18.959160 28.509878

H 12.728262 21.019991 30.453859

H 12.419749 21.190628 28.728432

H 11.257567 19.180157 28.663893

H 11.482963 18.934225 30.401052

H 13.511525 19.186512 27.483622

H 14.819407 18.405695 28.388626

O 39.875011 30.143389 6.918331

C 40.718410 31.152536 6.387355

C 40.017780 32.459930 6.646487

O 38.753899 32.143105 7.212177

C 38.649757 30.815664 6.762363

H 41.708004 31.052307 6.856237

H 40.870956 30.966255 5.309758

H 39.856434 33.048599 5.727791

H 40.540443 33.122429 7.351033

H 38.318752 30.796108 5.701878

H 37.882629 30.298868 7.354455

O 34.139397 26.112667 35.102585

C 34.043568 24.757359 34.707043

C 33.430450 24.081659 35.889759

O 33.809372 24.842920 37.042160

C 34.560364 25.870159 36.424644

H 33.454899 24.692329 33.781502

H 35.050270 24.366217 34.467468

H 33.729675 23.034376 36.033993

H 32.331341 24.087526 35.824734

H 35.638451 25.612494 36.439312

H 34.445953 26.788853 37.013805

O 34.561340 9.481292 41.316860

C 34.233269 9.309189 39.948841

C 33.177673 8.241859 39.929771

O 33.114605 7.696215 41.246452

C 34.365772 8.147496 41.712891

H 33.920357 10.280200 39.537651

H 35.136322 9.013388 39.383148

H 33.391338 7.435617 39.209091

H 32.179771 8.627134 39.682373

H 35.186680 7.494777 41.341797

H 34.386795 8.088824 42.810047

O 3.177054 35.670719 45.452213

C 2.729685 36.848549 46.110798

C 3.963250 37.570206 46.571369

O 5.038403 36.672043 46.345486

C 4.506449 36.047195 45.204327

H 2.098968 37.426460 45.418968

H 2.070610 36.544434 46.932198

H 3.956894 37.843025 47.634789

H 4.148065 38.506012 46.011547

H 5.091207 35.147076 44.969151

H 4.578322 36.713600 44.319366

O 43.511169 40.911537 14.886391

C 42.171017 40.453903 14.840919

C 42.058563 39.696064 13.547216

O 43.218300 40.024624 12.788401

C 43.621178 41.165291 13.506146

H 41.961758 39.874523 15.749837

H 41.493851 41.327614 14.875145

H 41.163338 39.956661 12.961481

H 42.036564 38.603310 13.666515

H 43.021263 42.055630 13.209856

H 44.665527 41.395088 13.261194

O 44.063656 3.887955 5.665209

C 44.660637 3.032820 6.627908

C 46.101021 2.885164 6.217697

O 46.231564 3.522726 4.951862

C 44.877724 3.548151 4.569521

H 44.502716 3.471878 7.621899

H 44.125263 2.067188 6.635242

H 46.419315 1.833969 6.134090

H 46.822189 3.356002 6.895351

H 44.577030 2.569317 4.141220

H 44.746201 4.302567 3.780880

O 8.683361 32.612476 27.602428

C 9.079882 31.484028 28.356846

C 9.118996 30.338470 27.394146

O 8.372892 30.747215 26.253477

C 7.795468 31.931398 26.752672

H 10.049426 31.701113 28.812525

H 8.365069 31.323660 29.185579

H 8.699007 29.403641 27.792624

H 10.138900 30.092541 27.062654

H 6.845970 31.705023 27.279737

H 7.542203 32.589008 25.914162

O 6.194229 31.411667 43.337112

C 5.069695 31.576925 44.197624

C 5.356206 30.795618 45.446835

O 6.371221 29.877411 45.083561

C 7.069410 30.734993 44.214249

H 4.896614 32.650124 44.369728

H 4.197447 31.205830 43.644646

H 4.502049 30.234819 45.853134

H 5.726325 31.437092 46.268234

H 7.771022 30.147299 43.609932

H 7.669324 31.459093 44.804382

O 35.105518 33.882710 14.027345

C 34.551075 32.598785 14.268386

C 35.717659 31.646355 14.271809

O 36.844147 32.386589 13.819061

C 36.159649 33.449032 13.203990

H 33.974140 32.634964 15.203705

H 33.823059 32.360676 13.469966

H 35.569511 30.778505 13.606378

H 35.962608 31.235163 15.261888

H 35.781216 33.152740 12.200220

H 36.862724 34.277275 13.048511

O 27.068031 34.133041 18.530859

C 26.124401 34.279232 19.582056

C 26.256899 35.696144 20.072451

O 27.235245 36.301926 19.242739

C 27.065100 35.477104 18.118204

H 25.114765 34.072414 19.187490

H 26.322416 33.496944 20.325226

H 26.583014 35.812996 21.114845

H 25.306423 36.251076 19.988842

H 27.888453 35.650185 17.410725

H 26.123911 35.723526 17.580873

O 9.472490 35.102097 0.480616

C 10.800420 35.372963 0.915273

C 10.820466 35.152946 2.401614

O 9.523829 34.692863 2.747775

C 8.814882 35.316734 1.707825

H 11.479540 34.715843 0.353984

H 11.067373 36.408024 0.638051

H 11.041950 36.077019 2.963881

H 11.552390 34.410263 2.745331

H 8.687761 36.400688 1.902419

H 7.801824 34.899193 1.660888

O 30.916384 31.899130 41.529545

C 31.754894 30.799042 41.182404

C 33.108250 31.364731 40.883671

O 32.892632 32.750843 40.679298

C 31.892773 32.904854 41.657642

H 31.759295 30.072983 42.009182

H 31.294325 30.295935 40.320427

H 33.600109 30.942297 39.995777

H 33.812794 31.221966 41.725113

H 31.410690 33.885155 41.536877

H 32.333298 32.890675 42.676079

O 15.286823 11.963090 37.628387

C 14.883457 11.515721 36.344463

C 15.352340 10.088540 36.237385

O 15.816821 9.710599 37.528156

C 15.200771 10.722680 38.286488

H 13.783857 11.601283 36.257435

H 15.302958 12.201198 35.594936

H 16.180584 9.934039 35.531372

H 14.550497 9.397685 35.928383

H 15.708767 10.796508 39.256031

H 14.140286 10.465504 38.495255

O 21.855082 43.443211 39.779182

C 21.796410 44.859631 39.885277

C 22.492153 45.401855 38.666870

O 22.913610 44.274384 37.913433

C 23.006018 43.357159 38.973915

H 20.744236 45.157391 39.991867

H 22.289249 45.173035 40.821575

H 23.374180 46.012524 38.929916

H 21.871706 46.026703 38.010731

H 23.927158 43.540016 39.569920

H 23.086691 42.339699 38.565662

O 9.450978 46.671600 39.574318

C 8.193455 46.254543 40.082806

C 8.310309 46.330326 41.579903

O 9.687130 46.552303 41.868370

C 10.231796 46.168980 40.629429

H 7.407748 46.887218 39.644238

H 7.991039 45.223885 39.735176

H 7.980283 45.404785 42.081055

H 7.738752 47.152214 42.036072

H 10.310513 45.063515 40.567333

H 11.252678 46.565502 40.544353

O 46.330326 39.719044 31.963179

C 45.901047 38.417515 31.606262

C 45.724056 38.460056 30.119431

O 45.654140 39.836384 29.755180

C 45.530933 40.413319 31.034704

H 46.639820 37.688034 31.968069

H 44.952530 38.187233 32.127460

H 44.816120 37.947655 29.769360

H 46.565502 38.017574 29.567432

H 44.468491 40.415279 31.353973

H 45.854603 41.463047 30.988745

O 8.828573 29.543964 33.112648

C 8.147007 30.704679 33.555618

C 6.741339 30.251442 33.803017

O 6.577059 29.020323 33.103848

C 7.764177 29.027164 32.348454

H 8.663316 31.109020 34.435688

H 8.202256 31.486963 32.774311

H 5.975189 30.971632 33.476902

H 6.538923 30.034357 34.857143

H 7.630699 29.618280 31.419003

H 7.996906 27.998951 32.038475

O 37.955482 21.811567 26.404558

C 37.069054 21.004837 25.634983

C 36.676445 21.806677 24.429287

O 37.631809 22.848583 24.361814

C 37.756973 23.044155 25.751348

H 37.605408 20.078806 25.382698

H 36.216854 20.720768 26.268635

H 35.663387 22.241823 24.524139

H 36.682312 21.254189 23.479300

H 36.856861 23.555573 26.156179

H 38.614067 23.701763 25.952787

O 17.027895 2.213866 47.719372

C 17.194130 3.590198 48.017128

C 18.673138 3.812660 48.159409

O 19.295544 2.573717 47.845516

C 18.259016 2.021228 47.068607

H 16.758007 4.193536 47.200623

H 16.610350 3.819017 0.024934

H 18.996319 4.112373 0.273798

H 19.045700 4.590056 47.470509

H 18.439919 0.944609 46.939041

H 18.238970 2.468109 46.050175

O 14.197491 39.161179 7.103146

C 12.795734 38.910847 7.112925

C 12.270137 39.447689 8.415917

O 13.411783 39.820251 9.166422

C 14.227316 40.174725 8.079535

H 12.340054 39.411018 6.242633

H 12.642700 37.831779 6.965757

H 11.677556 38.742653 9.014854

H 11.631598 40.337540 8.269239

H 15.258954 40.314068 8.426186

H 13.902179 41.146225 7.654657

O 28.807638 2.378635 18.179810

C 29.085836 1.015503 18.476099

C 27.776487 0.397498 18.894623

O 26.790812 1.400290 18.698072

C 27.625896 2.505756 18.932758

H 29.546406 0.559823 17.587229

H 29.851007 0.971500 19.271585

H 27.779911 0.103164 19.958530

H 27.480198 48.389690 18.337732

H 27.849827 2.605986 20.017200

H 27.110081 3.427385 18.633535

O 35.779263 5.139611 42.390545

C 36.473537 3.904090 42.480999

C 35.544575 2.952636 43.186520

O 34.427864 3.731498 43.599667

C 35.050758 4.992932 43.582554

H 36.792320 3.602421 41.472340

H 37.394680 4.062992 43.068687

H 36.012970 2.490110 44.073437

H 35.174458 2.117547 42.572914

H 35.706413 5.128366 44.472893

H 34.280697 5.774729 43.635357

O 10.042582 2.351255 41.599461

C 9.897858 0.994480 41.186806

C 8.997742 0.994480 39.987465

O 8.888711 2.349299 39.584591

C 9.001654 2.942858 40.856289

H 10.900649 0.608715 40.958965

H 9.520895 0.395543 42.030205

H 7.994462 0.586714 40.194283

H 9.383995 0.412166 39.142109

H 8.042377 2.871963 41.405357

H 9.213848 4.014588 40.737480

O 22.813869 7.244446 26.440739

C 21.910330 8.170476 25.864290

C 22.556692 9.489114 26.112179

O 23.966272 9.256873 26.086266

C 23.992186 7.852673 25.965498

H 21.788099 7.953392 24.786205

H 20.919273 8.054600 26.322416

H 22.292183 9.873900 27.108612

H 22.315163 10.280689 25.390520

H 24.851721 7.463486 26.529232

H 24.152554 7.584740 24.901592

O 35.124096 36.433937 48.211235

C 34.869858 36.828503 46.875481

C 33.374714 36.807480 46.737118

O 32.852539 36.256943 47.941345

C 34.016186 35.582226 48.358402

H 35.343628 37.805382 46.700935

H 35.360741 36.123466 46.181206

H 33.027573 36.179691 45.903004

H 32.936146 37.803913 46.590927

H 34.151619 34.642990 47.779999

H 33.916447 35.294247 0.522173

O 14.577876 38.918667 35.750904

C 15.972788 38.646336 35.840866

C 16.135113 37.158527 35.656052

O 14.838964 36.656399 35.377853

C 14.116817 37.630341 36.089729

H 16.493986 39.260918 35.091827

H 16.335573 38.990540 36.824100

H 16.512077 36.658844 36.565460

H 16.810322 36.858814 34.840519

H 14.187713 37.455795 37.182484

H 13.054867 37.545761 35.831577

O 23.706163 13.881643 22.238401

C 24.055258 15.186104 21.793964

C 23.034376 16.119955 22.392412

O 22.193909 15.311269 23.204033

C 22.338631 14.127086 22.455486

H 25.094719 15.381674 22.094166

H 24.043522 15.204682 20.688988

H 22.412460 16.611328 21.620396

H 23.448008 16.931576 23.007975

H 21.788588 14.203358 21.490831

H 21.899084 13.285641 23.010418

O 0.396033 4.885858 37.371212

C 48.699673 3.962273 38.277195

C 47.797112 4.759714 39.177311

O 48.015663 6.123823 38.846794

C 48.352535 5.926296 37.494907

H 48.161854 3.198078 37.698792

H 0.610673 3.422007 38.795948

H 47.977039 4.627214 40.253933

H 46.732227 4.518184 39.017433

H 48.800392 6.845970 37.093987

H 47.442638 5.715079 36.894016

O 32.184174 22.073631 39.840786

C 33.429962 21.870728 40.489105

C 33.864620 23.231903 40.962387

O 32.813423 24.131041 40.625519

C 31.769562 23.187899 40.589825

H 33.296486 21.154448 41.321751

H 34.114464 21.387177 39.778202

H 34.787716 23.605444 40.502308

H 34.039169 23.266617 42.050255

H 30.887535 23.633312 40.115562

H 31.451271 22.894543 41.611683

O 34.479691 47.753597 23.215279

C 33.623577 48.663002 22.541536

C 34.442532 1.005725 22.293650

O 35.770462 0.700145 22.701904

C 35.695656 48.187279 22.655457

H 33.250526 48.196079 21.611595

H 32.744976 48.832661 23.177143

H 34.107616 1.879928 22.868628

H 34.448891 1.325484 21.239031

H 36.531231 47.766308 23.232880

H 35.804195 47.816666 21.613062

O 3.722209 13.096914 9.730156

C 2.493533 12.442728 9.450489

C 2.789823 11.420380 8.390493

O 4.177890 11.526966 8.115227

C 4.577344 12.042296 9.360038

H 2.099946 11.994382 10.380430

H 1.768942 13.203012 9.139531

H 2.241246 11.573903 7.451263

H 2.560516 10.391187 8.717097

H 5.603115 12.421215 9.280831

H 4.590056 11.247299 10.137922

O 7.123192 46.846638 31.163782

C 5.864202 46.643730 30.545776

C 5.056493 45.827709 31.517277

O 5.831933 45.743614 32.706348

C 6.668000 46.852505 32.492687

H 5.408522 47.626965 30.334070

H 6.029460 46.175827 29.565964

H 4.835009 44.802917 31.185295

H 4.084015 46.284855 31.756363

H 7.529980 46.784054 33.165943

H 6.146314 47.802490 32.736664

O 13.373158 17.622433 32.603676

C 13.677272 18.966005 32.937611

C 15.094185 18.949383 33.434364

O 15.433011 17.580873 33.641178

C 14.140776 17.021540 33.617222

H 13.501746 19.597700 32.057053

H 12.971748 19.315100 33.714520

H 15.226195 19.499428 34.380440

H 15.821710 19.373283 32.725418

H 13.646470 17.136438 34.606323

H 14.219982 15.941986 33.423119

O 38.192608 7.692305 31.048393

C 38.416050 8.275106 29.765936

C 37.370720 7.712839 28.844309

O 36.452515 7.036652 29.685265

C 37.387348 6.609818 30.648451

H 38.372047 9.365416 29.887678

H 39.440353 8.031620 29.439331

H 37.813690 6.993626 28.130472

H 36.822144 8.450143 28.238525

H 38.012196 5.780596 30.248018

H 36.859791 6.216720 31.528522

O 18.125050 8.355290 13.999475

C 18.700518 9.610857 13.673360

C 19.503338 9.392796 12.418772

O 19.377193 8.013041 12.110258

C 18.115759 7.811113 12.702349

H 17.888899 10.344250 13.530105

H 19.272564 9.960930 14.544141

H 20.574089 9.628948 12.495045

H 19.118551 9.989287 11.571948

H 17.905033 6.734005 12.763465

H 17.306583 8.256527 12.081410

O 41.251343 13.898755 19.752203

C 42.081055 14.759758 20.518353

C 41.979359 16.118000 19.878345

O 41.123734 15.970345 18.753811

C 41.344242 14.598412 18.533794

H 41.742226 14.721622 21.561726

H 43.113670 14.366660 20.513952

H 42.956722 16.490564 19.532675

H 41.565723 16.905174 20.525686

H 42.333344 14.425332 18.062468

H 40.589825 14.219004 17.830225

O 43.555172 9.114106 5.114676

C 43.521435 8.324488 6.301304

C 42.931301 7.001449 5.919940

O 42.199375 7.261069 4.735268

C 43.133228 8.153852 4.173489

H 44.538406 8.263861 6.714448

H 42.913208 8.865731 7.040563

H 42.266357 6.549679 6.668489

H 43.708698 6.246544 5.708234

H 42.672169 8.681894 3.332044

H 44.004009 7.601852 3.762790

O 37.329651 33.878799 41.090488

C 37.678257 32.913166 42.067852

C 36.953178 31.653687 41.670357

O 36.136181 31.990070 40.555111

C 36.052574 33.372757 40.798595

H 38.774433 32.832005 42.103058

H 37.371212 33.280350 43.064777

H 36.310726 31.265478 42.480019

H 37.608341 30.824467 41.367222

H 35.339226 33.583977 41.625862

H 35.663876 33.881733 39.907280

O 14.304078 46.582127 38.221947

C 14.293322 47.542381 39.269718

C 15.167524 48.681580 38.816483

O 15.552310 48.377960 37.482197

C 15.505373 46.975712 37.608341

H 13.246526 47.819603 39.455021

H 14.661485 47.069588 40.197704

H 16.073019 48.784260 39.440845

H 14.683975 0.775927 38.826260

H 16.382511 46.607059 38.180386

H 15.559155 46.513187 36.612885

O 9.672462 26.841658 23.931559

C 9.882213 27.613674 25.104986

C 10.737837 28.780746 24.698198

O 11.062974 28.584198 23.333111

C 9.916438 27.852762 22.982061

H 8.908268 27.937346 25.505905

H 10.329093 26.957535 25.865269

H 11.673644 28.889290 25.263397

H 10.197572 29.736601 24.795982

H 10.071427 27.395124 21.996870

H 9.035878 28.524059 22.893564

O 30.643562 14.270341 8.784081

C 30.345806 15.246731 7.800846

C 31.272324 16.402067 8.073668

O 32.041409 16.046127 9.215804

C 31.130045 15.134766 9.781004

H 30.454836 14.779804 6.810767

H 29.281898 15.531775 7.890320

H 30.715921 17.328096 8.297108

H 31.967089 16.645554 7.257158

H 30.293489 15.670631 10.280200

H 31.638529 14.543652 10.556933

O 7.147150 42.754795 44.741798

C 7.120748 41.886951 45.857533

C 8.207146 42.376854 46.772808

O 8.957650 43.330265 46.026703

C 8.536683 42.950367 44.739357

H 6.104266 41.866413 46.269699

H 7.327075 40.861668 45.506973

H 8.868665 41.556927 47.093056

H 7.867340 42.863338 47.697369

H 9.071570 42.033142 44.402973

H 8.783102 43.741943 44.019653

O 4.248785 41.297791 30.581469

C 2.901299 41.630753 30.897316

C 2.360544 40.427010 31.635107

O 3.447431 39.515652 31.706982

C 4.031211 39.910213 30.492485

H 2.888587 42.578781 31.454693

H 2.362500 41.839035 29.953686

H 1.525944 39.931236 31.109510

H 1.987492 40.604004 32.649632

H 3.394138 39.638859 29.616325

H 4.981687 39.382172 30.349226

O 46.951267 23.364401 16.152714

C 48.244480 22.923388 15.741526

C 48.243992 23.002596 14.258118

O 46.918018 22.645189 13.908534

C 46.269215 23.388849 14.916215

H 0.111475 23.527216 16.251966

H 48.379913 21.879528 16.070574

H 0.049379 22.310762 13.770167

H 48.474277 24.022499 13.895334

H 45.259575 22.984505 15.060938

H 46.157738 24.441999 14.593523

O 48.285549 42.938637 32.870628

C 0.180415 42.303516 33.868530

C 48.886444 43.088245 35.138763

O 48.127140 44.225494 34.772560

C 47.401569 43.614334 33.731140

H 48.751011 41.251343 33.959961

H 1.215476 42.296673 33.509171

H 0.921628 43.432941 35.617916

H 48.339333 42.512779 35.907848

H 46.855434 44.382927 33.166428

H 46.647640 42.912724 34.139397

O 22.213955 1.437448 4.370039

C 22.577717 2.675414 3.756923

C 22.697014 2.411882 2.282805

O 22.060919 1.162182 2.077455

C 22.464775 0.569601 3.288041

H 21.804722 3.415651 4.007254

H 23.509125 3.046999 4.209670

H 23.752611 2.343921 1.953756

H 22.219822 3.162386 1.636442

H 23.542372 0.298735 3.247460

H 21.905441 48.528061 3.437164

O 30.178593 38.869778 29.820208

C 30.738903 38.077713 30.860157

C 30.226505 36.677910 30.651873

O 29.491648 36.701870 29.437376

C 29.081436 38.045444 29.512671

H 31.833124 38.184299 30.817131

H 30.431368 38.491344 31.834591

H 29.556677 36.356197 31.470339

H 31.002436 35.904427 30.573647

H 28.273727 38.165718 30.267088

H 28.663404 38.354446 28.545572

O 31.407267 46.905796 12.091678

C 30.056360 47.345341 12.044252

C 30.024580 48.484055 11.058574

O 31.384289 48.778877 10.763749

C 31.861483 47.459263 10.884515

H 29.418798 46.495094 11.750896

H 29.758604 47.613762 13.068556

H 29.538584 0.507507 11.421358

H 29.501425 48.205364 10.123254

H 32.961079 47.458282 10.885004

H 31.532923 46.845657 10.021069

O 24.561296 33.673447 27.867430

C 23.263195 34.236206 28.019976

C 23.061266 35.176414 26.863661

O 24.334435 35.291801 26.253967

C 24.766159 33.982452 26.512119

H 22.521980 33.423607 28.040998

H 23.225546 34.712910 29.010052

H 22.711683 36.183605 27.130613

H 22.335209 34.784782 26.124889

H 25.837891 33.902267 26.287703

H 24.243984 33.251995 25.855978

O 44.701218 21.881483 33.719898

C 44.075882 22.446684 32.573853

C 42.944012 23.296930 33.079403

O 42.954281 23.161985 34.494358

C 44.314476 22.850538 34.662064

H 44.821007 23.023619 32.000828

H 43.767857 21.625284 31.914776

H 41.949043 23.001617 32.715641

H 43.058910 24.363281 32.814404

H 44.469955 22.452063 35.675117

H 44.942261 23.762878 34.573078

O 39.724419 11.267345 24.893280

C 39.377281 11.931799 23.685139

C 40.385941 13.021130 23.477835

O 41.138889 13.065133 24.676685

C 41.061638 11.694669 24.980799

H 39.341591 11.192051 22.869608

H 38.366180 12.329297 23.807371

H 39.948349 14.015121 23.304264

H 41.058216 12.821159 22.623186

H 41.427357 11.526478 26.003633

H 41.711426 11.099643 24.301678

O 40.109207 8.331822 39.024277

C 39.338169 7.189197 38.679581

C 38.627758 7.533403 37.400547

O 39.178780 8.764524 36.948776

C 40.366875 8.746433 37.703194

H 40.006535 6.321350 38.551483

H 38.683983 6.932510 39.522984

H 37.541847 7.669324 37.507133

H 38.771503 6.769697 36.619728

H 40.807888 9.752646 37.695862

H 41.117378 8.075624 37.246532

O 6.352642 1.148003 41.716805

C 4.965552 1.379755 41.886463

C 4.625259 0.845845 43.249104

O 5.851490 0.441991 43.842663

C 6.718359 1.229654 43.071133

H 4.771448 2.464197 41.801388

H 4.429199 0.901583 41.053818

H 3.947116 48.877151 43.239815

H 4.147088 1.594883 43.898888

H 7.743642 0.858069 43.195320

H 6.716893 2.283783 43.421696

O 46.609997 41.665955 17.747108

C 46.722446 42.208664 19.055479

C 46.595814 43.696472 18.899023

O 46.740540 43.972717 17.509489

C 47.248043 42.732796 17.078745

H 45.956787 41.749073 19.694510

H 47.703236 41.927532 19.486713

H 47.364899 44.247005 19.463736

H 45.622849 44.098862 19.218781

H 48.346180 42.690258 17.243025

H 47.078876 42.638435 15.995769

O 35.627697 21.147602 8.889689

C 35.190102 20.703167 7.621899

C 34.938797 19.251541 7.835071

O 35.870689 18.819817 8.835418

C 36.480385 20.058271 9.145398

H 34.315411 21.290369 7.314852

H 35.975323 20.882603 6.862593

H 35.050270 18.634514 6.937399

H 33.920845 19.074059 8.215457

H 37.411301 20.170725 8.554773

H 36.762497 20.064138 10.207350

O 40.556580 12.144972 1.185650

C 40.734547 13.433786 1.756718

C 41.287525 14.313367 0.668853

O 41.355000 13.495879 48.403873

C 41.520741 12.265248 0.170147

H 39.772827 13.760389 2.167418

H 41.413177 13.346757 2.619187

H 42.299603 14.688864 0.903539

H 40.682720 15.199794 0.430746

H 42.551403 12.166485 0.579380

H 41.384823 11.448249 48.344223

O 25.035557 35.650185 39.311768

C 26.416290 35.954296 39.423241

C 26.752186 35.843800 40.885624

O 25.519596 35.640896 41.565723

C 24.646372 36.124935 40.575157

H 26.972202 35.278111 38.759766

H 26.586927 36.974201 39.038944

H 27.235733 36.754185 41.280190

H 27.417614 35.006268 41.141335

H 24.615078 37.235287 40.591778

H 23.626957 35.778774 40.790287

O 33.478855 40.003597 21.047861

C 33.948715 38.813061 21.678579

C 34.426395 37.899742 20.582401

O 34.374084 38.669315 19.390884

C 33.291595 39.489246 19.750246

H 34.731487 39.096638 22.397303

H 33.135139 38.372047 22.278004

H 33.796658 37.001095 20.460659

H 35.450211 37.521801 20.713924

H 32.337700 38.926491 19.678862

H 33.226082 40.328735 19.044235

O 44.534985 37.671413 26.205563

C 45.731880 37.291027 26.856325

C 46.444248 36.448605 25.850601

O 45.963631 36.852947 24.568142

C 45.111431 37.912945 24.944618

H 46.312241 38.192123 27.135994

H 45.478127 36.773739 27.790668

H 46.215916 35.380299 25.972834

H 47.537491 36.537590 25.876026

H 44.313011 38.016594 24.197535

H 45.670761 38.873199 24.949995

O 16.530655 34.501202 47.256359

C 16.731117 33.597668 48.327110

C 17.957836 34.087082 0.151080

O 18.281507 35.357803 48.486500

C 17.049898 35.651649 47.875340

H 15.835400 33.599621 0.082630

H 16.806900 32.583630 47.910053

H 18.840351 33.440231 0.034714

H 17.794535 34.200516 1.235031

H 17.201464 36.430511 47.115547

H 16.329706 36.059906 48.617531

O 0.489906 31.520210 44.705620

C 1.765519 30.892916 44.724686

C 1.567992 29.555696 45.356873

O 0.205839 29.248650 45.122677

C 48.605309 30.547733 45.359318

H 2.114125 30.828867 43.684250

H 2.471531 31.540745 45.257618

H 1.765030 29.593346 46.445713

H 2.187464 28.747988 44.947151

H 48.572060 30.760416 46.447670

H 47.580029 30.619604 44.981377

O 1.273166 13.498324 4.326524

C 48.768612 13.763324 4.238028

C 48.354980 14.365194 5.550311

O 0.543686 14.132464 6.437226

C 1.572395 14.249806 5.482839

H 48.584286 14.432666 3.383382

H 48.264526 12.812358 4.010187

H 47.441662 13.934937 5.984968

H 48.178474 15.454036 5.468660

H 2.515046 13.882133 5.913584

H 1.734228 15.316648 5.217840

O 21.704491 2.725774 27.494865

C 20.501728 3.356002 27.062654

C 19.684240 2.304318 26.383045

O 20.215216 1.074664 26.846060

C 21.561235 1.485852 26.840679

H 20.009867 3.770613 27.953482

H 20.754015 4.203803 26.409935

H 19.780071 2.359566 25.282465

H 18.608110 2.343921 26.599640

H 21.947489 1.543546 25.799751

H 22.175329 0.744637 27.366766

O 2.605008 31.227343 22.966413

C 3.110560 31.843391 21.786142

C 1.917576 32.269737 20.975500

O 0.797930 31.667868 21.605240

C 1.294192 31.739738 22.920456

H 3.757901 32.686790 22.073631

H 3.763768 31.117823 21.280592

H 1.934688 31.962202 19.919905

H 1.788499 33.369827 20.972076

H 0.651741 31.145201 23.585398

H 1.264857 32.786045 23.294485

O 37.566292 38.978317 0.565690

C 37.734486 39.584099 48.183365

C 37.950100 41.011772 48.509968

O 38.884445 40.958965 0.684499

C 38.425831 39.794827 1.343085

H 38.623356 39.162151 47.677322

H 36.857349 39.359680 47.563892

H 37.017715 41.490917 48.858574

H 38.360802 41.625374 47.699326

H 37.879208 40.090141 2.251024

H 39.301987 39.213493 1.665778

O 42.177860 6.989225 25.971365

C 41.727558 8.322533 26.162537

C 40.276421 8.334267 25.770906

O 39.913635 6.978469 25.565067

C 41.169693 6.550658 25.095207

H 41.924599 8.596332 27.208843

H 42.341652 8.998719 25.544041

H 40.112633 8.887244 24.827763

H 39.596321 8.775280 26.513098

H 41.354019 6.913930 24.061613

H 41.190228 5.454481 25.044357

Li 2.447085 11.814456 2.733596

Li 32.102524 16.068130 15.914117

Li 46.250633 39.661350 17.736841

Li 39.790428 22.068743 13.606378

Li 20.936384 0.379897 6.597106

Li 39.309811 48.703094 0.110498

Li 6.649421 5.953676 31.698181

Li 24.252783 48.204880 13.619578

Li 6.248500 0.397007 12.983482

Li 24.403374 41.952465 16.753607

Li 44.385372 27.127682 16.457317

Li 45.100674 39.543030 3.099805

Li 34.543743 36.116135 5.136678

Li 18.006239 2.335120 35.799797

Li 43.979073 47.634789 11.302548

Li 19.500404 15.367985 11.575859

Li 1.059996 41.808720 23.651403

Li 30.939852 12.815781 1.344554

Li 27.201019 6.161470 24.279675

Li 14.728956 34.310520 19.443689

Li 28.426273 38.392582 13.420094

Li 30.759438 9.059346 30.384430

Li 12.449084 2.549760 18.285908

Li 44.228428 38.547081 42.017982

Li 48.891335 16.407934 12.406548

Li 45.914742 26.038349 1.906819

Li 30.131166 32.700481 21.684444

Li 14.968530 37.082256 29.115662

Li 31.232231 7.417527 41.929485

Li 20.861092 34.308567 4.260519

Li 32.710262 1.378288 11.229209

Li 12.204132 7.434150 47.329208

Li 24.096817 40.209438 43.996677

Li 27.812181 42.609585 45.479595

Li 45.387672 29.182156 8.114738

Li 30.738903 17.518290 41.738316

Li 36.801121 28.388136 21.967047

Li 11.946956 4.717666 28.995874

Li 9.315056 35.047825 47.341431

Li 36.210007 14.709887 21.976336

Li 8.765501 9.806918 27.125723

Li 16.658754 41.677689 21.470295

Li 13.853774 28.801769 44.060234

Li 27.880142 11.601283 24.567165

Li 19.860254 15.305402 3.985252

Li 45.551464 24.433199 26.448071

Li 19.882746 44.413731 26.946289

Li 38.521168 9.541430 10.154057

Li 38.999828 2.573228 8.843241

Li 11.311349 37.035805 15.931719

Li 43.538059 18.297152 30.995100

Li 48.020065 27.103724 41.112976

Li 41.146225 8.649137 41.046970

Li 20.831266 16.021681 24.414131

Li 3.383384 43.813816 46.231075

Li 44.185890 46.163605 40.411854

Li 11.681468 42.978725 18.759678

Li 33.728210 18.900978 37.454327

Li 18.207678 39.460400 38.301640

Li 36.576214 17.127148 10.470881

Li 34.231804 46.628082 26.860237

Li 4.372972 23.401072 17.583807

Li 0.084583 9.430932 23.700294

Li 34.243050 0.577913 5.696989

Li 21.857037 40.204548 10.310513

Li 31.020525 36.576214 48.637089

Li 4.885368 41.227386 32.760620

Li 36.538078 45.258598 4.861900

Li 4.482003 7.845827 40.155167

Li 13.708074 7.856095 3.693362

Li 14.386217 32.063896 30.083740

Li 33.177185 13.520326 27.081722

Li 33.880264 39.343056 12.038384

Li 8.429118 28.240480 36.403622

Li 9.627970 10.627339 39.633480

Li 33.372269 25.325005 39.122063

Li 18.413029 16.670488 37.896320

Li 25.253620 35.183746 2.916456

Li 47.760929 17.868362 0.077251

Li 28.033176 3.966673 12.372812

Li 5.781085 32.387566 41.049416

Li 39.719044 7.838983 33.377647

Li 12.823114 41.346684 36.099022

Li 33.436806 2.390858 36.618263

Li 0.772506 36.026173 7.372056

Li 6.856726 48.194611 27.634209

Li 32.915611 25.529375 15.943454

Li 32.675545 42.271248 20.643028

Li 17.755909 38.566151 5.540043

Li 7.385257 21.736273 41.571594

Li 6.544301 11.495186 28.655092

N 9.083304 32.644257 38.325111

S 9.410398 33.538506 37.149727

S 7.607719 32.471664 38.567131

O 8.531304 33.330711 36.034973

O 10.806287 33.425072 36.811878

O 6.845481 33.591797 38.174522

O 7.368634 32.050209 39.931728

C 9.196246 35.254154 37.836182

C 7.184797 31.018082 37.462154

F 8.766968 35.277622 39.111305

F 10.369674 35.927898 37.805866

F 8.331333 35.961143 37.089100

F 8.208611 30.515463 36.747341

F 6.214275 31.377932 36.604084

F 6.711026 29.992798 38.192608

N 22.578205 45.876602 30.242641

S 21.923531 46.991848 29.484802

S 21.750452 44.622993 30.364874

O 22.631498 48.237148 29.666683

O 20.514441 47.109188 29.797230

O 22.590918 43.466679 30.562401

O 20.796062 44.463112 29.281898

C 22.142082 46.449139 27.717329

C 20.823933 44.917328 31.946066

F 22.781601 45.270821 27.575539

F 20.939808 46.299038 27.132080

F 22.822670 47.361965 26.998116

F 21.131468 43.968807 32.852051

F 19.489159 44.849854 31.748049

F 21.082087 46.109333 32.518112

N 10.021557 8.411028 29.433954

S 10.687966 7.469841 30.411810

S 9.405996 9.634815 30.049515

O 11.417446 8.181721 31.434160

O 11.481497 6.479274 29.720465

O 8.893111 9.388885 31.374512

O 8.401738 10.172147 29.146465

C 9.247095 6.563369 31.152046

C 10.824376 10.834644 30.112099

F 8.060956 6.899752 30.594179

F 9.400129 5.240330 30.941319

F 9.113129 6.724226 32.480465

F 11.059062 11.272235 31.357885

F 10.527597 11.914686 29.357681

F 11.971890 10.329093 29.617302

N 9.166911 13.885555 37.207909

S 9.612813 14.054235 35.775837

S 7.852184 13.190300 37.384411

O 9.317501 12.874942 34.994045

O 11.010658 14.425820 35.719124

O 6.893396 13.491479 36.351795

O 7.347610 13.394183 38.722607

C 8.596821 15.491684 35.168102

C 8.322043 11.398868 37.257290

F 7.799868 16.028526 36.116623

F 9.390841 16.481274 34.714375

F 7.816003 15.123521 34.136463

F 7.669324 10.753971 36.273079

F 7.995440 10.781840 38.414585

F 9.646061 11.172983 37.112568

N 25.504930 45.724545 43.632423

S 24.370127 44.770649 43.402626

S 26.721872 45.485462 42.800266

O 24.323679 44.271454 42.046829

O 23.134117 45.345139 43.858307

O 27.032829 44.075394 42.715195

O 27.831249 46.258942 43.297504

C 24.783760 43.354713 44.530586

C 26.258854 46.165558 41.126179

F 25.962078 43.495522 45.181347

F 23.839151 43.176254 45.472748

F 24.845854 42.197418 43.834839

F 25.010622 46.672089 41.062130

F 26.365932 45.219482 40.171791

F 27.091990 47.169819 40.774639

N 1.663822 35.211617 40.541420

S 2.951169 34.846390 41.225922

S 0.989101 34.108109 39.787003

O 3.006907 33.499878 41.744183

O 3.273862 35.851131 42.215023

O 0.526087 33.038822 40.642632

O 1.786543 33.641178 38.688381

C 4.235583 34.987686 39.897991

C 48.406319 35.019958 39.120106

F 3.788214 35.209171 38.654648

F 5.100008 35.976299 40.183525

F 4.937195 33.837730 39.904346

F 47.252445 34.448891 39.519562

F 48.411205 35.006756 37.771641

F 48.352043 36.317081 39.496094

N 14.278165 32.787022 20.708546

S 15.165079 31.793030 21.456116

S 13.278795 32.169018 19.747313

O 14.504048 30.529152 21.699112

O 15.709256 32.396366 22.635410

O 13.824927 31.014168 19.070637

O 12.834359 33.196743 18.855509

C 16.552168 31.559813 20.239664

C 11.856014 31.691824 20.847401

F 16.706181 30.279310 19.869545

F 16.376154 32.300541 19.122463

F 17.729507 31.955847 20.771128

F 12.078477 32.009140 22.137682

F 11.566569 30.385897 20.774549

F 10.744193 32.370453 20.481682

N 31.144714 36.577190 11.595416

S 31.875660 36.788410 12.893032

S 30.506662 37.804401 11.019948

O 32.515667 38.092381 12.942902

O 32.787510 35.708366 13.158031

O 29.953686 38.657093 12.055498

O 29.539564 37.481709 10.002000

C 30.494440 36.706760 14.138821

C 31.954376 38.639980 10.201482

F 29.278475 36.508743 13.578997

F 30.681211 35.731346 15.044803

F 30.397633 37.873341 14.803761

F 33.116070 37.964767 10.333493

F 32.164131 39.853012 10.757394

F 31.748049 38.824306 8.888222

N 7.216088 23.405962 15.593381

S 6.690979 24.674730 16.187918

S 7.976371 22.518068 16.537991

O 6.051950 24.431242 17.470863

O 5.779618 25.296646 15.263843

O 8.608067 23.249016 17.604830

O 8.865731 21.661467 15.798731

C 8.199812 25.757706 16.360020

C 6.628886 21.470783 17.273336

F 9.349770 25.170990 15.961054

F 8.070734 26.873438 15.619294

F 8.357246 26.136623 17.636612

F 6.606395 21.592037 18.612511

F 6.776053 20.164368 16.987314

F 5.393854 21.832102 16.850414

N 28.851641 14.241982 42.874584

S 28.399384 14.276209 41.439091

S 28.859951 15.569912 43.571796

O 27.338408 15.228150 41.213207

O 28.095757 12.944857 40.973145

O 29.244249 16.655821 42.688793

O 29.676952 15.500484 44.759403

C 29.922882 14.847276 40.540932

C 27.096390 15.782597 44.109127

F 29.680864 15.977678 39.843719

F 30.967722 15.121566 41.352062

F 30.336515 13.917825 39.658417

F 26.591326 16.920330 43.604065

F 27.016693 15.868647 45.455147

F 26.287212 14.767582 43.743412

N 31.278679 13.174653 26.092621

S 29.909191 13.848886 25.973810

S 32.087856 13.052911 24.814075

O 28.873644 12.889119 25.667253

O 29.912615 15.005199 25.119164

O 33.400627 12.605542 25.213039

O 32.147995 14.281097 24.070414

C 29.689175 14.412130 27.724174

C 31.278189 11.720582 23.794170

F 30.738903 14.066459 28.504501

F 29.565477 15.747882 27.798981

F 28.592510 13.865998 28.269815

F 30.155613 11.207208 24.337858

F 32.123547 10.688455 23.617178

F 30.952074 12.180174 22.573805

N 5.854424 18.977251 24.928482

S 5.904294 19.849010 23.695894

S 6.801477 19.379150 26.031017

O 6.156581 21.237076 24.006853

O 4.718644 19.656372 22.892097

O 8.060956 19.882257 25.527908

O 6.967713 18.292263 26.966335

C 7.345654 19.136152 22.758619

C 5.873981 20.735926 26.901306

F 8.309331 20.053381 22.540558

F 7.922589 18.081045 23.376135

F 6.939844 18.697094 21.550480

F 6.570703 21.888329 26.903751

F 5.650541 20.403944 28.191099

F 4.665840 20.994080 26.361532

N 35.719124 43.386494 4.856522

S 34.984264 43.279907 3.550106

S 36.680847 42.258537 5.158679

O 35.838421 42.782177 2.487666

O 34.381905 44.553562 3.249904

O 36.278458 41.001991 4.540185

O 36.895485 42.143639 6.574615

C 33.624557 42.064922 3.893334

C 38.242970 42.884853 4.395952

F 33.636292 41.585281 5.159657

F 32.410549 42.631588 3.714875

F 33.681759 41.002480 3.069001

F 38.711365 42.073231 3.440587

F 39.208115 43.014416 5.329804

F 38.064510 44.116951 3.861064

N 41.674267 7.158884 33.301376

S 42.324051 7.696704 34.558411

S 42.112835 5.743437 32.909252

O 42.162216 6.797565 35.667786

O 41.748585 8.993341 34.836117

O 43.046688 5.152812 33.826973

O 42.523533 5.718502 31.529013

C 44.126728 7.922589 34.148197

C 40.481773 4.874612 33.020729

F 44.457733 7.520690 32.908768

F 44.470448 9.223626 34.244518

F 44.902168 7.258136 35.025333

F 39.508804 5.742459 33.396225

F 40.496925 3.876221 33.916935

F 40.096985 4.368572 31.833124

N 48.213676 14.423864 47.559490

S 48.389690 14.718200 0.129567

S 47.599098 15.549377 46.761074

O 48.688427 16.117022 0.367672

O 0.444433 13.813684 0.740240

O 46.658398 16.324327 47.547268

O 47.067142 15.069738 45.507462

C 46.702404 14.357370 0.828734

C 0.142769 16.663645 46.404156

F 46.184139 15.476037 1.379758

F 45.815975 13.904134 48.812614

F 46.765961 13.437209 1.808057

F 1.308370 16.225565 46.910686

F 48.788170 17.867386 46.974735

F 0.308023 16.873394 45.088451

N 21.196495 36.328815 4.435555

S 20.469460 37.424503 3.696784

S 22.057497 36.818726 5.596270

O 21.383266 38.339291 3.074379

O 19.420219 38.022953 4.502538

O 22.249645 35.707878 6.485630

O 21.532389 37.998016 6.243610

C 19.652948 36.408512 2.386946

C 23.715942 37.211819 4.850655

F 20.043114 36.780098 1.159737

F 19.946306 35.095737 2.535092

F 18.314754 36.526344 2.459308

F 23.760923 37.081276 3.513926

F 24.655659 36.364017 5.323937

F 24.114416 38.457119 5.156723

N 25.622271 44.145309 14.230248

S 24.275274 43.642693 14.642415

S 25.917095 45.574444 14.590100

O 23.843552 44.190777 15.902383

O 24.273808 42.200356 14.675662

O 24.762247 46.442291 14.424843

O 27.057764 46.053104 13.853774

C 23.168343 44.202515 13.261683

C 26.409445 45.479595 16.385445

F 22.175329 44.983822 13.720786

F 23.829372 44.903145 12.322941

F 22.601673 43.146919 12.644167

F 25.609070 46.264812 17.129591

F 27.667458 45.935764 16.552168

F 26.361042 44.236740 16.902731

N 14.060102 34.093925 29.096594

S 14.780782 33.556107 27.878185

S 13.108159 35.241444 28.776834

O 13.985785 33.630421 26.684223

O 15.247708 32.213512 28.194521

O 13.681183 36.156223 27.832226

O 12.764443 35.935230 29.993776

C 16.245611 34.672817 27.711948

C 11.582215 34.414665 28.137806

F 16.250500 35.636494 28.673672

F 17.400459 34.000053 27.835649

F 16.265656 35.306469 26.527277

F 11.268812 34.840519 26.900818

F 10.529553 34.693352 28.932802

F 11.724004 33.081844 28.122160

N 37.989216 47.251957 48.231770

S 38.507969 47.504242 46.851036

S 37.868938 45.788597 48.627800

O 39.518581 46.559143 46.454514

O 38.974895 48.868843 46.808010

O 37.601986 44.927593 47.497398

O 36.927753 45.638496 0.813574

C 37.003536 47.347298 45.770016

C 39.570408 45.477150 0.411677

F 35.894650 46.996250 46.449627

F 36.732182 48.529037 45.170593

F 37.185905 46.443760 44.788738

F 40.204548 44.491959 48.654690

F 39.524937 45.147121 1.715157

F 40.340469 46.586037 0.322204

N 30.186415 15.207128 15.784552

S 29.813362 14.004365 14.919148

S 29.810917 15.073161 17.238625

O 29.584055 14.420932 13.561396

O 28.742611 13.212790 15.477505

O 30.504707 16.097466 17.971527

O 30.072493 13.749633 17.754930

C 31.415579 13.070024 14.981243

C 27.989172 15.450613 17.259647

F 32.321564 13.726654 15.745437

F 31.275747 11.831079 15.480927

F 31.969044 12.966860 13.758924

F 27.492422 15.701922 16.033903

F 27.751064 16.546301 18.010641

F 27.273869 14.443911 17.792089

N 13.193233 5.703834 6.961845

S 12.376234 5.572802 5.713124

S 14.536318 6.363398 6.775075

O 12.582561 6.685112 4.801762

O 10.988168 5.325404 6.009903

O 15.136723 6.000613 5.513641

O 15.413943 6.133113 7.897165

C 13.075401 4.046857 4.923016

C 14.125619 8.175364 6.698313

F 14.073792 3.457699 5.609960

F 12.133237 3.105671 4.721578

F 13.562374 4.384706 3.712919

F 12.822137 8.454054 6.869438

F 14.458579 8.650114 5.471104

F 14.835542 8.889689 7.587185

N 11.671200 37.894363 45.874161

S 12.008560 36.437359 45.903492

S 12.462775 38.704029 44.850342

O 12.241779 35.902470 44.584366

O 11.000391 35.690277 46.634441

O 13.781902 38.164742 44.605881

O 12.490644 40.093559 45.211658

C 13.575086 36.416336 46.904331

C 11.436515 38.549526 43.313152

F 14.591078 35.915672 46.188538

F 13.949605 37.632298 47.355610

F 13.422540 35.637474 47.997082

F 12.158661 38.033222 42.299118

F 11.000391 39.758160 42.903919

F 10.351095 37.772621 43.480854

N 18.345066 40.102852 24.620949

S 18.524015 38.686428 24.130552

S 18.961605 41.121292 23.743811

O 18.767502 38.605267 22.705816

O 17.416594 37.873829 24.562765

O 18.129450 41.355972 22.586029

O 20.349672 40.867046 23.466099

C 20.037247 38.156429 25.071739

C 18.846218 42.628162 24.810162

F 21.027327 37.748665 24.258652

F 20.540842 39.141621 25.848646

F 19.747801 37.132126 25.903404

F 18.008684 43.532681 24.267941

F 20.041647 43.237370 24.955374

F 18.400805 42.351433 26.046659

N 48.587219 30.421591 7.184308

S 0.629248 29.355236 6.637686

S 47.862137 30.032402 8.435964

O 1.196405 28.530905 7.672747

O 1.609551 29.943905 5.757616

O 47.367344 28.676115 8.377781

O 46.759117 30.928120 8.686295

C 48.385292 28.317732 5.591381

C 0.225883 30.244596 9.788339

F 48.356445 27.035761 5.999635

F 47.113102 28.768034 5.599692

F 48.784260 28.299154 4.302077

F 1.426205 30.673386 9.359060

F 0.397007 29.091217 10.456214

F 48.672295 31.156937 10.669875

N 26.059862 38.294796 17.175550

S 26.463717 38.946049 15.897983

S 24.723133 38.743145 17.723640

O 25.345049 39.240383 15.038447

O 27.499754 38.195053 15.217394

O 24.414131 40.118988 17.375034

O 24.641481 38.498680 19.142021

C 27.222532 40.522839 16.515009

C 23.560951 37.577049 16.862150

F 27.172174 40.659252 17.852716

F 28.515259 40.595692 16.143913

F 26.592304 41.592125 15.994791

F 22.687237 38.241016 16.084263

F 22.849560 36.865170 17.753952

F 24.198513 36.687691 16.073019

N 43.231991 43.097046 34.957375

S 43.831905 42.308407 33.824039

S 43.049622 42.324543 36.233963

O 44.775539 41.312946 34.273849

O 44.379993 43.187984 32.819290

O 42.713238 40.940388 35.997814

O 42.105503 42.984104 37.106701

C 42.347031 41.477230 33.082336

C 44.731533 42.448238 37.023582

F 41.189251 41.763741 33.712074

F 42.184219 41.852238 31.795475

F 42.501534 40.140987 33.089668

F 45.286469 41.234722 37.209373

F 44.642059 43.012951 38.248837

F 45.611603 43.188965 36.321484

N 36.297523 45.495239 7.000960

S 35.008713 45.479103 7.802802

S 37.339432 46.450603 7.518246

O 35.192062 45.799839 9.200158

O 34.274830 44.257763 7.594030

O 36.742451 47.702259 7.936768

O 38.365204 46.624172 6.525233

C 34.053345 46.857391 7.005360

C 38.095802 45.567600 8.974274

F 33.702297 47.802979 7.905965

F 34.725132 47.501308 6.025548

F 32.913654 46.401222 6.454827

F 37.563847 44.352127 9.211892

F 37.954990 46.295612 10.098808

F 39.418842 45.396473 8.782614

N 8.496591 25.343094 38.792526

S 8.566997 24.379416 39.956661

S 7.191642 26.049105 38.586197

O 9.680775 23.485655 39.787983

O 7.330009 23.670959 40.223618

O 7.395525 27.242579 37.787289

O 6.499320 26.308237 39.824654

C 8.966451 25.495150 41.387753

C 6.216230 24.851721 37.560425

F 8.986985 26.805479 41.067993

F 8.077579 25.327446 42.382725

F 10.179970 25.208639 41.902596

F 6.894374 23.733541 37.265114

F 5.866647 25.429144 36.393356

F 5.080940 24.484535 38.181366

N 33.459297 8.002285 34.455734

S 34.611702 8.595354 35.238506

S 33.677361 6.595150 33.961426

O 35.257576 7.623365 36.088264

O 34.165310 9.766826 35.954788

O 35.038536 6.365842 33.547306

O 32.708305 6.285169 32.935165

C 35.806152 9.151265 33.917912

C 33.295506 5.534176 35.447769

F 37.023094 8.578241 34.060192

F 35.385185 8.891644 32.663322

F 35.994392 10.486527 33.985874

F 32.942993 6.235788 36.542969

F 34.328613 4.736245 35.794418

F 32.272182 4.694686 35.182770

N 0.823844 5.988390 22.843204

S 48.640026 6.671422 22.040384

S 1.451138 6.831302 23.905645

O 47.964325 7.718217 22.789423

O 47.753109 5.697478 21.459049

O 1.535723 8.218880 23.507168

O 2.712573 6.291526 24.326612

C 0.714324 7.491354 20.682142

C 0.225885 6.704180 25.305933

F 2.045186 7.316318 20.785797

F 0.344205 7.029806 19.476936

F 0.490884 8.821728 20.695833

F 48.051353 5.938519 25.021379

F 48.650780 7.939702 25.597824

F 0.790597 6.220631 26.426067

N 41.015190 3.082691 10.844912

S 40.452435 3.709008 12.106346

S 41.949535 3.966184 10.072894

O 41.286549 4.749446 12.660790

O 40.100407 2.700349 13.068556

O 41.523186 5.340560 10.071427

O 42.113811 3.456232 8.734209

C 38.885422 4.446800 11.431136

C 43.545883 3.789681 11.011636

F 38.832619 5.778640 11.615462

F 38.733364 4.214559 10.102230

F 37.802937 3.914846 12.031051

F 43.466187 2.911077 12.032029

F 43.948761 4.964086 11.525010

F 44.526672 3.359424 10.195615

N 17.072388 41.575504 4.413553

S 17.538336 42.573406 3.378004

S 17.891830 41.532967 5.657875

O 18.227236 43.697941 3.956405

O 16.440205 42.952324 2.517490

O 19.302877 41.631729 5.404121

O 17.561804 40.331669 6.402023

C 18.723497 41.557903 2.373256

C 17.309029 43.020287 6.610796

F 18.860888 40.292557 2.826982

F 18.309376 41.465492 1.095199

F 19.948751 42.117233 2.352721

F 16.389355 43.752213 5.955632

F 18.337244 43.834839 6.913930

F 16.745785 42.644791 7.774444

N 39.274609 38.269375 42.262936

S 38.874664 37.212311 43.250568

S 39.153843 37.874805 40.821087

O 37.755508 36.415356 42.794399

O 38.645355 37.786800 44.553562

O 39.389019 36.464737 40.602535

O 39.993820 38.712830 40.000668

C 40.415764 36.172359 43.327820

C 37.379032 38.288441 40.441677

F 41.411221 36.616306 42.524513

F 40.917892 36.158180 44.577522

F 40.161526 34.895279 42.982147

F 36.701870 37.203506 40.011909

F 37.297871 39.207623 39.456978

F 36.696980 38.786171 41.493362

N 40.393764 6.840103 44.042145

S 41.800411 7.013183 44.596588

S 40.311134 6.315972 42.642830

O 42.716660 6.012836 44.109615

O 41.766186 7.127103 46.031593

O 41.332504 6.897797 41.796497

O 38.994453 6.540879 42.097191

C 42.301563 8.673094 43.916489

C 40.578091 4.487381 42.813957

F 43.422672 8.587043 43.174297

F 41.362820 9.234383 43.113670

F 42.555805 9.551208 44.902657

F 40.716946 4.070325 44.086147

F 41.669865 4.114818 42.127014

F 39.537163 3.809727 42.288361

N 23.525261 0.598450 9.578099

S 22.748842 0.491859 10.858603

S 22.800669 1.264365 8.433030

O 21.952377 1.688270 11.091821

O 23.601044 0.157923 11.961624

O 21.363708 1.088841 8.460899

O 23.322844 0.819933 7.175018

C 21.660000 47.921299 10.506574

C 23.237768 3.054331 8.660381

F 21.885883 47.394722 9.282787

F 21.920109 46.947357 11.403756

F 20.354563 48.218567 10.583336

F 23.984852 3.284131 9.758513

F 22.137192 3.819016 8.739588

F 23.948671 3.495349 7.602830

N 2.693015 38.504059 17.975437

S 2.531180 39.003254 19.387463

S 1.510787 37.777996 17.410725

O 1.179783 39.445732 19.659306

O 3.521260 40.014355 19.660284

O 0.813087 36.985447 18.395426

O 1.891662 37.035320 16.232899

C 2.945302 37.516422 20.428879

C 0.401899 39.164597 16.853348

F 3.213724 36.407536 19.709177

F 4.039034 37.758442 21.185249

F 1.941533 37.218174 21.274235

F 48.071892 39.080505 17.430773

F 0.196060 39.107395 15.522486

F 0.873714 40.400608 17.109056

N 7.628744 2.231956 29.864210

S 8.244304 1.293215 28.856041

S 7.128570 3.513926 29.264297

O 7.667369 1.389533 27.551582

O 8.175854 48.832172 29.347902

O 8.023309 4.054680 28.304531

O 6.792676 4.453156 30.342381

C 10.025958 1.825657 28.820839

C 5.529776 2.964370 28.485924

F 10.300246 2.903254 29.595789

F 10.829266 0.828244 29.231537

F 10.405855 2.143460 27.572115

F 5.279444 1.642798 28.635534

F 5.531242 3.226436 27.165329

F 4.492759 3.614156 29.033522

N 46.026703 48.267948 38.282085

S 44.827362 48.751987 39.008633

S 45.935764 46.837345 37.805866

O 44.660637 48.081669 40.280823

O 43.642693 48.727051 38.191631

O 45.020489 46.041862 38.602821

O 47.258801 46.267746 37.722260

C 45.295265 1.622263 39.324970

C 45.258598 47.003094 36.083862

F 46.487762 1.967449 38.802792

F 44.376572 2.454418 38.795456

F 45.349049 1.896062 40.641651

F 44.121838 46.295612 35.956741

F 46.127911 46.528835 35.171036

F 44.977467 48.275772 35.733303

N 35.702988 48.655666 1.696580

S 36.908688 0.316336 2.393791

S 34.396084 48.755901 2.442195

O 36.650043 1.584616 3.041132

O 38.042999 0.386742 1.498075

O 34.520275 0.374519 3.797015

O 33.655361 47.526737 2.394769

C 37.258270 47.909565 3.671849

C 33.486187 1.096177 1.400290

F 37.209373 48.454231 4.899058

F 36.387978 46.876949 3.683095

F 38.486946 47.393749 3.503658

F 33.149315 2.176219 2.127326

F 32.336231 0.569112 0.933364

F 34.183403 1.527411 0.332471

N 18.633535 1.437448 22.021807

S 17.586252 0.818953 22.913610

S 20.038713 0.868828 22.149416

O 17.914810 0.872738 24.315367

O 17.185820 48.403381 22.458418

O 20.388298 0.521200 23.505213

O 20.990658 1.768942 21.544123

C 16.181561 2.001184 22.648613

C 20.022579 48.221992 21.115822

F 16.500830 3.030866 21.840414

F 15.113742 1.376330 22.115679

F 15.771350 2.543891 23.818129

F 18.848175 47.975571 20.512484

F 20.329626 47.149769 21.868771

F 20.948608 48.296799 20.135523

N 39.151398 14.808163 12.419749

S 39.268742 15.603647 13.689006

S 40.216282 15.098097 11.388600

O 40.638718 15.858379 14.070369

O 38.489391 14.994933 14.739713

O 40.598137 16.491053 11.378821

O 39.784073 14.634593 10.092940

C 38.433651 17.195599 13.207413

C 41.651287 14.053746 11.943533

F 39.261894 18.250216 13.301286

F 37.929569 17.183865 11.944510

F 37.382946 17.438107 14.016588

F 41.393135 13.344312 13.060733

F 42.739151 14.813051 12.183597

F 41.993046 13.170253 10.983768

N 16.700802 36.008080 16.243166

S 16.676357 37.062698 17.303650

S 15.528842 35.068359 16.265656

O 15.338649 37.494907 17.612654

O 17.585762 38.137852 16.975090

O 15.065338 34.796516 17.606787

O 15.826600 33.884178 15.507329

C 17.368677 36.166004 18.778257

C 14.224382 36.009548 15.330827

F 17.727551 34.890388 18.525482

F 18.458010 36.798187 19.252518

F 16.459761 36.121510 19.780071

F 13.104248 36.149868 16.075464

F 13.857197 35.347538 14.215093

F 14.609168 37.240177 14.939196

N 3.312487 11.931310 26.860237

S 4.457556 11.213564 26.219742

S 2.895432 11.412068 28.204790

O 5.443235 10.754950 27.190262

O 5.044759 12.016384 25.180281

O 3.074868 9.981465 28.331423

O 1.567503 11.858459 28.543127

C 3.631268 9.765359 25.413988

C 4.108951 12.254979 29.331766

F 2.299917 9.715977 25.630583

F 3.822439 9.788339 24.081659

F 4.172512 8.624690 25.864780

F 5.025691 13.007930 28.681982

F 4.839898 11.332862 30.001598

F 3.507570 13.031398 30.241661

N 12.475487 14.649749 1.601239

S 12.425615 14.248341 3.055800

S 13.505169 13.951560 0.775440

O 12.692081 12.836804 3.248926

O 11.179828 14.674685 3.642514

O 14.733845 13.703673 1.499542

O 13.719809 14.654149 48.425385

C 13.791680 15.242330 3.832218

C 12.686704 12.326364 0.383808

F 14.660017 14.436089 4.472225

F 14.515782 15.977188 2.960459

F 13.296397 16.090620 4.753358

F 13.413739 11.292769 0.854646

F 12.581095 12.154262 47.941345

F 11.438471 12.200220 0.876159

N 46.948822 18.020908 30.919319

S 48.206345 17.672302 30.166367

S 46.284370 19.266695 30.471458

O 48.100735 18.001350 28.766079

O 48.542236 16.292059 30.404964

O 47.201595 20.330605 30.151699

O 45.264465 19.630459 31.429270

C 0.596982 18.743544 30.979944

C 45.358829 18.728876 28.956760

F 0.086052 19.515072 31.963179

F 1.560166 17.990595 31.548080

F 1.188584 19.557119 30.088140

F 45.476170 17.420015 28.663893

F 45.657562 19.450533 27.870361

F 44.047523 18.930803 29.213936

N 7.310451 29.617790 14.903013

S 5.863225 29.949772 14.659039

S 7.950947 30.361452 16.046127

O 5.166991 30.331139 15.867181

O 5.205616 28.885866 13.937870

O 7.530469 31.745607 16.089642

O 9.388395 30.225529 16.000656

C 5.967855 31.424379 13.530594

C 7.340765 29.453512 17.557405

F 7.229290 31.806232 13.233814

F 5.354740 31.174540 12.353744

F 5.343494 32.493176 14.066948

F 6.665555 30.271000 18.391027

F 8.384626 28.971916 18.261950

F 6.526700 28.407694 17.290449

N 43.410938 44.903145 13.512014

S 44.782379 45.165211 12.968327

S 42.438461 46.051640 13.443075

O 44.738869 45.932827 11.737206

O 45.531906 43.944359 12.842672

O 43.099491 47.341919 13.317910

O 41.465984 45.974388 14.495737

C 45.583736 46.195385 14.298210

C 41.555946 45.745571 11.844280

F 45.981724 47.384457 13.793148

F 44.779938 46.452560 15.342561

F 46.689201 45.587646 14.771982

F 42.018959 44.686550 11.162715

F 41.719738 46.827080 11.056129

F 40.231930 45.584713 12.023717

N 46.047729 43.388447 3.473344

S 46.773296 43.621666 2.188931

S 45.641918 44.672375 4.173978

O 48.145718 44.027477 2.404059

O 46.024750 44.479733 1.305438

O 46.572834 45.752415 3.934404

O 45.398430 44.437687 5.579646

C 46.814854 41.905041 1.482919

C 44.014275 45.117786 3.392182

F 48.087044 41.493851 1.298593

F 46.226189 40.976078 2.274004

F 46.196362 41.840015 0.288468

F 44.084682 46.323971 2.792757

F 43.048157 45.208729 4.330924

F 43.584019 44.234783 2.475442

N 48.250347 44.478268 24.702599

S 47.911522 43.258881 25.504930

S 48.153538 44.305187 23.207457

O 48.815548 42.163193 25.231617

O 46.524433 42.896587 25.375362

O 48.557884 42.977261 22.786489

O 48.882042 45.359806 22.546427

C 48.246925 43.882267 27.223511

C 46.353306 44.551609 22.818270

F 48.695763 45.156410 27.255291

F 47.144882 43.827995 27.994551

F 0.297759 43.129318 27.820980

F 45.843845 43.453964 22.228622

F 46.193428 45.575912 21.951889

F 45.593025 44.838608 23.888533

N 11.968957 9.547297 12.023229

S 12.257915 8.070734 11.912731

S 11.071774 10.112497 10.943187

O 11.101110 7.301651 11.525010

O 12.860763 7.581807 13.131140

O 11.128980 9.360038 9.711088

O 11.331883 11.520122 10.773040

C 13.546727 7.992506 10.575024

C 9.382528 9.943329 11.699069

F 13.863553 9.193314 10.055782

F 14.696687 7.456641 11.040483

F 13.137006 7.204843 9.563921

F 9.409908 9.421642 12.943879

F 8.574820 9.170334 10.948565

F 8.775768 11.144625 11.808100

N 40.411854 43.874931 20.143833

S 41.257702 42.707859 20.584356

S 39.004230 43.904266 20.625917

O 40.847000 42.188129 21.874149

O 42.650658 43.029575 20.502707

O 38.399918 42.589050 20.616627

O 38.225857 44.875278 19.919415

C 40.896870 41.439579 19.269630

C 39.174377 44.513474 22.373835

F 40.019245 41.855171 18.329422

F 42.021896 41.074841 18.612022

F 40.395721 40.314556 19.809895

F 38.633133 43.631935 23.234837

F 40.442169 44.752068 22.753731

F 38.497211 45.674675 22.530779

N 32.188576 2.858273 9.865589

S 31.602839 3.818038 10.887938

S 33.193321 3.508058 8.896534

O 32.586563 4.756781 11.351930

O 30.998524 3.051400 11.950868

O 32.799248 4.848699 8.535215

O 33.438274 2.647056 7.761243

C 30.231396 4.714243 10.016667

C 34.740780 3.544728 9.933550

F 30.429901 6.043639 9.979020

F 30.012844 4.281054 8.764034

F 29.080462 4.523073 10.709479

F 34.541786 2.943835 11.130445

F 35.179836 4.794917 10.149167

F 35.741127 2.866096 9.341458

N 2.902277 45.181835 44.871857

S 3.860087 44.690464 43.819683

S 1.859393 46.154804 44.352127

O 3.261639 44.571167 42.519623

O 4.450712 43.471077 44.318390

O 2.385480 47.021671 43.328308

O 1.254100 46.866192 45.451237

C 5.171880 45.999813 43.795235

C 0.586714 45.012177 43.628510

F 5.328826 46.514164 42.562160

F 4.903948 47.005051 44.645969

F 6.364865 45.489861 44.166332

F 0.408744 45.244907 42.317696

F 48.292885 45.181835 44.232826

F 0.912340 43.710163 43.780079

N 13.062690 41.052837 15.709746

S 13.793148 42.188618 16.380066

S 12.566427 39.999687 16.656309

O 13.235769 42.510334 17.681593

O 13.836174 43.346401 15.529331

O 13.422540 39.845676 17.800892

O 12.249113 38.763680 15.976701

C 15.526886 41.529545 16.538967

C 10.958832 40.738457 17.206842

F 15.917540 41.534431 17.828270

F 15.675520 40.282776 16.059330

F 16.400112 42.313293 15.871091

F 10.968611 41.027905 18.525482

F 9.951640 39.883324 16.979004

F 10.660097 41.895264 16.582972

N 20.854734 11.188628 35.303047

S 21.969002 10.457192 36.014435

S 19.442711 10.784774 35.669250

O 21.755341 10.324693 37.434772

O 23.247547 11.048795 35.699566

O 19.367905 9.629437 36.534164

O 18.639402 10.642007 34.477734

C 21.931355 8.779680 35.220905

C 18.837906 12.297028 36.563015

F 21.777830 7.799868 36.132267

F 20.958387 8.631046 34.303677

F 23.095491 8.536683 34.584324

F 18.382227 12.001716 37.792179

F 17.810669 12.856850 35.889271

F 19.776648 13.256794 36.698448

N 43.891556 22.329830 25.036535

S 43.876400 21.218008 24.013699

S 43.361557 23.647491 24.543207

O 42.748932 21.316772 23.117004

O 43.972717 19.923326 24.646862

O 43.659805 23.889021 23.152206

O 43.853416 24.702599 25.406166

C 45.462482 21.539722 23.096958

C 41.522209 23.472944 24.769093

F 45.239529 21.848724 21.806189

F 46.175827 22.554247 23.630379

F 46.256989 20.451368 23.108204

F 40.865582 23.625002 23.602020

F 41.061153 24.424887 25.603203

F 41.157959 22.283382 25.292736

N 2.899343 41.761784 24.750511

S 3.899201 42.627190 25.482437

S 3.509036 40.790287 23.782436

O 5.126899 41.929485 25.791929

O 3.299775 43.249104 26.638266

O 4.679530 41.317837 23.128738

O 2.475931 40.372738 22.867651

C 4.230205 43.957069 24.236160

C 3.987697 39.352837 24.857588

F 5.541510 44.040188 23.935471

F 3.539350 43.802082 23.090113

F 3.866443 45.145164 24.743666

F 3.717319 39.541073 26.166937

F 5.305847 39.084904 24.758823

F 3.329111 38.246880 24.472313

N 40.569778 24.672285 8.890178

S 41.869350 25.434525 8.910712

S 40.023647 24.373060 7.524602

O 42.751865 25.059515 7.831649

O 42.499577 25.318159 10.203927

O 40.273487 25.432079 6.573637

O 38.632156 23.993652 7.611142

C 41.332504 27.201019 8.684829

C 40.988789 22.860807 7.035184

F 40.006535 27.343788 8.525437

F 41.686001 27.950546 9.748735

F 41.937309 27.758400 7.612609

F 41.692844 23.066645 5.905761

F 40.161034 21.820856 6.792187

F 41.854683 22.446196 7.983216

N 46.717072 38.416050 6.009903

S 46.876461 37.622032 7.256181

S 45.950920 39.704865 6.183472

O 47.172752 38.449783 8.389027

O 47.860672 36.571815 7.070877

O 44.908527 39.605122 7.175508

O 45.463947 40.129745 4.892703

C 45.221928 36.816765 7.466908

C 47.239246 40.912518 6.772631

F 44.364349 37.097900 6.468029

F 45.369583 35.477104 7.493800

F 44.645969 37.174175 8.626645

F 48.458630 40.363453 6.960379

F 46.881840 41.491405 7.935791

F 47.391304 41.894283 5.869092

N 46.321037 32.637901 46.719025

S 45.308468 31.526077 46.636395

S 45.718189 34.020100 46.760098

O 44.112553 31.797430 47.392277

O 45.920605 30.266600 46.992336

O 44.548187 34.114464 45.925495

O 46.718536 35.014576 46.444736

C 44.890923 31.432203 44.827850

C 45.249310 34.215183 48.554462

F 45.572979 32.296139 44.056324

F 45.186237 30.197660 44.370705

F 43.578152 31.606262 44.597569

F 45.571022 33.139538 0.414609

F 43.928223 34.423466 48.693317

F 45.878559 35.265888 0.226862

N 30.337494 45.095295 20.731037

S 29.508760 46.297081 21.114355

S 29.820208 43.782036 21.226320

O 28.102602 46.014481 21.171560

O 29.826563 47.425526 20.262642

O 29.272608 43.873955 22.562561

O 30.860157 42.780220 21.108976

C 30.126276 46.702888 22.816313

C 28.470768 43.369381 20.016712

F 31.105110 45.879536 23.242170

F 30.628405 47.953079 22.860317

F 29.133265 46.660843 23.727186

F 28.283018 44.311546 19.072104

F 27.292450 43.170387 20.634716

F 28.781235 42.228710 19.368881

N 3.887955 8.031131 3.796037

S 2.620654 8.801682 3.648381

S 3.849819 6.922243 4.836476

O 1.463362 7.947525 3.632246

O 2.681770 9.704731 2.515534

O 2.973660 7.224889 5.943898

O 5.184593 6.577548 5.243752

C 2.624077 9.912037 5.134233

C 3.141362 5.485772 3.890889

F 3.690917 9.759491 5.935097

F 2.661724 11.192539 4.688819

F 1.506387 9.794206 5.869580

F 2.028073 5.021291 4.486892

F 4.022410 4.465868 3.830751

F 2.812803 5.783529 2.619676

N 4.836476 20.607338 44.197136

S 5.972255 19.839720 43.578152

S 5.222240 21.607685 45.258598

O 7.124170 20.683121 43.327335

O 5.535154 19.187490 42.368057

O 6.325262 21.158358 46.069241

O 4.059569 21.991003 46.027191

C 6.358509 18.521080 44.838120

C 5.749304 23.087667 44.268028

F 7.637545 18.605667 45.248329

F 5.569379 18.562151 45.931850

F 6.195207 17.292406 44.299809

F 5.695033 22.894543 42.929832

F 7.010250 23.456808 44.561386

F 4.954307 24.133974 44.550140

N 35.804688 43.702831 32.704884

O 36.687691 42.897564 33.077446

O 35.917141 44.930038 32.963524

O 34.809227 43.299461 32.050697

N 19.836298 40.739433 37.576073

O 19.411907 40.819618 38.753410

O 21.040039 40.937943 37.307163

O 19.058903 40.208950 36.742939

N 37.284184 10.549600 8.200300

O 37.150215 9.552186 8.963517

O 38.131496 11.403267 8.477034

O 36.579147 10.621472 7.153995

N 13.360936 2.086745 15.543021

O 12.461308 2.300406 14.698153

O 13.068068 2.070121 16.766319

O 14.547564 1.908286 15.209083

N 20.820021 14.661485 17.504110

O 20.730059 13.566774 16.892952

O 21.210186 14.694732 18.697584

O 20.586313 15.736636 16.902731

N 6.906108 6.450916 39.644238

O 6.261701 5.433946 39.975731

O 8.120116 6.460694 39.547428

O 6.213296 7.533403 39.525429

N 38.624332 22.109814 15.586535

O 39.810471 22.481398 15.485328

O 38.133450 21.500120 14.582767

O 37.914902 22.367966 16.574657

N 32.996284 17.658123 39.758160

O 33.352715 18.703941 39.184155

O 31.909885 17.610209 40.369808

O 33.703762 16.618662 39.610989

N 1.621285 32.606121 30.804419

O 2.830404 32.936146 30.892426

O 0.756860 33.222656 31.471806

O 1.276102 31.640488 30.079338

N 35.743080 34.091972 26.624084

O 35.512794 35.143654 27.273382

O 36.907219 33.624557 26.573727

O 34.809719 33.531174 25.995813

N 47.569763 9.726733 14.750469

O 46.389977 10.134500 14.896170

O 47.976547 8.739099 15.410032

O 48.341290 10.311002 13.947649

N 12.578650 26.033949 33.209946

O 11.790988 26.947754 32.861828

O 12.216355 24.830698 33.159588

O 13.718343 26.326328 33.651936

N 27.593628 9.111174 24.915770

O 27.885519 9.594723 26.032972

O 27.492422 7.864896 24.756868

O 27.457218 9.894436 23.946226

N 39.969372 36.681824 0.483058

O 39.401241 36.301437 1.543544

O 39.652061 36.153294 48.282131

O 40.858734 37.555538 0.535375

N 44.970131 24.133486 1.037994

O 44.852299 25.208639 0.409722

O 44.649391 23.036821 0.521686

O 45.496216 24.196558 2.176708

N 16.991714 0.645874 42.129459

O 16.308681 1.546479 41.581371

O 17.946102 0.952921 42.883873

O 16.740406 48.332489 41.875214

N 26.160091 40.237305 45.949940

O 26.961935 41.030350 45.396965

O 24.979818 40.173256 45.529953

O 26.536077 39.516140 46.901394

N 13.445520 8.399294 45.763168

O 13.406405 8.833462 46.941486

O 14.019032 9.036856 44.856209

O 12.794268 7.355433 45.514797

N 39.379726 26.129290 12.677415

O 39.490227 27.372633 12.557138

O 40.327759 25.457993 13.151185

O 38.281105 25.565554 12.448596

N 37.754044 26.103376 28.015085

O 37.219643 25.886782 26.899351

O 37.745731 25.205704 28.896132

O 38.280617 27.217646 28.253193

N 8.696074 16.537012 12.814314

O 9.040768 16.351707 11.621329

O 9.563921 16.568792 13.722741

O 7.484021 16.716448 13.094959

N 32.137238 35.074715 3.770613

O 32.095676 35.747971 2.712084

O 32.933701 35.400829 4.686863

O 31.387709 34.072903 3.913380

N 34.628323 27.960327 20.387810

O 33.998589 28.867287 20.978922

O 34.019119 26.992249 19.877857

O 35.883892 27.962769 20.449413

H 4.193536 13.002552 -0.351048

H 2.767821 11.971401 -0.029823

H 32.103500 6.751606 48.915783

C 4.346081 19.593300 49.279541

O 3.008374 19.719933 -0.062092

H 5.052582 20.045071 -0.334915

H 21.798855 49.489292 33.107269

H 23.552639 49.377327 32.807068

H 40.382519 32.546959 -0.235172

H 41.228367 34.116909 -0.194592

H 42.165638 32.590473 -0.139835

H -0.869804 28.603266 35.931808

H -0.266468 27.830271 37.426460

H -0.155968 22.385569 31.716269

H 14.428754 6.545768 49.792915

H 14.881013 5.048671 48.965160

C 12.374278 5.157701 48.905022

H 11.391533 5.162102 -0.480125

O 13.217191 6.039728 -0.706501

H -0.674232 39.770870 26.606482

H -0.033249 38.393070 28.573441

H -0.925053 38.699627 24.478180

C -0.343227 40.769749 29.420753

O 49.762112 40.270065 28.884399

C 1.375843 32.445263 -0.112942

C 1.298593 31.390644 49.840343

H 1.464339 33.403561 49.318169

H -0.173080 29.994267 2.646078

H -0.345184 31.374512 3.766701

C 6.270990 9.055435 49.513245

C 6.057817 9.395729 -0.825310

C 6.381488 33.106293 49.119663

H 5.547377 32.540112 -0.231262

O 7.384768 33.416763 -0.732903

O 50.112675 36.121025 29.831940

C -0.061604 35.967010 30.424522

H 27.658657 -0.410702 15.556710

H 25.971365 -0.562267 16.112133

H 19.628502 -0.080673 15.317136

H 21.897617 -0.889847 14.384749

O 21.809610 -0.138367 16.338018

C 21.711336 48.939735 14.938705

C 18.953295 -0.192638 5.096585

O 19.415331 49.921017 5.646141

H 21.857527 48.977383 42.000870

O 49.449688 15.137700 25.514217

C -0.755882 15.598759 25.254597

C 36.216362 32.976238 49.209625

H 37.055851 34.933907 49.011608

H 35.278599 32.972816 -0.263042

C 37.150215 33.945293 -0.365231

C 34.949551 -0.665920 17.259647

O 35.961632 49.145084 17.636122

H 35.210148 48.988632 15.274111

H 36.722404 -0.760769 19.300432

H 33.460766 38.888355 -0.241528

H 39.119617 -0.421455 19.277943

O 40.716946 -0.234196 17.277248

C 41.550568 48.987164 16.184984

C 40.102852 49.771893 17.903076

H 41.922642 -0.857578 15.786017

C 11.751874 49.939106 22.818270

O 11.794410 -0.101208 23.650425

H 10.544221 -0.035690 21.425802

H 12.985439 49.665306 25.139210

H 49.767002 13.345289 15.154813

H 39.031124 48.980804 41.100266

O 24.939238 50.104851 21.893707

C 24.416574 -0.099255 22.037451

H 35.944519 49.088860 33.322399

H 36.109287 48.968090 30.774595

C 43.678383 37.737907 50.205082

C 43.976627 38.279152 -0.071873

C 5.024224 -1.147514 25.764061

C 4.802740 49.214024 25.495640

C 20.930519 45.443901 49.249229

O 20.853268 45.065960 -1.002304

C 49.388084 20.717344 25.696587

O -0.914295 20.623960 25.564089

C 49.957195 4.726467 7.483532

O -0.244465 4.523562 7.977838

O 4.403775 25.304468 -0.096809

C 3.838085 24.988132 50.054493

H -0.072361 45.980743 19.215359

H -0.204372 47.945744 17.850273

H 39.052635 48.978363 25.901449

C 30.154144 -0.393097 36.250099

H 29.511696 48.994984 35.500084

C 30.476837 49.470222 37.362900

H 40.178146 -0.066006 44.492935

C 11.189117 45.860466 49.295189

H 10.707034 44.952038 -0.005375

O 12.542959 45.963142 -0.005375

H 28.713762 7.724084 -0.138367

H 11.146091 -0.131031 2.760488

H -0.187260 45.468838 32.458462

C 2.414326 48.973961 35.658985

O 2.829915 -1.264370 35.792953

H 32.251156 44.897770 -0.226376

C 46.965935 -0.285534 9.482269

O 46.981583 49.800739 10.238153

C 29.474047 -0.917717 31.108532

H 26.934067 -0.676189 30.625473

H 27.202488 -0.436615 32.369968

O 28.721586 49.170021 31.114887

C 49.693661 29.801140 41.421494

O -0.152058 28.907867 40.859226

O 40.193302 19.819185 49.924931

H 41.901619 19.012453 49.016987

C 40.821575 19.165977 -0.056717

C 3.349646 49.451157 49.545029

H 3.255772 46.874016 49.300564

O 4.219449 -0.234688 -0.132500

H -0.150589 33.152252 5.897449

C 3.454276 49.184200 12.129326

H 3.173143 -0.010265 13.151185

H 2.730663 -0.157921 11.429181

O 4.797851 -0.042538 11.836946

C 47.404503 -0.456657 33.252972

O 47.556072 49.338211 34.332523

H -0.198505 5.925807 15.881360

H 49.617390 24.491381 27.571629

C -0.152058 15.172903 10.174103

H 49.092285 14.475202 9.396218

O 49.807583 15.657430 10.972522

H -0.044003 8.365069 34.406353

C 49.009167 27.069986 13.098380

H -0.376476 26.255434 12.542469

O -0.794998 28.072289 13.495879

H 45.168633 49.263409 5.141567

C 43.277462 49.458000 9.648993

O 43.607979 -0.808685 9.525784

H 49.082993 31.040571 38.452721

H 49.156818 32.307384 37.192261

C 49.034100 48.029842 5.332738

O -0.676678 47.840137 6.476340

H -0.149612 47.358055 4.504004

C 0.034225 49.454086 4.864833

H -0.964169 0.737792 4.421865

C 0.141300 -0.862957 5.332738

H 49.101570 46.026703 7.066965

O 42.232624 41.298283 49.676552

C 42.534290 41.722668 -0.535378

C 49.638905 5.236419 32.620789

O -0.094852 6.084709 33.377647

H 40.816685 7.956814 -0.173080

O 42.816891 9.366393 -0.798908

C 42.546028 8.977207 49.427197

O -0.978344 43.709187 47.652878

C 49.317188 43.826527 47.517933

C 49.005745 33.835285 17.554960

O -1.014526 34.132061 16.755074

H -0.060139 34.076813 18.618378

C 5.686233 5.543954 49.848164

H 3.393160 4.818385 49.139709

C 2.964859 6.922243 48.918224

C 3.716342 5.680366 -0.359364

O 2.978549 7.840939 -1.053638

O 5.138144 5.805042 -0.326603

H 3.149674 9.559031 49.001343

C -0.110500 38.421917 2.019273

H 49.527428 38.987606 1.431092

O 49.133842 38.401382 3.390227

O 48.904533 37.060253 0.096808

H 49.599789 36.465717 1.975758

H -0.668854 40.224594 3.886488

C -0.129078 37.009892 1.505409

C 0.072850 35.776817 -0.491863

H -0.713348 35.095253 48.761276

H -0.059650 35.915672 47.318939

O 0.011733 37.060253 48.989609

O 29.306833 49.069794 26.277925

C 29.960041 -0.786198 27.096878

H 27.398060 -0.670807 26.198229

H 37.140926 -0.239574 10.352561

C 38.466412 -0.023468 12.080433

C 37.802937 49.397373 10.832200

O 37.570206 48.984718 13.176121

H 39.364571 49.487335 12.236402

H 39.080990 -0.651741 8.778703

H 40.220684 -0.368652 10.127166

H 39.062412 -0.714325 14.420442

H 37.581940 -0.100719 15.207128

H -0.087517 2.852895 27.867920

H -0.962212 1.319617 29.438354

C 49.276611 37.112568 12.938013

H 49.235538 34.583344 12.670081

O -0.733391 36.356686 12.517535

H 9.877323 32.302006 49.121620

H 11.456072 31.473272 49.085926

C -0.150589 3.549128 46.440826

H 49.544540 3.178521 45.778328

O 49.278076 4.130953 47.615719

H 1.430603 3.700207 49.301056

O 37.059765 16.549234 -0.779350

C 37.665058 16.600084 49.394440

C 25.537199 41.939266 -0.068939

O 24.258162 41.891350 49.419865

H 26.285257 42.396412 49.494183

H 9.593256 48.996944 25.857445

H 19.252518 10.905050 -0.593559

H 49.013077 9.386929 28.454142

C -0.269886 33.103848 26.166937

O 49.921993 33.392315 25.697567

H 49.517651 41.904064 38.274750

C 49.677040 43.676918 39.430088

C -0.023468 42.508865 38.930401

H 15.906294 -0.305580 27.580429

C 2.465664 -1.153870 8.408584

O 2.649501 48.974937 7.745108

C 23.832306 -0.130543 48.700653

C 26.084309 0.364740 49.152908

O 24.900612 0.774948 -0.408257

H 26.232941 -0.727524 0.191171

C 27.279249 1.088351 -0.308514

O 28.204300 1.277567 49.640858

H 22.913122 49.247269 48.341290

H 23.700785 48.513393 49.765537

O 24.900612 49.667747 48.484543

H 29.978621 0.824823 -0.268421

H 29.518538 2.552202 -0.287491

H 22.641277 13.299820 49.045341

H 23.630867 14.733845 49.313278

C 48.906979 18.473656 36.480385

H 49.394440 17.909922 33.968273

O -0.192638 17.263069 35.797352

O -1.040928 19.359104 36.190449

C 13.301286 49.393459 42.449219

O 12.807958 -0.671299 41.818501

O 13.634734 -1.340641 43.863197

C 13.858665 48.953426 43.776657

C 42.469261 -0.253265 0.865892

H 40.584934 -0.443459 2.794713

H 42.788536 -0.285046 3.493391

O 41.157471 49.153397 0.938742

H 42.814449 48.681580 -0.177479

O 43.326355 49.413021 1.670178

C -0.202415 24.570097 44.505646

O 49.285896 23.414761 43.928711

C 49.819805 25.448214 44.971107

C 32.182217 17.807735 49.061481

H 31.963667 19.934082 49.028229

H 31.986156 16.952112 -0.506039

C 32.175861 19.105350 -0.557865

C 34.167267 18.201323 -0.453728

O 33.495968 17.729019 49.585613

C 48.398983 49.960129 41.728050

H 49.239937 47.582474 41.432247

C 49.297634 49.133842 39.921459

C -0.325626 -0.786682 40.727703

C -0.493816 1.067328 41.728050

O 47.594208 -0.053291 41.452782

O 49.109882 1.492206 40.585915

H 49.105480 0.838514 42.553360

H 26.520433 -0.072361 36.691113

H 19.825052 39.610989 49.338699

H 22.010561 38.808659 49.364616

H -0.465950 15.367496 17.568649

C -0.458126 14.007788 19.304344

C 49.069302 15.011556 18.386137

C 49.749397 15.356251 20.351627

O -0.161346 14.425332 20.633251

H 47.168839 10.379453 -0.246418

H 49.260960 10.602404 0.877137

C 29.946350 -0.220016 4.997822

H 29.311724 49.168556 4.251718

O 29.917992 49.455067 6.182983

H 31.617996 49.009655 3.624423

O 32.146038 49.239937 5.594314

C 31.390644 -0.337360 4.599346

H 31.238098 -0.732414 7.122214

H 3.433741 -0.720680 19.838253

H 4.494226 -0.220997 21.967535

C 30.392252 33.769279 49.726421

O 31.316328 33.981960 -0.230286

O 29.344479 34.898701 -0.875179

C 29.051613 34.205891 49.217934

H 30.602491 30.214773 49.629612

H 33.020241 30.428923 49.705891

C -0.618984 14.404307 36.547855

O 49.619347 14.702065 36.899395

O 49.181267 12.607985 37.733997

C -0.889362 13.011841 37.050964

C 35.800774 10.584803 49.784115

O 35.448746 11.322595 -0.268909

O 37.521313 10.409277 -0.687920

C 37.134567 9.951152 49.492714

H 33.921337 -0.064537 34.119839

H 32.114258 -0.097298 32.615406

H 44.899727 28.614021 -0.429768

C 19.186024 -0.334427 39.321545

C 20.467014 -0.414124 37.570206

C 19.706244 49.768959 37.654789

O 19.348347 49.921501 39.014988

O 18.134829 49.169044 34.795052

H 19.500893 49.807095 33.382050

C 17.602386 -1.032616 34.886971

C 19.119040 -0.018093 33.814262

C 37.470955 4.111885 49.856964

O 37.929077 3.778435 -0.339317

O 35.635517 3.699229 -0.423901

C 35.967499 4.051746 49.804649

H 49.269760 1.887751 19.683752

H 11.010658 -0.092407 46.533234

H 10.846378 -0.089474 48.287018

C 42.822758 5.387987 -0.264999

C 43.250568 5.151345 50.045689

C 42.963570 3.310531 48.940228

O 42.136791 4.209670 -0.649784

H 43.936047 3.184877 -0.475727

C 45.832108 49.715664 27.641054

O 46.430557 -0.353008 28.187189

O 46.766941 -0.261578 25.863314

C 46.483852 49.941063 26.319971

C 48.570595 27.320318 49.449196

H 49.346035 28.337778 47.688080

C 48.410717 28.223858 -0.635117

H 49.528408 26.765873 0.525600

C 47.414280 26.338551 -0.917717

O 47.492020 26.398199 49.381237

C 12.517045 41.876682 49.012096

C 11.678534 40.885624 -0.639027

O 12.492109 43.083847 -0.627296

C 44.728111 49.047302 19.172823

H 43.646114 -0.052315 19.189936

C 45.530441 -0.805264 20.009378

C 45.874161 -1.207161 17.951969

O 45.204815 48.919201 17.837559

H 9.615258 49.542099 36.787922

H 8.908757 48.922134 34.423950

H -0.224415 22.468685 11.052707

H -0.075294 19.077971 20.170725

H -0.946075 21.256144 21.356863

H 49.708820 19.779581 8.783102

H -0.360828 27.775023 31.760763

H 6.084220 49.698063 2.102391

H 6.920287 49.723003 4.292299

C 25.651119 49.471691 45.521152

O 25.878471 -0.030315 44.260696

H 25.731791 -0.191662 46.306370

C 24.530985 -0.240551 43.913555

O 23.795147 49.826164 44.145309

H 14.778826 -0.627296 2.971704

C -0.284069 44.285629 11.645776

O 49.656994 43.793278 10.820954

H 48.977383 44.322792 12.680836

O 49.066372 45.838467 9.960930

C -0.699657 45.606228 11.056618

H -0.444927 44.023075 9.139042

O 35.398876 -0.180416 25.974789

C 34.349636 49.640369 25.843267

C 36.206097 49.552853 26.780540

H 26.003633 4.676596 -0.475727

H 16.272991 46.809967 49.165134

H 15.434479 49.413998 48.254745

H 8.207634 48.981293 45.590580

C 49.877499 24.407774 7.157906

C 48.901600 25.523018 8.766479

C -0.818466 24.390661 8.280973

H -0.417057 26.474962 8.416407

O -0.399456 24.235672 6.935932

C -0.445412 17.693815 14.759758

C -1.327927 19.366926 13.780436

C 49.027256 19.242739 13.443564

O 49.375374 17.892319 13.721764

C 40.834778 49.323547 36.523899

C 41.444962 -0.872250 36.121510

C 39.904346 -1.266815 37.487087

O 39.894569 49.035568 37.556026

H 7.795957 14.287943 49.018456

H 48.929466 31.054752 32.744976

H 34.583344 41.417091 49.542583

H 32.889210 41.463047 49.049744

O 33.851418 43.259861 48.976406

C 33.887600 41.852238 -0.087517

C 34.737354 43.610909 -0.950966

H 32.812935 49.035568 43.788391

H 48.925556 13.095448 40.753616

C 49.282963 15.196860 41.226410

C -0.317314 13.887511 41.435181

O -0.634140 16.170315 41.113464

O 46.188049 4.353904 -0.088985

C 46.738094 3.608778 49.891190

C 46.744450 5.621205 49.067837

H 46.096619 6.391756 -0.266956

H 47.735504 5.713613 -0.312424

C -0.371586 8.222791 46.737118

O 49.686817 7.720173 46.104931

C 49.500050 8.354313 44.866966

O -0.711388 8.150430 44.422531

H 21.564169 5.797219 49.877987

H -0.233707 43.882267 5.315136

C 49.352882 34.747623 43.974674

H 49.521072 36.583549 42.762623

O -0.081650 33.619175 43.310219

C -0.109520 35.954296 43.280396

C 48.843418 23.134607 -0.545155

O 48.032288 23.106737 49.507874

C 49.268784 21.715248 48.138870

H 49.664326 23.848930 48.504101

O -0.554932 20.896782 48.850754

C -0.049381 23.134607 48.347645

C 47.399124 21.880505 49.225758

O 48.337868 20.896782 -0.042046

H 46.645195 22.011049 -0.472794

H 34.640060 -0.509464 36.553722

C 36.546387 -0.319759 37.604427

C 35.403767 49.129440 36.807480

O 36.323929 49.005253 38.931381

H 37.515934 48.966629 37.255337

C -0.161835 0.585247 23.384937

C 1.158270 -0.737301 24.396528

C 49.782650 0.063072 22.442284

O 49.244339 0.375497 24.692329

H 0.506040 -0.742191 21.791033

O 1.933710 -0.455193 23.256838

C 0.889849 48.955872 22.442284

O 0.351539 49.268295 24.692329

C 8.979163 25.006222 49.551872

C 9.001654 23.889999 -0.352516

C 7.553937 25.346027 -0.890827

O 8.302975 26.092621 48.931423

C 12.759554 -0.198994 18.503481

C 13.432808 -0.919674 20.401009

O 12.950725 49.166107 19.827986

H 11.877039 49.198380 18.086424

H 13.621534 48.994003 17.884008

H 35.190102 21.330462 -0.685478

H 35.775352 23.593710 -0.099743

H 37.502735 20.911451 -0.026890

C 49.617878 18.768967 1.792899

O -0.648808 18.495169 2.034918

C -0.504574 18.044865 3.362358

O 49.441864 17.115412 3.443520

H 2.580073 -0.908428 31.489897

H 16.610350 3.819017 48.917732

H 18.996319 4.112373 49.166599

H 27.480198 -0.503109 18.337732

H 33.916447 35.294247 49.414974

C -0.193127 3.962273 38.277195

C -0.540264 5.926296 37.494907

H 49.503471 3.422007 38.795948

O 49.288834 4.885858 37.371212

C 34.442532 49.898525 22.293650

C 33.623577 -0.229797 22.541536

C 35.695656 -0.705521 22.655457

O 35.770462 49.592945 22.701904

H 14.683975 49.668728 38.826260

H 49.004276 23.527216 16.251966

H 48.942177 22.310762 13.770167

C 49.073215 42.303516 33.868530

H -0.141788 41.251343 33.959961

O -0.607250 42.938637 32.870628

C -0.006355 43.088245 35.138763

H 49.814426 43.432941 35.617916

H 21.905441 -0.364738 3.437164

H 29.538584 49.400307 11.421358

H 3.947116 -0.015648 43.239815

O 41.355000 13.495879 -0.488926

C 41.520741 12.265248 49.062946

C 41.287525 14.313367 49.561653

H 41.384823 11.448249 -0.548576

H 15.835400 33.599621 48.975430

C 17.957836 34.087082 49.043880

C 16.731117 33.597668 -0.565689

O 18.281507 35.357803 -0.406300

C -0.287491 30.547733 45.359318

O 49.098637 29.248650 45.122677

O 49.382706 31.520210 44.705620

C -0.124187 13.763324 4.238028

O 50.165966 13.498324 4.326524

O 49.436485 14.132464 6.437226

C -0.537819 14.365194 5.550311

C 37.734486 39.584099 -0.709435

O 37.566292 38.978317 49.458488

O 38.884445 40.958965 49.577297

C 37.950100 41.011772 -0.382832

N 37.989216 47.251957 -0.661030

S 38.507969 47.504242 -2.041763

O 38.974895 48.868843 -2.084789

O 38.042999 49.279541 1.498075

O 23.601044 49.050720 11.961624

O 26.153248 49.218426 14.286965

O 7.192131 -1.195919 12.472064

O -0.077251 42.163193 25.231617

O -0.334915 42.977261 22.786489

O 29.945862 12.704306 -0.691345

O 31.384289 -0.113922 10.763749

O 11.147558 8.008640 49.120152

O 9.472490 35.102097 49.373417

O 49.890701 26.001190 40.967278

O -0.928474 7.718217 22.789423

O -0.268421 11.223342 23.214790

F 34.725132 -1.391491 6.025548

O 46.658398 16.324327 -1.345531

F 48.788170 17.867386 -1.918064

O -1.032127 36.571815 7.070877

O 7.667369 50.282333 27.551582

N 48.213676 14.423864 -1.333309

O 49.337231 13.813684 0.740240

F 45.815975 13.904134 -0.080185

F -0.104630 17.867386 46.974735

C 46.702404 14.357370 49.721535

C 49.035568 16.663645 46.404156

O 36.927753 45.638496 49.706371

F 40.204548 44.491959 -0.238110

C 39.570408 45.477150 49.304478

N -0.305580 30.421591 7.184308

F -0.220505 31.156937 10.669875

C 49.118683 30.244596 9.788339

N 49.716644 5.988390 22.843204

F -0.841446 5.938519 25.021379

F -0.242020 7.939702 25.597824

C 49.118683 6.704180 25.305933

F -0.820908 39.080505 17.430773

C 49.294697 39.164597 16.853348

O 8.175854 -0.060627 29.347902

N 35.702988 -0.237133 1.696580

O 34.520275 49.267319 3.797015

O 17.185820 -0.489418 22.458418

O 13.719809 14.654149 -0.467415

F 12.581095 12.154262 -0.951454

C 12.686704 12.326364 49.276608

F 49.190559 43.129318 27.820980

C -0.645874 43.882267 27.223511

F -0.599915 45.181835 44.232826

C 49.479511 45.012177 43.628510

F 45.571022 33.139538 49.307407

F 45.878559 35.265888 49.119659

C 45.249310 34.215183 -0.338337

O 39.652061 36.153294 -0.610668

O 16.740406 -0.560310 41.875214

H 4.625259 18.528416 49.368526

C 4.437510 20.212282 50.654896

C 2.845561 19.152287 -1.345531

H 12.760531 4.123619 48.900131

H 12.240802 5.467193 49.956215

C 14.494269 6.069063 -0.090942

H -1.099110 40.983902 28.646292

H -0.106098 41.709957 29.940485

H -0.771526 40.070091 30.155123

C 49.591476 39.081482 28.118250

H 0.438080 32.487309 -0.700634

O 2.503800 32.214489 -0.938255

H 0.966122 30.425501 49.420353

O 0.417056 31.866369 50.841179

H 2.316541 31.242498 50.246639

H 5.419767 8.438408 49.854031

O 6.386867 10.229352 50.295044

H 7.174041 8.423251 49.584145

O 6.038750 8.161675 -1.518608

H 5.114187 9.950173 -0.944118

H 6.868460 10.049426 -1.189564

C 6.945222 32.354321 50.302868

H 5.941453 34.035744 49.524006

C 6.794144 34.196114 -1.753296

C 51.088573 36.506786 30.779974

C -0.920162 35.130455 29.515606

H -0.005375 35.495682 31.421448

H -0.533421 36.954643 30.561911

C 23.035843 -0.684010 16.772675

C 20.304203 49.380749 14.653172

H 22.430550 49.697090 14.575433

H 17.861029 -0.183834 4.928394

H 19.454445 -0.430744 4.141220

H 19.170378 -0.995457 5.816776

C 19.097038 50.998611 4.771448

C 49.589523 14.693264 26.848503

H -1.499542 14.803761 25.441366

C -0.859047 15.983056 23.803459

H -1.025280 16.450472 25.904871

H 36.612396 31.947044 49.174419

O 35.976788 33.379604 50.546844

O 36.764942 34.019119 -1.732273

H 38.208256 33.646069 -0.252285

H 33.958008 -0.281132 17.553982

C 34.968132 -0.857094 15.767927

H 35.075695 -1.644264 17.758354

C 36.206585 49.070282 19.026144

H 41.014702 49.522537 15.383630

H 42.421837 49.589523 16.494965

H 39.193447 50.077961 17.355965

C 39.769402 49.366081 19.310699

H 40.777084 50.640717 17.938768

C 10.532976 49.834476 21.943579

H 12.649056 50.029556 22.182175

H 11.700536 50.869045 23.409382

C 12.923344 -0.124676 24.500181

C 24.369638 50.955097 22.865696

C 24.940218 -0.976879 20.938829

H 23.311110 -0.105118 21.989048

H 24.701620 -0.541733 23.010908

H 43.390892 36.680355 50.085785

H 42.816402 38.243462 50.676895

O 44.821495 37.875786 51.047997

H 43.164516 38.921604 -0.443947

O 44.272430 37.248978 -1.025280

H 44.876743 38.903023 -0.010757

H 4.269319 -1.728848 25.221840

H 5.997680 -1.435493 25.344072

O 5.066272 -1.472164 27.161417

H 3.731498 49.437466 25.339672

O 5.573779 49.570942 24.364748

H 5.125921 49.784115 26.378645

C 19.568365 45.918159 49.660904

H 21.670755 46.247700 49.410576

H 21.212141 44.587299 49.888744

C 22.083900 44.638634 -1.543056

C 49.786072 22.133282 25.399321

H 49.912704 20.060715 24.978354

H 49.712730 20.416656 26.710136

C -1.317173 19.270609 25.508841

H 50.140541 3.951516 6.727160

H 50.716991 4.642371 8.277551

H 50.071114 5.708723 6.994115

C -0.650276 5.602137 8.802171

C 3.531038 25.027245 -1.171959

H 3.522237 23.929604 50.107296

H 2.947747 25.610048 50.260818

C 4.863367 25.256065 51.122311

O 29.499470 -1.513721 36.819702

H 31.074797 -0.708946 35.735748

H 29.524893 49.699532 37.869431

O 31.142759 50.661743 36.910152

H 31.116356 48.974449 38.111446

C 11.157825 45.826733 50.795219

H 10.593603 46.725872 48.956360

C 12.686704 46.092220 -1.406643

H 1.365087 49.055122 35.323090

H 3.054822 49.538677 34.961773

H 2.490599 49.429153 36.656399

C 2.906188 -1.943489 34.545696

H 46.429092 -0.066494 8.546950

H 46.439846 -1.104000 10.002000

H 47.981926 -0.629742 9.223138

C 47.752617 49.696598 11.421847

H 29.105394 -1.641331 30.359983

H 29.486269 -1.407623 32.098122

H 30.502752 -0.641964 30.842554

C 27.353565 48.911869 31.373531

H 49.994843 30.568266 40.689075

H 50.574223 29.220291 41.721695

H 49.312786 30.318426 42.317207

C -1.432556 29.545919 40.815708

C 40.486660 19.170378 51.146271

C 40.653385 20.001066 -1.293701

H 40.377140 18.170033 -0.235664

H 3.866443 50.405544 49.723976

H 3.132562 48.994003 50.525822

H 2.393303 49.664326 49.039478

C 3.692873 -1.510300 -0.468884

H 3.367736 50.277443 12.046697

C 4.990488 -1.439404 11.650665

H 46.343529 -0.686943 33.058868

H 47.865074 -0.079697 32.324986

H 47.894409 -1.393932 33.544861

C 46.911663 50.580090 34.083168

C -1.147022 14.514317 11.068841

H -0.651741 16.006035 9.647527

C 50.823086 16.245123 10.184370

H 49.560184 26.640707 13.952538

H 49.738155 27.554026 12.430994

C -1.795834 27.638609 14.395996

H 43.216835 49.964039 8.673582

H 42.322098 49.604187 10.182903

H 44.087124 49.910259 10.236197

C 42.921032 -1.486828 8.463832

H 50.094093 47.823025 5.569379

C 48.927025 49.454086 4.864833

C -0.770061 46.472118 6.825924

H -0.964169 49.630592 4.421865

O 1.071239 49.698551 3.929025

H 0.122719 50.121967 5.740015

O -0.676678 -1.052662 6.476340

H -0.149612 -1.534744 4.504004

H 1.201295 -1.069775 5.569379

C 43.368889 41.343750 50.511150

C 41.256721 41.727558 -1.321083

H 43.298485 41.083641 -1.009148

H 42.924454 42.757732 -0.536842

C 50.696945 6.126268 32.044830

H 49.086903 4.720600 31.813566

H 50.109253 4.462446 33.253948

C -1.116222 5.379675 34.044544

C 44.202999 9.540452 -1.008659

C 41.089020 8.625668 49.556763

H 43.130783 8.082957 49.711754

H 42.812004 9.776115 50.144455

C -1.633018 44.956928 47.561935

H 49.583164 44.325233 46.570393

C 49.877991 42.429661 47.507664

H 49.750378 44.428398 48.336399

H 49.311317 32.778221 17.479176

H 49.832520 34.461601 17.189241

C -2.116570 33.286705 17.029850

H 5.350339 6.269524 50.608448

H 6.777031 5.625606 49.747936

H 5.444213 4.527962 50.205570

H 1.922465 6.664088 49.183712

H 3.431786 7.358855 49.816872

H 3.459654 5.447147 -1.403225

C 2.480332 9.091616 -0.629742

H -1.091286 38.903999 1.853037

C 49.200333 39.711224 3.915824

C 48.965649 35.776817 -0.491863

H -1.061951 36.495052 1.804144

H -0.713348 35.095253 -0.131523

H -0.059650 35.915672 -1.573860

H 1.048263 35.301579 -0.318295

C -0.129078 37.009892 50.398209

C 27.914856 49.146065 26.511633

H 30.105253 -0.380875 28.113359

H 29.365992 -1.713203 27.192707

C 31.305573 -1.055595 26.460295

H 38.824795 -1.064396 11.978248

H 37.159992 50.230019 11.156360

O 38.715763 49.971863 9.904215

C 38.295773 48.971027 14.394040

H 50.226593 36.703335 12.551760

H 49.346035 37.162926 14.037612

H 49.159267 38.131985 12.547359

C -0.600891 35.040001 13.019175

H -0.806732 2.689593 46.672577

C -0.933365 4.603746 45.711346

C 50.024666 3.199056 48.369648

C 37.621056 17.482597 -1.680935

C 37.159508 15.431545 50.192860

H 38.765144 16.521366 49.329411

H 37.433796 17.547626 49.915638

H 25.455059 42.553848 -0.975899

H 25.894114 40.935986 -0.347137

C 24.260607 41.004925 50.524841

H -0.439545 32.032608 25.991901

H -0.373543 33.298443 27.247955

H -1.043373 33.672958 25.624228

C 50.256420 34.758869 25.871626

O 50.531197 44.146778 38.394047

H 50.279888 43.350311 40.295979

H 49.001831 44.471424 39.788471

O -0.469860 41.759830 40.047604

H -0.868824 42.863338 38.309956

H 1.501987 -1.610043 8.127939

C 3.585798 -2.066208 8.017442

H 2.451974 -1.026260 9.502805

C 1.668222 49.931770 8.091759

H 23.975073 -1.068306 48.135448

H 23.700785 -0.379406 49.765537

H 26.232941 -0.727524 49.083969

H 26.011946 0.619963 50.224640

C 23.832306 -0.130543 -0.192146

H 26.946289 2.061322 -0.707966

H 27.726130 0.524621 -1.147026

C 29.502893 1.630085 49.209137

C 26.084309 49.257538 49.152908

H 48.934849 18.281017 37.562870

H 49.890213 18.918579 36.209030

C -0.453236 17.821915 34.519295

C -1.089329 19.162066 34.781849

H 12.475975 50.117565 42.571941

H 14.017566 49.867722 41.765205

C 12.543448 -1.410557 42.982639

H 13.348712 49.446266 44.621525

H 14.932350 49.139221 43.919910

H 42.814449 -0.211220 -0.177479

H 42.497620 -1.323036 1.169516

C 41.092442 49.323059 2.350277

C 42.510822 49.446754 2.831382

H -0.874691 24.257185 45.321671

H -0.842422 25.026758 43.739986

C 50.556622 23.501791 44.527649

O 51.006435 24.834608 44.495869

H 49.871143 25.519108 46.071686

H 49.791451 26.482784 44.598545

H 31.456160 17.741241 49.883858

H 31.459583 19.175268 -1.386112

O 33.496944 19.280874 -1.071732

H 34.288521 17.377968 -1.184181

H 35.177391 18.526459 -0.173080

H 47.762398 50.787399 42.070786

O 49.109882 50.385006 40.585915

H 49.105480 49.731312 42.553360

H 48.893780 49.232117 38.901066

H 50.376205 48.940716 39.820744

O -1.298592 -0.053291 41.452782

H -0.830200 -1.566525 40.139523

H 0.347138 -1.310326 41.432247

H -1.130402 1.894598 42.070786

C 48.567173 -0.786682 40.727703

C 49.297634 0.241042 39.921459

H -0.078228 12.984462 19.155220

H -1.552349 13.950583 19.211447

H 49.985069 14.613568 17.907476

O 49.409107 16.112133 19.212915

H 50.724323 14.839454 20.209839

H 49.867722 16.029993 21.212141

H 29.479425 -1.202274 5.187037

C 31.212185 49.162685 6.645998

H 31.728981 -1.386112 4.535296

H 30.433323 32.716125 50.047157

H 30.727169 34.361858 50.580090

C 30.394697 34.060680 -1.286858

H 28.493746 34.875233 49.888256

H 28.379826 33.352222 49.003784

H -0.730461 14.490359 35.452656

H -1.262413 15.183659 36.981049

C 50.094585 13.382449 36.997181

H -1.090797 12.302406 36.227608

H -1.736671 12.922856 37.746220

H 34.982800 9.882702 49.996799

H 35.853577 11.270768 50.649521

C 36.755161 11.587105 -0.722633

H 37.130657 8.852041 49.474136

H 37.906097 10.247931 50.225132

H 18.225769 -0.715302 38.916222

O 20.262154 -1.083954 38.809147

H 19.145441 -0.458614 40.412834

H 20.131121 -1.071243 36.751740

H 21.551456 -0.286022 37.435261

H 18.783146 49.743534 37.052917

H 20.265577 50.663696 37.349209

O 17.589672 -1.733738 33.660736

H 16.572214 -0.968079 35.256111

H 18.178831 -1.626175 35.622314

C 18.392982 -0.878605 32.852539

H 19.974665 -0.552490 34.272385

H 37.904629 3.400983 50.580578

H 37.880676 5.097075 50.118053

C 36.820679 3.014241 -0.747574

H 35.546532 3.301731 50.494038

H 35.453148 4.992444 50.044224

H 43.682781 5.551289 -0.942654

H 42.150482 6.241655 -0.409233

O 43.167942 3.744699 50.265709

H 42.603718 5.647607 50.784462

H 44.273895 5.487728 50.266689

H 42.483444 2.325341 48.953918

H 44.754513 49.514225 27.526157

H 45.936253 50.539509 28.360758

C 47.215290 -0.784241 27.093945

H 45.859978 50.447590 25.571424

H 47.418682 50.526798 26.408955

H 48.542236 27.826359 50.424610

H 49.528408 26.765873 49.418400

O 47.427971 27.627853 -1.475098

H 48.068954 29.241318 -0.393097

H 49.346035 28.337778 -1.204720

H 46.485809 25.833490 -1.217430

H 48.252304 25.739126 -1.326462

H 13.564329 41.552525 49.134823

H 12.142037 42.105503 50.015377

O 11.163204 41.591148 -1.754276

H 10.827311 40.473949 -0.080185

H 12.261335 40.017780 -0.994968

C 12.194353 42.536247 -1.885796

H 44.833721 50.101917 19.462757

H 44.930527 -1.668224 20.350161

H 45.981236 -0.374519 20.914873

O 46.575279 -1.271214 19.170378

H 45.165211 -2.056919 17.865917

H 46.583103 -1.303970 17.117857

C 24.276741 50.079430 45.455147

H 26.464207 50.187969 45.705479

H 24.477203 -0.495773 42.848183

H 24.094860 -1.093731 44.477291

H -1.114758 43.560551 11.646753

C 49.322079 44.490978 9.647038

H -1.740093 45.579334 10.685032

H -0.639027 46.467716 11.736716

H 33.505745 49.162197 25.333803

H 34.648861 50.503819 25.206684

O 33.943336 50.076004 27.117413

H 36.750763 50.259842 26.125866

C 35.236061 50.247131 27.694347

H 36.980068 48.954403 27.281204

H 50.328293 23.437740 7.440506

H 50.350784 24.727043 6.219164

O 50.182590 25.355316 8.161186

H 49.020409 25.605160 9.853366

H -0.628273 23.460232 8.846663

H -1.899975 24.577923 8.308353

H -0.483063 16.627462 15.021335

O -1.711250 18.104515 14.307011

H -0.142281 18.235548 15.683832

H -1.987984 19.601124 12.932145

H -1.520565 20.143343 14.541697

H 49.294697 19.462757 12.400681

H 49.651123 19.912571 14.064991

H 40.322872 49.840340 35.699078

H 41.576481 50.025154 36.930199

H 40.983902 -1.275616 35.205257

O 41.193161 -1.750362 37.203506

H 42.522556 -0.826290 35.923985

H 39.183666 -1.621284 36.718983

H 39.559650 -1.674091 38.445873

C 34.335457 41.611195 -1.502476

O 34.406841 42.889740 -2.114616

H 34.657173 44.688995 -1.150936

H 35.787083 43.415829 -0.644894

H 49.891678 15.198326 40.302334

H 49.961597 15.491684 42.039497

O -1.700493 14.122196 41.189739

H -0.220016 13.489524 42.455574

C -1.585594 15.340606 40.494972

H 45.903004 3.111049 50.407497

H 47.379566 2.809869 49.491249

C 47.454372 4.594945 50.761482

O 46.864727 5.845623 50.451012

C -1.407135 8.316176 45.655609

H -0.620449 7.565183 47.580029

H -0.152546 9.214337 47.174217

H 50.197258 7.929924 44.131130

H 49.728374 9.440222 44.941280

H 50.446125 34.659126 43.941425

H 49.071747 34.709000 45.042492

C -1.282948 34.207355 42.886318

O -1.026260 35.465370 42.310360

H -0.645386 36.621685 43.973694

H 48.249859 23.511568 -1.398823

C 49.268784 21.715248 -0.753929

H 49.664326 23.848930 -0.388699

H 50.272068 21.518209 48.542728

H 49.283455 21.408201 47.081810

C -1.493675 21.880505 49.225758

H -0.642941 23.511568 47.493977

O -0.860512 23.106737 49.507874

H 46.854458 21.538746 50.116096

H 36.640266 -1.413979 37.593674

O 34.779404 50.129787 37.612740

H 35.723526 49.578766 35.855534

C 35.779751 50.257885 38.601845

H -1.121113 0.044981 23.281284

H -0.399944 1.653554 23.276884

H 0.541243 -1.650623 24.251318

H 1.821746 -0.939228 25.248241

H 49.398838 -0.742191 21.791033

O 50.826511 -0.455193 23.256838

H 50.216816 0.819443 21.774408

C 50.051071 -0.737301 24.396528

C -0.161835 49.478046 23.384937

H 1.324017 49.712242 21.774408

H 8.436942 24.727043 50.472038

H 9.968264 25.359230 49.878475

O 8.384626 24.404352 -1.523991

H 8.437430 23.004063 -0.009777

H 10.002488 23.525261 -0.625828

H 6.702225 24.837053 -0.392120

H 7.128081 26.023682 -1.644756

C 12.628033 -1.690224 18.584154

O 12.671547 -2.017319 19.962440

H 13.362891 -0.852203 21.494741

H 14.506004 -1.066353 20.167301

H 49.800251 18.681450 0.713346

H 49.832520 19.821629 2.055453

C 50.395763 17.797956 2.637278

H -1.435982 17.556425 3.684072

H -0.331493 18.899023 4.053702

H -0.730946 3.198078 37.698792

C -1.095688 4.759714 39.177311

H -1.450161 5.715079 36.894016

O -0.877136 6.123823 38.846794

H -0.092407 6.845970 37.093987

H 34.448891 50.218285 21.239031

H 34.107616 50.772728 22.868628

H 33.250526 -0.696720 21.611595

O 34.479691 -1.139202 23.215279

H 32.744976 -0.060139 23.177143

H 35.804195 -1.076134 21.613062

H 36.531231 -1.126492 23.232880

H 50.108276 42.296673 33.509171

C -1.491230 43.614334 33.731140

O -0.765659 44.225494 34.772560

H -0.553467 42.512779 35.907848

O 40.556580 12.144972 50.078449

H 42.551403 12.166485 49.472179

C 40.734547 13.433786 50.649517

H 40.682720 15.199794 49.323544

H 42.299603 14.688864 49.796337

H 17.794535 34.200516 50.127831

H 18.840351 33.440231 48.927513

H 16.806900 32.583630 -0.982746

O 16.530655 34.501202 -1.636440

C 17.049898 35.651649 -1.017460

H -0.320740 30.760416 46.447670

H -1.312771 30.619604 44.981377

C 50.460793 29.555696 45.356873

C 50.658318 30.892916 44.724686

H -0.628273 12.812358 4.010187

H -0.308514 14.432666 3.383382

C 50.465195 14.249806 5.482839

H -1.451138 13.934937 5.984968

H -0.714325 15.454036 5.468660

H 36.857349 39.359680 -1.328907

H 38.623356 39.162151 -1.215477

C 38.425831 39.794827 50.235886

H 37.017715 41.490917 -0.034225

H 38.360802 41.625374 -1.193474

O 39.518581 46.559143 -2.438286

C 27.016203 50.125877 13.619089

C 26.672977 48.983742 15.583114

C 6.438693 -1.660400 11.359263

C 8.568462 -1.483898 12.307784

C 28.737720 11.952333 -0.790596

C 29.929728 13.246526 -1.994339

C 30.024580 -0.408745 11.058574

C 31.861483 -1.433537 10.884515

C 10.102230 7.723595 50.036404

C 11.971401 8.695584 50.030045

C 8.814882 35.316734 50.600624

C 10.800420 35.372963 49.808071

C 50.874912 25.063425 41.342285

C 50.645607 26.691557 39.977684

C -1.140182 11.393000 22.101013

C -0.875179 12.121503 24.113440

C 34.053345 -2.035408 7.005360

C 49.035568 16.663645 -2.488644

F 46.184139 15.476037 50.272556

F 46.765961 13.437209 50.700855

F 49.200821 16.873394 45.088451

F 50.201168 16.225565 46.910686

F 39.524937 45.147121 50.607956

F 40.340469 46.586037 49.215004

F 50.319004 30.673386 9.359060

F 49.289806 29.091217 10.456214

F 49.683395 6.220631 26.426067

F 49.088860 39.107395 15.522486

F 49.766514 40.400608 17.109056

F 11.438471 12.200220 49.768959

F 13.413739 11.292769 49.747444

F -1.747917 43.827995 27.994551

F -0.197037 45.156410 27.255291

F 49.805138 43.710163 43.780079

F 49.301544 45.244907 42.317696

F 43.928223 34.423466 -0.199482

H 3.410273 20.405899 51.010834

O 5.092674 19.287720 51.505142

H 4.960175 21.187206 50.652451

H 1.802678 19.320969 -1.642796

H 3.046510 18.067356 -1.350418

H 3.492413 19.630947 -2.097988

C 15.517108 6.799033 -0.920162

C 49.240917 39.355770 26.680801

H 50.565910 38.569576 28.134382

C 2.721862 33.341957 -1.760143

C 0.296779 30.928606 51.886505

C 6.713959 9.869012 51.623955

C 5.442257 8.193455 -2.796669

H 6.934955 31.261078 50.145924

O 6.163915 32.737640 51.423492

H 7.998862 32.644257 50.451012

H 6.466073 35.177391 -1.368999

H 5.927763 33.692028 -2.215332

H 7.546114 34.356480 -2.536560

H 50.790821 37.404945 31.345663

H 52.008736 36.731205 30.222593

H 51.302238 35.690277 31.492342

H -0.750504 35.465370 28.476143

O -2.284271 35.282509 29.877411

H -0.599426 34.073883 29.571833

H 23.228479 -1.679466 16.339973

H 22.954679 -0.800865 17.862984

H 23.889511 -0.020535 16.555590

O 19.990311 49.152908 13.290529

H 20.201527 50.446121 14.918660

H 18.049755 50.943363 4.423820

H 19.727757 50.965855 3.864976

C 19.303856 52.322140 5.464259

H 48.988140 13.790703 27.049942

H 49.311810 15.472126 27.579451

H 50.649029 14.440488 26.997625

H -0.360828 15.199305 23.206478

O -2.235867 16.088177 23.487123

H -0.316338 16.924730 23.599577

C 34.962265 32.595852 51.141380

C 37.695862 34.781361 -2.477886

O 33.684204 -1.290283 15.340116

H 35.760685 -1.574348 15.493150

H 36.852459 49.917103 19.290165

H 35.277622 49.141174 19.611879

O 39.153843 50.440258 20.001556

H 40.711567 49.061970 19.802561

O 10.518797 50.907673 21.015102

H 9.639215 49.846699 22.592873

H 13.869421 -0.223442 23.938402

H 12.811380 -1.006214 25.147036

H 23.267595 50.974655 22.805069

H 24.655172 50.662231 23.891466

H 24.740246 51.971088 22.672081

O 24.434666 -2.282803 21.150047

H 24.625835 -0.570580 19.960485

H 26.044704 -0.971012 20.963766

C 44.780426 36.894505 52.070835

C 43.204121 37.020161 -1.935665

C 3.840529 -2.040295 27.621986

C 5.455947 50.949722 24.086548

O 19.557608 46.145023 51.059238

H 18.841330 45.136364 49.370483

H 19.306301 46.834412 49.099617

H 21.878550 44.361416 -2.586918

H 22.490688 43.758080 -1.017460

H 22.845160 45.437546 -1.540611

H 49.335770 22.398281 24.427332

O 51.194183 22.233023 25.351894

H 49.358257 22.808491 26.158136

H -2.415302 19.247139 25.492704

H -0.965633 18.697094 26.381577

H -0.949009 18.776791 24.595034

H -0.486973 6.566792 8.289774

C -2.136127 5.486750 9.061791

H -0.042538 5.629028 9.723800

H 3.247460 23.960894 -1.216942

H 2.613809 25.638895 -1.127468

H 4.072281 25.277578 -2.096035

O 4.246829 25.109386 52.389122

H 5.717035 24.562275 51.001057

H 5.254509 26.281836 50.985413

C 29.249630 -2.517979 35.855045

C 30.525730 51.825882 37.445042

O 9.809362 45.847755 51.230366

H 11.690757 44.929058 51.160446

H 11.717649 46.706802 51.157024

H 13.741322 45.895672 -1.642796

H 12.072610 45.369095 -1.963535

H 12.428550 47.105770 -1.753296

C 1.603195 -2.183064 33.830883

H 3.326177 -2.931614 34.770115

H 3.613667 -1.446247 33.858753

C 47.742840 51.041149 12.097056

H 47.329208 48.947559 12.115635

H 48.793056 49.391506 11.204762

C 26.595240 50.201172 31.251299

H -1.972824 29.110771 39.962040

C -2.184528 29.301943 42.087410

H -1.337219 30.625959 40.601559

H 41.568657 19.180645 51.369709

H 40.132675 18.124561 51.159470

H 39.968399 19.723356 51.942730

O 41.084129 19.238338 -2.406013

H 39.591923 20.288555 -1.398335

H 41.238632 20.931496 -1.181252

H 2.898365 -1.436958 -1.231609

H 3.255772 -2.018784 0.407766

C 4.837943 -2.346855 -0.966122

H 4.360260 -1.825169 10.828777

H 4.708376 -2.007538 12.557138

H 46.042839 50.466171 33.417252

C 46.420780 51.161915 35.377361

H 47.611809 51.277790 33.592777

H -1.933220 14.028811 10.464526

O -1.689247 15.512707 11.924953

H -0.662498 13.711007 11.656532

H 50.458347 17.110523 9.603523

H 51.279747 15.522975 9.483247

H 51.605862 16.595194 10.873758

C -2.590340 28.871199 14.720156

H -2.450508 26.878328 13.934447

H -1.357265 27.198574 15.308336

H 42.647232 -2.473000 8.862798

C 43.742920 -1.686802 7.230756

H 41.979359 -0.976879 8.198833

H 47.928631 49.630592 4.421865

O 49.964039 49.698551 3.929025

H 49.015518 50.121967 5.740015

H -1.220852 45.868290 6.026038

H -1.420826 46.400246 7.707461

C 1.029192 51.035774 3.474811

C -0.770061 -2.420681 6.825924

H 44.034813 40.492531 50.317535

H 43.020287 41.303661 51.551590

H 43.948269 42.268314 50.375717

H 40.910561 40.699833 -1.540611

H 40.500351 42.190575 -0.667385

O 41.435181 42.481487 -2.509178

H 51.159470 6.672400 32.888721

H 50.203125 6.876284 31.396511

O 51.664532 5.386031 31.326595

H -1.697067 6.118934 34.612682

H -0.712368 4.633571 34.750069

H -1.801212 4.870701 33.346355

H 44.777985 8.625668 -0.777885

H 44.605389 10.374563 -0.410210

H 44.342346 9.775626 -2.073055

O 40.921806 7.990062 50.809399

H 40.457325 9.527250 49.466801

H -2.713551 44.755001 47.601540

H -1.415447 45.467369 46.610973

H -1.376820 45.633118 48.397026

H 49.748425 41.971535 48.505081

H 49.261940 41.828770 46.813389

O 51.241608 42.444817 47.104790

H -2.354187 33.258350 18.108915

C -3.291954 33.831375 16.271034

H -1.912197 32.250668 16.703247

H 1.476563 9.006543 -0.184814

H 2.422638 9.742379 -1.513721

H 49.509827 39.621258 4.964086

H 49.942043 40.338516 3.392671

H 48.179451 35.095253 -0.131523

H 48.833149 35.915672 -1.573860

H 49.941063 35.301579 -0.318295

H -1.061951 36.495052 50.696945

C -0.110500 38.421917 50.912071

H 0.706988 36.465717 50.868557

H 27.532024 49.970398 25.896070

H 27.670879 49.355812 27.568205

H 31.165249 -1.642796 25.533289

H 31.675688 -0.067474 26.152760

O 32.324497 -1.620308 27.283159

C 39.604145 49.016499 9.355637

H 38.791058 49.938126 14.577876

C -1.722492 34.179977 12.520468

H -0.588181 35.022404 14.126107

O -1.314240 4.111395 44.440620

H -0.292870 5.497995 45.617470

H -1.814899 4.891236 46.312725

H 50.935051 2.874897 47.839649

H 49.436489 2.303340 48.632198

H 50.323402 3.700207 49.301056

H 37.095943 17.355965 -2.638256

H 38.697674 17.302183 -1.852058

H 37.488552 18.525482 -1.344551

H 36.066261 15.520041 50.323402

H 37.351166 14.514805 49.605656

O 37.830803 15.396343 51.438648

C 22.984505 41.203918 51.289524

H 24.386261 39.955685 50.197258

H 25.093252 41.232765 51.213253

H 49.553829 35.415012 25.326960

H 50.211437 35.041958 26.940422

C 51.629818 34.988178 25.333317

C 50.864647 45.509418 38.593529

C -1.267788 40.657787 39.667217

H 3.454276 -3.069977 8.450143

H 3.553529 -2.175728 6.923220

O 4.866789 -1.583149 8.446720

H 1.853037 50.810375 7.460553

H 0.643918 49.576809 7.892765

H 1.739117 50.237839 9.147842

H 22.913122 0.354470 -0.551510

H 23.975073 -1.068306 -0.757351

H 23.700785 -0.379406 0.872737

H 30.098408 1.799746 50.117565

H 26.232941 48.165276 49.083969

C 27.279249 49.981152 48.584286

H 26.011946 49.512764 50.224640

H -1.068798 17.111992 33.948715

H -2.138569 19.245184 34.461601

H -0.554447 19.992754 34.289009

H 11.618884 -1.039463 43.470100

H 12.368412 -2.459797 42.712749

H 40.455368 50.193348 2.562472

H 42.735237 50.366920 3.388760

H 50.526306 23.117495 45.570045

H 51.260189 22.866673 43.969292

H 48.062599 -1.566525 40.139523

H 49.239937 -1.310326 41.432247

H 50.376205 0.047916 39.820744

H 31.484028 49.891190 7.422416

H 30.022621 33.052513 -1.582172

H 30.886070 34.487514 -2.173283

H 50.267666 12.944368 35.989990

H 51.067062 13.387338 37.508598

H 36.711647 11.953800 -1.756718

H 37.231865 12.386012 -0.113922

H 17.728529 -0.266956 32.218399

H 19.017344 -1.486828 32.187107

H 36.836811 2.004605 -0.283089

H 36.866146 2.865118 -1.835922

H 48.270393 -0.492840 27.267513

H 47.183018 -1.879929 27.047987

H 13.102293 42.089855 -2.343922

H 11.858949 43.332710 -2.561005

H 23.573662 49.624722 46.173382

H 24.246428 51.161911 45.640453

H 50.164993 44.437687 8.945427

H 35.214062 49.832031 28.711807

H 35.426254 51.321304 27.814623

H 33.675892 40.967766 -2.096523

H 35.331406 41.137424 -1.545990

H -2.560028 15.846645 40.501328

H -1.320107 15.162634 39.430088

H 47.363434 4.414531 51.840546

H 48.540283 4.634059 50.548798

H -2.187466 7.542692 45.706455

H -1.931267 9.285720 45.645828

H -1.764053 33.557571 42.140701

H -1.987984 34.296345 43.737057

H 50.272068 21.518209 -0.350071

H 49.283455 21.408201 -1.810989

H -2.247604 22.011049 48.420006

H -2.038342 21.538746 50.116096

H 36.576214 50.954121 38.259102

H 35.339226 50.692547 39.506847

H 49.434044 -1.650623 24.251318

H 50.714546 -0.939228 25.248241

H -1.121113 48.937782 23.281284

H -0.399944 50.546352 23.276884

H 13.450898 -2.200665 18.057577

H 11.698580 -2.089188 18.155363

H 50.957542 17.044029 2.065232

H 51.133556 18.299109 3.285596

H -2.160572 4.518184 39.017433

H -0.915760 4.627214 40.253933

H -2.037365 44.382927 33.166428

H -2.245159 42.912724 34.139397

H 39.772827 13.760389 51.060219

H 41.413177 13.346757 51.511986

H 17.201464 36.430511 -1.777252

H 16.329706 36.059906 -0.275269

H 51.080261 28.747988 44.947151

H 50.657829 29.593346 46.445713

H 51.006924 30.828867 43.684250

H 51.364330 31.540745 45.257618

H 50.627026 15.316648 5.217840

H 51.407845 13.882133 5.913584

H 39.301987 39.213493 50.558578

H 37.879208 40.090141 51.143826

C 26.541456 50.321445 12.208043

H 27.041142 51.099331 14.140776

H 28.053221 49.739132 13.585353

H 26.768808 49.913193 16.170805

H 6.638175 -2.730175 11.164182

H 6.717871 -1.105957 10.442035

H 8.757678 -2.553673 12.114657

H 9.021210 -0.900604 11.488341

H 9.070103 -1.216942 13.247015

H 27.926100 12.498466 -0.281132

H 28.883421 11.015548 -0.238598

C 28.455610 11.755784 -2.254444

O 29.601658 12.245201 -2.927212

H 29.212469 14.095304 -2.054962

H 30.922739 13.651359 -2.235867

H 29.501425 -0.687435 10.123254

C 30.056360 -1.547459 12.044252

H 31.532923 -2.047142 10.021069

O 31.407267 -1.987003 12.091678

H 32.961079 -1.434517 10.885004

H 9.402085 8.580197 50.065739

H 9.526762 6.864549 49.660416

C 10.777928 7.511401 51.361397

H 11.544568 9.692509 50.276466

H 12.955125 8.864754 49.570942

O 12.134704 7.923078 51.194672

H 7.801824 34.899193 50.553688

H 8.687761 36.400688 50.795219

O 9.523829 34.692863 51.640575

H 11.479540 34.715843 49.246784

C 10.820466 35.152946 51.294415

H 11.067373 36.408024 49.530849

H 50.396252 24.236160 41.879128

O 51.515900 24.546629 40.204548

H 51.613686 25.520573 42.035095

H 49.961597 26.981979 39.167534

H 51.026482 27.626877 40.413319

C 51.769161 25.784594 39.566010

C -2.364944 12.082388 22.630520

H -1.337219 10.401454 21.665865

H -0.618004 11.971401 21.322149

O -2.274002 11.988514 24.043034

H -0.564713 13.160964 23.883642

H -0.530975 11.904419 25.134809

F 32.913654 -2.491577 6.454827

F 33.702297 -1.089821 7.905965

F 49.200821 16.873394 -3.804348

F 50.201168 16.225565 -1.982113

C 5.195838 19.778116 52.825249

H 15.524442 6.390778 -1.947403

H 15.273622 7.873696 -0.992523

O 16.766808 6.609328 -0.278198

O 49.368038 38.157894 25.936165

H 49.939106 40.127785 26.310682

H 1.856949 33.552197 -2.414326

H 3.592643 33.126827 -2.392815

H 2.944324 34.246960 -1.169518

H -0.173080 29.994267 51.538879

H -0.345184 31.374512 52.659500

H 1.275124 30.687567 52.335342

H 6.022126 9.122418 52.041985

H 6.645020 10.773528 52.240978

H 7.737774 9.463691 51.699245

H 4.442889 8.657937 -2.794712

H 5.329315 7.143727 -3.101273

H 6.072486 8.718076 -3.527615

C 6.413758 31.987625 52.594475

C -3.102245 34.365284 29.178246

C 18.743055 49.751358 12.982017

H 18.498590 52.484463 6.206941

H 20.258732 52.296715 6.018215

O 19.319990 53.348888 4.484936

C -2.483265 16.244633 22.106390

H 33.993206 32.708305 50.624096

H 34.844433 32.952278 52.173019

H 35.225307 31.524122 51.171692

H 37.283695 34.942219 -3.484589

H 38.653183 34.247936 -2.564919

H 37.884098 35.770462 -2.029541

C 33.760967 -2.126347 14.203847

C 38.881508 50.065250 21.336329

C 9.250518 51.048973 20.405899

C 24.799404 -3.172165 20.113520

H 43.908180 37.024075 52.734795

H 44.747669 35.873135 51.649376

H 45.697655 36.987892 52.669277

H 43.350803 36.033016 -2.384502

H 43.207058 37.768711 -2.740440

H 42.217953 37.020649 -1.445759

H 3.745188 -3.104202 27.340853

H 2.957036 -1.503452 27.239157

H 3.829773 -1.972336 28.717186

H 4.415998 51.251877 23.878754

H 6.054884 51.147736 23.188387

H 5.843668 51.572128 24.907948

C 18.240437 46.398777 51.506607

C 51.621506 23.512058 24.930927

H -2.551716 4.744557 8.363602

H -2.366901 5.121521 10.078761

O -2.719910 6.756985 8.816838

C 5.177258 25.302513 53.436897

H 30.173702 -2.883205 35.373451

H 28.788080 -3.366756 36.379665

H 28.553885 -2.173775 35.070316

H 29.473557 51.920727 37.121368

H 31.078707 52.699104 37.076385

H 30.550665 51.837612 38.548550

C 9.699353 46.022305 52.629189

O 1.859393 -3.067535 32.748398

H 0.871270 -2.620167 34.535431

H 1.188095 -1.224274 33.475433

O 48.359379 50.937008 13.369736

H 48.259148 51.783340 11.458516

H 46.689201 51.365307 12.185553

O 25.226240 49.898037 31.079685

H 26.996161 50.739479 30.372694

H 26.753162 50.840687 32.137238

H -3.230835 29.643215 42.002338

O -2.108746 27.909475 42.384190

H -1.727383 29.902349 42.895119

C 41.222008 20.026003 -3.570152

O 4.437999 -3.698254 -1.166096

H 5.630984 -2.263737 -0.197529

H 5.250598 -1.912197 -1.892151

H 45.683964 50.470081 35.819355

O 45.849220 52.433617 35.123123

H 47.262714 51.230366 36.085331

C -2.855827 15.029158 12.564960

O -3.752033 28.594954 15.491684

H -2.864628 29.329811 13.752567

H -1.920998 29.589432 15.234018

H 43.105850 -2.214355 6.496387

O 44.184914 -0.439545 6.724716

H 44.603924 -2.345879 7.456641

C 49.921993 51.035774 3.474811

H 1.086888 51.753513 4.309900

H 0.113918 51.243073 2.894943

H 1.900952 51.189785 2.823559

H -1.220852 -3.024509 6.026038

H -1.420826 -2.492554 7.707461

H 0.208770 -2.866096 7.066965

C 40.190369 42.896587 -3.039665

C 52.643368 6.290547 30.866022

C 39.605614 7.546114 51.045547

C 51.727116 41.121292 46.981583

O -4.400352 32.954235 16.381533

H -2.985394 33.964363 15.217394

H -3.524193 34.834164 16.673912

H -1.091286 38.903999 50.745838

H 0.634628 38.987606 50.323891

O 0.241042 38.401382 52.283028

C 31.948999 -2.903255 27.748129

H 40.278866 49.548943 8.670650

H -2.688614 34.533470 12.919434

O -1.437447 32.857426 12.932634

H -1.762096 34.262608 11.418425

C -2.046162 5.078006 43.712608

C 37.468994 14.268874 52.212620

H 22.141106 40.664143 50.822109

O 23.187899 40.789307 52.627232

H 22.742975 42.282005 51.246986

O 51.605862 34.880611 23.919334

H 52.304539 34.230824 25.770416

H 51.999447 35.982655 25.643784

H 49.989464 46.166534 38.444897

H 51.277302 45.694721 39.601212

H 51.626884 45.770996 37.846935

H -0.721657 39.941017 39.031124

H -2.182087 40.972656 39.136730

H -1.564079 40.142944 40.591778

C 5.734636 -1.308369 7.357388

H 26.946289 50.954121 48.184834

H 27.726130 49.417419 47.745773

O 28.204300 50.170364 49.640858

H 25.520086 50.742905 12.208531

H 26.487185 49.321590 11.742095

O 27.424950 51.181961 11.496163

H 27.568205 12.326852 -2.586918

H 28.283997 10.713390 -2.561493

H 29.418798 -2.397705 11.750896

H 29.758604 -1.279037 13.068556

H 10.779395 6.464117 51.698269

H 10.317848 8.096647 52.175949

H 11.552390 34.410263 51.638130

H 11.041950 36.077019 51.856682

H 52.750443 26.170847 39.898968

H 51.860104 25.609070 38.485477

H -3.322266 11.650176 22.308317

H -2.387436 13.149230 22.338631

H 4.211137 19.825542 53.316132

H 5.838289 19.084326 53.386536

H 5.659341 20.777973 52.868271

C 17.818981 7.281116 -0.937275

C 49.018456 38.381824 24.584278

H 6.116001 30.931051 52.481041

H 5.800153 32.438419 53.388493

H 7.469841 32.028206 52.906898

H -2.957035 33.334133 29.541029

H -2.924767 34.382397 28.089891

H -4.144154 34.657173 29.359150

H 18.514236 49.553341 11.923488

H 17.922146 49.355812 13.600021

H 18.779236 50.842648 13.134562

C 19.410442 54.627926 5.083873

H -3.568195 16.380066 22.002249

H -2.198223 15.350872 21.527988

H -1.968910 17.122259 21.680534

H 34.310036 -3.057266 14.426310

H 32.731285 -2.391834 13.942760

H 34.235226 -1.627151 13.341866

H 38.342709 50.901314 21.801298

H 38.241993 49.167088 21.395000

H 39.806072 49.874565 21.909353

H 8.964005 50.160099 19.816252

H 8.455521 51.251877 21.144180

H 9.313590 51.912907 19.729712

H 24.201937 -2.997128 19.202646

H 24.598455 -4.187180 20.480705

H 25.869181 -3.112026 19.854876

H 17.531492 45.615513 51.184895

H 18.264883 46.396332 52.605228

H 17.863472 47.375656 51.158493

H 51.223030 23.779991 23.936937

H 51.342819 24.301678 25.649164

H 52.717682 23.471966 24.863457

C -4.089394 6.795121 9.157132

H 4.619881 25.267309 54.383949

H 5.684277 26.280857 53.376270

H 5.941453 24.506538 53.455963

H 8.630069 45.960209 52.876583

H 10.234730 45.240509 53.195366

H 10.070450 47.009449 52.953835

C 0.731436 -3.185368 31.900595

C 48.223946 52.144169 14.096772

C 24.464977 51.066574 30.867981

C -2.504780 27.626877 43.715542

H 41.517323 19.349325 -4.383728

H 40.280823 20.521774 -3.856663

H 42.010647 20.790197 -3.458675

C 5.590403 -4.505959 -1.299568

C 45.078186 52.879517 36.217831

H -2.636299 14.193581 13.252394

H -3.619045 14.688864 11.842325

H -3.289017 15.862290 13.135540

C -4.423820 29.829008 15.679920

C 44.917812 -0.623871 5.530264

H 49.979687 51.753513 4.309900

H 49.006718 51.243073 2.894943

H 50.793751 51.189785 2.823559

H 39.520538 42.043407 -3.237682

H 40.393764 43.417782 -3.984764

H 39.666241 43.599667 -2.374722

H 53.395828 5.716057 30.313046

H 52.217022 7.057186 30.192770

H 53.144028 6.795610 31.705023

H 39.275585 6.807833 50.296513

H 38.885422 8.378270 51.070972

H 39.612946 7.065010 52.033184

H 51.166801 40.542889 46.226189

H 52.771954 41.185825 46.649597

H 51.697289 40.564888 47.936943

C -5.509243 33.464188 15.667209

C 0.307534 39.711224 52.808624

H 31.023458 -2.885166 28.350491

H 31.822369 -3.618557 26.919399

H 32.748886 -3.269951 28.404272

C -2.368366 31.904018 12.469131

H -2.980015 5.373319 44.223049

H -1.447716 5.981545 43.511169

H -2.309696 4.622814 42.747952

H 36.409000 14.289899 52.510376

H 37.666523 13.315954 51.692890

H 38.084068 14.298699 53.123981

C 22.028162 41.053326 53.387516

C 52.890274 35.101120 23.372225

H 6.000124 -2.228046 6.807833

H 5.289712 -0.595512 6.644043

H 6.648932 -0.863934 7.771999

C 29.502893 50.522884 49.209137

C 27.239645 50.987366 10.101253

H 17.618031 8.358713 -1.059017

H 18.029221 6.844992 -1.927353

H 18.712252 7.165240 -0.308022

H 49.153889 37.435749 24.040588

H 49.655525 39.143574 24.111973

H 18.534771 54.850388 5.719969

H 19.444668 55.368652 4.271764

H 20.324247 54.743801 5.692589

H -4.682953 6.039728 8.613444

H -4.244873 6.666045 10.239130

H -4.457558 7.794490 8.891155

H -0.187260 -3.423962 32.458462

H 0.930430 -4.005299 31.196053

H 0.547599 -2.260803 31.326595

H 48.631714 51.971088 15.101519

H 47.169327 52.445839 14.216071

H 48.781322 52.975838 13.631313

H 23.430895 50.746326 30.674854

H 24.821896 51.634708 29.992798

H 24.469868 51.727116 31.752449

H -2.383038 26.548302 43.875420

H -3.560863 27.875740 43.911110

H -1.889706 28.161764 44.458710

H 5.269177 -5.552753 -1.217918

H 6.084709 -4.346081 -2.272537

H 6.333084 -4.312832 -0.507019

H 44.189804 52.243423 36.371353

H 45.659519 52.908855 37.152660

H 44.739357 53.897957 35.987545

H -4.698597 30.304245 14.720156

H -5.347404 29.643703 16.246588

H -3.805328 30.546265 16.248543

H 44.329147 -1.148983 4.762648

H 45.856556 -1.180763 5.690144

H -6.314503 32.719063 15.738103

H -5.278957 33.626511 14.599879

H -5.874958 34.414173 16.094532

H -0.668854 40.224594 52.779289

H 0.617026 39.621258 53.856884

H 1.049242 40.338516 52.285469

H -2.574696 32.005714 11.390066

H -3.316887 31.956333 13.027486

H -1.910244 30.919806 12.636344

H 21.162271 40.462700 53.047710

H 22.239378 40.784904 54.431374

H 21.743116 42.117233 53.345467

H 52.787601 35.048801 22.279472

H 53.293152 36.095112 23.631357

H 53.613888 34.333504 23.696384

H 29.978621 49.717621 48.624378

H 30.098408 50.692547 50.117565

H 29.518538 51.445000 48.605309

H 26.184538 51.088573 9.795183

H 27.596563 49.993866 9.774159

H 27.820980 51.749115 9.565387

Li -0.001465 16.407934 12.406548

Li 34.243050 49.470711 5.696989
