# Supplementary material for: Unlocking Li‒S chemistry via acoustic-induced entropy-driven electrolyte
Source: Nat Commun. 2026 Apr 30;17:5915. doi: 10.1038/s41467-026-72486-6 (PMC13338023; doi:10.1038/s41467-026-72486-6)
Supplement: Supplementary file 4 — Transparent Peer Review file [file 41467_2026_72486_MOESM4_ESM.pdf]

nature portfolio

Peer Review File

Unlocking Li–S chemistry via acoustic-induced entropy-driven electrolyte

Corresponding Author: Professor Jingyu Sun

This file contains all reviewer reports in order by version, followed by all author rebuttals in order by version.

Version 0:

Reviewer comments:

Reviewer #1

(Remarks to the Author)
This study proposes a strategy employing an ultrasonic field to induce a high-entropy state within the electrolyte, aiming to mitigate the sluggish kinetics of sulfur conversion and suppress lithium dendrite growth in lithium–sulfur batteries. Leveraging the cavitation effect generated by ultrasonics, this approach enhances electrolyte mass transport, optimizes the lithium-ion solvation structure, and promotes both efficient sulfur species conversion and uniform lithium deposition. Comprehensive evidence from computational simulations and experimental measurements demonstrates that the introduction of an external acoustic field yields beneficial effects across multiple scales, from the molecular level to the full-cell configuration. Overall, this work exhibits strong innovation and a well-conceived experimental design; however, several issues still require further clarification before it can be recommended for publication:
1. In the AIMD simulations, the authors state that a cavity is formed within the high-entropy electrolyte during the low-pressure rarefaction phase of the ultrasonic field. Further clarification of the underlying mechanism is needed. Moreover, essential simulation details, including the initial configuration (e.g., electrolyte concentration, ion–solvent ratio) and the force field parameters, are not provided and should be supplemented.
2. Cavitation-induced convection enhancement is evidenced by monitoring bubble dynamics in the ultrasonic field. However, the reported bubble migration velocity is derived from the trajectory of a single bubble, without multiple independent measurements to ensure statistical validity. Repeated experiments are recommended to substantiate the reliability of the data.
3. Although the H-type electrolytic cell tests suggest that ultrasound causes no immediate electrode damage, long-term cycling durations are significantly longer than 120 min. The authors should clarify how they ensure that extended ultrasonic exposure will not degrade the electrode materials or separators, and provide post-cycling morphological characterization of these components.
4. While ultrasound-induced desolvation and enhanced mass transfer are repeatedly discussed throughout the manuscript, their mechanistic connection to sulfur species conversion kinetics remains insufficiently defined. It is currently unclear whether they are in a parallel relationship or a sequential relationship.
5. The manuscript contains a mislabeling: Fig. 6e presents the SANS results, whereas Fig. 6g corresponds to XPS data. This contradicts the figure descriptions in the text and should be corrected.
6. The claim that ultrasound-induced mass transfer enhancement and desolvation increase Li+ mobility is currently supported only by computational results. Additional experimental verification would strengthen this conclusion.
7. While ultrasound-enhanced Li-species mass transfer may accelerate sulfur conversion kinetics, it may also increase polysulfide migration, potentially aggravating the shuttle effect. The authors should address this concern, for example by visualizing polysulfide migration under ultrasonic conditions in an H-type cell or by employing operando Raman spectroscopy at the anode side to confirm whether the shuttle effect is suppressed.
8. The feasibility of integrating the ultrasonic device into practical battery systems is not discussed. Expanding on potential integration strategies would enhance the practical significance of this study.

Reviewer #2

(Remarks to the Author)
The purpose of this work is to induce a “high-entropy state” in the electrolyte via an external ultrasonic field, thereby enhancing convection, altering solvation structures and ionic clusters, and consequently promoting Li^{+} desolvation as well

as uniform transient nucleation of sulfur/lithium species, while suppressing the polysulfide shuttle and lithium dendrite growth. Compared with conventional modification strategies, this approach reduces the introduction of additional materials and associated side effects, offering relatively high novelty. However, the manuscript still suffers from insufficient experimental evidence, incomplete mechanistic justification, and inadequate assessment of the broader impacts of the external field.

1. The manuscript reports that the optimal electrochemical performance is achieved under 180 W ultrasonic irradiation. Has the effect of applying ultrasonic power beyond 180 W been evaluated to determine whether further improvements can be achieved, or whether detrimental side effects may occur?
2. The authors attribute the enhanced kinetics to increased molecular disorder and entropy induced by ultrasonication. However, has the possibility been considered that such intensified molecular motion may also aggravate exothermic interfacial side reactions, potentially leading to thermal runaway risks?
3. For the Ah-level Li–S pouch cell cycled at 0.5 C over 150 cycles, only a single representative voltage profile is shown. To fully demonstrate the cycling stability, discharge/charge voltage curves at different stages (initial, mid-cycle, and end-of-life) should be provided.
4. Direct evidence for the suppression of the polysulfide shuttle is limited. Although kinetics, impedance, and morphology data indicate improvements, more direct verification is needed. Could UV–Vis absorption spectroscopy or equivalent techniques be employed to quantitatively track the evolution of polysulfide concentration under ultrasonic conditions?
5. The manuscript only investigates the influence of ultrasonication on coin cells with low sulfur loading, while no corresponding tests were conducted on pouch cells with high sulfur loading. Since high-loading sulfur cathodes are typically thicker and more porous than low-loading ones, ultrasonic agitation may induce substantial cathode detachment in pouch cells, thereby compromising the overall electrochemical performance ?
6. There appears to be some ambiguity regarding whether ultrasonication is applied in situ during cell operation or ex situ as a pre-treatment of the electrolyte. For clarity, it is recommended to explicitly differentiate which experiments involved continuous ultrasonic irradiation during cycling and which involved only pre-sonicated electrolytes. A comparison between the two conditions, including reversibility after removing the external field, would strengthen the conclusions ?
7. The XPS results indicate LiF enrichment in the SEI, which is attributed to modified desolvation and $TFSI^-$ decomposition. However, the contribution from $LiNO_3$ depletion and related side reactions is not distinguished. Advanced characterization such as in situ/operando XPS, depth-profiling TOF-SIMS, or GC-MS/NMR of electrolyte decomposition products would be useful to separate the respective roles of $LiNO_3$ and TFSI.
8. Given the application of an external ultrasonic field, it is necessary to reassess the device performance across a broader range of cathode areal loadings and electrolyte-to-sulfur (E/S) ratios. The potential effects on cathode integrity, separator stability, anode morphology, and current collector adhesion under varying structural conditions should be systematically evaluated.

Reviewer #3

(Remarks to the Author)
In this study, the authors present an exogenous modification approach to Li–S battery electrolytes by applying ultrasound. They propose that ultrasound induces bubble nucleation and collapse, which in turn generates a “high entropy” electrolyte environment that improves cell performance. A series of electrochemical and structural characterizations are provided to support this claim and to demonstrate performance stabilization.

However, the manuscript contains several questionable claims, flawed arguments, and inconsistencies. In particular, the central assertion that ultrasound can induce “high entropy” in the electrolyte is problematic and not supported by thermodynamic reasoning. The reviewer does not agree with this interpretation. Specific objections and concerns are detailed below:

1. The first objection is in the use of the term “high entropy”. This term is often misused in battery related literature. High entropy generally refers to enhanced entropy of mixing due to the presence of multiple compositional constituents. Cavitation may enhance the mixing, solvation kinetics, dissolution, and homogenization but it does not fundamentally change the intrinsic entropy of the electrolyte, the later being more chemical in nature. From thermodynamic point of view, what the authors claim as high entropy is not actually high entropy. The use of the term "high entropy" will mislead the readers.
2. The authors state that “An ultrasonic field induces highly localized and chaotic effects within the electrolyte, disturbing the equilibrium and promoting a state of high entropy”. The reviewer cannot agree with this statement either. The term "entropy" from thermodynamics point of view is tied to composition, temperature, and energy distribution. One cannot correlate the local chaotic effect induced by transient fluctuations to a bulk state of higher entropy. The latter exists with or without the exogenous effect. In electrolyte chemistry, the term "high entropy" has well established meaning. The manner in which this term is used here is not responsible and does not correlate to thermodynamic principles.
3. A rotational mass transfer model was used to explain the mass transport and electrochemical reaction kinetics. How did the authors make sure that the rotational mass transfer model be effectively predicting the mass transport in such system? This seems like a over simplification because such model will not account for the spatial heterogeneity because of the random nature of the bubble formation and collapse.
4. The authors mention that they expect higher ionic mobility in the presence of ultrasound. Have they measured the ionic conductivity to support this claim?
5. What is the equivalent circuit used for fitting? is the EIS measured on the same cell before and after ultrasound?
6. Is the ultrasonication power optimized? They show improved performance with increasing ultrasonication power from 0 to 90 to 180. Best performance is achieved at 180. What would happen if they go higher? Will the performance keep on improving?
7. To explain the Li metal deposition difference, the authors refer to the preferential Li deposition on the higher electric field

intense region without the ultrasound. In the case of ultrasound, even though the rate of mixing would be enhanced, the electric field intensity distribution on the electrode surface should remain unchanged. How did the authors account for this when formulating the Li deposition mechanism?
8. Figures are mislabeled in the manuscript. Figure 6e in the text talks about XPS but in the Figure it shows SANS data.
9. The authors do not provide any details on the electrochemical cycling conditions (rate, cycle numbers, voltage etc.) for the ex-situ samples.
10. XPS needs additional core level elemental analysis to corroborate with the Li1s spectra.
11. The authors do not show the long-term cycling performance of the multi-layer pouch cell. What is the reason behind this?
12. Another questionable analysis is their energy density reporting and calculation for the Li-S pouch cell. The authors do not provide any information on the thickness of the Li metal used in the pouch cell. Thin Li metal is critical for high energy density (low N/P ratio). Assuming the loading of S cathode being 1.8 - 2.0 mg/cm2 of S loading, Li metal has to be extremely thin in addition to the lean electrolyte loading. What thickness of Li metal and S loading in cathode for pouch cells did the authors utilize? The authors did not include the mass of the Al-plastic film for energy density calculation. This weight should not be excluded for cell level energy density calculation. Right now, the reported energy density is giving the impression that the energy density is at the cell level. The authors should explicitly mention it in the manuscript including in the abstract that the energy density does not take into consideration the mass of the cell housing. Or, include the mass of the cell housing and revise the energy density of the cell.

Version 1:

Reviewer comments:

Reviewer #1

(Remarks to the Author)
Previous issues have been well addressed and I think the current version is acceptable.

Reviewer #2

(Remarks to the Author)
I am pleased with the changes made and it can be published in the current form.

Reviewer #3

(Remarks to the Author)
The authors modified the manuscript based on the previous round of comments, replacing the problematic “high-entropy” term with “acoustic-induced entropy driven state”, which is a good start. However, their claims are still rationalized based on equilibrium excess-entropy framework and utilizing structural-chemical proxies to imply entropy increase. Justification of entropy increase in this work still requires concrete measurement related to thermodynamic parameters (e.g., calculation of entropy term, entropy driven free energy decomposition, explicit excess entropy calculation from RDFs etc.). No explicit measurement of the ΔS term change upon ultrasonication is present in the work right now. This is necessary because the entropy change is the central assertion of this work.

1. The claim of entropy increase is rationalized by observations through Raman and Ab initio molecular dynamics simulations: demonstration of disrupted ion solvation shells and liberated solvation molecules. These analyses are structural proxies but not actual entropy measurements. To claim entropy increase, the authors need actual entropy measurement. Furthermore, if solvent molecules are released from the solvation shells, it would imply that the coordination number has decreased? Since the release of solvent molecules from the solvation shells is argued for a cause of enhanced entropy, the reviewer is curious if the authors think that solutions with smaller coordination number of the solvated species would be inherently more entropic than those of higher coordination numbers.
2. The work by Michaelidis et al referenced in the response in support of exogenous field effect to change entropy is an entirely different perturbation method. It can be best explained as a “conceptual inspiration” but not as a near-confirmation of their observations. Furthermore, the Frank and Evans paper they cited is about entropy in mixtures/solutions and interpretation of entropy data in condensed system. It does not specifically establish ultrasound disruption of solvation shells leads to entropy increase. The bottom line is the claim of thermodynamic entropy increase requires actual quantification of the entropy. They currently lean towards an equilibrium excess-entropy framework-based argument that does not directly apply to a cavitating non equilibrium electrolyte.
3. As for the rotational mass transfer model, the authors explain their rationalization of selecting the model of choice. However, the original criticism still holds. How does the model best reflect the mass transport modification induced by the random nature of bubble formation/collapse?
4. For multi-layer pouch cell, the authors state that “Simply achieving operation under these conditions demonstrates the efficacy of the acoustic method.” Is there an initial cycle performance of the similar pouch cell without the acoustic in the manuscript to reaffirm this statement?

Version 2:

Reviewer comments:

Reviewer #3

(Remarks to the Author)
The authors have responded to the reviewer's comments satisfactorily. The manuscript can now be accepted for publication at its current form.

Open Access This Peer Review File is licensed under a Creative Commons Attribution 4.0 International License, which permits use, sharing, adaptation, distribution and reproduction in any medium or format, as long as you give appropriate credit to the original author(s) and the source, provide a link to the Creative Commons license, and indicate if changes were made.
In cases where reviewers are anonymous, credit should be given to 'Anonymous Referee' and the source.
The images or other third party material in this Peer Review File are included in the article's Creative Commons license, unless indicated otherwise in a credit line to the material. If material is not included in the article's Creative Commons license and your intended use is not permitted by statutory regulation or exceeds the permitted use, you will need to obtain permission directly from the copyright holder.
To view a copy of this license, visit https://creativecommons.org/licenses/by/4.0/

Point-by-Point Response to Reviewers

Reviewer 1 (Comments to the Author):

This study proposes a strategy employing an ultrasonic field to induce a high-entropy state within the electrolyte, aiming to mitigate the sluggish kinetics of sulfur conversion and suppress lithium dendrite growth in lithium–sulfur batteries. Leveraging the cavitation effect generated by ultrasonics, this approach enhances electrolyte mass transport, optimizes the lithium-ion solvation structure, and promotes both efficient sulfur species conversion and uniform lithium deposition. Comprehensive evidence from computational simulations and experimental measurements demonstrates that the introduction of an external acoustic field yields beneficial effects across multiple scales, from the molecular level to the full-cell configuration. Overall, this work exhibits strong innovation and a well-conceived experimental design; however, several issues still require further clarification before it can be recommended for publication:

Our response:

We are grateful for the reviewer's positive and professional evaluation. We have revised the manuscript to enhance its clarity. All requested revisions based on the constructive comments from the reviewer have been performed.

Specific comments:

1. In the AIMD simulations, the authors state that a cavity is formed within the high-entropy electrolyte during the low-pressure rarefaction phase of the ultrasonic field. Further clarification of the underlying mechanism is needed. Moreover, essential simulation details, including the initial configuration (e.g., electrolyte concentration, ion–solvent ratio) and the force field parameters, are not provided and should be supplemented.

Our response:

We thank the reviewer for the constructive comment. We agree that a deeper explanation of the cavitation mechanism and a more detailed description of the simulation parameters are necessary.

We have expanded the discussion in the revised manuscript to clarify the physical origin of the cavity formation. During the rarefaction phase induced by the ultrasonic field, the local pressure drops significantly. When this local pressure falls below the saturated vapor pressure, the intermolecular forces holding the liquid phase together are overcome. This rupture in the

liquid structure leads to the spontaneous formation of cavities characterized by internal negative pressure (*Ultrasound in Chemistry: Analytica Applications,* **2009***,* Wiley-VCH Verlag GmbH & Co. KGaA). In our AIMD simulations, a vacant cavity of 20 Å in diameter was generated at the center of the simulation box to mimic the low-pressure rarefaction phase induced by the ultrasonic field. This simulated void creates an instantaneous and pressure gradient relative to the surrounding bulk electrolyte. The resulting interfacial tension and unbalanced intermolecular forces at the cavity boundary generate local shear force, inducing a structural transition of the electrolyte from an ordered, solvated state to a more disordered, partially desolvated state. The simulation provides a molecular-level link between macroscopic acoustic mediation and microscopic solvation-shell modification.

We apologize for the omission of specific configuration details in the original submission. We have updated the "Methods" section to include the exact composition of the simulation box. The electrolyte model consists of a mixed solvent system of DOL, DME, LiTFSI, and $LiNO_3$. The LiTFSI salt concentration was set to 1 M, corresponding to a Li-ion: solvent molecular ratio of approximately 1:7.6. Specifically, the simulation box contained about 100 Li-ions, 75 $TFSI^-$, 25 NO_3^- 454 DOL, and 306 DME molecules, ensuring a realistic liquid-phase density ($\approx$1.12 g cm^{-3}).

As for the force field, we clarify that we did not use a classical force field with empirical parameters. Instead, we employed an on-the-fly machine learning force field trained via ab initio density functional theory calculations using the VASP code. This DFT calculations utilized the PBE functional with DFT-D3 dispersion corrections to accurately capture the van der Waals forces essential for solvation dynamics, enabling the interpretation of the impact of cavitation. (*Phys. Rev. B,* **2019***, 100,* 014105; *Chem. Sci.,* **2025***,16,* 3620-3629)
The related explanations have been incorporated into the revised manuscript.

Revisions

Results section:

"During this rarefaction phase, the local pressure drops below the saturated vapor pressure of the electrolyte, overcoming intermolecular forces and inducing rupture of the liquid structure. This leads to the formation of cavities with internal negative pressure. In the AIMD model, a central 20 Å void was generated to mimic this state, creating an instantaneous pressure gradient and interfacial tension. The resulting local shear forces at the cavity boundary drive the structural transition from an ordered solvated state to a disordered, partially desolvated state, providing a molecular link between macroscopic acoustic mediation and microscopic solvation modification."

Methods section:

"The electrolyte model comprised a mixed solvent system of DOL, DME, $LiNO_3$ and LiTFSI salt at a concentration of 1 M. The simulation box was constructed with a Li-ion: solvent

molecular ratio of approximately 1:7.6, containing 100 Li-ions, 75 $TFSI^{-}$, 25 NO_3^{-}, 454 DOL, and 306 DME molecules. An on-the-fly machine learning force field was utilized, trained via ab initio DFT calculations. These calculations employed the PBE functional with DFT-D3 dispersion corrections to accurately capture the van der Waals interactions critical for solvation dynamics."

2. Cavitation-induced convection enhancement is evidenced by monitoring bubble dynamics in the ultrasonic field. However, the reported bubble migration velocity is derived from the trajectory of a single bubble, without multiple independent measurements to ensure statistical validity. Repeated experiments are recommended to substantiate the reliability of the data.

Our response:

We appreciate the reviewer for the instructive comment. We conducted multiple supplementary measurements (n=5) in the custom chamber using a microscope camera to statistically characterize convection enhancement. By tracking the movement trajectories and calculating the speed, the results were obtained. The average migration speed is calculated as 398.5 μm s^{-1}, consistent with the value reported in the original submission, validating that the ultrasonic cavitation can significantly enhance convective mass transfer. We have supplemented these images and the data in the revised manuscript.

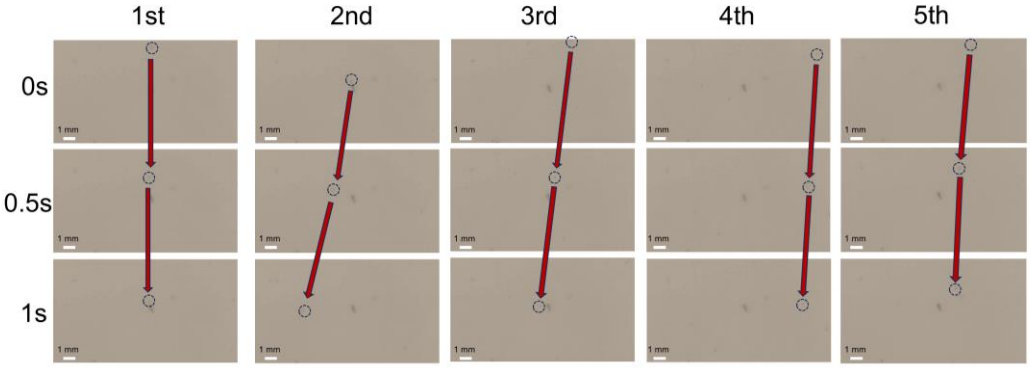

Supplementary Fig. 3|Representative time-resolved microscopy images showing the displacement of multiple bubbles in the ultrasonic field.

Revisions

Results section:

"To ensure statistical validity, multiple independent measurements (n=5) were conducted by tracking bubble trajectories within the liquid phase over a fixed time interval. The average migration speed is determined to be 398.5 μm s^{-1}. This result is consistent with our initial observations."

3. Although the H-type electrolytic cell tests suggest that ultrasound causes no immediate electrode damage, long-term cycling durations are significantly longer than 120 min. The authors should clarify how they ensure that extended ultrasonic exposure will not degrade the electrode materials or separators, and provide post-cycling morphological characterization of these components.

Our response:

We're grateful for the reviewer's constructive comment. We agree that long-term mechanical stability under the influence of ultrasound is important. Following the reviewer's suggestion, we examined cells of different loadings (2 and 6 mg cm^{-2}) subjected to ultrasonic treatment (180 W) for prolonged duration, and evaluated the electrodes and separators post-cycling. It's observed through digital photo characterization that even under the high loading of 6 mg cm^{-2}, the electrodes and separators well maintain their integrity without damage after the 100 cycles.

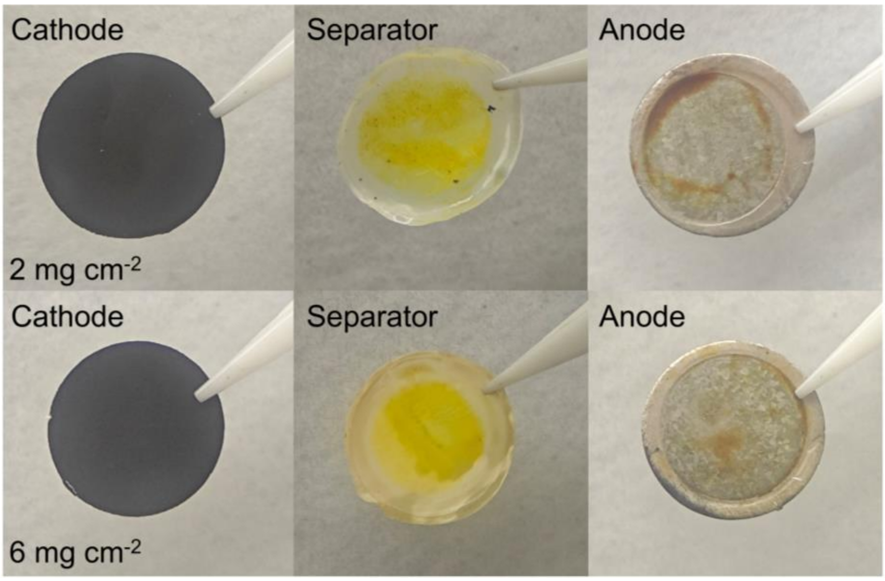

Supplementary Fig. 15|Photographs of cell components after 100 cycles (0.5 C, 180 W ultrasonication) at different sulfur loadings (2 vs. 6 mg cm^{-2}).

The stability is in alignment with the reported post-cycling morphological characterization of the cathodes subjected to ultrasound that display a well-preserved and uniform morphology after 100 cycles as shown in Supplementary Fig. 42 and 43. Similarly, for lithium anode, the electrode subjected to long-term ultrasound (180 W) exhibit a smooth and dense surface without fracture and damage after 100 cycles (Supplementary Fig. 35).

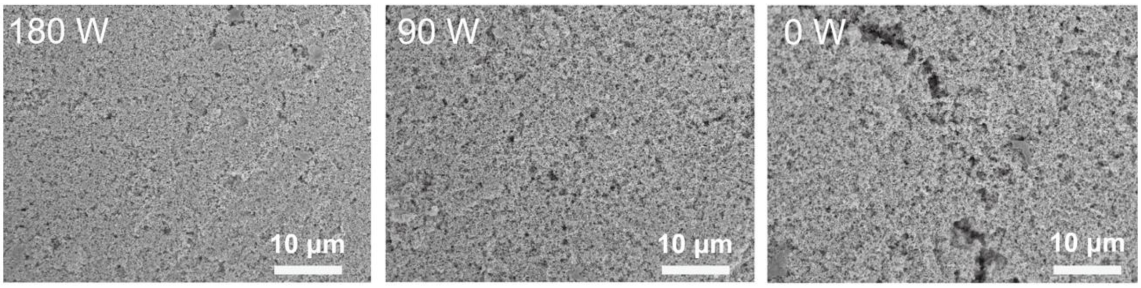

Supplementary Fig. 42|Front-view SEM images of sulfur cathodes cycled for 100 times at 0.5 C within a voltage window of 1.7–2.8 V, using ultrasonic-field-enabled electrolytes. Cells were disassembled in the fully charged state (2.8 V) for post-mortem analysis.

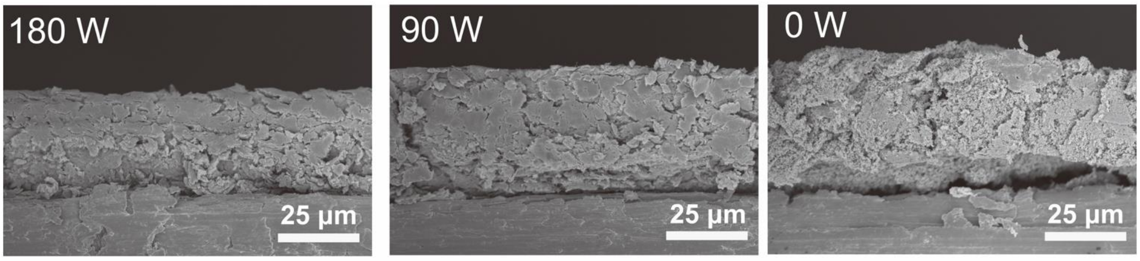

Supplementary Fig. 43|Cross-sectional SEM images of sulfur cathodes cycled for 100 cycles at 0.5 C within a voltage window of 1.7–2.8 V using ultrasonic-field-enabled electrolytes. Cells were disassembled in the fully charged state (2.8 V) for post-mortem analysis.

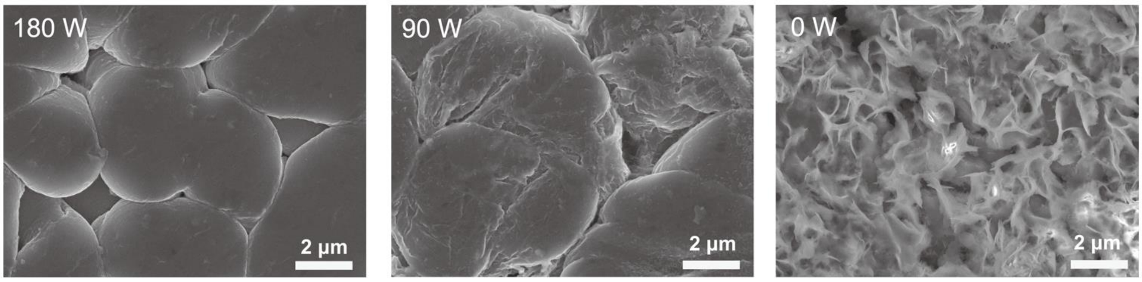

Supplementary Fig. 35|SEM images of lithium anodes after 100 cycles at 2.0 mA cm^{-2} and 2.0 mA h cm^{-2} in electrolytes subjected to ultrasonic fields of (a) 180 W, (b) 90 W, and (c) 0 W. Cells were disassembled at the symmetric cell's near-zero potential state for post-mortem analysis.

In addition, from an electrochemical perspective, any mechanical degradation of the separator or active material would manifest as internal short circuits or rapid capacity decay. However, our data indicates superior stability under ultrasonic conditions. The Li||Li symmetric cells operated stably for over 1000 hours under a 180 W ultrasonic field without short-circuiting, and the full Li-S cells maintained a capacity retention of 76.5% after 900

cycles at 1.0 C. This suggests that the cathode structure and the separator-electrode interface remained mechanically robust throughout the extended ultrasonic exposure. In summary, both electrochemical data and post-mortem imaging confirm that the ultrasonic parameters employed in this study do not compromise cell stability. The related information has been incorporated into the revised manuscript.

Revisions

Results section:

"The long-term mechanical stability of the cell components under sustained ultrasonic exposure was further verified across different sulfur loadings (2.0 and 6.0 mg cm^{-2}). Digital photographs of the electrodes and separators harvested after 100 cycles (0.5 C) at 180 W confirm that all components maintain their structural integrity without delamination or physical damage, even at the high loadings (Supplementary Fig. 15)."

"The surface morphology of the lithium anode was characterized post-cycling using SEM, with the results presented in Supplementary Fig. 35. The electrode subjected to long-term ultrasound (180 W) is free of fracture and damage."

"As observed from the microscopic morphologies of the front and cross-section in Supplementary Fig. 42 and 43, the electrode structure under the field influence is well-preserved with the surface developing a noticeably denser and more uniform morphology."

4. While ultrasound-induced desolvation and enhanced mass transfer are repeatedly discussed throughout the manuscript, their mechanistic connection to sulfur species conversion kinetics remains insufficiently defined. It is currently unclear whether they are in a parallel relationship or a sequential relationship.

Our response:

We thank the reviewer for highlighting the need to clarify the mechanistic hierarchy of these phenomena. We have revised the discussion to explicitly define the relationship between enhanced mass transfer and desolvation as parallel consequences of a single physical origin, acoustic cavitation. They function synergistically to accelerate sulfur conversion. Both effects originate simultaneously from the collapse of cavitation bubbles. On the macroscopic scale, the collapse generates micro-jets and turbulent fluid motion that drive enhanced convective mass transfer, ensuring a high flux of active species to the electrode interface. Concurrently, on the microscopic scale, the collapse generates shear forces that overcome the intermolecular forces holding the solvation shell together. This results in a partially desolvated Li-ion structure. Although enhanced mass transfer facilitates rapid ion transport, it does not inherently mitigate the kinetic bottlenecks associated with ion desolvation at the electrode interface. Conversely, accelerated desolvation alone is limited by the rate of ion arrival at the interface. Ultrasonic

irradiation provides a synergistic effect by simultaneously augmenting mass transport and facilitating Li-ion desolvation, thereby ensuring a high flux of kinetically active species at the electrode surface. They synergistically contribute to the observed enhanced sulfur species conversion kinetics. We have updated the corresponding discussion section to clearly articulate this parallel relationship.

Revision

> **Results section:**
> "The relationship between ultrasound-induced desolvation and enhanced mass transfer is characterized by a parallel mechanism, both originating simultaneously from the physical phenomenon of acoustic cavitation. On the macroscopic scale, the collapse of cavitation bubbles generates turbulent fluid motion, which drive enhanced convective mass transfer and ensure a high flux of active species to the electrode interface. Concurrently, on the microscopic scale, the localized shear forces produced during bubble collapse overcome the intermolecular forces within the solvation shell, resulting in a partially desolvated Li-ion structure. These two effects function in parallel to resolve different kinetic bottlenecks. By simultaneously augmenting transport and lowering the desolvation barrier, ultrasonic irradiation ensures a high flux of kinetically active species at the electrode surface."

5. The manuscript contains a mislabeling: Fig. 6e presents the SANS results, whereas Fig. 6g corresponds to XPS data. This contradicts the figure descriptions in the text and should be corrected.

Our response:

We appreciate the reviewer's comment and apologize for the error. The correct figure assignment and corresponding description have been updated in the revised manuscript.

Revisions

> **Results section:**
> "As shown in Fig. 6g, the SEI formed under all conditions primarily comprises lithium oxides, hydroxides, carbonate, and lithium fluoride (LiF)."
> "As shown in Fig. 6e, the R_g value for the lithium layer formed with the 180 W entropic electrolyte is 98.1 Å."

6. The claim that ultrasound-induced mass transfer enhancement and desolvation increase Li^+ mobility is currently supported only by computational results. Additional experimental verification would strengthen this conclusion.

Our response:

We thank the reviewer for this valuable suggestion. We agree that while our COMSOL and AIMD simulations strongly suggest enhanced Li-ion mobility, experimental validation is crucial to substantiate this claim. To address this, we conducted electrochemical impedance spectroscopy measurements to quantify the ionic conductivity (which is proportional to ion mobility) of the electrolyte under different ultrasonic power conditions (0 W, 90 W, and 180 W). The ionic conductivity σ was calculated using the equation $\sigma=L/R_bA$, where L is the distance between electrodes, A is the area, and R_b is the bulk resistance derived from the high-frequency intercept of the Nyquist plot. Our results show a clear positive correlation between ultrasonic power and ionic conductivity. Specifically, the conductivity increased from 0.187 mS cm^{-1} in the pristine state (0W) to 0.250 mS cm^{-1} at 90 W, and further to 0.361 mS cm^{-1} under 180 W excitation. These findings align with our computational models, experimentally validating the mechanism of acoustic-driven mobility enhancement proposed in our manuscript. We have added these new experimental data and the corresponding discussion to the revised manuscript.

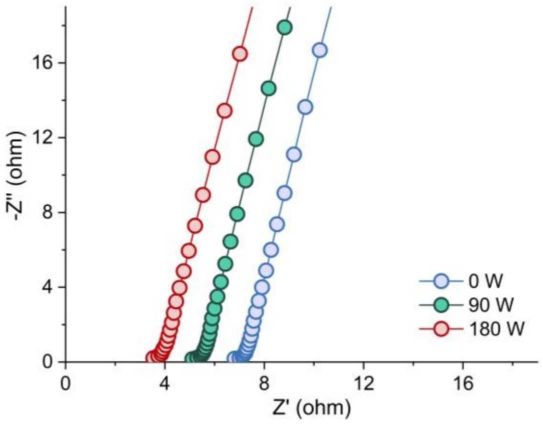

Supplementary Fig. 9|Electrolyte ionic conductivity under ultrasonication at different power levels evaluated by EIS in symmetric cells.

Revisions

Results section:

"To experimentally validate the Li^+ mobility enhancement suggested by the computational results, the electrolyte ionic conductivity (σ) was quantified under varying ultrasonic power levels using electrochemical impedance spectroscopy. As shown in Supplementary Fig. 9, a clear positive correlation between acoustic power and ion transport was observed, with ionic conductivity increasing progressively as the ultrasonic power is raised."

Supplementary Information section:

"The ionic conductivity σ was calculated using the equation $\sigma=L/R_bA$, where L is the

distance between electrodes, A is the area, and R_b is the bulk resistance derived from the high-frequency intercept of the Nyquist plot. The conductivity increased from 0.187 mS cm^{-1} in the pristine state (0 W) to 0.250 mS cm^{-1} at 90 W, and further to 0.361 mS cm^{-1} under 180 W excitation."

7. While ultrasound-enhanced Li-species mass transfer may accelerate sulfur conversion kinetics, it maiiy also increase polysulfide migration, potentially aggravating the shuttle effect. The authors should address this concern, for example by visualizing polysulfide migration under ultrasonic conditions in an H-type cell or by employing operando Raman spectroscopy at the anode side to confirm whether the shuttle effect is suppressed.

Our response:

We greatly appreciate this insightful comment. We agree that enhanced mass transport is potentially a "double-edged sword", while it aids ion supply, it theoretically poses a risk of accelerating the migration of soluble polysulfides (LiPSs) to the anode. To rigorously address this, we followed the reviewer's suggestions and performed operando Raman spectroscopy at the anode interface. Our results indicate that the ultrasonic field effectively suppresses the shuttle effect. It is crucial to emphasize that the shuttle effect is fundamentally driven by the accumulation of soluble LiPSs that fail to convert quickly at the cathode. As demonstrated in our main text, the ultrasonic field significantly accelerates the sulfur conversion kinetics. By facilitating the rapid transformation of soluble LiPSs into solid Li_2S_4, the source of shuttle species is depleted. Therefore, the total amount of LiPSs available to migrate to the anode can be reduced. To confirm this in cells under operation, we performed operando Raman spectroscopy specifically at the anode-separator interface during an LSV discharge process (0.5 mV s^{-1}). For the non-ultrasonic group, the Raman mapping reveals a rapid increase in signal intensity for polysulfide species at the anode side as discharge progresses, confirming severe shuttling. In stark contrast, under ultrasonication, the noticeable appearance of signals corresponding to LiPSs is delayed and the intensity of these LiPS signals is markedly suppressed and remains lower throughout the discharge process. This confirms that the ultrasonic field does not aggravate the shuttle effect. Instead, by suppressing the accumulation of LiPSs at the cathode, the ultrasonic strategy effectively protects the lithium anode. We have added these new data and discussion to the Supplementary Information and the revised manuscript.

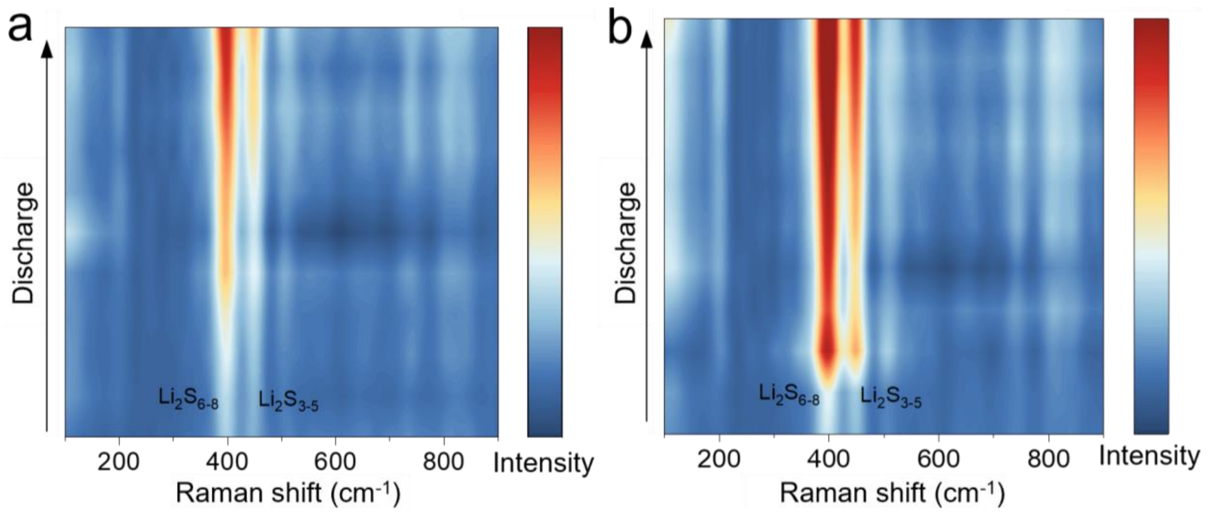

Supplementary Fig. 25|Time-resolved Raman spectra recorded at the anode/separator interface during discharge (a) with and (b) without 180 W ultrasonication.

Revisions

Results section:

"While enhanced mass transport typically aids ion supply, it theoretically poses a risk of accelerating the migration of soluble polysulfides to the anode. To rigorously address this, operando Raman spectroscopy was performed at the anode-separator interface during discharge. The results indicate that the ultrasonic field effectively suppresses the shuttle effect as shown in Supplementary Fig. 25."

Supplementary Information section:

"In the absence of ultrasound, Raman mapping reveals a rapid increase in signal intensity for polysulfide species at the anode side, confirming significant shuttling. In stark contrast, under 180 W ultrasonication, the appearance of lithium polysulfide signals is markedly delayed and the overall intensity remains substantially lower throughout the process (Supplementary Fig. 25). This suppression of the shuttle effect, despite enhanced convection, is attributed to the accelerated sulfur conversion kinetics at the cathode. By facilitating the rapid transformation of soluble lithium polysulfide into solid Li2S, the source of shuttle species is depleted, thereby reducing the total amount of lithium polysulfide available for migration."

8. The feasibility of integrating the ultrasonic device into practical battery systems is not discussed. Expanding on potential integration strategies would enhance the practical significance of this study.

Our response:

We thank the reviewer for this suggestion. We agree that translating the lab-scale ultrasonic

enhancement into a practical battery pack design is critical for the technology's commercial viability. While our current manuscript demonstrates the fundamental feasibility through the successful operation of a 1.1 Ah-level pouch cell and a model boat application, we acknowledge the need to explicitly outline the potential engineering strategies for system-level integration. For example, instead of the bulky probe-type sonicator used in laboratory settings, practical integration would utilize thin-film piezoelectric ceramic transducers. These planar transducers are compact, lightweight, and can be laminated directly onto the surface of pouch cells or embedded between cells in a battery module. This configuration ensures efficient acoustic coupling while minimizing the impact on the volumetric energy density of the pack. In another example, the ultrasonic effect can be integrated into modern EV battery packs employing liquid cooling systems. A synergistic design can be achieved where the liquid coolant also serves as the acoustic coupling medium. Transducers attached to the cooling plates could transmit ultrasonic waves through the coolant to the cells, simultaneously managing thermal loads and regulate the electrochemical processes without requiring complex structural redesigns. We believe these potential strategies could bridge the gap between our fundamental findings and practical application, and we have incorporated this discussion into the revised manuscript.

Revisions

Results section:

"To further enhance practical relevance, potential engineering routes for integrating ultrasonic enhancement into commercial battery systems are outlined. Rather than the bulky probe-type sonicators commonly used in laboratory studies, compact thin-film piezoelectric transducers could be laminated onto pouch-cell surfaces or embedded between cells, enabling efficient acoustic coupling with minimal impact on volumetric energy density. In addition, for electric-vehicle battery packs employing liquid cooling, the coolant itself could serve as an acoustic transmission medium, allowing ultrasonication to be delivered through cooling plates for concurrent electrochemical regulation and thermal management without significant architectural modification."

Reviewer 2 (Comments to the Author):

The purpose of this work is to induce a "high-entropy state" in the electrolyte via an external ultrasonic field, thereby enhancing convection, altering solvation structures and ionic clusters, and consequently promoting Li^+ desolvation as well as uniform transient nucleation of sulfur/lithium species, while suppressing the polysulfide shuttle and lithium dendrite growth. Compared with conventional modification strategies, this approach reduces the introduction of additional materials and associated side effects, offering relatively high novelty. However, the manuscript still suffers from insufficient experimental evidence, incomplete mechanistic justification, and inadequate assessment of the broader impacts of the external field.

Our response:

We sincerely thank the reviewer for the insightful comments and constructive suggestions. In accordance with the feedback, the manuscript has been thoroughly revised, and all requested modifications have been completed.

Specific comments:

1. The manuscript reports that the optimal electrochemical performance is achieved under 180 W ultrasonic irradiation. Has the effect of applying ultrasonic power beyond 180 W been evaluated to determine whether further improvements can be achieved, or whether detrimental side effects may occur?

Our response:

We thank the reviewer for this important question regarding the optimization of the ultrasonic parameters. To address this, we extended our evaluation to include a higher power setting of 210 W to determine if further enhancements could be realized or if the system hits a mechanical stability limit. Our experimental results indicate that 180 W represents the optimal balance point for this cell configuration. When the ultrasonic power was increased to 210 W, the electrochemical performance noticeably degraded compared to the 180 W condition (Supplementary Fig. 45). Specifically, the discharge specific capacity at 1 C dropped to 738.2 mA h g^{-1}, and the capacity decay rate increased to 0.14% per cycle over 100 cycles. This is significantly inferior to the performance at 180 W, where the cell maintained a higher capacity with a much smaller fading rate of 0.027% per cycle over 900 cycles.

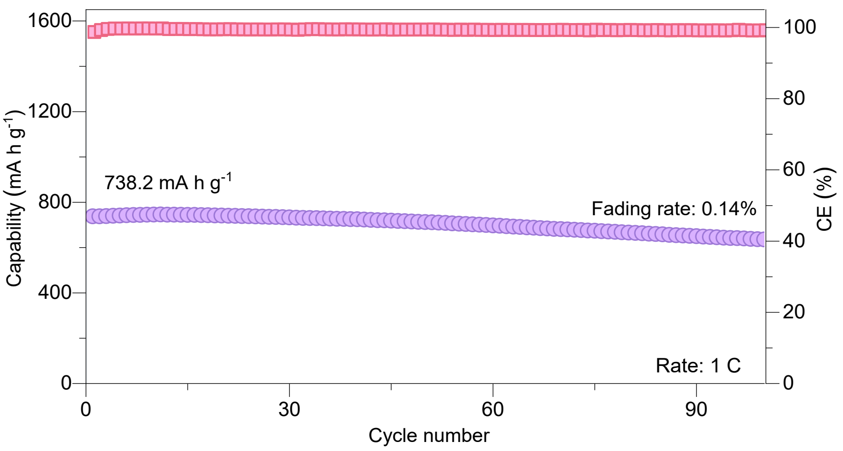

Supplementary Fig. 45|Cycling performance at 1 C under ultrasonication power of 210 W.

At higher ultrasonication power, the induced mechanical stress likely exceeds the binding strength of the electrode components, an effect that is particularly pronounced at high mass loadings, creating a challenging environment for maintaining structural integrity. This is evidenced in the comparison of digital photographs of electrode components dissembled from cycled cells. Specifically, as shown in the new Supplementary Fig. 46, the electrode cycled at 210 W (bottom-left) exhibits significant surface delamination and pulverization, evidenced by the widespread appearance of yellow patches on the black electrode surface. This suggest that the excessive shear forces from the 210 W ultrasonic field might exceed the mechanical strength of the binder, causing active material to shed and lose electrical contact. While at 180W, the mechanical stress remains within the tolerance of the electrode structure.

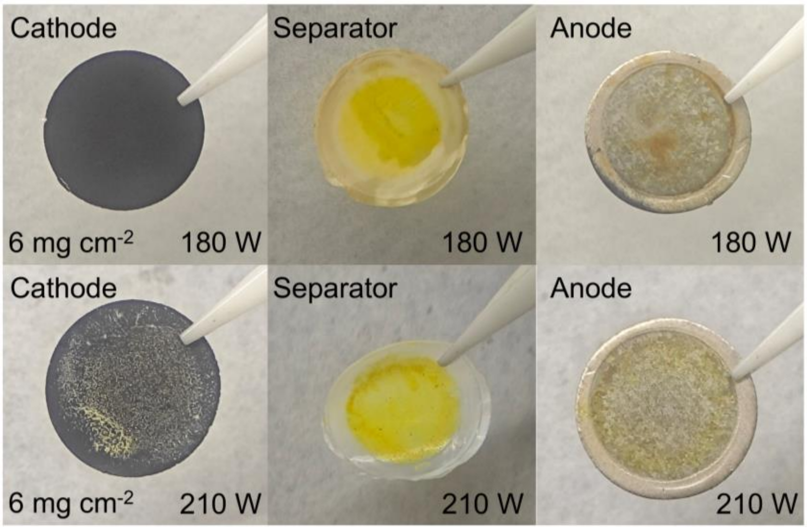

Supplementary Fig. 46|Digital photographs of disassembled cells (6 mg cm^{-2}) after 100 cycles at 0.5 C under ultrasonication at 180 W (top) and 210 W (bottom).

It's also demonstrated in our post-cycling SEM analysis (Supplementary Fig. 35, 42, 43) the sulfur cathodes and lithium anodes maintain a dense, uniform morphology after prolonged

cycling. This structural integrity is further confirmed by the stable operation of Li||Li symmetric cells for over 1000 hours and the high-capacity retention (76.5%) of full cells after 900 cycles as delineated in the manuscript. Therefore, we conclude that higher power poses a risk to the current cell design. The detailed fading mechanism at higher ultrasonic power may be more complex and warrants future investigation and optimization. Consequently, 180 W was adopted as the optimized parameter in this study to maximize electrochemical gains while ensuring long-term structural stability. We have added the associated discussion to the revised manuscript.

Revisions

Results section:

"To determine the upper limit of the beneficial acoustic field, the ultrasonic power was increased to 210 W. Experimental results indicate that 180 W represents the optimal threshold for the current cell configuration (Supplementary Fig. 45 and 46)."

Supplementary Information section:

"At 210 W, the electrochemical performance noticeably degraded, with the discharge specific capacity at 1.0 C dropping to 738.2 mA h g^{-1} and the capacity decay rate increasing significantly to 0.14% per cycle over 100 cycles (Supplementary Fig. 45). This contrast is stark compared to the 180 W condition, which maintained superior capacity and a low fading rate of 0.027% per cycle over 900 cycles."

"This performance degradation at 210 W is likely attributed to mechanical stress exceeding the binding strength of the electrode components. This is evidenced in the comparison of digital photographs of electrode components dissembled from cycled cells (Supplementary Fig. 46). Specifically, the electrode cycled at 210 W exhibits pronounced surface delamination and pulverization, accompanied by the widespread appearance of yellow patches on the black electrode surface. While at 180W, the mechanical stress remains within the tolerance of the electrode structure."

2. The authors attribute the enhanced kinetics to increased molecular disorder and entropy induced by ultrasonication. However, has the possibility been considered that such intensified molecular motion may also aggravate exothermic interfacial side reactions, potentially leading to thermal runaway risks?

Our response:

We're grateful for the reviewer's valuable comment. We agree that the introduction of external acoustic energy, which intensifies molecular motion, carries an inherent risk of thermal accumulation if left unregulated. To address this concern and validate the necessity of our safety protocols involving the use of closed-loop temperature control as mentioned in the

original submission, we conducted a detailed control experiment comparing the thermal behavior of a cell operating with our standard temperature control system against one without such regulation. In the absence of active cooling, we observed that the cell temperature gradually climbed over time (Supplementary Fig. 56), confirming that the intensified molecular motion and cavitation effects do generate accumulating heat that could eventually compromise safety. In contrast, the experimental setup utilized in this study effectively eliminates this risk through active thermal management. Our comparative data demonstrates that with this system engaged, the cell temperature is clamped at 28 °C throughout the operation. This active cooling continuously dissipates the excess heat generated by the acoustic transducer, preventing the thermal runaway.

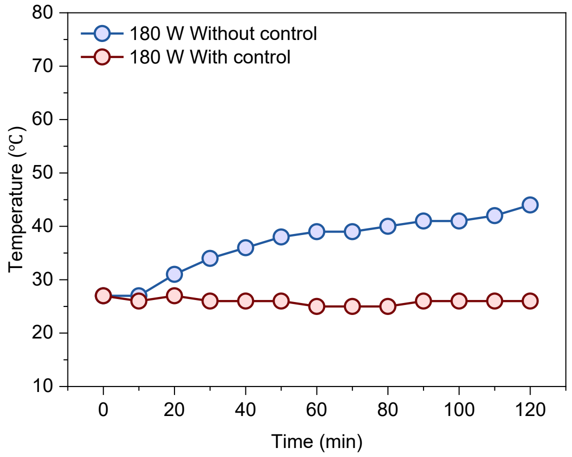

Supplementary Fig. 56|Temperature profiles of cells subjected to ultrasonication under conditions with and without temperature control.

Furthermore, it is important to note that this thermal stability is complemented by the formation of a robust interface. As detailed in our SEI analysis, the ultrasonic treatment promotes the formation of a LiF-rich passivation layer, which chemically stabilizes the electrode surface and suppresses continuous parasitic electrolyte decomposition, which is a common source of exothermic heat in failing batteries. Therefore, by combining active physical cooling with the chemical stabilization of the interface, we ensure that the enhanced kinetics are achieved safely, without increasing the risk of thermal runaway. We have added the data from this comparative temperature study to the revised manuscript.

Revisions

Methods section:

"To eliminate potential thermal runaway risks, a closed-loop control system was implemented to maintain the cell temperature at 28 °C (Supplementary Fig. 56)."

Supplementary Information section:

"To validate our safety protocols, control experiments were conducted comparing the

thermal behavior of a cell operating with active closed-loop temperature control against an unregulated system (Supplementary Fig. 56). In the absence of active regulation, the cell temperature climbed steadily over time. In contrast, with the closed-loop system engaged, the cell temperature is clamped at 28 °C throughout operation. Furthermore, thermal stability is chemically complemented by the formation of a robust interface. Ultrasonic treatment promotes the formation of a LiF-rich passivation layer, which chemically stabilizes the electrode surface and suppresses continuous parasitic electrolyte decomposition."

3. For the Ah-level Li–S pouch cell cycled at 0.5 C over 150 cycles, only a single representative voltage profile is shown. To fully demonstrate the cycling stability, discharge/charge voltage curves at different stages (initial, mid-cycle, and end-of-life) should be provided.

Our response:

We thank the reviewer for this suggestion, and we would like to offer a clarification regarding the pouch cell configurations presented in the manuscript. Two distinct pouch-cell prototypes are discussed: (i) a high-loading single-layer pouch cell (Supplementary Fig. 48, sulfur loading of 5.2 mg cm^{-2}), which was evaluated for long-term stability and cycled at 0.5 C for 150 cycles; and (ii) an Ah-level stacked pouch cell (Fig. 7h–i), ultra-high-loading 1.12-Ah configuration (9.4 mg cm^{-2} for each cathode layer) assembled to demonstrate the achievable gravimetric energy density (404.1 Wh kg^{-1}) and the scalability of the proposed technology. Based on the reviewer's specific reference to the "0.5 C over 150 cycles" condition, we understand this comment addresses the high-loading single-layer pouch cell (5.2 mg cm^{-2}). We agree that showing the evolution of voltage profiles is crucial to prove its stability. Therefore, we have extracted the charge/discharge curves for this single-layer pouch cell at the 1st (initial), 75th (mid-cycle), and 150th (end-of-life) cycles. (Supplementary Fig. 50) The comparison reveals that the voltage plateaus remain stable within 150 cycles, confirming the excellent reversibility even at a high sulfur loading, enabled by the ultrasonic field. These profiles have been added to the revised manuscript with related information discussed.

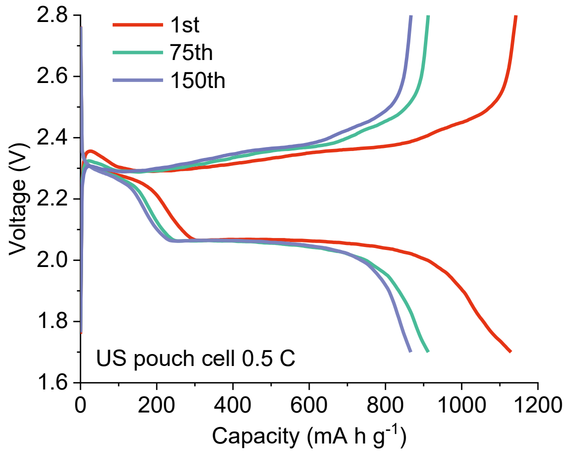

Supplementary Fig. 50|Charge–discharge profiles of the high-loading single-layer pouch cell (5.2 mg cm^{-2}) at the initial, mid-cycle, and end-of-life stages.

Revisions

Results section:

"The charge/discharge curves were also extracted for the high-loading pouch cell (5.2 mg cm^{-2}) at the 1st (initial), 75th (mid-cycle), and 150th (end-of-life) cycles (Supplementary Fig. 50). The comparison reveals that the voltage plateaus remain stable within 150 cycles, confirming the excellent reversibility even at a high sulfur loading, enabled by the ultrasonic field."

4. Direct evidence for the suppression of the polysulfide shuttle is limited. Although kinetics, impedance, and morphology data indicate improvements, more direct verification is needed. Could UV–Vis absorption spectroscopy or equivalent techniques be employed to quantitatively track the evolution of polysulfide concentration under ultrasonic conditions?

Our response:

We thank the reviewer for the valuable comment. To directly verify the implication of the ultrasonic mediation on polysulfide shuttling, operando Raman spectroscopy at the anode interface was performed. Our result indicates that the ultrasonic field effectively suppresses the shuttle effect. It is crucial to emphasize that the shuttle effect is fundamentally driven by the accumulation of soluble LiPSs that fail to convert quickly at the cathode. As demonstrated in our main text, the ultrasonic field significantly accelerates the sulfur conversion kinetics. By facilitating the rapid transformation of soluble LiPSs into solid Li_2S_4, the source of shuttle species is depleted. Therefore, the total amount of LiPSs available to migrate to the anode is reduced. To confirm this in cells under operation, we performed operando Raman spectroscopy specifically at the anode-separator interface during an LSV discharge process (0.5 mV s^{-1}). For the non-ultrasonic group, the Raman mapping reveals a rapid increase in signal intensity for

polysulfide species at the anode side as discharge progresses, confirming severe shuttling. In stark contrast, under ultrasonication, the noticeable appearance of signals corresponding to LiPSs is delayed and the intensity of these LiPS signals is markedly suppressed and remains lower throughout the discharge process. This confirms that by suppressing the accumulation of polysulfides at the cathode, the ultrasonic strategy effectively inhibits polysulfide shuttling, protecting the lithium anode. We have added these new data and discussion to the Supplementary Information and the revised manuscript.

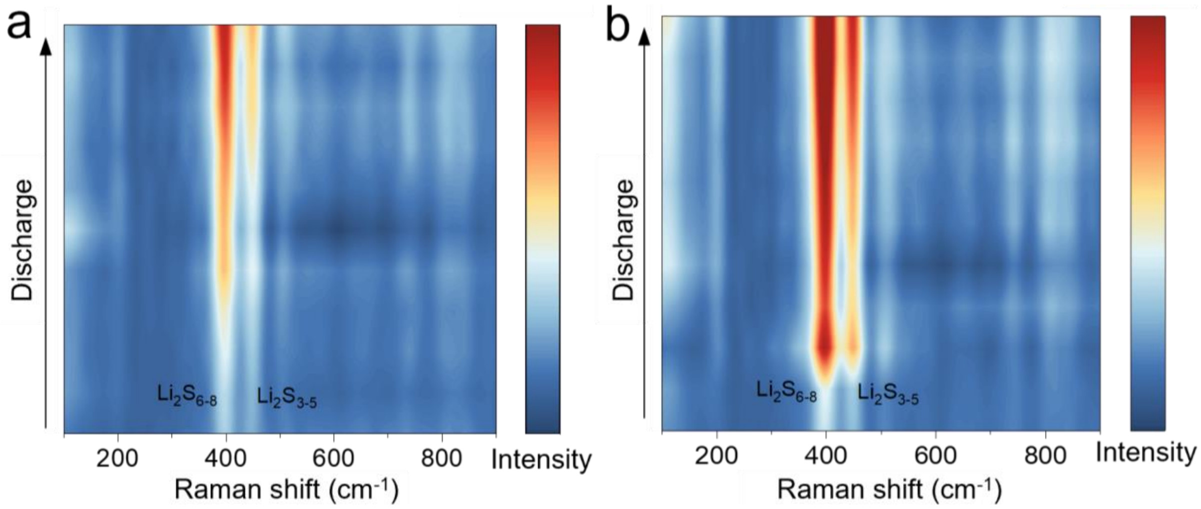

Supplementary Fig. 25|Time-resolved Raman spectra recorded at the anode/separator interface during discharge (a) with and (b) without 180 W ultrasonication.

Revisions

Results section:

"To rigorously address this, operando Raman spectroscopy was performed at the anode-separator interface during discharge. The results indicate that the ultrasonic field effectively suppresses the shuttle effect as shown in Supplementary Fig. 25."

Supplementary Information section:

"In the absence of ultrasound, Raman mapping reveals a rapid increase in signal intensity for polysulfide species at the anode side, confirming significant shuttling. In stark contrast, under 180 W ultrasonication, the appearance of lithium polysulfide signals is markedly delayed and the overall intensity remains substantially lower throughout the process (Supplementary Fig. 25). This suppression of the shuttle effect, despite enhanced convection, is attributed to the accelerated sulfur conversion kinetics at the cathode. By facilitating the rapid transformation of soluble lithium polysulfide into solid Li_2S, the source of shuttle species is depleted, thereby reducing the total amount of lithium polysulfide available for migration."

5. The manuscript only investigates the influence of ultrasonication on coin cells with low sulfur loading, while no corresponding tests were conducted on pouch cells with high sulfur loading. Since high-loading sulfur cathodes are typically thicker and more porous than low-loading ones, ultrasonic agitation may induce substantial cathode detachment in pouch cells, thereby compromising the overall electrochemical performance ?

Our response:

We greatly appreciate the reviewer's critical assessment regarding the mechanical stability of practical, high-loading electrodes. We fully agree that high-loading sulfur cathodes in pouch cells, characterized by thicker and more porous structures, are more susceptible to mechanical degradation than their low-loading counterparts. There is indeed a valid concern that the mechanical energy from ultrasonic agitation could cause active material detachment. However, we would like to clarify that our study did extend beyond low-loading coin cells to include high-loading practical devices. Specifically, the single-layer pouch cell presented in the manuscript was fabricated with a high sulfur loading of 5.2 mg cm^{-2}, significantly higher than the 1.8–2.0 mg cm^{-2} used in the coin cell tests. This high-loading cell was tested directly under the 180 W ultrasonic field and demonstrated stable cycling performance for 150 cycles with a low-capacity decay rate of 0.15% per cycle (Supplementary Fig. 48). Furthermore, the Ah-level stacked pouch cell, which utilized a total sulfur loading of 1.05 g, also operated successfully under ultrasonication. The robust electrochemical performance of these high-loading devices serves as strong evidence that the electrode integrity was maintained. To provide direct visual confirmation of this structural integrity, we followed the reviewer's suggestion and disassembled the high-loading pouch cell (5.2 mg cm^{-2}) after cycling to inspect the cathode interface. As shown in the newly added Supplementary Fig. 51, the digital photograph of the harvested cathode reveals a uniform surface with no signs of detachment. This post-mortem analysis confirms that the optimized ultrasonic power (180 W) provides sufficient enhancement without exceeding the mechanical yield strength of the thick, porous cathode structure. We have updated the manuscript to explicitly state the loading parameters of the pouch cells mediated by ultrasonication and included the related observations to substantiate their stability.

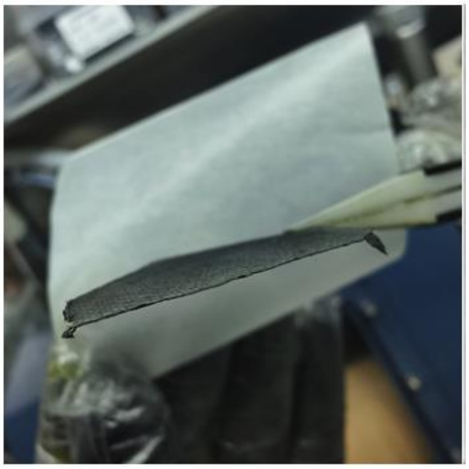

Supplementary Fig. 51|Tilted-angle digital photographs examining the surface condition and adhesion to the current collector of the cathode harvested from a high-loading pouch cell (5.2 mg cm^{-2}) after cycling under ultrasonication at 180 W.

Revisions

Supplementary Information section:
"The robust electrochemical performance of the high-loading pouch cell provides strong evidence that electrode integrity was maintained. Visual confirmation of the structural integrity of the high-loading pouch cell cathode after 150 cycles under ultrasonication at 180 W was also conducted (Supplementary Fig. 51). The digital photograph of the harvested cathode reveals a uniform surface with no signs of detachment, confirming that the optimized ultrasonic power (180 W) delivers sufficient enhancement without exceeding the mechanical yield strength of the thick, porous cathode structure."

6. There appears to be some ambiguity regarding whether ultrasonication is applied in situ during cell operation or ex situ as a pre-treatment of the electrolyte. For clarity, it is recommended to explicitly differentiate which experiments involved continuous ultrasonic irradiation during cycling and which involved only pre-sonicated electrolytes. A comparison between the two conditions, including reversibility after removing the external field, would strengthen the conclusions ?

Our response:

We appreciate the reviewer's request for clarification regarding the operational mode of the ultrasonic field. We apologize for any ambiguity in the original text. We confirm that all electrochemical performance data reported in this manuscript (including CV, galvanostatic cycling, and rate performance) were obtained under in situ, continuous ultrasonic irradiation during cell operation, rather than using ex situ pre-treated electrolytes. The fundamental

mechanism proposed in our work relies on transient cavitation. This is a dynamic process that exists only while the external acoustic field is applied. Once the ultrasonic field is removed, the cavitation bubbles collapse, and the fluid motion ceases, causing the electrolyte to relax back to its pristine state. To further substantiate this statement, a control experiment was conducted in which a cell (with an identical configuration to the coin cell in the manuscript) was pre-sonicated at 180 W for 1 h and subsequently tested without ultrasonication. As shown in Supplementary Fig. 18, the performance of the pre-sonicated cell is very similar to that obtained at 0 W (i.e., without sonication), thereby confirming the necessity of performing ultrasonic treatment in situ. The impact of the acoustic field is dynamic rather than permanently encoded in the material.

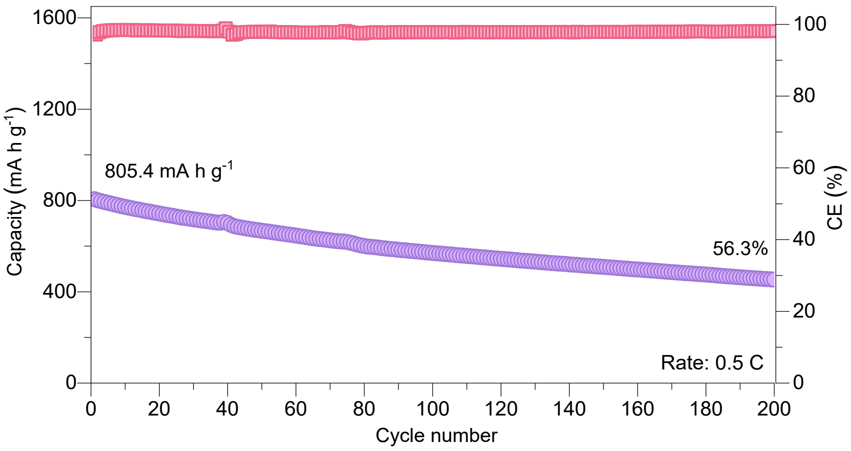

Supplementary Fig. 18|Cycling performance over 100 cycles at 0.5 C of a cell pre-sonicated at 180 W for 1 h and subsequently tested without ultrasonication.

To further address the reviewer's question regarding reversibility, the cell performance was examined sequentially under ultrasonication, without ultrasonication, and then under ultrasonication again, and the results were compared (Supplementary Fig. 19). As expected from the mechanism described above, removal of the external field resulted in an immediate performance decline, while reapplication of ultrasonication promptly restored the performance in terms of capacity and polarization, thereby confirming the reversibility of the process. The minor unrecovered capacity is likely attributed to the negative impact of the cycling history during the second stage (conducted without ultrasonication). This clear "on/off" dependence confirms the reversibility of this technique and further validates the necessity of in situ ultrasonication. We have updated the manuscript to explicitly state that the ultrasonic device was active in situ during the electrochemical measurements and to discuss this reversible, on-demand nature of the enhancement.

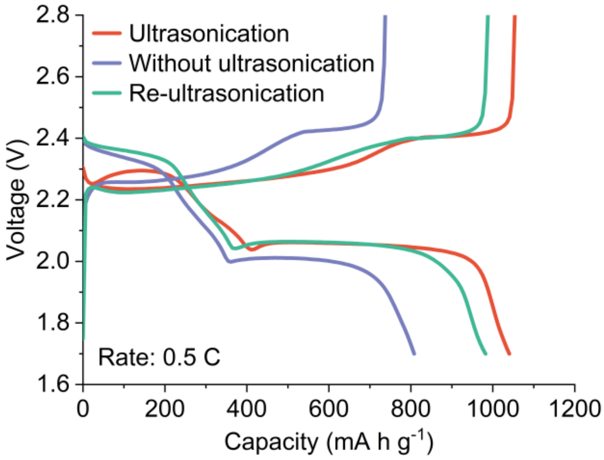

Supplementary Fig. 19|Charge-discharge profiles at 0.5 C of a cell subjected sequentially to ultrasonication, without ultrasonication, and re-ultrasonication.

Revisions

Results section:

"The external acoustic field is applied in situ throughout the duration of the electrochemical measurements to maintain the transient cavitation state, rather than serving as a pre-treatment for the electrolyte. The enhancement provided by the acoustic field is highly dynamic and reversible, as demonstrated in Supplementary Fig. 18 and 19."

Supplementary Information section:

"As shown in Supplementary Fig. 18, the performance of the pre-sonicated cell is very similar to that obtained at 0 W *(i.e.*, without sonication), confirming that the impact of the acoustic field is dynamic rather than permanently encoded in the material."

"The beneficial effects on capacity and polarization are only sustained during active sonication; removal of the field leads to an immediate performance decline, which can be promptly restored upon re-application (Supplementary Fig. 19). This 'on/off' dependence confirms that the enhancement is a transient reversible phenomenon governed by active cavitation."

7. The XPS results indicate LiF enrichment in the SEI, which is attributed to modified desolvation and TFSI$^-$ decomposition. However, the contribution from $LiNO_3$ depletion and related side reactions is not distinguished. Advanced characterization such as in situ/operando XPS, depth-profiling TOF-SIMS, or GC-MS/NMR of electrolyte decomposition products would be useful to separate the respective roles of $LiNO_3$ and TFSI.

Our response:

We sincerely appreciate the reviewer's rigorous attention to the SEI formation mechanism and the potential confounding factors introduced by the additives. We agree that decoupling the

contribution of $LiNO_3$ depletion from LiTFSI decomposition is essential to validate our proposed mechanism. Fundamentally, the formation of LiF requires a fluorine source. In our electrolyte system, the only source of fluorine is the $TFSI^-$ anion. Therefore, the significant enrichment of LiF observed under ultrasonic conditions serves as a specific chemical marker for the decomposition of the $TFSI^-$ anion. To experimentally verify that this process is independent of $LiNO_3$ depletion, we performed depth-profiling XPS analysis on lithium anodes cycled in electrolytes with varying $LiNO_3$ concentrations (0.5 wt% vs. 2.0 wt%). As presented in the newly added Supplementary Fig. 31, we analyzed the Li 1s spectra of the inner SEI after ion etching for 10 nm. The results reveal that, despite the four-fold difference in $LiNO_3$ concentration, the atomic content of inorganic LiF in the dense inner SEI remained remarkably consistent, approximately 22.3 at% for the 0.5 wt% $LiNO_3$ group and 23.5 at% for the 2.0 wt% $LiNO_3$ group. This consistency demonstrates that the formation of the LiF-rich interphase is independent of the $LiNO_3$ concentration. Instead, it confirms that LiF generation is governed by the decomposition of the bulk LiTFSI salt, a process facilitated by the ultrasonic desolvation effect we proposed. This effectively decouples the enhanced LiF formation from the depletion dynamics of the $LiNO_3$ additive. We deeply appreciate the reviewer's insight that the influence of the ultrasonic field on $LiNO_3$ and its subsequent impact on the fine structure of the SEI is a complex and scientifically intriguing topic. We agree that the specific interactions between acoustic cavitation and nitrate additives warrant further investigation. Future studies employing operando XPS, depth-profiling TOF-SIMS for chemical mapping, and GC-MS for decomposition product analysis will be instrumental in fully elucidating the mechanism of ultrasound-mediated additive decomposition. For the purpose of this study, however, the current experimental evidence sufficiently confirms that the beneficial LiF-rich interphase is derived from the ultrasound-promoted decomposition of $TFSI^-$. The related discussions have been updated in the revised manuscript.

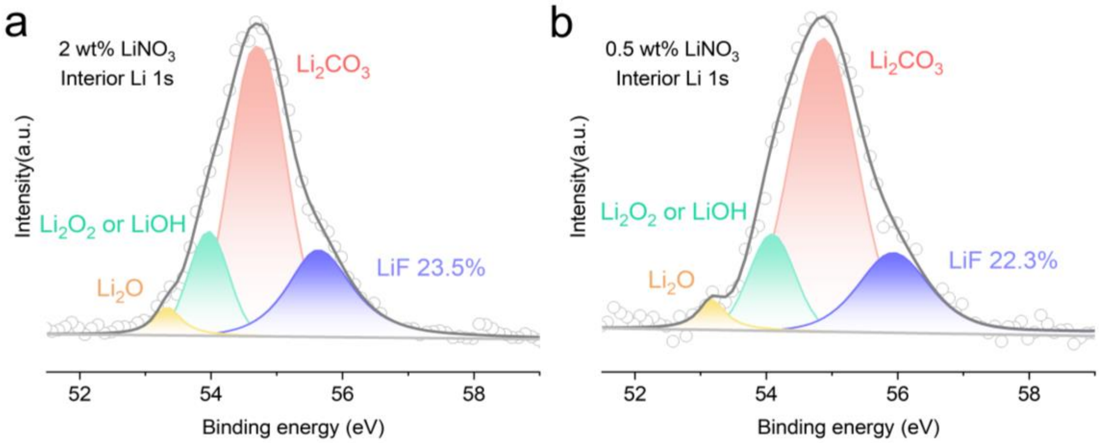

Supplementary Fig. 31|Depth-profiling XPS analysis of Li 1s spectra was used to evaluate the effect of (a) 2 wt% and (b) 0.5 wt% $LiNO_3$ concentration on LiF content within the inner SEI. The samples were obtained from ultrasonication mediated Li//Li symmetric cells cycled 100 times at 2 mA cm^{-2} and 2 mA h cm^{-2}.

Revisions

Results section:

"To decouple the respective contributions of $LiNO_3$ depletion and LiTFSI decomposition to SEI formation, the SEI compositions of lithium anodes cycled with different $LiNO_3$ concentrations (0.5 wt% and 2.0 wt%) were analyzed. As shown in Supplementary Fig. 31, depth-profiling XPS analysis after ~10 nm of etching reveals that the LiF content in the dense inner SEI remains essentially unchanged (22.3 at% vs. 23.5 at%) despite the substantial difference in $LiNO_3$ concentration. These results indicate that the formation of the LiF-rich interphase is predominantly governed by ultrasound-mediated desolvation and subsequent decomposition of the bulk LiTFSI salt, thereby effectively decoupling this process from the $LiNO_3$ additive."

8. Given the application of an external ultrasonic field, it is necessary to reassess the device performance across a broader range of cathode areal loadings and electrolyte-to-sulfur (E/S) ratios. The potential effects on cathode integrity, separator stability, anode morphology, and current collector adhesion under varying structural conditions should be systematically evaluated.

Our response:

We sincerely appreciate the reviewer's suggestion. We agree that variations in cathode loading and E/S ratios may alter the mechanical stress condition within the cells. To systematically address this concern, we expanded our experimental matrix to include three distinct cell configurations cycled for 100 cycles under the optimized 180 W ultrasonic field: (1) the baseline condition (2 mg cm^{-2}, E/S = 15 μL mg^{-1}), (2) a lean-electrolyte condition (2 mg cm^{-2}, E/S = 6 μL mg^{-1}), and (3) a practical high-loading, lean-electrolyte condition (6 mg cm^{-2}, E/S = 6 μL mg^{-1}). Following the cycling test, these cells were disassembled to visually inspect the structural integrity of the cathode, separator, anode, and the adhesion quality of the current collectors. As demonstrated in the newly added Supplementary Fig. 47, the post-mortem analysis confirms that the device maintains excellent structural integrity across all tested parameter combinations. Even under the most rigorous condition of high loading (6 mg cm^{-2}) paired with lean electrolyte (E/S = 6 μL mg^{-1}), we observed no signs of active material delamination or detachment from the current collector, confirming robust adhesion. Furthermore, the separators exhibited no mechanical damage, and the lithium anodes are free of pulverization. These results validate that the 180 W ultrasonic field is mechanically compatible with a wide range of cell configurations, effectively preserving component integrity even under stringent practical conditions. The related information and discussions have been added to the revised manuscript.

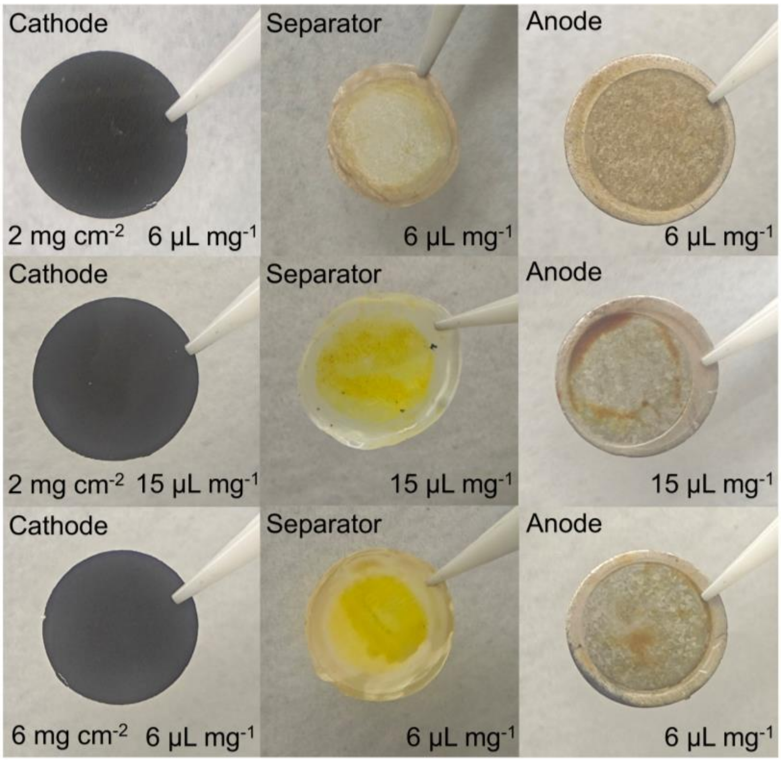

Supplementary Fig. 47|Examination of cell components harvested from disassembled cells after 100 cycles at 0.5 C, 180 W ultrasonication under different conditions: 2.0 mg cm^{-2}, E/S = 6 μL mg^{-1}; 2.0 mg cm^{-2}, E/S = 15 μL mg^{-1}; and 6.0 mg cm^{-2}, E/S = 6 μL mg^{-1}.

Revisions

Results section:

"To assess the mechanical robustness of the battery architecture under the 180 W ultrasonic field, devices across a range of cathode areal loadings and E/S ratios were systematically evaluated as shown in Supplementary Fig. 47."

Supplementary Information section:

"The post-mortem analysis confirms that the devices maintain excellent structural integrity across all tested parameter combinations (Supplementary Fig. 47). Even under the most rigorous condition, high mass loading (6.0 mg cm^{-2}) combined with a lean electrolyte (E/S = 6 μL mg^{-1}), no evidence of active-material delamination or detachment from the current collector was observed. Furthermore, the separators exhibited no mechanical damage, and the lithium anodes showed no signs of pulverization, demonstrating that the 180 W ultrasonic field is mechanically compatible with a wide range of cell configurations."

Reviewer 3 (Comments to the Author):

In this study, the authors present an exogenous modification approach to Li–S battery electrolytes by applying ultrasound. They propose that ultrasound induces bubble nucleation and collapse, which in turn generates a "high entropy" electrolyte environment that improves cell performance. A series of electrochemical and structural characterizations are provided to support this claim and to demonstrate performance stabilization.

However, the manuscript contains several questionable claims, flawed arguments, and inconsistencies. In particular, the central assertion that ultrasound can induce "high entropy" in the electrolyte is problematic and not supported by thermodynamic reasoning. The reviewer does not agree with this interpretation. Specific objections and concerns are detailed below:

Our response:

We sincerely appreciate the reviewer's professional insights and constructive feedback. Based on these comments, the manuscript has been carefully re-examined and revised to improve its quality, and all requested changes have been fully addressed.

Specific comments:

1. The first objection is in the use of the term "high entropy". This term is often misused in battery related literature. High entropy generally refers to enhanced entropy of mixing due to the presence of multiple compositional constituents. Cavitation may enhance the mixing, solvation kinetics, dissolution, and homogenization but it does not fundamentally change the intrinsic entropy of the electrolyte, the latter being more chemical in nature. From thermodynamic point of view, what the authors claim as high entropy is not actually high entropy. The use of the term "high entropy" will mislead the readers.

Our response:

We sincerely appreciate the reviewer's rigorous attention to thermodynamic terminology and share the concern regarding the precise usage of scientific definitions. We fully acknowledge that within the established conventions of materials science, specifically in the fields of high-entropy alloys and high-entropy oxides, the term "high entropy" strictly describes systems stabilized by high configurational entropy arising from compositional complexity. We agree that our electrolyte formulation, in its static state, does not meet this specific "compositional" criterion. However, we respectfully submit that our usage does not refer to a static chemical formulation but rather to a dynamic, non-equilibrium modification induced by the applied acoustic field. This interpretation is supported by well-established theoretical frameworks as

well as by experimental evidence. The reviewer argues that cavitation does not alter the "intrinsic" chemical entropy. We respectfully contend that, this argument overlooks the effect of ultrasonication on the solvation-shell structure. The solvation shell constitutes an essential component of the intrinsic chemical identity of ions in solution, and entropy has been explicitly proposed as a useful metric for analyzing specific solvation identity in liquid electrolytes (*Energy Environ. Sci.,* **2024**, *17*, 2406–2430). Our ab initio molecular dynamics simulations, together with operando Raman spectroscopy, demonstrate that the acoustic field disrupts ion solvation shells and liberates coordinated solvent molecules. This transition from an ordered, solvated state to a more disordered, partially desolvated state provides strong evidence for an entropy increase. This is consistent with established theoretical descriptions (*J. Chem. Phys.,* **1945**, *13, 11*, 507–532) and classical thermodynamics, which links macroscopic entropy with the most probable microscopic configuration (*Atkins' physical chemistry*. Oxford university press, **2023**.). In addition, the disordered state is functionally equivalent to the "weakened interactions" observed in compositionally complex high-entropy electrolytes with elevated configurational entropy (*Nat. Energy,* **2023**, *8*, 814–826). Furthermore, according to classical thermodynamics (*Phys. Rev. A,* **1977**, *15, 6,* 2545–2549) and emerging theoretical frameworks in battery research (*Energy Environ. Sci.,* **2024**, *17*, 2406–2430), this entropy increase associated with solvation-state modification corresponds to an increase in "excess entropy" (*J. Chem. Phys.,* **2018**, *149*, 210901; *Nat. Commun.,* **2023**, *14*, 440), a thermodynamic quantity that captures the degree to which the disorder of a real mixture deviates from that of an ideal mixture. In this context, the acoustic-field-induced disruption of solvation structures represents a physically meaningful increase in entropy, despite the absence of compositional changes. We emphasize that this "high-entropy" state is inherently dynamic and non-equilibrium, fundamentally distinct from conventional static configurational entropy defined by material composition. To experimentally validate this distinction, a control experiment was conducted in which a cell was pre-sonicated at 180 W for 1 h and subsequently tested without ultrasonication. As shown in Supplementary Fig. 18, the performance of the pre-sonicated cell closely resembles that obtained at 0 W (*i.e.*, without sonication), confirming that the impact of the acoustic field is dynamic rather than permanently encoded in the material.

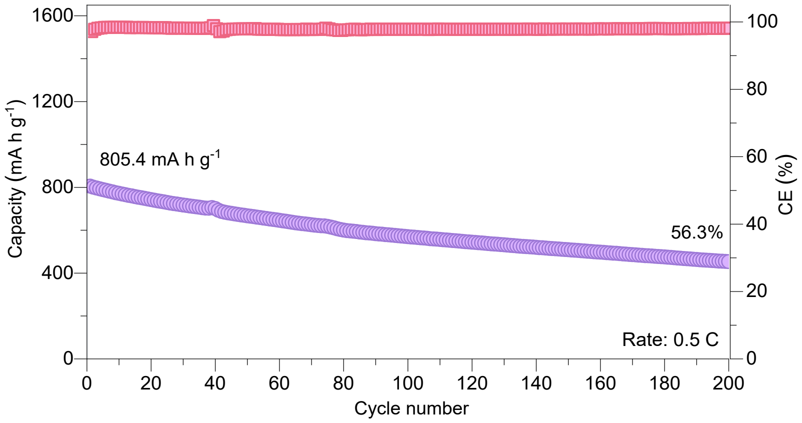

Supplementary Fig. 18|Cycling performance over 200 cycles at 0.5 C of a cell pre-sonicated at 180 W for 1 h and subsequently tested without ultrasonication.

In addition, of particular relevance to this discussion is a recent publication by Litman and Michaelides, titled "Entropy Governs the Structure and Reactivity of Water Dissociation Under Electric Fields" (*J. Am. Chem. Soc.*, **2025**, *147*, 44885–44894). This work, submitted concurrently with our submission (July of 2025), explores the same fundamental principle of utilizing exogenous fields to govern chemical reactions through entropic modulation. The authors demonstrate that the application of an exogenous electric field, without any modification to the chemical composition of the aqueous system, transforms the water dissociation reaction from an "entropically hindered" process to an "entropy-driven" one. Through comprehensive theoretical frameworks, it was proven that the electric field randomizes molecular orientations during proton transfer events, which weakens the existing hydrogen-bond network and drives the system toward a more disordered, higher-entropy state. This field-induced, large positive reaction entropy is responsible for dramatically enhancing the water dissociation reaction and increasing the equilibrium constant by several orders of magnitude. In essence, the findings from this study are in perfect alignment with our claim that an exogenous acoustic field can dynamically alter the electrolyte microenvironment. Just as the electric field in the study (*J. Am. Chem. Soc.*, **2025**, *147*, 44885–44894) disrupts hydrogen bonding to increase entropy, our acoustic cavitation field utilizes intense localized energy dispersal to disrupt the Li-ion solvation structure, creating a more disordered, desolvated state. This confirms that "high entropy state" is a valid and accurate description of this dynamic external field-driven system. Based on the above discussions, we respectfully submit that the acoustic-field-induced dynamic disorder in the electrolyte, accompanied by modified solvation structures, can be reasonably described as a high-entropy state. Nevertheless, to avoid misleading readers who may expect a static multi-salt formulation, we have adopted a more precise terminology throughout the revised manuscript. We now use the term "acoustic-induced entropy-driven electrolyte" or ‘electrolyte in an acoustic-induced entropy-driven state’ to emphasize that the entropy increase arises from a dynamic condition rather than from the static material composition, and to highlight its beneficial, entropy-driven impact on Li–S chemistry. We believe this distinction respects the established conventions of the field while accurately describing the novel non-equilibrium field-induced modification in this study. The related discussions have been incorporated into the revised manuscript.

Revisions

Introduction section:

“It is important to distinguish this from conventional high-entropy systems, where entropy increases through static configurational mixing of multiple chemical constituent.[28–30] In our system, the entropy-driven state is a dynamic, non-equilibrium phenomenon.[31] The acoustic

field facilitates energy dispersal that disrupts the ordered Li-ion solvation shells, transitioning the electrolyte from a localized ordered state to a disordered, partially desolvated state. This modification constitutes an increase in excess entropy,[32–35] characterizing the degree of disorder induced by the exogenous field rather than compositional complexity. This entropy-mediated regulation is expected to fundamentally impact the electrochemical dynamics."

Results section:

"These findings validate that the intense, localized shear forces originating from cavitation profoundly alter the microscopic structure of the electrolyte. According to established theoretical frameworks, the liberation of coordinated solvent molecules into the bulk electrolyte increases the system's disorder.[41] This field-induced entropy increase is functionally equivalent to the 'weakened interactions' often sought in static high-entropy electrolytes but is achieved here through exogenous energy dispersal.[42]"

Supplementary Information section:

"As shown in Supplementary Fig. 18, the performance of the pre-sonicated cell is very similar to that obtained at 0 W (*i.e.*, without sonication), confirming that the impact of the acoustic field is dynamic rather than permanently encoded in the material."

In addition, throughout the manuscript and Supplementary Information, the original phrasing of 'high-entropy electrolyte' has been replaced with 'acoustic-induced entropy-driven electrolyte' or 'electrolyte in an acoustic-induced entropy-driven state,' as appropriate, to emphasize that the entropy increase arises from a dynamic condition rather than from the static material composition, and to highlight its beneficial, entropy-driven impact on Li–S chemistry.

2. The authors state that "An ultrasonic field induces highly localized and chaotic effects within the electrolyte, disturbing the equilibrium and promoting a state of high entropy". The reviewer cannot agree with this statement either. The term "entropy" from thermodynamics point of view is tied to composition, temperature, and energy distribution. One cannot correlate the local chaotic effect induced by transient fluctuations to a bulk state of higher entropy. The latter exists with or without the exogenous effect. In electrolyte chemistry, the term "high entropy" has well established meaning. The manner in which this term is used here is not responsible and does not correlate to thermodynamic principles.

Our response:

We sincerely thank the reviewer for this rigorous thermodynamic assessment. We fully accept the criticism that our original phrasing, which linked "local chaotic effects" directly to a "bulk state of high entropy," was potentially misleading. We apologize for not clearly articulating the

underlying physicochemical mechanism. We agree that transient physical fluctuations alone do not constitute a high-entropy state. The actual entropy increase is rooted in a fundamental modification of the electrolyte's solvation structure, which represents the intrinsic chemical identity of ions in solution. As detailed in our response to Comment 1, mediated by the acoustic field, the transition from a highly ordered, tightly coordinated state to a disordered, partially desolvated state results in an increase in excess entropy. Therefore, the acoustic field does not merely create "chaos"; it drives a structural shift that increases the disorder of the solvation environment. This effect is functionally equivalent to increased configurational entropy observed in compositionally complex high-entropy electrolytes. We have now articulated in the revised manuscript that the entropy increase arises from changes in chemical identity rooted in the modification of solvation structures, rather than from the acoustic field itself merely inducing local chaotic disturbances. In addition, in alignment with our response to Comment 1, we have consistently adopted the term 'Acoustic-induced entropy-driven electrolyte' or 'electrolyte in an acoustic-induced entropy-driven state' throughout the revised manuscript.

Revisions

Results section:

"The application of an ultrasonic field promotes a dynamic, acoustic-induced entropy-driven state. This state is not merely a product of local chaotic disturbances, but is rooted in the fundamental modification of the electrolyte's solvation structure."

In addition, consistent with our response to Comment 1, the original phrasing of 'high-entropy electrolyte' has been replaced throughout the manuscript and Supplementary Information with 'acoustic-induced entropy-driven electrolyte' or 'electrolyte in an acoustic-induced entropy-driven state,' as appropriate.

3. A rotational mass transfer model was used to explain the mass transport and electrochemical reaction kinetics. How did the authors make sure that the rotational mass transfer model be effectively predicting the mass transport in such system? This seems like an over simplification because such model will not account for the spatial heterogeneity because of the random nature of the bubble formation and collapse.

Our response:

We thank the reviewer for the insightful comment regarding the spatial heterogeneity and complexity of bubble dynamics. We agree that the formation and collapse of cavitation bubbles are central to how acoustic effects influence electrochemical mass transfer. To clarify, the "rotational trajectories" described in the manuscript are not an a priori simplification, but rather an emergent phenomenon derived from a rigorous dispersed two-phase flow algorithm. We

would like to mention that, in the field of computational fluid dynamics, algorithms for simulating gas-liquid two-phase flows generally fall into two categories: separated and dispersed two-phase flow models, which are selected based on the scale difference between the bubble size and the computational domain. A separated two-phase flow approach is typically adopted when the bubble size is comparable to the characteristic scale of the model, allowing for detailed tracking of the shape and evolution of individual bubble interfaces (Supplementary Fig. 57). However, when there is a significant scale disparity, resolving each individual bubble interface not only demands substantial computational resources and time but also leads to convergence difficulties. In such cases, a dispersed two-phase flow model is more suitable (Supplementary Fig. 57), describing the gas and liquid phases through field variables ($0 < \varphi < 1$). Given the extreme scale disparity between the microscopic cavitation bubbles and the macroscopic electrolyte domain, tracking individual bubble interfaces would be computationally prohibitive and often fails to converge. Instead, our dispersed two-phase flow model incorporates the physical effects of bubbles through coupled source terms, including surface tension, buoyancy, and mass transfer. These terms mathematically represent the energy dispersal and localized turbulence generated by the cavitation field. Our simulations reveal that these coupled source terms induce toroidal convection vortices within the electrolyte. These vortices, in turn, drive the rotational mass transfer trajectories of Li-ions observed in our simulations, effectively bridging the microscale randomness of bubble collapse with organized macroscopic ion transport. By coupling these bubble-driven flow fields, our model effectively captures how cavitation manifests as enhanced mass transfer at the macroscale. We have added a more detailed description of the dispersed two-phase algorithm in the revised Supporting Information to further clarify these mechanisms.

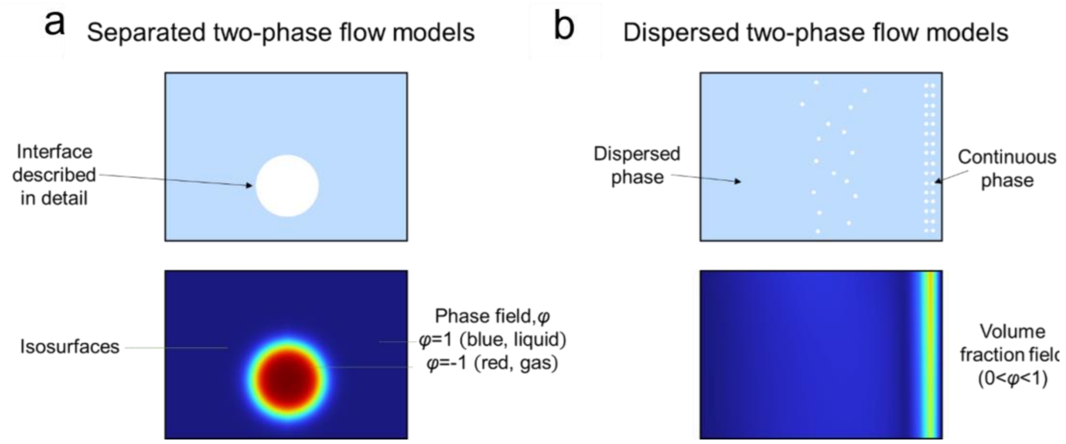

Supplementary Fig. 57|Schematic diagram for (a) separated two-phase flow models and (b) dispersed two-phase flow models.

Revisions

**Methods section:**

"The computational domain was modeled as a cylinder, geometrically representing the electrolyte confined between the anode at the upper boundary and the cathode at the lower boundary. To account for the scale disparity between the microscopic cavitation bubbles and the overall domain, a dispersed two-phase flow model was employed (Supplementary Fig. 57)."

Supplementary Information section:

"The choice between separated and dispersed two-phase flow models is governed by the relative length scales of the bubbles and the computational domain. A separated two-phase flow model is employed when the bubble size is comparable to the model scale, enabling explicit tracking of individual bubble interfaces (Supplementary Fig. 57a). In contrast, a dispersed two-phase flow model is adopted here due to the extreme scale disparity between the cavitation bubbles and the simulation domain, wherein the gas and liquid phases are represented by field variables ($0 < \varphi < 1$) (Supplementary Fig. 57b). This framework accounts for interfacial effects, such as surface tension, buoyancy, and mass transfer, through coupled source terms. These source terms mathematically describe the energy dispersal from the cavitation field, which drives the formation of toroidal vortices and governs the macroscopic rotational trajectories of reactive species."

4. The authors mention that they expect higher ionic mobility in the presence of ultrasound. Have they measured the ionic conductivity to support this claim?

Our response:

We thank the reviewer for this valuable suggestion. We agree that while our COMSOL and AIMD simulations strongly suggest enhanced Li-ion mobility, experimental validation is crucial to substantiate this claim. To address this, we conducted electrochemical impedance spectroscopy measurements to quantify the ionic conductivity (which is proportional to ion mobility) of the electrolyte under different ultrasonic power conditions (0 W, 90 W, and 180 W). The ionic conductivity σ was calculated using the equation $\sigma = L/R_bA$, where L is the distance between electrodes, A is the area, and R_b is the bulk resistance derived from the high-frequency intercept of the Nyquist plot. Our results show a clear positive correlation between ultrasonic power and ionic conductivity. Specifically, the conductivity increased from 0.187 mS cm^{-1} in the pristine state (0 W) to 0.250 mS cm^{-1} at 90 W, and further to 0.361 mS cm^{-1} under 180 W excitation. These findings align with our computational models, experimentally validating the mechanism of acoustic-driven mobility enhancement proposed. We have added these experimental data and the corresponding discussion to the revised manuscript.

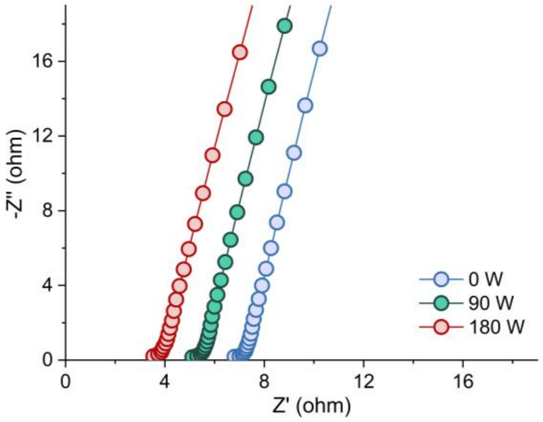

Supplementary Fig. 9|Electrolyte ionic conductivity under ultrasonication at different power levels evaluated by EIS in symmetric cells.

Revisions

Results section:

"To experimentally validate the Li^+ mobility enhancement suggested by the computational results, the electrolyte ionic conductivity (σ) was quantified under varying ultrasonic power levels using electrochemical impedance spectroscopy. As shown in Supplementary Fig. 9, a clear positive correlation between acoustic power and ion transport was observed, with ionic conductivity increasing progressively as the ultrasonic power is raised."

Supplementary Information section:

"The ionic conductivity σ was calculated using the equation $\sigma = L/R_bA$, where L is the distance between electrodes, A is the area, and R_b is the bulk resistance derived from the high-frequency intercept of the Nyquist plot. The conductivity increased from 0.187 mS cm^{-1} in the pristine state (0 W) to 0.250 mS cm^{-1} at 90 W, and further to 0.361 mS cm^{-1} under 180 W excitation."

5. What is the equivalent circuit used for fitting? is the EIS measured on the same cell before and after ultrasound?

Our response:

We appreciate the reviewer's attention to the specific details of our electrochemical impedance spectroscopy (EIS) analysis. Regarding the fitting model, we employed a standard Randles-type equivalent circuit, which has been illustrated in the newly added Supplementary Fig. 20 for clarity. The circuit consists of an ohmic resistance (R_{SOL}) connected in series with a parallel combination of a constant phase element (CPE) and a kinetic branch. The kinetic branch comprises the charge transfer resistance (R_{CT}) in series with a Warburg impedance (W). The standard circuit model provides an excellent fit to the experimental Nyquist plots, allowing us

to accurately extract the quantitative reduction in R_{CT} driven by the ultrasonic field. We verify that the EIS measurements were performed on the same individual cell under varied acoustic field strengths. This measurement protocol was chosen specifically to eliminate cell-to-cell variability. The related information has been added to the revised manuscript.

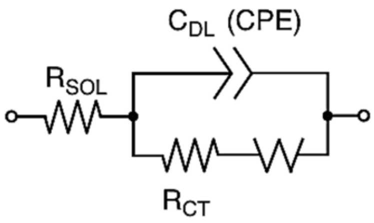

Supplementary Fig. 20|An equivalent circuit for fitting the R_{ct} values of EIS spectrum.

Revisions

Results section:

"Electrochemical impedance spectroscopy (EIS) was performed on Li–S batteries to elucidate the influence of the ultrasonic field on their charge-transport properties, with measurements conducted on the same cell under varying ultrasonic power levels (Fig. 4g). The impedance spectra were fitted using a standard Randles-type equivalent circuit (Supplementary Fig. 20)."

Supplementary Information section:

"The circuit consists of an ohmic resistance (R_{SOL}) connected in series with a parallel combination of a constant phase element (CPE) and a kinetic branch. The kinetic branch comprises the charge transfer resistance (R_{CT}) in series with a Warburg impedance (W). The standard circuit model provides an excellent fit to the experimental Nyquist plots, allowing us to accurately extract the quantitative reduction in RCT driven by the ultrasonic field."

6. Is the ultrasonication power optimized? They show improved performance with increasing ultrasonication power from 0 to 90 to 180. Best performance is achieved at 180. What would happen if they go higher? Will the performance keep on improving?

Our response:

We thank the reviewer for this important question regarding the optimization of the ultrasonic parameters. To address this, we extended our evaluation to include a higher power setting of 210 W to determine if further enhancements could be realized or if the system hits a mechanical stability limit. Our experimental results indicate that 180 W represents the optimal balance point for this cell configuration. When the ultrasonic power was increased to 210 W, the electrochemical performance noticeably degraded compared to the 180 W condition. Specifically, the discharge specific capacity at 1 C dropped to 738.2 mA h g^{-1}, and the capacity

decay rate increased to 0.14% per cycle over 100 cycles. This is significantly inferior to the performance at 180 W, where the cell maintained a higher capacity with a much smaller fading rate of 0.027% per cycle over 900 cycles.

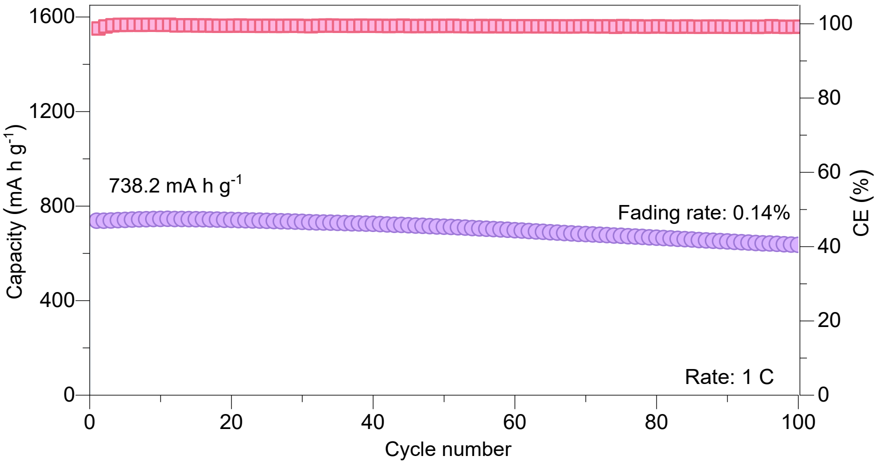

Supplementary Fig. 45|The cycling performance at 1 C under 210 W.

At higher ultrasonication power, the induced mechanical stress likely exceeds the binding strength of the electrode components, an effect that is particularly pronounced at high mass loadings, creating a challenging environment for maintaining structural integrity. This is evidenced in the comparison of digital photographs of electrode components dissembled from cycled cells. Specifically, as shown in new Supplementary Fig. 46, the electrode cycled at 210 W (bottom-left) exhibits significant surface delamination and pulverization, evidenced by the widespread appearance of yellow patches on the black electrode surface. This suggest that the excessive shear forces from the 210 W ultrasonic field might exceed the mechanical strength of the binder, causing active material to shed and lose electrical contact. While at 180 W, the mechanical stress remains within the tolerance of the electrode structure.

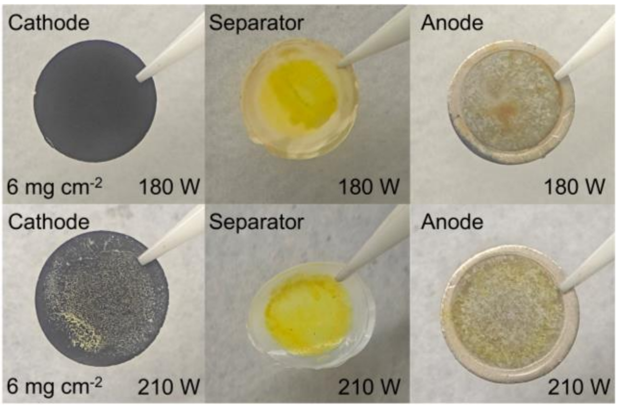

Supplementary Fig. 46| Digital photographs of disassembled cells (6 mg cm^{-2}) after 100 cycles at 0.5 C under ultrasonication at 180 W (top) and 210 W (bottom).

It's also demonstrated in our post-cycling SEM analysis (Supplementary Fig. 35, 42, and 43) the sulfur cathodes and lithium anodes maintain a dense, uniform morphology after prolonged cycling. This structural integrity is further confirmed by the stable operation of Li||Li symmetric cells for over 1000 hours and the high-capacity retention (76.5%) of full cells after 900 cycles. Therefore, we conclude that higher power poses a risk to the current cell design. The detailed fading mechanism at higher ultrasonic power may be more complex and warrants future investigation and optimization. Consequently, 180 W was adopted as the optimized parameter in this study to maximize electrochemical gains while ensuring long-term structural stability. We have added the associated discussion to the revised manuscript.

Revisions

Results section:

"To determine the upper limit of the beneficial acoustic field, the ultrasonic power was increased to 210 W. Experimental results indicate that 180 W represents the optimal threshold for the current cell configuration (Supplementary Fig. 45 and 46)."

Supplementary Information section:

"At 210 W, the electrochemical performance noticeably degraded, with the discharge specific capacity at 1.0 C dropping to 738.2 mA h g^{-1} and the capacity decay rate increasing significantly to 0.14% per cycle over 100 cycles (Supplementary Fig. 45). This contrast is stark compared to the 180 W condition, which maintained superior capacity and a low fading rate of 0.027% per cycle over 900 cycles."

"This performance degradation at 210 W is likely attributed to mechanical stress exceeding the binding strength of the electrode components. This is evidenced in the comparison of digital photographs of electrode components dissembled from cycled cells (Supplementary Fig. 46). Specifically, the electrode cycled at 210 W exhibits pronounced surface delamination and pulverization, accompanied by the widespread appearance of yellow patches on the black electrode surface. While at 180 W, the mechanical stress remains within the tolerance of the electrode structure."

7. To explain the Li metal deposition difference, the authors refer to the preferential Li deposition on the higher electric field intense region without the ultrasound. In the case of ultrasound, even though the rate of mixing would be enhanced, the electric field intensity distribution on the electrode surface should remain unchanged. How did the authors account for this when formulating the Li deposition mechanism?

Our response:

We thank the reviewer for this insightful comment regarding the interplay between electric field distribution and the mediating effects of ultrasound. We agree that the initial electric field

distribution, primarily determined by the electrode geometry and surface protrusions, remains largely unchanged upon the application of an external acoustic field. However, the lithium deposition mechanism is not governed solely by the static electric field, but rather by the dynamic competition between the ion reduction rate and local mass transport kinetics (*Adv. Sci.*, **2017**, 4, 1600168). In a conventional electrolyte without the mediation of ultrasonication, Li-ions preferentially deposit at regions with high electric field intensity, such as surface defects. This localization creates high current densities that cause the local Li-ion reduction rate to exceed the diffusion rate, leading to steep concentration gradients and the formation of sharp dendrites. The application of ultrasound fundamentally alters this landscape by providing a high-velocity, continuous supply of Li-ions to the interface. This effectively mitigates the formation of steep concentration gradients and suppresses the localized ion depletion that typically drives dendritic growth (*Nano-Micro Lett.,* **2024***, 16*, 78). In addition, the acoustic field promotes the modification of the Li-ion solvation structure, facilitating Li-ion desolvation and lowering the energy barrier for lithium metal deposition across the entire electrode (*Nat. Commun.*, **2022**, *13*, 172), the system becomes significantly less sensitive to localized "hot spots" of electric field intensity. Furthermore, we have validated that the acoustic field promotes the formation of a more robust, LiF-rich SEI under ultrasound. This chemically stable and mechanically robust layer helps equalize the entry points for Li-ions across the surface, effectively shielding the metal from electric field non-uniformities and promoting smoother deposition. From a theoretical perspective, we employed the electrochemical nucleation-growth equation for Li-ion coupled with the transport equation to analyze the impact of Li-ion flux on its deposition (*J. Electrochem. Soc.*, **2013**, 160, A662). Specifically, the free energy change of the system during nucleation consists of a volumetric term and an interfacial term, as expressed in Equation R1.

$$\Delta G = \frac{4}{3}\pi r^3 \Delta G_v + 4\pi r^2 \gamma \qquad \text{Equation R1}$$

where ΔG_v is the difference in free energy between Li-ion and Li metal anode and γ is interface energy. By differentiating with respect to r, we obtain Equation R2

$$\frac{d\Delta G}{dr} = 4\pi r^2 \Delta G_v + 8\pi r \gamma \qquad \text{Equation R2}$$

Setting Equation (2) to zero gives the critical nucleation radius, r^*.

$$r^* = -\frac{2\gamma}{\Delta G_v} \qquad \text{Equation R3}$$

Substituting Equation R3 into Equation R1 and incorporating the correction for interfacial wettability yields $f(\theta)$ and the critical nucleation barrier, ΔG^*。

$$\Delta G^* = \frac{16\pi\gamma^3}{3(\Delta G_v)^2} * f(\theta) \qquad \text{Equation R4}$$

Furthermore, the Li-ion chemical potential (μ_{Li^+}) depends on both the Li-ion concentration and

the electric field intensity nearby electrode, with its functional form given in Equation R5.

$$\mu_{Li^+} = \mu_{Li^+}^0 + RT\ln\left[c_{Li^+}\right] + zF\phi \qquad \text{Equation R5}$$

And, ΔG_v is defined as the change in free energy per unit volume, which corresponds to the chemical potential difference of Li-ions, as given by Equation R6.

$$\Delta G_v = (\mu_{Li^+} - \mu_{El})V_m \qquad \text{Equation R6}$$

Based on this theoretical framework, even under identical electric field distributions, ultrasound enhances Li-ion transport to increase the local chemical potential near the electrode. This increase in chemical potential elevates the volumetric free energy term (ΔG_v), which reduces the critical nucleation barrier (ΔG^*). This promotes multi-site dense nucleation even under identical electric field distributions, driving lithium deposition toward a dendrite-free morphology. The related discussions have been incorporated in the revised manuscript.

Revisions

Results section:

"While ultrasonication does not fundamentally alter the static electric field distribution dictated by electrode geometry, it profoundly reshapes the dynamic deposition landscape. By sustaining a high Li^+ flux to the interface, modifying the Li-ion solvation structure, and promoting the formation of a robust LiF-rich SEI, the acoustic field homogenizes lithium deposition. These combined effects lower the nucleation barrier and diminish sensitivity to local electric-field heterogeneity, thereby enabling uniform, dendrite-free lithium growth, consistent with the preceding findings."

Supplementary Information section:

"By supplying Li-ions to the electrode interface continuously and at high flux, the acoustic field mitigates localized ion depletion (*Nano-Micro Lett.*, **2024**, 16, 78). In parallel, ultrasound induces modifications to the Li^+ solvation structure, facilitating desolvation and lowering the energy barrier for lithium metal deposition. As a result, the system becomes markedly less sensitive to localized electric-field 'hot spots' (*Nat. Commun.*, **2022**, 13, 172). Moreover, ultrasonication promotes the formation of a chemically stable and mechanically robust, LiF-rich SEI. This interphase helps homogenize Li^+ entry points across the electrode surface, effectively shielding the lithium metal from electric-field non-uniformities and enabling smoother deposition. From a theoretical perspective based on electrochemical nucleation–growth models (*J. Electrochem. Soc.*, **2013**, 160, A662), ultrasound-enhanced Li^+ transport increases the local chemical potential near the electrode, thereby reducing the critical nucleation barrier. This effect favors dense, multi-site nucleation even under identical electric field distributions, ultimately driving lithium deposition toward a dendrite-free morphology (Supplementary Fig. 38)."

8. Figures are mislabeled in the manuscript. Figure 6e in the text talks about XPS but in the Figure it shows SANS data.

Our response:

We appreciate the reviewer's comment and apologize for the error. The correct figure assignment and corresponding description have been updated in the revised manuscript.

Revisions

**Results section:**<br>"As shown in Fig. 6g, the SEI formed under all conditions primarily comprises lithium oxides, hydroxides, carbonate, and lithium fluoride (LiF)."<br>"As shown in Fig. 6e, the R_g value for the lithium layer formed with the 180 W entropic electrolyte is 98.1 Å."

9. The authors do not provide any details on the electrochemical cycling conditions (rate, cycle numbers, voltage etc.) for the ex-situ samples.

Our response:

We appreciate the comment from the reviewer and apologize for the omission of the specific electrochemical conditions for the harvested ex-situ samples. We have updated the manuscript to explicitly state the cycling conditions for all ex-situ characterizations (XPS, SEM, AFM, and SANS). For clarity, the conditions for these samples are summarized below: For the cycled lithium anodes used in XPS, SEM, and AFM analyses, the electrodes were collected by disassembling Li–Li symmetric cells after 100 cycles at 2.0 mA cm^{-2} and 2.0 mA h cm^{-2}, with disassembly conducted at the symmetric cell's near-zero potential state for post-mortem examination. The lithium anodes used for SANS analysis were similarly harvested from symmetric cells after 100 cycles at 2.0 mA cm^{-2} and 2.0 mA h cm^{-2} for structural investigation. Cycled sulfur cathodes for SEM characterization were obtained from Li–S coin cells following 100 cycles at 0.5 C within a voltage window of 1.7–2.8 V, with cells disassembled in the fully charged state (2.8 V). We have ensured that these details are now consistently reflected across the revised manuscript and Supplementary Information.

Revisions

**Results section:**<br>"To elucidate the structure–activity relationship between the ultrasonic field and lithium deposition behavior, the compositions of the SEI were analyzed. Cells were disassembled after 100 cycles at 2.0 mA cm^{-2} and 2.0 mA h cm^{-2}, with disassembly carried out at the

symmetric cell's near-zero potential state for post-mortem analysis. The SEI layers were then characterized by X-ray photoelectron spectroscopy (XPS) combined with argon ion etching. To quantitatively evaluate surface flatness after lithium deposition, the morphology of lithium anodes cycled in symmetric cells for 100 cycles at 2.0 mA cm^{-2} and 2.0 mA h cm^{-2} was characterized by atomic force microscopy (AFM), with electrodes harvested at the symmetric cell's near-zero potential state for post-mortem analysis."

"SANS was employed to characterize the nanostructure of the deposited lithium layer after 100 cycles at 2.0 mA cm^{-2} and 2.0 mA h cm^{-2}, with disassembly carried out at the symmetric cell's near-zero potential state."

Supplementary Information section:

"Supplementary Fig. 35|SEM images of lithium anodes after 100 cycles at 2.0 mA cm^{-2} and 2.0 mA h cm^{-2} in electrolytes subjected to ultrasonic fields of (a) 180 W, (b) 90 W, and (c) 0 W. Cells were disassembled at the symmetric cell's near-zero potential state for post-mortem analysis."

"Supplementary Fig. 42|Front-view SEM images of sulfur cathodes cycled for 100 times at 0.5 C within a voltage window of 1.7–2.8 V, using ultrasonic-field-enabled electrolytes. Cells were disassembled in the fully charged state (2.8 V) for post-mortem analysis."

"Supplementary Fig. 43|Cross-sectional SEM images of sulfur cathodes cycled for 100 times at 0.5 C within a voltage window of 1.7–2.8 V using ultrasonic-field-enabled electrolytes. Cells were disassembled in the fully charged state (2.8 V) for post-mortem analysis."

10. XPS needs additional core level elemental analysis to corroborate with the Li 1s spectra.

Our response:

We agree with the reviewer that corroborating the Li 1s data with other core-level elemental spectra is essential for a comprehensive and accurate characterization of the SEI composition. To address this, we have provided the fine F 1s spectra (presented in the newly added Supplementary Fig. 30) for lithium metal anodes subjected to 100 cycles at 2.0 mA cm^{-2} and 2.0 mA h cm^{-2} under different ultrasonic power levels. The F 1s core-level analysis provides an unambiguous chemical signature for LiF. As shown in the provided spectra, a dominant peak corresponding to Li–F (at approximately 684.8 eV) is observed across all samples. Critically, the relative atomic proportion of LiF increases significantly from 86.6 % in the control group (0 W) to 90.4 % at 90 W, and reaches a maximum of 94.5 % under the optimized 180 W ultrasonic condition.

These F 1s results corroborate the findings from our Li 1s spectra. We have incorporated these spectra and the corresponding analysis into the revised manuscript to provide a more rigorous validation of the SEI chemistry.

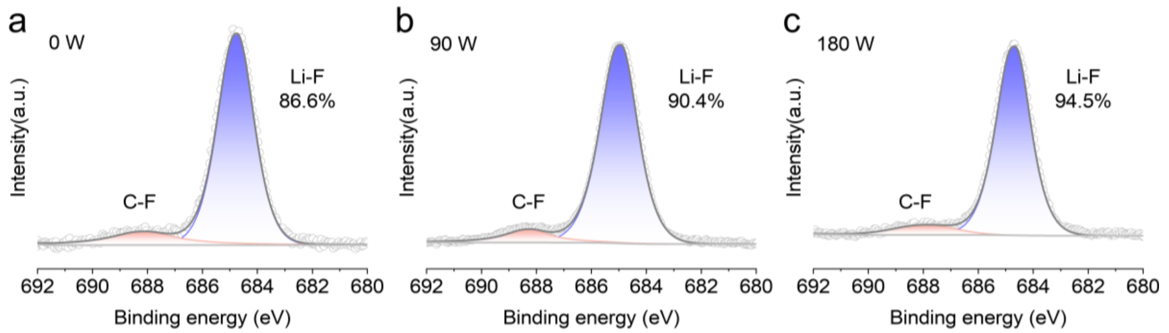

Supplementary Fig. 30|Depth-profiling XPS analysis of F 1s spectra for SEI formed on Li-metal anodes under varying ultrasonication power of (a) 0 W, (b) 90 W, and (c) 180 W . Samples were harvested from Li//Li symmetric cells after 100 cycles at a current density of 2 mA cm^{-2} and an areal capacity of 2 mA h cm^{-2}.

Revisions

Results section:

"This trend is further corroborated by analysis of the high-resolution F 1s core-level spectra of lithium anodes cycled under ultrasonication at different power levels (Supplementary Fig. 30)."

Supplementary Information section:

"To further corroborate the chemical signature of LiF, fine F 1s core-level spectra were analyzed (Supplementary Fig. 30). A dominant peak at approximately 684.8 eV, characteristic of the Li–F bond, is observed across all samples. Quantitative analysis reveals that the relative atomic proportion of LiF increases from 86.6% in the control group (0 W) to 90.4% at 90 W, and reaches a maximum of 94.5% under the 180 W ultrasonic condition. This trend directly supports the Li 1s data, confirming that the ultrasonic field facilitates a LiF-rich SEI layer."

11. The authors do not show the long-term cycling performance of the multi-layer pouch cell. What is the reason behind this?

Our response:

We sincerely appreciate the reviewer's inquiry regarding the long-term cycling of the Ah-level multi-layer pouch cell. We have included data for a multi-layer pouch cell over 3 cycles (Fig. 7i). The Ah-level pouch cell was designed to maximize gravimetric energy density under extreme conditions: ultra-high cathode loading (9.4 mg cm^{-2} per layer), a thin lithium anode (50 μm), and lean electrolyte (E/S ratio: 2.9 μL mg^{-1}). As reported in the literature (*Joule*, **2020**, *4*, 539–554), stable long-term cycling under such conditions is extremely challenging due to rapid electrolyte depletion, high polarization, and limited lithium inventory. Thus, the goal of this test was not cycling life, but to confirm that our acoustic-driven electrolyte mediation

remains effective and scalable under the strictest conditions required to achieve a record energy density of 404.1 Wh kg^{-1}. Consistent with standard practice for high-loading pouch cells (Supplementary Table 1; *Chem*, **2022**, *8*, 3031–3050; *J. Energy Chem.*, **2025**, *106*, 993–1001; *ACS Nano*, **2025**, *19*, 34858−34868; *Adv. Energy Mater.*, **2022**, *12*, 2201912, *etc.*), initial-cycle performance and energy density are the primary metrics for validating feasibility. Simply achieving operation under these conditions demonstrates the efficacy of the acoustic method. For long-term stability and mechanical robustness under ultrasonic irradiation, the single-layer pouch cell (5.2 mg cm^{-2} sulfur loading, 50 μm Li anode, E/S = 7.8 μL mg^{-1}) is more representative, showing 150 cycles with high-capacity retention (Supplementary Fig. 48). Our future work will seek to offer valuable insights into how to extend cell lifespan without compromising its high energy density. These insights will be derived from long-term experimental and theoretical investigations. The related points have been added to the revised manuscript.

Supplementary Table 1|Energy density comparison of multi-layer soft-packaged Li–S pouch cells in this work and literatures.

Ref.	E/S (μL mg^{-1})	Energy density (Wh kg^{-1})	Sulfur content (%)	Cycle number
This work	2.9	404	70	3
5	3.5	353	63	1
6	-	436	63	1
7	-	307	60	5
8	1.2	415	70	1
9	4.5	410	63	1
10	-	470	65	1

Revisions

Results section:

"This stacked pouch cell was designed to maximize gravimetric energy density under extreme conditions, featuring an ultrahigh cathode loading (9.4 mg cm^{-2}), a thin lithium reservoir (50 μm), and a lean electrolyte condition (E/S = 2.9 μL mg^{-1})."

Supplementary Information section:

"To demonstrate the scalability and practical potential of this strategy, an Ah-level stacked pouch cell was assembled using stringent parameter:[4] an ultra-high cathode loading (9.4 mg cm^{-2}), a thin lithium reservoir (50 μm), and a lean electrolyte condition (E/S = 2.9 μL mg^{-1}). While the single-layer pouch cell demonstrates the long-term chemical stability of the system over 150 cycles, The primary objective of this Ah-level prototype was to validate the effectiveness of acoustic-driven mediation under extreme constraints required for state-of-

the-art energy density, in comparison with related prominent works (Supplementary Table 1). Achieving 1.12 Ah and 404.1 Wh kg^{-1} under these conditions confirms that the strategy remains robust even as the E/S ratio and lithium excess are minimized to their practical limits."

4. Dörfler, S. et al. Challenges and key parameters of lithium-sulfur batteries on pouch cell level. Joule 4, 539–554 (2020).
5. Song, Y. W. *et al.* Cationic lithium polysulfides in lithium–sulfur batteries. *Chem* **8**, 3031–3050 (2022).
6. Song, Y. W. *et al.* Reducing the cathode Thiele modulus to promote the discharge capacity of lithium–sulfur batteries. *J. Energy Chem.* **106**, 993–1001 (2025).
7. Xiao, Y. *et al.* Triple-site integrated redox-active metal–organic cages enable complementary acceleration mechanisms for serially enhancing sulfur redox kinetics. *ACS Nano* **19**, 34858–34868 (2025).
8. Xie, Y. *et al*. Semi-flooded sulfur cathode with ultralean absorbed electrolyte in Li–S battery. *Adv. Sci.* **7**, 1903168 (2020).
9. Lin, Y. *et al*. Catalytic Disproportionation for suppressing polysulfide shuttle in Li–S pouch cells: beyond adsorption interactions. *Adv. Energy Mater.* **12**, 2201912 (2022).
10. Chen, J. *et al.* Improving lithium–sulfur battery performance under lean electrolyte through nanoscale confinement in soft swellable gels. *Nano Lett.* **17**, 3061–3067 (2017).

12. Another questionable analysis is their energy density reporting and calculation for the Li-S pouch cell. The authors do not provide any information on the thickness of the Li metal used in the pouch cell. Thin Li metal is critical for high energy density (low N/P ratio). Assuming the loading of S cathode being 1.8-2.0 mg/cm^2 of S loading, Li metal has to be extremely thin in addition to the lean electrolyte loading. What thickness of Li metal and S loading in cathode for pouch cells did the authors utilize? The authors did not include the mass of the Al-plastic film for energy density calculation. This weight should not be excluded for cell level energy density calculation. Right now, the reported energy density is giving the impression that the energy density is at the cell level. The authors should explicitly mention it in the manuscript including in the abstract that the energy density does not take into consideration the mass of the cell housing. Or, include the mass of the cell housing and revise the energy density of the cell.

Our response:

We sincerely appreciate the reviewer's rigorous assessment of our energy density reporting. Accurate disclosure of cell parameters and weight contributions is essential for evaluating the practical viability of high-energy-density Li–S batteries. To address the specific questions

regarding cell configuration, we utilized an ultra-high cathode loading of 9.4 mg cm^{-2} and a thin lithium anode with a thickness of 50 μm for the multi-layer stacked pouch cell. Furthermore, the cell was operated under a lean electrolyte condition with an E/S ratio of 2.9 μL mg^{-1}. These parameters represent the stringent conditions required to push the limits of gravimetric energy density in a practical format. Regarding the energy density calculation, the reported value of 404.1Wh kg^{-1} was indeed calculated based on the total mass including the cathode, electrolyte, separator, anode, and current collectors (Housing-free). We adopted this methodology to ensure a fair and consistent comparison with values widely reported in current literature, which often utilize this "housing-free" basis for bench-marking performance (*Nat. Catal.*, **2023**, *6*, 1073–1086; *Nat. Chem.*, **2025**, *17*, 614–623; *Nat. Nanotechnol.*, **2021**, *16*, 166–173; *J. Am. Chem. Soc.* **2025**, *147, 33*, 30042–30049; *Adv. Energy Mater.* **2025**, *15*, 2405461). However, we fully agree with the reviewer that including the cell housing is necessary for a complete cell-level analysis and to prevent any potential misinterpretation. Consequently, we have now provided a comprehensive weight breakdown for every component in the stacked cell, including the Al-plastic film, as shown in the newly added Supplementary Fig. 54. When accounting for the full mass of the cell housing, the energy density is 247.0 Wh kg^{-1}. We have revised the manuscript and the abstract to explicitly differentiate between the energy density calculated at the stack level (for literature comparison) and the full cell level (including housing). This transparency ensures that readers can accurately evaluate the system's performance within the context of both fundamental research and practical application.

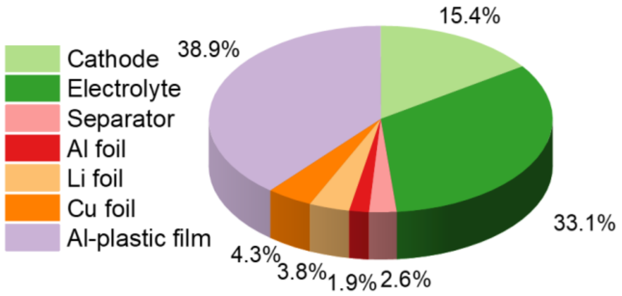

Component	Weight (g)
Cathode	1.5
Electrolyte	3.21
Separator	0.25
Al foil	0.18
Li foil	0.37
Cu foil	0.42
Al-plastic film	3.78
Total weight	9.72

Supplementary Fig. 54|Weight breakdown of the Ah-level stacked pouch cell. The pie chart and accompanying table specify the gravimetric contribution of each constituent, used for calculating the total cell energy density (with the weight of the Al-plastic film considered).

Revisions

Abstract section:

"The designed entropy-driven electrolyte at a lean dosage of 2.9 μL mg^{-1} readily enables a 1.1 Ah-level pouch cell with a delivered energy density of 404.1 Wh kg^{-1} (Housing-free)."

Results section:

"The total energy of the battery reached 2.4 Wh, translating to a gravimetric energy density

of 404.1 Wh kg^{-1} based on the stack mass (excluding the Al-plastic film housing, Supplementary Fig. 53). To provide a strictly practical evaluation, the energy density was also calculated using the total cell weight including the Al-plastic film. (Detailed weight breakdown in Supplementary Fig. 54)."

Supplementary Information section:

"A comprehensive mass breakdown of all components in the stacked cell, including the Al–plastic laminate film, is provided in Supplementary Fig. 54. When the full mass of the cell housing is considered, the resulting gravimetric energy density is 247.0 Wh kg^{-1}."

Reviewer 1 (Comments to the Author):

Previous issues have been well addressed and I think the current version is acceptable.

Response: Thank you for your approval of our work.

Reviewer 2 (Comments to the Author):

I am pleased with the changes made and it can be published in the current form.

Response: Thank you for your approval of our work.

Reviewer 3 (Comments to the Author):

The authors modified the manuscript based on the previous round of comments, replacing the problematic "high-entropy" term with "acoustic-induced entropy driven state", which is a good start. However, their claims are still rationalized based on equilibrium excess-entropy framework and utilizing structural-chemical proxies to imply entropy increase. Justification of entropy increase in this work still requires concrete measurement related to thermodynamic parameters (e.g., calculation of entropy term, entropy driven free energy decomposition, explicit excess entropy calculation from RDFs etc.). No explicit measurement of the ΔS term change upon ultrasonication is present in the work right now. This is necessary because the entropy change is the central assertion of this work.

Response:

Thank you for the constructive comments on our manuscript. The manuscript has been further revised to address all additional concerns, with particular emphasis placed on providing concrete and explicit justification for the observed increase in entropy. We sincerely appreciate your insightful suggestions, which have substantially enhanced the scientific rigor of this work.

1. ***The claim of entropy increase is rationalized by observations through Raman and Ab initio molecular dynamics simulations: demonstration of disrupted ion solvation shells and liberated solvation molecules. These analyses are structural proxies but not actual entropy measurements. To claim entropy increase, the authors need actual entropy measurement. Furthermore, if solvent molecules are released from the solvation shells, it would imply that the coordination number has decreased? Since the release of solvent molecules from the solvation shells is argued for a cause of enhanced entropy, the reviewer is curious if the authors think that solutions with smaller coordination number of the solvated species would be inherently more entropic than those of higher coordination numbers.***

Response:

We appreciate the your comment regarding the explicit substantiation of our "entropy-driven electrolyte" concept. We fully acknowledge that while operando Raman spectroscopy and AIMD simulations provide compelling structural evidence of solvation shell disruption, a rigorous claim of entropy increase necessitates a statistical transition from qualitative descriptions of "disorder" to quantitative confirmation. To move beyond these structural proxies and address the reviewer's request for actual entropy measurements, we have performed a statistical thermodynamic analysis focusing on the two-body correlation contribution to the excess entropy (s_2), often termed "**structural entropy**" (*Nature Mater.,* **2011**, 10, 512-520).

Before addressing the detailed statistical entropy variations, we would like to simultaneously respond to the second comment, which concerns the characterization of entropy in a cavitating,

non-equilibrium electrolyte. In particular, we aim to justify the adoption and use of the so-called "structural entropy".

We sincerely appreciate the reviewer's insightful comment, which pinpoints a fundamentally interesting and challenging issue regarding the characterization of entropy in systems not in equilibrium. We agree that, in a strictly classical sense, the full excess entropy framework is best defined for equilibrium states. However, the characterization of such states, particularly under mechanical driving, remains a subject of significant ongoing research. To address this rigorously, we have followed the protocol established in the literature where the two-body correlation contribution to excess entropy (s_2), also known as "structural entropy", is utilized as a robust predictor for dynamics in non-equilibrium states (*Nature Mater.*, **2010**, 9, 324-331).

The efficacy of s_2 in characterizing non-equilibrium systems stems from its reliance on two-body interparticle correlations (*Nature Mater.,* **2011**, 10, 512-520). As a geometric consideration, s_2 serves as a robust structural metric that evaluates a system independently of its equilibrium state. For example, it has been verified that structural relaxation times in strongly sheared, non-equilibrium liquids have been proven to collapse onto the same s_2 scaling curve as their equilibrium counterparts, regardless of whether the system is driven into nonlinear regimes. This demonstrates the success of applying s_2 to serve as a fundamental structural predictor even when a system is far from its quiescent state (*Proc. Natl. Acad. Sci.*, **2018**, 115, 87-92). Furthermore, experimental work on amorphous solids under nonstationary, oscillatory plastic deformation, a condition involving intense mechanical driving, has also shown that the time-dependent structural entropy can be used to accurately deduce the dynamical relaxation history of the material (*Proc. Natl. Acad. Sci*, **2020**, 117, 11887-11893).

We agree with the reviewer that directly applying the full excess entropy concept to a cavitating non-equilibrium electrolyte requires caution and analysis. Consequently, to maintain scientific rigor and align with the accepted concepts in the literature, we have refined our manuscript to focus specifically on the two-body structural entropy (s_2) rather than the full thermodynamic excess entropy. This ensures our argument remains grounded in a framework proven effective for describing nonequilibrium systems.

Consequently, the structural entropy (s_2) was quantified from the radial distribution functions g(r) obtained via AIMD simulations in this work, according to:

$$s_2 = -2\pi\rho k_B \int_0^\infty [g(r)\log(g(r)) - g(r) + 1] r^2\, dr$$

This two-body excess entropy can be further decomposed into partial contributions corresponding to the Li^+-DME and Li^+-DOL correlations (*Nat. Commun.*, **2018**, 9, 3272). Our analysis indicates that upon ultrasonication, the structural entropy of both Li^+-DME and Li^+-DOL interactions increases. Specifically, the Δs_2 for Li^+-DME shows a larger increase of 6.23 k_B, while Li^+-DOL increases by 2.42 k_B. The heightened sensitivity of the DME component is well aligned with existing literature and is attributed to its bidentate coordination nature. Acoustic disruption of a chelated DME solvation shell is therefore likely accompanied by enhanced configurational

degrees of freedom compared to the monodentate DOL species (*Nat. Commun.,* **2026**, 17, 590; *Chem. Sci*., **2024**, 15, 19805-19819).

This quantified increase in structural entropy unequivocally validates that the exogenous ultrasonic field disrupts liquid-structure correlations, effectively shifting the electrolyte from a structured solvated state toward a more randomized, "gas-like" configurational state. This transition from an ordered to a disordered state via solvent liberation is a hallmark of an entropy-driven process, and with this explicit statistical evidence, we believe the term "acoustic-induced entropy-driven" concept is now justified.

Regarding the reviewer's inquiry into the relationship between coordination number and entropy, we think that a reduction in coordination number does not inherently imply higher entropy and should be evaluated with caution. A primary counter-example is found in high-concentration electrolytes, where a scarcity of solvent molecules leads to a low coordination number (*ACS Energy Lett.*, **2024**, 9, 373–380). However, high-concentration electrolytes are characterized by tight packing and strong correlations that lock solvent molecules which certainly cannot be classified as a high entropy state despite the low coordination (*Science,* **2015**, 350, 938-943). In stark contrast, the decrease in coordination number observed in our acoustically induced system arises from an external field that actively disrupts rigid ion–solvent correlations, effectively transforming a "structured liquid" into a more "randomized liquid" and thereby enhancing the structural entropy. Thus, a smaller coordination number may correspond to a higher entropic state when it arises from structural disruption and randomization. In view of this, we suggest that caution should be exercised when interpreting this relationship based on specific cases.

The related discussions have been incorporated into the revised manuscript.

Revisions

Results section:

"To quantitatively substantiate this, structural entropy (s_2) was derived from radial distribution functions, g(r), using AIMD simulations and partitioned into specific Li^+-DME and Li^+-DOL correlations.[42] The data reveals that ultrasonication induces an increase in structural entropy for both interactions. The effect is more pronounced in the Li^+-DME component (Δs_2 = 6.23 k_B) relative to Li^+-DOL (2.42 k_B). This heightened sensitivity in DME is consistent with its bidentate coordination, as the acoustic disruption of the chelated solvation shell is likely accompanied by enhanced configurational degrees of freedom than the monodentate DOL.[43,44] This quantified increase in structural entropy unequivocally validates that the exogenous ultrasonic field disrupts liquid-structure correlations, effectively shifting the electrolyte from a structured solvated state toward a more randomized, "gas-like" configurational state."

Methods section:

"Structural entropy analysis: The two-body correlation contribution to the excess entropy (s_2), also referred to as "structural entropy," is employed as a predictor of dynamic behavior in

non-equilibrium systems.[58–61] In this work, s_2 is quantified from the radial distribution functions g(r) obtained via AIMD simulations, according to:

$$s_2 = -2\pi\rho k_B \int_0^\infty [g(r)\log(g(r)) - g(r) + 1]r^2\, dr$$

where ρ denotes the number density, k_B is the Boltzmann constant, and r represents the separation distance."

2. ***The work by Michaelidis et al referenced in the response in support of exogenous field effect to change entropy is an entirely different perturbation method. It can be best explained as a "conceptual inspiration" but not as a near-confirmation of their observations. Furthermore, the Frank and Evans paper they cited is about entropy in mixtures/solutions and interpretation of entropy data in condensed system. It does not specifically establish ultrasound disruption of solvation shells leads to entropy increase. The bottom line is the claim of thermodynamic entropy increase requires actual quantification of the entropy. They currently lean towards an equilibrium excess-entropy framework-based argument that does not directly apply to a cavitating non equilibrium electrolyte.***

Response:

We sincerely appreciate your professional insights. We acknowledge the reviewer's point that the work by Michaelides et al. and the paper by Frank and Evans serve as essential conceptual benchmarks rather than direct confirmations of our specific observations. While those studies establish the physical possibility of utilizing exogenous fields to govern reactivity through entropic shifts and provide a basis for analyzing solvation identity via entropy, we agree that a definitive claim of entropy increase in our system requires direct quantification. Furthermore, we value the reviewer's observation that simply applying an equilibrium-based full excess-entropy framework to a cavitating, non-equilibrium electrolyte necessitates justification.

To address these concerns, we have transitioned our analysis from qualitative structural descriptions to a quantitative statistical framework focusing on structural entropy, s_2, which serves as a robust characterization of the system's disorder even under mechanical driving, as justified and detailed in the response to comment 1.

With this explicit statistical evidence and refined theoretical framework, we believe the "acoustic-induced entropy-driven" concept is fully justified.

3. ***As for the rotational mass transfer model, the authors explain their rationalization of selecting the model of choice. However, the original criticism still holds. How does the model best reflect the mass transport modification induced by the random nature of bubble formation/collapse?***

Response:

We appreciate your instructive follow-up regarding the stochastic nature of bubble dynamics versus our choice of a rotational model. While we acknowledge that individual ultrasonic cavitation events, including bubble formation and collapse, are inherently random at the microscale, their collective behavior in an ensemble leads to well-defined macroscopic phenomena. It has been demonstrated in acoustics literature that the microstreaming generated by these random bubbles converges into an overall steady-state velocity distribution. Even though individual bubble structures are rapidly morphing, time-averaged result reveals macroscopic features that converge toward a steady-state velocity distribution, providing a stable basis for fluid dynamic modeling (*J. Acoust. Soc. Am.,* **1958**, 30, 329-339; *High-intensity ultrasonic fields*, **2013**, Springer Science & Business Media; *Physics of Fluids*, **2025**, 37, 023312).

Furthermore, the bounded geometry of the battery cell plays a role in regularizing these motions. In such a confined environment, incident ultrasonic waves reflect off the boundaries, interfering with incoming waves to form acoustic standing waves. Our simulations confirm a steady 2D in-plane acoustic pressure distribution within the cell domain (Supplementary Fig. 59a). These standing waves further dictate the behavior of the cavitation bubbles through Bjerknes radiation forces, which would effectively guide bubbles trajectories, preventing the chaotic "wandering" (*Fields of force: supplementary lectures, applications to meteorology; a course of lectures in mathematical physics delivered*, **1906**, Columbia University Press). This phenomenon further promotes the formation of steady-state velocity distribution characterized by macroscopic regularity, justifying our statistical approach.

In our multi-physics model, we reconcile this microscale randomness by coupling the bubble-generated impacts as an averaged term into the Navier-Stokes equations to solve for the velocity distribution. The results indicate that the electrolyte domain reaches a converged steady state dominated by a uniform vortex flow, which aligns with the circulating vortex patterns characteristic of acoustic streaming described in literature (*Handbook of Ultrasonics and Sonochemistry,* **2016**, Springer). This is evidenced in the uniform velocity distribution observed in our simulation results (Supplementary Fig. 59b, c). By incorporating this steady-state velocity as a "background field" within the convective source term of our electrochemical mass transport model, we are able to derive the rotational mass transfer trajectories for lithium ions. We believe this treatment effectively bridges the gap between microscale stochastic events and the organized macroscopic transport that dictates the battery's electrochemical response.

Finally, we fully concur with the reviewer that the fluid dynamics mediated by ultrasonication represent a complex multiscale problem. There is an evident and pressing need for continued innovation in multiscale modeling approaches capable of providing advanced characterization of these dynamic behaviors across a wider range of length scales with enhanced accuracy and reduced computational cost. Such advancements will undoubtedly deepen the scientific community’s understanding of how complex external fields influence and enhance electrochemical processes.

The related discussions have been updated into the revised manuscript.

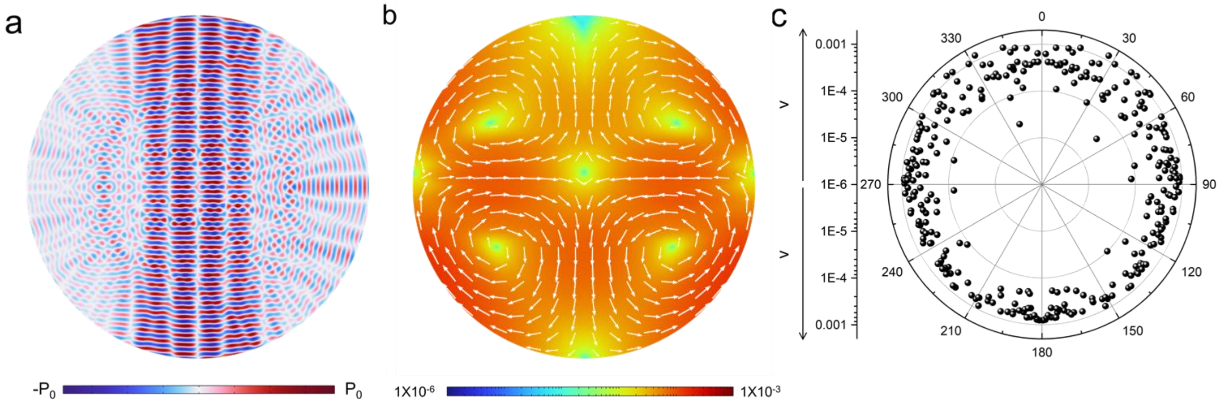

Supplementary Fig. 59| (a) The 2D in-plane distribution of acoustic pressure in the calculated domain. (b) The corresponding spatial distribution of the normalized fluid velocity, as represented by white arrows. (c) Polar coordinate statistics of the normalized velocity, in which the radial direction corresponds to the velocity magnitude and the angular coordinate represents the deflection angle, θ.

Revisions

Methods section:

"It should be noted that, while individual ultrasonic cavitation events, including bubble formation and collapse, are inherently random at the microscale, their collective behavior in an ensemble leads to well-defined macroscopic phenomena. Such microscale randomness is reconciled in this model by coupling the bubble-generated impacts as an averaged term into the Navier-Stokes equations to solve for the velocity distribution as demonstrated in Supplementary Fig. 59."

Supplementary Information section:

"Even though individual bubble structures are rapidly morphing, time-averaged result reveals macroscopic features that converge toward a steady-state velocity distribution, providing a stable basis for fluid dynamic modeling.[5-7] Furthermore, the bounded geometry of the battery cell plays a role in regularizing these motions. In such a confined environment, incident ultrasonic waves reflect off the boundaries, interfering with incoming waves to form acoustic standing waves. This is confirmed from a steady 2D in-plane acoustic pressure distribution within the cell domain from the simulation (Supplementary Fig. 59a). These standing waves further dictate the behavior of the cavitation bubbles through Bjerknes radiation forces, which would effectively guide bubbles trajectories, preventing the chaotic "wandering".[8] This phenomenon further promotes the formation of steady-state velocity distribution characterized by macroscopic regularity. As a result, the microscale randomness is reconciled in this work by coupling the bubble-generated impacts as an averaged term into the Navier-Stokes equations to solve for the velocity distribution. The results indicate that the electrolyte domain reaches a converged steady state dominated by a uniform vortex flow, which aligns with the circulating

vortex patterns characteristic of acoustic streaming.[9] This is evidenced in the uniform velocity distribution observed in the simulation results (Supplementary Fig. 59b, c)."

4. ***For multi-layer pouch cell, the authors state that "Simply achieving operation under these conditions demonstrates the efficacy of the acoustic method." Is there an initial cycle performance of the similar pouch cell without the acoustic in the manuscript to reaffirm this statement?***

Response:

We appreciate your insightful suggestion to include a direct comparison for the high-loading, multi-layer pouch cell configuration. To further substantiate the efficacy of the acoustic-induced entropy-driven strategy, we have conducted a control experiment using a multi-layer pouch cell fabricated under identical parameters, including an equivalent sulfur loading of 1.05 g, a lean electrolyte-to-sulfur (E/S) ratio of 2.9 μL mg^{-1}, but utilizing a conventional electrolyte without the acoustic-induced treatment.

As illustrated in the newly provided comparison (Supplementary Fig. 55), the control pouch cell exhibits a markedly reduced discharge capacity, unstable discharge plateau, and consequently lower housing-free energy density compared to the cell employing the entropy-driven electrolyte (Fig. 7h). This comparison clearly demonstrates the potential of the acoustically induced approach as a critical enabler for the operation of Li–S batteries under demanding, high-mass-loading conditions. The control data and the corresponding discussion have been incorporated into the revised manuscript.

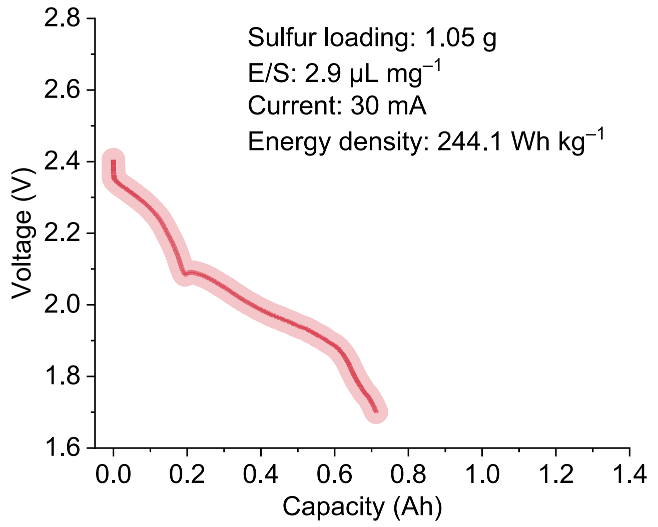

Supplementary Fig. 55|Discharge curve of a high-loading (1.05 g sulfur) multilayer Li–S pouch cell operated without ultrasonic mediation, delivering a housing-free gravimetric energy density of 244.1 Wh kg^{-1}.

Revisions

Results section:

"The efficacy of the acoustically induced, entropy-driven strategy is further substantiated through comparison with a control stacked pouch cell prepared under identical parameters but employing a conventional electrolyte without acoustic treatment, as shown in Supplementary Fig. 55. The control pouch cell exhibits a markedly reduced discharge capacity, an unstable discharge plateau, and consequently a lower housing-free energy density compared to the cell utilizing the entropy-driven electrolyte. These results clearly demonstrate the potential of the acoustically induced approach as a critical enabler for the operation of Li–S batteries under demanding, high-mass-loading conditions."
